# pRB-Depleted Pluripotent Stem Cell Retinal Organoids Recapitulate Cell State Transitions of Retinoblastoma Development and Suggest an Important Role for pRB in Retinal Cell Differentiation

**DOI:** 10.1093/stcltm/szac008

**Published:** 2022-03-23

**Authors:** Agata Rozanska, Rodrigo Cerna-Chavez, Rachel Queen, Joseph Collin, Darin Zerti, Birthe Dorgau, Chia Shyan Beh, Tracey Davey, Jonathan Coxhead, Rafiqul Hussain, Jumana Al-Aama, David H Steel, Nissim Benvenisty, Lyle Armstrong, Manoj Parulekar, Majlinda Lako

**Affiliations:** Biosciences Institute, Newcastle University, Newcastle upon Tyne, UK; Biosciences Institute, Newcastle University, Newcastle upon Tyne, UK; Biosciences Institute, Newcastle University, Newcastle upon Tyne, UK; Biosciences Institute, Newcastle University, Newcastle upon Tyne, UK; Biosciences Institute, Newcastle University, Newcastle upon Tyne, UK; Biosciences Institute, Newcastle University, Newcastle upon Tyne, UK; Biosciences Institute, Newcastle University, Newcastle upon Tyne, UK; Biosciences Institute, Newcastle University, Newcastle upon Tyne, UK; Biosciences Institute, Newcastle University, Newcastle upon Tyne, UK; Biosciences Institute, Newcastle University, Newcastle upon Tyne, UK; Faculty of Medicine, King Abdulaziz University, Riyadh, Saudi Arabia; Biosciences Institute, Newcastle University, Newcastle upon Tyne, UK; The Azrieli Center for Stem Cells and Genetic Research, The Hebrew University of Jerusalem, Jerusalem, Israel; Biosciences Institute, Newcastle University, Newcastle upon Tyne, UK; Birmingham Women’s and Children NHS Foundation Trust, Birmingham, UK; Biosciences Institute, Newcastle University, Newcastle upon Tyne, UK

**Keywords:** human embryonic stem cells, human induced pluripotent stem cells, retinoblastoma, retinoma, single-cell RNA-Seq, retinal organoids

## Abstract

Retinoblastoma (Rb) is a childhood cancer of the developing retina, accounting for up to 17% of all tumors in infancy. To gain insights into the transcriptional events of cell state transitions during Rb development, we established 2 disease models via retinal organoid differentiation of a pRB (retinoblastoma protein)-depleted human embryonic stem cell line (RB1-null hESCs) and a pRB patient-specific induced pluripotent (iPSC) line harboring a *RB1* biallelic mutation (c.2082delC). Both models were characterized by pRB depletion and accumulation of retinal progenitor cells at the expense of amacrine, horizontal and retinal ganglion cells, which suggests an important role for pRB in differentiation of these cell lineages. Importantly, a significant increase in the fraction of proliferating cone precursors (RXRγ^+^Ki67^+^) was observed in both pRB-depleted organoid models, which were defined as Rb-like clusters by single-cell RNA-Seq analysis. The pRB-depleted retinal organoids displayed similar features to Rb tumors, including mitochondrial cristae aberrations and rosette-like structures, and were able to undergo cell growth in an anchorage-independent manner, indicative of cell transformation in vitro. In both models, the Rb cones expressed retinal ganglion and horizontal cell markers, a novel finding, which could help to better characterize these tumors with possible therapeutic implications. Application of Melphalan, Topotecan, and TW-37 led to a significant reduction in the fraction of Rb proliferating cone precursors, validating the suitability of these in vitro models for testing novel therapeutics for Rb.

## Graphical Abstract

**Figure:**
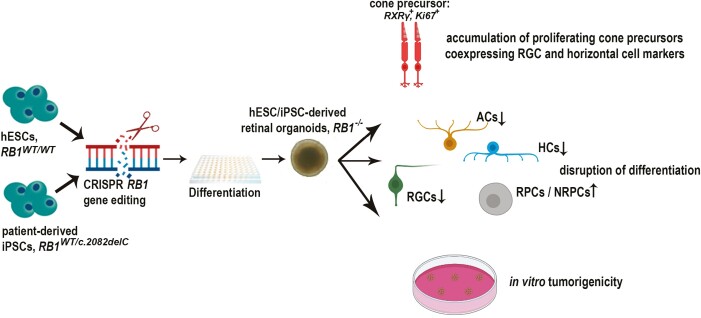

## Introduction

Retinoblastoma (Rb) is a malignant tumor of developing retina with a typical presentation occurring during the first 5 years of life.^1^ Diagnosis above the age of 6 years is extremely rare^2,3^ and more so in adults.^4^ Confinement of the malignant mass to the eye in early stages and prompt diagnosis in developed countries result in high cure rates up to 95%. Spread beyond the eye is associated with poor prognosis, lowering the cure rates below 5%-10%.^2,5^ Despite the progress in treatments, Rb survivors are left with impaired vision having negative effect on correct motion processing, depth perception, and judging distances. Patients can suffer hearing loss, cataracts, cosmetic deformities, and impaired neurocognitive development.^6,7^

Biallelic *RB1* gene inactivation is the causative factor in 96% cases of this retinal malignancy,^8^ that leads to formation of noninvasive tumors, known as retinomas.^9^ Singular cells of those retinal lesions undergo malignant transformation and present as bilateral or unilateral disease.^10^ Despite numerous studies, it remains unclear why retinal tissue is selectively susceptible to malignant transformation in the presence of heterozygous germline mutations in *RB1*, which account for 40% of cases and are transmitted as an autosomal dominant trait, with 90% penetrance.^11^

Incomplete understanding of Rb biology relates to the lack of a representative animal model as well as the complex expression pattern of pRB during retinal development in a cell type and species-specific manner,^12,13^ which has led to a number of proposed candidate cells of origin.^14^ Mice with *Rb*^*−/−*^ germline mutation are embryonic lethal causing placental failure^15^; however, when the wild-type placenta is provided either via tetraploid aggregation or genetic approaches, mice die shortly after birth.^16^ Mouse models of the retinal tumors with biallelic *Rb1* loss in combination with *Rbl1*^*−/−*^, *Rbl2*^*−/−*^, *Cdkn1b*^*−/−*^ or *Mycn* overexpression are characterized by the expansion of retinal interneurons.^17-20^ However, recent studies strongly point to the post-mitotic cone precursor^21,22^ at a maturing stage of development^23^ as the cell type of origin for human Rb.

Genetic and phenotypic divergence between spontaneous human Rb and induced mouse retinal tumors can be bypassed by taking advantage of recent developments in somatic cell-induced reprogramming,^24,25^ CRISPR/Cas9 gene editing^26-29^ and 3D retinal organoid generation from pluripotent stem cells.^30-37^ Zeng et al^38^ and Vincent et al^39^ reported generation of Rb patient-specific induced pluripotent stem cell (iPSC) lines heterozygous for *RB1* gene from skin fibroblasts and orbital adipose tissue, respectively. CRISPR/Cas9 generated *RB1*^*−/−*^ human embryonic stem cells (hESCs) were also generated and reported to form neural-enriched teratomas with characteristics similar to Rb and proposed as a tool in disease modeling and drug screening.^26^ Zheng et al^40^ showed that silencing of *RB1* in H9 hESC-derived retinal organoids promoted S-phase entry, apoptosis and reduced the number of photoreceptors, retinal ganglion, and bipolar cells. Yet, *RB1* knockout in retinal organoids did not trigger tumorigenesis in vitro or in vivo. Facilitating tumorigenesis in a developing and relatively complex tissue-like retina requires a number of factors coming together at a specific time point^41^ that might have not occurred in the presented model in which differentiation of retinal organoids was not carried beyond day 90.

While this manuscript was under preparation, Liu et al^42^ also reported development of an hESC line with biallelic mutation (p.R320X) in the *RB1* gene, which upon differentiation generated organoids with properties consistent with tumorigenesis in vivo and pointed to Arrestin3^+^ maturing cone precursors as cell of origin for Rb. However, this study did not consider the patient-specific genetic background, which can influence Rb development. To address this gap, a very recent study reported the generation of iPSCs from 15 patients with germline *RB1* mutations, their differentiation to retinal organoids up to day 45 in culture and intravitreal injections into immunocompromised mice, showing formation of tumors which are indistinguishable from human Rb tumors.^43^ This seminal study provides compelling support for application of patient-specific RB1 organoids for drug discovery, repurposing, and testing; however to enable this, a detailed characterization of RB1 organoid models in vitro is necessary and forms the main topic of this manuscript. To this end, we report the generation of a patient-specific iPSC model (c.2082delC) encompassing the heterozygous (*RB1*^*+/−*^), homozygous (*RB1*^*−/−*^), and fully corrected isogeneic control. We compare the retinal organoids developed from the patient-specific iPSC model to the engineered *RB1* knockout in hESC line (RB1-null^26^) along the differentiation trajectory. Our data show a significant enrichment in proliferating cone precursors (RXRγ^+^Ki67^+^) within the RB1 organoids, which display key histological and tumorigenic features. Both models were characterized by pRB depletion and accumulation of retinal progenitor cells at the expense of amacrine, horizontal and retinal ganglion cells, which in the absence of significant cell death, suggests an important role for pRB in differentiation of these cell lineages. Application of Melphalan, Topotecan, and TW-37 led to a significant reduction in proliferating cone precursors in both pRB-depleted models, validating their use for testing current and new Rb treatments.

## Materials and Methods

### Generation of Rb Patient-specific iPSC Lines

Informed consent was received from the parents of Rb patient (with bilateral, familial Rb; heterozygous mutation c.2082delC in *RB1*; female, 3 years old) and peripheral blood sample obtained with approval from the Children with Cancer and Leukaemia group Tissue Bank (ethics permission 18/EM/0134). Induced pluripotent stem cell lines were generated from erythroblast population expanded from isolated peripheral blood mononuclear cells (PBMCs) and transduced with Sendai viral vectors (CytoTune-iPS 2.0 Sendai Reprogramming Kit, ThermoFisher Scientific), expressing 4 reprogramming factors OCT4, SOX2, KLF4, and c-MYC according to the published protocol.^44^ The *RB1* gene mutation c.2082delC was confirmed using PCR with primers F: TGTGAACGCCTTCTGTCTGA, R: AGTAGGGAGGAGAGAAGGTGA, followed by Sanger sequencing.

### Generation of Wild-Type Isogenic and Homozygous Mutant iPSC Lines for c.2082delC Mutation

The c.2082delC cell line was electroporated using a 4D-Nucleofector (Lonza) machine to facilitate the transfer of gRNA/Cas9 and ssODN directly to the nucleus. gRNA and ssODN were designed with Benchling tools (Supplementary Fig. S12). gRNA was produced with GeneArt Precision gRNA Synthesis Kit (Thermo Fisher Scientific, A29377), while ssODN was synthesized by Thermo Fisher Scientific. TrueCut Cas9 Protein V2 (Thermo Fisher Scientific, A36499) was incubated with gRNA for 10 minutes, then mixed with ssODN in 100 µL nucleofection solution. Cells were grown for the last 8 hours prior to nucleofection in the presence of nonhomologous end joining inhibitor (SCR7 pyrazine, Sigma, SML1546, at concentration 10 μM) to encourage homology directed repair (HDR). StemPro Accutase (Gibco, A11105-01) was used to dissociate iPSCs and 8 × 10^5^ cells were used for nucleofection. The ratio of Cas9 Nuclease to gRNA/ssODN used was (1:2). The ribonucleoprotein complexes alongside ssODN were delivered to the cells using CB150 program. Instantly after electroporation, the cells were mixed with 500 L mTeSR1 Plus medium (Stem Cell Technologies, 05826) containing 10 μM ROCK inhibitor (Y27632, Tocris) and SCR7 pyrazine (10 μM). Cell suspension was distributed among at least 5 Matrigel (Corning, 354230) coated 10 cm plates supplemented with 4 mL of TeSR1 Plus medium (Stem Cell Technologies, 05826), 10 μM ROCK inhibitor (Y27632, Tocris), and SCR7 pyrazine (10 μM). Medium was replaced 48 hours post-seeding with mTeSR1 Plus only and continued to be changed every second day. Colonies were picked manually around 10 days later and grown in 24-well plates to be assessed for gene editing with restriction enzyme assay (*BspH*I, NEB, R0517L). Selected clones were sequenced to confirm the introduction of the mutation to the wild-type allele or obtaining isogenic wild type (F: ATTCCCACAGTGTATCGGCT, R: AAATGGTAGCCAAAAAGTGAACA). Five off-target sites, with the lowest number of mismatches, identified with Off-Spotter were sequenced to exclude additional changes to the genome. Primers used to obtain PCR products enclosing off-target sites and the sequencing result are listed in Supplementary Table S2.

### Culture of Pluripotent Stem Cell Lines

H9 control and RB1-null hESC lines, the RB1-patient-specific iPSC lines, and the control, were cultured in mTeSR1 Plus medium (Stem Cell Technologies, 05826) on Matrigel (Corning, 354230) coated plates (TPP) under 5% CO2 in a 37 °C humidified incubator. Medium was changed every second day. Cells were passaged twice per week in a ratio of 1:6 with Versene, EDTA (Lonza, 17-711E).

### Differentiation of Pluripotent Stem Cells to Retinal Organoids

Retinal organoids were generated according to the modified protocol described in Hallam et al.^34^ Briefly, cells grown in 6-well plates were dissociated at 90% confluence with Accutase (Thermo Fisher Scientific, Waltham, MA) and seeded at a concentration of 7000 per well in 96-well Lipidure-coated U-bottom plates (Amsbio, AMS.LCP-A-U96) in 100 μL of mTeSR1 (Stem Cell Technologies, 85851) with 10 μM ROCK inhibitor (Y27632, Tocris). After 48 hours, 200 µl of differentiation medium was added to each well [45% IMDM, 45% HAM’s F12, 10% KOSR, 1% GlutaMAX, 1% Chemically Defined Lipid Concentrate (11905031), 1% Pen/Strep (Thermo Scientific), and 225 µM 1-thioglycerol (M6145; Sigma)]. Medium was half-replaced every 2 days, up to day 6, when it was once supplemented with recombinant human BMP-4 (Gibco, PHC9534) at concentration 1.5 nM, followed by half-replacements every 3 days. At day 18, maintenance medium was introduced [DMEM/F12, 10% FBS, 1% GlutaMAX, 1% N2, 1% Pen/Strep, 0.1 mM Taurine, 40 ng/mL T3 (Sigma, T6397), 0.25 µg/mL Fungizone, and 0.5 µM Retinoic Acid (Sigma, R2625); the latter was added fresh up to day 120 of differentiation]. Media changes were performed 3 times per week.

### Immunohistochemistry

The collection of retinal organoids was carried out at days 35, 90, and 150. The retinal organoids were briefly washed with PBS and fixed in 2% paraformaldehyde for 15 minutes. After removal of fixative and 3 subsequent washes with PBS, organoids were placed in 30% sucrose overnight, then frozen in OCT-filled moulds. Approximately 12 μm slices were sectioned on a Leica cryostat (CM1850), then blocked and permeabilized for 1 hour, in 0.3% Triton X-100 (Sigma) and 10% normal goat serum (Thermo Scientific) in PBS. The blocking solution was removed, and the primary antibody was applied at concentrations shown in Supplementary Table S5 in 0.1% Triton X-100 and 1% normal goat serum in PBS. Sections were washed 3 times with PBS after overnight incubation at 4 °C and incubated with secondary antibodies (Supplementary Table S6) in PBS for 2 hours at room temperature. Sections were washed again and mounted in Vecta-shield (Vector Labs, Burlingame, CA) with Hoechst 33342 (1:1000, Thermo Scientific). Fluorescent images of the organoids’ sections were taken using Zeiss Axio ImagerZ2 equipped with an Apotome2 (Zeiss, Germany). Secondary antibody controls were carried out in the same manner but with the primary antibody step omitted. Representative examples are shown in Supplementary Fig. S21. Quantitative analyses of immunostained sections were carried out using ZEN (blue edition; ZEISS) and MATLAB (MathWorks ) software in at least 8 immunostained slices of retinal organoids of each biological replicate as described in.^32^

### Hematoxylin & Eosin Staining

OCT-12 μm slices of retinal organoids collected at day 150 of differentiation were stained with Hematoxylin and Eosin. Air-dried sections were incubated for 7 minutes at room temperature with Hematoxylin (LAMB/230-D), uniformly covering the tissue on the slide. Slides were washed in Milli-Q water, followed by 2-minute incubation at room temperature with Bluing Buffer (DAKO, CS702), repeated washes and final 1 minute incubation at RT with Eosin Mix (Eosin 1% AQUEOUS, LAMB/100-D) diluted 1:10 in tris-acetic buffer; 0.45 M, pH 6.0). The mix was then drained, slides washed with Milli-Q water, air-dried, and mounted with 85% glycerol (Merck Z0566194921). Bright-field images were taken using Zeiss Axio ImagerZ2 (Zeiss, Germany). The percentage of rosette-like structures was calculated by dividing the number of rosettes by number of cells in the section × 100.

### Cell-Cycle Phase Distribution Analysis

Organoids at day 90 of differentiation were dissociated to single cells with the Neurosphere Dissociation kit (P) (Miltenyi Biotech) according to the manufacturer’s protocol. Cell-cycle assay was performed with the BD Cycletest Plus DNA kit (BD Biosciences, 340242) following the protocol provided with the kit.

### TUNEL Assay

The OCT slices of retinal organoids collected at day 90 of differentiation were subjected to TUNEL assay (ApoBrdU-IHC DNA fragmentation assay kit, Bio Vision, K403-50) and examined with bright field microscope. TUNEL-positive stained cells were counted in at least 5 sections of H9 and iPSC-derived organoids.

### Lactate Dehydrogenase Cytotoxicity Assay

To assess cytotoxicity effect of 3 chosen chemotherapeutic agents, 150 days-old H9 RB1-null retinal organoids were incubated for 72 hours with melphalan (Cayman Chemical, 16665; final concentration in maintenance medium; 8, 16, and 32 μM), topotecan (Cayman Chemical, 14129; 5, 10, 15, and 150 μM) and TW-37 (Cayman Chemical, 20999; 0.1, 0.5, 1, and 10 μM) in 96-well plates. Sample’s medium was harvested and analyzed with CyQUANT LDH Cytotoxicity Assay (Invitrogen C20300) according to the manufacturer’s protocol.

### Western Blot Analysis

Cell or organoid lysates were prepared from pelleted hESCs/iPSCs or retinal organoids by the addition of cold RIPA Lysis Buffer (MILLIPORE, 20-188) with protease inhibitor mix (EDTA free, Roche), followed by 30-minute incubation on ice and pipetting in 10-minute intervals. Supernatants were retained after 5-minute centrifugation (4 °C) for SDS-PAGE. Between 10 to 20 µg of proteins from the cell or retinal organoids lysates were mixed with NuPAGE LDS Sample Buffer and NuPAGE Sample Reducing Agent (Thermofisher Scientific, NP0004), incubated at 70 °C for 10 minutes and separated on NuPage 4-12% Bis Tris Gel (Cell Signalling Technology, 125395) in Novex NuPAGE MOPS SDS Running Buffer alongside PageRuler Plus Prestained Protein Ladder (Thermo Scientific, 26619). The proteins were transferred to PVDF membranes by iBlot2 Life Technologies Apparatus. The following primary antibodies were incubated with the membranes overnight, at 4 °C: anti-pRB (abcam, ab1816160, dilution 1:1000), RBL1 (Cell Signalling Technology, 89798, 1:1000), RBL2 (Cell Signalling Technology, 13610, 1:1000), E2F1 (Cell Signalling Technology, 3742, 1:1000), Caspase-3 (abcam, 31A1067, 1:500), cyclin E1 (Cell Signalling Technology, 4129, 1:1000), and GAPDH (Santa Cruz Biotechnology, Sc-47724, 1:10 000). Subsequent incubation with secondary antibody was performed at room temperature for 1 hour, using peroxidase-conjugated anti-rabbit, or anti-mouse IgG (Dako). The signal was generated using the Thermo Fisher Scientific Super Signal West Pico Plus Chemiluminescent substrate kit and visualized by Amersham Imager 600.

### Transmission Electron Microscopy

Retinal organoids were fixed with 2% glutaraldehyde at 4 °C. Organoid processing and TEM was performed at Newcastle University Electron Microscopy Research Services. Ultrathin sections were stained with heavy metal salts (uranyl acetate and lead citrate) and imaged on a Hitachi HT7800 120 kV TEM using an EMSIS CMOS Xarosa high-resolution camera (Hitachi, Japan).

### Analysis Mitochondria in the Photoreceptor Inner Segments

To assess the number of mitochondria and mitochondrial cristae in photoreceptor inner segments (IS), TEM images of H9 or iPSC-derived organoids were analyzed with Microscopy Image Browser (MIB). Scale bars were recalibrated according to the image by specifying the length in micrometer. Segmentation was conducted by outlining organelles to generate raw data, mitochondrial counts, areas, and cristae number per IS. Mitochondria with areas below 1.0 × 10^-3^ μm^2^ were excluded from analyses.

### Single-Cell (sc) RNA-Seq Analysis

Retinal organoids (day 114) were dissociated to single cells using the Neurosphere Dissociation kit (P) (Miltenyi Biotech) according to the manufacturer’s protocol. Cell capture and library generation was carried out using the Chromium Single Cell 3ʹ Library & Gel Bead Kit, version 3.1 (10× Genomics). scRNA-Seq libraries were sequenced to 50 000 reads per cell on an Illumina NovaSeq 6000. The sequenced samples were de-multiplexed and aligned to human reference genome GRCh38 before being quantified using Quality Control CellRanger version 3.01. Cells with fewer than 1000 reads or 500 genes or >20% mitochondrial reads were filtered from the samples and not included in downstream analysis. DoubletFinder (version 2.0.3) was used to identify and remove doublets from the datasets. Data have been submitted to GEO (GSE173447).

### Combined Cluster Analysis

Two integrated datasets consisting of the Control and RB1-null retinal organoid samples and 3 patient organoid samples were created. The filtered datasets were downsampled so that there were equal numbers of cells in each sample to be combined (3000 cells for the Control and RB1- null organoids and 8000 cells for each of the patient samples). Seurat version 3.2.3 was used to predict cell cycle state of the cells. SCTransform (version 0.3.2) was used to normalize and scale the data. A nonregularized linear regression was used to subtract the effect of cell cycle, and other technical noise including percentage mitochondrial per cell and number of genes per cell. Principle component analysis was used to reduce the dimensions of the data. Harmony (version 1.0) was then applied to the joint datasets to remove batch effects. The combined datasets were then clustered using a resolution of 2.2 and markers were identified markers for each cluster using Seurat. The clusters were then annotated and assigned to cell types. Uniform manifold approximation and projection (UMAP) was used to visualize the clusters.

### Pseudotime Analysis

Monocle 2 (version 2.16) to study the trajectory of each sample. A CellDataset was created using counts from genes which were significantly differentially expressed (adjusted *P*-value < .05). Monocle was used to estimate size factors and dispersions then DDRTree was used to reduce the dimension of the data. The orderCells function was then used to learn a trajectory through the data.

### Soft Agar Colony Formation Assay

Retinal organoids at day 90 of differentiation were dissociated with Neurosphere Dissociation Kit (P) (Miltenyi Biotec, 130-095-943) according to the manufacturer’s protocol. Single-cell suspensions in organoids’ maintenance medium with 0.48% LB-agar (Invitrogen, 22700-025) were added to a 12-well plates precoated with 0.5% LB-agar in maintenance medium at concentration 1000/well. Cells were maintained in a 37 °C humidified incubator with 5% CO2, for 80 days.

### Statistical Analysis

An unpaired 2-tailed Student’s *t* test was used to compare the mean ± SEM values between pRB mutated and WT organoids or gene edited controls. The analyses were performed with GraphPad Prism software, values of *P* ≤ .05 were considered statistically significant (∗*P* ≤ .05, ∗∗*P* ≤ .01, ∗∗∗*P* ≤ .001, ∗∗∗∗*P* ≤ 0.0001).

## Results

### pRB Inactivation Results in a Significant Increase in Proliferating RXRγ^+^, Prox1^+^ and SNCG^+^ and a Decrease in AP2α^+^ Cells During Retinal Organoid Maturation

**Figure 1.**
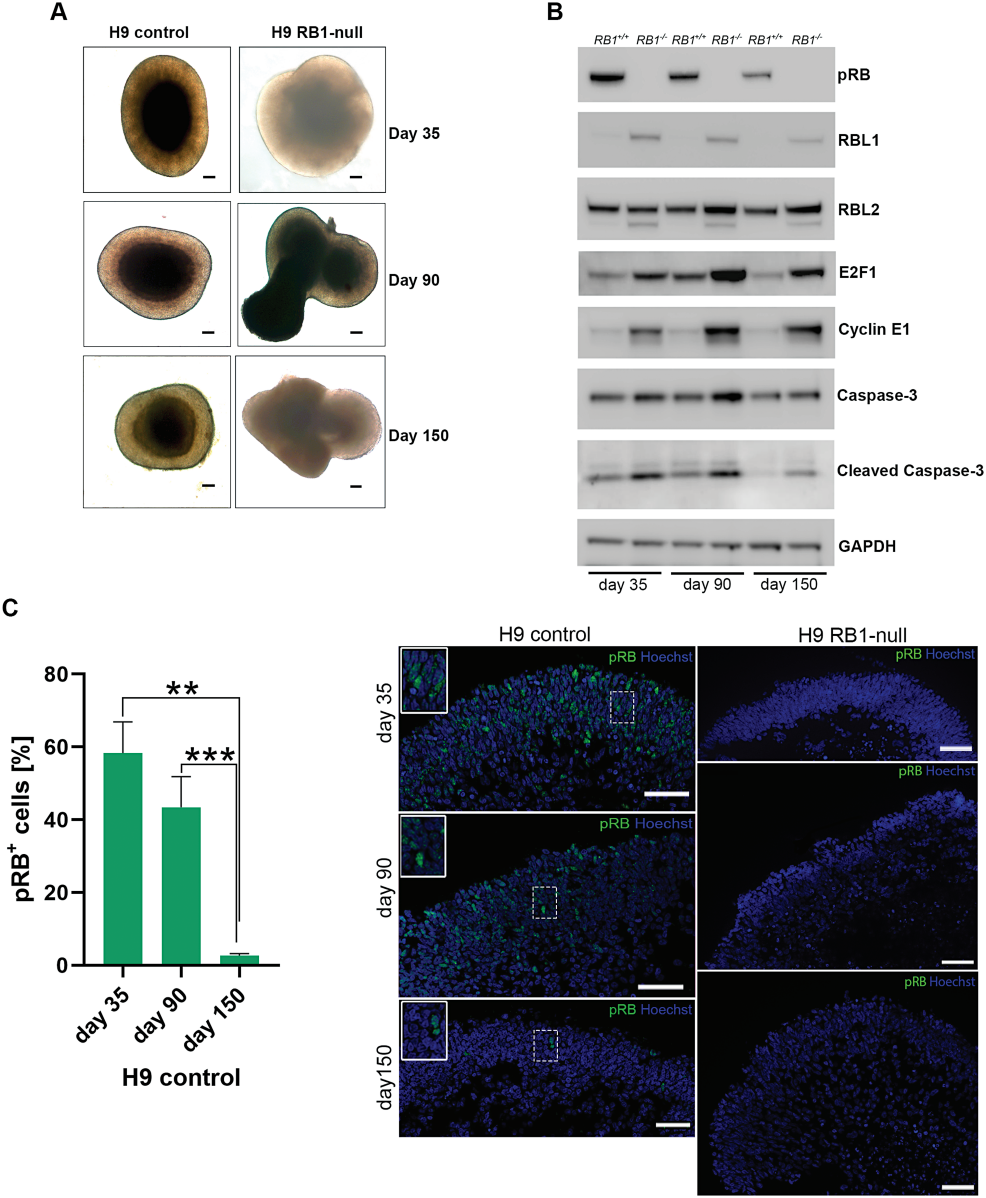
Characterization of H9 RB1-null-derived retinal organoids. (A) Representative light microscopy images of retinal organoids differentiated from H9 control and RB1-null at days 35, 90, and 150 (scale bar = 100 µm). (**B)** Representative Western-blot analysis of pRB and related proteins steady state levels in control (*RB1*^*+/+*^) and H9 RB1-null (*RB1*^*−/−*^) hESC organoids at days 35, 90, and 150 of differentiation; GAPDH was used as loading control. (**C**) Representative immunofluorescence images of anti-pRB staining at days 35, 90, and 150 in control and RB1-null hESC organoids alongside quantification of pRB-positive cells in H9 control. Insets show higher magnification images. Data are presented as mean ± SEM (*n* = 10 sections from each biological replicate). Scale bars = 50 µm.

Human ESC lines with homozygous inactivation of *RB1* (*RB1*^*−/−*^, named RB1-null for the rest of this manuscript)^26^ were differentiated to retinal organoids using our established 96-well plate protocol.^34^ Both the control and RB1-null hESC line generated retinal organoids under 3D culture conditions (Fig. 1A). Analysis of protein expression indicated an increase in the expression of p107 (RBL1) in RB1-null retinal organoids at all 3 time points alongside increased p130 (RBL2) expression at days 90 and 150 albeit to lesser extent (Fig. 1B). The expression of RBL1 as well as cyclin E1 and E2F1 is transcriptionally regulated by E2F transcription factors. Our data show increased expression of Cyclin E1 and E2F1 in RB1-null organoids at days 35, 90, and 150. The increase is also noticeable for the Caspase-3 and its cleaved isoform, corroborating published results in RB1-null organoids^40^ and mouse Rb1-deficient retinas.^45^

In wild-type organoids, the steady-state level of pRB is highest at the early stage of differentiation (day 35) and decreases with time (Fig. 1B). This is also mirrored by immunofluorescence analysis where around 40% of cells express pRB at day 90, in contrast to day 150 where only 3% of pRB-positive cells are found in control organoids (Fig. 1C). During early differentiation, pRB expression was observed in retinal progenitors (VSX2^+^) and post-mitotic photoreceptor precursors (CRX^+^), retinal ganglion (SNCG^+^), and proliferating cells (Ki67^+^) (Supplementary Fig. S1A). Although the percentage of pRB expressing cells declined over time, co-expression with retinal progenitor and bipolar cell (VSX2), rods and cones (RCVRN), cones (RXRγ, ARR3, OPN1LW/MW), rods (RHO), horizontal cell (PROX1), bipolar cell (PKCα), and Müller glia cell (VIMENTIN) markers were still observed at days 90 and 150 of differentiation (Supplementary Figs S1B and S2). In addition, pRB expression was co-localized with proliferation marker Ki67; however, no co-localization of pRB with apoptotic marker (CASP3) was detected. No pRB expression was detected during differentiation of RB1-null organoids (Fig. 1C).

**Figure 2.**
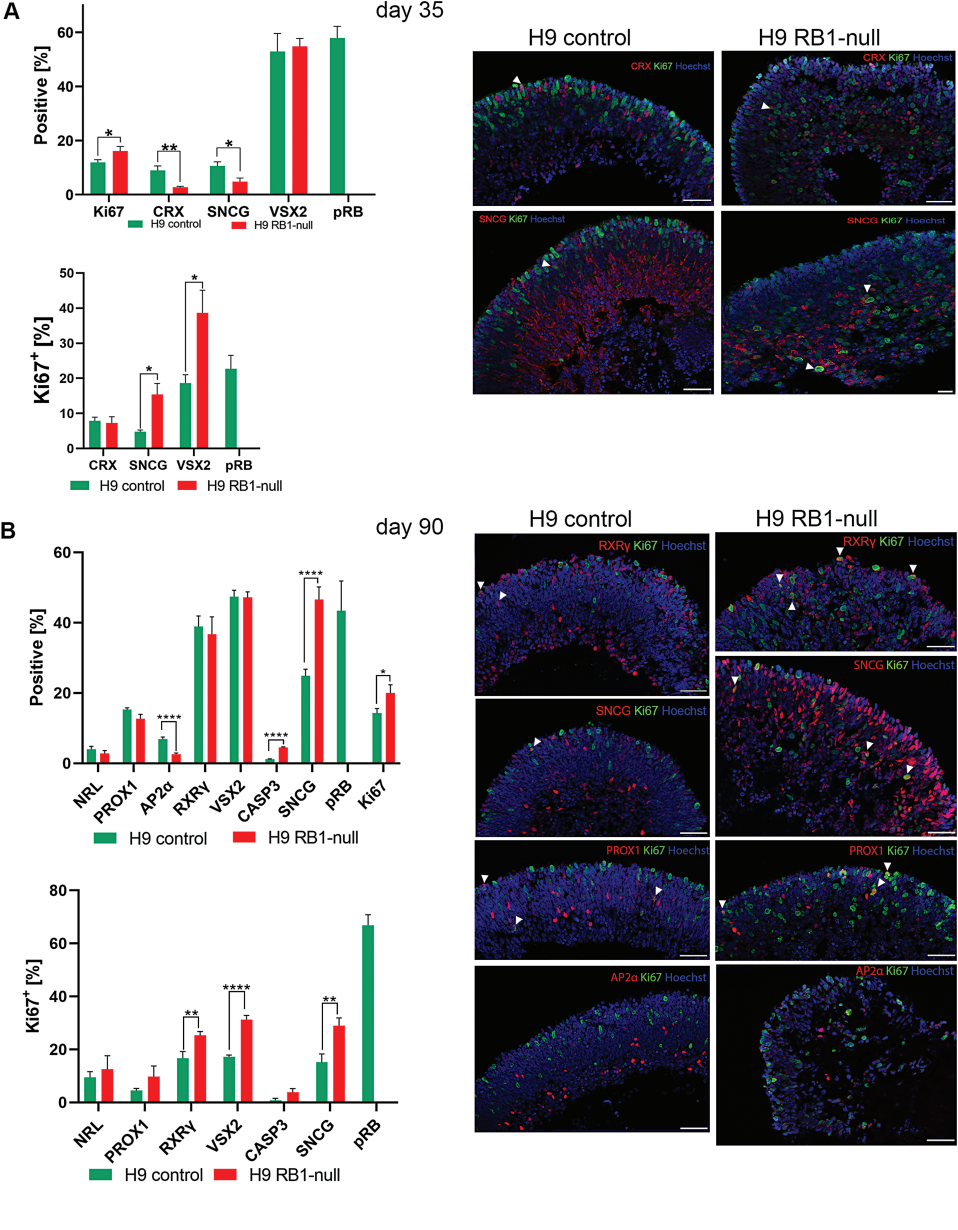
Immunohistochemical analysis of RB1-null hESC retinal organoids at days 35 and 90 of differentiation. (**A**) Bar charts showing the retinal cell types and Ki67^+^ co-expressing fractions (left) and representative immunofluorescence analysis for CRX, SNCG, and Ki67, counterstained with Hoechst (right) at day 35. (**B**) Bar charts showing the percentage of Caspase-3^+^ cells (CASP3), retinal cell types and Ki67^+^ co-expressing fractions (left) and representative immunofluorescence analysis for RXRγ, SNCG, PROX1, AP2α, and Ki67, counterstained with Hoechst (right) at day 90. Data are presented as mean ± SEM (*n* = 10 sections from each biological replicate). White arrowheads point at co-localization of Ki67 with the specified retinal marker. Scale bars = 50 µm.

**Figure 3.**
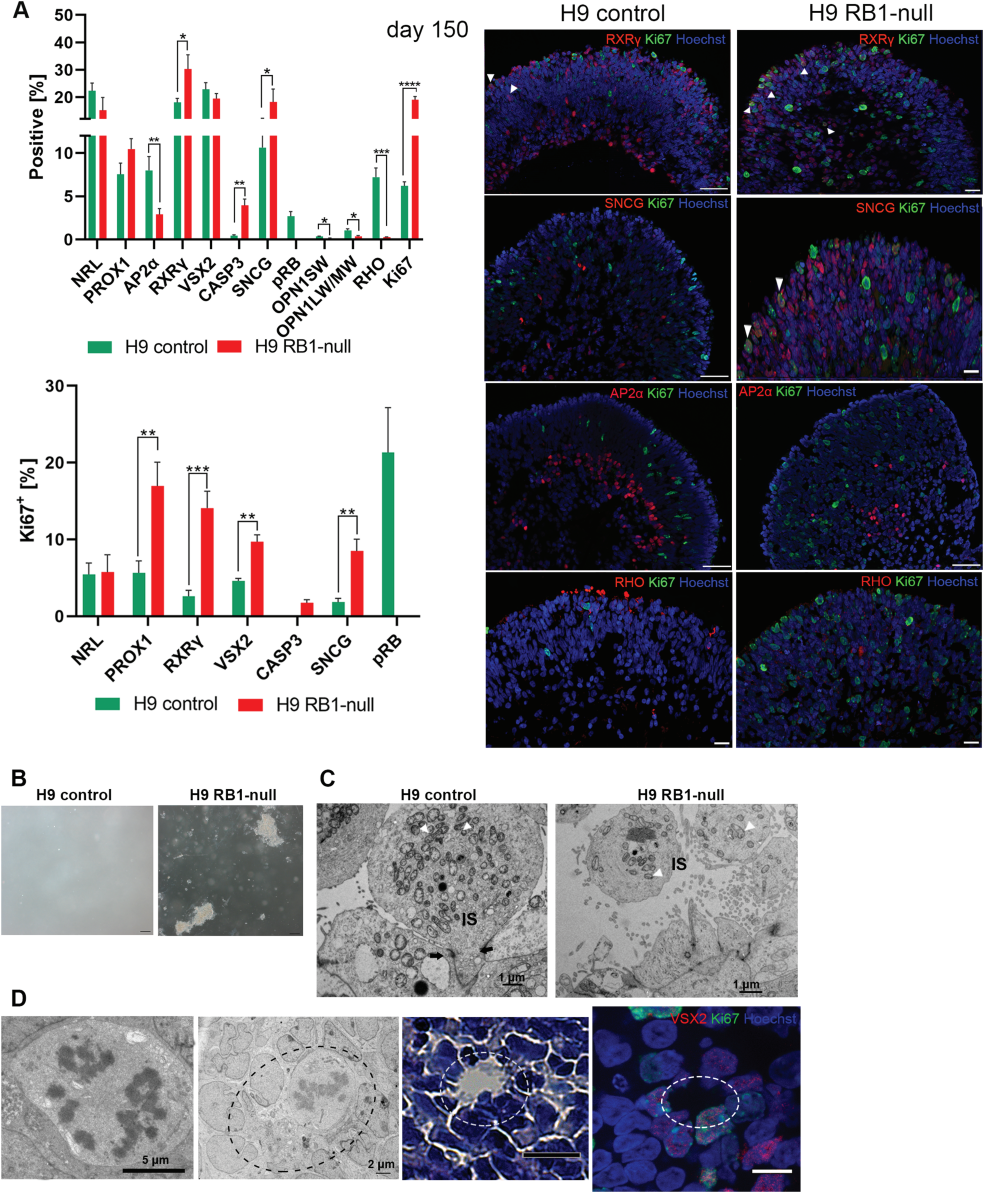
Tumorigenic characteristics of RB1-null hESC organoids. (**A**) Characterization of RB1-null-derived retinal organoids at day 150 of differentiation. Bar charts showing the percentage of Caspase-3^+^ cells (CASP3), the retinal cell types and Ki67^+^ co-expressing fractions (left) and representative immunofluorescence analysis for RXRγ, SNCG, AP2α, RHO, and Ki67, counterstained with Hoechst (right) at day 150 of differentiation. Data are presented as mean ± SEM (*n* = 10 sections from each biological replicate). White arrowheads point at co-localization of Ki67 with the specified retinal marker. Scale bar= 50 µm. (**B**) Soft agar colony formation assay showing ability of pRB-depleted retinal cells originating from day 90 RB1-null hESC retinal organoids to grow in suspension (bright field images, scale bar = 100 μm). (**C**) Transmission electron microscopy of photoreceptor inner segments (IS), visible mitochondria (white arrowheads) and outer limiting-like membrane (black arrows) (scale bar = 1μm). (**D**) Representative TEM example of mitotic cell in RB1-null organoid at day 200 of differentiation (left panel) followed by rosette-like structure (marked with dashed ellipse) shown in TEM images (middle panel) and H&E-stained sections in the right-hand side panel, followed by VSX2 and Ki67 immunofluorescence analysis; rosette-like structure marked with dashed ellipse (scale bars = 5, 2, 50, 5 µm, respectively).

Given the reported role of pRB on cell proliferation, apoptosis, and retinal development, we assessed the impact of pRB inactivation in different stages of retinal organoid development. At all timepoints assessed (days 35, 90 and 150), there was a significant increase in the percentage of Ki67^+^ cells in RB1-null compared with control organoids (Figs. 2A, B and 3A, Supplementary Figs. S3A, B and S4). The cell-cycle phase distribution analyses based on propidium iodide (PI) staining confirmed enriched fraction of cells in S and G2/M phase in day 90 RB1-null organoids (9.7%) compared with wild type (3.2%). A common feature of RB1-null organoids was the increased presence of proliferating retinal progenitors (VSX2^+^Ki67^+^) from day 35, and the increased fraction of proliferating cone precursors (RXRƴ^+^ Ki67^+^) from day 90 (Figs. 2A, B and 3A). The putative retinal ganglion cells (RGCs) immunostained with SNCG were also more frequent in the RB1-null organoids. In the control organoids, these putative RGCs were mostly located at the basal aspect; however, in the RB1-null organoids, these were found throughout retinal organoids and concentrated in the apical layer where normally photoreceptor precursors reside (Figs. 2A, B and 3A, Supplementary Fig. S5B). Moreover, RB1-null organoids were characterized by a significant increase in the fraction of proliferating SNCG^+^ (all time points, Figs. 2A, B and 3A) and PROX1^+^ (day 150, Fig. 3A) cells as well as a significant decrease in AP2α^+^ amacrine cells from day 90 of differentiation (Fig. 2B), with the latter corroborating data published by.^40^ The expression of mature cone and rod photoreceptor markers (OPN1SW^+^, OPN1LW/MW^+^ and Rhodopsin^+^) was significantly reduced in day 150 RB1-null organoids (Fig. 3A, Supplementary Fig. S6A). In parallel, we detected a low but significant increase in the percentage of apoptotic cells (Figs. 2B and 3A, Supplementary Figs. S3B and S4) in the days 90 and 150 RB1-null organoids, confirming the increase of the cleaved Caspase-3 steady state levels (Fig. 1B). The TUNEL assay corroborated the higher number of apoptotic cells in pRB-depleted organoids sections (Fig. S3C).

We performed soft colony agar assays to assess for tumorigenic potential of RB1-null organoids. This is an anchorage independent growth assay in soft agar widely used for detecting malignant transformation of cells. To this end, single-cell suspensions from organoids at day 90 of differentiation were cultured in soft agar medium. This revealed the presence of small and large sphere-like aggregates in RB1-null organoids only (Fig. 3B), indicating cell growth in an anchorage-independent manner, which is suggestive of cell transformation in vitro. Transmission electron microscopy analysis revealed the presence of tightly packed photoreceptors in the apical layer of the control and RB1-null organoids with inner segments displaying mitochondria of different morphologies (Fig. 3C). It is worth noting that RB1-null photoreceptors displayed less mitochondria in their inner segments and for those that were present, partial or complete cristolysis was the most common feature (Fig. S6B), corroborating data obtained in poorly differentiated Rb tumors.^46^ Furthermore, we observed condensed mitotic chromosomes (Fig. 3D) at day 200 of differentiation in RB1-null organoids and patterns of cells organized in the form of rosette-like structures, resembling pathologic features of Rb classified as Flexner-Wintersteiner rosettes and Homer Wright rosettes.^47^ Cells forming the rosette-like structures expressed both VSX2 and Ki67 markers and were found at higher frequency (0.43%) in pRB-depleted organoids compared to controls (0.12%). Together, the cellular differentiation, soft agar assays, and ultrastructural microscopy observations support the tumorigenic phenotype of RB1-null organoids in vitro.

In summary, our data show that inactivation of pRB results in a significant and consistent decrease in AP2α^+^ amacrine cells in retinal organoids as they develop and mature up to day 150. Importantly, pRB inactivation also results in a significant increase in the percentage of RXRγ^+^Ki67^+^, PROX1^+^Ki67^+^, VSX2^+^ Ki67^+^, and SNCG^+^Ki67^+^ cells as well as acquisition of tumorigenic features uncovered by the soft agar growth assays and transmission electron microscopy analyses.

### Single-cell RNA-Seq Analysis Identifies Stressed Proliferating Cones as the Likely Cell of Origin for Rb in RB1-Null Organoids

**Figure 4.**
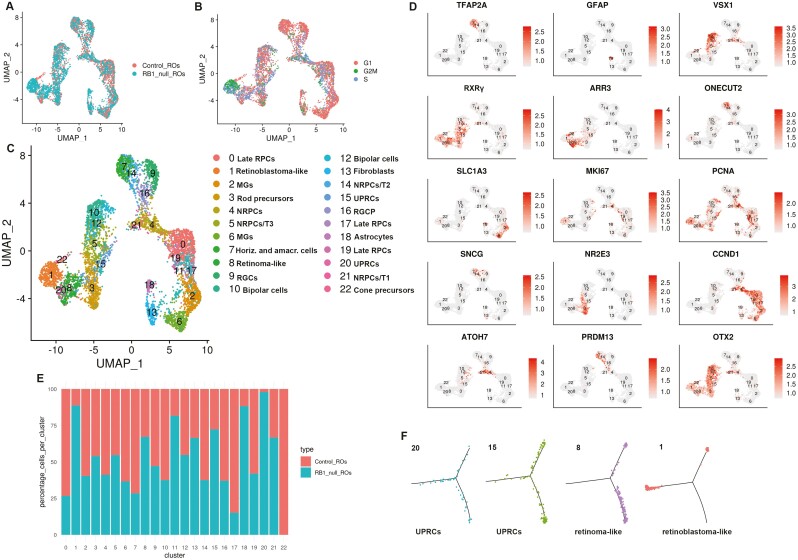
Single-cell RNA-Seq reveals the presence of retinoma and Rb cells in H9 RB1-null organoids. (**A, B**) UMAPs showing the cell clusters in control and RB1-null hESC organoids (A) and cell cycle distribution (B). (**C**) UMAP of integrated control and H9 RB1-null hESC organoids with cell cluster annotations. (**D**) Expression of key retinal cell type markers genes used to facilitate cluster definition in C overlaid onto the UMAPs. (**E**) Percentages of cells in each cell cluster of control and RB1-null hESC organoids. (**F**) Monocle-2 pseudotime showing progression from UPRCs to retinoma and Rb cell clusters. Cluster 11 was exempted from analysis because of low read number. RPCs: retinal progenitor cells, MGs: Müller glia cells, NRPCs: neurogenic RPCs, RGCs: retinal ganglion cells, UPRCs: cones with high expression of genes involved in unfolded protein response, RGCP: RGC precursor, T1, T2, T3: transient neurogenic progenitor populations.

To get detailed insights into their cellular composition, single-cell RNA-Seq of both control and RB1-null organoids was performed. After quality control and filtering, 12 545 and 9361 cells were obtained from hESC control and RB1-null organoids, respectively. Following cell cycle regression, these were merged using the Seurat package to allow analysis of a higher cell number. The findCluster function (*k* = 1.8) revealed 22 distinct clusters (Fig. 4A-C). The findMarkers function found the marker genes for each cluster (Supplementary Table S1). The differentially expressed genes were used to identify each cluster (Fig. 4C, D). As expected, all retinal cell types including late retinal progenitor cells (RPCs characterized by high expression of *SPP1*, *CLU*, *CCND1*, *SPP1*, etc.), neurogenic RPCs (NRPCs characterized by high expression of *HES6*, *GADD45A*, *GADD45G*), RGCs (characterized by high expression of *GAP43*, *SNCG*), cone and rod precursors (characterized by high expression of *RXRG* and *NR2E3* respectively), Mϋller glia cells (characterized by high expression of *CYP26A1*, *SLC1A3*), and interneurons (amacrine, horizontal, and bipolar cells characterized by high expression of *TFAP2A*, *ONECUT2*, and *VSX1*, respectively) were identified (Fig. 4C, D). Recent single-cell sequencing studies of human fetal retina and pluripotent stem cell-derived retinal organoids^48^ have indicated the existence of several transient neurogenic progenitor populations, with capacity to give rise to RGCs (T1), amacrine and horizontal cells (T2) and photoreceptor and bipolar cells (T3). All these 3 transient neurogenic populations were also identified in control and RB1-null organoids. Importantly, we found 8 clusters, which were enriched in the RB1-null organoids (Fig. 4E). Among those, 4 clusters expressing cone and proliferation markers were selected for further analysis as follows: (1) *proliferating cone precursors* (cluster 1) composed of cells in S and G2/M phase of the cell cycle (Fig. 4B) and characterized by high expression of cone precursors (*DCT*, *PDE6H*, *AIPL1*, *TULP1*, *RXRγ*), proliferation markers (*HIST1H4C*, *TOP2A, MKi67*, *PCNA*, *MCM3*, *CDC25A*, *CDC25C*, *CHEK1*, *E2F3*, *PRC1*, *SKP2*, *UBE2C*), and *DEK*, *KIF14*, *MYCN*, *SYK* (characteristic for Rb) which was defined as Rb-like cluster^21,22,42,49,50^ (Fig. S7A); (2) *cluster 8*, characterized by high expression of cone precursor markers (*PDE6H*, *GNB3*, *RXRγ*, *ARR3*, *GNGT2*), retinoma marker (*CDCA7*, *HELLS*), and *PCNA* proliferation marker, but greatly reduced expression of *MKi67*, which was defined as retinoma-like cluster^9,42^ (Fig. 4D, Supplementary Fig. S7B); (3) proliferating *cone precursor clusters 20 and 15* characterized by high expression of cone markers (*RXRγ*, *ARR3*), proliferation marker (*PCNA*) as well as genes involved in unfolded protein response (UPR) (*SCG3*, *AKAP9*, *VXN*) also identified recently by Liu et al,^42^ which for ease of presentation are abbreviated to UPRCs (Supplementary Table S1).

Pseudotime analysis with Monocle 2 was performed to identify Rb’s cell of origin within RB1-null organoids. This analysis positioned proliferating cones with expression of UPR markers (clusters 20 and 15) (Fig. 4F) at the beginning of the pseudotime. The right-hand side of the pseudotime is occupied by the retinoma-like cluster 8 and the left-hand side by Rb-like cluster 1. Together these analyses point to proliferating cone precursors with high expression of UPR-related genes as the most likely cell of origin for Rb within hESC RB1-null organoids. Some of these UPRCs likely give rise to nonmalignant retinoma tissue, while some others acquire high expression of *MKi67* and transform into Rb cells. Together these data suggest that tumor development from proliferating cone precursors to premalignant retinoma and development of malignant Rb tumors is replicated within the hESC RB1-null organoids.

### Rb Proliferating Cones Express Markers of RGCs and Horizontal Cells in Human ESC-derived RB1-Null Organoids

We compared the immunofluorescence analysis with single-cell RNA-seq data and noticed that the increased fraction of SNCG^+^Ki67^+^ and PROX1^+^Ki67^+^ shown by immunofluorescence analysis at day 90 and/or day 150 was not corroborated by single-cell RNA-Seq data, which in fact point to a reduction in the amacrine and horizontal cell cluster 7 and RGC progenitor cluster 16. Further we were puzzled by the co-expression of Ki67 with RGC and horizontal cell markers as one would expect these to be postmitotic neurones. Hence, we speculated that other cells apart from horizontal and RGCs may express horizontal cell and RGC markers. A careful assessment of single-cell RNA-Seq data focusing on the expression of another RGC marker, *GAP43*, indicated its co-expression with the cone marker *RXRγ* in the Rb cluster 1 characterized also by high expression of *Ki67* (Fig. S8A). We were able to validate these results by immunofluorescence analysis, which clearly demonstrated the co-expression of RXRγ with SNCG in the apical layer of the RB1-null organoids where normally photoreceptors reside (Fig. S8B). Equally, co-expression of the horizontal cell marker *ONECUT2* (Fig. S8C) with *RXRγ* was also observed in some cells of Rb cluster 1 and confirmed by immunofluorescence analysis, depicting the co-expression of RXRγ with PROX1 in the apical layer of RB1-null organoids (Fig. S8D). Recently, our group has published single-cell RNA-Seq of human Rb tumors,^49^ which were further assessed for marker co-expression indicated above. This analysis indicated co-expression of *RXRγ* with *Ki67* and *GAP43* or *ONECUT1* in G2/M cone precursors clusters 8 and 12 (Supplementary Fig. S9), which constitute the Rb cell of origin. Together these data indicate expression of other retinal cell type markers in the Rb proliferating cone clusters in both Rb tumors and pRB-depleted hESC organoids, a finding which is corroborated by a very recent publication showing expression of less differentiated cones together with neuronal/ganglion-cell markers in 102 Rb tumor samples.^51^

### A Reduced Presence of Amacrine Cells in Human ESC-Derived RB1-Null Organoids

Apart from increased presence of proliferating cones, single-cell RNA-Seq analysis also revealed a noticeable decrease in amacrine and horizontal cells (cluster 7) in the RB1-null organoids, which corresponds with the decrease in AP2α^+^ cells shown by the quantitative immunofluorescence (Figs. 2B and 3A). Immunofluorescence analysis indicated co-staining of AP2α with Caspase-3 in the RB1-null organoids (Supplementary Fig. S10) in very few amacrine cells, suggesting that while apoptosis may play a minor role in the loss of amacrine cells at this stage of differentiation, another mechanism may be in place to account for their significant decrease. To this end, it is worth noting the increase in NRPC/T1 cluster 21, which is thought to give rise to RGC progenitors (cluster 16), RGCs (cluster 9), and NPRC/T2 (cluster 14) in the RB1-null organoids (Fig. 4E). The NRPC/T1 is present in the RB1 control organoids, but at a smaller percentage, leading us to suggest that a delay or a block in differentiation of these progenitor cells may be the main underlying cause for the amacrine, horizontal and retinal ganglion-cell reduction revealed by the single-cell RNA-Seq of RB1-null organoids. This hypothesis is supported by the reduction of NRPCs/T2 cluster 14 and RGC progenitor cluster 16, which give rise to amacrine and horizontal cells and RGCs respectively (Fig. 4E). Our interpretation of these data is that RB1 depletion may cause a block in differentiation downstream of NRPC/T1 in the H9 RB1-null organoids, leading to a reduction in RGC, horizontal and amacrine cells and their progenitors. This hypothesis is further corroborated by the lack of co-localization between CASP3 and PROX1 or SNCG markers in immunofluorescence assays (Supplementary Fig. S10) in the RB1-null organoids, which indicates that apoptosis is unlikely to be the main mechanism for the reduction observed in these cell types in hESC RB1-null organoids. Nonetheless, these findings need to be further confirmed by detailed lineage tracing studies as well as apoptosis-based assays throughout differentiation timeline, to exclude the cell death at earlier differentiation time points as the cause for the reduced RGC, horizontal and amacrine cell presence in the hESC RB1-null organoids.

### Mitotic Activity and Tumorigenic Phenotype Observed in Homozygous But Not Heterozygous Patient-Specific Rb1-Ipsc Line Organoids

**Figure 5.**
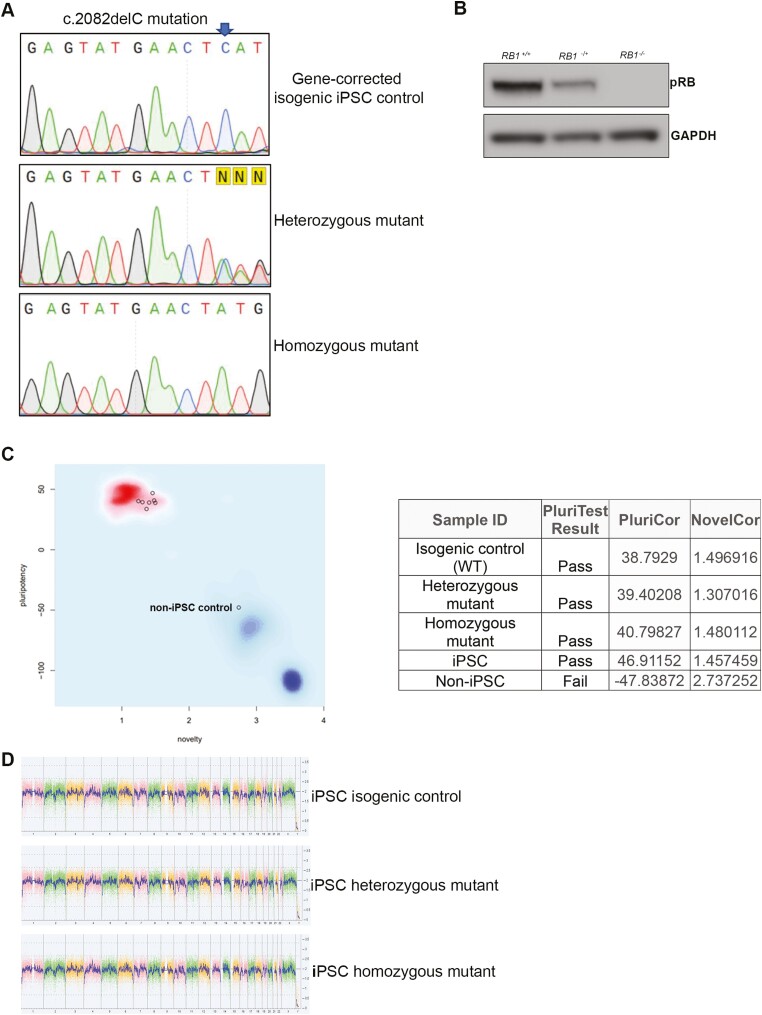
Characterization of *RB1* patient-iPSC-derived organoids. **(A**) Sequencing confirms the correction of c.2082delC mutation in the CRISPR-Cas9 corrected heterozygous *RB1* patient iPSC line and the introduction of the mutation in the second allele (homozygous mutant). Highlighted in yellow, the letter N, indicates heterozygosity of the original iPSC line caused by the single C deletion. C is marked by an arrow in wild-type sequence. (**B**) Western-blot analysis of pRB steady state level in iPSC lines (isogenic control, heterozygous, and homozygous mutant for c.2082delC), GAPDH was used as loading control. (**C**) Pluripotency test. The pluripotency plot window provides a visual representation of the tested samples in the analysis. The pluripotency and novelty *x*/*y* scatter plot combine the pluripotency score on the *y*-axis with the novelty score on the *x*-axis. The red and blue background hint to the empirical distribution of the pluripotent (red) and non-pluripotent (blue) samples in the reference data set. Samples were analyzed using an algorithm that integrates gene expression data to authenticate pluripotency status. Samples were screened against samples in the stem cell database and given a pluripotency score (PluriCor) and novelty score (NovelCor), which are shown in the table. Pass shows a clear pluripotency signature. Fail means the samples are not pluripotent. A non-iPSC sample was used in this experiment to serve as a negative control for non-pluripotency. (**D**) The whole-genome view displays all somatic and sex chromosomes in one frame with high level copy number. The smooth signal plot (right *y*-axis) is the smoothing of the log2 ratios which depict the signal intensities of probes on the microarray. A value of 2 represents a normal copy number state (CN = 2), value of 3 represents chromosomal gain (CN = 3), value of 1 represents a chromosomal loss (CN = 1). The pink, green and yellow colors indicate the raw signal for each individual chromosome probe, while the blue signal represents the normalized probe signal which is used to identify copy number and aberrations (if any).

To obtain a 3D in vitro model of Rb that resembles patient-specific genomic background in combination with a hereditary mutation in the *RB1* gene, we generated iPSC lines from a patient bearing the heterozygous mutation: c.2082delC. The child had developed malignant bilateral growths before the age of 4. As detailed in materials and methods and presented in Supplementary Fig. S11A, PBMCs were reprogrammed using non-integrating Sendai viruses containing the reprogramming factors OCT4, SOX2, KLF4, and MYC. More than 10 independent iPSC clones were established for the mutation, expanded, screened for the patient-specific *RB1* mutation confirming their heterozygosity (Supplementary Fig. S11B) and assessed for the expression of pRB (Supplementary Fig. S11C). To create the homozygous mutant patient-specific iPSC line as well as fully corrected isogeneic control from the heterozygous c.2082delC *RB1*-patient-specific iPSC line, gRNAs in complex with Cas9 enzyme were delivered to the cells along with single-stranded oligonucleotides (ssODN) allowing homology-directed repair mechanism (Fig. 5A, B, Supplementary Fig. S12). Off-target sequences identified for both gRNAs (Supplementary Table S2) were checked for possible mutational changes in isogenic control and homozygous mutant selected clones. Negative outcomes of this PCR-based screening indicated no off-target effects. Pluritest and KaryoStat assays confirmed the pluripotent nature and the lack of genomic instabilities in the homozygous mutant and isogenic control clones (Fig. 5C, D). As expected, no expression of pRB was observed in the homozygous mutant clone, while reduced expression was observed in the heterozygous clone (Fig. 5B).

**Figure 6.**
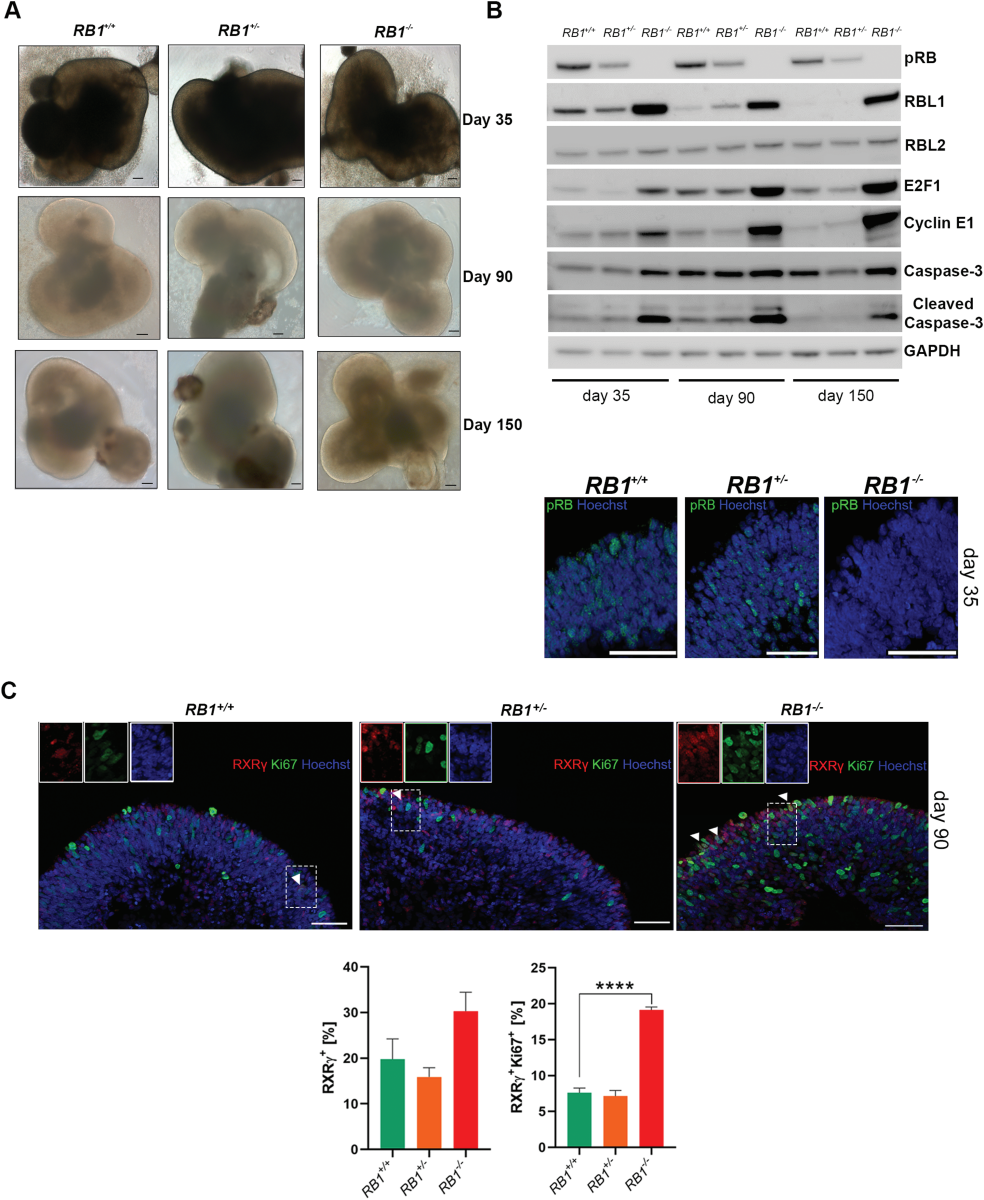
*RB1* patient-specific derived retinal organoids. (**A**) Representative light microscopy images of retinal organoids differentiated from iPSC isogenic control, heterozygous and homozygous mutant for c.2082delC at days 35, 90, and 150, scale bar = 100 μm. (**B**) Western-blot analysis of pRB and related proteins steady state levels in control (*RB1*^*+/+*^), heterozygous (*RB1*^*+/-*^) and homozygous (*RB1*^*−/−*^) organoids at days 35, 90, and 150 of differentiation, GAPDH was used as loading control. Below, example immunostaining for pRB, counterstained with Hoechst at day 35 of differentiation, scale bar = 50 µm. (**C**) Representative images of immunostaining for RXRγ and Ki67, counterstained with Hoechst at day 90 of differentiation alongside graphs depicting the percentage of RXRγ expressing cells and Ki67^+^ co-expression fractions. Data are presented as mean ± SEM (*n* = 9 sections from each biological replicate), scale bar = 50 µm.

The wild-type, heterozygous, and homozygous c.2082delC *RB1* iPSC lines were subjected to the same retinal differentiation protocol as control and RB1-null hESC lines. All 3 cell lines generated retinal organoids under 3D culture conditions (Fig. 6A). pRB expression was absent in homozygous patient organoids, while cell-cycle related proteins indicated a significant increase in the expression of RBL1, E2F1, cyclin E1 and cleaved Caspase-3 in RB1^*−/−*^ retinal organoids (Fig. 6B), corroborating our results in RB1-null hESC organoids.

Similarly to human ESC-derived RB1-null organoids, a significant increase in the percentage of SNCG^+^, VSX2^+^, and Ki67^+^ cells was noted in the homozygous *RB1* organoids throughout the differentiation time course (Supplementary Figs. S13A, B, S14A, B, S15A, B). The cell-cycle phase distribution analyses based on propidium iodide (PI) staining confirmed enriched fraction of cells in S and G2/M phase in *RB1*^*−/−*^ organoids (11.1%) compared to isogenic control (4.6%). Although the percentage of VSX2^+^ cells was increased in both heterozygous- and homozygous-derived retinal organoids at days 35 and 90 of differentiation, only homozygous organoids were characterized by an increased percentage of proliferating VSX2^+^ marked by Ki67 staining (Supplementary Figs. S13B and S14B). Importantly, the quantitative immunofluorescence analysis confirmed a significant increase in the percentage of proliferating cones in the homozygous mutant retinal organoids at days 90 and 150 of differentiation (Fig. 6C; Supplementary Fig. S15A, B), corroborating data obtained from RB1-null hESC organoids. Notably, there was also an enrichment of proliferating SNCG^+^ (throughout the differentiation time course) and PROX1^+^ cells (from day 90) cells but only in the *RB1*^*−/−*^retinal organoids (Supplementary Figs. S13B, S14B, S15B). The rod precursor cells, immunostained with the NRL antibody were unaffected by heterozygous, or homozygous mutation in *RB1* gene, but importantly the AP2α^+^ cells were undetectable in *RB1*^*−/−*^ organoids either at day 90 or day 150 of differentiation (Supplementary Figs. S14 and S15). Apoptotic cells (shown by CASP3 staining), although detected at very low level, were significantly increased in days 90 and 150 *RB1*^*−/−*^ immunostained sections (Supplementary Figs. S14A, B, S15A and B). These results were further verified by the TUNEL assay (Supplementary Fig. S14C). Soft colony agar assays confirmed *RB1*^*−/−*^ cell transformation in vitro (Supplementary Fig. S15C). TEM sections analyses revealed a trend for a decrease in the mitochondrial cristae number (Supplementary Fig. S15D and E). We also observed mitotic chromosomes in day 200 *RB1*^*−/−*^ organoids (Supplementary Fig. S15F) and patterns of cells organized in the form of rosette-like structures. Cells forming the rosette-like structures expressed both VSX2 and Ki67 markers and were found at higher frequency (0.72%) in homozygous compared with heterozygous (0.34%) and isogenic control organoids (0%).

In summary, homozygous Rb patient-derived organoids present a high level of proliferating retinal progenitors and cone precursors, increased fraction of SNCG^+^Ki67^+^, PROX1^+^Ki67^+^ cells and a complete lack of AP2α^+^ amacrine cells. It is interesting to note that an increased fraction of VSX2^+^ cells was observed in the heterozygous mutant clones at day 35 and 90 of differentiation; however, the higher mitotic and tumorigenic activity was restricted to the homozygous mutant clone only, indicating that inactivation of both copies of *RB1* is needed to initiate tumorigenesis.

### Presence of Rb-Like Cluster with Shared Expression of Cone, RGC and Horizontal Cell Markers and Increased Progenitor Cells in Homozygous Patient Organoids

**Figure 7.**
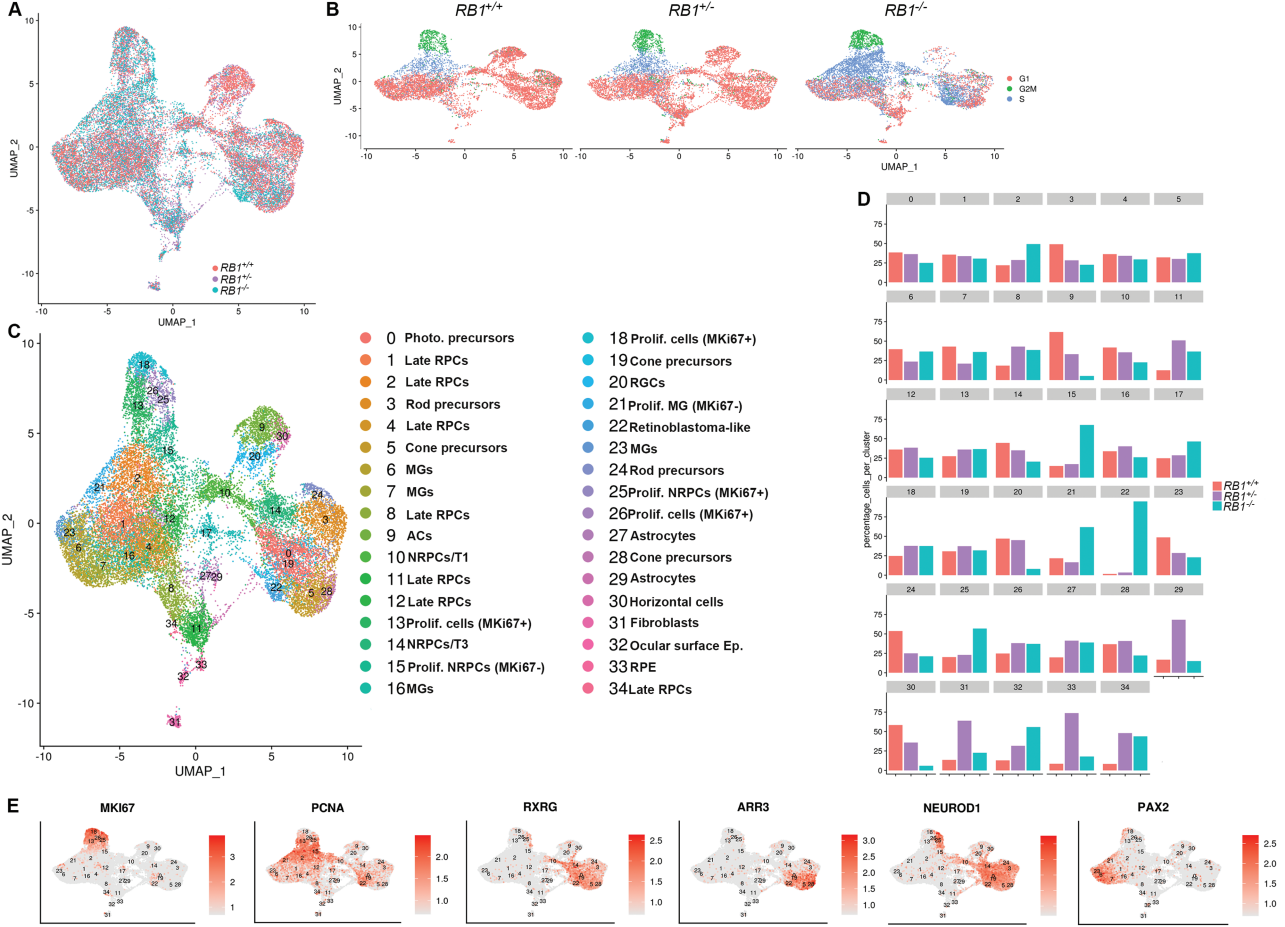
Single-cell RNA-Seq of *RB1* patient-specific iPSC-derived retinal organoids. UMAPs showing the cell clusters in control (*RB1*^*+/+*^), heterozygous (*RB1*^*+/-*^) and homozygous (*RB1*^*−/−*^) organoids (**A**) and cell cycle distribution (**B**). (**C**) UMAP of integrated control (*RB1*^*+/+*^), heterozygous (*RB1*^*+/-*^) and homozygous (*RB1*^*−/−*^) organoids with cell cluster annotations. Cluster 17 was exempted from analysis because of low read number. (**D**) Percentages of cells in each cell cluster of control *RB1*^*+/+*^, *RB1*^*+/-*^ and *RB1*^*−/−*^ organoids. (**E**) UMAPS with overlays of representative marker gene expression.

The wild type, heterozygous, and homozygous c.2082delC *RB1* iPSC lines were processed by single-cell RNA-Seq using the same pipeline as described for the control and RB1-null hESC lines above. Following quality control, 8375, 8577, and 12848 cells were obtained from *RB1* heterozygous, homozygous, and the wild-type organoids, respectively. These were downsampled to 8000 cells. Following cell cycle regression and integration using Harmony, 34 cell clusters were identified and defined based on the expression of highly and differentially expressed marker genes (Fig. 7A-C, Supplementary Table S3). Cell cycle phase overlays onto the UMAP indicated a great similarity between the heterozygous and wild-type organoids as expected. On the contrary, a significant increase in percentage of cells in S-phase of the cell cycle was clearly observed in the patient homozygous *RB1* retinal organoids (Fig. 7B). To gain clearer insights into the identity of these proliferating cells, a cell percentage by cluster analysis was performed, showing a significant increase in 5 retinal clusters, out of which only one (cluster 22;) was associated with Rb-like features including high expression of cone precursor markers (*RXRγ*, *ARR3*), proliferation markers (*MKi67*, *PCNA*, *CCNE2*, *NUSAP1*), DNA replication licencing factors (*MCM3*, *MCM4*), *DEK*, *SYK* (characteristic for Rb) and was almost exclusively present in the patient homozygous *RB1*^*−/−*^ retinal organoids (Fig. 7D, Supplementary Fig. S16). The other 4 enriched clusters (2, 15, 21, 25) were characterized by high expression of proliferation marker *PCNA*, however only cluster 25 showed high expression of *MKi67* (Fig. 7E). Two of these clusters (25 and 15) shared markers of neurogenic RPCs (eg, *HES6*, *GADD45A*, *NEUROD1*), one cluster shared expression of late RPC markers (eg, *SPP1*, cluster 2), while cluster 21 was associated with Müller Glia markers (eg, *VIM*, *PAX2*). Although these 4 proliferating clusters are enriched in *RB1*^*−/−*^ homozygous patient organoids, their presence also in the corrected wild-type and heterozygous organoids would argue against a dedifferentiation process induced by pRB depletion. Instead, such findings would be more in accordance with a block in/or delayed differentiation resulting in accumulation of progenitor cells at the expense of differentiated retinal cell types as observed herein for the amacrine (cluster 9), horizontal (cluster 30), and RGCs (cluster 20) (Fig. 7D). Corresponding with the differentiation block induced by pRB depletion, are also findings of reduced NRPC/T1 cluster 10, which gives rise to RGCs (cluster 20) that are also reduced in the patient homozygous retinal organoids. NRPC/T1 are thought to give rise to NRPCs/T2 too, which in turn generate horizontal and amacrine cells clusters 30 and 9, both reduced in the patient homozygous retinal organoids. Together these data suggest that the block in differentiation in the patient homozygous retinal organoids occurs before the emergence of NRPC/T1 cells. Our immunofluorescence data showed no co-localization of SNCG or PROX1 with apoptotic marker CASP3 in the *RB1*^*−/−*^ organoids (Supplementary Fig. S17), suggesting that a block in differentiation rather than clearance-based apoptosis is the most likely explanation for their marked reduction in homozygous organoids. It is worth noting that the differentiation block can also be observed in patient heterozygous retinal organoids, which like the homozygous organoids show an enrichment in 3 late RPC clusters (8, 11, 34) and a decrease in amacrine (cluster 9) and horizontal cell (cluster 30), although the latter are less pronounced that in the homozygous background and harder to pick up by immunofluorescence analysis. Nonetheless, these findings need to be further validated by frequent apoptosis-based assays to exclude the possibility that RGCs, amacrine and horizontal cell decrease is due to clearance-based apoptosis earlier during the differentiation process.

While the decrease in amacrine cluster 9 by single-cell RNA-Seq corroborated very nicely the immunofluorescence analysis, the decrease in horizontal and RGC clusters could not be easily reconciled with the increased percentage of proliferating SNCG^+^ and PROX1^+^ cells shown by immunofluorescence analysis at both days 90 and 150 of differentiation. Together these findings suggest that other clusters apart from horizontal and RGC clusters may express horizontal and RGC markers. A careful investigation of single-cell data, focusing on a typical RGC marker, *ELAVL3* shows its co-expression with *RXRγ* in the Rb (cluster 22, Supplementary Fig. S18A) only in the *RB1*^*−/−*^ homozygous patient organoids. We were able to validate the above results by immunofluorescence analysis (Supplementary Fig. S18B, C), allowing us to conclude that similarly to RB1-null hESC organoids, expression of RGC marker, SNCG and horizontal cell marker PROX1 is found in the Rb-like cell cluster.

Given that scRNA-Seq in both pRB-depleted organoids revealed a likely differentiation block, we compared if these was more pronounced in one of the models. To this end, data from scRNA-Seq of H9 RB1-null organoids were integrated with those from homozygous patient organoids (Supplementary Fig. S19A and Table S4). A side-by-side comparison of all clusters revealed an enrichment of 4 late RPC clusters (1, 8, 10, 17), proliferating NRPCs (cluster 5), and one cluster of proliferating Müller Glia cells (0) in the patient homozygous *RB1*^*−/−*^ organoids (Supplementary Fig. S19B), suggesting a more pronounced differentiation block in the patient *RB1*^*−/−*^ organoids.

### Drug Testing in pRB-Depleted Organoids

**Figure 8.**
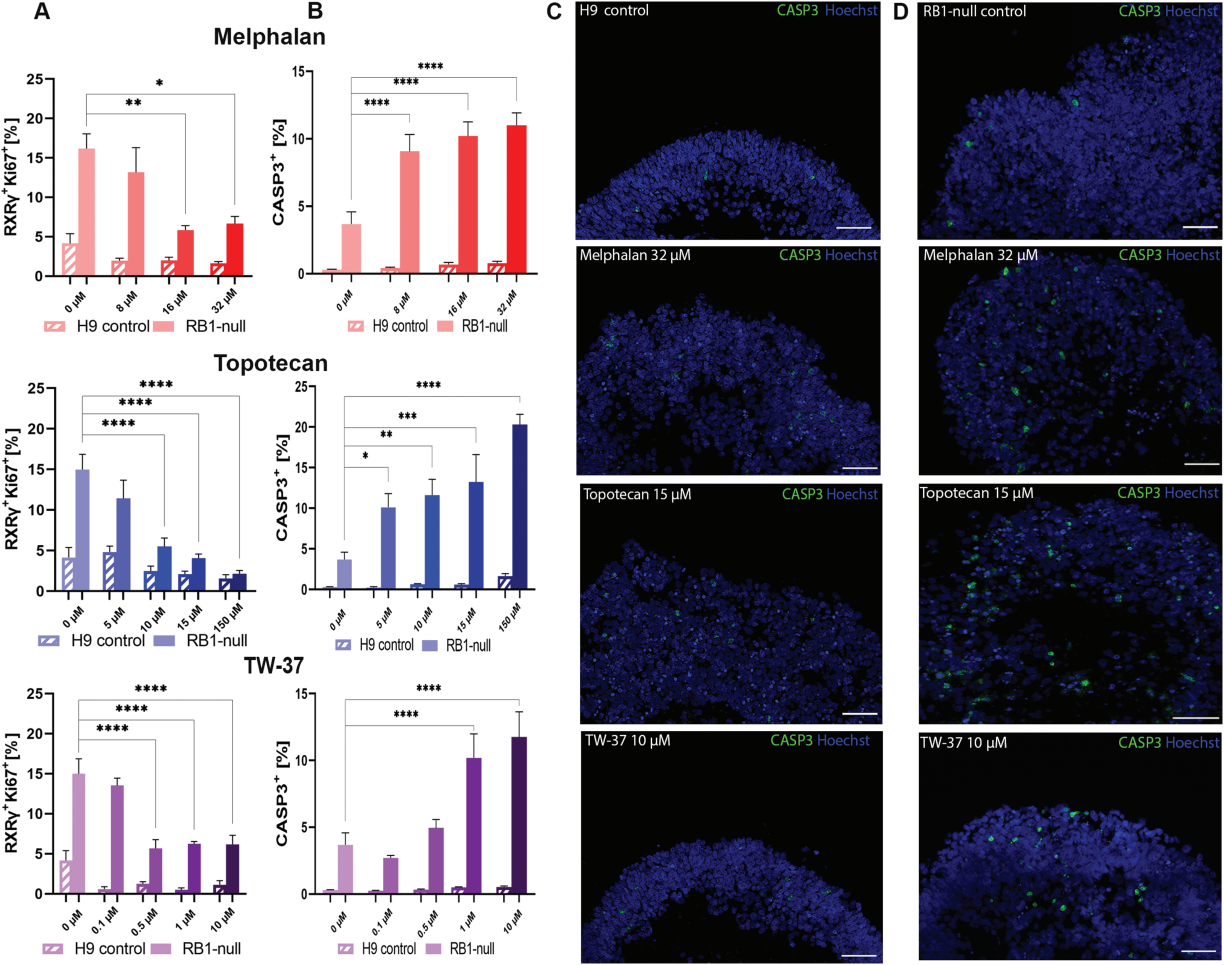
Assessment of clinically used chemotherapeutic agents for Rb treatment in control and RB1-null hESC organoids. (**A**) Bar graphs showing the percentage of proliferating cone precursors (RXRγ^+^Ki67^+^) in immunostained sections of treated organoids agent (Melphalan; 8, 16, 32 µM, Topotecan; 5, 10, 15, 150 µM, TW-37; 0.1, 0.5, 1, 10 µM alongside vehicle only sample; 0.1% DMSO). (**B**) Apoptotic response (cleaved-caspase-3; CASP3) after application of chemotherapeutic agents. Data presented as mean ± SEM (*n* = 5 sections from each biological replicate). Representative immunostaining of CASP3 counterstained with Hoechst for control (**C**) and RB1-null (**D**) hESC organoids; vehicle only, Melphalan 32 µM, Topotecan 15 µM, and TW-37 10 µM. Scale bars = 50 µm.

To assess the application of wild-type and pRB-depleted organoids for testing therapeutic agents, we incubated the organoids with varying doses of 3 drugs used in current treatments of Rb tumors: Melphalan, Topotecan, and TW-37 (Fig. 8, Supplementary Fig. S20). Melphalan is an alkylating agent that is highly effective against Rb, but high concentrations^52^ are needed to reach its metronomic IC50 (50% inhibitory concentration) in vitro, attainable only after intraarterial or intravitreal chemotherapy.^53^ Topotecan is a topoisomerase inhibitor, which prevents topoisomerase-I from re-ligating the nicked DNA strand, resulting in DNA damage and cell death.^54^ It is effective against Rb in combination with Melphalan. Bcl-2 inhibitors such as TW-37 act by competing with pro-apoptotic proteins (such as Bid, Bim, and Bad) and induce apoptosis.^55^ Day 150 wild-type and pRB-depleted hESC and iPSC organoids were incubated with each of these 3 drugs for 72 hours. To assess if these drugs were acting on proliferating cone precursors in both RB1-null and patient-derived *RB1*^*−/−*^organoids, we performed quantitative immunofluorescence analysis revealing 16-32 µM Melphalan, 10-150 µM Topotecan, and 0.5, 1 and 10 µM TW-37 to be the most effective doses in RB1-null organoids, as they significantly reduced the percentage of proliferating cone precursors to similar levels found within the hESC-control organoids (Fig. 8A). In addition to the above concentrations, we found 8 µM Melphalan and 5 µM Topotecan to significantly reduce the percentage of RXRƴ^+^ Ki67^+^ cells in iPSC-organoids (Supplementary Fig. S20A). We did not detect significant changes in the proliferating cone precursors of hESC- or iPSC-derived wild-type organoids (Fig. 8A, Supplementary Fig. S20A). To assess the drug specificity, we also assessed cell killing in wild-type and pRB-depleted organoids (Fig. 8B-D, Supplementary Fig S20B-D). Melphalan and Topotecan did cause an increase in percentage of Caspase-3^+^ apoptotic cells in all concentrations tested, while TW-37 did so only in the 2 highest concentrations in both RB1-null and patient-derived RB1^*−/−*^ organoids (1 and 10 µM; Fig. 8B, D, Supplementary Fig. S20B, D). The level of Caspase-3^+^ apoptotic cells in hESC-derived wild-type organoids remained very similar to untreated control (Fig. 8B, C), whereas in iPSC-derived wild type the highest concentration of Melphalan (32 µM) and Topotecan (150 µM) significantly increased Caspase-3^+^ apoptotic cells (Supplementary Fig. S20B, C). These assays in combination, point to 16 µM Melphalan, 10 µM Topotecan, and 1 µM TW-37 as most effective in lowering the level of proliferating cone precursors in both hESC and iPSC models, while retaining the healthy tissue unaffected. Together these data suggest that RB1-null and patient-derived *RB1*^*−/−*^organoids provide a useful platform for testing current and new Rb treatments.

## Discussion

The product of *RB1* gene is a tumor suppressor and regulator of cell cycle progression, affected in many malignancies^56^ and classified as a causative factor in Rb.^57^ Present management protocols of Rb aim not only to protect the eye but also its visual function.^58^ Preserving eye-sight proves to be difficult in recurrent malignant growths, leading to extended retinal damage or even enucleation.^47,59,60^ Further progression in management protocols as well as design of new efficacious therapies require detailed understanding of Rb tumors at the molecular and cellular level. Advances in next generation sequencing are enabling detailed expression and epigenetic studies of tumors, providing molecular insights at the single-cell level. Our group^49^ and others^43,51,61^ are utilizing these tools to understand the composition of Rb tumor tissues and to determine how pRB inactivation results in aberrant RNA processing events, genome instability and changes in metabolism and mitochondria biogenesis. These insights however focus on the end stage of tumor development, which precludes detailed insights into the transcriptional events of each state cell transition during Rb development. To fill this gap, we report here the development of 2 Rb models generated through CRISPR/Cas9 inactivation of *RB1* gene in hESC and derivation of patient-specific iPSC line from a child with heterozygous mutation (c.2082delC) in the *RB1* gene, who developed bilateral malignant growths before the age of 4. Through CRISPR/Cas9 gene editing, isogenic wild type, and homozygous mutated iPSC lines were generated to enable comparison of patient phenotypes within the same genetic background. Retinal organoids generated from both models were characterized at the early stage (day 35), mid stage (day 90), and late stage (day 150) of differentiation, showing lack of pRB expression throughout. Both models shared 4 key features: (1) increased percentage of proliferating cone precursors (RXRγ^+^Ki67^+^); (2) increased percentage of proliferating (Ki67^+^) and apoptotic cells (Caspase-3^+^), although very few apoptotic cells were observed overall; (3) expression of RGC and horizontal cell markers in Rb-proliferating cones, and (4) accumulation of retinal progenitor cells at the expense of amacrine, horizontal and retinal ganglion cells, which suggests an important role for pRB in differentiation of these retinal cell lineages. The pRB-depleted retinal organoids generated from both models, displayed similar features to Rb tumors including mitochondrial cristae aberration and rosette-like structures. Importantly, cell growth in an anchorage-independent manner, indicative of cell transformation in vitro, was a feature of pRB-depleted organoids, but not those derived from wild type or *RB1* heterozygous organoids.

To define at the molecular level, the cell of origin responsible for the malignant transformation within the pRB-depleted retinal organoids, we performed single-cell RNA-Seq analysis, which pointed to the stressed proliferating cone precursors as the most likely starting cell population in the RB1-null hESC organoids, corroborating previous findings derived from knockdown of *RB1* in human fetal retinal cells.^21^ Although we could detect the Rb-like cluster in the homozygous *RB1*^*−/−*^ patient organoids, we could not find evidence of a retinoma-like cluster, which was present in the hESC RB1-null organoids. These findings corroborate published literature, which reports either presence of Rb by itself, or the co-existence of retinoma and Rb clusters in primary Rb tumors.^9^ Alternatively, they could relate to clonal variations due to epigenetic modifications occurring during the reprogramming process or mutations in genes such as *TP53*, acquired during pluripotent stem cell expansion.^62^ To ascertain whether the lack of retinoma is specific to the patient-specific pRb-depleted organoids, a larger number of iPSC clones from several patients with multiple *RB1* mutations would need to be differentiated and analyzed together with appropriate isogeneic controls.

We also noticed some differences between the hESC and iPSC pRB-depleted organoids. For example, a reduction in percentage of amacrine cells was observed in the RB1-null hESC organoids, whilst a total lack of these cells was found in the homozygous patient *RB1*^*−/−*^ organoids. Similarly, a marked reduction in RGCs and horizontal cells was evident from the single-cell RNA-Seq analysis of homozygous patient-specific compared with RB1-null hESC-derived retinal organoids. Importantly, an enhanced accumulation of proliferating retinal progenitor cells was observed in the homozygous patient *RB1*^*−/−*^ organoids, which lacked the retinoma-like cluster observed in the hESC pRb-depleted organoids. The differences between the 2 models cannot be easily attributed to differences in *RB1* inactivation (knockdown vs specific mutation) as in both cases a complete lack of pRB protein was detected. Equally they cannot be explained by cell line- or differentiation-specific acquisition of chromosomal abnormalities as genomic stability assays indicated a normal karyotype for both RB1-null hESC and homozygous patient organoids up to day 150 of differentiation (data not shown). Most likely these small differences are due to the epigenetic starting point of hESC versus iPSC lines or iPSC clonal variations and to address these in detail, studies with a larger number of iPSC clones from a larger number of patients with *RB1* mutations and hESC-derived organoids need to be conducted.

During retinal organoid differentiation, we noticed a significant increase in the percentage of SNCG^+^Ki67^+^ and PROX1^+^Ki67^+^ cells in both pRB-depleted organoid models compared with controls, which could be interpreted as increased proliferation of RGCs and horizontal cells since SNCG and PROX1 are characteristic markers of these 2 retinal cell types. Yet the single-cell RNA-Seq analysis indicated a decrease in the fraction of RGCs and horizontal cells and moreover revealed the expression of these 2 markers in the Rb cell clusters. Published studies of human Rb tumors have provided evidence of expression of genes associated with multiple retinal cell types,^63,64^ although these conclusions were based on bulk RNA-Seq studies, leaving open the question of whether multiple retinal cell types act as cell of origin for Rb or a single-cell type expressing multiple retinal marker genes fulfills this role. Single-cell analysis makes it possible to dissect these 2 questions, providing clear evidence that Rb cones do also express RGC and horizontal cell markers, a new finding which we have also corroborated by immunofluorescence assays. A question that follows from these new findings is: Are these truly cone precursors, or have they dedifferentiated to an earlier progenitor enabling expression of multiple retinal cell types? Our single-cell RNA-Seq data would argue against this as in both retinal organoid models, the Rb clusters not only expressed key cone photoreceptor precursor markers (eg, *RXRγ*, *PDE6H,* and *ARR3*) but also clustered next to other photoreceptor clusters (cone and rod precursors emerging as part of normal retinal differentiation). We thus conclude that the Rb cones acquire expression of RGC and horizontal cell markers upon entry into cell cycle. Findings published while this manuscript was under review by Liu et al^51^ showing expression of neuronal/ganglion-cell markers in the less differentiated cones in 102 Rb tumor samples fully corroborate this conclusion.

Work performed in mouse models has indicted that differentiated interneurons are responsible for development of metastatic Rb in mice.^65^ On the contrary, our work shows a reduction in horizontal, amacrine and retinal ganglion cells in both pRB-depleted organoids. It is conceivable that pRB dysfunction could interfere with retinal cell differentiation or their survival. Previous studies have shown that transfection of either AP2α or AP2β constructs in Rb cells induces apoptosis, suggesting incompatibility between expression of AP2α in amacrine cells and survival in pRB-depleted organoids.^66^ We observed very few cells with co-expression of Caspase-3 and AP2α in RB1-null hESC organoids and none with co-expression of SNCG or PROX1 with Caspase-3. Instead, we observed in both models an accumulation of retinal progenitor cells at various stages of differentiation, which together with the decrease in RGCs, amacrine, and horizontal cells, suggests a block in differentiation. Our scRNA-Seq data analyses suggest that this block may occur either before or after the emergence of transient neurogenic RPCs (T1), which are thought to give rise to these retinal cell lineages. These findings are entirely in agreement with the published evidence of defects in rod differentiation^67^ in the absence of Rb1 and starburst amacrine cells upon combined loss of Rb1 and E2F3a^68^ in mouse models and provide for the first-time important insights into the role of pRB during human retinal differentiation. Importantly, we also observed accumulation of some retinal progenitor cell clusters and a decrease in horizontal and amacrine cells in the heterozygous patient organoids, suggesting that even a 50% reduction in pRB expression can disrupt the differentiation of these 2 retinal cell types. It is of interest to note, that a very recent single-cell RNA-Seq study of human Rb tumors and those arising from intravitreal injections of patient-specific RB1 organoids revealed a bias toward retinal progenitor cells and rods.^43^ The prevalence of progenitor cells following pRb inactivation both in vitro as shown in our study and in vivo suggest a role for pRb in retinal cell differentiation; however, lineage tracing studies are needed to fully address this important notion.

Together our data provide robust evidence that RB1-null hESC and patient-specific homozygous *RB1*^*−/−*^ organoids mimic the development and malignant transformation that occurs in vivo resulting in Rb tumors. To this end, we went on to assess whether these pRB-depleted organoid models could serve as a platform for drug screening, by incubating the pRB-depleted organoids with 3 chemotherapeutic agents namely Melphalan, Topotecan, and TW-37. All 3 compounds resulted in a significant decrease in the percentage of proliferating cone precursors (RXRγ^+^Ki67^+^) to the level found in control organoids as well as an increase in the percentage of apoptotic cells. No significant changes were observed in the control organoids, apart from Melphalan and Topotecan at highest concentration tested. Although more work employing a larger number of drugs and organoids from patients with various mutations in *RB1* gene is needed, our initial data corroborate the validity of these in vitro models as suitable platforms for drug and toxicity screening prior to large clinical trials.

In conclusion, we have developed and fully characterized an hESC RB1-null and an iPSC *RB1* patient-specific retinal organoid model and shown that both are characterized by a significant increase in the fraction of proliferating cone precursors (RXRγ^+^Ki67^+^), which were defined as Rb-like clusters by single-cell RNA-Seq analysis. We have shown that Rb proliferating cones express markers of RGCs and horizontal cells, which could help to better characterize these tumors with possible therapeutic implications. Importantly, we have demonstrated that these 2 models recapitulate the development of Rb in vivo and can be successfully used for drug and toxicology testing.

## Supplementary Material

szac008_suppl_Supplementary_Figure_S1

szac008_suppl_Supplementary_Figure_S2

szac008_suppl_Supplementary_Figure_S3_1

szac008_suppl_Supplementary_Figure_S3_2

szac008_suppl_Supplementary_Figure_S4

szac008_suppl_Supplementary_Figure_S5

szac008_suppl_Supplementary_Figure_S6

szac008_suppl_Supplementary_Figure_S7

szac008_suppl_Supplementary_Figure_S8

szac008_suppl_Supplementary_Figure_S9

szac008_suppl_Supplementary_Figure_S10

szac008_suppl_Supplementary_Figure_S11

szac008_suppl_Supplementary_Figure_S12

szac008_suppl_Supplementary_Figure_S13

szac008_suppl_Supplementary_Figure_S14_1

szac008_suppl_Supplementary_Figure_S14_2

szac008_suppl_Supplementary_Figure_S15_1

szac008_suppl_Supplementary_Figure_S15_2

szac008_suppl_Supplementary_Figure_S16

szac008_suppl_Supplementary_Figure_S17

szac008_suppl_Supplementary_Figure_S18

szac008_suppl_Supplementary_Figure_S19

szac008_suppl_Supplementary_Figure_S20

szac008_suppl_Supplementary_Figure_S21

szac008_suppl_Supplementary_Table_S1

szac008_suppl_Supplementary_Table_S2

szac008_suppl_Supplementary_Table_S3

szac008_suppl_Supplementary_Table_S4

szac008_suppl_Supplementary_Table_S5

szac008_suppl_Supplementary_Table_S6

szac008_suppl_Supplementary_Legends

## Data Availability

The data underlying this article are available at GEO (GSE173447).
