# Supplementary figures and images for: pRB-Depleted Pluripotent Stem Cell Retinal Organoids Recapitulate Cell State Transitions of Retinoblastoma Development and Suggest an Important Role for pRB in Retinal Cell Differentiation
Source: Stem Cells Transl Med. 2022 Mar 23;11(4):415–33. doi: 10.1093/stcltm/szac008 (PMC9052432; doi:10.1093/stcltm/szac008)

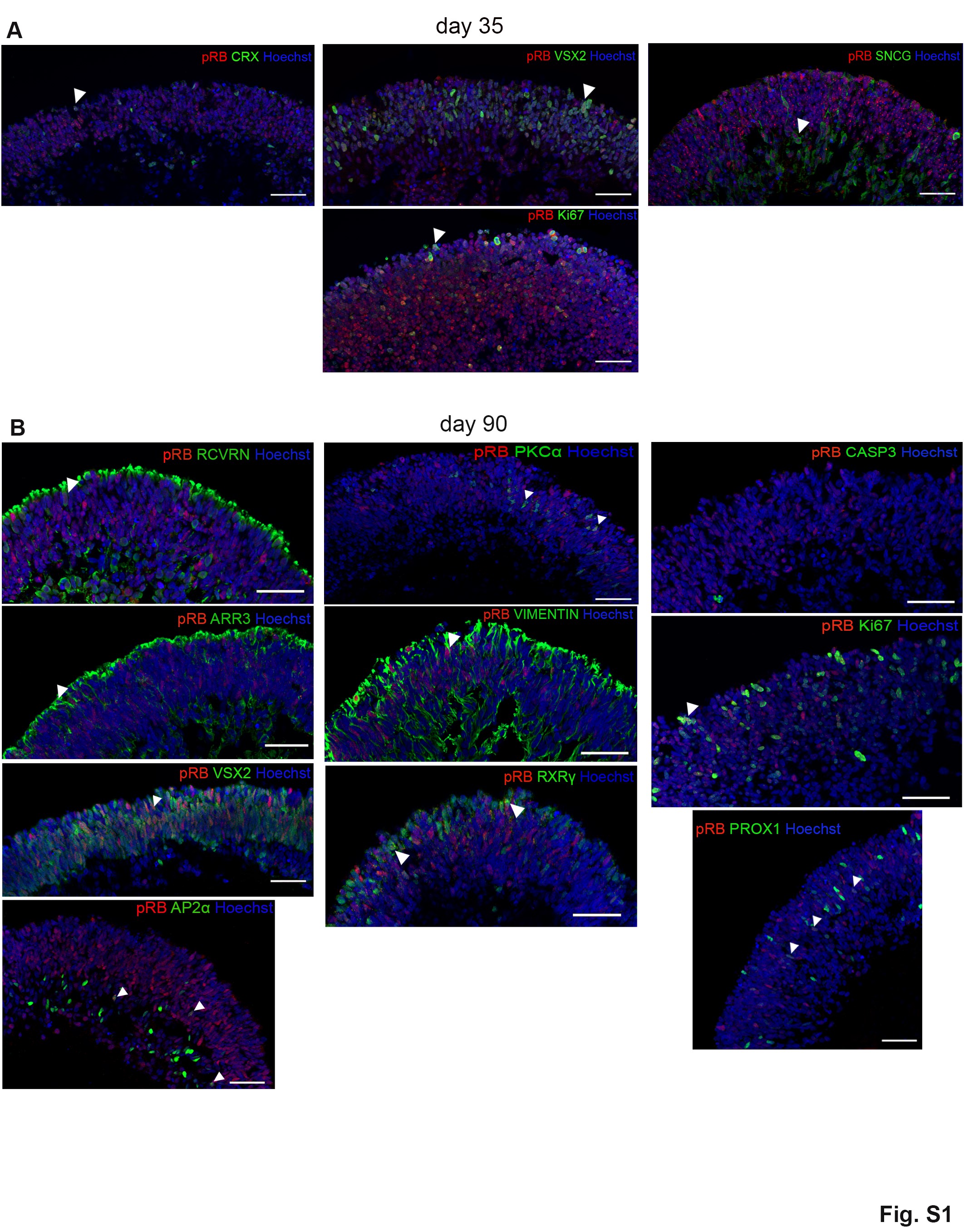

Supplement: szac008_suppl_Supplementary_Figure_S1 [file szac008_suppl_supplementary_figure_s1.jpeg]

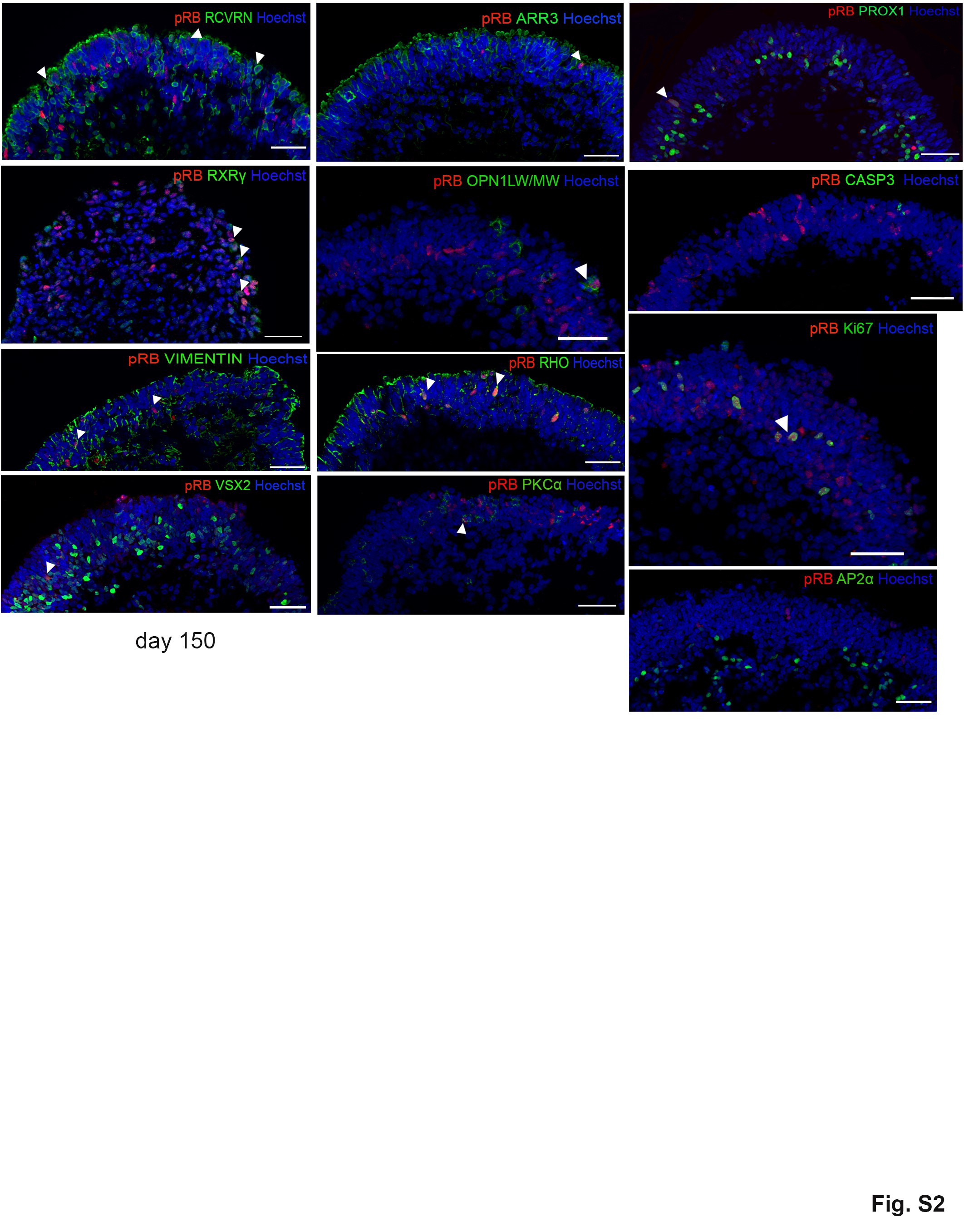

Supplement: szac008_suppl_Supplementary_Figure_S2 [file szac008_suppl_supplementary_figure_s2.jpeg]

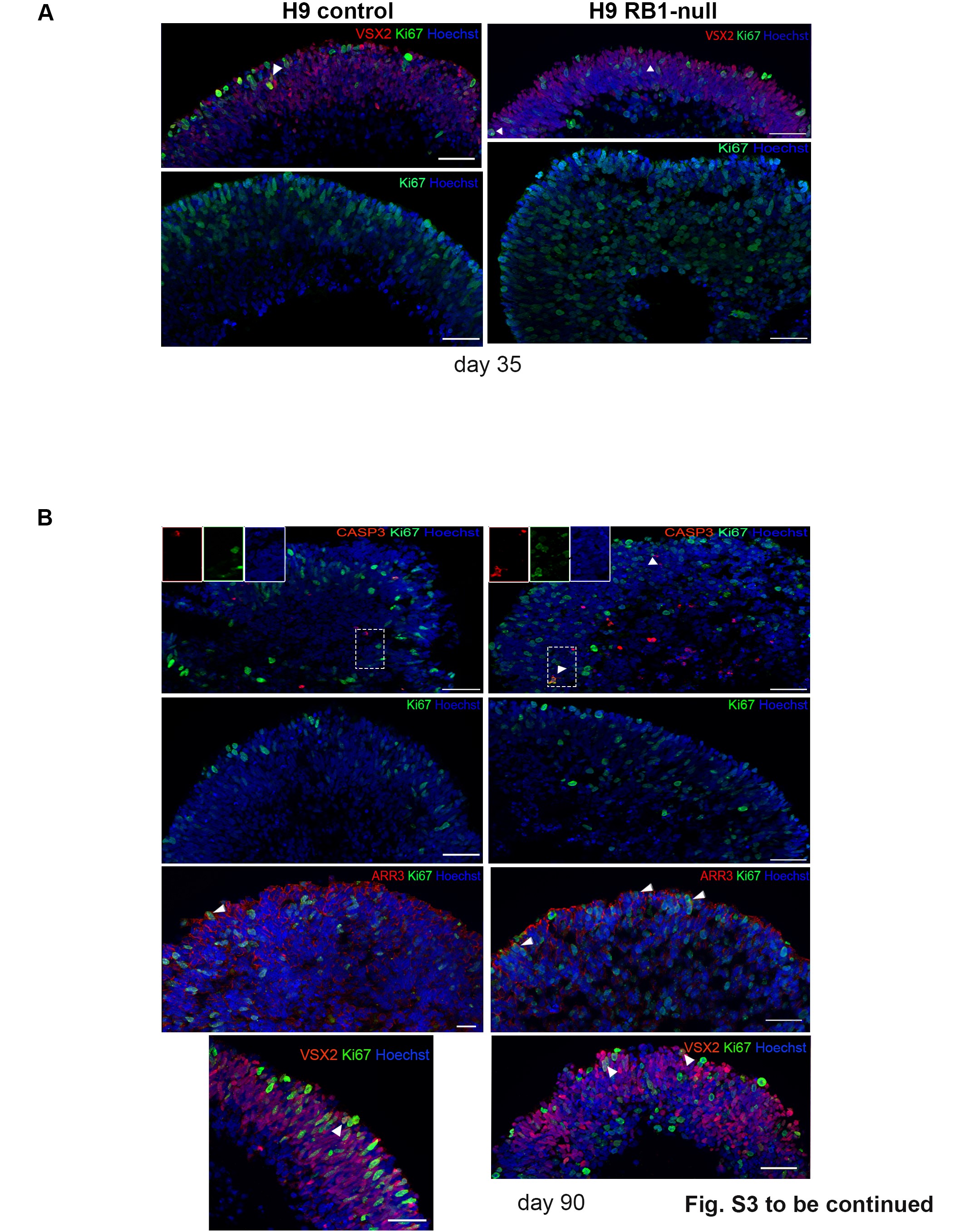

Supplement: szac008_suppl_Supplementary_Figure_S3_1 [file szac008_suppl_supplementary_figure_s3_1.jpeg]

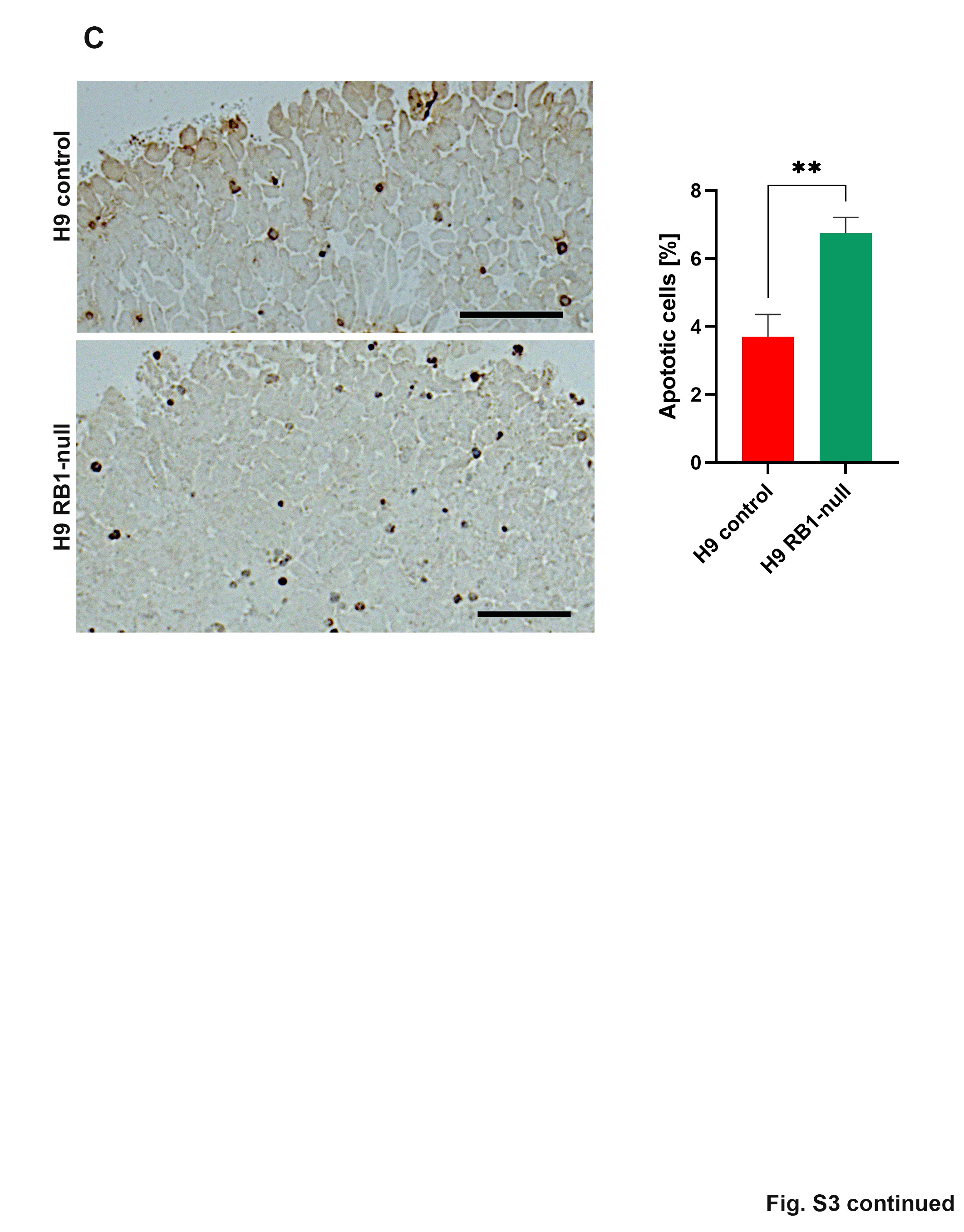

Supplement: szac008_suppl_Supplementary_Figure_S3_2 [file szac008_suppl_supplementary_figure_s3_2.jpeg]

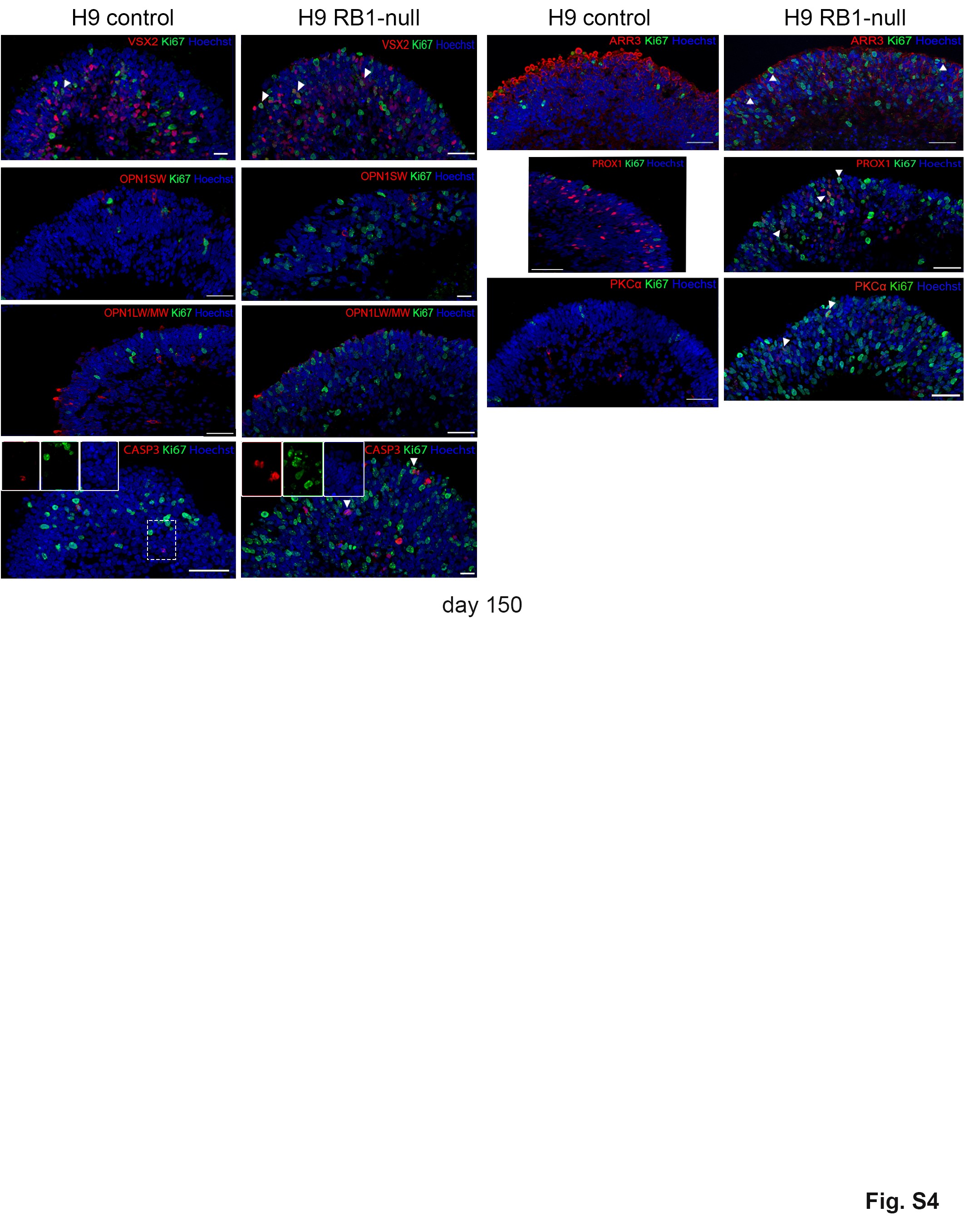

Supplement: szac008_suppl_Supplementary_Figure_S4 [file szac008_suppl_supplementary_figure_s4.jpeg]

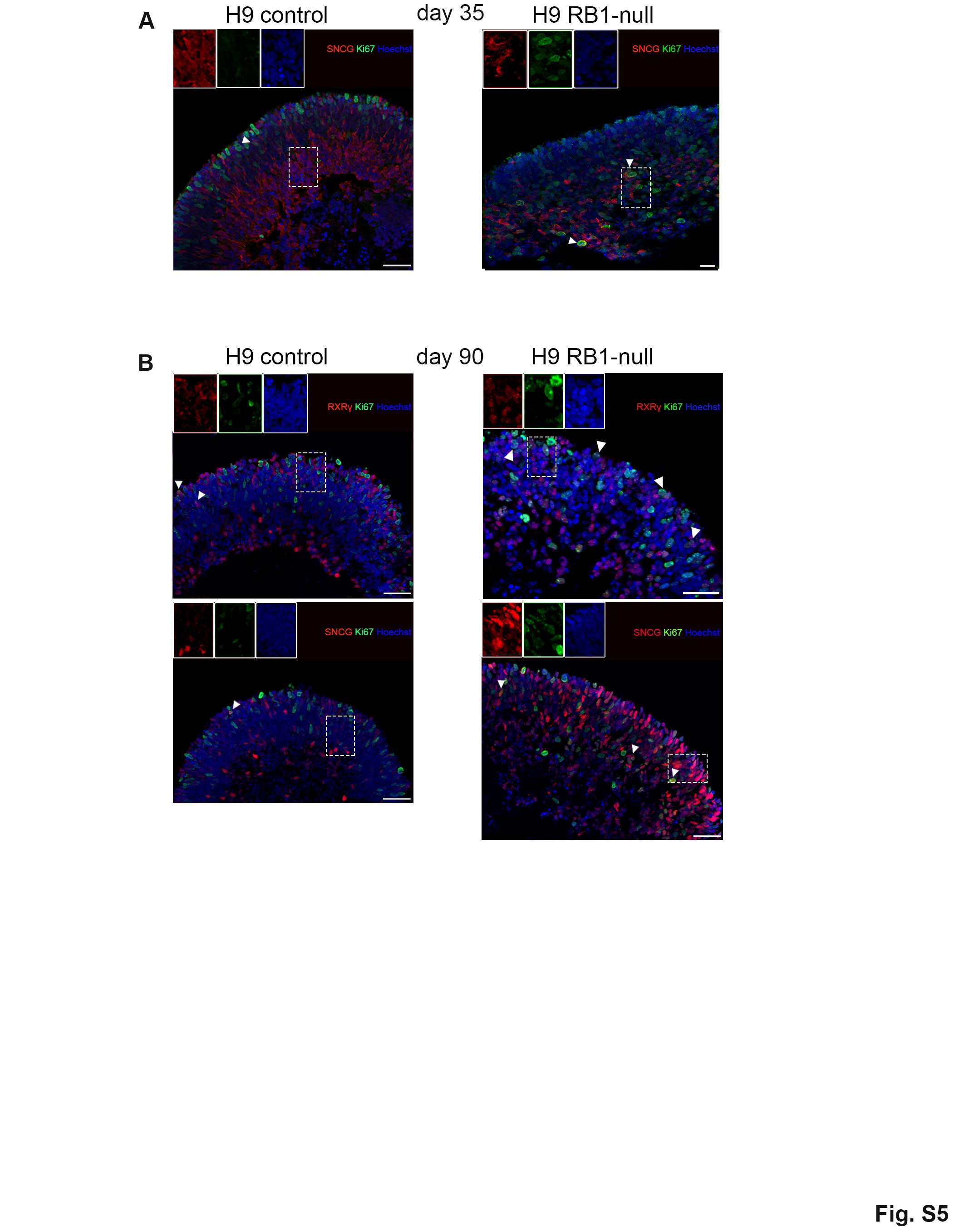

Supplement: szac008_suppl_Supplementary_Figure_S5 [file szac008_suppl_supplementary_figure_s5.jpeg]

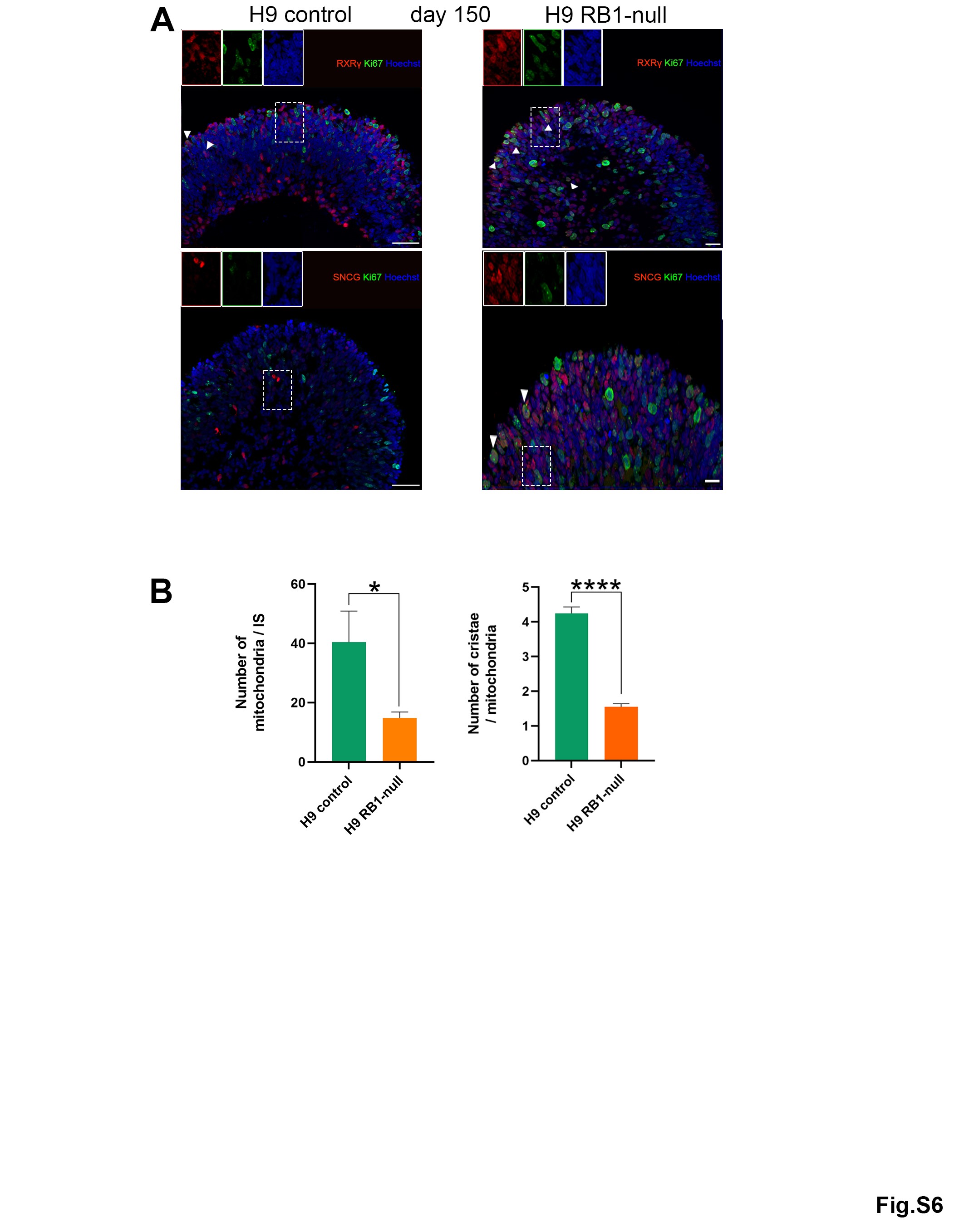

Supplement: szac008_suppl_Supplementary_Figure_S6 [file szac008_suppl_supplementary_figure_s6.jpeg]

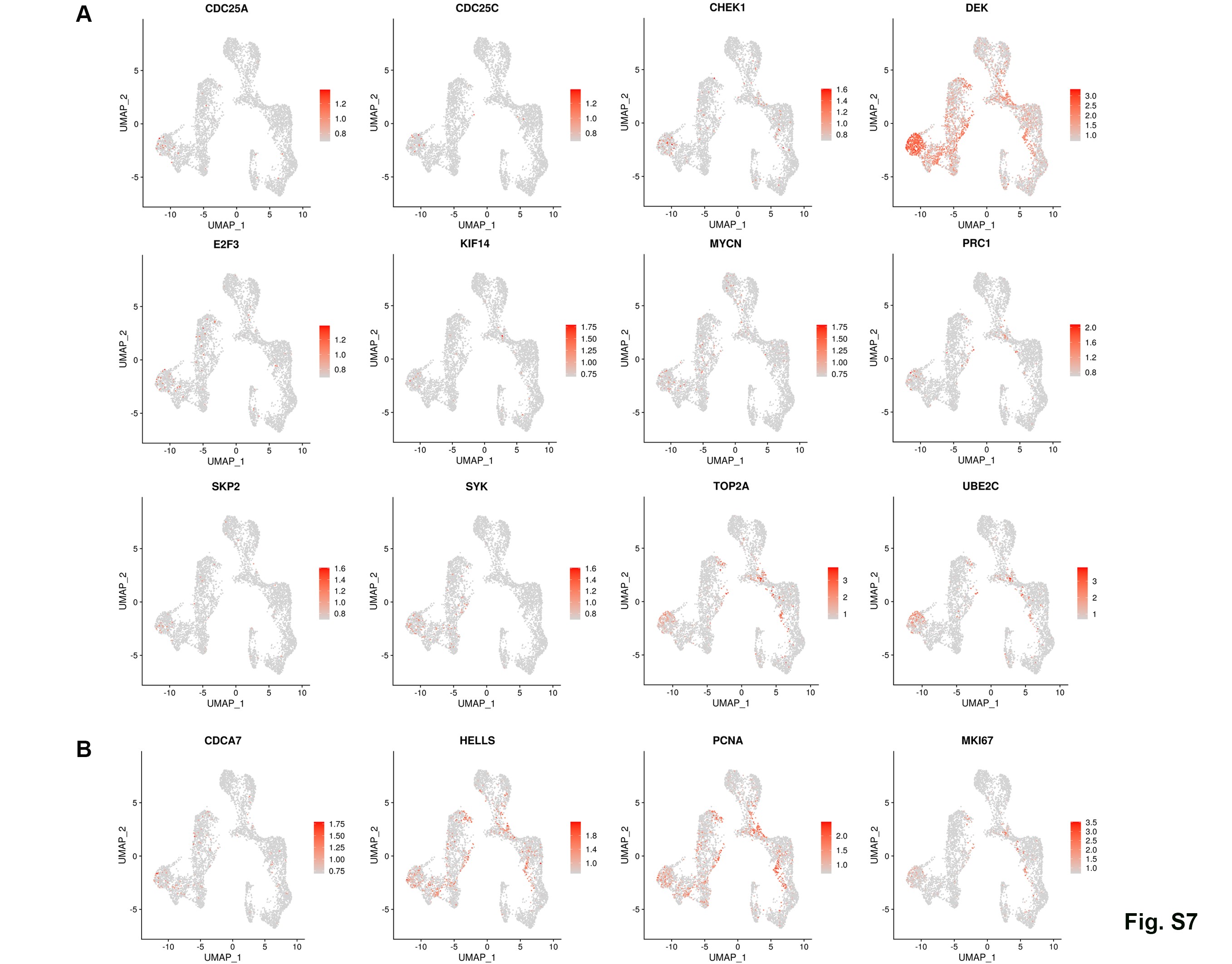

Supplement: szac008_suppl_Supplementary_Figure_S7 [file szac008_suppl_supplementary_figure_s7.jpeg]

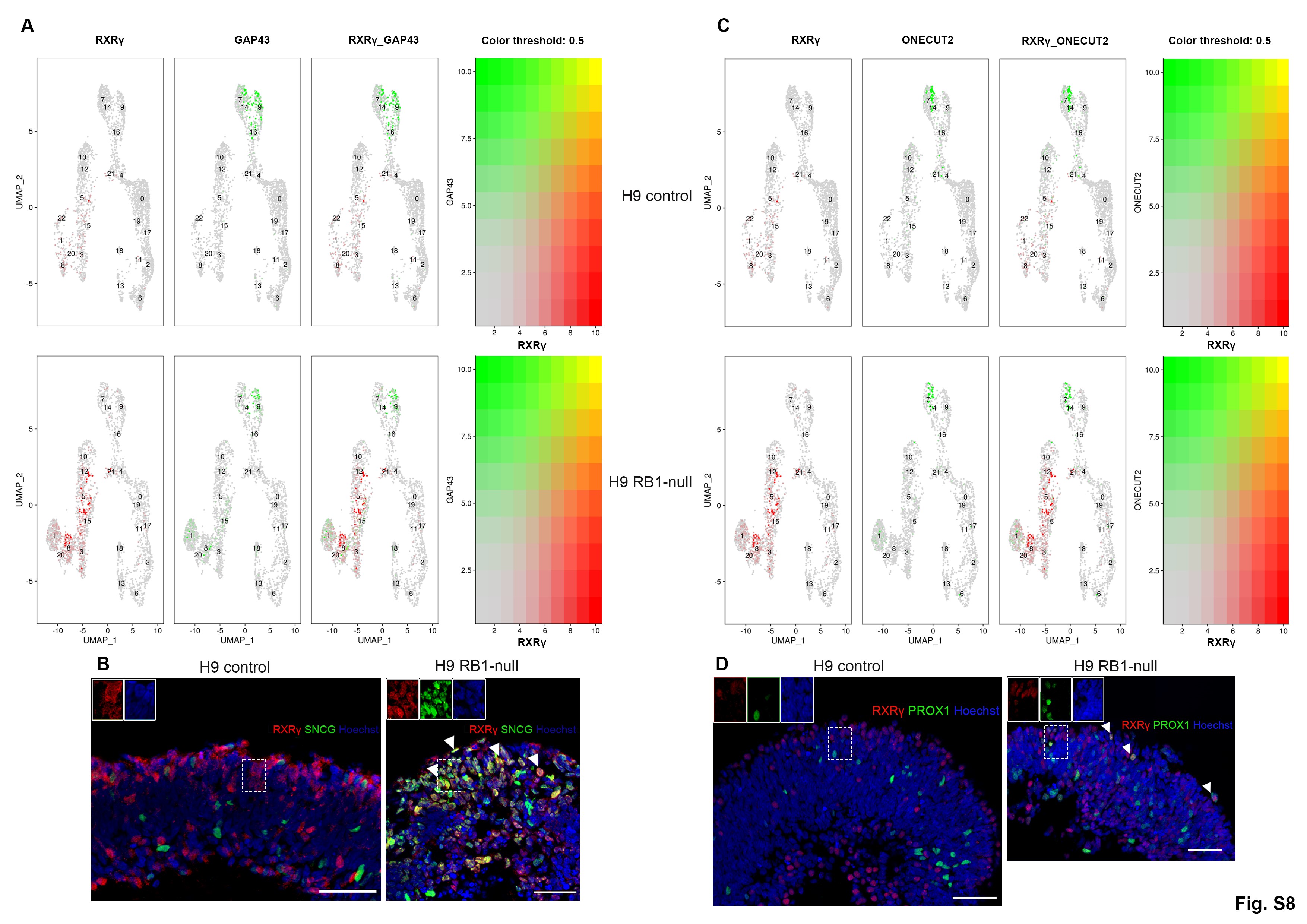

Supplement: szac008_suppl_Supplementary_Figure_S8 [file szac008_suppl_supplementary_figure_s8.jpeg]

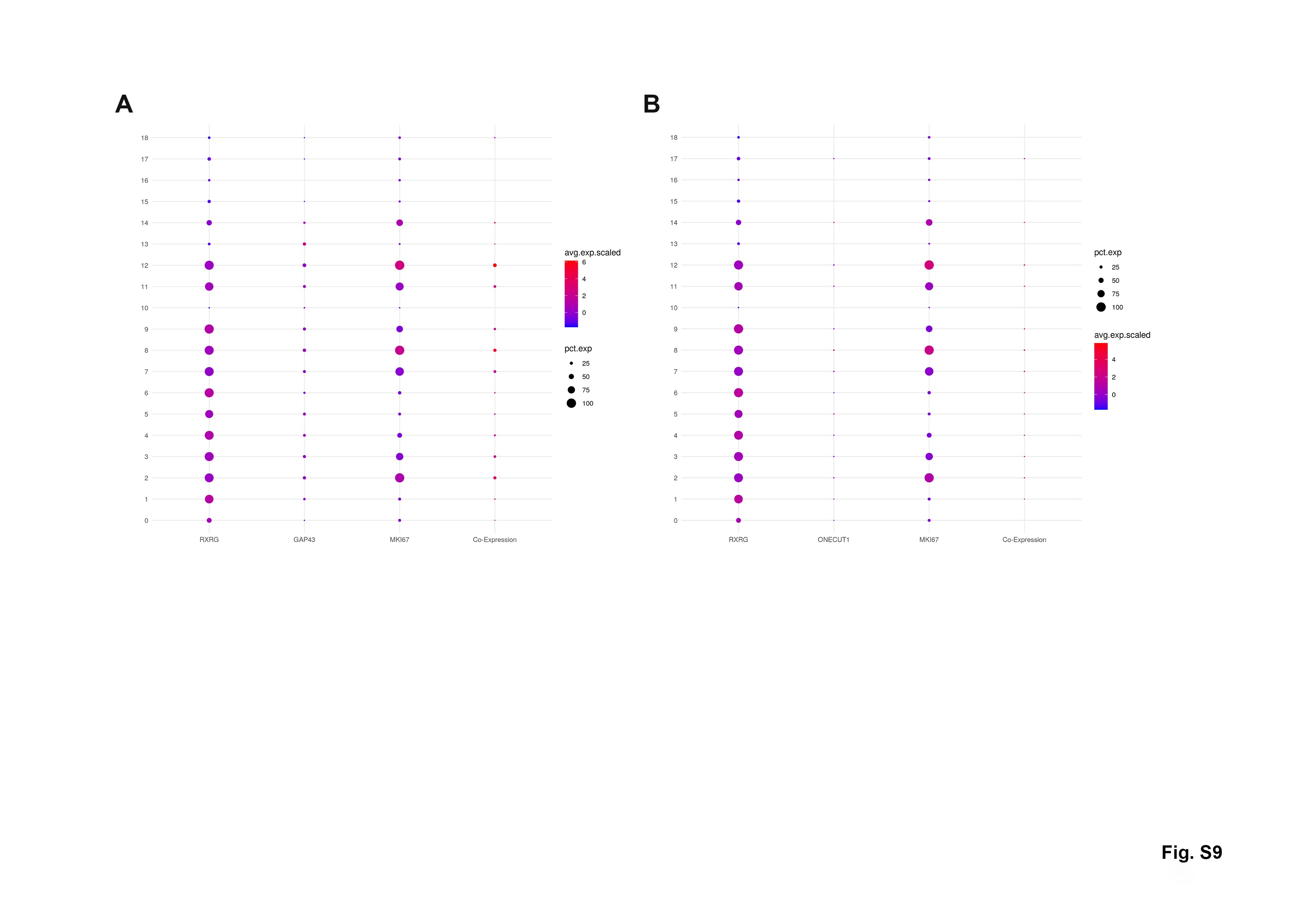

Supplement: szac008_suppl_Supplementary_Figure_S9 [file szac008_suppl_supplementary_figure_s9.jpeg]

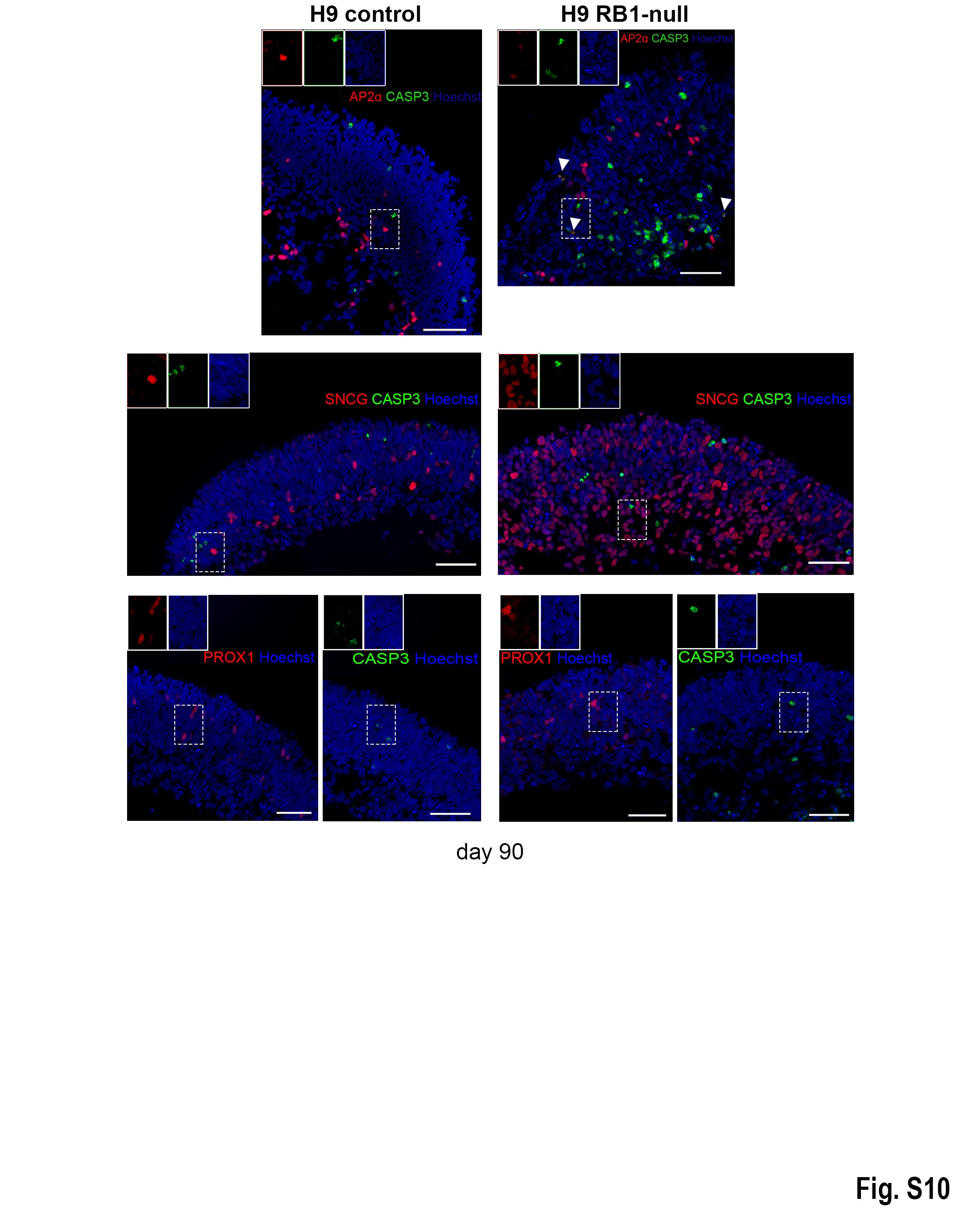

Supplement: szac008_suppl_Supplementary_Figure_S10 [file szac008_suppl_supplementary_figure_s10.jpeg]

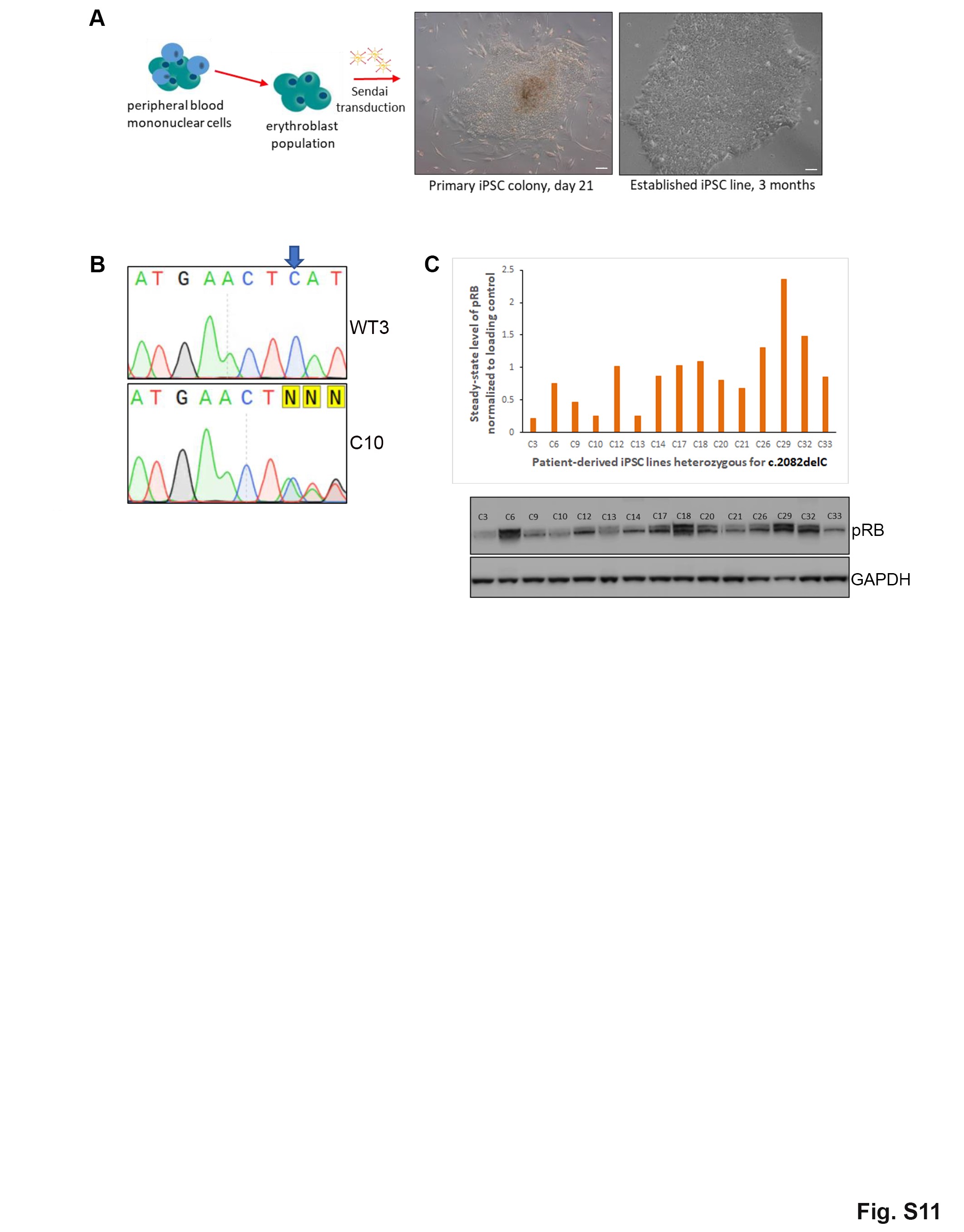

Supplement: szac008_suppl_Supplementary_Figure_S11 [file szac008_suppl_supplementary_figure_s11.jpeg]

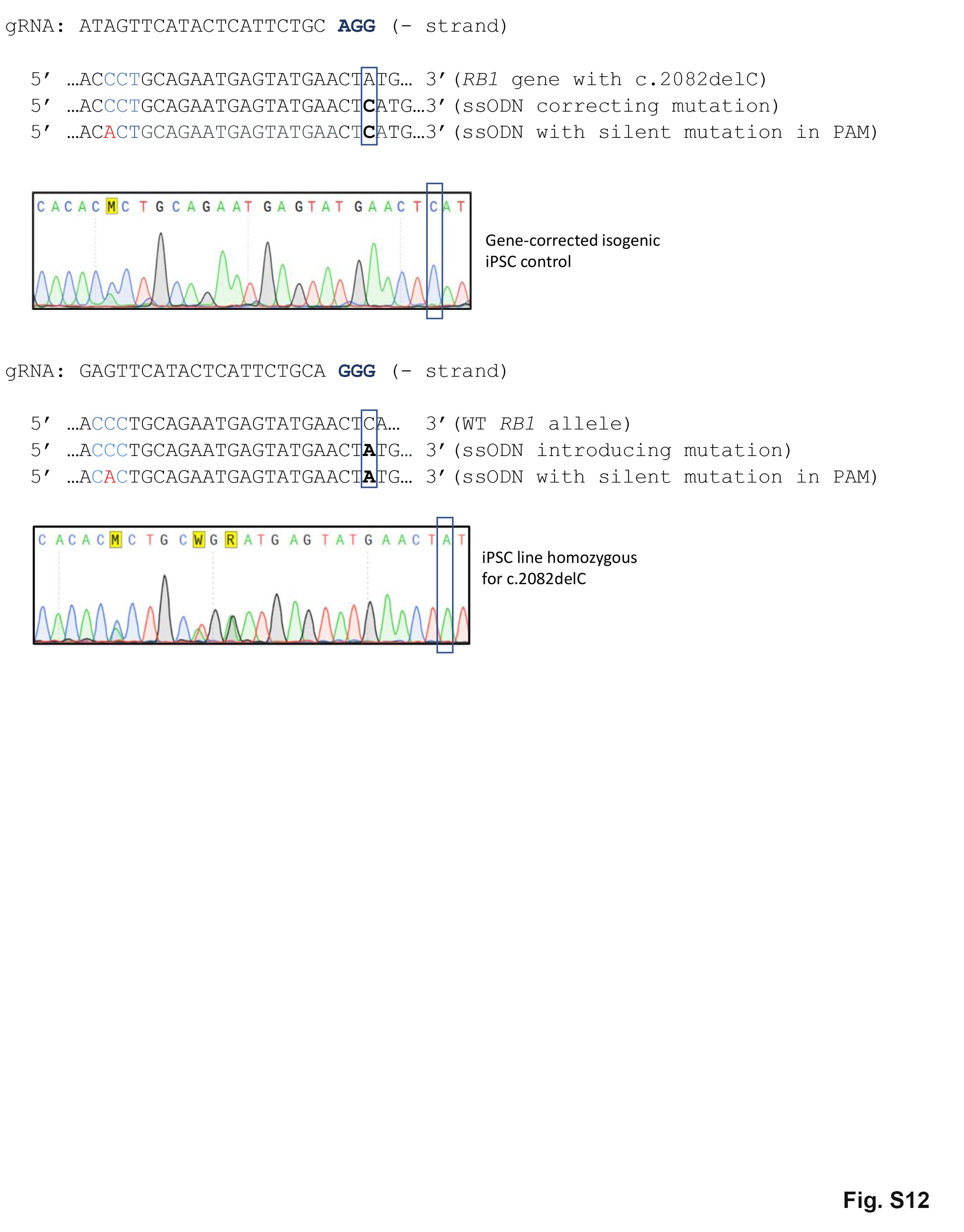

Supplement: szac008_suppl_Supplementary_Figure_S12 [file szac008_suppl_supplementary_figure_s12.jpeg]

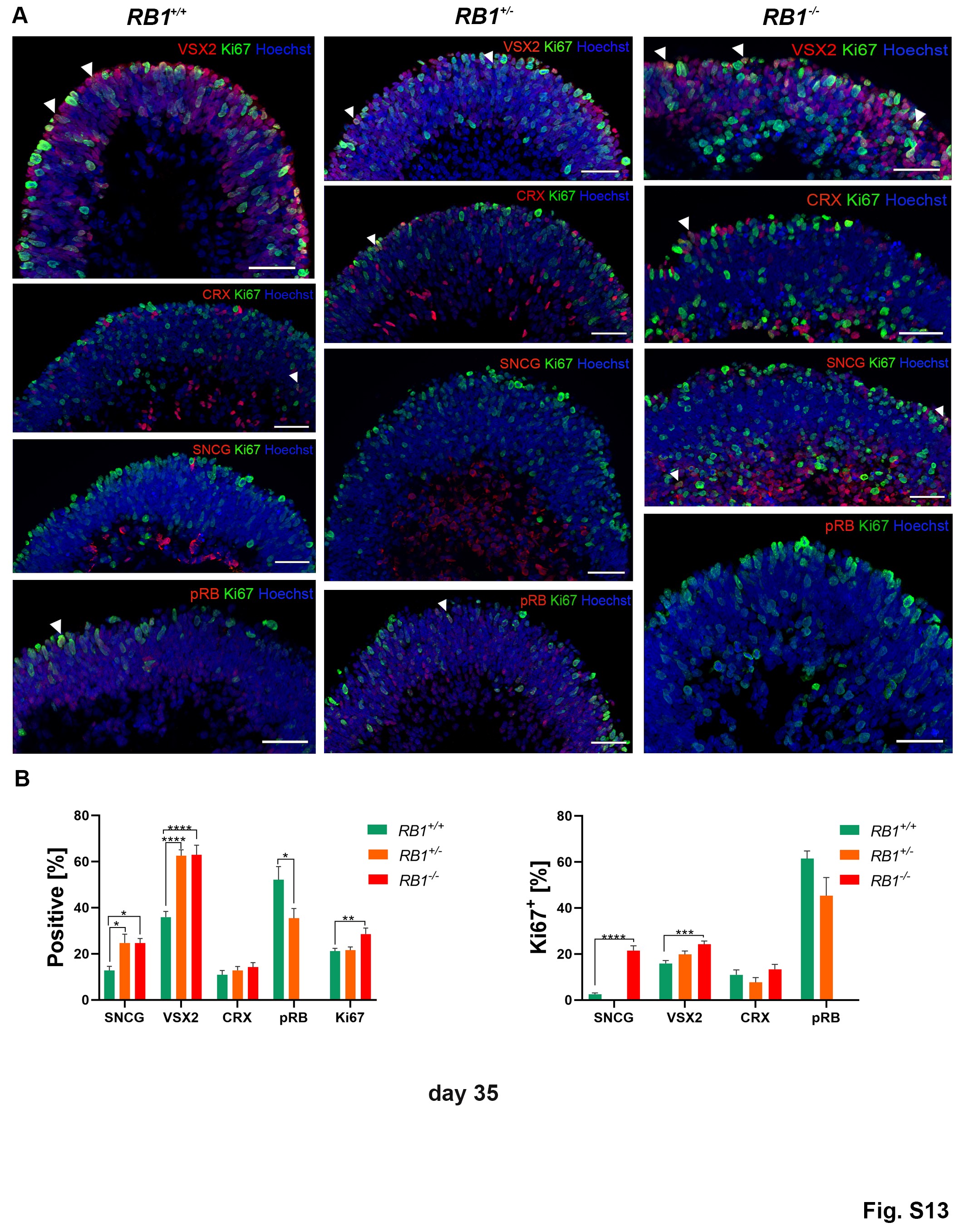

Supplement: szac008_suppl_Supplementary_Figure_S13 [file szac008_suppl_supplementary_figure_s13.jpeg]

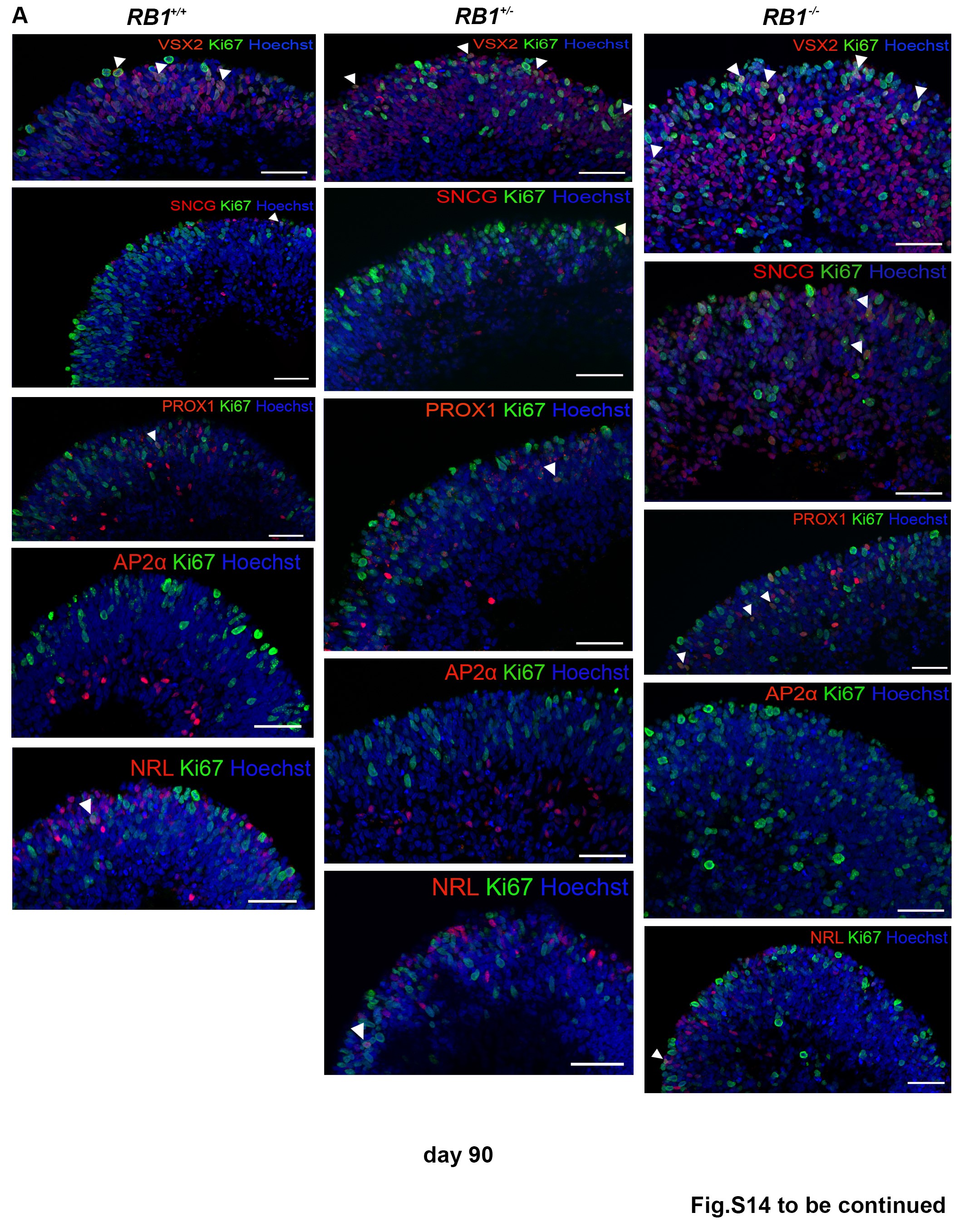

Supplement: szac008_suppl_Supplementary_Figure_S14_1 [file szac008_suppl_supplementary_figure_s14_1.jpeg]

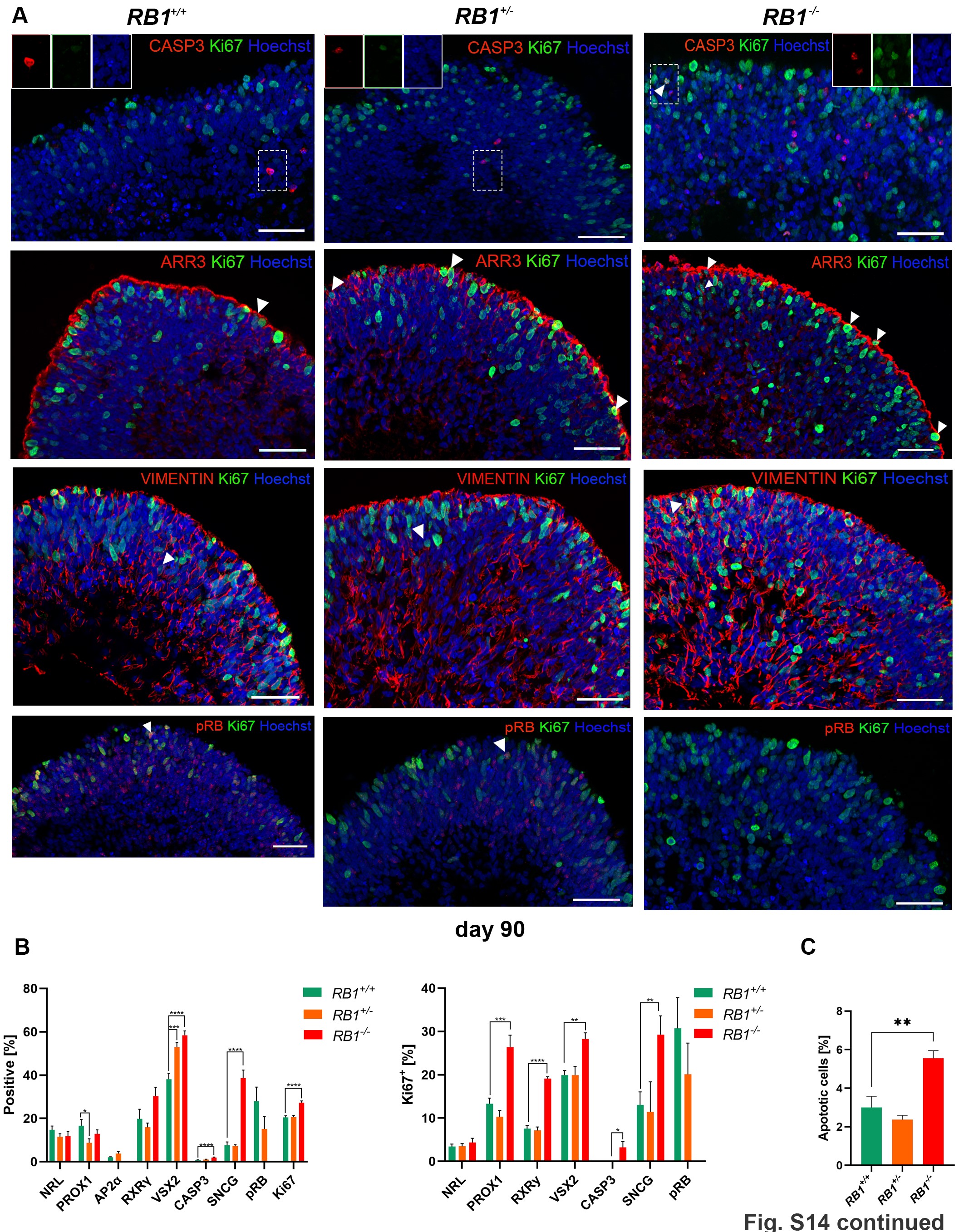

Supplement: szac008_suppl_Supplementary_Figure_S14_2 [file szac008_suppl_supplementary_figure_s14_2.jpeg]

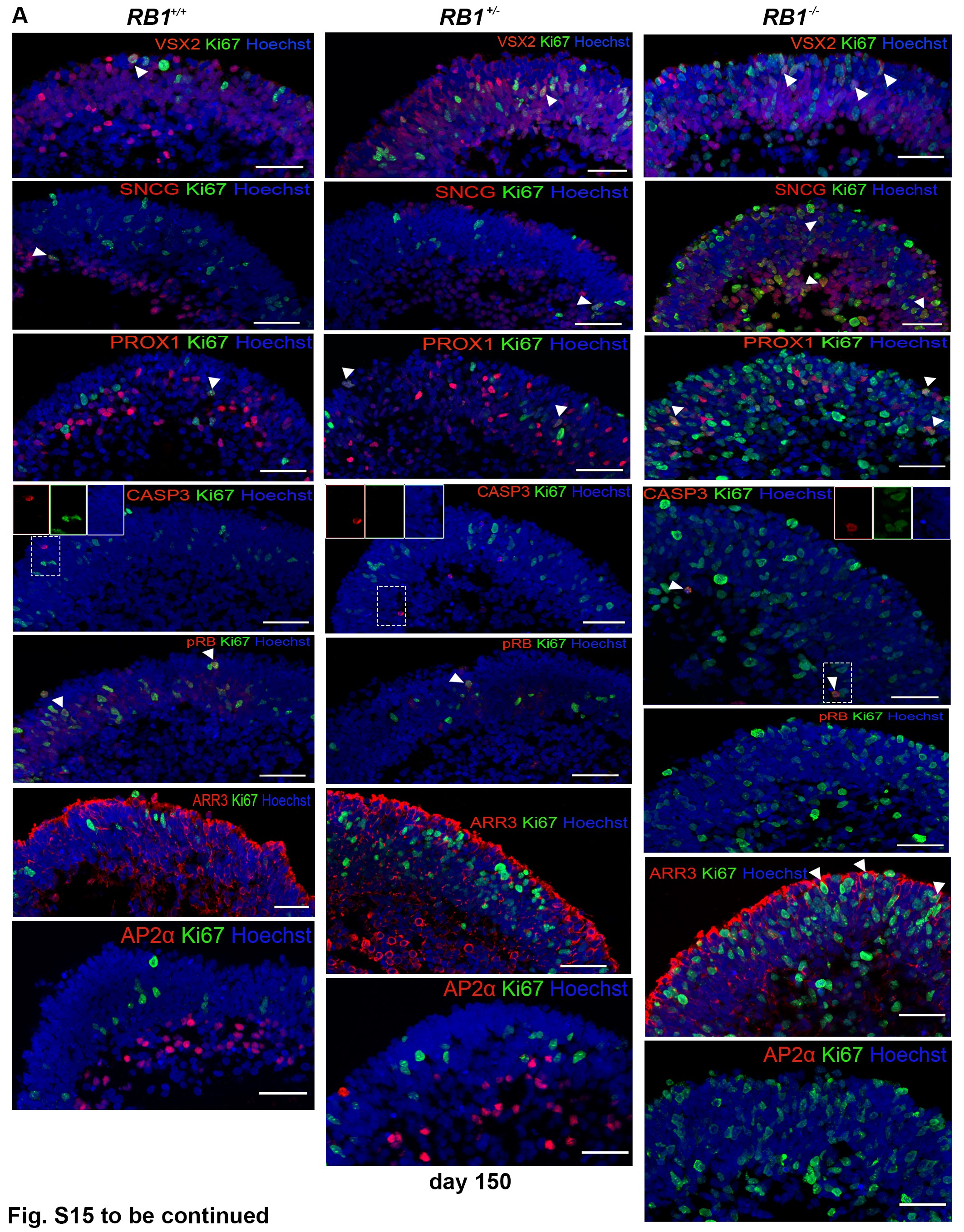

Supplement: szac008_suppl_Supplementary_Figure_S15_1 [file szac008_suppl_supplementary_figure_s15_1.jpeg]

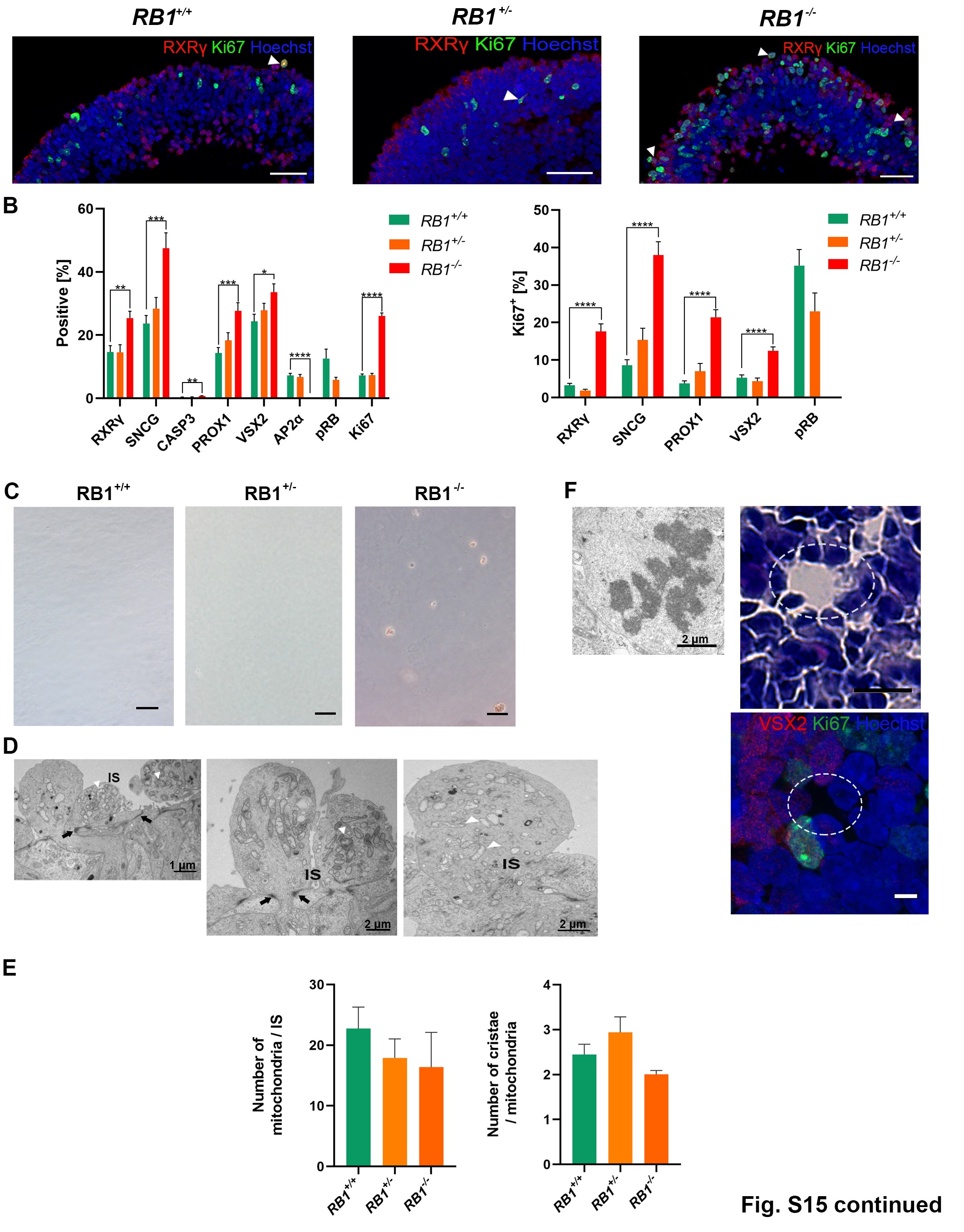

Supplement: szac008_suppl_Supplementary_Figure_S15_2 [file szac008_suppl_supplementary_figure_s15_2.jpeg]

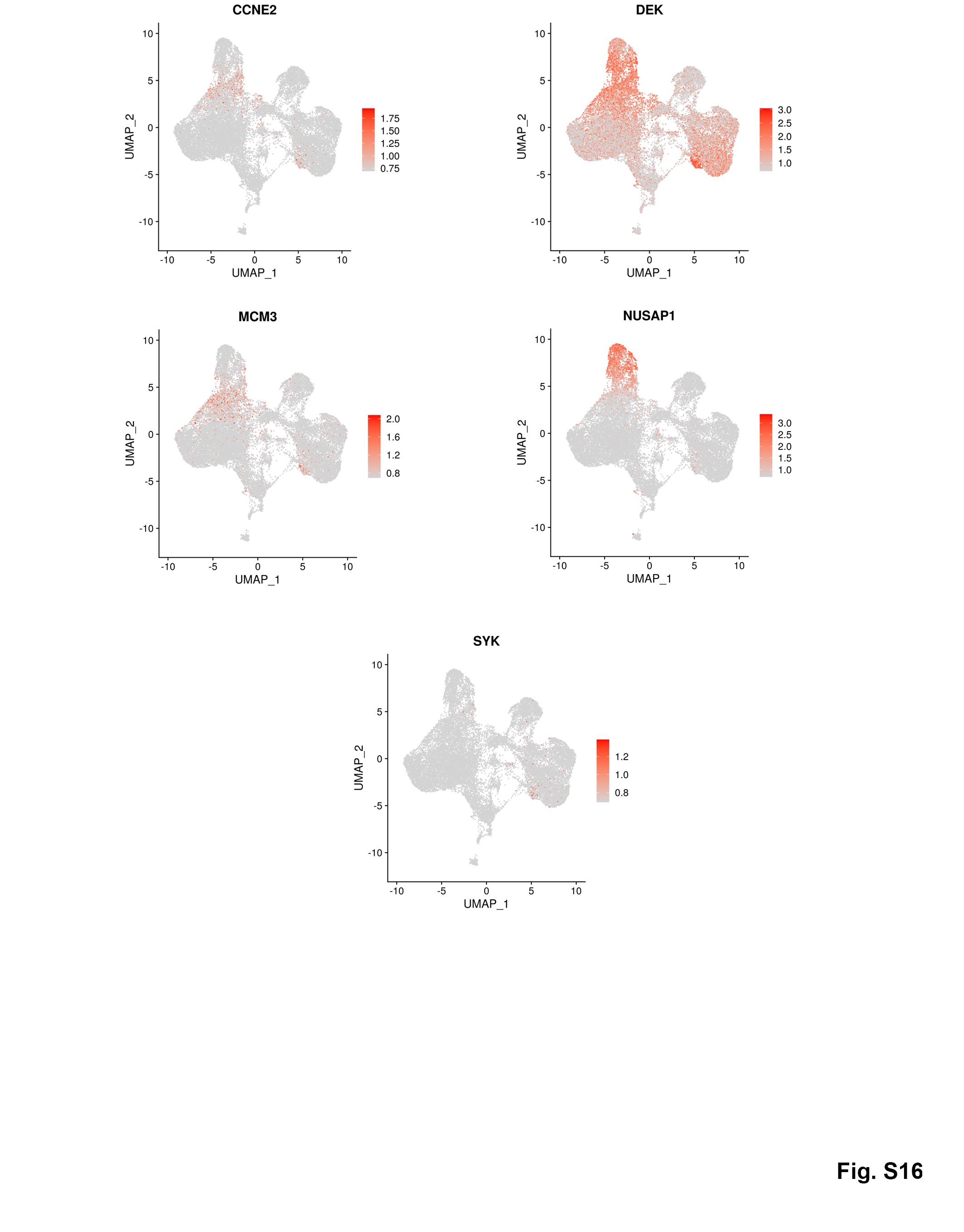

Supplement: szac008_suppl_Supplementary_Figure_S16 [file szac008_suppl_supplementary_figure_s16.jpeg]

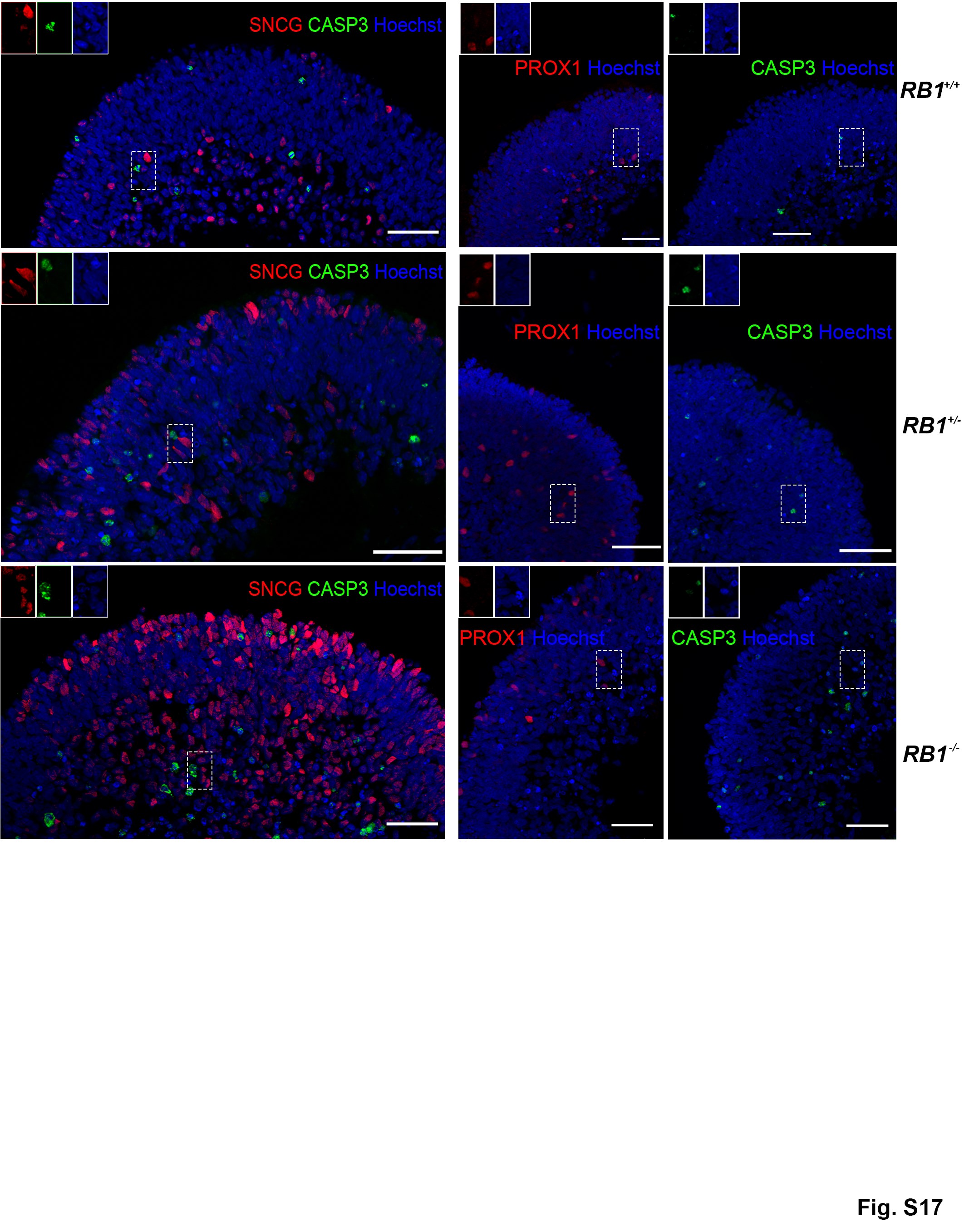

Supplement: szac008_suppl_Supplementary_Figure_S17 [file szac008_suppl_supplementary_figure_s17.jpeg]

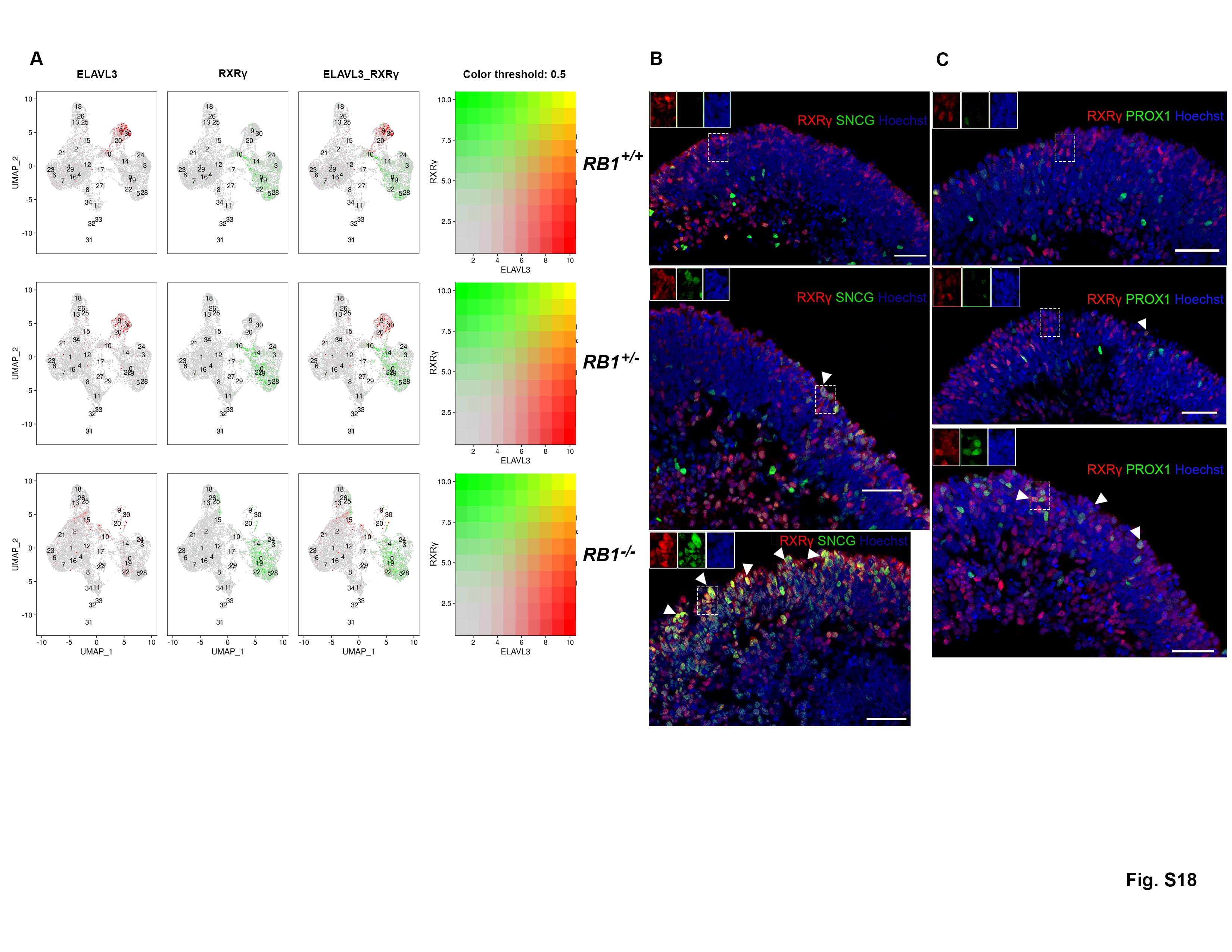

Supplement: szac008_suppl_Supplementary_Figure_S18 [file szac008_suppl_supplementary_figure_s18.jpeg]

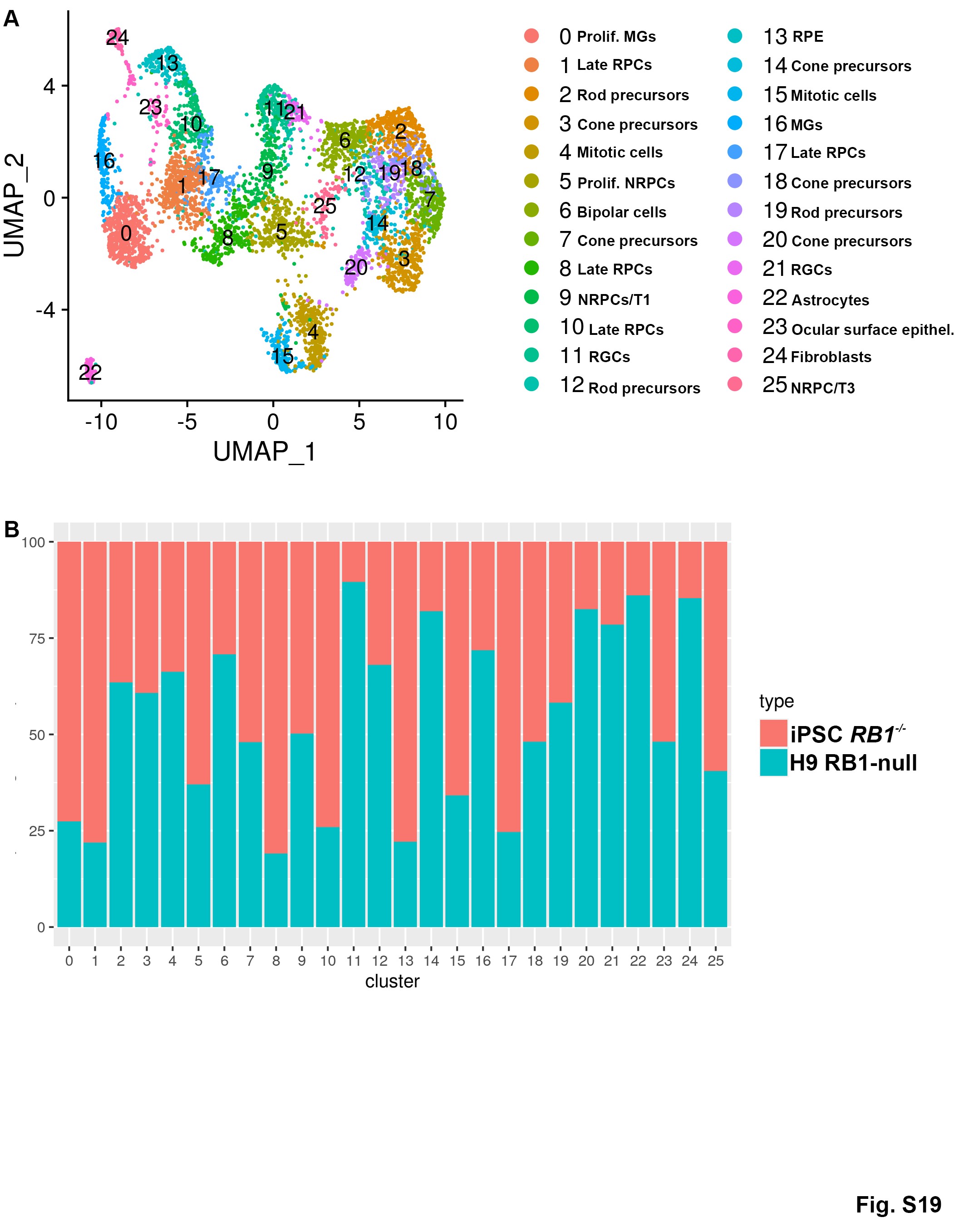

Supplement: szac008_suppl_Supplementary_Figure_S19 [file szac008_suppl_supplementary_figure_s19.jpeg]

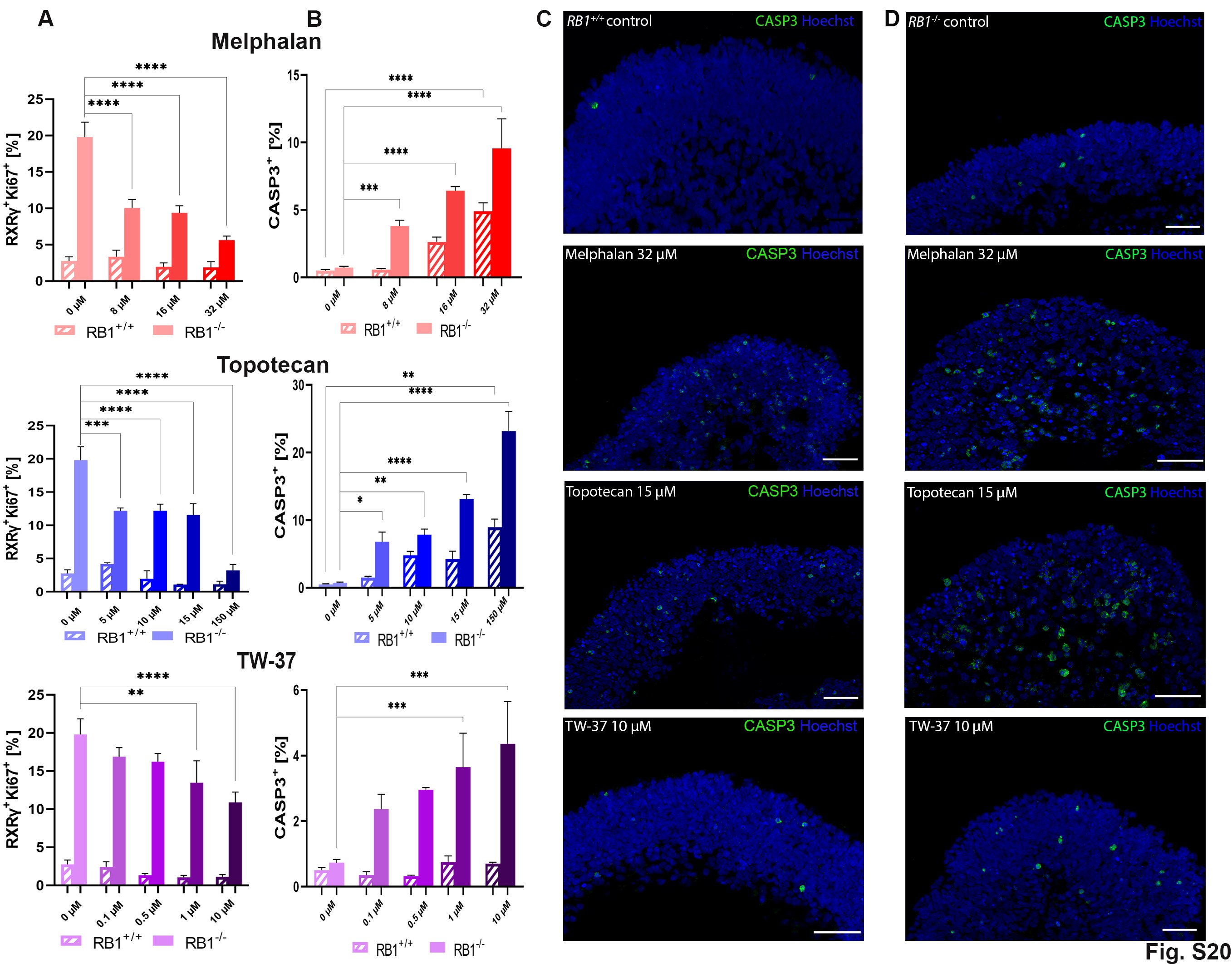

Supplement: szac008_suppl_Supplementary_Figure_S20 [file szac008_suppl_supplementary_figure_s20.jpeg]

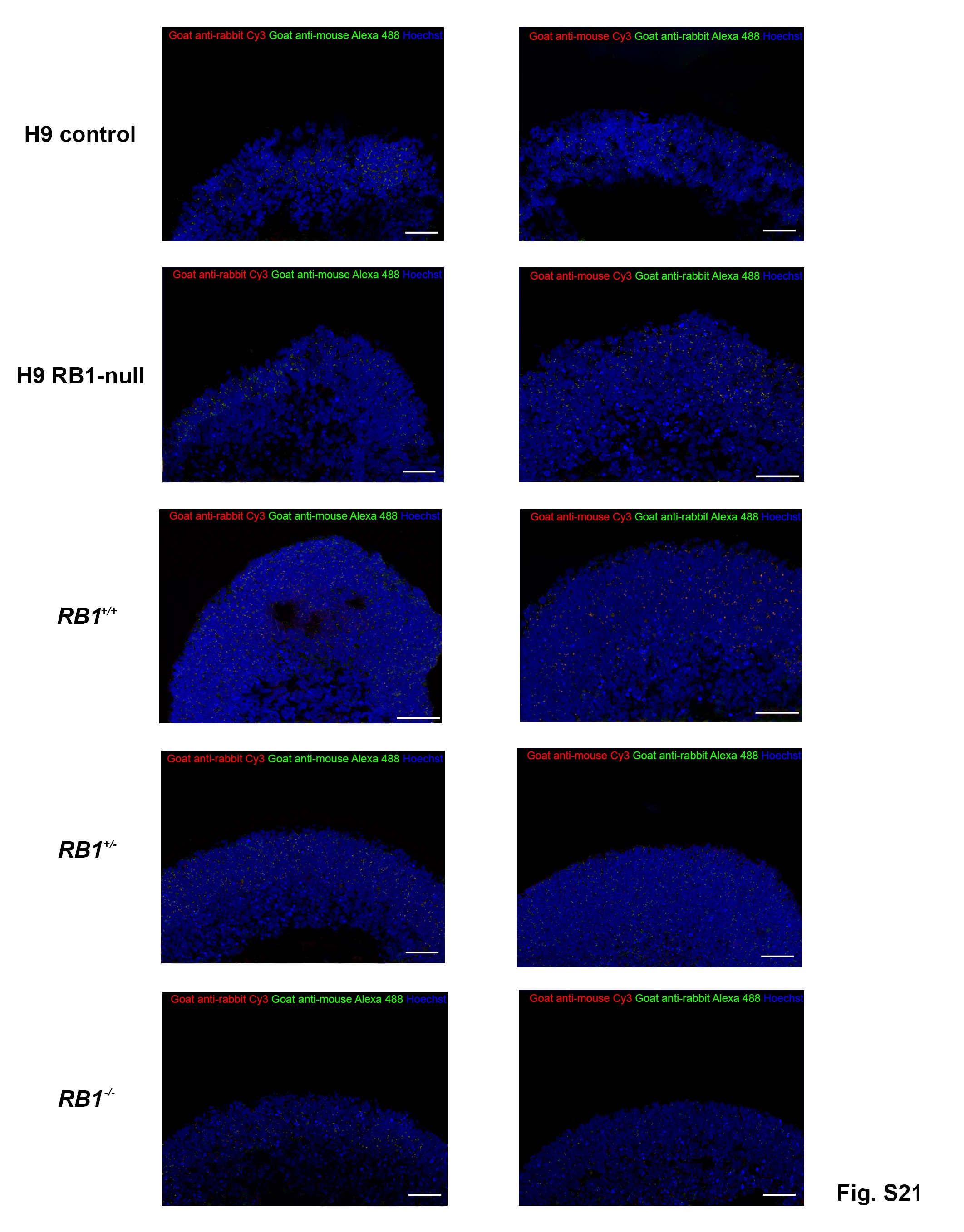

Supplement: szac008_suppl_Supplementary_Figure_S21 [file szac008_suppl_supplementary_figure_s21.jpeg]
