# Supplementary material for: pRB-Depleted Pluripotent Stem Cell Retinal Organoids Recapitulate Cell State Transitions of Retinoblastoma Development and Suggest an Important Role for pRB in Retinal Cell Differentiation
Source: Stem Cells Transl Med. 2022 Mar 23;11(4):415–33. doi: 10.1093/stcltm/szac008 (PMC9052432; doi:10.1093/stcltm/szac008)
Supplement: szac008_suppl_Supplementary_Table_S1 [file szac008_suppl_supplementary_table_s1.docx]

|  | p_val | avg_logFC | pct.1 | pct.2 | p_val_adj | cluster | gene | predicted cell fate |
| --- | --- | --- | --- | --- | --- | --- | --- | --- |
| SPP1 | 1.68E-285 | 1.356076 | 0.959 | 0.473 | 3.32E-281 | 0 | SPP1 | late RPCs |
| ZFP36L2 | 0 | 1.288866 | 0.913 | 0.271 | 0 | 0 | ZFP36L2 | late RPCs |
| FOS | 4.19E-213 | 1.214829 | 0.933 | 0.544 | 8.29E-209 | 0 | FOS | late RPCs |
| DKK3 | 3.83E-257 | 1.139683 | 0.94 | 0.422 | 7.59E-253 | 0 | DKK3 | late RPCs |
| ZFP36L1 | 0 | 1.109308 | 0.935 | 0.247 | 0 | 0 | ZFP36L1 | late RPCs |
| DAPL1 | 2.78E-191 | 1.03425 | 0.773 | 0.27 | 5.50E-187 | 0 | DAPL1 | late RPCs |
| TTYH1 | 4.54E-279 | 1.023004 | 0.922 | 0.304 | 8.99E-275 | 0 | TTYH1 | late RPCs |
| PLEKHA1 | 4.35E-224 | 1.016977 | 0.888 | 0.426 | 8.61E-220 | 0 | PLEKHA1 | late RPCs |
| SLC16A1 | 4.12E-212 | 1.00396 | 0.83 | 0.333 | 8.15E-208 | 0 | SLC16A1 | late RPCs |
| VIM | 8.32E-214 | 0.987657 | 0.999 | 0.723 | 1.65E-209 | 0 | VIM | late RPCs |
| EGR1 | 1.59E-194 | 0.985207 | 0.895 | 0.436 | 3.15E-190 | 0 | EGR1 | late RPCs |
| GPM6B | 1.27E-213 | 0.978544 | 0.948 | 0.697 | 2.51E-209 | 0 | GPM6B | late RPCs |
| PRSS23 | 2.14E-226 | 0.958241 | 0.844 | 0.298 | 4.23E-222 | 0 | PRSS23 | late RPCs |
| CLU | 3.95E-224 | 0.934138 | 0.98 | 0.571 | 7.83E-220 | 0 | CLU | late RPCs |
| HES5 | 1.34E-158 | 0.916295 | 0.472 | 0.102 | 2.65E-154 | 0 | HES5 | late RPCs |
| RTN4 | 1.09E-184 | 0.915292 | 0.986 | 0.951 | 2.15E-180 | 0 | RTN4 | late RPCs |
| QDPR | 1.45E-149 | 0.862186 | 0.637 | 0.229 | 2.86E-145 | 0 | QDPR | late RPCs |
| DIO3 | 9.29E-189 | 0.848098 | 0.633 | 0.158 | 1.84E-184 | 0 | DIO3 | late RPCs |
| JUNB | 3.11E-144 | 0.812787 | 0.87 | 0.518 | 6.15E-140 | 0 | JUNB | late RPCs |
| JUN | 4.04E-87 | 0.812147 | 0.885 | 0.721 | 8.00E-83 | 0 | JUN | late RPCs |
| CCND1 | 1.23E-159 | 0.795481 | 0.752 | 0.264 | 2.44E-155 | 0 | CCND1 | late RPCs |
| SOX2 | 7.96E-219 | 0.772416 | 0.867 | 0.277 | 1.58E-214 | 0 | SOX2 | late RPCs |
| SFRP2 | 2.76E-211 | 0.717854 | 0.949 | 0.439 | 5.47E-207 | 0 | SFRP2 | late RPCs |
| MGARP | 1.42E-241 | 0.715579 | 0.721 | 0.179 | 2.82E-237 | 0 | MGARP | late RPCs |
| ID4 | 1.12E-157 | 0.711882 | 0.73 | 0.273 | 2.22E-153 | 0 | ID4 | late RPCs |
| MDK | 1.66E-108 | 0.707801 | 0.75 | 0.354 | 3.28E-104 | 0 | MDK | late RPCs |
| PTPRZ1 | 3.55E-223 | 0.704154 | 0.728 | 0.198 | 7.03E-219 | 0 | PTPRZ1 | late RPCs |
| HES1 | 2.41E-161 | 0.702311 | 0.722 | 0.226 | 4.78E-157 | 0 | HES1 | late RPCs |
| HMX1 | 4.29E-160 | 0.675098 | 0.852 | 0.391 | 8.50E-156 | 0 | HMX1 | late RPCs |
| PLP1 | 5.73E-210 | 0.6661 | 0.516 | 0.091 | 1.13E-205 | 0 | PLP1 | late RPCs |
| DCBLD2 | 4.15E-158 | 0.655718 | 0.715 | 0.263 | 8.22E-154 | 0 | DCBLD2 | late RPCs |
| SLC2A1 | 2.79E-135 | 0.646596 | 0.769 | 0.33 | 5.52E-131 | 0 | SLC2A1 | late RPCs |
| IER2 | 1.05E-107 | 0.643134 | 0.915 | 0.725 | 2.07E-103 | 0 | IER2 | late RPCs |
| MIR503HG | 1.12E-214 | 0.642143 | 0.411 | 0.052 | 2.21E-210 | 0 | MIR503HG | late RPCs |
| LGALS3 | 6.33E-23 | 0.628187 | 0.19 | 0.08 | 1.25E-18 | 0 | LGALS3 | late RPCs |
| CKB | 2.09E-105 | 0.618341 | 0.974 | 0.981 | 4.15E-101 | 0 | CKB | late RPCs |
| RPL7 | 1.17E-126 | 0.614279 | 1 | 0.981 | 2.32E-122 | 0 | RPL7 | late RPCs |
| IFITM3 | 8.42E-145 | 0.605429 | 0.552 | 0.147 | 1.67E-140 | 0 | IFITM3 | late RPCs |
| PRSS35 | 2.91E-253 | 0.591755 | 0.536 | 0.08 | 5.76E-249 | 0 | PRSS35 | late RPCs |
| HES4 | 1.39E-81 | 0.586877 | 0.827 | 0.602 | 2.75E-77 | 0 | HES4 | late RPCs |
| RDH10 | 2.29E-143 | 0.562781 | 0.498 | 0.128 | 4.53E-139 | 0 | RDH10 | late RPCs |
| ENO1 | 1.22E-109 | 0.561521 | 0.993 | 0.968 | 2.43E-105 | 0 | ENO1 | late RPCs |
| CRB1 | 1.95E-157 | 0.55557 | 0.652 | 0.209 | 3.85E-153 | 0 | CRB1 | late RPCs |
| RAX | 4.49E-99 | 0.538408 | 0.879 | 0.573 | 8.89E-95 | 0 | RAX | late RPCs |
| IFITM2 | 3.49E-163 | 0.525918 | 0.445 | 0.085 | 6.91E-159 | 0 | IFITM2 | late RPCs |
| TF | 1.50E-74 | 0.520232 | 0.38 | 0.119 | 2.97E-70 | 0 | TF | late RPCs |
| RHOB | 2.21E-62 | 0.513771 | 0.647 | 0.369 | 4.37E-58 | 0 | RHOB | late RPCs |
| HIST1H4C | 1.87E-199 | 2.341647 | 0.822 | 0.318 | 3.71E-195 | 1 | HIST1H4C | Retinoblastoma-like |
| TFF1 | 0 | 1.815663 | 0.751 | 0.003 | 0 | 1 | TFF1 | Retinoblastoma-like |
| DEK | 6.03E-193 | 1.351683 | 0.969 | 0.667 | 1.19E-188 | 1 | DEK | Retinoblastoma-like |
| TUBB | 3.13E-179 | 1.30479 | 0.985 | 0.953 | 6.20E-175 | 1 | TUBB | Retinoblastoma-like |
| HMGA1 | 2.07E-169 | 1.112093 | 0.927 | 0.479 | 4.09E-165 | 1 | HMGA1 | Retinoblastoma-like |
| DCT | 1.02E-250 | 1.088926 | 0.806 | 0.169 | 2.02E-246 | 1 | DCT | Retinoblastoma-like |
| HSPA1A | 7.29E-41 | 0.952649 | 0.427 | 0.209 | 1.44E-36 | 1 | HSPA1A | Retinoblastoma-like |
| CENPF | 2.34E-147 | 0.899035 | 0.555 | 0.123 | 4.62E-143 | 1 | CENPF | Retinoblastoma-like |
| HLA-B | 7.68E-33 | 0.877108 | 0.33 | 0.15 | 1.52E-28 | 1 | HLA-B | Retinoblastoma-like |
| RPS18 | 1.03E-144 | 0.863837 | 0.998 | 0.999 | 2.04E-140 | 1 | RPS18 | Retinoblastoma-like |
| HMGB1 | 1.96E-124 | 0.839039 | 0.993 | 0.977 | 3.88E-120 | 1 | HMGB1 | Retinoblastoma-like |

| .36E-121 | 0.83815 | 0.894 | 0.496 | 2.70E-117 | 1 HMGB2 | Retinoblastoma-like |
| --- | --- | --- | --- | --- | --- | --- |
| .31E-153 | 0.827212 | 0.478 | 0.084 | 4.57E-149 | 1 UBE2C | Retinoblastoma-like |
| 1.14E-96 | 0.73525 | 0.566 | 0.206 | 2.26E-92 | 1 GMNN | Retinoblastoma-like |
| .92E-152 | 0.728809 | 1 | 0.999 | 7.77E-148 | 1 RPS7 | Retinoblastoma-like |
| .28E-156 | 0.719133 | 0.969 | 0.391 | 8.48E-152 | 1 RCVRN | Retinoblastoma-like |
| .05E-120 | 0.715446 | 0.996 | 0.978 | 2.09E-116 | 1 STMN1 | Retinoblastoma-like |
| 1.73E-64 | 0.678625 | 0.648 | 0.305 | 3.43E-60 | 1 PDE6H | Retinoblastoma-like |
| .67E-119 | 0.655591 | 1 | 0.998 | 9.24E-115 | 1 RPL21 | Retinoblastoma-like |
| 2.44E-54 | 0.649947 | 0.54 | 0.3 | 4.83E-50 | 1 FBL | Retinoblastoma-like |
| 1.12E-68 | 0.645326 | 0.784 | 0.543 | 2.22E-64 | 1 CUTA | Retinoblastoma-like |
| 1.14E-87 | 0.643249 | 0.991 | 0.897 | 2.25E-83 | 1 TUBA1B | Retinoblastoma-like |
| 2.69E-64 | 0.616544 | 0.531 | 0.23 | 5.33E-60 | 1 MCM3 | Retinoblastoma-like |
| .35E-132 | 0.614907 | 0.403 | 0.07 | 2.67E-128 | 1 HLA-DQB1 | Retinoblastoma-like |
| 0 | 0.59213 | 0.341 | 0.007 | 0 | 1 RPS4Y1 | Retinoblastoma-like |
| 7.91E-59 | 0.586632 | 0.454 | 0.187 | 1.57E-54 | 1 CKAP2 | Retinoblastoma-like |
| 3.95E-45 | 0.576245 | 0.608 | 0.404 | 7.81E-41 | 1 ANP32E | Retinoblastoma-like |
| 0.000589 | 0.574358 | 0.361 | 0.359 | 1 | 1 HLA-C | Retinoblastoma-like |
| 2.32E-10 | 0.572245 | 0.427 | 0.367 | 4.58E-06 | 1 HLA-A | Retinoblastoma-like |
| .42E-106 | 0.568123 | 0.496 | 0.124 | 1.07E-101 | 1 TPH1 | Retinoblastoma-like |
| .46E-102 | 0.563206 | 0.709 | 0.234 | 1.87E-97 | 1 AIPL1 | Retinoblastoma-like |
| 1.93E-69 | 0.559925 | 0.896 | 0.519 | 3.81E-65 | 1 CHCHD2 | Retinoblastoma-like |
| 7.84E-58 | 0.559319 | 0.811 | 0.626 | 1.55E-53 | 1 ILF2 | Retinoblastoma-like |
| .57E-105 | 0.55817 | 1 | 0.999 | 3.10E-101 | 1 RPS27A | Retinoblastoma-like |
| 2.90E-63 | 0.547675 | 0.938 | 0.798 | 5.74E-59 | 1 RPS10 | Retinoblastoma-like |
| 0 | 0.544134 | 0.315 | 0.001 | 0 | 1 TPRX1 | Retinoblastoma-like |
| 1.11E-87 | 0.537681 | 0.454 | 0.124 | 2.19E-83 | 1 TOP2A | Retinoblastoma-like |
| 2.74E-48 | 0.523074 | 0.676 | 0.488 | 5.42E-44 | 1 PPP1CC | Retinoblastoma-like |
| 9.16E-64 | 0.519501 | 0.548 | 0.222 | 1.81E-59 | 1 TULP1 | Retinoblastoma-like |
| 1.27E-54 | 0.519351 | 0.949 | 0.758 | 2.52E-50 | 1 NUCKS1 | Retinoblastoma-like |
| .35E-130 | 0.516556 | 0.352 | 0.052 | 1.06E-125 | 1 KIFC1 | Retinoblastoma-like |
| 2.88E-84 | 0.515588 | 1 | 0.952 | 5.70E-80 | 1 H2AFZ | Retinoblastoma-like |
| 1.85E-64 | 0.510742 | 0.903 | 0.653 | 3.67E-60 | 1 SLC25A6 | Retinoblastoma-like |
| 4.19E-61 | 0.510585 | 0.434 | 0.154 | 8.29E-57 | 1 SSTR2 | Retinoblastoma-like |
| .41E-237 | 2.142173 | 0.986 | 0.46 | 8.74E-233 | 2 SFRP2 | Muller Glia cells |
| .41E-237 | 1.945756 | 0.984 | 0.506 | 2.79E-233 | 2 TRH | Muller Glia cells |
| 0 | 1.660795 | 0.968 | 0.25 | 0 | 2 PTN | Muller Glia cells |
| 0 | 1.515613 | 0.832 | 0.126 | 0 | 2 WIF1 | Muller Glia cells |
| .88E-306 | 1.499155 | 0.975 | 0.241 | 5.70E-302 | 2 LAMP5 | Muller Glia cells |
| 4.08E-97 | 1.37994 | 0.474 | 0.124 | 8.08E-93 | 2 TF | Muller Glia cells |
| .86E-189 | 1.354264 | 1 | 0.736 | 5.66E-185 | 2 VIM | Muller Glia cells |
| 0 | 1.339819 | 0.925 | 0.165 | 0 | 2 CRYM | Muller Glia cells |
| .09E-224 | 1.336908 | 0.977 | 0.436 | 2.16E-220 | 2 METRN | Muller Glia cells |
| .63E-265 | 1.32172 | 0.934 | 0.223 | 3.23E-261 | 2 GPC3 | Muller Glia cells |
| .80E-299 | 1.242162 | 0.776 | 0.125 | 1.54E-294 | 2 NDP | Muller Glia cells |
| 0 | 1.123111 | 0.837 | 0.107 | 0 | 2 PAX2 | Muller Glia cells |
| .29E-271 | 1.08686 | 0.916 | 0.22 | 1.44E-266 | 2 COL18A1 | Muller Glia cells |
| .68E-191 | 0.988913 | 0.862 | 0.239 | 7.29E-187 | 2 HES1 | Muller Glia cells |
| .42E-189 | 0.981832 | 0.469 | 0.061 | 2.81E-185 | 2 GPX3 | Muller Glia cells |
| .78E-223 | 0.978638 | 0.81 | 0.189 | 9.47E-219 | 2 CYP1B1 | Muller Glia cells |
| .83E-136 | 0.95934 | 0.973 | 0.671 | 1.55E-131 | 2 FABP7 | Muller Glia cells |
| .26E-185 | 0.952929 | 0.959 | 0.33 | 2.50E-181 | 2 TTYH1 | Muller Glia cells |
| .84E-155 | 0.930474 | 0.968 | 0.444 | 5.63E-151 | 2 DKK3 | Muller Glia cells |
| 0 | 0.918185 | 0.776 | 0.114 | 0 | 2 ANGPTL1 | Muller Glia cells |
| .41E-172 | 0.90132 | 0.778 | 0.232 | 1.27E-167 | 2 AGL | Muller Glia cells |
| .92E-140 | 0.889463 | 0.984 | 0.59 | 3.81E-136 | 2 CLU | Muller Glia cells |
| .64E-135 | 0.882295 | 0.971 | 0.495 | 3.25E-131 | 2 SPP1 | Muller Glia cells |
| .86E-178 | 0.864779 | 0.932 | 0.3 | 1.75E-173 | 2 SOX2 | Muller Glia cells |
| .63E-224 | 0.852631 | 0.655 | 0.111 | 9.17E-220 | 2 CDH6 | Muller Glia cells |
| .52E-220 | 0.835543 | 0.735 | 0.147 | 6.98E-216 | 2 GSN | Muller Glia cells |

| HMGB2 1 |
| --- |
| UBE2C 2 |
| GMNN |
| RPS7 3 |
| RCVRN 4 |
| STMN1 1 |
| PDE6H |
| RPL21 4 |
| FBL |
| CUTA |
| TUBA1B |
| MCM3 |
| HLA-DQB1 1 |
| RPS4Y1 |
| CKAP2 |
| ANP32E |
| HLA-C |
| HLA-A |
| TPH1 5 |
| AIPL1 9 |
| CHCHD2 |
| ILF2 |
| RPS27A 1 |
| RPS10 |
| TPRX1 |
| TOP2A |
| PPP1CC |
| TULP1 |
| NUCKS1 |
| KIFC1 5 |
| H2AFZ |
| SLC25A6 |
| SSTR2 |
| SFRP21 4 |
| TRH 1 |
| PTN |
| WIF1 |
| LAMP5 2 |
| TF1 |
| VIM1 2 |
| CRYM |
| METRN 1 |
| GPC3 1 |
| NDP 7 |
| PAX2 |
| COL18A1 7 |
| HES11 3 |
| GPX3 1 |
| CYP1B1 4 |
| FABP7 7 |
| TTYH11 1 |
| DKK31 2 |
| ANGPTL1 |
| AGL 6 |
| CLU1 1 |
| SPP11 1 |
| SOX21 8 |
| CDH6 4 |
| GSN 3 |

| .47E-102 | 0.82612 | 1 | 0.819 | 1.87E-97 | 2 IGFBP2 | Muller Glia cells |
| --- | --- | --- | --- | --- | --- | --- |
| .33E-134 | 0.812852 | 0.68 | 0.196 | 4.61E-130 | 2 AKAP12 | Muller Glia cells |
| .13E-155 | 0.809978 | 0.746 | 0.228 | 2.24E-151 | 2 KLHL8 | Muller Glia cells |
| .38E-140 | 0.808662 | 0.68 | 0.177 | 8.67E-136 | 2 DIO3 | Muller Glia cells |
| .62E-238 | 0.792282 | 0.741 | 0.139 | 5.19E-234 | 2 OAF | Muller Glia cells |
| 0 | 0.784539 | 0.712 | 0.081 | 0 | 2 PAX8 | Muller Glia cells |
| .99E-154 | 0.747468 | 0.837 | 0.286 | 7.89E-150 | 2 LINC01833 | Muller Glia cells |
| .05E-165 | 0.72785 | 0.612 | 0.131 | 4.06E-161 | 2 GAS1 | Muller Glia cells |
| .95E-193 | 0.723871 | 0.655 | 0.129 | 1.57E-188 | 2 COL2A1 | Muller Glia cells |
| .65E-156 | 0.71708 | 0.844 | 0.29 | 1.91E-151 | 2 RHOC | Muller Glia cells |
| .64E-201 | 0.707186 | 0.744 | 0.163 | 3.24E-197 | 2 TPBG | Muller Glia cells |
| .34E-147 | 0.701288 | 0.825 | 0.27 | 2.66E-143 | 2 DNER | Muller Glia cells |
| .33E-152 | 0.698245 | 0.671 | 0.168 | 1.06E-147 | 2 COL9A3 | Muller Glia cells |
| .06E-152 | 0.697106 | 0.778 | 0.216 | 1.40E-147 | 2 CD9 | Muller Glia cells |
| 2.38E-73 | 0.66383 | 0.991 | 0.76 | 4.70E-69 | 2 CRABP1 | Muller Glia cells |
| .46E-182 | 0.663444 | 0.685 | 0.142 | 8.84E-178 | 2 THY1 | Muller Glia cells |
| 5.80E-92 | 0.660578 | 0.955 | 0.708 | 1.15E-87 | 2 GPM6B | Muller Glia cells |
| .22E-110 | 0.657124 | 0.846 | 0.324 | 2.41E-106 | 2 PRSS23 | Muller Glia cells |
| .14E-148 | 0.640907 | 0.66 | 0.168 | 4.23E-144 | 2 CHPF | Muller Glia cells |
| .67E-209 | 0.624201 | 0.585 | 0.086 | 1.12E-204 | 2 SLC1A3 | Muller Glia cells |
| .36E-299 | 0.623897 | 0.449 | 0.026 | 1.06E-294 | 2 SLC15A3 | Muller Glia cells |
| 1.07E-74 | 0.621123 | 0.565 | 0.208 | 2.11E-70 | 2 SRPRB | Muller Glia cells |
| 6.33E-95 | 0.618077 | 0.873 | 0.536 | 1.25E-90 | 2 SEC11C | Muller Glia cells |
| .84E-208 | 0.612459 | 0.408 | 0.038 | 3.64E-204 | 2 PTX3 | Muller Glia cells |
| 4.93E-80 | 0.590814 | 0.803 | 0.424 | 9.75E-76 | 2 NLRP1 | Muller Glia cells |
| 1.41E-96 | 0.587906 | 0.406 | 0.09 | 2.80E-92 | 2 SFRP1 | Muller Glia cells |
| .60E-113 | 0.586576 | 0.769 | 0.285 | 1.11E-108 | 2 SELENOP | Muller Glia cells |
| .35E-107 | 0.584851 | 0.812 | 0.364 | 1.26E-102 | 2 SSR3 | Muller Glia cells |
| .76E-155 | 0.584301 | 0.712 | 0.181 | 9.42E-151 | 2 MOB3B | Muller Glia cells |
| 6.38E-66 | 0.582939 | 0.991 | 0.953 | 1.26E-61 | 2 RTN4 | Muller Glia cells |
| .16E-117 | 0.579057 | 0.696 | 0.232 | 6.25E-113 | 2 OBSL1 | Muller Glia cells |
| 2.68E-99 | 0.571417 | 0.848 | 0.384 | 5.31E-95 | 2 FXYD6 | Muller Glia cells |
| 1.61E-99 | 0.568284 | 0.787 | 0.284 | 3.19E-95 | 2 CCND1 | Muller Glia cells |
| .33E-127 | 0.567382 | 0.737 | 0.228 | 2.64E-123 | 2 PLA2G16 | Muller Glia cells |
| .48E-120 | 0.562761 | 0.637 | 0.179 | 2.93E-116 | 2 BAMBI | Muller Glia cells |
| .09E-128 | 0.562219 | 0.751 | 0.241 | 4.14E-124 | 2 LIMCH1 | Muller Glia cells |
| 4.31E-71 | 0.555223 | 0.794 | 0.418 | 8.52E-67 | 2 HMX1 | Muller Glia cells |
| 1.02E-90 | 0.55519 | 0.8 | 0.354 | 2.01E-86 | 2 ATP1B3 | Muller Glia cells |
| 1.93E-75 | 0.551945 | 0.746 | 0.36 | 3.81E-71 | 2 ZBTB20 | Muller Glia cells |
| 2.86E-51 | 0.550187 | 0.694 | 0.356 | 5.66E-47 | 2 SLC2A1 | Muller Glia cells |
| 1.09E-68 | 0.549803 | 0.9 | 0.586 | 2.16E-64 | 2 RAX | Muller Glia cells |
| .74E-146 | 0.545558 | 0.66 | 0.16 | 9.39E-142 | 2 CREB5 | Muller Glia cells |
| 9.91E-83 | 0.545138 | 0.859 | 0.449 | 1.96E-78 | 2 CNN3 | Muller Glia cells |
| 4.01E-54 | 0.544666 | 0.891 | 0.736 | 7.94E-50 | 2 TXNIP | Muller Glia cells |
| .27E-143 | 0.544607 | 0.644 | 0.157 | 2.51E-139 | 2 FZD5 | Muller Glia cells |
| .56E-100 | 0.540934 | 0.751 | 0.278 | 1.30E-95 | 2 LINC00461 | Muller Glia cells |
| 1.21E-82 | 0.535488 | 0.859 | 0.455 | 2.39E-78 | 2 PHYHIPL | Muller Glia cells |
| .22E-200 | 0.535226 | 0.569 | 0.087 | 1.03E-195 | 2 KIAA1217 | Muller Glia cells |
| .16E-239 | 0.529956 | 0.549 | 0.063 | 2.30E-235 | 2 FAM69C | Muller Glia cells |
| .15E-101 | 0.52673 | 0.821 | 0.289 | 1.02E-96 | 2 ZFP36L1 | Muller Glia cells |
| .41E-105 | 0.525694 | 0.49 | 0.114 | 1.66E-100 | 2 PLP1 | Muller Glia cells |
| 3.71E-39 | 0.524215 | 0.488 | 0.21 | 7.35E-35 | 2 CYP26A1 | Muller Glia cells |
| 3.77E-23 | 0.522885 | 0.732 | 0.603 | 7.46E-19 | 2 NNAT | Muller Glia cells |
| .16E-110 | 0.520676 | 0.776 | 0.283 | 1.62E-105 | 2 NPC2 | Muller Glia cells |
| 5.63E-83 | 0.52012 | 0.8 | 0.346 | 1.12E-78 | 2 MEST | Muller Glia cells |
| .70E-144 | 0.518552 | 0.601 | 0.135 | 1.13E-139 | 2 TGFB2 | Muller Glia cells |
| 1.80E-98 | 0.515816 | 0.639 | 0.21 | 3.57E-94 | 2 PKDCC | Muller Glia cells |
| .23E-112 | 0.509451 | 0.633 | 0.183 | 2.44E-108 | 2 SOX9 | Muller Glia cells |
| 9.74E-88 | 0.509057 | 0.757 | 0.314 | 1.93E-83 | 2 LGR4 | Muller Glia cells |

| IGFBP2 9 |
| --- |
| AKAP12 2 |
| KLHL8 1 |
| DIO31 4 |
| OAF 2 |
| PAX8 |
| LINC01833 3 |
| GAS1 2 |
| COL2A1 7 |
| RHOC 9 |
| TPBG 1 |
| DNER 1 |
| COL9A3 5 |
| CD9 7 |
| CRABP1 |
| THY1 4 |
| GPM6B1 |
| PRSS231 1 |
| CHPF 2 |
| SLC1A3 5 |
| SLC15A3 5 |
| SRPRB |
| SEC11C |
| PTX3 1 |
| NLRP1 |
| SFRP1 |
| SELENOP 5 |
| SSR3 6 |
| MOB3B 4 |
| RTN41 |
| OBSL1 3 |
| FXYD6 |
| CCND11 |
| PLA2G16 1 |
| BAMBI 1 |
| LIMCH1 2 |
| HMX11 |
| ATP1B3 |
| ZBTB20 |
| SLC2A11 |
| RAX1 |
| CREB5 4 |
| CNN3 |
| TXNIP |
| FZD5 1 |
| LINC00461 6 |
| PHYHIPL |
| KIAA1217 5 |
| FAM69C 1 |
| ZFP36L11 5 |
| PLP11 8 |
| CYP26A1 |
| NNAT |
| NPC2 8 |
| MEST |
| TGFB2 5 |
| PKDCC |
| SOX9 1 |
| LGR4 |

| BEX1 | 6.53E-51 | 0.503593 | 0.918 | 0.793 | 1.29E-46 | 2 | BEX1 | Muller Glia cells |
| --- | --- | --- | --- | --- | --- | --- | --- | --- |
| EFNB1 | 3.66E-172 | 0.503387 | 0.587 | 0.109 | 7.24E-168 | 2 | EFNB1 | Muller Glia cells |
| GNAI2 | 4.20E-75 | 0.50048 | 0.871 | 0.493 | 8.31E-71 | 2 | GNAI2 | Muller Glia cells |
| NRL | 0 | 2.67887 | 1 | 0.256 | 0 | 3 | NRL | Rod precursors |
| PDC | 6.64E-285 | 1.934466 | 0.998 | 0.373 | 1.31E-280 | 3 | PDC | Rod precursors |
| RCVRN1 | 2.30E-222 | 1.914779 | 0.957 | 0.393 | 4.55E-218 | 3 | RCVRN | Rod precursors |
| ROM1 | 0 | 1.799277 | 0.945 | 0.102 | 0 | 3 | ROM1 | Rod precursors |
| GNAT1 | 0 | 1.508533 | 0.634 | 0.022 | 0 | 3 | GNAT1 | Rod precursors |
| UNC119 | 3.51E-219 | 1.439264 | 0.984 | 0.471 | 6.94E-215 | 3 | UNC119 | Rod precursors |
| AIPL11 | 1.05E-227 | 1.278804 | 0.874 | 0.222 | 2.08E-223 | 3 | AIPL1 | Rod precursors |
| NR2E3 | 0 | 1.24379 | 0.787 | 0.047 | 0 | 3 | NR2E3 | Rod precursors |
| GNB3 | 2.85E-221 | 1.12904 | 0.979 | 0.33 | 5.64E-217 | 3 | GNB3 | Rod precursors |
| CRX | 2.35E-224 | 1.088392 | 0.913 | 0.26 | 4.66E-220 | 3 | CRX | Rod precursors |
| RP1 | 2.63E-258 | 0.993624 | 0.634 | 0.084 | 5.22E-254 | 3 | RP1 | Rod precursors |
| Sep-04 | 4.69E-208 | 0.98777 | 0.934 | 0.289 | 9.28E-204 | 3 | Sep-04 | Rod precursors |
| PIK3R1 | 6.18E-147 | 0.910971 | 0.856 | 0.356 | 1.22E-142 | 3 | PIK3R1 | Rod precursors |
| SLC38A5 | 1.34E-180 | 0.907884 | 0.792 | 0.208 | 2.64E-176 | 3 | SLC38A5 | Rod precursors |
| NEUROD1 | 7.02E-134 | 0.876534 | 0.86 | 0.338 | 1.39E-129 | 3 | NEUROD1 | Rod precursors |
| TUBB4B | 5.93E-128 | 0.859133 | 0.991 | 0.849 | 1.17E-123 | 3 | TUBB4B | Rod precursors |
| CPE | 6.43E-118 | 0.83364 | 0.959 | 0.653 | 1.27E-113 | 3 | CPE | Rod precursors |
| GNGT2 | 6.08E-214 | 0.812734 | 0.698 | 0.124 | 1.20E-209 | 3 | GNGT2 | Rod precursors |
| FAM57B | 3.42E-133 | 0.810539 | 0.888 | 0.361 | 6.77E-129 | 3 | FAM57B | Rod precursors |
| PCBP4 | 3.59E-131 | 0.807892 | 0.966 | 0.526 | 7.11E-127 | 3 | PCBP4 | Rod precursors |
| CABP5 | 0 | 0.806176 | 0.513 | 0.029 | 0 | 3 | CABP5 | Rod precursors |
| PTP4A3 | 6.38E-160 | 0.793008 | 0.826 | 0.271 | 1.26E-155 | 3 | PTP4A3 | Rod precursors |
| RRAD | 2.88E-90 | 0.790682 | 0.563 | 0.175 | 5.70E-86 | 3 | RRAD | Rod precursors |
| KCNV2 | 3.45E-166 | 0.781488 | 0.684 | 0.153 | 6.82E-162 | 3 | KCNV2 | Rod precursors |
| IMPG2 | 7.08E-223 | 0.769545 | 0.776 | 0.153 | 1.40E-218 | 3 | IMPG2 | Rod precursors |
| GNGT1 | 2.94E-207 | 0.746421 | 0.314 | 0.018 | 5.82E-203 | 3 | GNGT1 | Rod precursors |
| PTPN13 | 8.40E-153 | 0.725443 | 0.668 | 0.173 | 1.66E-148 | 3 | PTPN13 | Rod precursors |
| PSIP1 | 1.20E-133 | 0.723383 | 0.995 | 0.851 | 2.38E-129 | 3 | PSIP1 | Rod precursors |
| MYO9A | 6.75E-167 | 0.708705 | 0.67 | 0.166 | 1.34E-162 | 3 | MYO9A | Rod precursors |
| SCG3 | 7.96E-82 | 0.700522 | 0.883 | 0.488 | 1.58E-77 | 3 | SCG3 | Rod precursors |
| EPS8 | 6.77E-166 | 0.7002 | 0.703 | 0.184 | 1.34E-161 | 3 | EPS8 | Rod precursors |
| NEUROG1 | 1.56E-77 | 0.683696 | 0.54 | 0.181 | 3.10E-73 | 3 | NEUROG1 | Rod precursors |
| PKM | 5.43E-100 | 0.670908 | 0.995 | 0.931 | 1.08E-95 | 3 | PKM | Rod precursors |
| ENO2 | 5.88E-95 | 0.670565 | 0.952 | 0.664 | 1.16E-90 | 3 | ENO2 | Rod precursors |
| PRDX1 | 1.80E-93 | 0.658279 | 0.982 | 0.867 | 3.57E-89 | 3 | PRDX1 | Rod precursors |
| RBP3 | 1.28E-183 | 0.654876 | 0.65 | 0.125 | 2.54E-179 | 3 | RBP3 | Rod precursors |
| MAP2 | 9.74E-75 | 0.653232 | 0.87 | 0.562 | 1.93E-70 | 3 | MAP2 | Rod precursors |
| ALDOC | 4.90E-117 | 0.650502 | 0.547 | 0.143 | 9.70E-113 | 3 | ALDOC | Rod precursors |
| GPR160 | 7.03E-202 | 0.641784 | 0.437 | 0.047 | 1.39E-197 | 3 | GPR160 | Rod precursors |
| TULP11 | 4.19E-145 | 0.639928 | 0.755 | 0.207 | 8.30E-141 | 3 | TULP1 | Rod precursors |
| EPB41L2 | 8.85E-127 | 0.632317 | 0.636 | 0.182 | 1.75E-122 | 3 | EPB41L2 | Rod precursors |
| AANAT | 1.88E-150 | 0.631914 | 0.675 | 0.167 | 3.72E-146 | 3 | AANAT | Rod precursors |
| AMER2 | 6.08E-73 | 0.631231 | 0.796 | 0.435 | 1.20E-68 | 3 | AMER2 | Rod precursors |
| AKAP9 | 1.05E-84 | 0.630629 | 0.915 | 0.592 | 2.08E-80 | 3 | AKAP9 | Rod precursors |
| MAP1LC3A | 4.41E-91 | 0.629161 | 0.801 | 0.37 | 8.74E-87 | 3 | MAP1LC3A | Rod precursors |
| OTX2 | 2.48E-111 | 0.627287 | 0.817 | 0.292 | 4.90E-107 | 3 | OTX2 | Rod precursors |
| LINC00599 | 8.74E-92 | 0.623659 | 0.725 | 0.282 | 1.73E-87 | 3 | LINC00599 | Rod precursors |
| PDE6G | 5.58E-179 | 0.623501 | 0.348 | 0.031 | 1.10E-174 | 3 | PDE6G | Rod precursors |
| CPLX4 | 4.07E-143 | 0.622484 | 0.565 | 0.119 | 8.06E-139 | 3 | CPLX4 | Rod precursors |
| SYP | 1.31E-98 | 0.616491 | 0.799 | 0.33 | 2.60E-94 | 3 | SYP | Rod precursors |
| PDE4DIP | 1.41E-109 | 0.608742 | 0.586 | 0.173 | 2.80E-105 | 3 | PDE4DIP | Rod precursors |
| PLEKHB1 | 8.73E-106 | 0.605482 | 0.686 | 0.224 | 1.73E-101 | 3 | PLEKHB1 | Rod precursors |
| RHO | 1.63E-114 | 0.591227 | 0.126 | 0.003 | 3.22E-110 | 3 | RHO | Rod precursors |
| LAPTM4B | 8.36E-84 | 0.589132 | 0.902 | 0.631 | 1.66E-79 | 3 | LAPTM4B | Rod precursors |
| REEP6 | 1.99E-189 | 0.584941 | 0.57 | 0.095 | 3.95E-185 | 3 | REEP6 | Rod precursors |
| SLC1A7 | 2.16E-158 | 0.581581 | 0.645 | 0.145 | 4.28E-154 | 3 | SLC1A7 | Rod precursors |

| PRNP | 1.87E-96 | 0.575509 | 0.783 | 0.346 | 3.70E-92 | 3 | PRNP | Rod precursors |
| --- | --- | --- | --- | --- | --- | --- | --- | --- |
| TUBA4A | 7.94E-101 | 0.573575 | 0.7 | 0.239 | 1.57E-96 | 3 | TUBA4A | Rod precursors |
| EYS | 1.13E-187 | 0.571772 | 0.602 | 0.103 | 2.24E-183 | 3 | EYS | Rod precursors |
| EPB41 | 4.71E-66 | 0.570968 | 0.73 | 0.377 | 9.32E-62 | 3 | EPB41 | Rod precursors |
| MAK | 9.80E-159 | 0.565626 | 0.581 | 0.115 | 1.94E-154 | 3 | MAK | Rod precursors |
| ATP1B2 | 2.11E-81 | 0.564068 | 0.773 | 0.384 | 4.18E-77 | 3 | ATP1B2 | Rod precursors |
| ATP1A3 | 4.90E-70 | 0.563794 | 0.703 | 0.322 | 9.70E-66 | 3 | ATP1A3 | Rod precursors |
| STX3 | 5.50E-125 | 0.559526 | 0.7 | 0.21 | 1.09E-120 | 3 | STX3 | Rod precursors |
| PRPH2 | 3.70E-224 | 0.551473 | 0.478 | 0.05 | 7.32E-220 | 3 | PRPH2 | Rod precursors |
| NRN1 | 2.35E-63 | 0.549097 | 0.593 | 0.232 | 4.65E-59 | 3 | NRN1 | Rod precursors |
| NFIB | 3.41E-37 | 0.545013 | 0.59 | 0.349 | 6.76E-33 | 3 | NFIB | Rod precursors |
| FSTL5 | 4.70E-91 | 0.540799 | 0.661 | 0.225 | 9.30E-87 | 3 | FSTL5 | Rod precursors |
| NEUROD4 | 2.66E-80 | 0.540417 | 0.652 | 0.24 | 5.27E-76 | 3 | NEUROD4 | Rod precursors |
| CRABP2 | 5.11E-47 | 0.538576 | 0.684 | 0.366 | 1.01E-42 | 3 | CRABP2 | Rod precursors |
| GUK1 | 9.11E-88 | 0.537706 | 0.963 | 0.778 | 1.80E-83 | 3 | GUK1 | Rod precursors |
| HMGN1 | 2.47E-49 | 0.535741 | 0.984 | 0.932 | 4.88E-45 | 3 | HMGN1 | Rod precursors |
| MT-ATP6 | 1.23E-48 | 0.531714 | 0.998 | 0.995 | 2.43E-44 | 3 | MT-ATP6 | Rod precursors |
| MPP4 | 2.39E-123 | 0.527753 | 0.574 | 0.13 | 4.74E-119 | 3 | MPP4 | Rod precursors |
| MAP1LC3B | 4.69E-66 | 0.524351 | 0.794 | 0.481 | 9.29E-62 | 3 | MAP1LC3B | Rod precursors |
| FAM161A | 9.92E-72 | 0.501967 | 0.54 | 0.2 | 1.96E-67 | 3 | FAM161A | Rod precursors |
| ZNF385A | 8.22E-77 | 0.500664 | 0.751 | 0.347 | 1.63E-72 | 3 | ZNF385A | Rod precursors |
| HES6 | 2.15E-160 | 2.122182 | 0.954 | 0.549 | 4.27E-156 | 4 | HES6 | NRPCs |
| GADD45A | 2.47E-131 | 1.637143 | 0.829 | 0.364 | 4.89E-127 | 4 | GADD45A | NRPCs |
| ATOH7 | 2.07E-64 | 1.436426 | 0.254 | 0.045 | 4.10E-60 | 4 | ATOH7 | NRPCs |
| CCND12 | 6.35E-63 | 1.275655 | 0.649 | 0.301 | 1.26E-58 | 4 | CCND1 | NRPCs |
| RGS16 | 1.06E-92 | 1.111437 | 0.891 | 0.529 | 2.09E-88 | 4 | RGS16 | NRPCs |
| CKB1 | 1.05E-107 | 1.077483 | 1 | 0.979 | 2.09E-103 | 4 | CKB | NRPCs |
| MIAT | 1.24E-102 | 0.944345 | 0.811 | 0.337 | 2.45E-98 | 4 | MIAT | NRPCs |
| GADD45G | 4.90E-74 | 0.899282 | 0.814 | 0.42 | 9.70E-70 | 4 | GADD45G | NRPCs |
| CXCR4 | 1.36E-107 | 0.861413 | 0.726 | 0.242 | 2.69E-103 | 4 | CXCR4 | NRPCs |
| PMAIP1 | 2.24E-99 | 0.842457 | 0.617 | 0.184 | 4.43E-95 | 4 | PMAIP1 | NRPCs |
| CRABP11 | 3.72E-56 | 0.819445 | 0.966 | 0.766 | 7.36E-52 | 4 | CRABP1 | NRPCs |
| PFN2 | 5.49E-78 | 0.780644 | 0.777 | 0.404 | 1.09E-73 | 4 | PFN2 | NRPCs |
| HES51 | 3.31E-25 | 0.745633 | 0.326 | 0.134 | 6.55E-21 | 4 | HES5 | NRPCs |
| HMGB21 | 2.17E-15 | 0.729597 | 0.643 | 0.519 | 4.30E-11 | 4 | HMGB2 | NRPCs |
| DOK5 | 1.13E-96 | 0.703927 | 0.549 | 0.146 | 2.23E-92 | 4 | DOK5 | NRPCs |
| RBP1 | 1.16E-69 | 0.702943 | 0.991 | 0.967 | 2.30E-65 | 4 | RBP1 | NRPCs |
| BTG2 | 5.28E-67 | 0.702142 | 0.711 | 0.324 | 1.05E-62 | 4 | BTG2 | NRPCs |
| MDK1 | 1.05E-35 | 0.700798 | 0.657 | 0.385 | 2.08E-31 | 4 | MDK | NRPCs |
| RORB | 2.48E-78 | 0.694201 | 0.897 | 0.499 | 4.90E-74 | 4 | RORB | NRPCs |
| SHD | 5.34E-83 | 0.692184 | 0.583 | 0.185 | 1.06E-78 | 4 | SHD | NRPCs |
| TCF4 | 2.39E-92 | 0.691208 | 0.854 | 0.391 | 4.73E-88 | 4 | TCF4 | NRPCs |
| PLEKHA11 | 2.72E-62 | 0.691205 | 0.817 | 0.459 | 5.38E-58 | 4 | PLEKHA1 | NRPCs |
| RASD1 | 6.44E-73 | 0.672494 | 0.543 | 0.172 | 1.28E-68 | 4 | RASD1 | NRPCs |
| CLDN5 | 2.66E-33 | 0.624633 | 0.263 | 0.081 | 5.27E-29 | 4 | CLDN5 | NRPCs |
| DAPL11 | 1.99E-46 | 0.615719 | 0.649 | 0.309 | 3.95E-42 | 4 | DAPL1 | NRPCs |
| E2F1 | 4.61E-83 | 0.615226 | 0.629 | 0.205 | 9.13E-79 | 4 | E2F1 | NRPCs |
| HMGN2 | 1.15E-15 | 0.614258 | 0.923 | 0.894 | 2.27E-11 | 4 | HMGN2 | NRPCs |
| SH3BGRL3 | 1.24E-56 | 0.607715 | 0.777 | 0.421 | 2.45E-52 | 4 | SH3BGRL3 | NRPCs |
| SOX11 | 6.83E-49 | 0.604182 | 0.877 | 0.619 | 1.35E-44 | 4 | SOX11 | NRPCs |
| TRH1 | 1.39E-43 | 0.59111 | 0.823 | 0.523 | 2.75E-39 | 4 | TRH | NRPCs |
| TFDP2 | 3.00E-82 | 0.586297 | 0.754 | 0.318 | 5.95E-78 | 4 | TFDP2 | NRPCs |
| FAM131C | 5.76E-148 | 0.580887 | 0.391 | 0.045 | 1.14E-143 | 4 | FAM131C | NRPCs |
| VSTM2B | 2.08E-86 | 0.573553 | 0.503 | 0.127 | 4.12E-82 | 4 | VSTM2B | NRPCs |
| TOP2A1 | 3.93E-16 | 0.569957 | 0.297 | 0.14 | 7.79E-12 | 4 | TOP2A | NRPCs |
| TYMS | 6.18E-17 | 0.564793 | 0.497 | 0.325 | 1.22E-12 | 4 | TYMS | NRPCs |
| FBLN1 | 7.51E-48 | 0.56338 | 0.614 | 0.288 | 1.49E-43 | 4 | FBLN1 | NRPCs |
| PTMA | 1.53E-64 | 0.548878 | 1 | 0.999 | 3.03E-60 | 4 | PTMA | NRPCs |
| UBE2S | 5.58E-06 | 0.540202 | 0.714 | 0.642 | 0.110468 | 4 | UBE2S | NRPCs |

| LCA5 | 6.54E-49 | 0.52453 | 0.609 | 0.28 | 1.29E-44 | 4 | LCA5 | NRPCs |
| --- | --- | --- | --- | --- | --- | --- | --- | --- |
| ASCL1 | 1.01E-42 | 0.511316 | 0.406 | 0.141 | 2.00E-38 | 4 | ASCL1 | NRPCs |
| RBPJ | 1.39E-51 | 0.506047 | 0.826 | 0.497 | 2.76E-47 | 4 | RBPJ | NRPCs |
| MFAP4 | 2.73E-69 | 0.502997 | 0.397 | 0.096 | 5.40E-65 | 4 | MFAP4 | NRPCs |
| AMER21 | 2.12E-168 | 1.146814 | 0.977 | 0.429 | 4.20E-164 | 5 | AMER2 | NRPCs/T3 |
| OTX21 | 3.14E-181 | 1.049532 | 0.937 | 0.293 | 6.22E-177 | 5 | OTX2 | NRPCs/T3 |
| NEUROG11 | 1.03E-181 | 0.969284 | 0.784 | 0.172 | 2.05E-177 | 5 | NEUROG1 | NRPCs/T3 |
| NEUROD11 | 1.64E-139 | 0.956292 | 0.937 | 0.341 | 3.25E-135 | 5 | NEUROD1 | NRPCs/T3 |
| NEUROD41 | 9.40E-163 | 0.942878 | 0.848 | 0.235 | 1.86E-158 | 5 | NEUROD4 | NRPCs/T3 |
| SLC38A51 | 1.36E-150 | 0.916698 | 0.813 | 0.215 | 2.70E-146 | 5 | SLC38A5 | NRPCs/T3 |
| CHODL | 1.44E-105 | 0.9018 | 0.532 | 0.121 | 2.86E-101 | 5 | CHODL | NRPCs/T3 |
| VSX1 | 1.25E-75 | 0.795866 | 0.486 | 0.13 | 2.47E-71 | 5 | VSX1 | NRPCs/T3 |
| RXRG | 3.01E-100 | 0.793286 | 0.713 | 0.227 | 5.97E-96 | 5 | RXRG | NRPCs/T3 |
| TPH11 | 6.10E-67 | 0.764129 | 0.468 | 0.132 | 1.21E-62 | 5 | TPH1 | NRPCs/T3 |
| Sep-41 | 4.44E-119 | 0.759954 | 0.891 | 0.302 | 8.80E-115 | 5 | Sep-04 | NRPCs/T3 |
| C11orf96 | 8.91E-124 | 0.750503 | 0.649 | 0.156 | 1.76E-119 | 5 | C11orf96 | NRPCs/T3 |
| RRAD1 | 5.53E-40 | 0.732991 | 0.477 | 0.186 | 1.10E-35 | 5 | RRAD | NRPCs/T3 |
| DCT1 | 3.07E-63 | 0.730396 | 0.572 | 0.195 | 6.09E-59 | 5 | DCT | NRPCs/T3 |
| PDC1 | 2.85E-76 | 0.728684 | 0.845 | 0.392 | 5.65E-72 | 5 | PDC | NRPCs/T3 |
| SCG31 | 4.21E-76 | 0.726533 | 0.922 | 0.492 | 8.34E-72 | 5 | SCG3 | NRPCs/T3 |
| VXN | 3.93E-80 | 0.704732 | 0.56 | 0.161 | 7.79E-76 | 5 | VXN | NRPCs/T3 |
| GSG1 | 6.39E-110 | 0.691347 | 0.606 | 0.146 | 1.26E-105 | 5 | GSG1 | NRPCs/T3 |
| PCBP41 | 9.51E-97 | 0.688308 | 0.974 | 0.532 | 1.88E-92 | 5 | PCBP4 | NRPCs/T3 |
| NFIB1 | 2.04E-61 | 0.686176 | 0.718 | 0.345 | 4.04E-57 | 5 | NFIB | NRPCs/T3 |
| FABP71 | 6.65E-13 | 0.681649 | 0.764 | 0.689 | 1.32E-08 | 5 | FABP7 | NRPCs/T3 |
| CADM3 | 6.97E-100 | 0.680926 | 0.759 | 0.279 | 1.38E-95 | 5 | CADM3 | NRPCs/T3 |
| SPCS1 | 1.90E-97 | 0.674276 | 0.98 | 0.837 | 3.76E-93 | 5 | SPCS1 | NRPCs/T3 |
| GADD45G1 | 4.04E-70 | 0.663809 | 0.845 | 0.418 | 7.99E-66 | 5 | GADD45G | NRPCs/T3 |
| FAM57B1 | 1.58E-79 | 0.65841 | 0.851 | 0.371 | 3.14E-75 | 5 | FAM57B | NRPCs/T3 |
| LINC00599 | 2.26E-90 | 0.621577 | 0.784 | 0.286 | 4.48E-86 | 5 | LINC00599 | NRPCs/T3 |
| SERPINF1 | 9.33E-112 | 0.617228 | 0.767 | 0.245 | 1.85E-107 | 5 | SERPINF1 | NRPCs/T3 |
| DLL3 | 1.31E-93 | 0.597393 | 0.615 | 0.184 | 2.59E-89 | 5 | DLL3 | NRPCs/T3 |
| NLK | 3.10E-124 | 0.592881 | 0.739 | 0.205 | 6.14E-120 | 5 | NLK | NRPCs/T3 |
| MIR7-3HG | 1.01E-56 | 0.585672 | 0.566 | 0.209 | 2.01E-52 | 5 | MIR7-3HG | NRPCs/T3 |
| CADPS | 1.55E-80 | 0.573536 | 0.767 | 0.296 | 3.06E-76 | 5 | CADPS | NRPCs/T3 |
| PTP4A31 | 2.55E-70 | 0.560264 | 0.73 | 0.286 | 5.05E-66 | 5 | PTP4A3 | NRPCs/T3 |
| PRDM1 | 7.67E-106 | 0.554508 | 0.382 | 0.061 | 1.52E-101 | 5 | PRDM1 | NRPCs/T3 |
| CRX1 | 7.17E-99 | 0.551746 | 0.856 | 0.274 | 1.42E-94 | 5 | CRX | NRPCs/T3 |
| GNB31 | 1.59E-87 | 0.548694 | 0.891 | 0.346 | 3.14E-83 | 5 | GNB3 | NRPCs/T3 |
| EPB411 | 4.46E-70 | 0.546547 | 0.81 | 0.378 | 8.83E-66 | 5 | EPB41 | NRPCs/T3 |
| PGRMC2 | 4.22E-63 | 0.540177 | 0.75 | 0.36 | 8.36E-59 | 5 | PGRMC2 | NRPCs/T3 |
| INSM1 | 1.58E-72 | 0.536909 | 0.759 | 0.304 | 3.13E-68 | 5 | INSM1 | NRPCs/T3 |
| MAP1LC3A | 1.17E-60 | 0.536881 | 0.784 | 0.378 | 2.31E-56 | 5 | MAP1LC3A | NRPCs/T3 |
| IGSF21 | 4.16E-69 | 0.530529 | 0.661 | 0.236 | 8.24E-65 | 5 | IGSF21 | NRPCs/T3 |
| SYP1 | 7.29E-74 | 0.523759 | 0.813 | 0.337 | 1.44E-69 | 5 | SYP | NRPCs/T3 |
| ATOH71 | 3.65E-25 | 0.512493 | 0.181 | 0.049 | 7.22E-21 | 5 | ATOH7 | NRPCs/T3 |
| NRL1 | 6.19E-102 | 0.505258 | 0.816 | 0.279 | 1.23E-97 | 5 | NRL | NRPCs/T3 |
| TMEM70 | 5.03E-24 | 0.503348 | 0.506 | 0.271 | 9.96E-20 | 5 | TMEM70 | NRPCs/T3 |
| TMX1 | 5.89E-72 | 0.501189 | 0.784 | 0.354 | 1.17E-67 | 5 | TMX1 | NRPCs/T3 |
| CYP26A11 | 9.68E-286 | 2.360876 | 0.923 | 0.189 | 1.92E-281 | 6 | CYP26A1 | Muller Glia cells |
| GPC31 | 2.94E-280 | 1.930078 | 0.979 | 0.234 | 5.82E-276 | 6 | GPC3 | Muller Glia cells |
| LAMP51 | 1.28E-184 | 1.405403 | 0.906 | 0.258 | 2.53E-180 | 6 | LAMP5 | Muller Glia cells |
| IGFBP7 | 2.53E-31 | 1.231669 | 0.301 | 0.106 | 5.00E-27 | 6 | IGFBP7 | Muller Glia cells |
| HOXB6 | 2.64E-260 | 1.109089 | 0.77 | 0.106 | 5.23E-256 | 6 | HOXB6 | Muller Glia cells |
| PPY | 1.06E-71 | 1.046275 | 0.192 | 0.021 | 2.09E-67 | 6 | PPY | Muller Glia cells |
| HOXB5 | 8.70E-192 | 1.035979 | 0.782 | 0.154 | 1.72E-187 | 6 | HOXB5 | Muller Glia cells |
| MEST1 | 1.46E-123 | 0.994603 | 0.885 | 0.349 | 2.88E-119 | 6 | MEST | Muller Glia cells |
| DHRS3 | 1.53E-126 | 0.938715 | 0.779 | 0.242 | 3.03E-122 | 6 | DHRS3 | Muller Glia cells |
| BCAM | 6.24E-54 | 0.883678 | 0.478 | 0.171 | 1.24E-49 | 6 | BCAM | Muller Glia cells |

| .32E-116 | 0.862036 | 0.853 | 0.318 | 8.56E-112 | 6 HOXB2 | Muller Glia cells |
| --- | --- | --- | --- | --- | --- | --- |
| .84E-101 | 0.859156 | 0.912 | 0.434 | 7.60E-97 | 6 SAT1 | Muller Glia cells |
| .51E-148 | 0.849806 | 0.673 | 0.143 | 1.09E-143 | 6 NDP | Muller Glia cells |
| .20E-136 | 0.823743 | 0.357 | 0.039 | 1.03E-131 | 6 MAL | Muller Glia cells |
| 3.19E-67 | 0.822564 | 0.897 | 0.571 | 6.31E-63 | 6 FOS | Muller Glia cells |
| .61E-106 | 0.816378 | 0.788 | 0.255 | 1.51E-101 | 6 HES1 | Muller Glia cells |
| .07E-162 | 0.792724 | 0.649 | 0.129 | 2.12E-158 | 6 PDZRN3 | Muller Glia cells |
| .62E-150 | 0.785131 | 0.844 | 0.237 | 1.71E-145 | 6 COL18A1 | Muller Glia cells |
| .90E-147 | 0.763831 | 0.69 | 0.16 | 5.74E-143 | 6 ATP2B4 | Muller Glia cells |
| .13E-176 | 0.753743 | 0.655 | 0.115 | 8.17E-172 | 6 HOXB4 | Muller Glia cells |
| .09E-158 | 0.748268 | 0.587 | 0.1 | 2.15E-154 | 6 PAX8 | Muller Glia cells |
| 7.48E-79 | 0.720659 | 0.628 | 0.211 | 1.48E-74 | 6 CYP1B1 | Muller Glia cells |
| 1.38E-70 | 0.700501 | 0.799 | 0.351 | 2.73E-66 | 6 TTYH1 | Muller Glia cells |
| 3.84E-94 | 0.69919 | 0.463 | 0.102 | 7.61E-90 | 6 SLC1A3 | Muller Glia cells |
| 1.60E-84 | 0.6991 | 0.298 | 0.045 | 3.17E-80 | 6 ALDH1A1 | Muller Glia cells |
| 7.38E-66 | 0.69676 | 0.85 | 0.468 | 1.46E-61 | 6 EGR1 | Muller Glia cells |
| .14E-141 | 0.68626 | 0.513 | 0.084 | 2.27E-137 | 6 CFI | Muller Glia cells |
| 6.35E-69 | 0.684952 | 0.587 | 0.192 | 1.26E-64 | 6 DIO3 | Muller Glia cells |
| 1.98E-97 | 0.683515 | 0.667 | 0.194 | 3.93E-93 | 6 CRYM | Muller Glia cells |
| .65E-102 | 0.670581 | 0.808 | 0.292 | 3.26E-98 | 6 SELENOP | Muller Glia cells |
| 2.98E-89 | 0.669943 | 0.914 | 0.45 | 5.90E-85 | 6 METRN | Muller Glia cells |
| 2.23E-59 | 0.666509 | 0.853 | 0.477 | 4.41E-55 | 6 SFRP2 | Muller Glia cells |
| 2.14E-59 | 0.656175 | 0.876 | 0.593 | 4.24E-55 | 6 RAX | Muller Glia cells |
| 5.77E-87 | 0.648149 | 0.442 | 0.097 | 1.14E-82 | 6 CYR61 | Muller Glia cells |
| 2.13E-46 | 0.642602 | 0.861 | 0.51 | 4.21E-42 | 6 SPP1 | Muller Glia cells |
| .37E-160 | 0.629056 | 0.696 | 0.129 | 1.86E-155 | 6 HOXB8 | Muller Glia cells |
| 1.48E-44 | 0.624495 | 0.9 | 0.738 | 2.94E-40 | 6 IER2 | Muller Glia cells |
| 2.83E-41 | 0.622053 | 0.826 | 0.605 | 5.60E-37 | 6 PRDX6 | Muller Glia cells |
| 3.25E-87 | 0.613362 | 0.711 | 0.264 | 6.43E-83 | 6 ATP1A1 | Muller Glia cells |
| .04E-114 | 0.602622 | 0.481 | 0.089 | 2.05E-110 | 6 FRZB | Muller Glia cells |
| 1.99E-65 | 0.599429 | 0.767 | 0.364 | 3.93E-61 | 6 ATP1B3 | Muller Glia cells |
| 7.74E-86 | 0.598438 | 0.994 | 0.88 | 1.53E-81 | 6 ITM2B | Muller Glia cells |
| .50E-101 | 0.597627 | 0.487 | 0.103 | 4.96E-97 | 6 EVA1B | Muller Glia cells |
| 1.49E-78 | 0.595814 | 0.77 | 0.321 | 2.96E-74 | 6 LGR4 | Muller Glia cells |
| 1.03E-61 | 0.593285 | 0.186 | 0.023 | 2.03E-57 | 6 TNFSF10 | Muller Glia cells |
| 8.67E-13 | 0.584834 | 0.215 | 0.098 | 1.72E-08 | 6 PTGDS | Muller Glia cells |
| 2.63E-60 | 0.578395 | 0.991 | 0.741 | 5.20E-56 | 6 VIM | Muller Glia cells |
| .86E-125 | 0.578028 | 0.378 | 0.049 | 3.68E-121 | 6 LHX1 | Muller Glia cells |
| .14E-109 | 0.575559 | 0.617 | 0.155 | 4.25E-105 | 6 HOXB3 | Muller Glia cells |
| 9.39E-74 | 0.571291 | 0.994 | 0.967 | 1.86E-69 | 6 RBP1 | Muller Glia cells |
| 4.17E-60 | 0.564739 | 0.735 | 0.363 | 8.25E-56 | 6 MYL12A | Muller Glia cells |
| 5.53E-78 | 0.560231 | 0.755 | 0.3 | 1.10E-73 | 6 LINC01833 | Muller Glia cells |
| .22E-100 | 0.557488 | 0.563 | 0.143 | 1.03E-95 | 6 C9orf3 | Muller Glia cells |
| 2.30E-49 | 0.547105 | 0.313 | 0.077 | 4.55E-45 | 6 GPX3 | Muller Glia cells |
| 2.36E-81 | 0.537059 | 0.761 | 0.293 | 4.67E-77 | 6 NPC2 | Muller Glia cells |
| .69E-118 | 0.53638 | 0.41 | 0.062 | 3.35E-114 | 6 CHST2 | Muller Glia cells |
| 6.14E-44 | 0.521051 | 0.785 | 0.476 | 1.22E-39 | 6 APLP2 | Muller Glia cells |
| 5.69E-22 | 0.520811 | 0.729 | 0.55 | 1.13E-17 | 6 JUNB | Muller Glia cells |
| 9.94E-48 | 0.519948 | 0.54 | 0.213 | 1.97E-43 | 6 AKAP12 | Muller Glia cells |
| 5.66E-58 | 0.518324 | 0.339 | 0.083 | 1.12E-53 | 6 CP | Muller Glia cells |
| 6.98E-21 | 0.513103 | 0.543 | 0.324 | 1.38E-16 | 6 TPM1 | Muller Glia cells |
| 5.31E-63 | 0.509691 | 0.652 | 0.272 | 1.05E-58 | 6 TSPAN4 | Muller Glia cells |
| 4.65E-39 | 0.509314 | 0.389 | 0.136 | 9.20E-35 | 6 CDH6 | Muller Glia cells |
| 2.73E-93 | 0.506314 | 0.416 | 0.08 | 5.40E-89 | 6 CAV1 | Muller Glia cells |
| .83E-252 | 1.79922 | 0.997 | 0.315 | 5.61E-248 | 7 PAX6 | Horizontal and amacrine cells |
| .13E-200 | 1.663325 | 0.997 | 0.401 | 1.81E-195 | 7 STMN2 | Horizontal and amacrine cells |
| .17E-155 | 1.471603 | 1 | 0.933 | 8.26E-151 | 7 SOX4 | Horizontal and amacrine cells |
| .43E-131 | 1.258163 | 0.981 | 0.718 | 2.82E-127 | 7 MAB21L1 | Horizontal and amacrine cells |
| 1.16E-61 | 1.253969 | 0.684 | 0.392 | 2.29E-57 | 7 MEIS2 | Horizontal and amacrine cells |

| HOXB2 4 |
| --- |
| SAT1 3 |
| NDP1 5 |
| MAL 5 |
| FOS1 |
| HES12 7 |
| PDZRN3 1 |
| COL18A11 8 |
| ATP2B4 2 |
| HOXB4 4 |
| PAX81 1 |
| CYP1B11 |
| TTYH12 |
| SLC1A31 |
| ALDH1A1 |
| EGR11 |
| CFI 1 |
| DIO32 |
| CRYM1 |
| SELENOP1 1 |
| METRN1 |
| SFRP22 |
| RAX2 |
| CYR61 |
| SPP12 |
| HOXB8 9 |
| IER21 |
| PRDX6 |
| ATP1A1 |
| FRZB 1 |
| ATP1B31 |
| ITM2B |
| EVA1B 2 |
| LGR41 |
| TNFSF10 |
| PTGDS |
| VIM2 |
| LHX1 1 |
| HOXB3 2 |
| RBP11 |
| MYL12A |
| LINC01833 |
| C9orf3 5 |
| GPX31 |
| NPC21 |
| CHST2 1 |
| APLP2 |
| JUNB1 |
| AKAP121 |
| CP |
| TPM1 |
| TSPAN4 |
| CDH61 |
| CAV1 |
| PAX6 2 |
| STMN2 9 |
| SOX4 4 |
| MAB21L1 1 |
| MEIS2 |

| RTN1 | 3.89E-261 | 1.15381 | 0.889 | 0.158 | 7.70E-257 | 7 | RTN1 | Horizontal and amacrine cells |
| --- | --- | --- | --- | --- | --- | --- | --- | --- |
| RND3 | 2.56E-125 | 1.003723 | 0.69 | 0.179 | 5.07E-121 | 7 | RND3 | Horizontal and amacrine cells |
| SYT4 | 1.21E-129 | 0.963244 | 0.628 | 0.145 | 2.40E-125 | 7 | SYT4 | Horizontal and amacrine cells |
| ONECUT2 | 1.22E-64 | 0.962378 | 0.359 | 0.084 | 2.41E-60 | 7 | ONECUT2 | Horizontal and amacrine cells |
| MAB21L2 | 1.34E-223 | 0.94415 | 0.663 | 0.09 | 2.65E-219 | 7 | MAB21L2 | Horizontal and amacrine cells |
| DCX | 5.99E-171 | 0.933858 | 0.889 | 0.233 | 1.19E-166 | 7 | DCX | Horizontal and amacrine cells |
| GRIA4 | 1.01E-268 | 0.924822 | 0.728 | 0.091 | 2.01E-264 | 7 | GRIA4 | Horizontal and amacrine cells |
| ZNF385D | 1.40E-126 | 0.910825 | 0.765 | 0.234 | 2.76E-122 | 7 | ZNF385D | Horizontal and amacrine cells |
| BASP1 | 7.53E-126 | 0.895913 | 1 | 0.779 | 1.49E-121 | 7 | BASP1 | Horizontal and amacrine cells |
| RORB1 | 6.78E-93 | 0.87608 | 0.907 | 0.5 | 1.34E-88 | 7 | RORB | Horizontal and amacrine cells |
| ZFHX4 | 8.77E-103 | 0.869809 | 0.687 | 0.22 | 1.74E-98 | 7 | ZFHX4 | Horizontal and amacrine cells |
| TFAP2A | 0 | 0.86916 | 0.495 | 0.014 | 0 | 7 | TFAP2A | Horizontal and amacrine cells |
| TUBB2A | 7.11E-107 | 0.867627 | 0.898 | 0.394 | 1.41E-102 | 7 | TUBB2A | Horizontal and amacrine cells |
| CD24 | 1.07E-123 | 0.863539 | 0.972 | 0.432 | 2.12E-119 | 7 | CD24 | Horizontal and amacrine cells |
| MIR181A1 | 1.42E-196 | 0.855498 | 0.706 | 0.123 | 2.81E-192 | 7 | MIR181A1 | Horizontal and amacrine cells |
| RUNX1T1 | 3.02E-199 | 0.847373 | 0.746 | 0.136 | 5.97E-195 | 7 | RUNX1T1 | Horizontal and amacrine cells |
| NSG1 | 3.94E-260 | 0.837697 | 0.78 | 0.107 | 7.81E-256 | 7 | NSG1 | Horizontal and amacrine cells |
| PCDH9 | 5.94E-114 | 0.824759 | 0.7 | 0.195 | 1.18E-109 | 7 | PCDH9 | Horizontal and amacrine cells |
| JPT1 | 4.06E-113 | 0.820889 | 0.975 | 0.655 | 8.04E-109 | 7 | JPT1 | Horizontal and amacrine cells |
| ZFHX3 | 2.05E-116 | 0.804613 | 0.669 | 0.186 | 4.05E-112 | 7 | ZFHX3 | Horizontal and amacrine cells |
| PARD3 | 1.92E-76 | 0.774797 | 0.477 | 0.131 | 3.81E-72 | 7 | PARD3 | Horizontal and amacrine cells |
| CRABP12 | 2.88E-46 | 0.769458 | 0.935 | 0.768 | 5.71E-42 | 7 | CRABP1 | Horizontal and amacrine cells |
| MIR124-2H | 9.23E-118 | 0.768701 | 0.836 | 0.283 | 1.83E-113 | 7 | MIR124-2H | Horizontal and amacrine cells |
| NREP | 2.17E-106 | 0.768171 | 0.975 | 0.746 | 4.30E-102 | 7 | NREP | Horizontal and amacrine cells |
| TUBB2B | 7.14E-105 | 0.76604 | 1 | 0.914 | 1.41E-100 | 7 | TUBB2B | Horizontal and amacrine cells |
| CITED2 | 4.86E-99 | 0.755719 | 0.737 | 0.257 | 9.63E-95 | 7 | CITED2 | Horizontal and amacrine cells |
| AP1S2 | 2.08E-72 | 0.754911 | 0.895 | 0.598 | 4.12E-68 | 7 | AP1S2 | Horizontal and amacrine cells |
| STMN4 | 4.69E-75 | 0.753614 | 0.746 | 0.3 | 9.29E-71 | 7 | STMN4 | Horizontal and amacrine cells |
| PCSK1N | 3.60E-99 | 0.740407 | 0.932 | 0.487 | 7.13E-95 | 7 | PCSK1N | Horizontal and amacrine cells |
| CELF4 | 2.17E-87 | 0.729691 | 0.737 | 0.28 | 4.31E-83 | 7 | CELF4 | Horizontal and amacrine cells |
| MLLT11 | 7.67E-100 | 0.718165 | 0.981 | 0.738 | 1.52E-95 | 7 | MLLT11 | Horizontal and amacrine cells |
| RBFOX2 | 3.08E-169 | 0.706067 | 0.793 | 0.179 | 6.10E-165 | 7 | RBFOX2 | Horizontal and amacrine cells |
| ARL4C | 6.07E-40 | 0.703629 | 0.793 | 0.518 | 1.20E-35 | 7 | ARL4C | Horizontal and amacrine cells |
| MARCKSL1 | 1.56E-112 | 0.703506 | 1 | 0.964 | 3.09E-108 | 7 | MARCKSL1 | Horizontal and amacrine cells |
| NRXN1 | 1.18E-134 | 0.700248 | 0.706 | 0.169 | 2.33E-130 | 7 | NRXN1 | Horizontal and amacrine cells |
| CCDC88A | 5.61E-77 | 0.697088 | 0.836 | 0.416 | 1.11E-72 | 7 | CCDC88A | Horizontal and amacrine cells |
| NSG2 | 4.18E-190 | 0.696251 | 0.65 | 0.098 | 8.27E-186 | 7 | NSG2 | Horizontal and amacrine cells |
| KIF5C | 5.74E-73 | 0.678697 | 0.737 | 0.321 | 1.14E-68 | 7 | KIF5C | Horizontal and amacrine cells |
| ZNF503 | 3.28E-123 | 0.675375 | 0.539 | 0.104 | 6.49E-119 | 7 | ZNF503 | Horizontal and amacrine cells |
| AL391650. | 6.82E-130 | 0.645539 | 0.755 | 0.197 | 1.35E-125 | 7 | AL391650. | Horizontal and amacrine cells |
| PCDH7 | 5.62E-128 | 0.642889 | 0.495 | 0.083 | 1.11E-123 | 7 | PCDH7 | Horizontal and amacrine cells |
| ELAVL3 | 2.35E-104 | 0.636125 | 0.69 | 0.199 | 4.66E-100 | 7 | ELAVL3 | Horizontal and amacrine cells |
| GRIA2 | 8.88E-152 | 0.631725 | 0.526 | 0.078 | 1.76E-147 | 7 | GRIA2 | Horizontal and amacrine cells |
| INA | 4.82E-93 | 0.621113 | 0.759 | 0.265 | 9.55E-89 | 7 | INA | Horizontal and amacrine cells |
| PKIA | 1.00E-81 | 0.603343 | 0.78 | 0.318 | 1.98E-77 | 7 | PKIA | Horizontal and amacrine cells |
| TUBA1A | 7.63E-72 | 0.603329 | 0.997 | 0.965 | 1.51E-67 | 7 | TUBA1A | Horizontal and amacrine cells |
| TMSB10 | 3.47E-71 | 0.603223 | 0.997 | 0.942 | 6.87E-67 | 7 | TMSB10 | Horizontal and amacrine cells |
| CRIM1 | 5.75E-175 | 0.600788 | 0.495 | 0.06 | 1.14E-170 | 7 | CRIM1 | Horizontal and amacrine cells |
| MARCKS | 8.26E-77 | 0.597979 | 0.997 | 0.946 | 1.64E-72 | 7 | MARCKS | Horizontal and amacrine cells |
| CRMP1 | 9.98E-79 | 0.595658 | 0.839 | 0.381 | 1.98E-74 | 7 | CRMP1 | Horizontal and amacrine cells |
| SNCA | 2.83E-57 | 0.594568 | 0.471 | 0.15 | 5.61E-53 | 7 | SNCA | Horizontal and amacrine cells |
| ONECUT1 | 1.97E-59 | 0.5829 | 0.229 | 0.037 | 3.90E-55 | 7 | ONECUT1 | Horizontal and amacrine cells |
| TTC3 | 2.25E-72 | 0.581577 | 0.981 | 0.885 | 4.45E-68 | 7 | TTC3 | Horizontal and amacrine cells |
| NNAT1 | 3.45E-54 | 0.579674 | 0.892 | 0.597 | 6.83E-50 | 7 | NNAT | Horizontal and amacrine cells |
| FNBP1L | 1.29E-70 | 0.572072 | 0.743 | 0.321 | 2.55E-66 | 7 | FNBP1L | Horizontal and amacrine cells |
| CADM2 | 8.45E-52 | 0.569117 | 0.666 | 0.301 | 1.67E-47 | 7 | CADM2 | Horizontal and amacrine cells |
| MAPT | 2.04E-123 | 0.564393 | 0.585 | 0.122 | 4.03E-119 | 7 | MAPT | Horizontal and amacrine cells |
| GNG3 | 2.94E-141 | 0.56336 | 0.659 | 0.133 | 5.82E-137 | 7 | GNG3 | Horizontal and amacrine cells |
| MGARP1 | 1.54E-85 | 0.561419 | 0.681 | 0.217 | 3.06E-81 | 7 | MGARP | Horizontal and amacrine cells |

| 2.45E-79 | 0.550564 | 0.604 | 0.194 | 4.85E-75 | 7 MEIS1 Horizontal and amacrine cells |
| --- | --- | --- | --- | --- | --- |
| .22E-185 | 0.547691 | 0.331 | 0.02 | 2.42E-181 | 7 PRDM13 Horizontal and amacrine cells |
| 7.35E-87 | 0.543908 | 0.604 | 0.177 | 1.46E-82 | 7 ELP4 Horizontal and amacrine cells |
| 9.59E-57 | 0.536649 | 0.619 | 0.262 | 1.90E-52 | 7 CSRNP3 Horizontal and amacrine cells |
| 2.00E-65 | 0.530275 | 0.765 | 0.345 | 3.95E-61 | 7 CEP170 Horizontal and amacrine cells |
| 9.74E-51 | 0.527849 | 0.724 | 0.372 | 1.93E-46 | 7 DPYSL2 Horizontal and amacrine cells |
| 6.10E-55 | 0.523606 | 0.907 | 0.714 | 1.21E-50 | 7 ETFB Horizontal and amacrine cells |
| 2.68E-70 | 0.516564 | 0.876 | 0.418 | 5.31E-66 | 7 TAGLN3 Horizontal and amacrine cells |
| 8.79E-71 | 0.515224 | 0.495 | 0.14 | 1.74E-66 | 7 LSAMP Horizontal and amacrine cells |
| 6.72E-56 | 0.514964 | 0.839 | 0.479 | 1.33E-51 | 7 PAFAH1B3 Horizontal and amacrine cells |
| 1.19E-11 | 0.511555 | 0.254 | 0.129 | 2.36E-07 | 7 PCP4 Horizontal and amacrine cells |
| 4.77E-21 | 0.509546 | 0.762 | 0.654 | 9.44E-17 | 7 CXXC5 Horizontal and amacrine cells |
| 2.69E-64 | 0.509216 | 0.771 | 0.351 | 5.33E-60 | 7 KIDINS220 Horizontal and amacrine cells |
| 9.66E-78 | 0.50875 | 0.288 | 0.045 | 1.91E-73 | 7 LHX9 Horizontal and amacrine cells |
| 1.14E-83 | 0.504957 | 0.728 | 0.252 | 2.26E-79 | 7 FEZ1 Horizontal and amacrine cells |
| .92E-225 | 2.519335 | 0.966 | 0.295 | 3.80E-221 | 8 PDE6H Retinoma-like |
| .20E-111 | 1.972325 | 0.576 | 0.143 | 1.03E-106 | 8 GUCA1A Retinoma-like |
| .22E-288 | 1.909643 | 0.771 | 0.099 | 2.41E-284 | 8 ARR3 Retinoma-like |
| .07E-207 | 1.641411 | 0.991 | 0.342 | 2.12E-203 | 8 GNB3 Retinoma-like |
| .20E-151 | 1.590808 | 0.954 | 0.405 | 2.38E-147 | 8 RCVRN Retinoma-like |
| .59E-277 | 1.473927 | 0.904 | 0.151 | 3.14E-273 | 8 KCNV2 Retinoma-like |
| .39E-175 | 1.384051 | 1 | 0.78 | 1.26E-170 | 8 GUK1 Retinoma-like |
| .01E-248 | 1.370562 | 0.985 | 0.229 | 2.00E-244 | 8 AIPL1 Retinoma-like |
| .64E-134 | 1.366131 | 0.693 | 0.175 | 3.25E-130 | 8 RRAD Retinoma-like |
| .71E-295 | 1.353825 | 0.87 | 0.126 | 3.39E-291 | 8 GNGT2 Retinoma-like |
| .45E-276 | 1.327542 | 0.981 | 0.216 | 1.87E-271 | 8 PLEKHB1 Retinoma-like |
| .19E-165 | 1.326348 | 0.997 | 0.386 | 1.22E-160 | 8 PDC Retinoma-like |
| 0 | 1.307163 | 0.913 | 0.119 | 0 | 8 MPP4 Retinoma-like |
| .46E-139 | 1.305987 | 0.972 | 0.482 | 6.85E-135 | 8 UNC119 Retinoma-like |
| .17E-199 | 1.291907 | 0.777 | 0.151 | 1.42E-194 | 8 VXN Retinoma-like |
| .33E-224 | 1.248345 | 0.938 | 0.235 | 1.25E-219 | 8 TUBA4A Retinoma-like |
| .05E-205 | 1.237479 | 0.988 | 0.299 | 1.20E-200 | 8 Sep-04 Retinoma-like |
| 0 | 1.213875 | 0.771 | 0.081 | 0 | 8 MYL4 Retinoma-like |
| .72E-197 | 1.210508 | 0.452 | 0.042 | 7.36E-193 | 8 GUCA1B Retinoma-like |
| 0 | 1.140772 | 0.82 | 0.079 | 0 | 8 RS1 Retinoma-like |
| 0 | 1.129544 | 0.824 | 0.086 | 0 | 8 RTBDN Retinoma-like |
| .76E-183 | 1.122126 | 0.994 | 0.365 | 3.49E-179 | 8 FAM57B Retinoma-like |
| .39E-244 | 1.094871 | 0.96 | 0.206 | 1.26E-239 | 8 TULP1 Retinoma-like |
| 3.28E-88 | 1.078175 | 0.808 | 0.366 | 6.49E-84 | 8 CRABP2 Retinoma-like |
| .18E-136 | 1.05402 | 0.981 | 0.595 | 1.42E-131 | 8 AKAP9 Retinoma-like |
| .22E-220 | 1.0247 | 0.768 | 0.133 | 1.03E-215 | 8 HRASLS Retinoma-like |
| .61E-126 | 1.022124 | 0.978 | 0.49 | 3.18E-122 | 8 SCG3 Retinoma-like |
| .18E-186 | 1.008738 | 0.972 | 0.27 | 8.28E-182 | 8 CRX Retinoma-like |
| 3.07E-91 | 0.989106 | 0.845 | 0.44 | 6.08E-87 | 8 DHRS7 Retinoma-like |
| .59E-234 | 0.975101 | 0.842 | 0.158 | 5.14E-230 | 8 PRCD Retinoma-like |
| .31E-226 | 0.969845 | 0.898 | 0.182 | 2.59E-222 | 8 NANOS1 Retinoma-like |
| .10E-244 | 0.961372 | 0.796 | 0.119 | 2.18E-240 | 8 RBP4 Retinoma-like |
| .70E-153 | 0.952597 | 0.901 | 0.279 | 3.38E-149 | 8 DPYSL3 Retinoma-like |
| .58E-185 | 0.920497 | 0.889 | 0.219 | 1.10E-180 | 8 RXRG Retinoma-like |
| .54E-137 | 0.916955 | 0.997 | 0.667 | 5.03E-133 | 8 ENO2 Retinoma-like |
| .49E-174 | 0.900569 | 0.895 | 0.213 | 1.09E-169 | 8 SLC38A5 Retinoma-like |
| .29E-113 | 0.888799 | 0.988 | 0.657 | 1.25E-108 | 8 CPE Retinoma-like |
| .59E-155 | 0.881528 | 0.935 | 0.336 | 5.13E-151 | 8 COTL1 Retinoma-like |
| .10E-202 | 0.875403 | 0.836 | 0.162 | 2.17E-198 | 8 IMPG2 Retinoma-like |
| .50E-181 | 0.8684 | 0.901 | 0.22 | 8.90E-177 | 8 FSTL5 Retinoma-like |
| .24E-158 | 0.862905 | 0.851 | 0.227 | 2.45E-154 | 8 IGSF21 Retinoma-like |
| 5.14E-84 | 0.853511 | 0.991 | 0.852 | 1.02E-79 | 8 TUBB4B Retinoma-like |
| 6.76E-64 | 0.837769 | 0.867 | 0.569 | 1.34E-59 | 8 MAP2 Retinoma-like |
| .68E-132 | 0.836275 | 0.91 | 0.318 | 5.30E-128 | 8 ATP1A3 Retinoma-like |

| MEIS1 |
| --- |
| PRDM13 1 |
| ELP4 |
| CSRNP3 |
| CEP170 |
| DPYSL2 |
| ETFB |
| TAGLN3 |
| LSAMP |
| PAFAH1B3 |
| PCP4 |
| CXXC5 |
| KIDINS220 |
| LHX9 |
| FEZ1 |
| PDE6H1 1 |
| GUCA1A 5 |
| ARR3 1 |
| GNB32 1 |
| RCVRN2 1 |
| KCNV21 1 |
| GUK11 6 |
| AIPL12 1 |
| RRAD2 1 |
| GNGT21 1 |
| PLEKHB11 9 |
| PDC2 6 |
| MPP41 |
| UNC1191 3 |
| VXN1 7 |
| TUBA4A1 6 |
| Sep-42 6 |
| MYL4 |
| GUCA1B 3 |
| RS1 |
| RTBDN |
| FAM57B2 1 |
| TULP12 6 |
| CRABP21 |
| AKAP91 7 |
| HRASLS 5 |
| SCG32 1 |
| CRX2 4 |
| DHRS7 |
| PRCD 2 |
| NANOS1 1 |
| RBP4 1 |
| DPYSL3 1 |
| RXRG1 5 |
| ENO21 2 |
| SLC38A52 5 |
| CPE1 6 |
| COTL1 2 |
| IMPG21 1 |
| FSTL51 4 |
| IGSF211 1 |
| TUBB4B1 |
| MAP21 |
| ATP1A31 2 |

| CA2 | 7.74E-87 | 0.824029 | 0.641 | 0.2 | 1.53E-82 | 8 | CA2 | Retinoma-like |
| --- | --- | --- | --- | --- | --- | --- | --- | --- |
| CPLX3 | 7.24E-87 | 0.806765 | 0.604 | 0.184 | 1.43E-82 | 8 | CPLX3 | Retinoma-like |
| MAP1LC3A | 2.91E-128 | 0.801539 | 0.954 | 0.37 | 5.76E-124 | 8 | MAP1LC3A | Retinoma-like |
| CPLX41 | 1.24E-145 | 0.790204 | 0.632 | 0.124 | 2.46E-141 | 8 | CPLX4 | Retinoma-like |
| NME1 | 2.38E-113 | 0.7736 | 0.932 | 0.435 | 4.71E-109 | 8 | NME1 | Retinoma-like |
| UQCC2 | 1.26E-84 | 0.763893 | 0.811 | 0.366 | 2.50E-80 | 8 | UQCC2 | Retinoma-like |
| PHOX2A | 1.40E-159 | 0.758335 | 0.464 | 0.056 | 2.77E-155 | 8 | PHOX2A | Retinoma-like |
| CHCHD10 | 1.84E-177 | 0.757972 | 0.867 | 0.214 | 3.64E-173 | 8 | CHCHD10 | Retinoma-like |
| FAM107A | 1.78E-304 | 0.751482 | 0.793 | 0.091 | 3.53E-300 | 8 | FAM107A | Retinoma-like |
| PEX5L | 1.14E-154 | 0.749735 | 0.731 | 0.158 | 2.25E-150 | 8 | PEX5L | Retinoma-like |
| NT5DC2 | 6.51E-75 | 0.745506 | 0.746 | 0.345 | 1.29E-70 | 8 | NT5DC2 | Retinoma-like |
| PTP4A32 | 6.05E-124 | 0.740273 | 0.861 | 0.28 | 1.20E-119 | 8 | PTP4A3 | Retinoma-like |
| GNAT2 | 3.43E-291 | 0.733141 | 0.709 | 0.073 | 6.80E-287 | 8 | GNAT2 | Retinoma-like |
| NRN11 | 1.17E-99 | 0.73208 | 0.737 | 0.231 | 2.32E-95 | 8 | NRN1 | Retinoma-like |
| RBP31 | 5.51E-186 | 0.729076 | 0.74 | 0.13 | 1.09E-181 | 8 | RBP3 | Retinoma-like |
| PCBP42 | 8.32E-86 | 0.717238 | 0.969 | 0.535 | 1.65E-81 | 8 | PCBP4 | Retinoma-like |
| PRDX11 | 5.30E-60 | 0.709761 | 0.947 | 0.871 | 1.05E-55 | 8 | PRDX1 | Retinoma-like |
| LAPTM4B1 | 3.18E-97 | 0.707739 | 0.963 | 0.633 | 6.29E-93 | 8 | LAPTM4B | Retinoma-like |
| NEUROD12 | 1.37E-106 | 0.704235 | 0.944 | 0.343 | 2.70E-102 | 8 | NEUROD1 | Retinoma-like |
| OLFM1 | 6.38E-113 | 0.699577 | 0.765 | 0.224 | 1.26E-108 | 8 | OLFM1 | Retinoma-like |
| SLC17A7 | 1.43E-182 | 0.695288 | 0.796 | 0.16 | 2.83E-178 | 8 | SLC17A7 | Retinoma-like |
| CC2D2A | 6.21E-178 | 0.693544 | 0.762 | 0.156 | 1.23E-173 | 8 | CC2D2A | Retinoma-like |
| MIR7-3HG | 1.13E-97 | 0.674702 | 0.681 | 0.204 | 2.25E-93 | 8 | MIR7-3HG | Retinoma-like |
| ABHD14A | 6.16E-109 | 0.672355 | 0.824 | 0.297 | 1.22E-104 | 8 | ABHD14A | Retinoma-like |
| ZNF385A1 | 1.07E-112 | 0.665041 | 0.898 | 0.347 | 2.11E-108 | 8 | ZNF385A | Retinoma-like |
| NEDD4L | 5.11E-124 | 0.66194 | 0.715 | 0.193 | 1.01E-119 | 8 | NEDD4L | Retinoma-like |
| PKM1 | 1.78E-99 | 0.659929 | 0.997 | 0.933 | 3.52E-95 | 8 | PKM | Retinoma-like |
| STX31 | 2.65E-127 | 0.652551 | 0.783 | 0.215 | 5.25E-123 | 8 | STX3 | Retinoma-like |
| PROM1 | 3.46E-161 | 0.645244 | 0.653 | 0.12 | 6.85E-157 | 8 | PROM1 | Retinoma-like |
| SEZ6L2 | 4.66E-91 | 0.63704 | 0.783 | 0.289 | 9.23E-87 | 8 | SEZ6L2 | Retinoma-like |
| RP11 | 8.06E-139 | 0.634609 | 0.573 | 0.099 | 1.60E-134 | 8 | RP1 | Retinoma-like |
| SYP2 | 5.04E-99 | 0.618851 | 0.892 | 0.335 | 9.98E-95 | 8 | SYP | Retinoma-like |
| FAIM | 9.55E-79 | 0.618789 | 0.474 | 0.122 | 1.89E-74 | 8 | FAIM | Retinoma-like |
| IMPDH1 | 5.25E-180 | 0.608563 | 0.69 | 0.12 | 1.04E-175 | 8 | IMPDH1 | Retinoma-like |
| TPD52 | 1.37E-66 | 0.605946 | 0.762 | 0.327 | 2.71E-62 | 8 | TPD52 | Retinoma-like |
| NEUROG12 | 2.72E-81 | 0.60544 | 0.628 | 0.183 | 5.38E-77 | 8 | NEUROG1 | Retinoma-like |
| ECHDC2 | 1.07E-171 | 0.599908 | 0.74 | 0.145 | 2.12E-167 | 8 | ECHDC2 | Retinoma-like |
| MIF | 1.92E-50 | 0.593807 | 0.972 | 0.931 | 3.80E-46 | 8 | MIF | Retinoma-like |
| CNTNAP2 | 1.15E-137 | 0.589223 | 0.712 | 0.161 | 2.27E-133 | 8 | CNTNAP2 | Retinoma-like |
| PPP1CC1 | 1.62E-40 | 0.58701 | 0.793 | 0.486 | 3.21E-36 | 8 | PPP1CC | Retinoma-like |
| RD3 | 1.60E-163 | 0.582276 | 0.641 | 0.11 | 3.17E-159 | 8 | RD3 | Retinoma-like |
| IFI27L2 | 6.10E-110 | 0.579002 | 0.789 | 0.25 | 1.21E-105 | 8 | IFI27L2 | Retinoma-like |
| UGCG | 1.33E-84 | 0.578665 | 0.737 | 0.262 | 2.64E-80 | 8 | UGCG | Retinoma-like |
| NR2F6 | 5.37E-85 | 0.57823 | 0.901 | 0.449 | 1.06E-80 | 8 | NR2F6 | Retinoma-like |
| GUCA1C | 1.47E-270 | 0.576973 | 0.387 | 0.016 | 2.90E-266 | 8 | GUCA1C | Retinoma-like |
| COX17 | 1.05E-88 | 0.573866 | 0.842 | 0.358 | 2.08E-84 | 8 | COX17 | Retinoma-like |
| CALCOCO2 | 3.18E-109 | 0.572703 | 0.755 | 0.233 | 6.29E-105 | 8 | CALCOCO2 | Retinoma-like |
| ARL6IP5 | 1.66E-48 | 0.571359 | 0.808 | 0.49 | 3.29E-44 | 8 | ARL6IP5 | Retinoma-like |
| ATP5MC1 | 7.23E-82 | 0.567172 | 0.879 | 0.426 | 1.43E-77 | 8 | ATP5MC1 | Retinoma-like |
| LMOD1 | 1.18E-194 | 0.566456 | 0.622 | 0.084 | 2.34E-190 | 8 | LMOD1 | Retinoma-like |
| CHCHD21 | 1.64E-18 | 0.565376 | 0.647 | 0.542 | 3.24E-14 | 8 | CHCHD2 | Retinoma-like |
| TMEM14B | 4.66E-79 | 0.565282 | 0.963 | 0.656 | 9.22E-75 | 8 | TMEM14B | Retinoma-like |
| FAM19A4 | 6.77E-117 | 0.562576 | 0.659 | 0.151 | 1.34E-112 | 8 | FAM19A4 | Retinoma-like |
| NDUFB1 | 1.01E-71 | 0.560603 | 0.885 | 0.462 | 2.01E-67 | 8 | NDUFB1 | Retinoma-like |
| NTM | 1.05E-102 | 0.557009 | 0.7 | 0.194 | 2.08E-98 | 8 | NTM | Retinoma-like |
| PARP1 | 1.82E-72 | 0.554994 | 0.889 | 0.476 | 3.60E-68 | 8 | PARP1 | Retinoma-like |
| KIF2A | 7.25E-69 | 0.552039 | 0.771 | 0.353 | 1.44E-64 | 8 | KIF2A | Retinoma-like |
| SNAP25 | 6.90E-93 | 0.550246 | 0.814 | 0.293 | 1.37E-88 | 8 | SNAP25 | Retinoma-like |
| TMEM54 | 8.43E-152 | 0.548962 | 0.656 | 0.124 | 1.67E-147 | 8 | TMEM54 | Retinoma-like |

| NHP2 | 2.42E-53 | 0.545986 | 0.796 | 0.458 | 4.80E-49 | 8 | NHP2 | Retinoma-like |
| --- | --- | --- | --- | --- | --- | --- | --- | --- |
| AGAP1 | 6.94E-75 | 0.544696 | 0.759 | 0.294 | 1.37E-70 | 8 | AGAP1 | Retinoma-like |
| ALDOC1 | 5.72E-82 | 0.540099 | 0.548 | 0.151 | 1.13E-77 | 8 | ALDOC | Retinoma-like |
| CYP26B1 | 4.62E-47 | 0.535943 | 0.378 | 0.11 | 9.15E-43 | 8 | CYP26B1 | Retinoma-like |
| TMA7 | 3.81E-65 | 0.535127 | 0.978 | 0.822 | 7.55E-61 | 8 | TMA7 | Retinoma-like |
| COX7B | 1.32E-47 | 0.534963 | 0.935 | 0.767 | 2.62E-43 | 8 | COX7B | Retinoma-like |
| CHRNA3 | 3.67E-115 | 0.528016 | 0.616 | 0.136 | 7.27E-111 | 8 | CHRNA3 | Retinoma-like |
| MLXIP | 1.30E-100 | 0.52586 | 0.616 | 0.157 | 2.58E-96 | 8 | MLXIP | Retinoma-like |
| TMX4 | 1.99E-27 | 0.525116 | 0.607 | 0.339 | 3.93E-23 | 8 | TMX4 | Retinoma-like |
| ARL4D | 3.48E-70 | 0.523492 | 0.848 | 0.394 | 6.89E-66 | 8 | ARL4D | Retinoma-like |
| PAQR4 | 8.34E-104 | 0.52211 | 0.734 | 0.217 | 1.65E-99 | 8 | PAQR4 | Retinoma-like |
| ANKRD33B | 8.08E-123 | 0.520799 | 0.632 | 0.136 | 1.60E-118 | 8 | ANKRD33B | Retinoma-like |
| FAM213A | 3.72E-73 | 0.513885 | 0.926 | 0.488 | 7.37E-69 | 8 | FAM213A | Retinoma-like |
| PPA1 | 2.61E-54 | 0.512889 | 0.941 | 0.744 | 5.16E-50 | 8 | PPA1 | Retinoma-like |
| C1QTNF4 | 4.65E-77 | 0.512486 | 0.669 | 0.232 | 9.21E-73 | 8 | C1QTNF4 | Retinoma-like |
| THRB | 5.69E-142 | 0.509963 | 0.455 | 0.061 | 1.13E-137 | 8 | THRB | Retinoma-like |
| SLC38A1 | 5.23E-69 | 0.50924 | 0.87 | 0.389 | 1.04E-64 | 8 | SLC38A1 | Retinoma-like |
| SOX7 | 6.78E-157 | 0.505989 | 0.508 | 0.067 | 1.34E-152 | 8 | SOX7 | Retinoma-like |
| DCT2 | 7.75E-56 | 0.505652 | 0.579 | 0.197 | 1.53E-51 | 8 | DCT | Retinoma-like |
| EYS1 | 1.62E-122 | 0.505105 | 0.579 | 0.115 | 3.21E-118 | 8 | EYS | Retinoma-like |
| VTN | 4.76E-139 | 0.503861 | 0.452 | 0.061 | 9.43E-135 | 8 | VTN | Retinoma-like |
| INSR | 3.61E-58 | 0.502984 | 0.598 | 0.224 | 7.15E-54 | 8 | INSR | Retinoma-like |
| TSPAN7 | 4.83E-82 | 0.501527 | 0.728 | 0.259 | 9.55E-78 | 8 | TSPAN7 | Retinoma-like |
| PKIB | 5.71E-95 | 0.501209 | 0.641 | 0.175 | 1.13E-90 | 8 | PKIB | Retinoma-like |
| PLTP | 7.22E-77 | 0.501118 | 0.632 | 0.203 | 1.43E-72 | 8 | PLTP | Retinoma-like |
| HOXB51 | 1.08E-301 | 2.051038 | 0.901 | 0.15 | 2.14E-297 | 9 | HOXB5 | Retinal Ganglion cells |
| HOXB81 | 0 | 1.700065 | 0.908 | 0.12 | 0 | 9 | HOXB8 | Retinal Ganglion cells |
| HOXB61 | 0 | 1.533846 | 0.828 | 0.105 | 0 | 9 | HOXB6 | Retinal Ganglion cells |
| STMN21 | 8.24E-116 | 1.284525 | 0.898 | 0.407 | 1.63E-111 | 9 | STMN2 | Retinal Ganglion cells |
| TUBA1A1 | 1.34E-141 | 1.253033 | 1 | 0.965 | 2.65E-137 | 9 | TUBA1A | Retinal Ganglion cells |
| HOXB21 | 1.35E-129 | 1.186999 | 0.841 | 0.321 | 2.67E-125 | 9 | HOXB2 | Retinal Ganglion cells |
| BASP11 | 4.49E-141 | 1.185356 | 1 | 0.779 | 8.88E-137 | 9 | BASP1 | Retinal Ganglion cells |
| TUBB2A1 | 2.24E-123 | 1.121903 | 0.892 | 0.395 | 4.43E-119 | 9 | TUBB2A | Retinal Ganglion cells |
| CBLN1 | 0 | 1.039526 | 0.666 | 0.056 | 0 | 9 | CBLN1 | Retinal Ganglion cells |
| TUBB2B1 | 1.21E-111 | 0.977561 | 1 | 0.914 | 2.40E-107 | 9 | TUBB2B | Retinal Ganglion cells |
| DCX1 | 7.15E-131 | 0.967395 | 0.79 | 0.24 | 1.42E-126 | 9 | DCX | Retinal Ganglion cells |
| HOXB9 | 1.95E-123 | 0.963458 | 0.344 | 0.039 | 3.86E-119 | 9 | HOXB9 | Retinal Ganglion cells |
| GAP43 | 1.76E-116 | 0.954755 | 0.752 | 0.236 | 3.48E-112 | 9 | GAP43 | Retinal Ganglion cells |
| AC007614. | 1.07E-237 | 0.91976 | 0.497 | 0.04 | 2.12E-233 | 9 | AC007614. | Retinal Ganglion cells |
| RND31 | 2.03E-97 | 0.909243 | 0.637 | 0.183 | 4.01E-93 | 9 | RND3 | Retinal Ganglion cells |
| PCSK1N1 | 8.91E-102 | 0.908294 | 0.904 | 0.489 | 1.76E-97 | 9 | PCSK1N | Retinal Ganglion cells |
| RTN11 | 7.46E-103 | 0.89892 | 0.637 | 0.173 | 1.48E-98 | 9 | RTN1 | Retinal Ganglion cells |
| HOXB7 | 7.12E-210 | 0.875964 | 0.659 | 0.092 | 1.41E-205 | 9 | HOXB7 | Retinal Ganglion cells |
| NOVA1 | 3.82E-87 | 0.81913 | 0.682 | 0.241 | 7.56E-83 | 9 | NOVA1 | Retinal Ganglion cells |
| STMN41 | 2.88E-89 | 0.817797 | 0.78 | 0.299 | 5.70E-85 | 9 | STMN4 | Retinal Ganglion cells |
| MLLT111 | 2.17E-100 | 0.798236 | 0.994 | 0.737 | 4.30E-96 | 9 | MLLT11 | Retinal Ganglion cells |
| SYT41 | 8.07E-58 | 0.789914 | 0.481 | 0.154 | 1.60E-53 | 9 | SYT4 | Retinal Ganglion cells |
| SOX111 | 6.97E-67 | 0.7877 | 0.924 | 0.618 | 1.38E-62 | 9 | SOX11 | Retinal Ganglion cells |
| SNCA1 | 2.78E-106 | 0.784518 | 0.586 | 0.144 | 5.50E-102 | 9 | SNCA | Retinal Ganglion cells |
| CD241 | 1.01E-87 | 0.773675 | 0.892 | 0.437 | 2.00E-83 | 9 | CD24 | Retinal Ganglion cells |
| UCHL1 | 1.05E-59 | 0.763076 | 0.825 | 0.515 | 2.07E-55 | 9 | UCHL1 | Retinal Ganglion cells |
| DNER1 | 3.03E-86 | 0.747017 | 0.742 | 0.287 | 5.99E-82 | 9 | DNER | Retinal Ganglion cells |
| ELAVL4 | 1.14E-245 | 0.74106 | 0.58 | 0.054 | 2.26E-241 | 9 | ELAVL4 | Retinal Ganglion cells |
| TMSB101 | 3.43E-74 | 0.724467 | 1 | 0.941 | 6.78E-70 | 9 | TMSB10 | Retinal Ganglion cells |
| NSG11 | 2.83E-150 | 0.713585 | 0.637 | 0.116 | 5.61E-146 | 9 | NSG1 | Retinal Ganglion cells |
| HOXB41 | 1.74E-140 | 0.698418 | 0.621 | 0.12 | 3.45E-136 | 9 | HOXB4 | Retinal Ganglion cells |
| HOXB31 | 7.52E-130 | 0.690226 | 0.662 | 0.154 | 1.49E-125 | 9 | HOXB3 | Retinal Ganglion cells |
| JPT11 | 6.80E-71 | 0.671338 | 0.952 | 0.657 | 1.35E-66 | 9 | JPT1 | Retinal Ganglion cells |
| CRABP13 | 1.49E-43 | 0.668271 | 0.955 | 0.767 | 2.94E-39 | 9 | CRABP1 | Retinal Ganglion cells |

| POU3F1 | 1.50E-49 | 0.66189 | 0.516 | 0.205 | 2.98E-45 | 9 | POU3F1 | Retinal Ganglion cells |
| --- | --- | --- | --- | --- | --- | --- | --- | --- |
| KIF5C1 | 7.88E-83 | 0.65387 | 0.777 | 0.319 | 1.56E-78 | 9 | KIF5C | Retinal Ganglion cells |
| GNG31 | 1.18E-133 | 0.643325 | 0.643 | 0.135 | 2.34E-129 | 9 | GNG3 | Retinal Ganglion cells |
| ANK3 | 1.67E-87 | 0.640423 | 0.672 | 0.225 | 3.31E-83 | 9 | ANK3 | Retinal Ganglion cells |
| RBFOX21 | 9.64E-104 | 0.63204 | 0.666 | 0.187 | 1.91E-99 | 9 | RBFOX2 | Retinal Ganglion cells |
| C4orf48 | 2.59E-63 | 0.623016 | 0.793 | 0.422 | 5.13E-59 | 9 | C4orf48 | Retinal Ganglion cells |
| EEF1A2 | 2.64E-66 | 0.618173 | 0.787 | 0.353 | 5.22E-62 | 9 | EEF1A2 | Retinal Ganglion cells |
| NRXN11 | 4.55E-76 | 0.588898 | 0.58 | 0.177 | 9.00E-72 | 9 | NRXN1 | Retinal Ganglion cells |
| TSHZ2 | 2.58E-76 | 0.584961 | 0.529 | 0.153 | 5.10E-72 | 9 | TSHZ2 | Retinal Ganglion cells |
| MAP1B | 1.05E-62 | 0.580602 | 1 | 0.899 | 2.07E-58 | 9 | MAP1B | Retinal Ganglion cells |
| ADCYAP1 | 0 | 0.57065 | 0.408 | 0.012 | 0 | 9 | ADCYAP1 | Retinal Ganglion cells |
| APP | 8.58E-44 | 0.569849 | 0.729 | 0.422 | 1.70E-39 | 9 | APP | Retinal Ganglion cells |
| CALM2 | 1.50E-66 | 0.569145 | 1 | 0.986 | 2.97E-62 | 9 | CALM2 | Retinal Ganglion cells |
| CELF41 | 8.77E-62 | 0.567097 | 0.688 | 0.283 | 1.74E-57 | 9 | CELF4 | Retinal Ganglion cells |
| NSG21 | 1.75E-129 | 0.564045 | 0.567 | 0.104 | 3.46E-125 | 9 | NSG2 | Retinal Ganglion cells |
| PRPH | 1.43E-140 | 0.557774 | 0.296 | 0.023 | 2.84E-136 | 9 | PRPH | Retinal Ganglion cells |
| TCEAL7 | 1.25E-41 | 0.5554 | 0.892 | 0.689 | 2.47E-37 | 9 | TCEAL7 | Retinal Ganglion cells |
| BEX2 | 1.25E-54 | 0.554739 | 0.949 | 0.794 | 2.48E-50 | 9 | BEX2 | Retinal Ganglion cells |
| CRMP11 | 4.23E-60 | 0.552284 | 0.796 | 0.384 | 8.37E-56 | 9 | CRMP1 | Retinal Ganglion cells |
| CDKN1C | 5.25E-39 | 0.551849 | 0.615 | 0.304 | 1.04E-34 | 9 | CDKN1C | Retinal Ganglion cells |
| FNBP1L1 | 9.19E-55 | 0.547758 | 0.682 | 0.325 | 1.82E-50 | 9 | FNBP1L | Retinal Ganglion cells |
| CALM1 | 9.99E-50 | 0.538305 | 0.962 | 0.789 | 1.98E-45 | 9 | CALM1 | Retinal Ganglion cells |
| LY6H | 1.38E-96 | 0.534077 | 0.471 | 0.095 | 2.74E-92 | 9 | LY6H | Retinal Ganglion cells |
| RALYL | 1.85E-137 | 0.524437 | 0.414 | 0.051 | 3.66E-133 | 9 | RALYL | Retinal Ganglion cells |
| PKIA1 | 2.11E-53 | 0.524356 | 0.682 | 0.324 | 4.19E-49 | 9 | PKIA | Retinal Ganglion cells |
| PLPPR3 | 2.31E-65 | 0.516218 | 0.732 | 0.312 | 4.58E-61 | 9 | PLPPR3 | Retinal Ganglion cells |
| IGFBP5 | 4.02E-10 | 0.50969 | 0.468 | 0.328 | 7.95E-06 | 9 | IGFBP5 | Retinal Ganglion cells |
| NEFM | 4.51E-120 | 0.508908 | 0.28 | 0.024 | 8.93E-116 | 9 | NEFM | Retinal Ganglion cells |
| CAMK2N1 | 9.59E-39 | 0.5024 | 0.774 | 0.476 | 1.90E-34 | 9 | CAMK2N1 | Retinal Ganglion cells |
| NREP1 | 5.15E-40 | 0.502185 | 0.908 | 0.75 | 1.02E-35 | 9 | NREP | Retinal Ganglion cells |
| VSX11 | 0 | 1.838508 | 0.864 | 0.112 | 0 | 10 | VSX1 | Bipolar cells |
| GSG11 | 6.09E-231 | 1.376946 | 0.811 | 0.139 | 1.21E-226 | 10 | GSG1 | Bipolar cells |
| CADPS1 | 6.03E-142 | 1.14822 | 0.884 | 0.294 | 1.19E-137 | 10 | CADPS | Bipolar cells |
| CRYBG3 | 1.59E-165 | 1.038588 | 0.88 | 0.237 | 3.15E-161 | 10 | CRYBG3 | Bipolar cells |
| TAGLN31 | 2.88E-95 | 1.033877 | 0.884 | 0.419 | 5.69E-91 | 10 | TAGLN3 | Bipolar cells |
| OTX22 | 1.31E-145 | 0.937357 | 0.934 | 0.298 | 2.60E-141 | 10 | OTX2 | Bipolar cells |
| TMEM215 | 4.87E-251 | 0.8941 | 0.508 | 0.037 | 9.65E-247 | 10 | TMEM215 | Bipolar cells |
| BTBD8 | 6.72E-148 | 0.849766 | 0.804 | 0.21 | 1.33E-143 | 10 | BTBD8 | Bipolar cells |
| NEUROD42 | 1.30E-119 | 0.848661 | 0.811 | 0.242 | 2.57E-115 | 10 | NEUROD4 | Bipolar cells |
| LINC00599 | 5.96E-125 | 0.826991 | 0.88 | 0.285 | 1.18E-120 | 10 | LINC00599 | Bipolar cells |
| FEZF2 | 2.70E-58 | 0.76919 | 0.558 | 0.209 | 5.34E-54 | 10 | FEZF2 | Bipolar cells |
| CA10 | 0 | 0.754239 | 0.635 | 0.04 | 0 | 10 | CA10 | Bipolar cells |
| BTG1 | 4.52E-79 | 0.752074 | 0.993 | 0.881 | 8.94E-75 | 10 | BTG1 | Bipolar cells |
| TPD521 | 1.68E-102 | 0.744668 | 0.857 | 0.324 | 3.34E-98 | 10 | TPD52 | Bipolar cells |
| PCBP43 | 2.08E-91 | 0.743392 | 0.977 | 0.536 | 4.12E-87 | 10 | PCBP4 | Bipolar cells |
| IRX6 | 1.94E-207 | 0.718742 | 0.409 | 0.028 | 3.83E-203 | 10 | IRX6 | Bipolar cells |
| CCNG2 | 1.58E-89 | 0.707053 | 0.718 | 0.248 | 3.12E-85 | 10 | CCNG2 | Bipolar cells |
| FABP72 | 8.70E-24 | 0.700376 | 0.827 | 0.686 | 1.72E-19 | 10 | FABP7 | Bipolar cells |
| RCOR2 | 2.56E-87 | 0.677455 | 0.787 | 0.31 | 5.07E-83 | 10 | RCOR2 | Bipolar cells |
| LHX4 | 9.12E-202 | 0.669272 | 0.681 | 0.097 | 1.81E-197 | 10 | LHX4 | Bipolar cells |
| INSM11 | 1.01E-58 | 0.614292 | 0.728 | 0.31 | 2.00E-54 | 10 | INSM1 | Bipolar cells |
| SYT1 | 1.41E-65 | 0.611345 | 0.947 | 0.652 | 2.80E-61 | 10 | SYT1 | Bipolar cells |
| C11orf961 | 5.43E-49 | 0.599619 | 0.495 | 0.168 | 1.07E-44 | 10 | C11orf96 | Bipolar cells |
| ZNF385B | 2.44E-148 | 0.585438 | 0.585 | 0.095 | 4.82E-144 | 10 | ZNF385B | Bipolar cells |
| SLC38A11 | 5.51E-63 | 0.575675 | 0.824 | 0.393 | 1.09E-58 | 10 | SLC38A1 | Bipolar cells |
| C3orf70 | 5.29E-149 | 0.556803 | 0.505 | 0.07 | 1.05E-144 | 10 | C3orf70 | Bipolar cells |
| SYP3 | 1.52E-71 | 0.55666 | 0.821 | 0.34 | 3.00E-67 | 10 | SYP | Bipolar cells |
| NETO1 | 5.92E-215 | 0.551647 | 0.395 | 0.024 | 1.17E-210 | 10 | NETO1 | Bipolar cells |
| IGSF212 | 1.99E-89 | 0.547145 | 0.767 | 0.234 | 3.93E-85 | 10 | IGSF21 | Bipolar cells |

| ZFHX41 | 3.52E-66 | 0.546733 | 0.635 | 0.224 | 6.96E-62 | 10 | ZFHX4 | Bipolar cells |
| --- | --- | --- | --- | --- | --- | --- | --- | --- |
| CAMK2N11 | 2.02E-53 | 0.545921 | 0.844 | 0.473 | 4.00E-49 | 10 | CAMK2N1 | Bipolar cells |
| AC027031. | 5.79E-53 | 0.542551 | 0.615 | 0.242 | 1.15E-48 | 10 | AC027031. | Bipolar cells |
| PIK3R11 | 2.99E-41 | 0.530841 | 0.718 | 0.376 | 5.92E-37 | 10 | PIK3R1 | Bipolar cells |
| HEBP1 | 6.10E-54 | 0.526416 | 0.658 | 0.288 | 1.21E-49 | 10 | HEBP1 | Bipolar cells |
| SPCS11 | 6.97E-54 | 0.524001 | 0.94 | 0.84 | 1.38E-49 | 10 | SPCS1 | Bipolar cells |
| MIR124-2H | 1.49E-63 | 0.520709 | 0.721 | 0.291 | 2.95E-59 | 10 | MIR124-2H | Bipolar cells |
| CPE2 | 9.61E-52 | 0.516873 | 0.957 | 0.66 | 1.90E-47 | 10 | CPE | Bipolar cells |
| NPR3 | 1.09E-153 | 0.51586 | 0.289 | 0.018 | 2.16E-149 | 10 | NPR3 | Bipolar cells |
| SOX41 | 6.60E-65 | 0.512017 | 1 | 0.933 | 1.31E-60 | 10 | SOX4 | Bipolar cells |
| PPP1CC2 | 7.98E-39 | 0.507931 | 0.797 | 0.487 | 1.58E-34 | 10 | PPP1CC | Bipolar cells |
| PPFIA2 | 3.87E-62 | 0.502029 | 0.625 | 0.223 | 7.67E-58 | 10 | PPFIA2 | Bipolar cells |
| SLITRK6 | 8.57E-154 | 0.501676 | 0.442 | 0.049 | 1.70E-149 | 10 | SLITRK6 | Bipolar cells |
| CHGB | 3.39E-08 | 0.612968 | 0.336 | 0.216 | 0.000672 | 12 | CHGB | Bipolar cells |
| GSG12 | 5.71E-45 | 0.597576 | 0.502 | 0.159 | 1.13E-40 | 12 | GSG1 | Bipolar cells |
| COL1A1 | 3.61E-162 | 2.853307 | 0.478 | 0.043 | 7.15E-158 | 13 | COL1A1 | Fibroblasts |
| MGP | 8.62E-150 | 2.756773 | 0.414 | 0.033 | 1.71E-145 | 13 | MGP | Fibroblasts |
| COL1A2 | 1.25E-53 | 2.26249 | 0.606 | 0.22 | 2.47E-49 | 13 | COL1A2 | Fibroblasts |
| COL3A1 | 3.60E-268 | 2.059504 | 0.448 | 0.017 | 7.13E-264 | 13 | COL3A1 | Fibroblasts |
| LGALS1 | 1.09E-223 | 1.53788 | 0.512 | 0.033 | 2.16E-219 | 13 | LGALS1 | Fibroblasts |
| S100A6 | 8.58E-91 | 1.507965 | 0.532 | 0.102 | 1.70E-86 | 13 | S100A6 | Fibroblasts |
| CXCL14 | 4.34E-137 | 1.444628 | 0.241 | 0.01 | 8.60E-133 | 13 | CXCL14 | Fibroblasts |
| S100A11 | 5.48E-254 | 1.343829 | 0.576 | 0.037 | 1.08E-249 | 13 | S100A11 | Fibroblasts |
| LUM | 1.31E-119 | 1.326906 | 0.261 | 0.015 | 2.59E-115 | 13 | LUM | Fibroblasts |
| FN1 | 2.84E-102 | 1.213558 | 0.34 | 0.034 | 5.61E-98 | 13 | FN1 | Fibroblasts |
| APOE | 1.55E-14 | 1.207395 | 0.389 | 0.204 | 3.07E-10 | 13 | APOE | Fibroblasts |
| KRT19 | 1.89E-166 | 1.205967 | 0.276 | 0.01 | 3.75E-162 | 13 | KRT19 | Fibroblasts |
| S100A10 | 2.08E-188 | 1.197225 | 0.552 | 0.048 | 4.12E-184 | 13 | S100A10 | Fibroblasts |
| IGFBP71 | 1.25E-82 | 1.189826 | 0.517 | 0.103 | 2.48E-78 | 13 | IGFBP7 | Fibroblasts |
| SPARC | 2.91E-48 | 1.188881 | 0.645 | 0.273 | 5.75E-44 | 13 | SPARC | Fibroblasts |
| KRT17 | 1.88E-193 | 1.172555 | 0.202 | 0.002 | 3.72E-189 | 13 | KRT17 | Fibroblasts |
| KRT12 | 9.45E-149 | 1.148789 | 0.138 | 0.001 | 1.87E-144 | 13 | KRT12 | Fibroblasts |
| B2M | 1.15E-47 | 1.134829 | 0.882 | 0.568 | 2.28E-43 | 13 | B2M | Fibroblasts |
| ANXA2 | 4.15E-100 | 1.130786 | 0.685 | 0.167 | 8.23E-96 | 13 | ANXA2 | Fibroblasts |
| PMEL | 0.007068 | 1.009615 | 0.108 | 0.067 | 1 | 13 | PMEL | Fibroblasts |
| DCN | 0 | 1.002229 | 0.365 | 0.004 | 0 | 13 | DCN | Fibroblasts |
| PTGDS1 | 2.07E-13 | 0.987074 | 0.246 | 0.1 | 4.10E-09 | 13 | PTGDS | Fibroblasts |
| KRT13 | 2.17E-195 | 0.961384 | 0.158 | 0 | 4.30E-191 | 13 | KRT13 | Fibroblasts |
| ID3 | 1.02E-31 | 0.933594 | 0.522 | 0.215 | 2.03E-27 | 13 | ID3 | Fibroblasts |
| ANXA1 | 3.04E-209 | 0.914221 | 0.419 | 0.021 | 6.02E-205 | 13 | ANXA1 | Fibroblasts |
| HSPB1 | 1.95E-51 | 0.886949 | 0.581 | 0.213 | 3.85E-47 | 13 | HSPB1 | Fibroblasts |
| HSPA1A1 | 9.38E-14 | 0.879247 | 0.399 | 0.22 | 1.86E-09 | 13 | HSPA1A | Fibroblasts |
| S100A4 | 9.93E-32 | 0.878221 | 0.246 | 0.056 | 1.97E-27 | 13 | S100A4 | Fibroblasts |
| RPL41 | 1.92E-68 | 0.850457 | 1 | 0.995 | 3.79E-64 | 13 | RPL41 | Fibroblasts |
| ADIRF | 2.20E-115 | 0.837005 | 0.167 | 0.005 | 4.35E-111 | 13 | ADIRF | Fibroblasts |
| RPS12 | 8.13E-66 | 0.811458 | 0.995 | 0.997 | 1.61E-61 | 13 | RPS12 | Fibroblasts |
| TAGLN | 1.12E-63 | 0.806975 | 0.246 | 0.028 | 2.22E-59 | 13 | TAGLN | Fibroblasts |
| GSN1 | 2.06E-18 | 0.805757 | 0.399 | 0.183 | 4.08E-14 | 13 | GSN | Fibroblasts |
| SERPINF11 | 0.000907 | 0.805091 | 0.325 | 0.274 | 1 | 13 | SERPINF1 | Fibroblasts |
| LXN | 1.42E-18 | 0.804018 | 0.182 | 0.048 | 2.82E-14 | 13 | LXN | Fibroblasts |
| IGFBP51 | 3.48E-08 | 0.79017 | 0.463 | 0.331 | 0.00069 | 13 | IGFBP5 | Fibroblasts |
| CRYAB | 4.00E-33 | 0.78922 | 0.266 | 0.059 | 7.91E-29 | 13 | CRYAB | Fibroblasts |
| IFITM31 | 2.71E-49 | 0.777091 | 0.562 | 0.182 | 5.36E-45 | 13 | IFITM3 | Fibroblasts |
| IGFBP6 | 2.82E-102 | 0.775775 | 0.315 | 0.028 | 5.58E-98 | 13 | IGFBP6 | Fibroblasts |
| IGFBP4 | 2.39E-23 | 0.755653 | 0.32 | 0.113 | 4.73E-19 | 13 | IGFBP4 | Fibroblasts |
| TPM2 | 2.40E-48 | 0.754256 | 0.31 | 0.06 | 4.76E-44 | 13 | TPM2 | Fibroblasts |
| FTH1 | 1.30E-45 | 0.745839 | 1 | 1 | 2.57E-41 | 13 | FTH1 | Fibroblasts |
| LYPD2 | 4.00E-104 | 0.731066 | 0.128 | 0.002 | 7.93E-100 | 13 | LYPD2 | Fibroblasts |
| TIMP3 | 1.75E-37 | 0.729922 | 0.374 | 0.104 | 3.47E-33 | 13 | TIMP3 | Fibroblasts |

ZFP36 TACSTD2 MEG3 IER3 FOS2 CST3 KRT5 FOSB ACTA2 JUNB2 COL5A2 ID1 LGALS31 SERPINE2 TIMP1 TPM11 SERF2 RARRES1 TPT1 COL6A2 KRT18 RPS26 PLAT RPL34 TGFBI FTL

| 1.41E-37 | 0.728581 | 0.424 | 0.135 | 2.80E-33 | 13 | ZFP36 | Fibroblasts |
| --- | --- | --- | --- | --- | --- | --- | --- |
| 1.83E-209 | 0.723975 | 0.182 | 0.001 | 3.63E-205 | 13 | TACSTD2 | Fibroblasts |
| 2.33E-178 | 0.718746 | 0.3 | 0.011 | 4.61E-174 | 13 | MEG3 | Fibroblasts |
| 5.27E-34 | 0.715026 | 0.384 | 0.122 | 1.04E-29 | 13 | IER3 | Fibroblasts |
| 9.92E-32 | 0.71287 | 0.882 | 0.579 | 1.96E-27 | 13 | FOS | Fibroblasts |
| 1.19E-09 | 0.712143 | 0.591 | 0.517 | 2.36E-05 | 13 | CST3 | Fibroblasts |
| 3.07E-233 | 0.709897 | 0.192 | 0 | 6.08E-229 | 13 | KRT5 | Fibroblasts |
| 3.49E-48 | 0.704315 | 0.458 | 0.125 | 6.92E-44 | 13 | FOSB | Fibroblasts |
| 1.19E-21 | 0.699296 | 0.167 | 0.036 | 2.36E-17 | 13 | ACTA2 | Fibroblasts |
| 5.99E-22 | 0.696233 | 0.793 | 0.551 | 1.19E-17 | 13 | JUNB | Fibroblasts |
| 1.64E-62 | 0.695256 | 0.286 | 0.04 | 3.25E-58 | 13 | COL5A2 | Fibroblasts |
| 8.85E-40 | 0.690233 | 0.483 | 0.162 | 1.75E-35 | 13 | ID1 | Fibroblasts |
| 1.03E-44 | 0.68869 | 0.365 | 0.083 | 2.04E-40 | 13 | LGALS3 | Fibroblasts |
| 0.000771 | 0.678822 | 0.197 | 0.132 | 1 | 13 | SERPINE2 | Fibroblasts |
| 6.95E-07 | 0.6778 | 0.389 | 0.296 | 0.013769 | 13 | TIMP1 | Fibroblasts |
| 2.96E-15 | 0.6671 | 0.507 | 0.33 | 5.86E-11 | 13 | TPM1 | Fibroblasts |
| 2.62E-48 | 0.665392 | 0.995 | 0.881 | 5.18E-44 | 13 | SERF2 | Fibroblasts |
| 3.20E-33 | 0.663095 | 0.167 | 0.025 | 6.34E-29 | 13 | RARRES1 | Fibroblasts |
| 4.47E-65 | 0.659278 | 1 | 0.999 | 8.86E-61 | 13 | TPT1 | Fibroblasts |
| 9.21E-12 | 0.651689 | 0.345 | 0.197 | 1.82E-07 | 13 | COL6A2 | Fibroblasts |
| 1.58E-59 | 0.650302 | 0.305 | 0.047 | 3.14E-55 | 13 | KRT18 | Fibroblasts |
| 6.39E-30 | 0.630038 | 0.946 | 0.768 | 1.26E-25 | 13 | RPS26 | Fibroblasts |
| 5.59E-12 | 0.629239 | 0.172 | 0.059 | 1.11E-07 | 13 | PLAT | Fibroblasts |
| 9.69E-45 | 0.617966 | 1 | 0.996 | 1.92E-40 | 13 | RPL34 | Fibroblasts |
| 5.86E-97 | 0.616909 | 0.202 | 0.011 | 1.16E-92 | 13 | TGFBI | Fibroblasts |
| 8.81E-10 | 0.616108 | 1 | 0.997 | 1.74E-05 | 13 | FTL | Fibroblasts |
| 1.13E-37 | 0.615301 | 0.227 | 0.039 | 2.24E-33 | 13 | RGS5 | Fibroblasts |
| 1.86E-09 | 0.61454 | 0.271 | 0.136 | 3.67E-05 | 13 | MT2A | Fibroblasts |
| 1.37E-28 | 0.598989 | 0.365 | 0.124 | 2.71E-24 | 13 | GADD45B | Fibroblasts |
| 6.54E-126 | 0.598746 | 0.123 | 0.001 | 1.29E-121 | 13 | SLPI | Fibroblasts |
| 2.58E-51 | 0.596758 | 0.502 | 0.153 | 5.10E-47 | 13 | LMNA | Fibroblasts |
| 8.19E-29 | 0.584347 | 0.365 | 0.117 | 1.62E-24 | 13 | GNG11 | Fibroblasts |
| 2.32E-46 | 0.58431 | 1 | 0.999 | 4.59E-42 | 13 | RPLP1 | Fibroblasts |
| 3.03E-16 | 0.581983 | 0.512 | 0.335 | 6.00E-12 | 13 | ANXA5 | Fibroblasts |
| 2.66E-61 | 0.571599 | 0.374 | 0.071 | 5.26E-57 | 13 | SERPINH1 | Fibroblasts |
| 5.91E-32 | 0.570134 | 0.31 | 0.082 | 1.17E-27 | 13 | CEBPD | Fibroblasts |
| 6.66E-35 | 0.560963 | 1 | 0.981 | 1.32E-30 | 13 | RPL12 | Fibroblasts |
| 1.70E-61 | 0.557788 | 0.187 | 0.016 | 3.37E-57 | 13 | TFPI2 | Fibroblasts |
| 4.54E-18 | 0.544476 | 0.626 | 0.393 | 8.99E-14 | 13 | MDK | Fibroblasts |
| 5.05E-17 | 0.541524 | 0.177 | 0.049 | 1.00E-12 | 13 | CLDN4 | Fibroblasts |
| 8.72E-40 | 0.540953 | 1 | 0.998 | 1.73E-35 | 13 | RPS14 | Fibroblasts |
| 1.15E-72 | 0.533324 | 0.207 | 0.016 | 2.28E-68 | 13 | NUPR1 | Fibroblasts |
| 5.10E-07 | 0.531496 | 0.256 | 0.149 | 0.010098 | 13 | PERP | Fibroblasts |
| 3.70E-38 | 0.525822 | 1 | 0.992 | 7.32E-34 | 13 | RPL28 | Fibroblasts |
| 3.06E-29 | 0.524524 | 0.488 | 0.209 | 6.07E-25 | 13 | CD99 | Fibroblasts |
| 2.13E-12 | 0.512749 | 0.547 | 0.432 | 4.23E-08 | 13 | CSTB | Fibroblasts |
| 1.02E-42 | 0.505381 | 1 | 1 | 2.01E-38 | 13 | EEF1A1 | Fibroblasts |
| 1.00E-31 | 0.502768 | 1 | 0.999 | 1.98E-27 | 13 | RPS23 | Fibroblasts |
| 1.14E-17 | 0.501732 | 0.576 | 0.378 | 2.25E-13 | 13 | MYL12A | Fibroblasts |
| 4.18E-40 | 0.501628 | 1 | 0.999 | 8.27E-36 | 13 | RPS27A | Fibroblasts |
| 1.57E-13 | 0.500912 | 0.493 | 0.313 | 3.10E-09 | 13 | NPC2 | Fibroblasts |
| 4.03E-65 | 1.104689 | 0.893 | 0.417 | 7.98E-61 | 14 | STMN2 | NRPCs/T2 |
| 2.31E-56 | 0.971571 | 0.777 | 0.337 | 4.57E-52 | 14 | PAX6 | NRPCs/T2 |
| 3.74E-55 | 0.944001 | 1 | 0.934 | 7.41E-51 | 14 | SOX4 | NRPCs/T2 |
| 2.55E-81 | 0.743069 | 0.254 | 0.022 | 5.06E-77 | 14 | PTF1A | NRPCs/T2 |
| 1.05E-40 | 0.705423 | 0.706 | 0.311 | 2.08E-36 | 14 | STMN4 | NRPCs/T2 |
| 9.37E-29 | 0.687157 | 0.873 | 0.626 | 1.85E-24 | 14 | SOX11 | NRPCs/T2 |
| 2.45E-45 | 0.655547 | 0.863 | 0.447 | 4.85E-41 | 14 | CD24 | NRPCs/T2 |
| 9.29E-40 | 0.631391 | 0.964 | 0.744 | 1.84E-35 | 14 | MLLT11 | NRPCs/T2 |

RGS5 MT2A GADD45B SLPI LMNA GNG11 RPLP1 ANXA5 SERPINH1 CEBPD RPL12 TFPI2 MDK2 CLDN4 RPS14 NUPR1 PERP RPL28 CD99 CSTB EEF1A1 RPS23 MYL12A1 RPS27A1 NPC22 STMN22 PAX61 SOX42 PTF1A STMN42 SOX112 CD242 MLLT112

RTN12 RORB2 NREP2 RUNX1T11 AP1S21 BASP12 APOC1 SCG33 TMEM701 OLAH PCLAF HSPB11 CHODL1 RBP41 TYMS1 C1QL1 MT2A1 RGS161 CPE3 ENPP2 PHOX2A1 CPLX31 NT5DC21 RAI14 CAMK2B ATP1B1 TMEM97 INSR1 CCNE2 AKAP92 GNG8 UQCC21 NEAT1 UBE2T WFDC2 BTG3 PRCD1 GGH

| 1.38E-38 | 0.587436 | 0.543 | 0.186 | 2.74E-34 | 14 | RTN1 | NRPCs/T2 |
| --- | --- | --- | --- | --- | --- | --- | --- |
| 2.42E-20 | 0.577688 | 0.741 | 0.515 | 4.78E-16 | 14 | RORB | NRPCs/T2 |
| 4.27E-35 | 0.554046 | 0.944 | 0.752 | 8.45E-31 | 14 | NREP | NRPCs/T2 |
| 2.64E-42 | 0.541345 | 0.508 | 0.157 | 5.23E-38 | 14 | RUNX1T1 | NRPCs/T2 |
| 3.20E-20 | 0.526177 | 0.812 | 0.607 | 6.33E-16 | 14 | AP1S2 | NRPCs/T2 |
| 2.69E-30 | 0.520357 | 0.975 | 0.784 | 5.32E-26 | 14 | BASP1 | NRPCs/T2 |
| 1.07E-96 | 1.219809 | 0.561 | 0.093 | 2.11E-92 | 15 | APOC1 | UPRCs |
| 3.58E-47 | 1.00181 | 0.884 | 0.506 | 7.09E-43 | 15 | SCG3 | UPRCs |
| 3.03E-25 | 0.910574 | 0.555 | 0.276 | 5.99E-21 | 15 | TMEM70 | UPRCs |
| 1.15E-70 | 0.904153 | 0.306 | 0.035 | 2.28E-66 | 15 | OLAH | UPRCs |
| 3.08E-39 | 0.779315 | 0.503 | 0.158 | 6.09E-35 | 15 | PCLAF | UPRCs |
| 2.92E-21 | 0.778827 | 0.52 | 0.292 | 5.79E-17 | 15 | HSPB11 | UPRCs |
| 7.63E-36 | 0.774735 | 0.457 | 0.136 | 1.51E-31 | 15 | CHODL | UPRCs |
| 1.54E-64 | 0.747652 | 0.595 | 0.142 | 3.05E-60 | 15 | RBP4 | UPRCs |
| 1.21E-21 | 0.733433 | 0.584 | 0.328 | 2.40E-17 | 15 | TYMS | UPRCs |
| 3.46E-38 | 0.720112 | 0.509 | 0.169 | 6.86E-34 | 15 | C1QL1 | UPRCs |
| 6.29E-38 | 0.718368 | 0.451 | 0.132 | 1.25E-33 | 15 | MT2A | UPRCs |
| 1.05E-12 | 0.713606 | 0.78 | 0.544 | 2.07E-08 | 15 | RGS16 | UPRCs |
| 4.87E-32 | 0.677778 | 0.965 | 0.666 | 9.64E-28 | 15 | CPE | UPRCs |
| 4.44E-54 | 0.674198 | 0.393 | 0.074 | 8.79E-50 | 15 | ENPP2 | UPRCs |
| 1.26E-64 | 0.66208 | 0.416 | 0.068 | 2.49E-60 | 15 | PHOX2A | UPRCs |
| 1.82E-40 | 0.653874 | 0.578 | 0.195 | 3.61E-36 | 15 | CPLX3 | UPRCs |
| 9.03E-24 | 0.652719 | 0.613 | 0.359 | 1.79E-19 | 15 | NT5DC2 | UPRCs |
| 9.73E-41 | 0.648545 | 0.578 | 0.206 | 1.93E-36 | 15 | RAI14 | UPRCs |
| 1.93E-33 | 0.645286 | 0.509 | 0.192 | 3.81E-29 | 15 | CAMK2B | UPRCs |
| 3.20E-21 | 0.6433 | 0.821 | 0.62 | 6.34E-17 | 15 | ATP1B1 | UPRCs |
| 1.50E-35 | 0.637892 | 0.566 | 0.228 | 2.96E-31 | 15 | TMEM97 | UPRCs |
| 7.29E-27 | 0.62826 | 0.526 | 0.236 | 1.44E-22 | 15 | INSR | UPRCs |
| 1.37E-55 | 0.625582 | 0.48 | 0.109 | 2.72E-51 | 15 | CCNE2 | UPRCs |
| 3.40E-28 | 0.617857 | 0.908 | 0.607 | 6.73E-24 | 15 | AKAP9 | UPRCs |
| 4.45E-74 | 0.614816 | 0.387 | 0.054 | 8.82E-70 | 15 | GNG8 | UPRCs |
| 2.37E-18 | 0.61358 | 0.59 | 0.384 | 4.70E-14 | 15 | UQCC2 | UPRCs |
| 3.95E-13 | 0.610564 | 0.884 | 0.712 | 7.83E-09 | 15 | NEAT1 | UPRCs |
| 1.00E-30 | 0.604196 | 0.584 | 0.249 | 1.98E-26 | 15 | UBE2T | UPRCs |
| 9.24E-31 | 0.603907 | 0.497 | 0.187 | 1.83E-26 | 15 | WFDC2 | UPRCs |
| 9.82E-19 | 0.6021 | 0.636 | 0.43 | 1.94E-14 | 15 | BTG3 | UPRCs |
| 1.39E-37 | 0.59996 | 0.549 | 0.184 | 2.74E-33 | 15 | PRCD | UPRCs |
| 1.75E-22 | 0.599254 | 0.543 | 0.297 | 3.47E-18 | 15 | GGH | UPRCs |
| 7.10E-25 | 0.596731 | 0.618 | 0.335 | 1.41E-20 | 15 | AHI1 | UPRCs |
| 2.20E-49 | 0.596117 | 0.503 | 0.134 | 4.36E-45 | 15 | MTHFD2 | UPRCs |
| 1.07E-30 | 0.592968 | 0.555 | 0.22 | 2.11E-26 | 15 | MIR7-3HG | UPRCs |
| 1.40E-36 | 0.583175 | 0.168 | 0.02 | 2.77E-32 | 15 | PPP1R27 | UPRCs |
| 1.08E-19 | 0.58126 | 0.913 | 0.782 | 2.13E-15 | 15 | H2AFY | UPRCs |
| 3.28E-48 | 0.579092 | 0.457 | 0.107 | 6.49E-44 | 15 | FAM111B | UPRCs |
| 3.06E-52 | 0.57673 | 0.497 | 0.115 | 6.06E-48 | 15 | RTBDN | UPRCs |
| 3.11E-32 | 0.575643 | 0.543 | 0.216 | 6.16E-28 | 15 | MFGE8 | UPRCs |
| 1.33E-25 | 0.573963 | 0.659 | 0.367 | 2.62E-21 | 15 | EEF1A2 | UPRCs |
| 1.58E-22 | 0.573463 | 0.52 | 0.245 | 3.13E-18 | 15 | OLFM1 | UPRCs |
| 2.79E-20 | 0.566304 | 0.572 | 0.339 | 5.53E-16 | 15 | TMEM106 | UPRCs |
| 1.76E-38 | 0.565197 | 0.491 | 0.15 | 3.49E-34 | 15 | CDKN2C | UPRCs |
| 1.37E-17 | 0.559478 | 0.451 | 0.196 | 2.72E-13 | 15 | RRAD | UPRCs |
| 5.82E-82 | 0.556893 | 0.393 | 0.05 | 1.15E-77 | 15 | CRIP1 | UPRCs |
| 1.17E-25 | 0.554973 | 0.931 | 0.683 | 2.32E-21 | 15 | DEK | UPRCs |
| 1.64E-19 | 0.549132 | 0.41 | 0.173 | 3.26E-15 | 15 | RARRES2 | UPRCs |
| 1.86E-30 | 0.536951 | 0.561 | 0.242 | 3.68E-26 | 15 | RFC4 | UPRCs |
| 2.62E-37 | 0.530783 | 0.457 | 0.132 | 5.19E-33 | 15 | CLSPN | UPRCs |
| 3.17E-19 | 0.529863 | 0.52 | 0.282 | 6.27E-15 | 15 | ACYP1 | UPRCs |
| 3.21E-43 | 0.520795 | 0.468 | 0.125 | 6.35E-39 | 15 | SH3KBP1 | UPRCs |
| 2.47E-30 | 0.519611 | 0.965 | 0.676 | 4.90E-26 | 15 | ENO2 | UPRCs |

AHI1 MTHFD2 MIR7-3HG PPP1R27 H2AFY FAM111B RTBDN1 MFGE8 EEF1A21 OLFM11 TMEM106 CDKN2C RRAD3 CRIP1 DEK1 RARRES2 RFC4 CLSPN ACYP1 SH3KBP1 ENO22

| FAM57B3 | 3.59E-28 | 0.519226 | 0.763 | 0.388 | 7.10E-24 | 15 | FAM57B | UPRCs |
| --- | --- | --- | --- | --- | --- | --- | --- | --- |
| PLEKHB12 | 3.75E-32 | 0.517646 | 0.624 | 0.247 | 7.42E-28 | 15 | PLEKHB1 | UPRCs |
| NAGK | 5.91E-29 | 0.511437 | 0.578 | 0.257 | 1.17E-24 | 15 | NAGK | UPRCs |
| PRKDC | 3.12E-14 | 0.50926 | 0.601 | 0.447 | 6.18E-10 | 15 | PRKDC | UPRCs |
| DYNLT1 | 5.82E-26 | 0.508964 | 0.919 | 0.743 | 1.15E-21 | 15 | DYNLT1 | UPRCs |
| GNG4 | 2.66E-30 | 0.506507 | 0.491 | 0.178 | 5.27E-26 | 15 | GNG4 | UPRCs |
| MYLK | 2.73E-46 | 0.503545 | 0.428 | 0.099 | 5.41E-42 | 15 | MYLK | UPRCs |
| TMEM541 | 8.50E-37 | 0.501976 | 0.474 | 0.143 | 1.68E-32 | 15 | TMEM54 | UPRCs |
| DLK1 | 1.16E-152 | 1.710884 | 0.628 | 0.064 | 2.30E-148 | 16 | DLK1 | Retinal ganglion cell progenitor |
| CRABP14 | 1.35E-57 | 1.48664 | 1 | 0.772 | 2.67E-53 | 16 | CRABP1 | Retinal ganglion cell progenitor |
| CDKN1C1 | 2.25E-63 | 1.449116 | 0.811 | 0.308 | 4.46E-59 | 16 | CDKN1C | Retinal ganglion cell progenitor |
| KLHL35 | 6.95E-153 | 1.247562 | 0.743 | 0.093 | 1.38E-148 | 16 | KLHL35 | Retinal ganglion cell progenitor |
| STMN23 | 3.09E-62 | 1.199107 | 0.953 | 0.42 | 6.12E-58 | 16 | STMN2 | Retinal ganglion cell progenitor |
| CNTN2 | 1.61E-210 | 1.195233 | 0.716 | 0.059 | 3.19E-206 | 16 | CNTN2 | Retinal ganglion cell progenitor |
| BASP13 | 6.46E-72 | 1.180421 | 1 | 0.785 | 1.28E-67 | 16 | BASP1 | Retinal ganglion cell progenitor |
| TUBA1A2 | 5.52E-69 | 1.16578 | 1 | 0.966 | 1.09E-64 | 16 | TUBA1A | Retinal ganglion cell progenitor |
| STMN43 | 1.23E-70 | 1.112562 | 0.899 | 0.309 | 2.44E-66 | 16 | STMN4 | Retinal ganglion cell progenitor |
| GADD45A1 | 4.42E-34 | 1.089869 | 0.736 | 0.383 | 8.75E-30 | 16 | GADD45A | Retinal ganglion cell progenitor |
| MAFB | 9.99E-58 | 1.020826 | 0.5 | 0.1 | 1.98E-53 | 16 | MAFB | Retinal ganglion cell progenitor |
| SOX113 | 3.68E-53 | 1.019994 | 0.966 | 0.625 | 7.28E-49 | 16 | SOX11 | Retinal ganglion cell progenitor |
| KCNQ1OT1 | 1.63E-29 | 0.972681 | 0.872 | 0.596 | 3.23E-25 | 16 | KCNQ1OT1 | Retinal ganglion cell progenitor |
| CD243 | 1.21E-62 | 0.906652 | 0.98 | 0.448 | 2.40E-58 | 16 | CD24 | Retinal ganglion cell progenitor |
| NHLH1 | 2.74E-112 | 0.868719 | 0.682 | 0.103 | 5.43E-108 | 16 | NHLH1 | Retinal ganglion cell progenitor |
| TUBB2B2 | 3.64E-52 | 0.867322 | 1 | 0.917 | 7.21E-48 | 16 | TUBB2B | Retinal ganglion cell progenitor |
| NNAT2 | 1.10E-31 | 0.86324 | 0.892 | 0.606 | 2.17E-27 | 16 | NNAT | Retinal ganglion cell progenitor |
| DNER2 | 1.97E-39 | 0.841486 | 0.723 | 0.3 | 3.89E-35 | 16 | DNER | Retinal ganglion cell progenitor |
| RBP12 | 3.61E-45 | 0.838017 | 1 | 0.967 | 7.16E-41 | 16 | RBP1 | Retinal ganglion cell progenitor |
| RND32 | 5.64E-58 | 0.823934 | 0.709 | 0.194 | 1.12E-53 | 16 | RND3 | Retinal ganglion cell progenitor |
| SOX43 | 5.30E-45 | 0.821017 | 0.993 | 0.935 | 1.05E-40 | 16 | SOX4 | Retinal ganglion cell progenitor |
| DCX2 | 1.40E-64 | 0.800671 | 0.838 | 0.254 | 2.76E-60 | 16 | DCX | Retinal ganglion cell progenitor |
| MIAT1 | 7.24E-48 | 0.795693 | 0.858 | 0.352 | 1.43E-43 | 16 | MIAT | Retinal ganglion cell progenitor |
| EEF1A22 | 2.79E-47 | 0.792132 | 0.858 | 0.363 | 5.52E-43 | 16 | EEF1A2 | Retinal ganglion cell progenitor |
| MLLT113 | 9.54E-55 | 0.792058 | 1 | 0.745 | 1.89E-50 | 16 | MLLT11 | Retinal ganglion cell progenitor |
| ROBO3 | 6.54E-112 | 0.732727 | 0.466 | 0.045 | 1.30E-107 | 16 | ROBO3 | Retinal ganglion cell progenitor |
| ARL4D1 | 2.60E-17 | 0.729796 | 0.696 | 0.411 | 5.15E-13 | 16 | ARL4D | Retinal ganglion cell progenitor |
| GAP431 | 1.26E-42 | 0.714853 | 0.743 | 0.251 | 2.50E-38 | 16 | GAP43 | Retinal ganglion cell progenitor |
| INSM12 | 6.53E-40 | 0.707367 | 0.764 | 0.32 | 1.29E-35 | 16 | INSM1 | Retinal ganglion cell progenitor |
| ELAVL2 | 1.02E-57 | 0.66985 | 0.655 | 0.166 | 2.03E-53 | 16 | ELAVL2 | Retinal ganglion cell progenitor |
| MAP1B1 | 2.24E-40 | 0.658946 | 1 | 0.902 | 4.43E-36 | 16 | MAP1B | Retinal ganglion cell progenitor |
| KIF5C2 | 1.81E-40 | 0.649405 | 0.784 | 0.332 | 3.58E-36 | 16 | KIF5C | Retinal ganglion cell progenitor |
| CASP3 | 3.34E-29 | 0.629485 | 0.649 | 0.287 | 6.61E-25 | 16 | CASP3 | Retinal ganglion cell progenitor |
| TMSB102 | 3.68E-30 | 0.623485 | 1 | 0.943 | 7.29E-26 | 16 | TMSB10 | Retinal ganglion cell progenitor |
| DDAH2 | 1.24E-36 | 0.621862 | 0.899 | 0.506 | 2.46E-32 | 16 | DDAH2 | Retinal ganglion cell progenitor |
| TAGLN32 | 5.28E-38 | 0.618222 | 0.892 | 0.431 | 1.05E-33 | 16 | TAGLN3 | Retinal ganglion cell progenitor |
| UCHL11 | 1.24E-40 | 0.602499 | 0.939 | 0.521 | 2.45E-36 | 16 | UCHL1 | Retinal ganglion cell progenitor |
| GNG32 | 3.47E-58 | 0.601367 | 0.628 | 0.15 | 6.86E-54 | 16 | GNG3 | Retinal ganglion cell progenitor |
| ANK31 | 2.98E-33 | 0.594178 | 0.628 | 0.239 | 5.91E-29 | 16 | ANK3 | Retinal ganglion cell progenitor |
| CABP7 | 8.64E-44 | 0.588391 | 0.547 | 0.142 | 1.71E-39 | 16 | CABP7 | Retinal ganglion cell progenitor |
| CRYBG31 | 8.14E-35 | 0.578153 | 0.689 | 0.259 | 1.61E-30 | 16 | CRYBG3 | Retinal ganglion cell progenitor |
| HOXB22 | 1.50E-13 | 0.574546 | 0.595 | 0.342 | 2.97E-09 | 16 | HOXB2 | Retinal ganglion cell progenitor |
| ELAVL41 | 2.34E-51 | 0.574155 | 0.412 | 0.073 | 4.64E-47 | 16 | ELAVL4 | Retinal ganglion cell progenitor |
| JPT12 | 5.19E-34 | 0.571843 | 0.946 | 0.665 | 1.03E-29 | 16 | JPT1 | Retinal ganglion cell progenitor |
| IER5L | 1.09E-25 | 0.560605 | 0.561 | 0.223 | 2.15E-21 | 16 | IER5L | Retinal ganglion cell progenitor |
| TMSB4X | 7.58E-14 | 0.559313 | 1 | 0.982 | 1.50E-09 | 16 | TMSB4X | Retinal ganglion cell progenitor |
| TCEAL71 | 1.24E-24 | 0.556077 | 0.899 | 0.695 | 2.46E-20 | 16 | TCEAL7 | Retinal ganglion cell progenitor |
| ELAVL31 | 1.58E-51 | 0.555426 | 0.73 | 0.212 | 3.13E-47 | 16 | ELAVL3 | Retinal ganglion cell progenitor |
| TMEM176 | 3.31E-36 | 0.54904 | 0.561 | 0.171 | 6.55E-32 | 16 | TMEM176 | Retinal ganglion cell progenitor |
| JUND | 2.03E-26 | 0.546381 | 0.993 | 0.929 | 4.03E-22 | 16 | JUND | Retinal ganglion cell progenitor |
| PPP1R14B | 1.52E-29 | 0.542207 | 0.831 | 0.469 | 3.01E-25 | 16 | PPP1R14B | Retinal ganglion cell progenitor |

| 2.06E-19 | 0.529659 | 0.926 | 0.795 | 4.07E-15 | 16 CALM1 | Retinal ganglion cell progenitor |
| --- | --- | --- | --- | --- | --- | --- |
| 4.70E-29 | 0.528302 | 0.73 | 0.339 | 9.31E-25 | 16 SRGAP3 | Retinal ganglion cell progenitor |
| 1.63E-25 | 0.523105 | 0.669 | 0.292 | 3.24E-21 | 16 VEGFA | Retinal ganglion cell progenitor |
| 6.19E-69 | 0.514392 | 0.608 | 0.123 | 1.23E-64 | 16 ADGRG1 | Retinal ganglion cell progenitor |
| 3.70E-27 | 0.512848 | 0.703 | 0.335 | 7.33E-23 | 16 FNBP1L | Retinal ganglion cell progenitor |
| 7.38E-41 | 0.511906 | 0.561 | 0.152 | 1.46E-36 | 16 CHRNA3 | Retinal ganglion cell progenitor |
| 6.97E-33 | 0.507318 | 0.486 | 0.14 | 1.38E-28 | 16 TMEM176 | Retinal ganglion cell progenitor |
| 5.00E-27 | 0.507123 | 0.669 | 0.303 | 9.89E-23 | 16 MAP6 | Retinal ganglion cell progenitor |
| 1.89E-38 | 0.503509 | 0.405 | 0.092 | 3.75E-34 | 16 LMO1 | Retinal ganglion cell progenitor |
| 6.29E-39 | 1.428686 | 0.562 | 0.141 | 1.25E-34 | 17 TF | late RPCs |
| 6.02E-52 | 1.326138 | 0.982 | 0.532 | 1.19E-47 | 17 TRH | late RPCs |
| 4.86E-50 | 1.159503 | 0.973 | 0.473 | 9.63E-46 | 17 DKK3 | late RPCs |
| 2.68E-46 | 1.081234 | 1 | 0.955 | 5.31E-42 | 17 RTN4 | late RPCs |
| 8.66E-48 | 1.077175 | 0.991 | 0.521 | 1.71E-43 | 17 SPP1 | late RPCs |
| 5.13E-50 | 1.066988 | 0.929 | 0.366 | 1.02E-45 | 17 TTYH1 | late RPCs |
| 1.73E-38 | 1.055901 | 1 | 0.751 | 3.42E-34 | 17 VIM | late RPCs |
| 3.12E-36 | 1.053854 | 0.982 | 0.83 | 6.18E-32 | 17 IGFBP2 | late RPCs |
| 1.15E-46 | 0.998053 | 0.982 | 0.489 | 2.29E-42 | 17 SFRP2 | late RPCs |
| 1.17E-35 | 0.977575 | 0.83 | 0.373 | 2.32E-31 | 17 SLC2A1 | late RPCs |
| 7.97E-55 | 0.907124 | 0.848 | 0.246 | 1.58E-50 | 17 CD9 | late RPCs |
| 2.17E-42 | 0.878244 | 1 | 0.612 | 4.30E-38 | 17 CLU | late RPCs |
| 2.54E-20 | 0.863743 | 0.866 | 0.584 | 5.03E-16 | 17 FOS | late RPCs |
| 1.15E-35 | 0.84689 | 0.866 | 0.382 | 2.29E-31 | 17 SLC16A1 | late RPCs |
| 5.20E-33 | 0.840264 | 1 | 0.687 | 1.03E-28 | 17 FABP7 | late RPCs |
| 2.05E-30 | 0.831367 | 0.795 | 0.312 | 4.06E-26 | 17 CCND1 | late RPCs |
| 6.29E-61 | 0.830961 | 0.759 | 0.166 | 1.25E-56 | 17 WIF1 | late RPCs |
| 4.40E-31 | 0.814871 | 0.875 | 0.473 | 8.71E-27 | 17 PLEKHA1 | late RPCs |
| 1.04E-36 | 0.79721 | 0.866 | 0.336 | 2.06E-32 | 17 SOX2 | late RPCs |
| 1.72E-32 | 0.753532 | 0.973 | 0.722 | 3.41E-28 | 17 GPM6B | late RPCs |
| 1.11E-10 | 0.74835 | 0.866 | 0.738 | 2.19E-06 | 17 JUN | late RPCs |
| 4.39E-32 | 0.722796 | 0.812 | 0.319 | 8.70E-28 | 17 ZFP36L1 | late RPCs |
| 7.94E-32 | 0.695221 | 0.661 | 0.226 | 1.57E-27 | 17 SRPRB | late RPCs |
| 1.59E-24 | 0.673813 | 1 | 0.97 | 3.15E-20 | 17 ENO1 | late RPCs |
| 8.03E-22 | 0.666076 | 0.92 | 0.603 | 1.59E-17 | 17 RAX | late RPCs |
| 1.36E-10 | 0.662617 | 0.5 | 0.272 | 2.69E-06 | 17 QDPR | late RPCs |
| 1.87E-26 | 0.661495 | 0.723 | 0.276 | 3.71E-22 | 17 HES1 | late RPCs |
| 2.10E-18 | 0.64722 | 0.929 | 0.744 | 4.16E-14 | 17 IER2 | late RPCs |
| 9.18E-30 | 0.643077 | 0.929 | 0.618 | 1.82E-25 | 17 CADM1 | late RPCs |
| 2.35E-20 | 0.642657 | 0.848 | 0.483 | 4.66E-16 | 17 EGR1 | late RPCs |
| 1.08E-40 | 0.625602 | 0.545 | 0.125 | 2.15E-36 | 17 PRSS35 | late RPCs |
| 9.50E-29 | 0.618708 | 0.848 | 0.353 | 1.88E-24 | 17 PRSS23 | late RPCs |
| 5.58E-26 | 0.602266 | 0.473 | 0.135 | 1.10E-21 | 17 PLP1 | late RPCs |
| 3.80E-19 | 0.599268 | 0.857 | 0.544 | 7.53E-15 | 17 RGS16 | late RPCs |
| 7.40E-26 | 0.589628 | 0.777 | 0.338 | 1.47E-21 | 17 ZFP36L2 | late RPCs |
| 1.19E-22 | 0.577522 | 0.705 | 0.305 | 2.36E-18 | 17 LINC00461 | late RPCs |
| 2.24E-11 | 0.569643 | 0.786 | 0.625 | 4.43E-07 | 17 HES4 | late RPCs |
| 1.29E-17 | 0.569054 | 0.795 | 0.455 | 2.55E-13 | 17 SAT1 | late RPCs |
| 2.52E-17 | 0.566053 | 0.589 | 0.267 | 4.98E-13 | 17 HTRA1 | late RPCs |
| 1.47E-17 | 0.558816 | 0.777 | 0.448 | 2.92E-13 | 17 FABP5 | late RPCs |
| 3.03E-24 | 0.555526 | 0.741 | 0.336 | 6.01E-20 | 17 CDC42EP4 | late RPCs |
| 3.47E-21 | 0.544393 | 0.714 | 0.342 | 6.87E-17 | 17 CNTLN | late RPCs |
| 1.78E-23 | 0.522935 | 0.661 | 0.265 | 3.53E-19 | 17 AGL | late RPCs |
| 7.31E-19 | 0.513215 | 0.661 | 0.268 | 1.45E-14 | 17 GPC3 | late RPCs |
| 1.33E-13 | 0.508691 | 0.67 | 0.397 | 2.64E-09 | 17 RHOB | late RPCs |
| 7.07E-11 | 0.505985 | 0.768 | 0.556 | 1.40E-06 | 17 JUNB | late RPCs |
| .02E-270 | 2.697045 | 0.784 | 0.038 | 1.19E-265 | 18 SPARCL1 | Astrocytes |
| .04E-138 | 2.390347 | 0.657 | 0.056 | 2.07E-134 | 18 CRYAB | Astrocytes |
| .50E-130 | 1.951979 | 0.559 | 0.042 | 1.68E-125 | 18 NMB | Astrocytes |
| 2.13E-74 | 1.874997 | 0.784 | 0.169 | 4.23E-70 | 18 C1QL1 | Astrocytes |

| CALM11 |
| --- |
| SRGAP3 |
| VEGFA |
| ADGRG1 |
| FNBP1L2 |
| CHRNA31 |
| TMEM176 |
| MAP6 |
| LMO1 |
| TF2 |
| TRH2 |
| DKK32 |
| RTN42 |
| SPP13 |
| TTYH13 |
| VIM3 |
| IGFBP21 |
| SFRP23 |
| SLC2A12 |
| CD91 |
| CLU2 |
| FOS3 |
| SLC16A11 |
| FABP73 |
| CCND13 |
| WIF11 |
| PLEKHA12 |
| SOX22 |
| GPM6B2 |
| Jun-01 |
| ZFP36L12 |
| SRPRB1 |
| ENO11 |
| RAX3 |
| QDPR1 |
| HES13 |
| IER22 |
| CADM1 |
| EGR12 |
| PRSS351 |
| PRSS232 |
| PLP12 |
| RGS162 |
| ZFP36L21 |
| LINC00461 |
| HES41 |
| SAT11 |
| HTRA1 |
| FABP5 |
| CDC42EP4 |
| CNTLN |
| AGL1 |
| GPC32 |
| RHOB1 |
| JUNB3 |
| SPARCL1 6 |
| CRYAB1 1 |
| NMB 8 |
| C1QL11 |

| ID31 | 1.75E-45 | 1.863835 | 0.706 | 0.217 | 3.47E-41 | 18 | ID3 | Astrocytes |
| --- | --- | --- | --- | --- | --- | --- | --- | --- |
| HOTAIRM1 | 2.85E-198 | 1.69908 | 0.657 | 0.037 | 5.64E-194 | 18 | HOTAIRM1 | Astrocytes |
| PTN1 | 3.89E-49 | 1.68249 | 0.853 | 0.293 | 7.69E-45 | 18 | PTN | Astrocytes |
| SERPINI1 | 4.76E-126 | 1.668633 | 0.569 | 0.046 | 9.42E-122 | 18 | SERPINI1 | Astrocytes |
| POSTN | 0 | 1.585405 | 0.52 | 0.009 | 0 | 18 | POSTN | Astrocytes |
| PDLIM3 | 4.84E-95 | 1.503327 | 0.735 | 0.115 | 9.59E-91 | 18 | PDLIM3 | Astrocytes |
| MGST1 | 1.10E-204 | 1.502703 | 0.725 | 0.045 | 2.18E-200 | 18 | MGST1 | Astrocytes |
| APOE1 | 1.19E-34 | 1.491761 | 0.647 | 0.203 | 2.35E-30 | 18 | APOE | Astrocytes |
| AGT | 2.37E-182 | 1.487375 | 0.578 | 0.03 | 4.70E-178 | 18 | AGT | Astrocytes |
| PTGDS2 | 7.68E-99 | 1.449717 | 0.706 | 0.094 | 1.52E-94 | 18 | PTGDS | Astrocytes |
| HOPX | 5.06E-78 | 1.411692 | 0.539 | 0.066 | 1.00E-73 | 18 | HOPX | Astrocytes |
| S100A101 | 1.06E-104 | 1.396764 | 0.578 | 0.056 | 2.09E-100 | 18 | S100A10 | Astrocytes |
| SAT12 | 5.43E-40 | 1.381186 | 0.882 | 0.454 | 1.08E-35 | 18 | SAT1 | Astrocytes |
| IGFBP72 | 1.95E-31 | 1.332504 | 0.471 | 0.111 | 3.86E-27 | 18 | IGFBP7 | Astrocytes |
| IGFBP52 | 1.03E-27 | 1.302534 | 0.745 | 0.328 | 2.05E-23 | 18 | IGFBP5 | Astrocytes |
| GOLIM4 | 4.38E-40 | 1.287658 | 0.696 | 0.253 | 8.67E-36 | 18 | GOLIM4 | Astrocytes |
| CLU3 | 1.92E-31 | 1.281175 | 0.971 | 0.613 | 3.79E-27 | 18 | CLU | Astrocytes |
| CST31 | 1.73E-12 | 1.268812 | 0.716 | 0.516 | 3.43E-08 | 18 | CST3 | Astrocytes |
| CEBPD1 | 6.24E-95 | 1.236494 | 0.637 | 0.08 | 1.24E-90 | 18 | CEBPD | Astrocytes |
| DHRS31 | 9.23E-49 | 1.209764 | 0.794 | 0.263 | 1.83E-44 | 18 | DHRS3 | Astrocytes |
| METRN2 | 8.94E-39 | 1.188938 | 0.922 | 0.468 | 1.77E-34 | 18 | METRN | Astrocytes |
| C1orf61 | 5.89E-93 | 1.175424 | 0.451 | 0.038 | 1.17E-88 | 18 | C1orf61 | Astrocytes |
| VIM4 | 5.88E-30 | 1.16876 | 0.99 | 0.751 | 1.16E-25 | 18 | VIM | Astrocytes |
| CD991 | 1.68E-63 | 1.165204 | 0.794 | 0.209 | 3.32E-59 | 18 | CD99 | Astrocytes |
| HOXB91 | 1.54E-205 | 1.050795 | 0.735 | 0.043 | 3.06E-201 | 18 | HOXB9 | Astrocytes |
| BAALC | 2.52E-38 | 1.048039 | 0.657 | 0.211 | 4.99E-34 | 18 | BAALC | Astrocytes |
| GFAP | 2.48E-227 | 1.047431 | 0.324 | 0.005 | 4.91E-223 | 18 | GFAP | Astrocytes |
| CD92 | 5.98E-45 | 1.044058 | 0.784 | 0.248 | 1.18E-40 | 18 | CD9 | Astrocytes |
| TMOD1 | 2.93E-205 | 1.042819 | 0.52 | 0.02 | 5.80E-201 | 18 | TMOD1 | Astrocytes |
| LAPTM4B2 | 1.13E-22 | 1.015959 | 0.892 | 0.646 | 2.24E-18 | 18 | LAPTM4B | Astrocytes |
| ANGPTL4 | 2.11E-120 | 1.015408 | 0.539 | 0.042 | 4.18E-116 | 18 | ANGPTL4 | Astrocytes |
| S100B | 6.54E-215 | 1.013366 | 0.549 | 0.021 | 1.29E-210 | 18 | S100B | Astrocytes |
| NFIA | 2.25E-90 | 0.989939 | 0.676 | 0.096 | 4.45E-86 | 18 | NFIA | Astrocytes |
| SKAP2 | 3.25E-193 | 0.977414 | 0.588 | 0.029 | 6.43E-189 | 18 | SKAP2 | Astrocytes |
| PSAP | 5.00E-34 | 0.974027 | 0.794 | 0.405 | 9.90E-30 | 18 | PSAP | Astrocytes |
| IFITM32 | 7.81E-28 | 0.968416 | 0.578 | 0.188 | 1.55E-23 | 18 | IFITM3 | Astrocytes |
| ITM2A | 3.17E-93 | 0.966513 | 0.431 | 0.034 | 6.27E-89 | 18 | ITM2A | Astrocytes |
| HOXB71 | 6.91E-85 | 0.96283 | 0.716 | 0.112 | 1.37E-80 | 18 | HOXB7 | Astrocytes |
| CFI1 | 3.53E-70 | 0.941718 | 0.618 | 0.099 | 6.98E-66 | 18 | CFI | Astrocytes |
| CYP26A12 | 5.53E-16 | 0.940474 | 0.549 | 0.225 | 1.10E-11 | 18 | CYP26A1 | Astrocytes |
| ANXA21 | 1.60E-31 | 0.935589 | 0.578 | 0.178 | 3.17E-27 | 18 | ANXA2 | Astrocytes |
| LGALS32 | 5.80E-65 | 0.927767 | 0.569 | 0.084 | 1.15E-60 | 18 | LGALS3 | Astrocytes |
| IGFBP22 | 7.77E-22 | 0.926341 | 0.99 | 0.83 | 1.54E-17 | 18 | IGFBP2 | Astrocytes |
| SERPINE21 | 1.18E-33 | 0.887457 | 0.51 | 0.128 | 2.33E-29 | 18 | SERPINE2 | Astrocytes |
| FAM181B | 0 | 0.87716 | 0.51 | 0.009 | 0 | 18 | FAM181B | Astrocytes |
| A2M | 1.70E-128 | 0.876568 | 0.382 | 0.017 | 3.37E-124 | 18 | A2M | Astrocytes |
| B2M1 | 1.30E-29 | 0.868031 | 0.902 | 0.573 | 2.57E-25 | 18 | B2M | Astrocytes |
| CNN31 | 3.53E-24 | 0.866358 | 0.794 | 0.474 | 6.99E-20 | 18 | CNN3 | Astrocytes |
| TKT | 9.74E-26 | 0.857951 | 0.824 | 0.506 | 1.93E-21 | 18 | TKT | Astrocytes |
| SCRG1 | 2.50E-47 | 0.843134 | 0.373 | 0.052 | 4.95E-43 | 18 | SCRG1 | Astrocytes |
| TIMP11 | 2.59E-39 | 0.838749 | 0.775 | 0.291 | 5.13E-35 | 18 | TIMP1 | Astrocytes |
| HOXB82 | 1.94E-56 | 0.835091 | 0.716 | 0.151 | 3.84E-52 | 18 | HOXB8 | Astrocytes |
| RGMA | 3.76E-165 | 0.829395 | 0.49 | 0.023 | 7.44E-161 | 18 | RGMA | Astrocytes |
| ID41 | 1.19E-20 | 0.821818 | 0.676 | 0.321 | 2.36E-16 | 18 | ID4 | Astrocytes |
| ITM2B1 | 1.60E-31 | 0.803886 | 0.98 | 0.885 | 3.17E-27 | 18 | ITM2B | Astrocytes |
| PRCP | 2.11E-50 | 0.801788 | 0.608 | 0.136 | 4.18E-46 | 18 | PRCP | Astrocytes |
| PLTP1 | 7.26E-41 | 0.78998 | 0.716 | 0.217 | 1.44E-36 | 18 | PLTP | Astrocytes |
| CHPF1 | 2.75E-36 | 0.775806 | 0.647 | 0.197 | 5.44E-32 | 18 | CHPF | Astrocytes |
| CDO1 | 8.73E-33 | 0.772455 | 0.588 | 0.182 | 1.73E-28 | 18 | CDO1 | Astrocytes |

| LMO4 | 4.12E-19 | 0.769344 | 0.608 | 0.263 | 8.15E-15 | 18 | LMO4 | Astrocytes |
| --- | --- | --- | --- | --- | --- | --- | --- | --- |
| SFRP4 | 7.80E-184 | 0.76465 | 0.422 | 0.014 | 1.54E-179 | 18 | SFRP4 | Astrocytes |
| ID11 | 6.29E-19 | 0.761805 | 0.48 | 0.168 | 1.25E-14 | 18 | ID1 | Astrocytes |
| GPC33 | 8.84E-29 | 0.745246 | 0.755 | 0.267 | 1.75E-24 | 18 | GPC3 | Astrocytes |
| NDUFA4L2 | 6.24E-19 | 0.735853 | 0.235 | 0.047 | 1.23E-14 | 18 | NDUFA4L2 | Astrocytes |
| RPS27L | 1.26E-17 | 0.726132 | 0.784 | 0.55 | 2.50E-13 | 18 | RPS27L | Astrocytes |
| IGFBP41 | 7.87E-45 | 0.721024 | 0.539 | 0.113 | 1.56E-40 | 18 | IGFBP4 | Astrocytes |
| HILPDA | 7.04E-19 | 0.71981 | 0.52 | 0.211 | 1.39E-14 | 18 | HILPDA | Astrocytes |
| SOX23 | 3.93E-23 | 0.711443 | 0.765 | 0.339 | 7.78E-19 | 18 | SOX2 | Astrocytes |
| F3 | 1.08E-80 | 0.710393 | 0.441 | 0.042 | 2.14E-76 | 18 | F3 | Astrocytes |
| BRINP1 | 2.52E-87 | 0.700767 | 0.529 | 0.056 | 5.00E-83 | 18 | BRINP1 | Astrocytes |
| NLRP11 | 1.34E-10 | 0.676946 | 0.657 | 0.448 | 2.65E-06 | 18 | NLRP1 | Astrocytes |
| CD81 | 5.08E-28 | 0.675747 | 0.99 | 0.835 | 1.00E-23 | 18 | CD81 | Astrocytes |
| LINC02381 | 6.98E-76 | 0.671124 | 0.618 | 0.088 | 1.38E-71 | 18 | LINC02381 | Astrocytes |
| SELENOM | 2.63E-28 | 0.66866 | 0.578 | 0.197 | 5.20E-24 | 18 | SELENOM | Astrocytes |
| MIR99AHG | 3.95E-65 | 0.667372 | 0.51 | 0.07 | 7.82E-61 | 18 | MIR99AHG | Astrocytes |
| LY6H1 | 1.38E-29 | 0.665974 | 0.451 | 0.109 | 2.72E-25 | 18 | LY6H | Astrocytes |
| GPM6B3 | 4.30E-20 | 0.663992 | 0.971 | 0.722 | 8.51E-16 | 18 | GPM6B | Astrocytes |
| PLA2G161 | 3.22E-24 | 0.660803 | 0.647 | 0.259 | 6.37E-20 | 18 | PLA2G16 | Astrocytes |
| RARRES21 | 4.29E-24 | 0.654038 | 0.529 | 0.174 | 8.49E-20 | 18 | RARRES2 | Astrocytes |
| VCAN | 3.29E-23 | 0.64732 | 0.402 | 0.105 | 6.51E-19 | 18 | VCAN | Astrocytes |
| TSC22D4 | 2.81E-51 | 0.645387 | 0.618 | 0.129 | 5.56E-47 | 18 | TSC22D4 | Astrocytes |
| LGALS11 | 3.85E-100 | 0.632771 | 0.5 | 0.041 | 7.63E-96 | 18 | LGALS1 | Astrocytes |
| FXYD61 | 2.04E-13 | 0.631671 | 0.676 | 0.414 | 4.04E-09 | 18 | FXYD6 | Astrocytes |
| ARL4A | 1.61E-20 | 0.630091 | 0.539 | 0.199 | 3.19E-16 | 18 | ARL4A | Astrocytes |
| VEGFA1 | 2.34E-08 | 0.628367 | 0.49 | 0.298 | 0.000464 | 18 | VEGFA | Astrocytes |
| SRI | 1.25E-19 | 0.626547 | 0.765 | 0.481 | 2.48E-15 | 18 | SRI | Astrocytes |
| ZIC1 | 8.39E-38 | 0.623409 | 0.52 | 0.12 | 1.66E-33 | 18 | ZIC1 | Astrocytes |
| NTRK2 | 3.78E-48 | 0.622456 | 0.373 | 0.05 | 7.47E-44 | 18 | NTRK2 | Astrocytes |
| GNG111 | 7.57E-35 | 0.617523 | 0.52 | 0.119 | 1.50E-30 | 18 | GNG11 | Astrocytes |
| CYP26B11 | 1.29E-31 | 0.616927 | 0.5 | 0.118 | 2.55E-27 | 18 | CYP26B1 | Astrocytes |
| SPARC1 | 6.66E-27 | 0.615688 | 0.706 | 0.278 | 1.32E-22 | 18 | SPARC | Astrocytes |
| TUBB2A2 | 3.00E-10 | 0.61274 | 0.647 | 0.417 | 5.94E-06 | 18 | TUBB2A | Astrocytes |
| DCLK1 | 2.95E-25 | 0.612381 | 0.627 | 0.241 | 5.85E-21 | 18 | DCLK1 | Astrocytes |
| CD63 | 3.50E-24 | 0.609789 | 1 | 0.909 | 6.94E-20 | 18 | CD63 | Astrocytes |
| HOXB-AS3 | 3.44E-64 | 0.60374 | 0.382 | 0.038 | 6.82E-60 | 18 | HOXB-AS3 | Astrocytes |
| FRZB1 | 0.000529 | 0.598014 | 0.206 | 0.11 | 1 | 18 | FRZB | Astrocytes |
| ID2 | 4.75E-19 | 0.589709 | 0.48 | 0.165 | 9.41E-15 | 18 | ID2 | Astrocytes |
| HSPB12 | 3.23E-35 | 0.58563 | 0.686 | 0.217 | 6.40E-31 | 18 | HSPB1 | Astrocytes |
| CA14 | 6.32E-22 | 0.585429 | 0.451 | 0.136 | 1.25E-17 | 18 | CA14 | Astrocytes |
| CHL1 | 4.82E-29 | 0.582732 | 0.569 | 0.174 | 9.54E-25 | 18 | CHL1 | Astrocytes |
| ATP6AP2 | 3.72E-21 | 0.582398 | 0.902 | 0.665 | 7.36E-17 | 18 | ATP6AP2 | Astrocytes |
| GSN2 | 7.81E-21 | 0.579358 | 0.529 | 0.184 | 1.55E-16 | 18 | GSN | Astrocytes |
| G0S2 | 2.52E-15 | 0.578611 | 0.304 | 0.087 | 4.98E-11 | 18 | G0S2 | Astrocytes |
| PON2 | 1.57E-24 | 0.567659 | 0.608 | 0.217 | 3.11E-20 | 18 | PON2 | Astrocytes |
| RPL411 | 1.48E-18 | 0.562002 | 1 | 0.995 | 2.94E-14 | 18 | RPL41 | Astrocytes |
| NOVA11 | 8.69E-19 | 0.560057 | 0.598 | 0.258 | 1.72E-14 | 18 | NOVA1 | Astrocytes |
| ATP6V0E1 | 1.63E-24 | 0.558055 | 0.716 | 0.334 | 3.22E-20 | 18 | ATP6V0E1 | Astrocytes |
| C1orf54 | 3.62E-41 | 0.55659 | 0.549 | 0.124 | 7.17E-37 | 18 | C1orf54 | Astrocytes |
| GAS6 | 1.14E-27 | 0.555179 | 0.559 | 0.176 | 2.25E-23 | 18 | GAS6 | Astrocytes |
| JUNB4 | 1.65E-10 | 0.553924 | 0.765 | 0.556 | 3.27E-06 | 18 | JUNB | Astrocytes |
| PPP1R17 | 1.23E-102 | 0.551672 | 0.294 | 0.013 | 2.43E-98 | 18 | PPP1R17 | Astrocytes |
| ATP1A2 | 1.11E-151 | 0.550948 | 0.451 | 0.021 | 2.20E-147 | 18 | ATP1A2 | Astrocytes |
| CP1 | 3.44E-14 | 0.544837 | 0.304 | 0.094 | 6.81E-10 | 18 | CP | Astrocytes |
| HSPA5 | 3.47E-15 | 0.541937 | 0.922 | 0.681 | 6.88E-11 | 18 | HSPA5 | Astrocytes |
| SNX3 | 7.04E-16 | 0.536405 | 0.765 | 0.56 | 1.39E-11 | 18 | SNX3 | Astrocytes |
| ATP1B21 | 5.33E-14 | 0.532564 | 0.686 | 0.407 | 1.05E-09 | 18 | ATP1B2 | Astrocytes |
| MT-ND3 | 9.55E-14 | 0.530659 | 1 | 0.986 | 1.89E-09 | 18 | MT-ND3 | Astrocytes |
| ZFP36L22 | 3.06E-14 | 0.527764 | 0.676 | 0.341 | 6.05E-10 | 18 | ZFP36L2 | Astrocytes |

ELN EMP3 LTBP3 EDNRB FEZ11 SOCS3 BEX3 HOXA5 HSP90B1 TNFRSF1 NUPR11 HOXA3 ZFP361 GATM FTH11 WLS TOP2A2 CENPF1 HMGB22 PTTG1 UBE2C1 CCNB1 CCNB2 NUSAP1 CKS2 CDK1 MKI67 TPX2 KPNA2 SMC4 TYMS2 ASPM TUBA1B1 UBE2S1 MAD2L1 CDKN3 CDC20 PBK UBE2T1 H2AFX NUF2 PCLAF1 NEK2 CKS1B CCNA2 BIRC5 ARL6IP1 H2AFZ1 KIF20B SGO2 CKAP21 FBLN11 TUBA1C CKB2 PCNA DLGAP5 CCDC34 CCNA1 LMNB1

| 6.06E-63 | 0.527506 | 0.343 | 0.031 | 1.20E-58 | 18 | ELN | Astrocytes |
| --- | --- | --- | --- | --- | --- | --- | --- |
| 1.40E-22 | 0.525594 | 0.549 | 0.19 | 2.77E-18 | 18 | EMP3 | Astrocytes |
| 3.13E-26 | 0.523922 | 0.529 | 0.161 | 6.20E-22 | 18 | LTBP3 | Astrocytes |
| 4.31E-59 | 0.522299 | 0.373 | 0.04 | 8.53E-55 | 18 | EDNRB | Astrocytes |
| 1.23E-15 | 0.522238 | 0.578 | 0.272 | 2.45E-11 | 18 | FEZ1 | Astrocytes |
| 4.36E-26 | 0.522204 | 0.314 | 0.061 | 8.64E-22 | 18 | SOCS3 | Astrocytes |
| 2.01E-20 | 0.520459 | 0.99 | 0.927 | 3.97E-16 | 18 | BEX3 | Astrocytes |
| 1.76E-164 | 0.515604 | 0.333 | 0.009 | 3.49E-160 | 18 | HOXA5 | Astrocytes |
| 5.96E-14 | 0.513658 | 0.892 | 0.701 | 1.18E-09 | 18 | HSP90B1 | Astrocytes |
| A 9.81E-49 | 0.513278 | 0.618 | 0.131 | 1.94E-44 | 18 | TNFRSF1A | Astrocytes |
| 7.71E-194 | 0.510477 | 0.461 | 0.015 | 1.53E-189 | 18 | NUPR1 | Astrocytes |
| 1.55E-220 | 0.506209 | 0.441 | 0.012 | 3.07E-216 | 18 | HOXA3 | Astrocytes |
| 2.97E-20 | 0.505846 | 0.451 | 0.14 | 5.88E-16 | 18 | ZFP36 | Astrocytes |
| 3.72E-28 | 0.50426 | 0.324 | 0.062 | 7.37E-24 | 18 | GATM | Astrocytes |
| 6.83E-16 | 0.503773 | 1 | 1 | 1.35E-11 | 18 | FTH1 | Astrocytes |
| 2.31E-113 | 0.503701 | 0.333 | 0.015 | 4.58E-109 | 18 | WLS | Astrocytes |
| 7.82E-69 | 1.852923 | 0.742 | 0.14 | 1.55E-64 | 19 | TOP2A | Late RPCs |
| 1.58E-47 | 1.656741 | 0.656 | 0.147 | 3.13E-43 | 19 | CENPF | Late RPCs |
| 8.27E-47 | 1.620425 | 0.978 | 0.519 | 1.64E-42 | 19 | HMGB2 | Late RPCs |
| 1.50E-26 | 1.60131 | 0.624 | 0.219 | 2.97E-22 | 19 | PTTG1 | Late RPCs |
| 1.83E-49 | 1.515063 | 0.57 | 0.106 | 3.63E-45 | 19 | UBE2C | Late RPCs |
| 4.91E-39 | 1.511948 | 0.452 | 0.084 | 9.72E-35 | 19 | CCNB1 | Late RPCs |
| 4.02E-73 | 1.506992 | 0.559 | 0.07 | 7.95E-69 | 19 | CCNB2 | Late RPCs |
| 4.03E-67 | 1.475313 | 0.763 | 0.155 | 7.98E-63 | 19 | NUSAP1 | Late RPCs |
| 3.19E-46 | 1.397467 | 0.871 | 0.299 | 6.32E-42 | 19 | CKS2 | Late RPCs |
| 8.01E-73 | 1.392874 | 0.699 | 0.115 | 1.59E-68 | 19 | CDK1 | Late RPCs |
| 8.23E-70 | 1.312427 | 0.591 | 0.082 | 1.63E-65 | 19 | MKI67 | Late RPCs |
| 1.63E-69 | 1.254688 | 0.602 | 0.087 | 3.22E-65 | 19 | TPX2 | Late RPCs |
| 9.01E-17 | 1.215158 | 0.602 | 0.284 | 1.78E-12 | 19 | KPNA2 | Late RPCs |
| 3.14E-64 | 1.206126 | 0.828 | 0.188 | 6.22E-60 | 19 | SMC4 | Late RPCs |
| 3.36E-49 | 1.178941 | 0.925 | 0.326 | 6.65E-45 | 19 | TYMS | Late RPCs |
| 1.29E-97 | 1.175488 | 0.538 | 0.047 | 2.55E-93 | 19 | ASPM | Late RPCs |
| 4.81E-43 | 1.15721 | 1 | 0.902 | 9.53E-39 | 19 | TUBA1B | Late RPCs |
| 6.48E-14 | 1.136086 | 0.828 | 0.643 | 1.28E-09 | 19 | UBE2S | Late RPCs |
| 5.82E-63 | 1.130733 | 0.742 | 0.158 | 1.15E-58 | 19 | MAD2L1 | Late RPCs |
| 4.21E-58 | 1.128905 | 0.57 | 0.092 | 8.34E-54 | 19 | CDKN3 | Late RPCs |
| 3.20E-74 | 1.123082 | 0.43 | 0.04 | 6.33E-70 | 19 | CDC20 | Late RPCs |
| 3.24E-105 | 1.094627 | 0.548 | 0.046 | 6.41E-101 | 19 | PBK | Late RPCs |
| 1.20E-48 | 1.090001 | 0.828 | 0.25 | 2.38E-44 | 19 | UBE2T | Late RPCs |
| 9.33E-45 | 1.056035 | 0.753 | 0.231 | 1.85E-40 | 19 | H2AFX | Late RPCs |
| 1.73E-116 | 1.054449 | 0.538 | 0.039 | 3.43E-112 | 19 | NUF2 | Late RPCs |
| 3.07E-60 | 1.032445 | 0.763 | 0.158 | 6.08E-56 | 19 | PCLAF | Late RPCs |
| 4.38E-53 | 1.019115 | 0.419 | 0.053 | 8.66E-49 | 19 | NEK2 | Late RPCs |
| 1.19E-53 | 0.977443 | 0.688 | 0.149 | 2.36E-49 | 19 | CKS1B | Late RPCs |
| 1.92E-106 | 0.950653 | 0.538 | 0.043 | 3.80E-102 | 19 | CCNA2 | Late RPCs |
| 4.33E-66 | 0.939206 | 0.495 | 0.06 | 8.57E-62 | 19 | BIRC5 | Late RPCs |
| 0.000427 | 0.912577 | 0.688 | 0.585 | 1 | 19 | ARL6IP1 | Late RPCs |
| 2.50E-40 | 0.895936 | 1 | 0.955 | 4.95E-36 | 19 | H2AFZ | Late RPCs |
| 2.68E-61 | 0.88411 | 0.591 | 0.094 | 5.31E-57 | 19 | KIF20B | Late RPCs |
| 3.82E-81 | 0.871502 | 0.516 | 0.053 | 7.56E-77 | 19 | SGO2 | Late RPCs |
| 3.95E-29 | 0.856224 | 0.634 | 0.2 | 7.83E-25 | 19 | CKAP2 | Late RPCs |
| 8.13E-30 | 0.855985 | 0.753 | 0.3 | 1.61E-25 | 19 | FBLN1 | Late RPCs |
| 7.73E-21 | 0.85491 | 0.484 | 0.16 | 1.53E-16 | 19 | TUBA1C | Late RPCs |
| 9.36E-27 | 0.846585 | 1 | 0.98 | 1.85E-22 | 19 | CKB | Late RPCs |
| 2.06E-30 | 0.843018 | 0.796 | 0.336 | 4.07E-26 | 19 | PCNA | Late RPCs |
| 4.14E-112 | 0.838065 | 0.462 | 0.029 | 8.20E-108 | 19 | DLGAP5 | Late RPCs |
| 1.19E-44 | 0.834905 | 0.839 | 0.266 | 2.36E-40 | 19 | CCDC34 | Late RPCs |
| 4.54E-31 | 0.826571 | 0.301 | 0.046 | 8.99E-27 | 19 | CCNA1 | Late RPCs |
| 7.17E-50 | 0.804307 | 0.742 | 0.187 | 1.42E-45 | 19 | LMNB1 | Late RPCs |

| HMMR | 8.90E-72 | 0.797499 | 0.366 | 0.029 | 1.76E-67 | 19 | HMMR | Late RPCs |
| --- | --- | --- | --- | --- | --- | --- | --- | --- |
| CENPA | 1.82E-63 | 0.791516 | 0.387 | 0.037 | 3.60E-59 | 19 | CENPA | Late RPCs |
| MZT1 | 1.97E-22 | 0.780806 | 0.72 | 0.321 | 3.90E-18 | 19 | MZT1 | Late RPCs |
| CCND14 | 1.45E-17 | 0.776823 | 0.677 | 0.315 | 2.88E-13 | 19 | CCND1 | Late RPCs |
| HMGN21 | 2.10E-24 | 0.772783 | 0.978 | 0.894 | 4.16E-20 | 19 | HMGN2 | Late RPCs |
| CENPE | 1.82E-58 | 0.769575 | 0.398 | 0.043 | 3.61E-54 | 19 | CENPE | Late RPCs |
| SMC2 | 1.63E-43 | 0.7692 | 0.731 | 0.197 | 3.23E-39 | 19 | SMC2 | Late RPCs |
| CDCA3 | 1.24E-61 | 0.764777 | 0.441 | 0.051 | 2.45E-57 | 19 | CDCA3 | Late RPCs |
| AURKB | 6.97E-121 | 0.763936 | 0.484 | 0.03 | 1.38E-116 | 19 | AURKB | Late RPCs |
| FAM111B1 | 2.73E-45 | 0.749112 | 0.57 | 0.11 | 5.41E-41 | 19 | FAM111B | Late RPCs |
| HMGB3 | 1.19E-26 | 0.747597 | 0.753 | 0.312 | 2.36E-22 | 19 | HMGB3 | Late RPCs |
| MXD3 | 3.42E-93 | 0.739634 | 0.452 | 0.034 | 6.78E-89 | 19 | MXD3 | Late RPCs |
| KNSTRN | 7.56E-12 | 0.731843 | 0.355 | 0.137 | 1.50E-07 | 19 | KNSTRN | Late RPCs |
| NUCKS11 | 5.47E-24 | 0.731219 | 0.925 | 0.77 | 1.08E-19 | 19 | NUCKS1 | Late RPCs |
| ASCL11 | 2.58E-44 | 0.725566 | 0.667 | 0.148 | 5.12E-40 | 19 | ASCL1 | Late RPCs |
| HIST1H4C1 | 0.001255 | 0.72394 | 0.473 | 0.354 | 1 | 19 | HIST1H4C | Late RPCs |
| DTYMK | 3.69E-40 | 0.716924 | 0.753 | 0.238 | 7.30E-36 | 19 | DTYMK | Late RPCs |
| MIS18BP1 | 1.99E-46 | 0.712458 | 0.559 | 0.107 | 3.93E-42 | 19 | MIS18BP1 | Late RPCs |
| PIMREG | 2.79E-115 | 0.706344 | 0.484 | 0.031 | 5.53E-111 | 19 | PIMREG | Late RPCs |
| PLK1 | 1.66E-57 | 0.702429 | 0.333 | 0.03 | 3.29E-53 | 19 | PLK1 | Late RPCs |
| CRNDE | 1.08E-42 | 0.700807 | 0.796 | 0.235 | 2.13E-38 | 19 | CRNDE | Late RPCs |
| KIF23 | 4.35E-74 | 0.698584 | 0.398 | 0.033 | 8.61E-70 | 19 | KIF23 | Late RPCs |
| ECT2 | 3.07E-42 | 0.695483 | 0.527 | 0.103 | 6.08E-38 | 19 | ECT2 | Late RPCs |
| TUBB4B2 | 8.19E-12 | 0.692803 | 0.968 | 0.857 | 1.62E-07 | 19 | TUBB4B | Late RPCs |
| NDC80 | 2.40E-96 | 0.69205 | 0.484 | 0.038 | 4.75E-92 | 19 | NDC80 | Late RPCs |
| NMU | 3.37E-25 | 0.690182 | 0.366 | 0.079 | 6.67E-21 | 19 | NMU | Late RPCs |
| PMAIP11 | 1.56E-30 | 0.682678 | 0.667 | 0.202 | 3.09E-26 | 19 | PMAIP1 | Late RPCs |
| TMEM106 | 1.03E-27 | 0.681672 | 0.806 | 0.339 | 2.03E-23 | 19 | TMEM106 | Late RPCs |
| TAGLN2 | 7.78E-21 | 0.680229 | 0.774 | 0.372 | 1.54E-16 | 19 | TAGLN2 | Late RPCs |
| CENPU | 5.98E-65 | 0.679243 | 0.699 | 0.116 | 1.18E-60 | 19 | CENPU | Late RPCs |
| AURKA | 1.33E-71 | 0.660898 | 0.387 | 0.033 | 2.64E-67 | 19 | AURKA | Late RPCs |
| TMPO | 1.54E-34 | 0.655901 | 0.86 | 0.338 | 3.04E-30 | 19 | TMPO | Late RPCs |
| RAD21 | 3.48E-17 | 0.652322 | 0.774 | 0.432 | 6.90E-13 | 19 | RAD21 | Late RPCs |
| GTSE1 | 4.76E-68 | 0.650578 | 0.473 | 0.052 | 9.42E-64 | 19 | GTSE1 | Late RPCs |
| LSM5 | 1.84E-18 | 0.645645 | 0.699 | 0.339 | 3.65E-14 | 19 | LSM5 | Late RPCs |
| ZWINT | 1.87E-66 | 0.642215 | 0.667 | 0.104 | 3.70E-62 | 19 | ZWINT | Late RPCs |
| CKAP2L | 7.78E-99 | 0.640627 | 0.473 | 0.035 | 1.54E-94 | 19 | CKAP2L | Late RPCs |
| PRSS233 | 9.11E-21 | 0.636394 | 0.785 | 0.356 | 1.80E-16 | 19 | PRSS23 | Late RPCs |
| ASF1B | 3.09E-81 | 0.636194 | 0.591 | 0.067 | 6.11E-77 | 19 | ASF1B | Late RPCs |
| H2AFV | 8.48E-24 | 0.632508 | 0.892 | 0.515 | 1.68E-19 | 19 | H2AFV | Late RPCs |
| CENPK | 2.66E-67 | 0.630417 | 0.656 | 0.101 | 5.28E-63 | 19 | CENPK | Late RPCs |
| ATAD2 | 1.22E-52 | 0.621671 | 0.656 | 0.126 | 2.42E-48 | 19 | ATAD2 | Late RPCs |
| HELLS | 1.73E-33 | 0.619068 | 0.677 | 0.195 | 3.42E-29 | 19 | HELLS | Late RPCs |
| RRM1 | 1.09E-33 | 0.615502 | 0.753 | 0.239 | 2.16E-29 | 19 | RRM1 | Late RPCs |
| FOS4 | 2.43E-11 | 0.615456 | 0.806 | 0.586 | 4.82E-07 | 19 | FOS | Late RPCs |
| HES14 | 4.39E-13 | 0.614196 | 0.613 | 0.279 | 8.70E-09 | 19 | HES1 | Late RPCs |
| BTG31 | 4.05E-24 | 0.611776 | 0.86 | 0.43 | 8.01E-20 | 19 | BTG3 | Late RPCs |
| FBXO5 | 8.37E-37 | 0.606772 | 0.538 | 0.122 | 1.66E-32 | 19 | FBXO5 | Late RPCs |
| ANP32E1 | 3.88E-20 | 0.60607 | 0.785 | 0.413 | 7.68E-16 | 19 | ANP32E | Late RPCs |
| VSTM2B1 | 5.08E-37 | 0.603351 | 0.602 | 0.142 | 1.01E-32 | 19 | VSTM2B | Late RPCs |
| DUT | 2.23E-13 | 0.600091 | 0.828 | 0.617 | 4.41E-09 | 19 | DUT | Late RPCs |
| HMGB11 | 3.86E-21 | 0.599346 | 1 | 0.977 | 7.63E-17 | 19 | HMGB1 | Late RPCs |
| NCAPG | 8.34E-90 | 0.598146 | 0.538 | 0.05 | 1.65E-85 | 19 | NCAPG | Late RPCs |
| RTKN2 | 1.17E-77 | 0.592934 | 0.495 | 0.05 | 2.32E-73 | 19 | RTKN2 | Late RPCs |
| VIM5 | 3.30E-17 | 0.591939 | 0.978 | 0.752 | 6.53E-13 | 19 | VIM | Late RPCs |
| GPM6B4 | 7.79E-18 | 0.590433 | 0.914 | 0.723 | 1.54E-13 | 19 | GPM6B | Late RPCs |
| TROAP | 3.27E-85 | 0.589112 | 0.387 | 0.027 | 6.48E-81 | 19 | TROAP | Late RPCs |
| PLEKHA13 | 1.52E-13 | 0.588632 | 0.731 | 0.476 | 3.02E-09 | 19 | PLEKHA1 | Late RPCs |
| RAN | 8.69E-26 | 0.585189 | 0.989 | 0.91 | 1.72E-21 | 19 | RAN | Late RPCs |

| SYNE2 | 7.85E-20 | 0.583308 | 0.828 | 0.453 | 1.55E-15 | 19 | SYNE2 | Late RPCs |
| --- | --- | --- | --- | --- | --- | --- | --- | --- |
| GINS2 | 3.24E-30 | 0.582013 | 0.667 | 0.209 | 6.41E-26 | 19 | GINS2 | Late RPCs |
| CDCA8 | 6.65E-89 | 0.575069 | 0.376 | 0.024 | 1.32E-84 | 19 | CDCA8 | Late RPCs |
| TACC3 | 1.52E-49 | 0.570985 | 0.505 | 0.08 | 3.02E-45 | 19 | TACC3 | Late RPCs |
| CENPH | 6.16E-42 | 0.570881 | 0.699 | 0.17 | 1.22E-37 | 19 | CENPH | Late RPCs |
| KIF11 | 8.30E-89 | 0.569864 | 0.462 | 0.038 | 1.64E-84 | 19 | KIF11 | Late RPCs |
| FEN1 | 1.84E-28 | 0.568647 | 0.677 | 0.223 | 3.65E-24 | 19 | FEN1 | Late RPCs |
| ZFP36L13 | 1.48E-20 | 0.568181 | 0.785 | 0.321 | 2.94E-16 | 19 | ZFP36L1 | Late RPCs |
| CDKN2C1 | 6.27E-25 | 0.565303 | 0.527 | 0.154 | 1.24E-20 | 19 | CDKN2C | Late RPCs |
| E2F11 | 1.83E-22 | 0.564279 | 0.624 | 0.223 | 3.63E-18 | 19 | E2F1 | Late RPCs |
| SGO1 | 1.32E-69 | 0.562331 | 0.419 | 0.04 | 2.61E-65 | 19 | SGO1 | Late RPCs |
| SPC25 | 8.90E-87 | 0.562299 | 0.452 | 0.037 | 1.76E-82 | 19 | SPC25 | Late RPCs |
| BUB1 | 2.12E-130 | 0.55987 | 0.43 | 0.02 | 4.20E-126 | 19 | BUB1 | Late RPCs |
| TUBB6 | 8.93E-47 | 0.556115 | 0.505 | 0.085 | 1.77E-42 | 19 | TUBB6 | Late RPCs |
| KNL1 | 4.65E-76 | 0.555433 | 0.366 | 0.027 | 9.20E-72 | 19 | KNL1 | Late RPCs |
| BUB3 | 4.19E-18 | 0.555105 | 0.699 | 0.343 | 8.30E-14 | 19 | BUB3 | Late RPCs |
| SOX24 | 4.44E-18 | 0.548918 | 0.774 | 0.339 | 8.80E-14 | 19 | SOX2 | Late RPCs |
| DBF4 | 4.09E-26 | 0.54784 | 0.495 | 0.131 | 8.10E-22 | 19 | DBF4 | Late RPCs |
| PRC1 | 1.10E-58 | 0.547774 | 0.495 | 0.064 | 2.17E-54 | 19 | PRC1 | Late RPCs |
| GGH1 | 1.50E-28 | 0.546641 | 0.785 | 0.297 | 2.98E-24 | 19 | GGH | Late RPCs |
| NAP1L1 | 7.31E-22 | 0.543997 | 0.978 | 0.827 | 1.45E-17 | 19 | NAP1L1 | Late RPCs |
| TTK | 1.92E-101 | 0.53725 | 0.376 | 0.021 | 3.79E-97 | 19 | TTK | Late RPCs |
| TMSB15A | 1.49E-18 | 0.536926 | 0.806 | 0.418 | 2.95E-14 | 19 | TMSB15A | Late RPCs |
| EZH2 | 2.21E-31 | 0.535968 | 0.677 | 0.207 | 4.38E-27 | 19 | EZH2 | Late RPCs |
| CALM3 | 4.44E-11 | 0.531275 | 0.774 | 0.594 | 8.78E-07 | 19 | CALM3 | Late RPCs |
| DEPDC1 | 2.89E-127 | 0.52926 | 0.398 | 0.018 | 5.72E-123 | 19 | DEPDC1 | Late RPCs |
| RRM2 | 2.38E-34 | 0.527957 | 0.473 | 0.093 | 4.71E-30 | 19 | RRM2 | Late RPCs |
| RACGAP1 | 1.70E-39 | 0.52665 | 0.495 | 0.094 | 3.36E-35 | 19 | RACGAP1 | Late RPCs |
| GMNN1 | 1.41E-28 | 0.524482 | 0.699 | 0.226 | 2.80E-24 | 19 | GMNN | Late RPCs |
| FABP51 | 4.70E-19 | 0.521996 | 0.849 | 0.448 | 9.30E-15 | 19 | FABP5 | Late RPCs |
| MCM7 | 1.48E-20 | 0.521769 | 0.667 | 0.27 | 2.93E-16 | 19 | MCM7 | Late RPCs |
| NASP | 2.15E-13 | 0.521582 | 0.774 | 0.615 | 4.26E-09 | 19 | NASP | Late RPCs |
| JUNB5 | 2.21E-07 | 0.52066 | 0.742 | 0.557 | 0.004382 | 19 | JUNB | Late RPCs |
| ESCO2 | 6.35E-91 | 0.514195 | 0.398 | 0.026 | 1.26E-86 | 19 | ESCO2 | Late RPCs |
| CLGN | 1.16E-26 | 0.512751 | 0.753 | 0.29 | 2.30E-22 | 19 | CLGN | Late RPCs |
| RAD51AP1 | 2.19E-47 | 0.511277 | 0.505 | 0.082 | 4.33E-43 | 19 | RAD51AP1 | Late RPCs |
| DNAJC9 | 5.43E-28 | 0.509113 | 0.656 | 0.212 | 1.08E-23 | 19 | DNAJC9 | Late RPCs |
| SIVA1 | 4.46E-18 | 0.504863 | 0.71 | 0.342 | 8.82E-14 | 19 | SIVA1 | Late RPCs |
| FAM111A | 5.40E-35 | 0.504773 | 0.484 | 0.097 | 1.07E-30 | 19 | FAM111A | Late RPCs |
| ZFP36L23 | 1.46E-12 | 0.500287 | 0.677 | 0.341 | 2.90E-08 | 19 | ZFP36L2 | Late RPCs |
| AHI11 | 0.000278 | 0.652557 | 0.49 | 0.342 | 1 | 20 | AHI1 | UPRCs |
| RS11 | 6.76E-14 | 0.646309 | 0.449 | 0.117 | 1.34E-09 | 20 | RS1 | UPRCs |
| NT5DC22 | 2.06E-07 | 0.615339 | 0.633 | 0.364 | 0.004071 | 20 | NT5DC2 | UPRCs |
| CPE4 | 2.57E-08 | 0.592835 | 0.959 | 0.672 | 0.00051 | 20 | CPE | UPRCs |
| VXN2 | 2.38E-07 | 0.581984 | 0.449 | 0.182 | 0.004703 | 20 | VXN | UPRCs |
| AKAP93 | 0.000123 | 0.52741 | 0.878 | 0.613 | 1 | 20 | AKAP9 | UPRCs |
| SCG34 | 6.16E-08 | 0.527027 | 0.837 | 0.514 | 0.00122 | 20 | SCG3 | UPRCs |
| HES61 | 2.53E-11 | 0.948138 | 0.956 | 0.57 | 5.00E-07 | 21 | HES6 | NRPCs/T1 |
| MIAT2 | 5.34E-05 | 0.756252 | 0.533 | 0.363 | 1 | 21 | MIAT | NRPCs/T1 |
| GADD45A2 | 7.18E-11 | 0.647971 | 0.756 | 0.389 | 1.42E-06 | 21 | GADD45A | NRPCs/T1 |
| CXCR41 | 3.70E-05 | 0.63735 | 0.489 | 0.268 | 0.733026 | 21 | CXCR4 | NRPCs/T1 |
| HMGB23 | 2.91E-05 | 0.598983 | 0.778 | 0.525 | 0.57666 | 21 | HMGB2 | NRPCs/T1 |
| PDE6H2 | 1.09E-26 | 2.209413 | 1 | 0.328 | 2.16E-22 | 22 | PDE6H | Cone precursors |
| GUCA1A1 | 6.05E-44 | 2.147127 | 1 | 0.162 | 1.20E-39 | 22 | GUCA1A | Cone precursors |
| ARR31 | 2.20E-46 | 1.793692 | 0.967 | 0.131 | 4.36E-42 | 22 | ARR3 | Cone precursors |
| RCVRN3 | 7.75E-20 | 1.568238 | 1 | 0.431 | 1.53E-15 | 22 | RCVRN | Cone precursors |
| GNB33 | 2.40E-22 | 1.540815 | 1 | 0.374 | 4.75E-18 | 22 | GNB3 | Cone precursors |
| GUCA1B1 | 2.82E-87 | 1.518438 | 0.933 | 0.059 | 5.59E-83 | 22 | GUCA1B | Cone precursors |
| KCNV22 | 1.70E-35 | 1.517561 | 1 | 0.187 | 3.36E-31 | 22 | KCNV2 | Cone precursors |

UNC1192 DST VSX12 GNGT22 PDC3 AKAP94 MYL41 GUK12 CPLX42 IMPG22 AIPL13

| 1.37E-19 | 1.435698 | 1 | 0.506 | 2.72E-15 | 22 | UNC119 | Cone precursors |
| --- | --- | --- | --- | --- | --- | --- | --- |
| 7.47E-21 | 1.29715 | 1 | 0.422 | 1.48E-16 | 22 | DST | Cone precursors |
| 1.93E-22 | 1.255962 | 0.767 | 0.147 | 3.83E-18 | 22 | VSX1 | Cone precursors |
| 1.90E-38 | 1.249921 | 1 | 0.162 | 3.77E-34 | 22 | GNGT2 | Cone precursors |
| 5.06E-17 | 1.20432 | 1 | 0.416 | 1.00E-12 | 22 | PDC | Cone precursors |
| 5.05E-17 | 1.183198 | 1 | 0.613 | 1.00E-12 | 22 | AKAP9 | Cone precursors |
| 7.67E-47 | 1.162636 | 0.933 | 0.114 | 1.52E-42 | 22 | MYL4 | Cone precursors |
| 6.47E-17 | 1.157634 | 1 | 0.791 | 1.28E-12 | 22 | GUK1 | Cone precursors |
| 1.25E-43 | 1.135062 | 1 | 0.147 | 2.48E-39 | 22 | CPLX4 | Cone precursors |
| 1.27E-30 | 1.105984 | 0.967 | 0.195 | 2.52E-26 | 22 | IMPG2 | Cone precursors |
| 4.39E-23 | 1.101883 | 1 | 0.266 | 8.69E-19 | 22 | AIPL1 | Cone precursors |
| 1.78E-20 | 1.098482 | 1 | 0.333 | 3.52E-16 | 22 | Sep-04 | Cone precursors |
| 5.86E-27 | 1.0946 | 1 | 0.253 | 1.16E-22 | 22 | FSTL5 | Cone precursors |
| 6.66E-36 | 1.065362 | 0.967 | 0.158 | 1.32E-31 | 22 | MPP4 | Cone precursors |
| 5.67E-22 | 1.036122 | 1 | 0.304 | 1.12E-17 | 22 | CRX | Cone precursors |
| 1.54E-16 | 1.032314 | 1 | 0.556 | 3.05E-12 | 22 | PCBP4 | Cone precursors |
| 6.82E-20 | 1.016113 | 0.8 | 0.181 | 1.35E-15 | 22 | VXN | Cone precursors |
| 1.48E-19 | 1.014407 | 0.933 | 0.27 | 2.94E-15 | 22 | TUBA4A | Cone precursors |
| 2.85E-13 | 1.010592 | 0.7 | 0.201 | 5.64E-09 | 22 | RRAD | Cone precursors |
| 1.73E-20 | 1.006622 | 1 | 0.32 | 3.43E-16 | 22 | CADPS | Cone precursors |
| 1.49E-19 | 0.993981 | 0.967 | 0.347 | 2.95E-15 | 22 | TPD52 | Cone precursors |
| 4.26E-16 | 0.986971 | 0.8 | 0.221 | 8.43E-12 | 22 | CA2 | Cone precursors |
| 4.75E-15 | 0.982174 | 1 | 0.583 | 9.39E-11 | 22 | MAP2 | Cone precursors |
| 1.35E-25 | 0.969013 | 1 | 0.243 | 2.67E-21 | 22 | TULP1 | Cone precursors |
| 2.94E-20 | 0.923857 | 0.8 | 0.186 | 5.81E-16 | 22 | PEX5L | Cone precursors |
| 3.84E-17 | 0.89643 | 0.933 | 0.31 | 7.60E-13 | 22 | DPYSL3 | Cone precursors |
| 1.22E-49 | 0.894749 | 0.7 | 0.057 | 2.41E-45 | 22 | TMEM215 | Cone precursors |
| 6.24E-28 | 0.870882 | 0.767 | 0.121 | 1.24E-23 | 22 | RP1 | Cone precursors |
| 1.59E-25 | 0.866859 | 1 | 0.242 | 3.15E-21 | 22 | STX3 | Cone precursors |
| 2.99E-13 | 0.848936 | 1 | 0.961 | 5.93E-09 | 22 | MT-ND2 | Cone precursors |
| 2.37E-10 | 0.847403 | 1 | 0.858 | 4.69E-06 | 22 | TUBB4B | Cone precursors |
| 3.99E-13 | 0.841732 | 1 | 0.673 | 7.90E-09 | 22 | CPE | Cone precursors |
| 2.65E-23 | 0.831654 | 0.833 | 0.175 | 5.24E-19 | 22 | FAM19A4 | Cone precursors |
| 5.39E-09 | 0.825622 | 0.9 | 0.601 | 0.000107 | 22 | KCNQ1OT1 | Cone precursors |
| 3.86E-33 | 0.805831 | 0.833 | 0.116 | 7.63E-29 | 22 | RS1 | Cone precursors |
| 1.95E-13 | 0.804103 | 0.967 | 0.5 | 3.87E-09 | 22 | PPP1CC | Cone precursors |
| 4.64E-17 | 0.801677 | 1 | 0.361 | 9.18E-13 | 22 | SYP | Cone precursors |
| 5.18E-24 | 0.79428 | 0.9 | 0.198 | 1.02E-19 | 22 | RIMS2 | Cone precursors |
| 2.75E-12 | 0.794216 | 0.767 | 0.256 | 5.44E-08 | 22 | NRN1 | Cone precursors |
| 2.06E-20 | 0.793626 | 0.767 | 0.17 | 4.07E-16 | 22 | ALDOC | Cone precursors |
| 6.40E-13 | 0.781178 | 0.933 | 0.505 | 1.27E-08 | 22 | ARL6IP5 | Cone precursors |
| 5.03E-24 | 0.779702 | 0.733 | 0.123 | 9.95E-20 | 22 | RTBDN | Cone precursors |
| 1.13E-10 | 0.7765 | 0.867 | 0.46 | 2.24E-06 | 22 | DHRS7 | Cone precursors |
| 1.70E-21 | 0.774375 | 0.833 | 0.17 | 3.36E-17 | 22 | GSG1 | Cone precursors |
| 1.63E-45 | 0.766889 | 0.9 | 0.103 | 3.23E-41 | 22 | GNAT2 | Cone precursors |
| 2.28E-19 | 0.760073 | 0.767 | 0.164 | 4.52E-15 | 22 | HRASLS | Cone precursors |
| 4.64E-13 | 0.759502 | 0.967 | 0.399 | 9.19E-09 | 22 | MAP1LC3A | Cone precursors |
| 1.65E-10 | 0.758915 | 0.967 | 0.875 | 3.27E-06 | 22 | PRDX1 | Cone precursors |
| 1.11E-11 | 0.754473 | 1 | 0.665 | 2.19E-07 | 22 | SYT1 | Cone precursors |
| 1.33E-13 | 0.753113 | 0.867 | 0.318 | 2.64E-09 | 22 | CADM2 | Cone precursors |
| 1.03E-17 | 0.750821 | 0.933 | 0.254 | 2.04E-13 | 22 | PLEKHB1 | Cone precursors |
| 7.42E-14 | 0.741535 | 0.867 | 0.309 | 1.47E-09 | 22 | PTP4A3 | Cone precursors |
| 7.13E-10 | 0.74062 | 0.833 | 0.342 | 1.41E-05 | 22 | DDIT3 | Cone precursors |
| 8.53E-16 | 0.736861 | 0.833 | 0.247 | 1.69E-11 | 22 | ANK2 | Cone precursors |
| 1.39E-10 | 0.731467 | 0.867 | 0.398 | 2.76E-06 | 22 | ZNF326 | Cone precursors |
| 1.09E-12 | 0.729458 | 1 | 0.683 | 2.15E-08 | 22 | ENO2 | Cone precursors |
| 7.92E-17 | 0.726875 | 0.767 | 0.192 | 1.57E-12 | 22 | PRCD | Cone precursors |
| 1.18E-18 | 0.724559 | 0.967 | 0.267 | 2.34E-14 | 22 | NEUROD4 | Cone precursors |
| 1.67E-12 | 0.71057 | 1 | 0.649 | 3.31E-08 | 22 | LAPTM4B | Cone precursors |

Sep-43 FSTL52 MPP42 CRX3 PCBP44 VXN3 TUBA4A2 RRAD4 CADPS2 TPD522 CA21 MAP22 TULP13 PEX5L1 DPYSL31 TMEM215 RP12 STX32

MT-ND2 TUBB4B3 CPE5 FAM19A41 KCNQ1OT1 RS12 PPP1CC3 SYP4 RIMS2 NRN12 ALDOC2 ARL6IP51 RTBDN2 DHRS71 GSG13 GNAT21 HRASLS1 MAP1LC3A PRDX12 SYT11 CADM21 PLEKHB13 PTP4A33 DDIT3 ANK2 ZNF326 ENO23 PRCD2 NEUROD43 LAPTM4B3

| 4.53E-14 | 0.694008 | 0.967 | 0.412 | 8.96E-10 | 22 SLC38A1 | Cone precursors |
| --- | --- | --- | --- | --- | --- | --- |
| 1.96E-33 | 0.693578 | 0.8 | 0.109 | 3.89E-29 | 22 PPP2R2B | Cone precursors |
| 6.80E-10 | 0.69124 | 0.867 | 0.387 | 1.35E-05 | 22 CRABP2 | Cone precursors |
| 3.43E-27 | 0.68882 | 0.833 | 0.145 | 6.79E-23 | 22 PROM1 | Cone precursors |
| 5.04E-13 | 0.687849 | 0.9 | 0.312 | 9.99E-09 | 22 LINC00599 | Cone precursors |
| 1.16E-16 | 0.687653 | 0.867 | 0.261 | 2.30E-12 | 22 LBH | Cone precursors |
| 7.39E-15 | 0.685524 | 0.867 | 0.284 | 1.46E-10 | 22 UGCG | Cone precursors |
| 1.94E-17 | 0.68258 | 0.833 | 0.218 | 3.84E-13 | 22 NEDD4L | Cone precursors |
| 2.16E-11 | 0.67132 | 0.833 | 0.316 | 4.28E-07 | 22 AGAP1 | Cone precursors |
| 5.60E-21 | 0.667464 | 0.9 | 0.205 | 1.11E-16 | 22 PTPN13 | Cone precursors |
| 1.36E-25 | 0.665299 | 0.8 | 0.136 | 2.68E-21 | 22 EYS | Cone precursors |
| .000174 | 0.661006 | 0.6 | 0.352 | 1 | 22 TMX4 | Cone precursors |
| 7.17E-12 | 0.659391 | 1 | 0.396 | 1.42E-07 | 22 FAM57B | Cone precursors |
| 5.53E-47 | 0.656606 | 0.8 | 0.079 | 1.10E-42 | 22 UBAP1L | Cone precursors |
| 3.82E-13 | 0.652694 | 0.7 | 0.202 | 7.56E-09 | 22 AANAT | Cone precursors |
| 8.44E-23 | 0.651321 | 0.6 | 0.09 | 1.67E-18 | 22 RABL3 | Cone precursors |
| 1.08E-24 | 0.649862 | 0.833 | 0.159 | 2.14E-20 | 22 ANKRD33B | Cone precursors |
| 6.44E-19 | 0.648124 | 0.8 | 0.185 | 1.28E-14 | 22 CC2D2A | Cone precursors |
| 5.22E-19 | 0.640821 | 0.8 | 0.195 | 1.03E-14 | 22 MEGF9 | Cone precursors |
| 1.10E-16 | 0.640521 | 0.867 | 0.236 | 2.17E-12 | 22 BTBD8 | Cone precursors |
| 2.96E-33 | 0.640066 | 0.8 | 0.11 | 5.87E-29 | 22 LMOD1 | Cone precursors |
| 2.03E-28 | 0.639765 | 0.833 | 0.136 | 4.03E-24 | 22 RD3 | Cone precursors |
| 2.45E-13 | 0.636288 | 0.967 | 0.327 | 4.86E-09 | 22 OTX2 | Cone precursors |
| 5.13E-70 | 0.636017 | 0.633 | 0.033 | 1.02E-65 | 22 GUCA1C | Cone precursors |
| 1.02E-13 | 0.632132 | 0.967 | 0.452 | 2.01E-09 | 22 WRB | Cone precursors |
| 2.85E-13 | 0.629359 | 0.967 | 0.397 | 5.63E-09 | 22 MAP4 | Cone precursors |
| 1.24E-14 | 0.626475 | 0.8 | 0.245 | 2.45E-10 | 22 STRADB | Cone precursors |
| 1.75E-09 | 0.625028 | 0.867 | 0.39 | 3.46E-05 | 22 PIK3R1 | Cone precursors |
| 1.79E-19 | 0.624799 | 0.7 | 0.132 | 3.55E-15 | 22 PCP4 | Cone precursors |
| 2.74E-13 | 0.620161 | 0.867 | 0.31 | 5.42E-09 | 22 MIR124-2HCone precursors | |
| 2.63E-20 | 0.616479 | 0.833 | 0.187 | 5.20E-16 | 22 DUSP8 Cone precursors | |
| 2.10E-11 | 0.613056 | 0.967 | 0.373 | 4.16E-07 | 22 NEUROD1 Cone precursors | |
| 2.32E-09 | 0.605609 | 1 | 0.904 | 4.59E-05 | 22 MAP1B Cone precursors | |
| 2.93E-11 | 0.600938 | 0.9 | 0.396 | 5.79E-07 | 22 CLCN3 Cone precursors | |
| 1.54E-28 | 0.600095 | 0.767 | 0.116 | 3.04E-24 | 22 ZNF385B Cone precursors | |
| 6.56E-16 | 0.597438 | 0.833 | 0.217 | 1.30E-11 | 22 NANOS1 Cone precursors | |
| 5.85E-15 | 0.588736 | 0.733 | 0.191 | 1.16E-10 | 22 SLC17A7 Cone precursors | |
| 7.40E-19 | 0.583409 | 0.933 | 0.232 | 1.46E-14 | 22 NLK Cone precursors | |
| 2.49E-10 | 0.577349 | 0.967 | 0.671 | 4.93E-06 | 22 NDUFV2 Cone precursors | |
| 6.56E-15 | 0.573901 | 0.767 | 0.21 | 1.30E-10 | 22 ELOVL4 Cone precursors | |
| 1.39E-08 | 0.571282 | 0.8 | 0.374 | 0.000274 | 22 KIF2A Cone precursors | |
| 3.50E-10 | 0.569568 | 0.833 | 0.384 | 6.93E-06 | 22 KIF1B Cone precursors | |
| 1.13E-08 | 0.568446 | 1 | 0.963 | 0.000223 | 22 MT-ND5 Cone precursors | |
| 8.03E-08 | 0.566799 | 1 | 0.995 | 0.001589 | 22 MT-ATP6 Cone precursors | |
| 9.72E-17 | 0.564379 | 0.8 | 0.2 | 1.92E-12 | 22 PDE4DIP Cone precursors | |
| 1.36E-10 | 0.557234 | 1 | 0.753 | 2.69E-06 | 22 PPA1 Cone precursors | |
| 6.87E-07 | 0.555714 | 1 | 0.992 | 0.013603 | 22 MT-CYB Cone precursors | |
| 4.55E-11 | 0.552022 | 0.767 | 0.276 | 9.00E-07 | 22 RORA Cone precursors | |
| 3.76E-10 | 0.543647 | 0.967 | 0.762 | 7.44E-06 | 22 ATP5F1A Cone precursors | |
| 3.15E-08 | 0.541775 | 1 | 0.889 | 0.000624 | 22 TTC3 Cone precursors | |
| 1.11E-07 | 0.538545 | 0.767 | 0.354 | 0.002189 | 22 NBEA Cone precursors | |
| 9.50E-10 | 0.536544 | 0.733 | 0.259 | 1.88E-05 | 22 AC027031. Cone precursors | |
| 3.74E-19 | 0.53344 | 0.767 | 0.161 | 7.41E-15 | 22 ROBO2 Cone precursors | |
| 2.80E-28 | 0.52907 | 0.633 | 0.077 | 5.54E-24 | 22 PRDM1 Cone precursors | |
| 1.52E-08 | 0.528692 | 0.833 | 0.41 | 0.000302 | 22 ATP1B2 Cone precursors | |
| 4.88E-23 | 0.528335 | 0.667 | 0.107 | 9.66E-19 | 22 KCNB2 Cone precursors | |
| 1.25E-24 | 0.528015 | 0.833 | 0.146 | 2.48E-20 | 22 MAK Cone precursors | |
| 1.36E-24 | 0.521283 | 0.667 | 0.101 | 2.70E-20 | 22 TRAK2 Cone precursors | |
| 1.61E-25 | 0.518312 | 0.367 | 0.031 | 3.19E-21 | 22 IMPG1 Cone precursors | |

| SLC38A12 |
| --- |
| PPP2R2B |
| CRABP22 |
| PROM11 |
| LINC00599 |
| LBH |
| UGCG1 |
| NEDD4L1 |
| AGAP11 |
| PTPN131 |
| EYS2 |
| TMX41 0 |
| FAM57B4 |
| UBAP1L |
| AANAT1 |
| RABL3 |
| ANKRD33B |
| CC2D2A1 |
| MEGF9 |
| BTBD81 |
| LMOD11 |
| RD31 |
| OTX23 |
| GUCA1C1 |
| WRB |
| MAP4 |
| STRADB |
| PIK3R12 |
| PCP41 |
| MIR124-2H |
| DUSP8 |
| NEUROD13 |
| MAP1B2 |
| CLCN3 |
| ZNF385B1 |
| NANOS11 |
| SLC17A71 |
| NLK1 |
| NDUFV2 |
| ELOVL4 |
| KIF2A1 |
| KIF1B |
| MT-ND5 |
| MT-ATP61 |
| PDE4DIP1 |
| PPA11 |
| MT-CYB |
| RORA |
| ATP5F1A |
| TTC31 |
| NBEA |
| AC027031. |
| ROBO2 |
| PRDM11 |
| ATP1B22 |
| KCNB2 |
| MAK1 |
| TRAK2 |
| IMPG1 |

ABHD14A1 RBP32 CCDC85B MT-ND1 FAM107A1

| 4.90E-11 | 0.518059 | 0.833 | 0.323 | 9.71E-07 | 22 | ABHD14A | Cone precursors |
| --- | --- | --- | --- | --- | --- | --- | --- |
| 7.86E-14 | 0.51737 | 0.667 | 0.16 | 1.56E-09 | 22 | RBP3 | Cone precursors |
| 5.52E-09 | 0.516778 | 0.867 | 0.393 | 0.000109 | 22 | CCDC85B | Cone precursors |
| 1.09E-08 | 0.514587 | 1 | 0.979 | 0.000215 | 22 | MT-ND1 | Cone precursors |
| 1.42E-26 | 0.512922 | 0.8 | 0.125 | 2.80E-22 | 22 | FAM107A | Cone precursors |
