# Supplementary material for: pRB-Depleted Pluripotent Stem Cell Retinal Organoids Recapitulate Cell State Transitions of Retinoblastoma Development and Suggest an Important Role for pRB in Retinal Cell Differentiation
Source: Stem Cells Transl Med. 2022 Mar 23;11(4):415–33. doi: 10.1093/stcltm/szac008 (PMC9052432; doi:10.1093/stcltm/szac008)
Supplement: szac008_suppl_Supplementary_Table_S2 [file szac008_suppl_supplementary_table_s2.docx]

| Primers | Off-target sites for gRNA:  ATAGTTCATACTCATTCTGC **AGG**  correcting heterozygous c.2082delC mutation to create isogenic control | Sequencing result |
| --- | --- | --- |
| **F;** TCCTGCAATGCTGCAAGTTC  **R;** GAAGCACAGGGGTTCGTGTA | **I. c**TAGTTCA**g**ACT**t**ATTCTGC **AGG**  **(chr4, + strand)** | GGCTAGTTCAGACTTATTCTGC**AGG**CC |
| **F;** AAACTCACTGAGGAGGAACGC  **R;** TCTCATCTGTTCCCATCCAGC | II. A**g**AG**c**TCAT**c**CTCATTCTGC **AGG**  **(chr21, + strand)** | GAAGAGCTCATCCTCATTCTGC**AGG**AT |
| **F;** TGAGACAAAGACAACACATGCC  **R;** ACCACAATTTGTATTCTGATAGCTC | III. A**c**AGTT**t**ATA**t**TCATTCTGC **TGG**  **(chr1, - strand)** | AC**CCA**GCAGAATGAATATAAACTGTGG |
| **F;** ACATGCAATAGAGAACTACAGGCA  **R;** CAGATAAGCGGGAGAAAACTGC | IV. ATtGTgCATcCTCATTCTGC **AGG**  **(chr10, - strand)** | CT**CCT**GCAGAATGAGGATGCACAATTG |
| **F;** CCACTCTTAGGAAGAAGCGGG  **R;** TTTGGCAAAGTGGCCTATTGT | V. AT**t**GTTCA**a**A**g**TCATTCTGC **AGG**  **(chr10, + strand)** | AAATTGTTCAAAGTCATTCTGC**AGG**AG |
|  |  |  |
|  | Off-target sites for gRNA:  GAGTTCATACTCATTCTGCA **GGG**  introducing homozygous c.2082delC mutation |  |
| **F:** TCCACCATTGTGATGGACTGT  **R:** TGAGTAGCATTCTCATGACTGTGT | VI. G**gta**TCATACTCATTCTGCA **GGG**  **(chr1, - strand)** | AT**CCCTGCAGAATGAGTATGATACC**AA |
| **F;** GTGCATGGATGTTCCCTCCT  **R;** ACTAACCACCCCAGATACAGA | VII. G**g**G**g**TCAT**t**CTCATTCTGCA **CGG**  **(chr11, + strand)** | CT**GGGRTCATTCTCATTCTGCACGG**CA |
| **F;** GTGCTTGTGGTGGGGAATAGA  **R;** GACTAGCAGCAGGAGAGGTTT | VIII. GA**t**TT**t**A**g**ACTCATTCTGCA **AGG**  **(chr10, + strand)** | TA**GATTTTAGACTCATTCTGCAAGG**TG |
| **F;** TGGAAGCTAGAAGCCAAAAATCA  **R;** CCACCACCACCTGTGTGTAT | IX. GA**c**TTCATAC**a**CAT**g**CTGCA **GGG**  **(chr5, - strand)** | TC**CCCTGCAGCATGTGTATGAAGTC**AC |
| **F;** ATGAATCACAGAGAAATGCAGAAC  **R;** AGCCAATGGAAGAAAGAAGTTAGA | **X. c**A**t**T**g**CAT**t**CTCATTCTGCA **GGG**  **(chr6, + strand)** | TT**CATTGCATTCTCATTCTGCAGGG**AA |

**Table S2. Off-target sequences for c.2082delC in *RB1*.**
