# Supplementary material for: pRB-Depleted Pluripotent Stem Cell Retinal Organoids Recapitulate Cell State Transitions of Retinoblastoma Development and Suggest an Important Role for pRB in Retinal Cell Differentiation
Source: Stem Cells Transl Med. 2022 Mar 23;11(4):415–33. doi: 10.1093/stcltm/szac008 (PMC9052432; doi:10.1093/stcltm/szac008)
Supplement: szac008_suppl_Supplementary_Table_S3 [file szac008_suppl_supplementary_table_s3.docx]

|  | **p_val** |  | **avg_logFC** | **pct.1** | **pct.2** | **p_val_adj** | **cluster** | ***gene*** | **predicted cell fate** |
| --- | --- | --- | --- | --- | --- | --- | --- | --- | --- |
| NRL |  | 0 | 1.231339 | 0.93 | 0.357 | 0 | 0 | *NRL* | Photoreceptor precursors |
| PDC |  | 0 | 1.19397 | 0.981 | 0.424 | 0 | 0 | *PDC* | Photoreceptor precursors |
| PIK3R1 |  | 0 | 1.152838 | 0.945 | 0.472 | 0 | 0 | *PIK3R1* | Photoreceptor precursors |
| SLC38A5 |  | 0 | 1.093884 | 0.922 | 0.217 | 0 | 0 | *SLC38A5* | Photoreceptor precursors |
| UNC119 |  | 0 | 1.079561 | 0.977 | 0.467 | 0 | 0 | *UNC119* | Photoreceptor precursors |
| AMER2 |  | 0 | 1.067305 | 0.945 | 0.308 | 0 | 0 | *AMER2* | Photoreceptor precursors |
| NEUROD1 |  | 0 | 1.05285 | 0.949 | 0.26 | 0 | 0 | *NEUROD1* | Photoreceptor precursors |
| GNB3 |  | 0 | 1.035498 | 0.964 | 0.321 | 0 | 0 | *GNB3* | Photoreceptor precursors |
| RRAD |  | 0 | 1.002915 | 0.864 | 0.291 | 0 | 0 | *RRAD* | Photoreceptor precursors |
| NEUROD4 |  | 0 | 0.98792 | 0.865 | 0.182 | 0 | 0 | *NEUROD4* | Photoreceptor precursors |
| SCG3 |  | 0 | 0.983498 | 0.976 | 0.372 | 0 | 0 | *SCG3* | Photoreceptor precursors |
| PCBP4 |  | 0 | 0.947646 | 0.958 | 0.356 | 0 | 0 | *PCBP4* | Photoreceptor precursors |
| OTX2 |  | 0 | 0.937902 | 0.92 | 0.229 | 0 | 0 | *OTX2* | Photoreceptor precursors |
| AKAP9 |  | 0 | 0.937294 | 0.983 | 0.601 | 0 | 0 | *AKAP9* | Photoreceptor precursors |
| RCVRN |  | 0 | 0.930568 | 0.816 | 0.321 | 0 | 0 | *RCVRN* | Photoreceptor precursors |
| CRX |  | 0 | 0.920603 | 0.934 | 0.238 | 0 | 0 | *CRX* | Photoreceptor precursors |
| LINC00599 |  | 0 | 0.904502 | 0.877 | 0.196 | 0 | 0 | *LINC00599* | Photoreceptor precursors |
| SLC38A1 |  | 0 | 0.885392 | 0.946 | 0.331 | 0 | 0 | *SLC38A1* | Photoreceptor precursors |
| SYP |  | 0 | 0.88488 | 0.946 | 0.245 | 0 | 0 | *SYP* | Photoreceptor precursors |
| CADPS |  | 0 | 0.850777 | 0.869 | 0.192 | 0 | 0 | *CADPS* | Photoreceptor precursors |
| Sep-04 |  | 0 | 0.849347 | 0.912 | 0.295 | 0 | 0 | *Sep-04* | Photoreceptor precursors |
| FAM57B |  | 0 | 0.838777 | 0.928 | 0.278 | 0 | 0 | *FAM57B* | Photoreceptor precursors |
| AIPL1 |  | 0 | 0.838637 | 0.828 | 0.25 | 0 | 0 | *AIPL1* | Photoreceptor precursors |
| TP53INP2 |  | 0 | 0.833221 | 0.839 | 0.192 | 0 | 0 | *TP53INP2* | Photoreceptor precursors |
| VSX1 |  | 0 | 0.802254 | 0.508 | 0.094 | 0 | 0 | *VSX1* | Photoreceptor precursors |
| EPB41 |  | 0 | 0.794477 | 0.939 | 0.385 | 0 | 0 | *EPB41* | Photoreceptor precursors |
| PKM |  | 0 | 0.783468 | 0.991 | 0.817 | 0 | 0 | *PKM* | Photoreceptor precursors |
| NEUROG1 |  | 0 | 0.766491 | 0.761 | 0.127 | 0 | 0 | *NEUROG1* | Photoreceptor precursors |
| ANKRD33B |  | 0 | 0.751567 | 0.822 | 0.156 | 0 | 0 | *ANKRD33B* | Photoreceptor precursors |
| CHODL |  | 0 | 0.748672 | 0.54 | 0.096 | 0 | 0 | *CHODL* | Photoreceptor precursors |
| MAP2 |  | 0 | 0.743024 | 0.905 | 0.406 | 0 | 0 | *MAP2* | Photoreceptor precursors |
| DPYSL3 |  | 0 | 0.737999 | 0.829 | 0.208 | 0 | 0 | *DPYSL3* | Photoreceptor precursors |
| ZNF326 |  | 0 | 0.736188 | 0.905 | 0.383 | 0 | 0 | *ZNF326* | Photoreceptor precursors |
| PDE6H | 2.17E-253 | | 0.735721 | 0.735 | 0.382 | 4.65E-249 | 0 | *PDE6H* | Photoreceptor precursors |
| CPE |  | 0 | 0.734328 | 0.975 | 0.532 | 0 | 0 | *CPE* | Photoreceptor precursors |
| DCT |  | 0 | 0.725471 | 0.673 | 0.14 | 0 | 0 | *DCT* | Photoreceptor precursors |
| MAP1LC3A |  | 0 | 0.725403 | 0.884 | 0.303 | 0 | 0 | *MAP1LC3A* | Photoreceptor precursors |
| IMPG2 |  | 0 | 0.72378 | 0.805 | 0.169 | 0 | 0 | *IMPG2* | Photoreceptor precursors |
| PRDX1 |  | 0 | 0.722641 | 0.984 | 0.804 | 0 | 0 | *PRDX1* | Photoreceptor precursors |
| HOPX |  | 0 | 0.717216 | 0.497 | 0.071 | 0 | 0 | *HOPX* | Photoreceptor precursors |
| CRYBG3 |  | 0 | 0.715508 | 0.708 | 0.229 | 0 | 0 | *CRYBG3* | Photoreceptor precursors |
| TUBA4A |  | 0 | 0.712157 | 0.795 | 0.244 | 0 | 0 | *TUBA4A* | Photoreceptor precursors |
| TULP1 |  | 0 | 0.695866 | 0.829 | 0.205 | 0 | 0 | *TULP1* | Photoreceptor precursors |
| NLK |  | 0 | 0.677733 | 0.798 | 0.179 | 0 | 0 | *NLK* | Photoreceptor precursors |
| SPCS1 |  | 0 | 0.675469 | 0.985 | 0.765 | 0 | 0 | *SPCS1* | Photoreceptor precursors |
| PCP2 |  | 0 | 0.675109 | 0.318 | 0.027 | 0 | 0 | *PCP2* | Photoreceptor precursors |
| NFIB |  | 0 | 0.670665 | 0.835 | 0.408 | 0 | 0 | *NFIB* | Photoreceptor precursors |
| GUK1 |  | 0 | 0.668627 | 0.972 | 0.702 | 0 | 0 | *GUK1* | Photoreceptor precursors |
| PTP4A3 |  | 0 | 0.665927 | 0.844 | 0.363 | 0 | 0 | *PTP4A3* | Photoreceptor precursors |
| AGAP1 |  | 0 | 0.663503 | 0.819 | 0.23 | 0 | 0 | *AGAP1* | Photoreceptor precursors |
| ROM1 |  | 0 | 0.658231 | 0.676 | 0.19 | 0 | 0 | *ROM1* | Photoreceptor precursors |
| COBLL1 |  | 0 | 0.649509 | 0.753 | 0.173 | 0 | 0 | *COBLL1* | Photoreceptor precursors |
| CRABP2 | 2.49E-157 | | 0.644984 | 0.803 | 0.566 | 5.34E-153 | 0 | *CRABP2* | Photoreceptor precursors |
| ENO2 |  | 0 | 0.636784 | 0.931 | 0.461 | 0 | 0 | *ENO2* | Photoreceptor precursors |
| GADD45G |  | 0 | 0.6355 | 0.867 | 0.321 | 0 | 0 | *GADD45G* | Photoreceptor precursors |
| PDE1C |  | 0 | 0.633569 | 0.704 | 0.129 | 0 | 0 | *PDE1C* | Photoreceptor precursors |
| RXRG |  | 0 | 0.628684 | 0.749 | 0.173 | 0 | 0 | *RXRG* | Photoreceptor precursors |
| TMX4 |  | 0 | 0.624363 | 0.816 | 0.391 | 0 | 0 | *TMX4* | Photoreceptor precursors |
| MLXIP |  | 0 | 0.622043 | 0.705 | 0.172 | 0 | 0 | *MLXIP* | Photoreceptor precursors |
| AANAT |  | 0 | 0.62181 | 0.761 | 0.169 | 0 | 0 | *AANAT* | Photoreceptor precursors |
| SLC1A2 |  | 0 | 0.616899 | 0.746 | 0.152 | 0 | 0 | *SLC1A2* | Photoreceptor precursors |
| GNGT2 |  | 0 | 0.612777 | 0.677 | 0.191 | 0 | 0 | *GNGT2* | Photoreceptor precursors |

| STX3 | 0 | 0.612157 | 0.785 | 0.196 | 0 | 0 | *STX3* | Photoreceptor precursors |
| --- | --- | --- | --- | --- | --- | --- | --- | --- |
| NR2E3 | 0 | 0.61108 | 0.607 | 0.104 | 0 | 0 | *NR2E3* | Photoreceptor precursors |
| DST | 9.95E-277 | 0.608418 | 0.767 | 0.391 | 2.13E-272 | 0 | *DST* | Photoreceptor precursors |
| FSTL5 | 0 | 0.605577 | 0.744 | 0.168 | 0 | 0 | *FSTL5* | Photoreceptor precursors |
| RP1 | 0 | 0.596802 | 0.623 | 0.135 | 0 | 0 | *RP1* | Photoreceptor precursors |
| MPP4 | 0 | 0.59576 | 0.659 | 0.152 | 0 | 0 | *MPP4* | Photoreceptor precursors |
| SEZ6L2 | 0 | 0.586379 | 0.826 | 0.262 | 0 | 0 | *SEZ6L2* | Photoreceptor precursors |
| MEIS2 | 0 | 0.58395 | 0.859 | 0.348 | 0 | 0 | *MEIS2* | Photoreceptor precursors |
| SLC17A7 | 0 | 0.579393 | 0.712 | 0.144 | 0 | 0 | *SLC17A7* | Photoreceptor precursors |
| UGCG | 0 | 0.57422 | 0.752 | 0.242 | 0 | 0 | *UGCG* | Photoreceptor precursors |
| SYT1 | 0 | 0.573056 | 0.948 | 0.616 | 0 | 0 | *SYT1* | Photoreceptor precursors |
| CADM3 | 0 | 0.572605 | 0.696 | 0.193 | 0 | 0 | *CADM3* | Photoreceptor precursors |
| TPH1 | 0 | 0.567034 | 0.439 | 0.065 | 0 | 0 | *TPH1* | Photoreceptor precursors |
| SCARB2 | 0 | 0.558988 | 0.928 | 0.563 | 0 | 0 | *SCARB2* | Photoreceptor precursors |
| ATP1B2 | 0 | 0.554308 | 0.845 | 0.434 | 0 | 0 | *ATP1B2* | Photoreceptor precursors |
| RICTOR | 0 | 0.550224 | 0.654 | 0.187 | 0 | 0 | *RICTOR* | Photoreceptor precursors |
| TUBB4B | 4.57E-305 | 0.54974 | 0.972 | 0.737 | 9.80E-301 | 0 | *TUBB4B* | Photoreceptor precursors |
| PEX5L | 0 | 0.548615 | 0.638 | 0.132 | 0 | 0 | *PEX5L* | Photoreceptor precursors |
| ALDOC | 0 | 0.543028 | 0.649 | 0.199 | 0 | 0 | *ALDOC* | Photoreceptor precursors |
| PTPN13 | 0 | 0.536431 | 0.692 | 0.208 | 0 | 0 | *PTPN13* | Photoreceptor precursors |
| CADM2 | 0 | 0.53628 | 0.774 | 0.256 | 0 | 0 | *CADM2* | Photoreceptor precursors |
| GUCA1A | 1.79E-152 | 0.53613 | 0.549 | 0.274 | 3.83E-148 | 0 | *GUCA1A* | Photoreceptor precursors |
| KIF1B | 0 | 0.532549 | 0.831 | 0.364 | 0 | 0 | *KIF1B* | Photoreceptor precursors |
| CTBS | 0 | 0.529741 | 0.711 | 0.22 | 0 | 0 | *CTBS* | Photoreceptor precursors |
| MAP1B | 5.92E-306 | 0.529621 | 0.993 | 0.855 | 1.27E-301 | 0 | *MAP1B* | Photoreceptor precursors |
| ATP2B1 | 0 | 0.5281 | 0.83 | 0.366 | 0 | 0 | *ATP2B1* | Photoreceptor precursors |
| KCNB1 | 0 | 0.524364 | 0.659 | 0.133 | 0 | 0 | *KCNB1* | Photoreceptor precursors |
| HRASLS | 0 | 0.523486 | 0.661 | 0.176 | 0 | 0 | *HRASLS* | Photoreceptor precursors |
| CLCN3 | 0 | 0.517284 | 0.852 | 0.419 | 0 | 0 | *CLCN3* | Photoreceptor precursors |
| VXN | 0 | 0.515381 | 0.558 | 0.146 | 0 | 0 | *VXN* | Photoreceptor precursors |
| KCNK3 | 0 | 0.510221 | 0.619 | 0.103 | 0 | 0 | *KCNK3* | Photoreceptor precursors |
| MIR124-2H | 0 | 0.509691 | 0.663 | 0.17 | 0 | 0 | *MIR124-2H* | Photoreceptor precursors |
| VAMP2 | 4.47E-308 | 0.503517 | 0.957 | 0.681 | 9.58E-304 | 0 | *VAMP2* | Photoreceptor precursors |
| IFITM2 | 0 | 0.752199 | 0.879 | 0.397 | 0 | 1 | *IFITM2* | Late RPCs |
| SPP1 | 0 | 0.729091 | 0.991 | 0.65 | 0 | 1 | *SPP1* | Late RPCs |
| TF | 6.38E-146 | 0.71824 | 0.655 | 0.372 | 1.37E-141 | 1 | *TF* | Late RPCs |
| FOS | 7.23E-245 | 0.710767 | 0.94 | 0.646 | 1.55E-240 | 1 | *FOS* | Late RPCs |
| ZFP36L2 | 0 | 0.696794 | 0.857 | 0.422 | 0 | 1 | *ZFP36L2* | Late RPCs |
| IFITM3 | 3.31E-304 | 0.643173 | 0.805 | 0.353 | 7.09E-300 | 1 | *IFITM3* | Late RPCs |
| TTYH1 | 0 | 0.639091 | 0.873 | 0.428 | 0 | 1 | *TTYH1* | Late RPCs |
| ZFP36L1 | 1.06E-289 | 0.628551 | 0.921 | 0.515 | 2.27E-285 | 1 | *ZFP36L1* | Late RPCs |
| DKK3 | 1.22E-276 | 0.614933 | 0.902 | 0.514 | 2.62E-272 | 1 | *DKK3* | Late RPCs |
| SOX2 | 4.42E-303 | 0.5998 | 0.873 | 0.427 | 9.47E-299 | 1 | *SOX2* | Late RPCs |
| EGR1 | 5.99E-215 | 0.560439 | 0.96 | 0.664 | 1.28E-210 | 1 | *EGR1* | Late RPCs |
| GPM6B | 3.97E-254 | 0.540315 | 0.898 | 0.542 | 8.51E-250 | 1 | *GPM6B* | Late RPCs |
| HES1 | 1.61E-176 | 0.538783 | 0.83 | 0.476 | 3.45E-172 | 1 | *HES1* | Late RPCs |
| BAALC | 4.11E-256 | 0.535874 | 0.735 | 0.333 | 8.82E-252 | 1 | *BAALC* | Late RPCs |
| CYP26A1 | 1.44E-155 | 0.535775 | 0.838 | 0.54 | 3.08E-151 | 1 | *CYP26A1* | Late RPCs |
| PLEKHA1 | 4.54E-208 | 0.532692 | 0.9 | 0.609 | 9.73E-204 | 1 | *PLEKHA1* | Late RPCs |
| QDPR | 3.60E-188 | 0.529167 | 0.618 | 0.291 | 7.71E-184 | 1 | *QDPR* | Late RPCs |
| RTN4 | 4.42E-297 | 0.527128 | 1 | 0.961 | 9.48E-293 | 1 | *RTN4* | Late RPCs |
| DIO3 | 8.54E-201 | 0.526553 | 0.676 | 0.316 | 1.83E-196 | 1 | *DIO3* | Late RPCs |
| DCBLD2 | 9.74E-244 | 0.524664 | 0.785 | 0.407 | 2.09E-239 | 1 | *DCBLD2* | Late RPCs |
| PTPRZ1 | 2.46E-259 | 0.521534 | 0.755 | 0.334 | 5.27E-255 | 1 | *PTPRZ1* | Late RPCs |
| VIM | 3.42E-252 | 0.519064 | 1 | 0.847 | 7.34E-248 | 1 | *VIM* | Late RPCs |
| CLU | 7.93E-245 | 0.512909 | 0.99 | 0.738 | 1.70E-240 | 1 | *CLU* | Late RPCs |
| SLC16A1 | 1.11E-141 | 0.51275 | 0.743 | 0.48 | 2.38E-137 | 1 | *SLC16A1* | Late RPCs |
| NES | 1.87E-227 | 0.505081 | 0.621 | 0.258 | 4.01E-223 | 1 | *NES* | Late RPCs |
| MGARP | 2.52E-263 | 0.500844 | 0.75 | 0.34 | 5.41E-259 | 1 | *MGARP* | Late RPCs |
| TYMS | 0 | 0.732316 | 0.851 | 0.312 | 0 | 2 | *TYMS* | Late RPCs |
| HELLS | 0 | 0.647378 | 0.78 | 0.239 | 0 | 2 | *HELLS* | Late RPCs |
| FAM111B | 0 | 0.640595 | 0.699 | 0.156 | 0 | 2 | *FAM111B* | Late RPCs |
| PCNA | 0 | 0.636452 | 0.829 | 0.307 | 0 | 2 | *PCNA* | Late RPCs |

| PCLAF | 0 | 0.634017 | 0.707 | 0.191 | 0 | 2 | *PCLAF* | Late RPCs |
| --- | --- | --- | --- | --- | --- | --- | --- | --- |
| SLBP | 0 | 0.618676 | 0.746 | 0.271 | 0 | 2 | *SLBP* | Late RPCs |
| NASP | 0 | 0.596728 | 0.936 | 0.562 | 0 | 2 | *NASP* | Late RPCs |
| DUT | 5.91E-277 | 0.55089 | 0.841 | 0.456 | 1.27E-272 | 2 | *DUT* | Late RPCs |
| LGALS3 | 1.50E-172 | 0.537178 | 0.329 | 0.098 | 3.21E-168 | 2 | *LGALS3* | Late RPCs |
| TMEM106 | 5.24E-239 | 0.532738 | 0.681 | 0.314 | 1.12E-234 | 2 | *TMEM106* | Late RPCs |
| TUBA1B | 5.15E-306 | 0.529129 | 0.997 | 0.884 | 1.10E-301 | 2 | *TUBA1B* | Late RPCs |
| PLEKHA11 | 8.60E-216 | 0.521875 | 0.913 | 0.609 | 1.84E-211 | 2 | *PLEKHA1* | Late RPCs |
| GINS2 | 0 | 0.52106 | 0.663 | 0.166 | 0 | 2 | *GINS2* | Late RPCs |
| PMAIP1 | 3.36E-222 | 0.509571 | 0.627 | 0.258 | 7.20E-218 | 2 | *PMAIP1* | Late RPCs |
| PRSS23 | 1.28E-208 | 0.507516 | 0.783 | 0.41 | 2.74E-204 | 2 | *PRSS23* | Late RPCs |
| NRL1 | 0 | 2.021204 | 0.979 | 0.366 | 0 | 3 | *NRL* | Rod precursors |
| RCVRN1 | 0 | 1.80888 | 0.94 | 0.324 | 0 | 3 | *RCVRN* | Rod precursors |
| ROM11 | 0 | 1.56121 | 0.89 | 0.188 | 0 | 3 | *ROM1* | Rod precursors |
| PDC1 | 0 | 1.507183 | 0.994 | 0.434 | 0 | 3 | *PDC* | Rod precursors |
| UNC1191 | 0 | 1.359199 | 0.979 | 0.477 | 0 | 3 | *UNC119* | Rod precursors |
| GNAT1 | 0 | 1.356974 | 0.713 | 0.087 | 0 | 3 | *GNAT1* | Rod precursors |
| AIPL11 | 0 | 1.241557 | 0.882 | 0.258 | 0 | 3 | *AIPL1* | Rod precursors |
| RP11 | 0 | 1.060392 | 0.73 | 0.139 | 0 | 3 | *RP1* | Rod precursors |
| NR2E31 | 0 | 1.009068 | 0.709 | 0.109 | 0 | 3 | *NR2E3* | Rod precursors |
| PIK3R11 | 0 | 0.956885 | 0.876 | 0.485 | 0 | 3 | *PIK3R1* | Rod precursors |
| GNGT1 | 0 | 0.891491 | 0.462 | 0.055 | 0 | 3 | *GNGT1* | Rod precursors |
| GNB31 | 0 | 0.886652 | 0.903 | 0.337 | 0 | 3 | *GNB3* | Rod precursors |
| MAP21 | 0 | 0.867778 | 0.843 | 0.42 | 0 | 3 | *MAP2* | Rod precursors |
| CRX1 | 0 | 0.862205 | 0.816 | 0.258 | 0 | 3 | *CRX* | Rod precursors |
| TUBB4B1 | 1.80E-290 | 0.78525 | 0.96 | 0.742 | 3.86E-286 | 3 | *TUBB4B* | Rod precursors |
| ENO21 | 1.30E-294 | 0.782257 | 0.857 | 0.474 | 2.78E-290 | 3 | *ENO2* | Rod precursors |
| PDE6G | 0 | 0.782193 | 0.42 | 0.046 | 0 | 3 | *PDE6G* | Rod precursors |
| EPB41L2 | 2.56E-279 | 0.771881 | 0.669 | 0.282 | 5.50E-275 | 3 | *EPB41L2* | Rod precursors |
| GNGT21 | 0 | 0.767769 | 0.705 | 0.199 | 0 | 3 | *GNGT2* | Rod precursors |
| CPLX4 | 0 | 0.750369 | 0.612 | 0.145 | 0 | 3 | *CPLX4* | Rod precursors |
| SAG | 0 | 0.748089 | 0.298 | 0.038 | 0 | 3 | *SAG* | Rod precursors |
| PSIP1 | 0 | 0.747958 | 0.993 | 0.856 | 0 | 3 | *PSIP1* | Rod precursors |
| PKM1 | 1.06E-292 | 0.743223 | 0.983 | 0.821 | 2.27E-288 | 3 | *PKM* | Rod precursors |
| ALDOC1 | 0 | 0.728769 | 0.647 | 0.208 | 0 | 3 | *ALDOC* | Rod precursors |
| CPE1 | 1.13E-269 | 0.705782 | 0.882 | 0.545 | 2.42E-265 | 3 | *CPE* | Rod precursors |
| PCBP41 | 0 | 0.705729 | 0.834 | 0.374 | 0 | 3 | *PCBP4* | Rod precursors |
| GUCA1A1 | 1.67E-166 | 0.677131 | 0.585 | 0.277 | 3.57E-162 | 3 | *GUCA1A* | Rod precursors |
| PRDX11 | 1.40E-271 | 0.676958 | 0.972 | 0.808 | 2.99E-267 | 3 | *PRDX1* | Rod precursors |
| MT1X | 8.93E-255 | 0.676199 | 0.474 | 0.136 | 1.91E-250 | 3 | *MT1X* | Rod precursors |
| AKAP91 | 4.30E-278 | 0.67459 | 0.951 | 0.61 | 9.22E-274 | 3 | *AKAP9* | Rod precursors |
| GPR160 | 0 | 0.653871 | 0.495 | 0.064 | 0 | 3 | *GPR160* | Rod precursors |
| TULP11 | 0 | 0.652334 | 0.706 | 0.224 | 0 | 3 | *TULP1* | Rod precursors |
| Sep-41 | 2.32E-278 | 0.645418 | 0.763 | 0.315 | 4.97E-274 | 3 | *Sep-04* | Rod precursors |
| LAPTM4B | 8.97E-229 | 0.640415 | 0.792 | 0.482 | 1.92E-224 | 3 | *LAPTM4B* | Rod precursors |
| MAP1LC3B | 2.42E-175 | 0.608497 | 0.755 | 0.498 | 5.20E-171 | 3 | *MAP1LC3B* | Rod precursors |
| FAM57B1 | 2.46E-248 | 0.59466 | 0.732 | 0.301 | 5.27E-244 | 3 | *FAM57B* | Rod precursors |
| CADM1 | 4.42E-136 | 0.585365 | 0.799 | 0.611 | 9.47E-132 | 3 | *CADM1* | Rod precursors |
| SLC38A51 | 5.02E-216 | 0.584296 | 0.637 | 0.246 | 1.08E-211 | 3 | *SLC38A5* | Rod precursors |
| NEUROD11 | 8.85E-236 | 0.584001 | 0.714 | 0.286 | 1.90E-231 | 3 | *NEUROD1* | Rod precursors |
| EPS8 | 7.03E-235 | 0.570677 | 0.579 | 0.218 | 1.51E-230 | 3 | *EPS8* | Rod precursors |
| YPEL2 | 0 | 0.560879 | 0.472 | 0.1 | 0 | 3 | *YPEL2* | Rod precursors |
| IMPG21 | 4.16E-262 | 0.557903 | 0.592 | 0.193 | 8.92E-258 | 3 | *IMPG2* | Rod precursors |
| GSTP1 | 8.94E-196 | 0.546738 | 0.994 | 0.958 | 1.92E-191 | 3 | *GSTP1* | Rod precursors |
| RBP3 | 0 | 0.539908 | 0.505 | 0.125 | 0 | 3 | *RBP3* | Rod precursors |
| SEC62 | 9.53E-177 | 0.53913 | 0.908 | 0.798 | 2.04E-172 | 3 | *SEC62* | Rod precursors |
| RHO | 0 | 0.532233 | 0.188 | 0.015 | 0 | 3 | *RHO* | Rod precursors |
| CABP5 | 0 | 0.530773 | 0.345 | 0.047 | 0 | 3 | *CABP5* | Rod precursors |
| PTPN131 | 2.24E-194 | 0.523326 | 0.559 | 0.224 | 4.80E-190 | 3 | *PTPN13* | Rod precursors |
| SYP1 | 1.76E-195 | 0.521545 | 0.651 | 0.274 | 3.78E-191 | 3 | *SYP* | Rod precursors |
| STX31 | 9.55E-236 | 0.517729 | 0.595 | 0.217 | 2.05E-231 | 3 | *STX3* | Rod precursors |
| KCNV2 | 1.54E-259 | 0.51447 | 0.479 | 0.131 | 3.30E-255 | 3 | *KCNV2* | Rod precursors |
| AANAT1 | 2.48E-209 | 0.506187 | 0.542 | 0.192 | 5.31E-205 | 3 | *AANAT* | Rod precursors |

| SCG31 | 6.18E-176 | 0.502982 | 0.791 | 0.394 | 1.33E-171 | 3 | *SCG3* | Rod precursors |
| --- | --- | --- | --- | --- | --- | --- | --- | --- |
| SPP11 | 2.33E-283 | 0.710784 | 0.981 | 0.655 | 5.01E-279 | 4 | *SPP1* | Late RPCs |
| ZFP36L21 | 1.39E-171 | 0.566187 | 0.786 | 0.431 | 2.98E-167 | 4 | *ZFP36L2* | Late RPCs |
| DIO31 | 1.04E-127 | 0.55453 | 0.624 | 0.323 | 2.23E-123 | 4 | *DIO3* | Late RPCs |
| IFITM21 | 1.04E-156 | 0.548891 | 0.765 | 0.41 | 2.22E-152 | 4 | *IFITM2* | Late RPCs |
| PRSS231 | 1.71E-145 | 0.527035 | 0.745 | 0.416 | 3.67E-141 | 4 | *PRSS23* | Late RPCs |
| PDE6H1 | 0 | 1.915708 | 0.975 | 0.377 | 0 | 5 | *PDE6H* | Cone precursors |
| RRAD1 | 0 | 1.31321 | 0.931 | 0.3 | 0 | 5 | *RRAD* | Cone precursors |
| GUCA1A2 | 0 | 1.286927 | 0.878 | 0.263 | 0 | 5 | *GUCA1A* | Cone precursors |
| AIPL12 | 0 | 1.193186 | 0.969 | 0.255 | 0 | 5 | *AIPL1* | Cone precursors |
| UNC1192 | 0 | 1.149794 | 0.985 | 0.477 | 0 | 5 | *UNC119* | Cone precursors |
| GNB32 | 0 | 1.140409 | 0.973 | 0.334 | 0 | 5 | *GNB3* | Cone precursors |
| AKAP92 | 0 | 1.127732 | 0.997 | 0.608 | 0 | 5 | *AKAP9* | Cone precursors |
| GUK11 | 0 | 1.120358 | 0.994 | 0.706 | 0 | 5 | *GUK1* | Cone precursors |
| GNGT22 | 0 | 1.076628 | 0.855 | 0.192 | 0 | 5 | *GNGT2* | Cone precursors |
| RCVRN2 | 0 | 1.07461 | 0.894 | 0.327 | 0 | 5 | *RCVRN* | Cone precursors |
| SCG32 | 0 | 1.047959 | 0.976 | 0.385 | 0 | 5 | *SCG3* | Cone precursors |
| FAM57B2 | 0 | 1.01861 | 0.963 | 0.29 | 0 | 5 | *FAM57B* | Cone precursors |
| PDC2 | 0 | 0.995023 | 0.995 | 0.435 | 0 | 5 | *PDC* | Cone precursors |
| NRN1 | 0 | 0.982815 | 0.774 | 0.151 | 0 | 5 | *NRN1* | Cone precursors |
| TULP12 | 0 | 0.953862 | 0.926 | 0.214 | 0 | 5 | *TULP1* | Cone precursors |
| CRABP21 | 4.17E-264 | 0.940108 | 0.902 | 0.566 | 8.93E-260 | 5 | *CRABP2* | Cone precursors |
| MPP41 | 0 | 0.916836 | 0.86 | 0.152 | 0 | 5 | *MPP4* | Cone precursors |
| TUBA4A1 | 0 | 0.904313 | 0.873 | 0.251 | 0 | 5 | *TUBA4A* | Cone precursors |
| ENO22 | 0 | 0.893382 | 0.971 | 0.469 | 0 | 5 | *ENO2* | Cone precursors |
| Sep-42 | 0 | 0.854269 | 0.922 | 0.308 | 0 | 5 | *Sep-04* | Cone precursors |
| PPP1CC | 0 | 0.852108 | 0.88 | 0.402 | 0 | 5 | *PPP1CC* | Cone precursors |
| VXN1 | 0 | 0.842359 | 0.66 | 0.15 | 0 | 5 | *VXN* | Cone precursors |
| ARR3 | 0 | 0.804781 | 0.563 | 0.112 | 0 | 5 | *ARR3* | Cone precursors |
| MAP22 | 0 | 0.791886 | 0.928 | 0.416 | 0 | 5 | *MAP2* | Cone precursors |
| FSTL51 | 0 | 0.785162 | 0.842 | 0.176 | 0 | 5 | *FSTL5* | Cone precursors |
| PCBP42 | 0 | 0.783115 | 0.946 | 0.369 | 0 | 5 | *PCBP4* | Cone precursors |
| CPE2 | 0 | 0.761408 | 0.975 | 0.541 | 0 | 5 | *CPE* | Cone precursors |
| DPYSL31 | 0 | 0.753884 | 0.852 | 0.22 | 0 | 5 | *DPYSL3* | Cone precursors |
| PLEKHB1 | 0 | 0.748529 | 0.827 | 0.206 | 0 | 5 | *PLEKHB1* | Cone precursors |
| NME1 | 0 | 0.741048 | 0.893 | 0.452 | 0 | 5 | *NME1* | Cone precursors |
| ARF4 | 1.44E-242 | 0.740949 | 0.829 | 0.49 | 3.08E-238 | 5 | *ARF4* | Cone precursors |
| PRDX12 | 3.02E-269 | 0.732337 | 0.974 | 0.808 | 6.48E-265 | 5 | *PRDX1* | Cone precursors |
| RABAC1 | 2.93E-286 | 0.72397 | 0.87 | 0.485 | 6.28E-282 | 5 | *RABAC1* | Cone precursors |
| MAP1LC3A | 0 | 0.723639 | 0.882 | 0.316 | 0 | 5 | *MAP1LC3A* | Cone precursors |
| CRX2 | 0 | 0.720515 | 0.89 | 0.255 | 0 | 5 | *CRX* | Cone precursors |
| MYL4 | 0 | 0.708166 | 0.662 | 0.121 | 0 | 5 | *MYL4* | Cone precursors |
| AANAT2 | 0 | 0.701667 | 0.782 | 0.18 | 0 | 5 | *AANAT* | Cone precursors |
| RS1 | 0 | 0.701381 | 0.713 | 0.103 | 0 | 5 | *RS1* | Cone precursors |
| YIF1A | 0 | 0.692391 | 0.776 | 0.319 | 0 | 5 | *YIF1A* | Cone precursors |
| KCNV21 | 0 | 0.690614 | 0.723 | 0.119 | 0 | 5 | *KCNV2* | Cone precursors |
| IMPG22 | 0 | 0.686681 | 0.801 | 0.183 | 0 | 5 | *IMPG2* | Cone precursors |
| VAMP21 | 0 | 0.679148 | 0.974 | 0.686 | 0 | 5 | *VAMP2* | Cone precursors |
| ALDOC2 | 0 | 0.672265 | 0.729 | 0.205 | 0 | 5 | *ALDOC* | Cone precursors |
| CC2D2A | 0 | 0.671124 | 0.731 | 0.169 | 0 | 5 | *CC2D2A* | Cone precursors |
| NEDD4L | 0 | 0.66509 | 0.805 | 0.223 | 0 | 5 | *NEDD4L* | Cone precursors |
| SEZ6L21 | 0 | 0.662633 | 0.871 | 0.272 | 0 | 5 | *SEZ6L2* | Cone precursors |
| MLXIP1 | 0 | 0.658029 | 0.747 | 0.181 | 0 | 5 | *MLXIP* | Cone precursors |
| HRASLS1 | 0 | 0.657484 | 0.698 | 0.184 | 0 | 5 | *HRASLS* | Cone precursors |
| SLC38A52 | 0 | 0.652234 | 0.825 | 0.237 | 0 | 5 | *SLC38A5* | Cone precursors |
| DHRS7 | 5.99E-304 | 0.64639 | 0.849 | 0.411 | 1.29E-299 | 5 | *DHRS7* | Cone precursors |
| MIR7-3HG | 0 | 0.64255 | 0.672 | 0.146 | 0 | 5 | *MIR7-3HG* | Cone precursors |
| RXRG1 | 0 | 0.628691 | 0.763 | 0.185 | 0 | 5 | *RXRG* | Cone precursors |
| SEC61G | 7.53E-220 | 0.610186 | 0.921 | 0.723 | 1.61E-215 | 5 | *SEC61G* | Cone precursors |
| CNTNAP2 | 0 | 0.606187 | 0.738 | 0.187 | 0 | 5 | *CNTNAP2* | Cone precursors |
| SLC38A11 | 0 | 0.60579 | 0.889 | 0.347 | 0 | 5 | *SLC38A1* | Cone precursors |
| SYP2 | 0 | 0.605141 | 0.85 | 0.264 | 0 | 5 | *SYP* | Cone precursors |
| CPLX41 | 0 | 0.600017 | 0.666 | 0.143 | 0 | 5 | *CPLX4* | Cone precursors |

| SLC17A71 | 0 | 0.594418 | 0.729 | 0.155 | 0 | 5 | *SLC17A7* | Cone precursors |
| --- | --- | --- | --- | --- | --- | --- | --- | --- |
| RBP4 | 0 | 0.594325 | 0.591 | 0.071 | 0 | 5 | *RBP4* | Cone precursors |
| SLC25A6 | 1.31E-243 | 0.593366 | 0.867 | 0.505 | 2.81E-239 | 5 | *SLC25A6* | Cone precursors |
| PRCD | 0 | 0.582299 | 0.664 | 0.161 | 0 | 5 | *PRCD* | Cone precursors |
| PKIB | 0 | 0.579141 | 0.736 | 0.183 | 0 | 5 | *PKIB* | Cone precursors |
| YBX1 | 0 | 0.571545 | 0.998 | 0.98 | 0 | 5 | *YBX1* | Cone precursors |
| TPD52 | 6.73E-302 | 0.565564 | 0.778 | 0.304 | 1.44E-297 | 5 | *TPD52* | Cone precursors |
| OLFM1 | 0 | 0.565299 | 0.605 | 0.122 | 0 | 5 | *OLFM1* | Cone precursors |
| STX32 | 0 | 0.565088 | 0.772 | 0.209 | 0 | 5 | *STX3* | Cone precursors |
| FAM107A | 0 | 0.561855 | 0.632 | 0.095 | 0 | 5 | *FAM107A* | Cone precursors |
| COTL1 | 0 | 0.55952 | 0.734 | 0.257 | 0 | 5 | *COTL1* | Cone precursors |
| ATP5MC1 | 2.76E-243 | 0.558078 | 0.87 | 0.491 | 5.92E-239 | 5 | *ATP5MC1* | Cone precursors |
| PKM2 | 1.23E-214 | 0.555532 | 0.978 | 0.822 | 2.64E-210 | 5 | *PKM* | Cone precursors |
| ARL6IP5 | 3.05E-268 | 0.553555 | 0.896 | 0.48 | 6.54E-264 | 5 | *ARL6IP5* | Cone precursors |
| LMOD1 | 0 | 0.553317 | 0.633 | 0.121 | 0 | 5 | *LMOD1* | Cone precursors |
| CLSTN2 | 5.85E-188 | 0.549827 | 0.413 | 0.125 | 1.25E-183 | 5 | *CLSTN2* | Cone precursors |
| DDIT3 | 1.26E-245 | 0.541316 | 0.741 | 0.311 | 2.70E-241 | 5 | *DDIT3* | Cone precursors |
| ABHD14A | 0 | 0.537982 | 0.734 | 0.245 | 0 | 5 | *ABHD14A* | Cone precursors |
| SOX7 | 0 | 0.537971 | 0.54 | 0.068 | 0 | 5 | *SOX7* | Cone precursors |
| KIF2A | 2.51E-223 | 0.537201 | 0.832 | 0.438 | 5.38E-219 | 5 | *KIF2A* | Cone precursors |
| PEX5L1 | 0 | 0.536347 | 0.635 | 0.143 | 0 | 5 | *PEX5L* | Cone precursors |
| IGSF21 | 0 | 0.53535 | 0.654 | 0.119 | 0 | 5 | *IGSF21* | Cone precursors |
| COX17 | 2.95E-239 | 0.533211 | 0.815 | 0.408 | 6.33E-235 | 5 | *COX17* | Cone precursors |
| AGAP11 | 0 | 0.532576 | 0.729 | 0.247 | 0 | 5 | *AGAP1* | Cone precursors |
| IFI27L2 | 2.45E-257 | 0.529216 | 0.839 | 0.408 | 5.25E-253 | 5 | *IFI27L2* | Cone precursors |
| ATP6V0B | 3.98E-227 | 0.52596 | 0.928 | 0.628 | 8.54E-223 | 5 | *ATP6V0B* | Cone precursors |
| SERF2 | 1.62E-158 | 0.52449 | 0.967 | 0.93 | 3.48E-154 | 5 | *SERF2* | Cone precursors |
| TTC3 | 2.44E-188 | 0.521339 | 0.968 | 0.849 | 5.24E-184 | 5 | *TTC3* | Cone precursors |
| LINC00599 | 0 | 0.520217 | 0.744 | 0.218 | 0 | 5 | *LINC00599* | Cone precursors |
| LAPTM4B1 | 2.10E-240 | 0.519708 | 0.878 | 0.478 | 4.50E-236 | 5 | *LAPTM4B* | Cone precursors |
| UQCC2 | 2.35E-227 | 0.518508 | 0.871 | 0.481 | 5.05E-223 | 5 | *UQCC2* | Cone precursors |
| SYT11 | 3.92E-211 | 0.517134 | 0.949 | 0.623 | 8.41E-207 | 5 | *SYT1* | Cone precursors |
| ARF1 | 5.64E-206 | 0.516098 | 0.876 | 0.552 | 1.21E-201 | 5 | *ARF1* | Cone precursors |
| PRMT1 | 1.31E-222 | 0.515735 | 0.927 | 0.597 | 2.81E-218 | 5 | *PRMT1* | Cone precursors |
| GOLGA2 | 5.66E-273 | 0.515418 | 0.664 | 0.238 | 1.21E-268 | 5 | *GOLGA2* | Cone precursors |
| SSR4 | 1.36E-201 | 0.514808 | 0.923 | 0.687 | 2.92E-197 | 5 | *SSR4* | Cone precursors |
| CYCS | 1.99E-192 | 0.509593 | 0.9 | 0.609 | 4.26E-188 | 5 | *CYCS* | Cone precursors |
| HM13 | 1.37E-254 | 0.506454 | 0.723 | 0.299 | 2.93E-250 | 5 | *HM13* | Cone precursors |
| MAGEH1 | 3.80E-227 | 0.501781 | 0.706 | 0.31 | 8.14E-223 | 5 | *MAGEH1* | Cone precursors |
| PTP4A31 | 4.57E-193 | 0.50034 | 0.779 | 0.377 | 9.79E-189 | 5 | *PTP4A3* | Cone precursors |
| TRH | 0 | 1.595802 | 0.989 | 0.606 | 0 | 6 | *TRH* | Muller glia cells |
| TF1 | 4.93E-286 | 1.546171 | 0.765 | 0.371 | 1.06E-281 | 6 | *TF* | Muller glia cells |
| CRYM | 0 | 1.243419 | 0.937 | 0.259 | 0 | 6 | *CRYM* | Muller glia cells |
| WIF1 | 0 | 1.202516 | 0.869 | 0.24 | 0 | 6 | *WIF1* | Muller glia cells |
| SFRP2 | 0 | 1.172154 | 0.99 | 0.694 | 0 | 6 | *SFRP2* | Muller glia cells |
| LAMP5 | 0 | 1.145378 | 0.934 | 0.267 | 0 | 6 | *LAMP5* | Muller glia cells |
| GPX3 | 0 | 1.122459 | 0.616 | 0.107 | 0 | 6 | *GPX3* | Muller glia cells |
| AGL | 0 | 1.114339 | 0.916 | 0.384 | 0 | 6 | *AGL* | Muller glia cells |
| METRN | 0 | 1.067673 | 0.878 | 0.316 | 0 | 6 | *METRN* | Muller glia cells |
| CYP26A11 | 6.83E-308 | 1.03013 | 0.946 | 0.539 | 1.47E-303 | 6 | *CYP26A1* | Muller glia cells |
| VIM1 | 0 | 1.024309 | 1 | 0.85 | 0 | 6 | *VIM* | Muller glia cells |
| PAX2 | 0 | 0.935734 | 0.783 | 0.109 | 0 | 6 | *PAX2* | Muller glia cells |
| PTN | 3.90E-284 | 0.811175 | 0.872 | 0.436 | 8.35E-280 | 6 | *PTN* | Muller glia cells |
| FABP7 | 0 | 0.805557 | 0.963 | 0.525 | 0 | 6 | *FABP7* | Muller glia cells |
| DKK31 | 0 | 0.777595 | 0.947 | 0.518 | 0 | 6 | *DKK3* | Muller glia cells |
| SFRP1 | 0 | 0.768372 | 0.633 | 0.173 | 0 | 6 | *SFRP1* | Muller glia cells |
| GPC3 | 1.24E-297 | 0.72701 | 0.669 | 0.225 | 2.65E-293 | 6 | *GPC3* | Muller glia cells |
| COL18A1 | 0 | 0.70106 | 0.762 | 0.25 | 0 | 6 | *COL18A1* | Muller glia cells |
| CLU1 | 2.23E-282 | 0.685371 | 0.991 | 0.742 | 4.78E-278 | 6 | *CLU* | Muller glia cells |
| RTN41 | 6.32E-282 | 0.648436 | 0.999 | 0.962 | 1.35E-277 | 6 | *RTN4* | Muller glia cells |
| HES11 | 4.71E-223 | 0.630215 | 0.912 | 0.477 | 1.01E-218 | 6 | *HES1* | Muller glia cells |
| PLP1 | 0 | 0.629941 | 0.623 | 0.161 | 0 | 6 | *PLP1* | Muller glia cells |
| LINC01833 | 1.06E-290 | 0.62607 | 0.805 | 0.35 | 2.28E-286 | 6 | *LINC01833* | Muller glia cells |

| SRPRB | 5.89E-170 | 0.594084 | 0.564 | 0.24 | 1.26E-165 | 6 *SRPRB* | Muller glia cells |
| --- | --- | --- | --- | --- | --- | --- | --- |
| THY1 | 0 | 0.585968 | 0.64 | 0.144 | 0 | 6 *THY1* | Muller glia cells |
| TTYH11 | 1.32E-241 | 0.584085 | 0.887 | 0.434 | 2.83E-237 | 6 *TTYH1* | Muller glia cells |
| SPP12 | 4.51E-202 | 0.57244 | 0.973 | 0.656 | 9.68E-198 | 6 *SPP1* | Muller glia cells |
| NDP | 0 | 0.571944 | 0.528 | 0.075 | 0 | 6 *NDP* | Muller glia cells |
| CRABP1 | 2.45E-177 | 0.546928 | 0.997 | 0.835 | 5.26E-173 | 6 *CRABP1* | Muller glia cells |
| ANGPTL1 | 0 | 0.540368 | 0.633 | 0.173 | 0 | 6 *ANGPTL1* | Muller glia cells |
| SLC1A3 | 0 | 0.533327 | 0.503 | 0.097 | 0 | 6 *SLC1A3* | Muller glia cells |
| IFITM22 | 3.02E-163 | 0.523138 | 0.8 | 0.409 | 6.47E-159 | 6 *IFITM2* | Muller glia cells |
| SEC11C | 5.81E-198 | 0.52257 | 0.795 | 0.425 | 1.25E-193 | 6 *SEC11C* | Muller glia cells |
| CYP1B1 | 1.13E-190 | 0.508178 | 0.735 | 0.335 | 2.42E-186 | 6 *CYP1B1* | Muller glia cells |
| TF2 | 5.27E-241 | 1.187393 | 0.762 | 0.372 | 1.13E-236 | 7 *TF* | Muller glia cells |
| CYP26A12 | 0 | 1.080728 | 0.942 | 0.54 | 0 | 7 *CYP26A1* | Muller glia cells |
| TRH1 | 7.39E-262 | 0.858892 | 0.943 | 0.609 | 1.58E-257 | 7 *TRH* | Muller glia cells |
| AGL1 | 8.34E-261 | 0.780047 | 0.803 | 0.391 | 1.79E-256 | 7 *AGL* | Muller glia cells |
| SFRP21 | 1.22E-202 | 0.716288 | 0.978 | 0.695 | 2.61E-198 | 7 *SFRP2* | Muller glia cells |
| LAMP51 | 1.21E-242 | 0.674541 | 0.706 | 0.279 | 2.59E-238 | 7 *LAMP5* | Muller glia cells |
| GPC31 | 9.29E-265 | 0.670904 | 0.652 | 0.227 | 1.99E-260 | 7 *GPC3* | Muller glia cells |
| VIM2 | 1.56E-211 | 0.662259 | 0.999 | 0.85 | 3.34E-207 | 7 *VIM* | Muller glia cells |
| CRYM1 | 8.14E-246 | 0.610232 | 0.705 | 0.271 | 1.74E-241 | 7 *CRYM* | Muller glia cells |
| SPP13 | 2.72E-203 | 0.602544 | 0.974 | 0.656 | 5.83E-199 | 7 *SPP1* | Muller glia cells |
| IFITM23 | 1.14E-149 | 0.590092 | 0.755 | 0.412 | 2.44E-145 | 7 *IFITM2* | Muller glia cells |
| HES12 | 4.68E-147 | 0.587318 | 0.818 | 0.482 | 1.00E-142 | 7 *HES1* | Muller glia cells |
| FABP71 | 2.11E-166 | 0.580628 | 0.852 | 0.531 | 4.52E-162 | 7 *FABP7* | Muller glia cells |
| WIF11 | 5.54E-174 | 0.574745 | 0.609 | 0.254 | 1.19E-169 | 7 *WIF1* | Muller glia cells |
| IER2 | 1.09E-126 | 0.531333 | 0.925 | 0.74 | 2.33E-122 | 7 *IER2* | Muller glia cells |
| TTYH12 | 1.23E-170 | 0.51362 | 0.818 | 0.438 | 2.63E-166 | 7 *TTYH1* | Muller glia cells |
| HOXB5 | 9.93E-165 | 0.510326 | 0.35 | 0.095 | 2.13E-160 | 7 *HOXB5* | Muller glia cells |
| FOS1 | 7.19E-93 | 0.506688 | 0.88 | 0.654 | 1.54E-88 | 7 *FOS* | Muller glia cells |
| CYP1B11 | 0 | 1.195097 | 0.808 | 0.333 | 0 | 8 *CYP1B1* | Late RPCs |
| DAPL1 | 0 | 1.116589 | 0.966 | 0.518 | 0 | 8 *DAPL1* | Late RPCs |
| SFRP22 | 0 | 1.08359 | 0.994 | 0.695 | 0 | 8 *SFRP2* | Late RPCs |
| IGFBP5 | 1.78E-146 | 0.723207 | 0.803 | 0.466 | 3.81E-142 | 8 *IGFBP5* | Late RPCs |
| RDH10 | 0 | 0.708985 | 0.762 | 0.272 | 0 | 8 *RDH10* | Late RPCs |
| IFITM31 | 3.01E-237 | 0.67442 | 0.832 | 0.361 | 6.46E-233 | 8 *IFITM3* | Late RPCs |
| PSAT1 | 1.77E-282 | 0.660392 | 0.588 | 0.171 | 3.80E-278 | 8 *PSAT1* | Late RPCs |
| ALDH1A1 | 0 | 0.635184 | 0.678 | 0.196 | 0 | 8 *ALDH1A1* | Late RPCs |
| TSC22D1 | 1.50E-156 | 0.621016 | 0.837 | 0.515 | 3.22E-152 | 8 *TSC22D1* | Late RPCs |
| LRRC17 | 0 | 0.602154 | 0.588 | 0.088 | 0 | 8 *LRRC17* | Late RPCs |
| EFNA5 | 0 | 0.548867 | 0.595 | 0.157 | 0 | 8 *EFNA5* | Late RPCs |
| NR2F1 | 1.90E-175 | 0.53599 | 0.971 | 0.797 | 4.08E-171 | 8 *NR2F1* | Late RPCs |
| RPS12 | 5.09E-288 | 0.526566 | 1 | 0.999 | 1.09E-283 | 8 *RPS12* | Late RPCs |
| TKT | 1.10E-162 | 0.515368 | 0.82 | 0.488 | 2.36E-158 | 8 *TKT* | Late RPCs |
| RPS6 | 1.87E-290 | 0.515359 | 1 | 0.996 | 4.01E-286 | 8 *RPS6* | Late RPCs |
| FOS2 | 3.10E-114 | 0.50201 | 0.922 | 0.653 | 6.64E-110 | 8 *FOS* | Late RPCs |
| STMN2 | 0 | 1.778848 | 0.963 | 0.204 | 0 | 9 *STMN2* | Amacrine cells |
| SOX4 | 0 | 1.563219 | 0.999 | 0.857 | 0 | 9 *SOX4* | Amacrine cells |
| PAX6 | 0 | 1.351831 | 0.923 | 0.486 | 0 | 9 *PAX6* | Amacrine cells |
| MEIS21 | 2.62E-140 | 1.038239 | 0.618 | 0.374 | 5.62E-136 | 9 *MEIS2* | Amacrine cells |
| RND3 | 0 | 0.896912 | 0.567 | 0.124 | 0 | 9 *RND3* | Amacrine cells |
| MAB21L1 | 3.69E-253 | 0.888562 | 0.85 | 0.538 | 7.90E-249 | 9 *MAB21L1* | Amacrine cells |
| GRIA4 | 0 | 0.870106 | 0.651 | 0.132 | 0 | 9 *GRIA4* | Amacrine cells |
| RORB | 2.45E-263 | 0.838454 | 0.922 | 0.617 | 5.26E-259 | 9 *RORB* | Amacrine cells |
| ZFHX4 | 2.83E-285 | 0.818214 | 0.646 | 0.222 | 6.07E-281 | 9 *ZFHX4* | Amacrine cells |
| ZFHX3 | 0 | 0.811931 | 0.572 | 0.16 | 0 | 9 *ZFHX3* | Amacrine cells |
| TUBB2B | 2.29E-284 | 0.788963 | 0.945 | 0.703 | 4.91E-280 | 9 *TUBB2B* | Amacrine cells |
| XIST | 4.93E-124 | 0.777379 | 0.684 | 0.405 | 1.06E-119 | 9 *XIST* | Amacrine cells |
| CELF4 | 0 | 0.767144 | 0.695 | 0.225 | 0 | 9 *CELF4* | Amacrine cells |
| CD24 | 1.38E-271 | 0.745344 | 0.741 | 0.31 | 2.96E-267 | 9 *CD24* | Amacrine cells |
| DCX | 0 | 0.709536 | 0.593 | 0.148 | 0 | 9 *DCX* | Amacrine cells |
| BASP1 | 1.80E-228 | 0.682274 | 0.944 | 0.662 | 3.87E-224 | 9 *BASP1* | Amacrine cells |
| PCDH9 | 1.03E-124 | 0.675896 | 0.58 | 0.299 | 2.21E-120 | 9 *PCDH9* | Amacrine cells |
| MLLT11 | 1.88E-200 | 0.671339 | 0.816 | 0.511 | 4.03E-196 | 9 *MLLT11* | Amacrine cells |

| TUBA1A | 5.52E-239 | 0.669504 | 1 | 0.937 | 1.18E-234 | 9 | *TUBA1A* | Amacrine cells |
| --- | --- | --- | --- | --- | --- | --- | --- | --- |
| NSG2 | 0 | 0.662107 | 0.512 | 0.056 | 0 | 9 | *NSG2* | Amacrine cells |
| PARD3 | 6.26E-152 | 0.6476 | 0.456 | 0.168 | 1.34E-147 | 9 | *PARD3* | Amacrine cells |
| MIR181A1 | 0 | 0.647034 | 0.513 | 0.118 | 0 | 9 | *MIR181A1* | Amacrine cells |
| ZNF385D | 8.81E-199 | 0.643496 | 0.512 | 0.175 | 1.89E-194 | 9 | *ZNF385D* | Amacrine cells |
| KIF5C | 8.84E-167 | 0.642976 | 0.612 | 0.293 | 1.90E-162 | 9 | *KIF5C* | Amacrine cells |
| JPT1 | 5.31E-173 | 0.63451 | 0.777 | 0.495 | 1.14E-168 | 9 | *JPT1* | Amacrine cells |
| TFAP2A | 0 | 0.632472 | 0.324 | 0.024 | 0 | 9 | *TFAP2A* | Amacrine cells |
| TUBB2A | 5.41E-186 | 0.609203 | 0.613 | 0.262 | 1.16E-181 | 9 | *TUBB2A* | Amacrine cells |
| TMSB10 | 4.48E-166 | 0.596934 | 0.999 | 0.965 | 9.61E-162 | 9 | *TMSB10* | Amacrine cells |
| CRABP11 | 4.34E-74 | 0.592845 | 0.98 | 0.837 | 9.31E-70 | 9 | *CRABP1* | Amacrine cells |
| DPYSL2 | 7.86E-110 | 0.589436 | 0.641 | 0.416 | 1.69E-105 | 9 | *DPYSL2* | Amacrine cells |
| STMN4 | 5.04E-225 | 0.580853 | 0.52 | 0.153 | 1.08E-220 | 9 | *STMN4* | Amacrine cells |
| RUNX1T1 | 2.40E-286 | 0.580377 | 0.453 | 0.1 | 5.15E-282 | 9 | *RUNX1T1* | Amacrine cells |
| RTN1 | 0 | 0.568606 | 0.454 | 0.087 | 0 | 9 | *RTN1* | Amacrine cells |
| SOX11 | 9.30E-85 | 0.53765 | 0.659 | 0.443 | 1.99E-80 | 9 | *SOX11* | Amacrine cells |
| ELAVL3 | 4.20E-287 | 0.534141 | 0.52 | 0.127 | 8.99E-283 | 9 | *ELAVL3* | Amacrine cells |
| PCSK1N | 1.50E-140 | 0.531851 | 0.635 | 0.329 | 3.21E-136 | 9 | *PCSK1N* | Amacrine cells |
| NRXN1 | 2.36E-307 | 0.530145 | 0.445 | 0.088 | 5.06E-303 | 9 | *NRXN1* | Amacrine cells |
| CSRNP3 | 7.84E-175 | 0.527903 | 0.512 | 0.186 | 1.68E-170 | 9 | *CSRNP3* | Amacrine cells |
| GRIA2 | 0 | 0.527636 | 0.381 | 0.044 | 0 | 9 | *GRIA2* | Amacrine cells |
| CCDC88A | 6.71E-102 | 0.525446 | 0.654 | 0.428 | 1.44E-97 | 9 | *CCDC88A* | Amacrine cells |
| NREP | 1.82E-129 | 0.524266 | 0.81 | 0.598 | 3.91E-125 | 9 | *NREP* | Amacrine cells |
| NSG1 | 0 | 0.514142 | 0.423 | 0.038 | 0 | 9 | *NSG1* | Amacrine cells |
| MARCKS | 5.25E-155 | 0.504977 | 0.988 | 0.895 | 1.13E-150 | 9 | *MARCKS* | Amacrine cells |
| GADD45A | 0 | 1.281983 | 0.89 | 0.41 | 0 | 10 | *GADD45A* | NRPCs/T1 |
| HES6 | 4.16E-234 | 1.172932 | 0.923 | 0.584 | 8.93E-230 | 10 | *HES6* | NRPCs/T1 |
| IGFBP51 | 2.23E-110 | 1.146929 | 0.722 | 0.471 | 4.78E-106 | 10 | *IGFBP5* | NRPCs/T1 |
| GADD45G1 | 0 | 1.0642 | 0.881 | 0.337 | 0 | 10 | *GADD45G* | NRPCs/T1 |
| MIAT | 5.69E-258 | 0.969696 | 0.863 | 0.444 | 1.22E-253 | 10 | *MIAT* | NRPCs/T1 |
| SOX111 | 6.27E-253 | 0.845967 | 0.85 | 0.435 | 1.34E-248 | 10 | *SOX11* | NRPCs/T1 |
| RBP1 | 2.28E-263 | 0.821106 | 0.998 | 0.96 | 4.88E-259 | 10 | *RBP1* | NRPCs/T1 |
| PFN2 | 2.79E-210 | 0.761781 | 0.773 | 0.411 | 5.98E-206 | 10 | *PFN2* | NRPCs/T1 |
| RGS16 | 2.35E-157 | 0.688393 | 0.889 | 0.579 | 5.03E-153 | 10 | *RGS16* | NRPCs/T1 |
| SOX41 | 1.58E-204 | 0.639814 | 0.987 | 0.858 | 3.40E-200 | 10 | *SOX4* | NRPCs/T1 |
| BASP11 | 1.65E-185 | 0.635514 | 0.932 | 0.663 | 3.54E-181 | 10 | *BASP1* | NRPCs/T1 |
| SESN3 | 6.87E-141 | 0.634258 | 0.654 | 0.327 | 1.47E-136 | 10 | *SESN3* | NRPCs/T1 |
| ZBTB18 | 4.43E-247 | 0.613593 | 0.642 | 0.214 | 9.50E-243 | 10 | *ZBTB18* | NRPCs/T1 |
| RASD1 | 2.45E-263 | 0.594062 | 0.505 | 0.126 | 5.26E-259 | 10 | *RASD1* | NRPCs/T1 |
| RORB1 | 3.54E-141 | 0.585724 | 0.883 | 0.619 | 7.60E-137 | 10 | *RORB* | NRPCs/T1 |
| CLDN5 | 1.25E-84 | 0.561713 | 0.25 | 0.079 | 2.69E-80 | 10 | *CLDN5* | NRPCs/T1 |
| CRYBG31 | 6.84E-223 | 0.559289 | 0.678 | 0.245 | 1.47E-218 | 10 | *CRYBG3* | NRPCs/T1 |
| OTX21 | 3.21E-119 | 0.525215 | 0.583 | 0.264 | 6.87E-115 | 10 | *OTX2* | NRPCs/T1 |
| VSX11 | 1.01E-161 | 0.52242 | 0.406 | 0.111 | 2.17E-157 | 10 | *VSX1* | NRPCs/T1 |
| TFDP2 | 3.23E-118 | 0.502371 | 0.748 | 0.461 | 6.92E-114 | 10 | *TFDP2* | NRPCs/T1 |
| TCF4 | 7.96E-115 | 0.500066 | 0.805 | 0.526 | 1.71E-110 | 10 | *TCF4* | NRPCs/T1 |
| AOC2 | 0 | 1.522992 | 0.679 | 0.026 | 0 | 11 | *AOC2* | Late RPCs |
| PTGDS | 0 | 1.379522 | 0.844 | 0.198 | 0 | 11 | *PTGDS* | Late RPCs |
| COL9A1 | 0 | 1.256034 | 0.722 | 0.119 | 0 | 11 | *COL9A1* | Late RPCs |
| TSC22D11 | 0 | 1.216748 | 0.974 | 0.512 | 0 | 11 | *TSC22D1* | Late RPCs |
| TKT1 | 0 | 1.196469 | 0.977 | 0.484 | 0 | 11 | *TKT* | Late RPCs |
| ID3 | 0 | 1.167674 | 0.821 | 0.306 | 0 | 11 | *ID3* | Late RPCs |
| ITM2B | 0 | 1.078989 | 1 | 0.892 | 0 | 11 | *ITM2B* | Late RPCs |
| COL1A2 | 0 | 1.052872 | 0.946 | 0.341 | 0 | 11 | *COL1A2* | Late RPCs |
| COL9A2 | 0 | 1.032249 | 0.84 | 0.129 | 0 | 11 | *COL9A2* | Late RPCs |
| APOE | 0 | 1.016977 | 0.905 | 0.286 | 0 | 11 | *APOE* | Late RPCs |
| GNG11 | 0 | 1.015383 | 0.702 | 0.118 | 0 | 11 | *GNG11* | Late RPCs |
| PSAT11 | 0 | 0.970371 | 0.806 | 0.165 | 0 | 11 | *PSAT1* | Late RPCs |
| GJA1 | 0 | 0.956804 | 0.69 | 0.051 | 0 | 11 | *GJA1* | Late RPCs |
| EFNA51 | 0 | 0.945909 | 0.838 | 0.151 | 0 | 11 | *EFNA5* | Late RPCs |
| CPAMD8 | 0 | 0.931242 | 0.698 | 0.035 | 0 | 11 | *CPAMD8* | Late RPCs |
| LMO4 | 0 | 0.928927 | 0.807 | 0.216 | 0 | 11 | *LMO4* | Late RPCs |
| SAT1 | 2.95E-221 | 0.92083 | 0.849 | 0.438 | 6.33E-217 | 11 | *SAT1* | Late RPCs |

| 68E-246 | 0.920446 | 0.99 | 0.745 | 1.00E-241 | 11 *CLU* | Late RPCs |
| --- | --- | --- | --- | --- | --- | --- |
| 58E-219 | 0.891978 | 0.889 | 0.524 | 1.20E-214 | 11 *DAPL1* | Late RPCs |
| 0 | 0.889565 | 0.816 | 0.176 | 0 | 11 *COL9A3* | Late RPCs |
| 0 | 0.888726 | 0.709 | 0.079 | 0 | 11 *HSD17B2* | Late RPCs |
| 06E-159 | 0.877646 | 0.923 | 0.7 | 2.26E-155 | 11 *SFRP2* | Late RPCs |
| 39E-278 | 0.855307 | 0.989 | 0.797 | 1.80E-273 | 11 *NR2F1* | Late RPCs |
| 0 | 0.835937 | 0.751 | 0.1 | 0 | 11 *ZIC1* | Late RPCs |
| 0 | 0.830988 | 0.744 | 0.167 | 0 | 11 *ARL4A* | Late RPCs |
| 45E-220 | 0.825141 | 0.602 | 0.199 | 9.54E-216 | 11 *ID1* | Late RPCs |
| 0 | 0.800112 | 0.639 | 0.065 | 0 | 11 *FAM84A* | Late RPCs |
| 0 | 0.798704 | 0.834 | 0.265 | 0 | 11 *CYSTM1* | Late RPCs |
| 0 | 0.787747 | 1 | 0.999 | 0 | 11 *FTH1* | Late RPCs |
| 0 | 0.772495 | 0.744 | 0.197 | 0 | 11 *ALDH1A1* | Late RPCs |
| 0 | 0.767447 | 0.829 | 0.234 | 0 | 11 *GCHFR* | Late RPCs |
| 0 | 0.74764 | 0.539 | 0.029 | 0 | 11 *CXCL14* | Late RPCs |
| 0 | 0.729447 | 0.684 | 0.161 | 0 | 11 *PARD3* | Late RPCs |
| 21E-105 | 0.717939 | 0.65 | 0.342 | 2.60E-101 | 11 *CYP1B1* | Late RPCs |
| 0 | 0.714977 | 0.707 | 0.126 | 0 | 11 *FBN1* | Late RPCs |
| 05E-147 | 0.709619 | 0.814 | 0.443 | 4.40E-143 | 11 *PTN* | Late RPCs |
| 0 | 0.677734 | 0.425 | 0.058 | 0 | 11 *IGFBP6* | Late RPCs |
| 51E-253 | 0.672527 | 0.914 | 0.472 | 3.25E-249 | 11 *NPC2* | Late RPCs |
| 0 | 0.672247 | 0.731 | 0.213 | 0 | 11 *CDON* | Late RPCs |
| 29E-276 | 0.668109 | 0.82 | 0.32 | 7.05E-272 | 11 *MFAP2* | Late RPCs |
| 0 | 0.655359 | 0.629 | 0.135 | 0 | 11 *MFAP4* | Late RPCs |
| 0 | 0.649878 | 0.616 | 0.135 | 0 | 11 *TIMP3* | Late RPCs |
| 26E-228 | 0.649176 | 0.853 | 0.402 | 2.70E-224 | 11 *DHRS3* | Late RPCs |
| 0 | 0.640065 | 0.565 | 0.081 | 0 | 11 *BST2* | Late RPCs |
| 0 | 0.626285 | 0.667 | 0.163 | 0 | 11 *PKP4* | Late RPCs |
| 0 | 0.613752 | 0.671 | 0.14 | 0 | 11 *COLEC12* | Late RPCs |
| 33E-186 | 0.613696 | 0.878 | 0.491 | 2.85E-182 | 11 *ITM2C* | Late RPCs |
| 24E-275 | 0.612688 | 0.812 | 0.292 | 6.95E-271 | 11 *PCDH9* | Late RPCs |
| 90E-188 | 0.610252 | 0.804 | 0.394 | 1.69E-183 | 11 *SLC2A1* | Late RPCs |
| 41E-161 | 0.602943 | 0.804 | 0.365 | 3.02E-157 | 11 *IFITM3* | Late RPCs |
| 30E-150 | 0.59134 | 0.914 | 0.608 | 2.78E-146 | 11 *B2M* | Late RPCs |
| 77E-306 | 0.584827 | 0.773 | 0.247 | 5.93E-302 | 11 *SELENOM* | Late RPCs |
| 44E-132 | 0.575275 | 0.839 | 0.467 | 3.09E-128 | 11 *IGFBP5* | Late RPCs |
| 47E-133 | 0.561105 | 0.861 | 0.512 | 1.82E-128 | 11 *JUNB* | Late RPCs |
| 0 | 0.559461 | 0.548 | 0.032 | 0 | 11 *CHSY3* | Late RPCs |
| 30E-244 | 0.555755 | 0.788 | 0.311 | 7.08E-240 | 11 *TSPAN4* | Late RPCs |
| 0 | 0.542104 | 0.524 | 0.089 | 0 | 11 *NBL1* | Late RPCs |
| 0 | 0.541108 | 0.645 | 0.138 | 0 | 11 *CFI* | Late RPCs |
| 0 | 0.537061 | 0.567 | 0.092 | 0 | 11 *LRRC17* | Late RPCs |
| 0 | 0.533631 | 0.574 | 0.109 | 0 | 11 *TRPM3* | Late RPCs |
| 5.04E-74 | 0.533445 | 0.362 | 0.149 | 1.08E-69 | 11 *ID2* | Late RPCs |
| 0 | 0.533334 | 0.643 | 0.161 | 0 | 11 *PCDH7* | Late RPCs |
| 0 | 0.531723 | 0.54 | 0.023 | 0 | 11 *MECOM* | Late RPCs |
| 0 | 0.528881 | 0.431 | 0.059 | 0 | 11 *ATP1A2* | Late RPCs |
| 10E-227 | 0.520449 | 0.739 | 0.278 | 1.31E-222 | 11 *HSPB1* | Late RPCs |
| 30E-182 | 0.518808 | 0.797 | 0.379 | 9.21E-178 | 11 *SQSTM1* | Late RPCs |
| 0 | 0.515286 | 0.557 | 0.11 | 0 | 11 *PLK2* | Late RPCs |
| 0 | 0.505583 | 0.574 | 0.12 | 0 | 11 *VCAN* | Late RPCs |
| 0 | 0.504347 | 0.629 | 0.109 | 0 | 11 *KRT8* | Late RPCs |
| 65E-267 | 0.503198 | 1 | 1 | 7.84E-263 | 11 *RPL41* | Late RPCs |
| 42E-244 | 0.502402 | 0.543 | 0.146 | 1.59E-239 | 11 *NECTIN3* | Late RPCs |
| 0 | 0.501651 | 0.549 | 0.075 | 0 | 11 *TPD52L1* | Late RPCs |
| 33E-206 | 1.043864 | 0.913 | 0.586 | 4.99E-202 | 12 *HES6* | Late RPCs |
| 81E-282 | 0.935524 | 0.905 | 0.443 | 3.89E-278 | 12 *MIAT* | Late RPCs |
| 39E-208 | 0.898759 | 0.946 | 0.639 | 5.13E-204 | 12 *CCND1* | Late RPCs |
| 89E-254 | 0.854 | 1 | 0.951 | 1.91E-249 | 12 *CKB* | Late RPCs |
| 95E-257 | 0.821069 | 0.879 | 0.416 | 8.46E-253 | 12 *PRSS23* | Late RPCs |
| 17E-209 | 0.713498 | 0.94 | 0.616 | 1.54E-204 | 12 *PLEKHA1* | Late RPCs |
| 96E-130 | 0.664056 | 0.867 | 0.581 | 6.34E-126 | 12 *RGS16* | Late RPCs |
| 98E-197 | 0.641893 | 0.775 | 0.345 | 6.39E-193 | 12 *PTPRZ1* | Late RPCs |

| CLU2 4. |
| --- |
| DAPL11 5. |
| COL9A3 |
| HSD17B2 |
| SFRP23 1. |
| NR2F11 8. |
| ZIC1 |
| ARL4A |
| ID1 4. |
| FAM84A |
| CYSTM1 |
| FTH1 |
| ALDH1A11 |
| GCHFR |
| CXCL14 |
| PARD31 |
| CYP1B12 1. |
| FBN1 |
| PTN1 2. |
| IGFBP6 |
| NPC2 1. |
| CDON |
| MFAP2 3. |
| MFAP4 |
| TIMP3 |
| DHRS3 1. |
| BST2 |
| PKP4 |
| COLEC12 |
| ITM2C 1. |
| PCDH91 3. |
| SLC2A1 7. |
| IFITM32 1. |
| B2M 1. |
| SELENOM 2. |
| IGFBP52 1. |
| JUNB 8. |
| CHSY3 |
| TSPAN4 3. |
| NBL1 |
| CFI |
| LRRC171 |
| TRPM3 |
| ID2 |
| PCDH7 |
| MECOM |
| ATP1A2 |
| HSPB1 6. |
| SQSTM1 4. |
| PLK2 |
| VCAN |
| KRT8 |
| RPL41 3. |
| NECTIN3 7. |
| TPD52L1 |
| HES61 2. |
| MIAT1 1. |
| CCND1 2. |
| CKB 8. |
| PRSS232 3. |
| PLEKHA12 7. |
| RGS161 2. |
| PTPRZ11 2. |

| BTG2 1. |
| --- |
| SPP14 6. |
| ITGA6 4. |
| HIST1H4C |
| TOP2A |
| NUSAP1 |
| HMGB2 |
| TUBA1B1 |
| MKI67 |
| HIST1H1D |
| CENPF |
| UBE2C |
| SMC4 |
| TYMS1 |
| CDK1 |
| PCLAF1 |
| H2AFZ |
| HMGB1 |
| UBE2T |
| CKS1B |
| BIRC5 |
| CKS2 |
| TUBB |
| PTTG1 |
| H2AFX |
| TPX2 |
| MAD2L1 |
| ASPM |
| PBK |
| HIST1H1C |
| CCDC34 |
| CENPU |
| TMSB15A 4. |
| SPC25 |
| HMGN2 3. |
| RAD51AP1 |
| ESCO2 |
| FBXO5 |
| MIS18BP1 |
| KNL1 |
| CDKN3 |
| CENPH |
| ATAD2 |
| TMPO 3. |
| NUCKS1 3. |
| DLGAP5 |
| H2AFV 1. |
| STMN1 9. |
| AURKB |
| CKAP2 |
| SMC2 |
| DTYMK |
| HIST1H1B |
| TUBB4B2 2. |
| CALM3 4. |
| NDC80 |
| RRM2 |
| ANP32E 5. |
| CDCA3 |
| ZWINT |
| DEK 4. |
| GTSE1 |
| KIF11 |

| 18E-117 | 0.626542 | 0.762 | 0.466 | 2.53E-113 | 12 *BTG2* | Late RPCs |
| --- | --- | --- | --- | --- | --- | --- |
| 76E-133 | 0.540391 | 0.956 | 0.66 | 1.45E-128 | 12 *SPP1* | Late RPCs |
| 09E-166 | 0.531718 | 0.515 | 0.17 | 8.76E-162 | 12 *ITGA6* | Late RPCs |
| 0 | 1.936912 | 0.935 | 0.352 | 0 | 13 *HIST1H4C* | proliferating cells (MKi67+) |
| 0 | 1.633891 | 0.992 | 0.166 | 0 | 13 *TOP2A* | proliferating cells (MKi67+) |
| 0 | 1.335859 | 0.969 | 0.175 | 0 | 13 *NUSAP1* | proliferating cells (MKi67+) |
| 0 | 1.331609 | 0.998 | 0.508 | 0 | 13 *HMGB2* | proliferating cells (MKi67+) |
| 0 | 1.318795 | 1 | 0.887 | 0 | 13 *TUBA1B* | proliferating cells (MKi67+) |
| 0 | 1.300544 | 0.927 | 0.106 | 0 | 13 *MKI67* | proliferating cells (MKi67+) |
| 0 | 1.282247 | 0.839 | 0.132 | 0 | 13 *HIST1H1D* | proliferating cells (MKi67+) |
| 0 | 1.135576 | 0.949 | 0.171 | 0 | 13 *CENPF* | proliferating cells (MKi67+) |
| 0 | 1.129284 | 0.897 | 0.097 | 0 | 13 *UBE2C* | proliferating cells (MKi67+) |
| 0 | 1.105006 | 0.934 | 0.193 | 0 | 13 *SMC4* | proliferating cells (MKi67+) |
| 0 | 1.066774 | 0.97 | 0.321 | 0 | 13 *TYMS* | proliferating cells (MKi67+) |
| 0 | 1.025339 | 0.907 | 0.116 | 0 | 13 *CDK1* | proliferating cells (MKi67+) |
| 0 | 0.996797 | 0.894 | 0.198 | 0 | 13 *PCLAF* | proliferating cells (MKi67+) |
| 0 | 0.995816 | 1 | 0.922 | 0 | 13 *H2AFZ* | proliferating cells (MKi67+) |
| 0 | 0.990104 | 1 | 0.982 | 0 | 13 *HMGB1* | proliferating cells (MKi67+) |
| 0 | 0.961119 | 0.916 | 0.222 | 0 | 13 *UBE2T* | proliferating cells (MKi67+) |
| 0 | 0.954546 | 0.864 | 0.165 | 0 | 13 *CKS1B* | proliferating cells (MKi67+) |
| 0 | 0.936803 | 0.852 | 0.074 | 0 | 13 *BIRC5* | proliferating cells (MKi67+) |
| 0 | 0.908514 | 0.9 | 0.305 | 0 | 13 *CKS2* | proliferating cells (MKi67+) |
| 0 | 0.90851 | 1 | 0.909 | 0 | 13 *TUBB* | proliferating cells (MKi67+) |
| 0 | 0.904109 | 0.869 | 0.209 | 0 | 13 *PTTG1* | proliferating cells (MKi67+) |
| 0 | 0.899232 | 0.863 | 0.189 | 0 | 13 *H2AFX* | proliferating cells (MKi67+) |
| 0 | 0.850084 | 0.806 | 0.103 | 0 | 13 *TPX2* | proliferating cells (MKi67+) |
| 0 | 0.849335 | 0.865 | 0.154 | 0 | 13 *MAD2L1* | proliferating cells (MKi67+) |
| 0 | 0.843945 | 0.754 | 0.075 | 0 | 13 *ASPM* | proliferating cells (MKi67+) |
| 0 | 0.835914 | 0.803 | 0.063 | 0 | 13 *PBK* | proliferating cells (MKi67+) |
| 0 | 0.824961 | 0.766 | 0.178 | 0 | 13 *HIST1H1C* | proliferating cells (MKi67+) |
| 0 | 0.770272 | 0.897 | 0.344 | 0 | 13 *CCDC34* | proliferating cells (MKi67+) |
| 0 | 0.74941 | 0.81 | 0.17 | 0 | 13 *CENPU* | proliferating cells (MKi67+) |
| 14E-305 | 0.738555 | 0.939 | 0.41 | 8.88E-301 | 13 *TMSB15A* | proliferating cells (MKi67+) |
| 0 | 0.73832 | 0.74 | 0.054 | 0 | 13 *SPC25* | proliferating cells (MKi67+) |
| 33E-245 | 0.731438 | 0.991 | 0.901 | 7.14E-241 | 13 *HMGN2* | proliferating cells (MKi67+) |
| 0 | 0.728625 | 0.744 | 0.096 | 0 | 13 *RAD51AP1* | proliferating cells (MKi67+) |
| 0 | 0.723671 | 0.673 | 0.039 | 0 | 13 *ESCO2* | proliferating cells (MKi67+) |
| 0 | 0.723207 | 0.789 | 0.139 | 0 | 13 *FBXO5* | proliferating cells (MKi67+) |
| 0 | 0.700205 | 0.742 | 0.139 | 0 | 13 *MIS18BP1* | proliferating cells (MKi67+) |
| 0 | 0.669085 | 0.657 | 0.052 | 0 | 13 *KNL1* | proliferating cells (MKi67+) |
| 0 | 0.659039 | 0.702 | 0.098 | 0 | 13 *CDKN3* | proliferating cells (MKi67+) |
| 0 | 0.658204 | 0.785 | 0.185 | 0 | 13 *CENPH* | proliferating cells (MKi67+) |
| 0 | 0.656886 | 0.742 | 0.154 | 0 | 13 *ATAD2* | proliferating cells (MKi67+) |
| 75E-241 | 0.654124 | 0.885 | 0.427 | 8.03E-237 | 13 *TMPO* | proliferating cells (MKi67+) |
| 59E-251 | 0.651915 | 0.993 | 0.904 | 7.70E-247 | 13 *NUCKS1* | proliferating cells (MKi67+) |
| 0 | 0.650881 | 0.625 | 0.05 | 0 | 13 *DLGAP5* | proliferating cells (MKi67+) |
| 54E-246 | 0.64938 | 0.947 | 0.551 | 3.30E-242 | 13 *H2AFV* | proliferating cells (MKi67+) |
| 86E-252 | 0.648445 | 1 | 0.952 | 2.11E-247 | 13 *STMN1* | proliferating cells (MKi67+) |
| 0 | 0.641271 | 0.671 | 0.043 | 0 | 13 *AURKB* | proliferating cells (MKi67+) |
| 0 | 0.6288 | 0.805 | 0.226 | 0 | 13 *CKAP2* | proliferating cells (MKi67+) |
| 0 | 0.626179 | 0.747 | 0.174 | 0 | 13 *SMC2* | proliferating cells (MKi67+) |
| 0 | 0.62519 | 0.803 | 0.257 | 0 | 13 *DTYMK* | proliferating cells (MKi67+) |
| 0 | 0.618477 | 0.548 | 0.022 | 0 | 13 *HIST1H1B* | proliferating cells (MKi67+) |
| 07E-201 | 0.610525 | 0.986 | 0.744 | 4.43E-197 | 13 *TUBB4B* | proliferating cells (MKi67+) |
| 59E-193 | 0.601659 | 0.865 | 0.485 | 9.84E-189 | 13 *CALM3* | proliferating cells (MKi67+) |
| 0 | 0.596088 | 0.63 | 0.054 | 0 | 13 *NDC80* | proliferating cells (MKi67+) |
| 0 | 0.593807 | 0.601 | 0.067 | 0 | 13 *RRM2* | proliferating cells (MKi67+) |
| 42E-214 | 0.588902 | 0.884 | 0.455 | 1.16E-209 | 13 *ANP32E* | proliferating cells (MKi67+) |
| 0 | 0.588455 | 0.606 | 0.051 | 0 | 13 *CDCA3* | proliferating cells (MKi67+) |
| 0 | 0.58131 | 0.66 | 0.097 | 0 | 13 *ZWINT* | proliferating cells (MKi67+) |
| 62E-203 | 0.580498 | 0.983 | 0.772 | 9.91E-199 | 13 *DEK* | proliferating cells (MKi67+) |
| 0 | 0.575991 | 0.633 | 0.073 | 0 | 13 *GTSE1* | proliferating cells (MKi67+) |
| 0 | 0.574454 | 0.614 | 0.065 | 0 | 13 *KIF11* | proliferating cells (MKi67+) |

| MXD3 | 0 | 0.572831 | 0.584 | 0.054 | 0 | 13 | *MXD3* | proliferating cells (MKi67+) |
| --- | --- | --- | --- | --- | --- | --- | --- | --- |
| RRM1 | 2.18E-285 | 0.572542 | 0.766 | 0.245 | 4.68E-281 | 13 | *RRM1* | proliferating cells (MKi67+) |
| HELLS1 | 1.92E-219 | 0.566358 | 0.72 | 0.255 | 4.11E-215 | 13 | *HELLS* | proliferating cells (MKi67+) |
| KIFC1 | 0 | 0.564828 | 0.618 | 0.061 | 0 | 13 | *KIFC1* | proliferating cells (MKi67+) |
| ORC6 | 0 | 0.56176 | 0.674 | 0.127 | 0 | 13 | *ORC6* | proliferating cells (MKi67+) |
| CDCA5 | 0 | 0.560402 | 0.584 | 0.038 | 0 | 13 | *CDCA5* | proliferating cells (MKi67+) |
| USP1 | 2.55E-221 | 0.559001 | 0.845 | 0.367 | 5.47E-217 | 13 | *USP1* | proliferating cells (MKi67+) |
| ASF1B | 0 | 0.557959 | 0.639 | 0.088 | 0 | 13 | *ASF1B* | proliferating cells (MKi67+) |
| CCNA2 | 0 | 0.555531 | 0.622 | 0.054 | 0 | 13 | *CCNA2* | proliferating cells (MKi67+) |
| FAM111A | 0 | 0.555118 | 0.629 | 0.132 | 0 | 13 | *FAM111A* | proliferating cells (MKi67+) |
| DUT1 | 2.53E-139 | 0.554842 | 0.802 | 0.467 | 5.43E-135 | 13 | *DUT* | proliferating cells (MKi67+) |
| KIF20B | 0 | 0.553466 | 0.636 | 0.104 | 0 | 13 | *KIF20B* | proliferating cells (MKi67+) |
| PIMREG | 0 | 0.553044 | 0.59 | 0.047 | 0 | 13 | *PIMREG* | proliferating cells (MKi67+) |
| UBE2S | 3.62E-177 | 0.553003 | 0.839 | 0.444 | 7.77E-173 | 13 | *UBE2S* | proliferating cells (MKi67+) |
| H1FX | 3.19E-128 | 0.552718 | 0.921 | 0.719 | 6.83E-124 | 13 | *H1FX* | proliferating cells (MKi67+) |
| DHFR | 0 | 0.548796 | 0.652 | 0.151 | 0 | 13 | *DHFR* | proliferating cells (MKi67+) |
| CENPK | 0 | 0.546307 | 0.645 | 0.122 | 0 | 13 | *CENPK* | proliferating cells (MKi67+) |
| KIF23 | 0 | 0.545936 | 0.592 | 0.048 | 0 | 13 | *KIF23* | proliferating cells (MKi67+) |
| GMNN | 1.22E-306 | 0.540533 | 0.769 | 0.228 | 2.61E-302 | 13 | *GMNN* | proliferating cells (MKi67+) |
| PCNA1 | 3.92E-197 | 0.538396 | 0.78 | 0.323 | 8.40E-193 | 13 | *PCNA* | proliferating cells (MKi67+) |
| LMNB1 | 3.06E-306 | 0.529699 | 0.726 | 0.203 | 6.56E-302 | 13 | *LMNB1* | proliferating cells (MKi67+) |
| RANBP1 | 4.11E-172 | 0.528182 | 0.933 | 0.601 | 8.82E-168 | 13 | *RANBP1* | proliferating cells (MKi67+) |
| NUF2 | 0 | 0.527917 | 0.563 | 0.056 | 0 | 13 | *NUF2* | proliferating cells (MKi67+) |
| MND1 | 0 | 0.527103 | 0.582 | 0.041 | 0 | 13 | *MND1* | proliferating cells (MKi67+) |
| UBB | 1.01E-130 | 0.526273 | 0.98 | 0.924 | 2.16E-126 | 13 | *UBB* | proliferating cells (MKi67+) |
| RTKN2 | 0 | 0.524108 | 0.581 | 0.062 | 0 | 13 | *RTKN2* | proliferating cells (MKi67+) |
| NCAPG | 0 | 0.522851 | 0.589 | 0.055 | 0 | 13 | *NCAPG* | proliferating cells (MKi67+) |
| TMEM106 | 1.95E-211 | 0.520674 | 0.79 | 0.32 | 4.18E-207 | 13 | *TMEM106* | proliferating cells (MKi67+) |
| ITGB3BP | 1.57E-300 | 0.520603 | 0.679 | 0.185 | 3.36E-296 | 13 | *ITGB3BP* | proliferating cells (MKi67+) |
| TAGLN2 | 1.36E-131 | 0.517926 | 0.827 | 0.5 | 2.91E-127 | 13 | *TAGLN2* | proliferating cells (MKi67+) |
| C21orf58 | 0 | 0.517463 | 0.618 | 0.115 | 0 | 13 | *C21orf58* | proliferating cells (MKi67+) |
| SGO2 | 0 | 0.517195 | 0.574 | 0.066 | 0 | 13 | *SGO2* | proliferating cells (MKi67+) |
| SIVA1 | 3.94E-181 | 0.515818 | 0.849 | 0.449 | 8.45E-177 | 13 | *SIVA1* | proliferating cells (MKi67+) |
| BUB3 | 6.98E-205 | 0.512476 | 0.774 | 0.324 | 1.50E-200 | 13 | *BUB3* | proliferating cells (MKi67+) |
| CKB1 | 3.09E-105 | 0.51173 | 0.988 | 0.952 | 6.63E-101 | 13 | *CKB* | proliferating cells (MKi67+) |
| FAM111B1 | 3.08E-232 | 0.507758 | 0.616 | 0.174 | 6.61E-228 | 13 | *FAM111B* | proliferating cells (MKi67+) |
| PPIA | 1.43E-183 | 0.503827 | 0.994 | 0.901 | 3.07E-179 | 13 | *PPIA* | proliferating cells (MKi67+) |
| TACC3 | 0 | 0.502932 | 0.559 | 0.063 | 0 | 13 | *TACC3* | proliferating cells (MKi67+) |
| VSX12 | 6.68E-226 | 1.018916 | 0.471 | 0.111 | 1.43E-221 | 14 | *VSX1* | NRPCs/T3 |
| FABP72 | 2.47E-66 | 0.84319 | 0.709 | 0.54 | 5.29E-62 | 14 | *FABP7* | NRPCs/T3 |
| NEUROD41 | 3.34E-285 | 0.826043 | 0.713 | 0.213 | 7.16E-281 | 14 | *NEUROD4* | NRPCs/T3 |
| CRYBG32 | 1.62E-211 | 0.822287 | 0.669 | 0.248 | 3.47E-207 | 14 | *CRYBG3* | NRPCs/T3 |
| OTX22 | 1.21E-297 | 0.817678 | 0.793 | 0.259 | 2.58E-293 | 14 | *OTX2* | NRPCs/T3 |
| AMER21 | 1.97E-217 | 0.789251 | 0.79 | 0.337 | 4.23E-213 | 14 | *AMER2* | NRPCs/T3 |
| PIK3R12 | 3.60E-122 | 0.707295 | 0.78 | 0.495 | 7.73E-118 | 14 | *PIK3R1* | NRPCs/T3 |
| CHODL1 | 2.18E-152 | 0.691317 | 0.417 | 0.117 | 4.68E-148 | 14 | *CHODL* | NRPCs/T3 |
| SLC38A53 | 2.47E-132 | 0.667496 | 0.614 | 0.254 | 5.29E-128 | 14 | *SLC38A5* | NRPCs/T3 |
| PCBP43 | 1.33E-196 | 0.655603 | 0.846 | 0.382 | 2.84E-192 | 14 | *PCBP4* | NRPCs/T3 |
| SPCS11 | 1.43E-156 | 0.637095 | 0.931 | 0.775 | 3.06E-152 | 14 | *SPCS1* | NRPCs/T3 |
| NEUROD12 | 2.48E-145 | 0.609123 | 0.696 | 0.295 | 5.31E-141 | 14 | *NEUROD1* | NRPCs/T3 |
| SOX42 | 5.04E-163 | 0.585077 | 0.992 | 0.858 | 1.08E-158 | 14 | *SOX4* | NRPCs/T3 |
| SCG33 | 2.25E-140 | 0.579282 | 0.821 | 0.4 | 4.83E-136 | 14 | *SCG3* | NRPCs/T3 |
| NRL2 | 8.81E-137 | 0.529535 | 0.785 | 0.383 | 1.89E-132 | 14 | *NRL* | NRPCs/T3 |
| GADD45G2 | 3.61E-91 | 0.528079 | 0.657 | 0.348 | 7.74E-87 | 14 | *GADD45G* | NRPCs/T3 |
| SYP3 | 3.23E-140 | 0.509286 | 0.676 | 0.28 | 6.93E-136 | 14 | *SYP* | NRPCs/T3 |
| LINC00599 | 6.03E-124 | 0.502755 | 0.582 | 0.232 | 1.29E-119 | 14 | *LINC00599* | NRPCs/T3 |
| PMAIP11 | 0 | 1.380843 | 0.919 | 0.259 | 0 | 15 | *PMAIP1* | Proliferating NRPCs (MKi67-) |
| GADD45A1 | 0 | 1.225315 | 0.917 | 0.412 | 0 | 15 | *GADD45A* | Proliferating NRPCs (MKi67-) |
| HES62 | 7.29E-222 | 1.102377 | 0.965 | 0.585 | 1.56E-217 | 15 | *HES6* | Proliferating NRPCs (MKi67-) |
| TYMS2 | 0 | 1.002242 | 0.936 | 0.325 | 0 | 15 | *TYMS* | Proliferating NRPCs (MKi67-) |
| PCLAF2 | 0 | 0.924067 | 0.845 | 0.202 | 0 | 15 | *PCLAF* | Proliferating NRPCs (MKi67-) |
| PTMA | 0 | 0.855963 | 1 | 1 | 0 | 15 | *PTMA* | Proliferating NRPCs (MKi67-) |
| PCNA2 | 0 | 0.836973 | 0.9 | 0.32 | 0 | 15 | *PCNA* | Proliferating NRPCs (MKi67-) |

| FAM111B2 | 0 | 0.814243 | 0.807 | 0.168 | 0 | 15 | *FAM111B* | Proliferating NRPCs (MKi67-) |
| --- | --- | --- | --- | --- | --- | --- | --- | --- |
| L1TD1 | 0 | 0.800411 | 0.388 | 0.045 | 0 | 15 | *L1TD1* | Proliferating NRPCs (MKi67-) |
| NEAT1 | 2.53E-97 | 0.797479 | 0.962 | 0.919 | 5.43E-93 | 15 | *NEAT1* | Proliferating NRPCs (MKi67-) |
| TUBA1B2 | 5.54E-251 | 0.795515 | 1 | 0.887 | 1.19E-246 | 15 | *TUBA1B* | Proliferating NRPCs (MKi67-) |
| SLBP1 | 0 | 0.760998 | 0.842 | 0.282 | 0 | 15 | *SLBP* | Proliferating NRPCs (MKi67-) |
| BTG3 | 1.20E-253 | 0.759634 | 0.894 | 0.449 | 2.58E-249 | 15 | *BTG3* | Proliferating NRPCs (MKi67-) |
| NASP1 | 2.47E-255 | 0.741013 | 0.965 | 0.572 | 5.29E-251 | 15 | *NASP* | Proliferating NRPCs (MKi67-) |
| HMGN21 | 8.92E-197 | 0.741008 | 0.995 | 0.901 | 1.91E-192 | 15 | *HMGN2* | Proliferating NRPCs (MKi67-) |
| HELLS2 | 0 | 0.735244 | 0.83 | 0.253 | 0 | 15 | *HELLS* | Proliferating NRPCs (MKi67-) |
| USP11 | 1.07E-270 | 0.732555 | 0.875 | 0.367 | 2.29E-266 | 15 | *USP1* | Proliferating NRPCs (MKi67-) |
| CARHSP1 | 2.99E-296 | 0.72527 | 0.837 | 0.312 | 6.41E-292 | 15 | *CARHSP1* | Proliferating NRPCs (MKi67-) |
| CENPU1 | 0 | 0.718411 | 0.765 | 0.173 | 0 | 15 | *CENPU* | Proliferating NRPCs (MKi67-) |
| SHD | 0 | 0.716651 | 0.705 | 0.155 | 0 | 15 | *SHD* | Proliferating NRPCs (MKi67-) |
| TMSB15A1 | 2.73E-241 | 0.713861 | 0.898 | 0.413 | 5.85E-237 | 15 | *TMSB15A* | Proliferating NRPCs (MKi67-) |
| CHGB | 2.18E-49 | 0.679068 | 0.325 | 0.148 | 4.67E-45 | 15 | *CHGB* | Proliferating NRPCs (MKi67-) |
| TUBB1 | 1.02E-219 | 0.674658 | 0.999 | 0.909 | 2.19E-215 | 15 | *TUBB* | Proliferating NRPCs (MKi67-) |
| HMGB21 | 6.73E-246 | 0.673992 | 0.95 | 0.511 | 1.44E-241 | 15 | *HMGB2* | Proliferating NRPCs (MKi67-) |
| MIAT2 | 3.02E-127 | 0.659114 | 0.806 | 0.448 | 6.47E-123 | 15 | *MIAT* | Proliferating NRPCs (MKi67-) |
| H2AFZ1 | 4.08E-228 | 0.658075 | 1 | 0.922 | 8.74E-224 | 15 | *H2AFZ* | Proliferating NRPCs (MKi67-) |
| PPIA1 | 1.11E-208 | 0.655852 | 0.99 | 0.901 | 2.39E-204 | 15 | *PPIA* | Proliferating NRPCs (MKi67-) |
| CXCR4 | 4.87E-248 | 0.652596 | 0.712 | 0.23 | 1.04E-243 | 15 | *CXCR4* | Proliferating NRPCs (MKi67-) |
| DUT2 | 4.24E-203 | 0.6459 | 0.876 | 0.466 | 9.10E-199 | 15 | *DUT* | Proliferating NRPCs (MKi67-) |
| CKB2 | 8.91E-141 | 0.641345 | 0.991 | 0.952 | 1.91E-136 | 15 | *CKB* | Proliferating NRPCs (MKi67-) |
| RANBP11 | 4.92E-198 | 0.635821 | 0.936 | 0.602 | 1.06E-193 | 15 | *RANBP1* | Proliferating NRPCs (MKi67-) |
| SYNE2 | 2.71E-151 | 0.634651 | 0.881 | 0.552 | 5.80E-147 | 15 | *SYNE2* | Proliferating NRPCs (MKi67-) |
| RRM11 | 6.65E-283 | 0.630066 | 0.77 | 0.246 | 1.43E-278 | 15 | *RRM1* | Proliferating NRPCs (MKi67-) |
| PRKDC | 1.34E-161 | 0.624239 | 0.87 | 0.525 | 2.88E-157 | 15 | *PRKDC* | Proliferating NRPCs (MKi67-) |
| RGS162 | 9.89E-130 | 0.621185 | 0.904 | 0.581 | 2.12E-125 | 15 | *RGS16* | Proliferating NRPCs (MKi67-) |
| HNRNPAB | 2.49E-193 | 0.618713 | 0.909 | 0.534 | 5.33E-189 | 15 | *HNRNPAB* | Proliferating NRPCs (MKi67-) |
| DEK1 | 1.32E-199 | 0.604095 | 0.987 | 0.772 | 2.84E-195 | 15 | *DEK* | Proliferating NRPCs (MKi67-) |
| NNAT | 1.86E-134 | 0.603894 | 0.778 | 0.385 | 3.99E-130 | 15 | *NNAT* | Proliferating NRPCs (MKi67-) |
| PLEKHA13 | 1.29E-131 | 0.599672 | 0.924 | 0.617 | 2.76E-127 | 15 | *PLEKHA1* | Proliferating NRPCs (MKi67-) |
| H2AFY | 3.00E-163 | 0.599628 | 0.908 | 0.619 | 6.43E-159 | 15 | *H2AFY* | Proliferating NRPCs (MKi67-) |
| MCM7 | 1.07E-296 | 0.592185 | 0.782 | 0.236 | 2.29E-292 | 15 | *MCM7* | Proliferating NRPCs (MKi67-) |
| HSPB11 | 1.33E-218 | 0.588485 | 0.743 | 0.283 | 2.85E-214 | 15 | *HSPB11* | Proliferating NRPCs (MKi67-) |
| CCNE2 | 0 | 0.586038 | 0.641 | 0.096 | 0 | 15 | *CCNE2* | Proliferating NRPCs (MKi67-) |
| MCM4 | 0 | 0.577743 | 0.721 | 0.186 | 0 | 15 | *MCM4* | Proliferating NRPCs (MKi67-) |
| CLSPN | 0 | 0.573363 | 0.672 | 0.147 | 0 | 15 | *CLSPN* | Proliferating NRPCs (MKi67-) |
| SNRPB | 5.07E-173 | 0.571095 | 0.916 | 0.561 | 1.09E-168 | 15 | *SNRPB* | Proliferating NRPCs (MKi67-) |
| CENPV | 9.82E-173 | 0.565454 | 0.879 | 0.477 | 2.11E-168 | 15 | *CENPV* | Proliferating NRPCs (MKi67-) |
| SUPT16H | 6.46E-162 | 0.565363 | 0.837 | 0.461 | 1.38E-157 | 15 | *SUPT16H* | Proliferating NRPCs (MKi67-) |
| RBBP7 | 3.56E-160 | 0.559216 | 0.811 | 0.421 | 7.63E-156 | 15 | *RBBP7* | Proliferating NRPCs (MKi67-) |
| TMPO1 | 1.94E-173 | 0.539164 | 0.85 | 0.43 | 4.16E-169 | 15 | *TMPO* | Proliferating NRPCs (MKi67-) |
| DBI | 7.12E-142 | 0.537889 | 0.864 | 0.511 | 1.53E-137 | 15 | *DBI* | Proliferating NRPCs (MKi67-) |
| FEN1 | 4.76E-243 | 0.526637 | 0.714 | 0.226 | 1.02E-238 | 15 | *FEN1* | Proliferating NRPCs (MKi67-) |
| FADS1 | 5.12E-144 | 0.524588 | 0.759 | 0.383 | 1.10E-139 | 15 | *FADS1* | Proliferating NRPCs (MKi67-) |
| TMEM106 | 6.69E-143 | 0.52171 | 0.699 | 0.324 | 1.44E-138 | 15 | *TMEM106* | Proliferating NRPCs (MKi67-) |
| ASF1B1 | 0 | 0.520862 | 0.537 | 0.093 | 0 | 15 | *ASF1B* | Proliferating NRPCs (MKi67-) |
| GMNN1 | 4.60E-227 | 0.520842 | 0.707 | 0.232 | 9.85E-223 | 15 | *GMNN* | Proliferating NRPCs (MKi67-) |
| H3F3A | 1.19E-187 | 0.518442 | 1 | 0.999 | 2.55E-183 | 15 | *H3F3A* | Proliferating NRPCs (MKi67-) |
| SNRPD1 | 1.79E-154 | 0.515074 | 0.9 | 0.558 | 3.84E-150 | 15 | *SNRPD1* | Proliferating NRPCs (MKi67-) |
| MSH6 | 9.41E-170 | 0.513625 | 0.744 | 0.322 | 2.02E-165 | 15 | *MSH6* | Proliferating NRPCs (MKi67-) |
| UBE2T1 | 5.21E-228 | 0.51071 | 0.724 | 0.23 | 1.12E-223 | 15 | *UBE2T* | Proliferating NRPCs (MKi67-) |
| KPNB1 | 1.29E-126 | 0.505279 | 0.827 | 0.502 | 2.77E-122 | 15 | *KPNB1* | Proliferating NRPCs (MKi67-) |
| CCDC341 | 4.86E-136 | 0.502658 | 0.75 | 0.35 | 1.04E-131 | 15 | *CCDC34* | Proliferating NRPCs (MKi67-) |
| VIM3 | 2.97E-191 | 0.859463 | 1 | 0.852 | 6.37E-187 | 16 | *VIM* | Muller Glia cells |
| TF3 | 5.61E-99 | 0.805892 | 0.696 | 0.38 | 1.20E-94 | 16 | *TF* | Muller Glia cells |
| SPP15 | 2.34E-167 | 0.727909 | 0.975 | 0.661 | 5.02E-163 | 16 | *SPP1* | Muller Glia cells |
| QDPR1 | 2.93E-116 | 0.657162 | 0.636 | 0.3 | 6.29E-112 | 16 | *QDPR* | Muller Glia cells |
| S100A6 | 1.29E-60 | 0.649583 | 0.72 | 0.518 | 2.77E-56 | 16 | *S100A6* | Muller Glia cells |
| IFITM24 | 3.92E-103 | 0.561132 | 0.769 | 0.416 | 8.40E-99 | 16 | *IFITM2* | Muller Glia cells |
| RTN42 | 2.75E-153 | 0.552822 | 0.999 | 0.962 | 5.91E-149 | 16 | *RTN4* | Muller Glia cells |
| NES1 | 7.69E-116 | 0.549371 | 0.615 | 0.27 | 1.65E-111 | 16 | *NES* | Muller Glia cells |

| ZFP36L22 | 3.74E-84 | 0.54851 | 0.745 | 0.439 | 8.02E-80 | 16 | *ZFP36L2* | Muller Glia cells |
| --- | --- | --- | --- | --- | --- | --- | --- | --- |
| DKK32 | 4.33E-115 | 0.532347 | 0.881 | 0.527 | 9.28E-111 | 16 | *DKK3* | Muller Glia cells |
| TNFRSF12A | 4.26E-100 | 0.527654 | 0.402 | 0.139 | 9.13E-96 | 16 | *TNFRSF12A* | Muller Glia cells |
| TTYH13 | 2.40E-120 | 0.521777 | 0.817 | 0.444 | 5.15E-116 | 16 | *TTYH1* | Muller Glia cells |
| ZFP36L11 | 1.92E-104 | 0.506061 | 0.874 | 0.53 | 4.12E-100 | 16 | *ZFP36L1* | Muller Glia cells |
| CENPF1 | 0 | 2.720841 | 1 | 0.181 | 0 | 18 | *CENPF* | proliferating cells (MKi67+) |
| TOP2A1 | 0 | 2.502428 | 1 | 0.177 | 0 | 18 | *TOP2A* | proliferating cells (MKi67+) |
| UBE2C1 | 0 | 2.258244 | 0.983 | 0.107 | 0 | 18 | *UBE2C* | proliferating cells (MKi67+) |
| MKI671 | 0 | 2.120076 | 0.996 | 0.117 | 0 | 18 | *MKI67* | proliferating cells (MKi67+) |
| PTTG11 | 0 | 2.119769 | 1 | 0.216 | 0 | 18 | *PTTG1* | proliferating cells (MKi67+) |
| HMGB22 | 0 | 2.104139 | 1 | 0.515 | 0 | 18 | *HMGB2* | proliferating cells (MKi67+) |
| CCNB1 | 0 | 2.019135 | 0.994 | 0.069 | 0 | 18 | *CCNB1* | proliferating cells (MKi67+) |
| ASPM1 | 0 | 2.018771 | 0.994 | 0.079 | 0 | 18 | *ASPM* | proliferating cells (MKi67+) |
| CKS21 | 0 | 1.951698 | 0.998 | 0.312 | 0 | 18 | *CKS2* | proliferating cells (MKi67+) |
| NUSAP11 | 0 | 1.873297 | 0.998 | 0.186 | 0 | 18 | *NUSAP1* | proliferating cells (MKi67+) |
| KPNA2 | 0 | 1.838349 | 0.959 | 0.188 | 0 | 18 | *KPNA2* | proliferating cells (MKi67+) |
| TPX21 | 0 | 1.825094 | 0.996 | 0.11 | 0 | 18 | *TPX2* | proliferating cells (MKi67+) |
| CCNB2 | 0 | 1.724995 | 0.988 | 0.073 | 0 | 18 | *CCNB2* | proliferating cells (MKi67+) |
| UBE2S1 | 0 | 1.676743 | 0.998 | 0.446 | 0 | 18 | *UBE2S* | proliferating cells (MKi67+) |
| ARL6IP1 | 0 | 1.632641 | 0.996 | 0.434 | 0 | 18 | *ARL6IP1* | proliferating cells (MKi67+) |
| CKAP21 | 0 | 1.540618 | 0.979 | 0.23 | 0 | 18 | *CKAP2* | proliferating cells (MKi67+) |
| CDK11 | 0 | 1.516991 | 0.903 | 0.128 | 0 | 18 | *CDK1* | proliferating cells (MKi67+) |
| TUBB4B3 | 5.16E-296 | 1.516968 | 0.996 | 0.748 | 1.11E-291 | 18 | *TUBB4B* | proliferating cells (MKi67+) |
| BIRC51 | 0 | 1.508765 | 0.979 | 0.083 | 0 | 18 | *BIRC5* | proliferating cells (MKi67+) |
| CDC20 | 0 | 1.498016 | 0.975 | 0.04 | 0 | 18 | *CDC20* | proliferating cells (MKi67+) |
| NEK2 | 0 | 1.482527 | 0.952 | 0.044 | 0 | 18 | *NEK2* | proliferating cells (MKi67+) |
| SMC41 | 0 | 1.463287 | 0.986 | 0.202 | 0 | 18 | *SMC4* | proliferating cells (MKi67+) |
| CDKN31 | 0 | 1.454899 | 0.979 | 0.101 | 0 | 18 | *CDKN3* | proliferating cells (MKi67+) |
| DLGAP51 | 0 | 1.447976 | 0.983 | 0.05 | 0 | 18 | *DLGAP5* | proliferating cells (MKi67+) |
| CKS1B1 | 0 | 1.362958 | 0.971 | 0.173 | 0 | 18 | *CKS1B* | proliferating cells (MKi67+) |
| CENPE | 0 | 1.35 | 0.928 | 0.05 | 0 | 18 | *CENPE* | proliferating cells (MKi67+) |
| TUBA1C | 0 | 1.302154 | 0.865 | 0.136 | 0 | 18 | *TUBA1C* | proliferating cells (MKi67+) |
| GTSE11 | 0 | 1.271398 | 0.932 | 0.075 | 0 | 18 | *GTSE1* | proliferating cells (MKi67+) |
| SGO21 | 0 | 1.257438 | 0.932 | 0.066 | 0 | 18 | *SGO2* | proliferating cells (MKi67+) |
| CENPA | 0 | 1.240825 | 0.946 | 0.029 | 0 | 18 | *CENPA* | proliferating cells (MKi67+) |
| CCNA21 | 0 | 1.238953 | 0.919 | 0.055 | 0 | 18 | *CCNA2* | proliferating cells (MKi67+) |
| PBK1 | 0 | 1.234999 | 0.938 | 0.071 | 0 | 18 | *PBK* | proliferating cells (MKi67+) |
| RAD21 | 0 | 1.231024 | 0.998 | 0.521 | 0 | 18 | *RAD21* | proliferating cells (MKi67+) |
| KIF20B1 | 0 | 1.227256 | 0.948 | 0.105 | 0 | 18 | *KIF20B* | proliferating cells (MKi67+) |
| TUBA1B3 | 4.96E-261 | 1.222385 | 1 | 0.889 | 1.06E-256 | 18 | *TUBA1B* | proliferating cells (MKi67+) |
| AURKA | 0 | 1.209247 | 0.909 | 0.039 | 0 | 18 | *AURKA* | proliferating cells (MKi67+) |
| MAD2L11 | 0 | 1.190687 | 0.965 | 0.162 | 0 | 18 | *MAD2L1* | proliferating cells (MKi67+) |
| NUF21 | 0 | 1.185443 | 0.93 | 0.055 | 0 | 18 | *NUF2* | proliferating cells (MKi67+) |
| UBE2T2 | 0 | 1.182782 | 0.973 | 0.231 | 0 | 18 | *UBE2T* | proliferating cells (MKi67+) |
| CCNA1 | 0 | 1.170055 | 0.888 | 0.046 | 0 | 18 | *CCNA1* | proliferating cells (MKi67+) |
| MIS18BP11 | 0 | 1.168462 | 0.94 | 0.144 | 0 | 18 | *MIS18BP1* | proliferating cells (MKi67+) |
| ECT2 | 0 | 1.158172 | 0.926 | 0.121 | 0 | 18 | *ECT2* | proliferating cells (MKi67+) |
| HMMR | 0 | 1.154132 | 0.876 | 0.037 | 0 | 18 | *HMMR* | proliferating cells (MKi67+) |
| KNL11 | 0 | 1.151442 | 0.89 | 0.056 | 0 | 18 | *KNL1* | proliferating cells (MKi67+) |
| JPT11 | 1.66E-293 | 1.138259 | 0.994 | 0.496 | 3.56E-289 | 18 | *JPT1* | proliferating cells (MKi67+) |
| CDCA8 | 0 | 1.101621 | 0.89 | 0.042 | 0 | 18 | *CDCA8* | proliferating cells (MKi67+) |
| NUCKS11 | 1.23E-289 | 1.096815 | 1 | 0.906 | 2.64E-285 | 18 | *NUCKS1* | proliferating cells (MKi67+) |
| KIF231 | 0 | 1.059987 | 0.843 | 0.05 | 0 | 18 | *KIF23* | proliferating cells (MKi67+) |
| CALM2 | 2.79E-267 | 1.051529 | 1 | 0.964 | 5.98E-263 | 18 | *CALM2* | proliferating cells (MKi67+) |
| HMGB3 | 0 | 1.047768 | 0.942 | 0.274 | 0 | 18 | *HMGB3* | proliferating cells (MKi67+) |
| H2AFZ2 | 1.49E-255 | 1.03135 | 1 | 0.923 | 3.20E-251 | 18 | *H2AFZ* | proliferating cells (MKi67+) |
| HIST1H4C1 | 9.29E-59 | 1.011962 | 0.634 | 0.367 | 1.99E-54 | 18 | *HIST1H4C* | proliferating cells (MKi67+) |
| PLK1 | 0 | 1.011131 | 0.851 | 0.021 | 0 | 18 | *PLK1* | proliferating cells (MKi67+) |
| NDC801 | 0 | 1.008754 | 0.888 | 0.056 | 0 | 18 | *NDC80* | proliferating cells (MKi67+) |
| HMGN22 | 6.25E-230 | 0.996954 | 1 | 0.902 | 1.34E-225 | 18 | *HMGN2* | proliferating cells (MKi67+) |
| GAS2L3 | 0 | 0.991321 | 0.868 | 0.074 | 0 | 18 | *GAS2L3* | proliferating cells (MKi67+) |
| PIMREG1 | 0 | 0.986244 | 0.876 | 0.048 | 0 | 18 | *PIMREG* | proliferating cells (MKi67+) |
| CDCA31 | 0 | 0.981189 | 0.841 | 0.053 | 0 | 18 | *CDCA3* | proliferating cells (MKi67+) |

| KNSTRN | 0 | 0.979714 | 0.839 | 0.063 | 0 | 18 | *KNSTRN* | proliferating cells (MKi67+) |
| --- | --- | --- | --- | --- | --- | --- | --- | --- |
| HSP90B1 | 8.36E-237 | 0.973656 | 0.99 | 0.686 | 1.79E-232 | 18 | *HSP90B1* | proliferating cells (MKi67+) |
| HP1BP3 | 2.11E-254 | 0.971835 | 0.971 | 0.517 | 4.52E-250 | 18 | *HP1BP3* | proliferating cells (MKi67+) |
| AURKB1 | 0 | 0.952499 | 0.826 | 0.049 | 0 | 18 | *AURKB* | proliferating cells (MKi67+) |
| H2AFX1 | 0 | 0.950886 | 0.849 | 0.199 | 0 | 18 | *H2AFX* | proliferating cells (MKi67+) |
| KIF111 | 0 | 0.938374 | 0.843 | 0.068 | 0 | 18 | *KIF11* | proliferating cells (MKi67+) |
| LSM5 | 1.08E-274 | 0.935296 | 0.95 | 0.391 | 2.32E-270 | 18 | *LSM5* | proliferating cells (MKi67+) |
| BUB31 | 3.64E-304 | 0.933422 | 0.934 | 0.327 | 7.80E-300 | 18 | *BUB3* | proliferating cells (MKi67+) |
| TAGLN21 | 6.13E-207 | 0.928095 | 0.959 | 0.502 | 1.32E-202 | 18 | *TAGLN2* | proliferating cells (MKi67+) |
| LMNB11 | 0 | 0.926247 | 0.88 | 0.207 | 0 | 18 | *LMNB1* | proliferating cells (MKi67+) |
| SGO1 | 0 | 0.917929 | 0.839 | 0.053 | 0 | 18 | *SGO1* | proliferating cells (MKi67+) |
| CKAP5 | 0 | 0.912523 | 0.892 | 0.211 | 0 | 18 | *CKAP5* | proliferating cells (MKi67+) |
| DBF4 | 0 | 0.912106 | 0.824 | 0.137 | 0 | 18 | *DBF4* | proliferating cells (MKi67+) |
| BUB1 | 0 | 0.906313 | 0.793 | 0.034 | 0 | 18 | *BUB1* | proliferating cells (MKi67+) |
| KIF2C | 0 | 0.903319 | 0.816 | 0.033 | 0 | 18 | *KIF2C* | proliferating cells (MKi67+) |
| TROAP | 0 | 0.895202 | 0.838 | 0.032 | 0 | 18 | *TROAP* | proliferating cells (MKi67+) |
| CALM31 | 1.90E-243 | 0.889009 | 0.975 | 0.488 | 4.07E-239 | 18 | *CALM3* | proliferating cells (MKi67+) |
| TMPO2 | 1.85E-222 | 0.883132 | 0.938 | 0.433 | 3.97E-218 | 18 | *TMPO* | proliferating cells (MKi67+) |
| DEPDC1 | 0 | 0.879941 | 0.783 | 0.027 | 0 | 18 | *DEPDC1* | proliferating cells (MKi67+) |
| KIF14 | 0 | 0.877498 | 0.799 | 0.029 | 0 | 18 | *KIF14* | proliferating cells (MKi67+) |
| MXD31 | 0 | 0.875105 | 0.783 | 0.057 | 0 | 18 | *MXD3* | proliferating cells (MKi67+) |
| HMGB11 | 4.48E-238 | 0.874388 | 1 | 0.982 | 9.60E-234 | 18 | *HMGB1* | proliferating cells (MKi67+) |
| NMU | 0 | 0.873931 | 0.795 | 0.067 | 0 | 18 | *NMU* | proliferating cells (MKi67+) |
| CKAP2L | 0 | 0.863996 | 0.785 | 0.045 | 0 | 18 | *CKAP2L* | proliferating cells (MKi67+) |
| SFPQ | 1.17E-197 | 0.858117 | 0.985 | 0.689 | 2.50E-193 | 18 | *SFPQ* | proliferating cells (MKi67+) |
| MARCKS1 | 4.37E-201 | 0.855249 | 0.998 | 0.896 | 9.38E-197 | 18 | *MARCKS* | proliferating cells (MKi67+) |
| CKB3 | 3.93E-131 | 0.850603 | 0.983 | 0.952 | 8.43E-127 | 18 | *CKB* | proliferating cells (MKi67+) |
| MZT1 | 0 | 0.840208 | 0.876 | 0.252 | 0 | 18 | *MZT1* | proliferating cells (MKi67+) |
| CCDC342 | 2.29E-226 | 0.836414 | 0.919 | 0.351 | 4.91E-222 | 18 | *CCDC34* | proliferating cells (MKi67+) |
| ANP32E1 | 1.51E-214 | 0.819386 | 0.944 | 0.46 | 3.24E-210 | 18 | *ANP32E* | proliferating cells (MKi67+) |
| PRR11 | 0 | 0.812344 | 0.747 | 0.041 | 0 | 18 | *PRR11* | proliferating cells (MKi67+) |
| CEP70 | 0 | 0.809218 | 0.855 | 0.2 | 0 | 18 | *CEP70* | proliferating cells (MKi67+) |
| DYNLL1 | 3.62E-183 | 0.796012 | 0.983 | 0.869 | 7.76E-179 | 18 | *DYNLL1* | proliferating cells (MKi67+) |
| KIF20A | 0 | 0.786465 | 0.743 | 0.017 | 0 | 18 | *KIF20A* | proliferating cells (MKi67+) |
| CEP55 | 0 | 0.783382 | 0.739 | 0.034 | 0 | 18 | *CEP55* | proliferating cells (MKi67+) |
| UBB1 | 1.06E-190 | 0.7792 | 0.994 | 0.925 | 2.28E-186 | 18 | *UBB* | proliferating cells (MKi67+) |
| KIFC11 | 0 | 0.773856 | 0.789 | 0.066 | 0 | 18 | *KIFC1* | proliferating cells (MKi67+) |
| TTK | 0 | 0.770733 | 0.735 | 0.037 | 0 | 18 | *TTK* | proliferating cells (MKi67+) |
| HNRNPA2B | 1.28E-212 | 0.766645 | 1 | 0.945 | 2.75E-208 | 18 | *HNRNPA2B* | proliferating cells (MKi67+) |
| HJURP | 0 | 0.761745 | 0.752 | 0.04 | 0 | 18 | *HJURP* | proliferating cells (MKi67+) |
| DTYMK1 | 5.88E-278 | 0.761539 | 0.878 | 0.263 | 1.26E-273 | 18 | *DTYMK* | proliferating cells (MKi67+) |
| PRC1 | 0 | 0.761108 | 0.756 | 0.063 | 0 | 18 | *PRC1* | proliferating cells (MKi67+) |
| CDCA2 | 0 | 0.758556 | 0.702 | 0.034 | 0 | 18 | *CDCA2* | proliferating cells (MKi67+) |
| RTKN21 | 0 | 0.747936 | 0.756 | 0.065 | 0 | 18 | *RTKN2* | proliferating cells (MKi67+) |
| KIF4A | 0 | 0.747538 | 0.729 | 0.058 | 0 | 18 | *KIF4A* | proliferating cells (MKi67+) |
| H2AFV1 | 7.49E-188 | 0.744731 | 0.971 | 0.556 | 1.61E-183 | 18 | *H2AFV* | proliferating cells (MKi67+) |
| VIM4 | 2.77E-128 | 0.743094 | 1 | 0.854 | 5.95E-124 | 18 | *VIM* | proliferating cells (MKi67+) |
| CDC25B | 0 | 0.742816 | 0.743 | 0.074 | 0 | 18 | *CDC25B* | proliferating cells (MKi67+) |
| RACGAP1 | 0 | 0.727325 | 0.75 | 0.08 | 0 | 18 | *RACGAP1* | proliferating cells (MKi67+) |
| KIF15 | 0 | 0.722413 | 0.723 | 0.046 | 0 | 18 | *KIF15* | proliferating cells (MKi67+) |
| AC084033. | 1.19E-241 | 0.718638 | 0.803 | 0.24 | 2.56E-237 | 18 | *AC084033.* | proliferating cells (MKi67+) |
| ASCL1 | 4.63E-182 | 0.718362 | 0.598 | 0.152 | 9.92E-178 | 18 | *ASCL1* | proliferating cells (MKi67+) |
| RBMX | 1.67E-168 | 0.715851 | 0.961 | 0.594 | 3.58E-164 | 18 | *RBMX* | proliferating cells (MKi67+) |
| KIF18A | 0 | 0.71308 | 0.677 | 0.029 | 0 | 18 | *KIF18A* | proliferating cells (MKi67+) |
| FAM83D | 0 | 0.710633 | 0.675 | 0.017 | 0 | 18 | *FAM83D* | proliferating cells (MKi67+) |
| RAN | 2.69E-181 | 0.710177 | 0.988 | 0.804 | 5.78E-177 | 18 | *RAN* | proliferating cells (MKi67+) |
| BUB1B | 0 | 0.705718 | 0.72 | 0.035 | 0 | 18 | *BUB1B* | proliferating cells (MKi67+) |
| HES63 | 9.54E-23 | 0.702778 | 0.721 | 0.595 | 2.05E-18 | 18 | *HES6* | proliferating cells (MKi67+) |
| PIF1 | 0 | 0.700402 | 0.632 | 0.007 | 0 | 18 | *PIF1* | proliferating cells (MKi67+) |
| TACC31 | 0 | 0.693152 | 0.729 | 0.066 | 0 | 18 | *TACC3* | proliferating cells (MKi67+) |
| STMN11 | 8.03E-179 | 0.689594 | 1 | 0.953 | 1.72E-174 | 18 | *STMN1* | proliferating cells (MKi67+) |
| NCAPD2 | 0 | 0.67752 | 0.735 | 0.078 | 0 | 18 | *NCAPD2* | proliferating cells (MKi67+) |
| ARHGAP11 | 0 | 0.675552 | 0.654 | 0.031 | 0 | 18 | *ARHGAP11* | proliferating cells (MKi67+) |

| NUDCD2 | 9.27E-229 | 0.667107 | 0.791 | 0.24 | 1.99E-224 | 18 | *NUDCD2* | proliferating cells (MKi67+) |
| --- | --- | --- | --- | --- | --- | --- | --- | --- |
| CRNDE | 2.19E-247 | 0.662375 | 0.847 | 0.241 | 4.69E-243 | 18 | *CRNDE* | proliferating cells (MKi67+) |
| KMT5A | 0 | 0.658989 | 0.708 | 0.096 | 0 | 18 | *KMT5A* | proliferating cells (MKi67+) |
| PSRC1 | 0 | 0.658589 | 0.685 | 0.058 | 0 | 18 | *PSRC1* | proliferating cells (MKi67+) |
| LBR | 1.21E-246 | 0.648398 | 0.795 | 0.223 | 2.59E-242 | 18 | *LBR* | proliferating cells (MKi67+) |
| HMG20B | 3.65E-260 | 0.647681 | 0.745 | 0.184 | 7.83E-256 | 18 | *HMG20B* | proliferating cells (MKi67+) |
| CENPW | 0 | 0.642642 | 0.681 | 0.06 | 0 | 18 | *CENPW* | proliferating cells (MKi67+) |
| SMC21 | 6.20E-263 | 0.636713 | 0.76 | 0.182 | 1.33E-258 | 18 | *SMC2* | proliferating cells (MKi67+) |
| NCAPG1 | 0 | 0.629652 | 0.662 | 0.062 | 0 | 18 | *NCAPG* | proliferating cells (MKi67+) |
| DEPDC1B | 0 | 0.622391 | 0.671 | 0.047 | 0 | 18 | *DEPDC1B* | proliferating cells (MKi67+) |
| G2E3 | 1.75E-274 | 0.620139 | 0.731 | 0.166 | 3.74E-270 | 18 | *G2E3* | proliferating cells (MKi67+) |
| TUBB2 | 2.93E-128 | 0.604522 | 0.99 | 0.91 | 6.29E-124 | 18 | *TUBB* | proliferating cells (MKi67+) |
| CCDC88A1 | 1.60E-131 | 0.601405 | 0.861 | 0.428 | 3.44E-127 | 18 | *CCDC88A* | proliferating cells (MKi67+) |
| TMSB15A2 | 4.01E-134 | 0.591559 | 0.894 | 0.419 | 8.59E-130 | 18 | *TMSB15A* | proliferating cells (MKi67+) |
| FBXO51 | 2.97E-213 | 0.586452 | 0.64 | 0.152 | 6.36E-209 | 18 | *FBXO5* | proliferating cells (MKi67+) |
| EMC9 | 8.27E-232 | 0.585008 | 0.737 | 0.193 | 1.77E-227 | 18 | *EMC9* | proliferating cells (MKi67+) |
| SPDL1 | 0 | 0.575469 | 0.646 | 0.067 | 0 | 18 | *SPDL1* | proliferating cells (MKi67+) |
| BRD8 | 4.86E-177 | 0.572837 | 0.698 | 0.216 | 1.04E-172 | 18 | *BRD8* | proliferating cells (MKi67+) |
| PCM1 | 1.08E-101 | 0.571369 | 0.899 | 0.579 | 2.31E-97 | 18 | *PCM1* | proliferating cells (MKi67+) |
| CBX1 | 6.78E-118 | 0.569994 | 0.936 | 0.623 | 1.45E-113 | 18 | *CBX1* | proliferating cells (MKi67+) |
| MYEF2 | 7.01E-132 | 0.569981 | 0.836 | 0.402 | 1.50E-127 | 18 | *MYEF2* | proliferating cells (MKi67+) |
| GPSM2 | 2.84E-276 | 0.568586 | 0.71 | 0.151 | 6.09E-272 | 18 | *GPSM2* | proliferating cells (MKi67+) |
| CNTRL | 2.87E-254 | 0.558714 | 0.615 | 0.122 | 6.16E-250 | 18 | *CNTRL* | proliferating cells (MKi67+) |
| TRIM59 | 0 | 0.54864 | 0.565 | 0.037 | 0 | 18 | *TRIM59* | proliferating cells (MKi67+) |
| CDKN2C | 0 | 0.547601 | 0.603 | 0.086 | 0 | 18 | *CDKN2C* | proliferating cells (MKi67+) |
| ILF2 | 5.59E-123 | 0.546373 | 0.874 | 0.465 | 1.20E-118 | 18 | *ILF2* | proliferating cells (MKi67+) |
| DCAF7 | 1.23E-129 | 0.54384 | 0.81 | 0.371 | 2.64E-125 | 18 | *DCAF7* | proliferating cells (MKi67+) |
| KIF22 | 1.32E-185 | 0.542915 | 0.766 | 0.239 | 2.83E-181 | 18 | *KIF22* | proliferating cells (MKi67+) |
| KIF5B | 4.17E-99 | 0.542175 | 0.909 | 0.594 | 8.94E-95 | 18 | *KIF5B* | proliferating cells (MKi67+) |
| CIT | 0 | 0.53852 | 0.578 | 0.043 | 0 | 18 | *CIT* | proliferating cells (MKi67+) |
| CDKN2D | 2.09E-222 | 0.537913 | 0.609 | 0.131 | 4.49E-218 | 18 | *CDKN2D* | proliferating cells (MKi67+) |
| TUBB6 | 2.03E-232 | 0.537752 | 0.627 | 0.135 | 4.36E-228 | 18 | *TUBB6* | proliferating cells (MKi67+) |
| HSD17B11 | 2.32E-281 | 0.535705 | 0.621 | 0.113 | 4.98E-277 | 18 | *HSD17B11* | proliferating cells (MKi67+) |
| GPX4 | 9.67E-107 | 0.529267 | 0.986 | 0.87 | 2.07E-102 | 18 | *GPX4* | proliferating cells (MKi67+) |
| ANLN | 0 | 0.528878 | 0.565 | 0.03 | 0 | 18 | *ANLN* | proliferating cells (MKi67+) |
| PNRC2 | 8.37E-130 | 0.524434 | 0.787 | 0.348 | 1.80E-125 | 18 | *PNRC2* | proliferating cells (MKi67+) |
| NDE1 | 0 | 0.524222 | 0.574 | 0.05 | 0 | 18 | *NDE1* | proliferating cells (MKi67+) |
| RNF26 | 0 | 0.523934 | 0.596 | 0.074 | 0 | 18 | *RNF26* | proliferating cells (MKi67+) |
| C21orf581 | 4.74E-267 | 0.523632 | 0.642 | 0.122 | 1.02E-262 | 18 | *C21orf58* | proliferating cells (MKi67+) |
| NAV2 | 2.01E-222 | 0.517917 | 0.578 | 0.119 | 4.30E-218 | 18 | *NAV2* | proliferating cells (MKi67+) |
| PTMS | 3.62E-99 | 0.514562 | 0.99 | 0.906 | 7.76E-95 | 18 | *PTMS* | proliferating cells (MKi67+) |
| HMGN3 | 1.08E-108 | 0.513506 | 0.971 | 0.787 | 2.31E-104 | 18 | *HMGN3* | proliferating cells (MKi67+) |
| SPA17 | 0 | 0.513426 | 0.555 | 0.038 | 0 | 18 | *SPA17* | proliferating cells (MKi67+) |
| CEP135 | 0 | 0.512303 | 0.586 | 0.077 | 0 | 18 | *CEP135* | proliferating cells (MKi67+) |
| FRMD4B | 2.39E-140 | 0.511305 | 0.642 | 0.212 | 5.12E-136 | 18 | *FRMD4B* | proliferating cells (MKi67+) |
| BORA | 0 | 0.508757 | 0.511 | 0.021 | 0 | 18 | *BORA* | proliferating cells (MKi67+) |
| TUBA1A1 | 3.13E-103 | 0.508137 | 0.996 | 0.939 | 6.71E-99 | 18 | *TUBA1A* | proliferating cells (MKi67+) |
| CCDC18 | 0 | 0.506507 | 0.528 | 0.038 | 0 | 18 | *CCDC18* | proliferating cells (MKi67+) |
| H1FX1 | 1.58E-76 | 0.506405 | 0.923 | 0.722 | 3.38E-72 | 18 | *H1FX* | proliferating cells (MKi67+) |
| KHDRBS1 | 2.72E-101 | 0.501477 | 0.975 | 0.759 | 5.82E-97 | 18 | *KHDRBS1* | proliferating cells (MKi67+) |
| SCG34 | 1.93E-125 | 0.742412 | 0.901 | 0.404 | 4.15E-121 | 19 | *SCG3* | Cone precursors |
| ARF41 | 1.30E-58 | 0.730083 | 0.723 | 0.502 | 2.78E-54 | 19 | *ARF4* | Cone precursors |
| DCT1 | 1.32E-128 | 0.657993 | 0.584 | 0.169 | 2.82E-124 | 19 | *DCT* | Cone precursors |
| RRAD2 | 4.10E-73 | 0.647217 | 0.693 | 0.324 | 8.80E-69 | 19 | *RRAD* | Cone precursors |
| YIF1A1 | 5.48E-76 | 0.641911 | 0.655 | 0.335 | 1.17E-71 | 19 | *YIF1A* | Cone precursors |
| FAM57B3 | 7.35E-137 | 0.615995 | 0.843 | 0.312 | 1.58E-132 | 19 | *FAM57B* | Cone precursors |
| AANAT3 | 1.90E-120 | 0.566561 | 0.631 | 0.201 | 4.08E-116 | 19 | *AANAT* | Cone precursors |
| PDC3 | 8.57E-91 | 0.548783 | 0.906 | 0.454 | 1.84E-86 | 19 | *PDC* | Cone precursors |
| GUK12 | 4.29E-80 | 0.516115 | 0.951 | 0.716 | 9.19E-76 | 19 | *GUK1* | Cone precursors |
| MAP1LC3A | 2.14E-71 | 0.50924 | 0.702 | 0.336 | 4.60E-67 | 19 | *MAP1LC3A* | Cone precursors |
| TPT1 | 1.03E-38 | 0.505814 | 1 | 1 | 2.21E-34 | 19 | *TPT1* | Cone precursors |
| PCBP44 | 1.03E-91 | 0.504814 | 0.841 | 0.389 | 2.20E-87 | 19 | *PCBP4* | Cone precursors |
| GNB33 | 7.99E-98 | 0.501004 | 0.845 | 0.356 | 1.71E-93 | 19 | *GNB3* | Cone precursors |

| STMN21 | 0 | 1.542601 | 0.886 | 0.223 | 0 | 20 | *STMN2* | Retinal ganglion cells |
| --- | --- | --- | --- | --- | --- | --- | --- | --- |
| HOXB51 | 5.76E-126 | 1.201841 | 0.424 | 0.1 | 1.23E-121 | 20 | *HOXB5* | Retinal ganglion cells |
| SOX112 | 2.78E-183 | 1.162923 | 0.892 | 0.443 | 5.95E-179 | 20 | *SOX11* | Retinal ganglion cells |
| TUBA1A2 | 2.45E-163 | 1.13952 | 0.998 | 0.939 | 5.25E-159 | 20 | *TUBA1A* | Retinal ganglion cells |
| BASP12 | 3.04E-176 | 1.052941 | 0.96 | 0.668 | 6.52E-172 | 20 | *BASP1* | Retinal ganglion cells |
| SOX43 | 2.38E-151 | 0.957165 | 0.993 | 0.86 | 5.11E-147 | 20 | *SOX4* | Retinal ganglion cells |
| TUBB2B1 | 3.17E-128 | 0.926101 | 0.936 | 0.709 | 6.81E-124 | 20 | *TUBB2B* | Retinal ganglion cells |
| HOXB8 | 8.12E-195 | 0.874567 | 0.371 | 0.052 | 1.74E-190 | 20 | *HOXB8* | Retinal ganglion cells |
| TMSB101 | 1.21E-140 | 0.871818 | 1 | 0.966 | 2.59E-136 | 20 | *TMSB10* | Retinal ganglion cells |
| GAP43 | 2.40E-155 | 0.863787 | 0.512 | 0.122 | 5.14E-151 | 20 | *GAP43* | Retinal ganglion cells |
| CNTN2 | 9.37E-247 | 0.838744 | 0.415 | 0.052 | 2.01E-242 | 20 | *CNTN2* | Retinal ganglion cells |
| MLLT111 | 3.36E-135 | 0.810238 | 0.903 | 0.516 | 7.20E-131 | 20 | *MLLT11* | Retinal ganglion cells |
| STMN41 | 9.33E-154 | 0.785928 | 0.589 | 0.16 | 2.00E-149 | 20 | *STMN4* | Retinal ganglion cells |
| CRABP12 | 8.30E-43 | 0.736771 | 0.963 | 0.84 | 1.78E-38 | 20 | *CRABP1* | Retinal ganglion cells |
| DCX1 | 7.60E-171 | 0.729797 | 0.615 | 0.158 | 1.63E-166 | 20 | *DCX* | Retinal ganglion cells |
| CD241 | 8.46E-144 | 0.716409 | 0.796 | 0.319 | 1.82E-139 | 20 | *CD24* | Retinal ganglion cells |
| TUBB2A1 | 5.53E-78 | 0.636745 | 0.602 | 0.27 | 1.19E-73 | 20 | *TUBB2A* | Retinal ganglion cells |
| KCNQ1OT1 | 1.09E-39 | 0.586937 | 0.864 | 0.687 | 2.34E-35 | 20 | *KCNQ1OT1* | Retinal ganglion cells |
| MIAT3 | 1.52E-56 | 0.586488 | 0.758 | 0.454 | 3.26E-52 | 20 | *MIAT* | Retinal ganglion cells |
| HOXB6 | 8.74E-91 | 0.584929 | 0.253 | 0.047 | 1.87E-86 | 20 | *HOXB6* | Retinal ganglion cells |
| NNAT1 | 5.74E-55 | 0.582494 | 0.686 | 0.392 | 1.23E-50 | 20 | *NNAT* | Retinal ganglion cells |
| UCHL1 | 1.03E-39 | 0.571509 | 0.719 | 0.535 | 2.21E-35 | 20 | *UCHL1* | Retinal ganglion cells |
| JPT12 | 1.82E-80 | 0.568258 | 0.826 | 0.501 | 3.90E-76 | 20 | *JPT1* | Retinal ganglion cells |
| GNG3 | 1.79E-196 | 0.545753 | 0.453 | 0.074 | 3.84E-192 | 20 | *GNG3* | Retinal ganglion cells |
| FNBP1L | 3.36E-63 | 0.507932 | 0.673 | 0.373 | 7.21E-59 | 20 | *FNBP1L* | Retinal ganglion cells |
| TRH2 | 1.11E-122 | 1.052104 | 0.961 | 0.618 | 2.38E-118 | 21 | *TRH* | Proliferating MG (MKi67-) |
| PTN2 | 1.59E-141 | 0.964955 | 0.896 | 0.448 | 3.41E-137 | 21 | *PTN* | Proliferating MG (MKi67-) |
| CRYM2 | 8.86E-197 | 0.864254 | 0.863 | 0.28 | 1.90E-192 | 21 | *CRYM* | Proliferating MG (MKi67-) |
| METRN1 | 9.82E-122 | 0.818921 | 0.794 | 0.335 | 2.11E-117 | 21 | *METRN* | Proliferating MG (MKi67-) |
| LAMP52 | 8.35E-190 | 0.802046 | 0.893 | 0.288 | 1.79E-185 | 21 | *LAMP5* | Proliferating MG (MKi67-) |
| TF4 | 1.21E-42 | 0.776159 | 0.652 | 0.385 | 2.59E-38 | 21 | *TF* | Proliferating MG (MKi67-) |
| GPC32 | 9.29E-171 | 0.758686 | 0.784 | 0.236 | 1.99E-166 | 21 | *GPC3* | Proliferating MG (MKi67-) |
| GAD2 | 6.01E-268 | 0.75608 | 0.596 | 0.094 | 1.29E-263 | 21 | *GAD2* | Proliferating MG (MKi67-) |
| TYMS3 | 2.51E-151 | 0.753179 | 0.879 | 0.335 | 5.39E-147 | 21 | *TYMS* | Proliferating MG (MKi67-) |
| PCLAF3 | 1.02E-162 | 0.731899 | 0.74 | 0.213 | 2.19E-158 | 21 | *PCLAF* | Proliferating MG (MKi67-) |
| PCNA3 | 8.44E-148 | 0.720536 | 0.838 | 0.33 | 1.81E-143 | 21 | *PCNA* | Proliferating MG (MKi67-) |
| SFRP24 | 3.99E-73 | 0.70953 | 0.965 | 0.703 | 8.56E-69 | 21 | *SFRP2* | Proliferating MG (MKi67-) |
| GSN | 6.44E-140 | 0.640684 | 0.673 | 0.21 | 1.38E-135 | 21 | *GSN* | Proliferating MG (MKi67-) |
| SLBP2 | 8.37E-143 | 0.629691 | 0.796 | 0.291 | 1.79E-138 | 21 | *SLBP* | Proliferating MG (MKi67-) |
| WIF12 | 7.60E-128 | 0.628287 | 0.761 | 0.261 | 1.63E-123 | 21 | *WIF1* | Proliferating MG (MKi67-) |
| SRPRB1 | 3.65E-84 | 0.625738 | 0.61 | 0.249 | 7.83E-80 | 21 | *SRPRB* | Proliferating MG (MKi67-) |
| AGL2 | 2.14E-98 | 0.613096 | 0.845 | 0.402 | 4.59E-94 | 21 | *AGL* | Proliferating MG (MKi67-) |
| FAM111B3 | 7.85E-149 | 0.602474 | 0.661 | 0.181 | 1.68E-144 | 21 | *FAM111B* | Proliferating MG (MKi67-) |
| CRABP13 | 6.59E-90 | 0.600093 | 1 | 0.84 | 1.41E-85 | 21 | *CRABP1* | Proliferating MG (MKi67-) |
| THY11 | 1.38E-169 | 0.597213 | 0.645 | 0.159 | 2.97E-165 | 21 | *THY1* | Proliferating MG (MKi67-) |
| VIM5 | 3.01E-90 | 0.594032 | 1 | 0.854 | 6.46E-86 | 21 | *VIM* | Proliferating MG (MKi67-) |
| PAX21 | 3.22E-203 | 0.591758 | 0.64 | 0.132 | 6.91E-199 | 21 | *PAX2* | Proliferating MG (MKi67-) |
| FABP73 | 3.26E-84 | 0.588084 | 0.912 | 0.539 | 6.98E-80 | 21 | *FABP7* | Proliferating MG (MKi67-) |
| TUBA1B4 | 3.20E-102 | 0.58133 | 0.998 | 0.889 | 6.87E-98 | 21 | *TUBA1B* | Proliferating MG (MKi67-) |
| COL18A11 | 2.61E-115 | 0.563766 | 0.719 | 0.266 | 5.60E-111 | 21 | *COL18A1* | Proliferating MG (MKi67-) |
| CYP26A13 | 3.32E-65 | 0.541332 | 0.914 | 0.552 | 7.11E-61 | 21 | *CYP26A1* | Proliferating MG (MKi67-) |
| DUT3 | 6.33E-73 | 0.533069 | 0.828 | 0.473 | 1.36E-68 | 21 | *DUT* | Proliferating MG (MKi67-) |
| HELLS3 | 6.25E-110 | 0.532804 | 0.729 | 0.264 | 1.34E-105 | 21 | *HELLS* | Proliferating MG (MKi67-) |
| TMEM106 | 1.42E-83 | 0.520312 | 0.726 | 0.329 | 3.04E-79 | 21 | *TMEM106* | Proliferating MG (MKi67-) |
| GAPDH | 1.08E-107 | 0.514616 | 1 | 0.994 | 2.31E-103 | 21 | *GAPDH* | Proliferating MG (MKi67-) |
| RANBP12 | 1.34E-76 | 0.510091 | 0.921 | 0.607 | 2.88E-72 | 21 | *RANBP1* | Proliferating MG (MKi67-) |
| SFRP11 | 2.29E-89 | 0.504946 | 0.559 | 0.188 | 4.92E-85 | 21 | *SFRP1* | Proliferating MG (MKi67-) |
| CNTLN | 1.24E-83 | 0.500981 | 0.854 | 0.452 | 2.66E-79 | 21 | *CNTLN* | Proliferating MG (MKi67-) |
| ATP1B1 | 5.12E-177 | 1.113362 | 0.969 | 0.47 | 1.10E-172 | 22 | *ATP1B1* | Retinoblastoma-like |
| SCG35 | 5.64E-173 | 1.014606 | 0.983 | 0.405 | 1.21E-168 | 22 | *SCG3* | Retinoblastoma-like |
| DEK2 | 3.34E-174 | 0.99895 | 0.994 | 0.776 | 7.15E-170 | 22 | *DEK* | Retinoblastoma-like |
| H2AFY1 | 7.32E-170 | 0.962387 | 0.983 | 0.623 | 1.57E-165 | 22 | *H2AFY* | Retinoblastoma-like |
| AKAP93 | 2.98E-130 | 0.921237 | 0.983 | 0.622 | 6.38E-126 | 22 | *AKAP9* | Retinoblastoma-like |

| PDC4 | 1.73E-136 | 0.890753 | 0.986 | 0.455 | 3.71E-132 | 22 | *PDC* | Retinoblastoma-like |
| --- | --- | --- | --- | --- | --- | --- | --- | --- |
| PRKDC1 | 5.56E-150 | 0.884352 | 0.961 | 0.53 | 1.19E-145 | 22 | *PRKDC* | Retinoblastoma-like |
| GNG4 | 0 | 0.871076 | 0.809 | 0.11 | 0 | 22 | *GNG4* | Retinoblastoma-like |
| APOC1 | 2.21E-223 | 0.868147 | 0.652 | 0.117 | 4.75E-219 | 22 | *APOC1* | Retinoblastoma-like |
| TUBB3 | 6.09E-137 | 0.859287 | 1 | 0.911 | 1.31E-132 | 22 | *TUBB* | Retinoblastoma-like |
| VXN2 | 7.23E-181 | 0.846854 | 0.722 | 0.166 | 1.55E-176 | 22 | *VXN* | Retinoblastoma-like |
| CLSTN21 | 8.32E-268 | 0.839332 | 0.747 | 0.13 | 1.78E-263 | 22 | *CLSTN2* | Retinoblastoma-like |
| RAI14 | 1.01E-175 | 0.819592 | 0.888 | 0.304 | 2.17E-171 | 22 | *RAI14* | Retinoblastoma-like |
| TMEM97 | 3.13E-213 | 0.812906 | 0.885 | 0.249 | 6.71E-209 | 22 | *TMEM97* | Retinoblastoma-like |
| TUBA4A2 | 1.68E-174 | 0.812035 | 0.888 | 0.273 | 3.60E-170 | 22 | *TUBA4A* | Retinoblastoma-like |
| UQCC21 | 1.91E-150 | 0.811227 | 0.969 | 0.493 | 4.10E-146 | 22 | *UQCC2* | Retinoblastoma-like |
| HACD3 | 1.80E-137 | 0.803891 | 0.978 | 0.599 | 3.85E-133 | 22 | *HACD3* | Retinoblastoma-like |
| WFDC2 | 1.33E-151 | 0.802878 | 0.803 | 0.255 | 2.85E-147 | 22 | *WFDC2* | Retinoblastoma-like |
| RRAD3 | 6.56E-99 | 0.787367 | 0.809 | 0.324 | 1.41E-94 | 22 | *RRAD* | Retinoblastoma-like |
| PDE6H2 | 9.02E-97 | 0.785847 | 0.848 | 0.4 | 1.93E-92 | 22 | *PDE6H* | Retinoblastoma-like |
| MYLK | 0 | 0.781627 | 0.719 | 0.096 | 0 | 22 | *MYLK* | Retinoblastoma-like |
| PCLAF4 | 1.95E-204 | 0.781622 | 0.868 | 0.213 | 4.19E-200 | 22 | *PCLAF* | Retinoblastoma-like |
| ENO23 | 9.59E-135 | 0.774681 | 0.98 | 0.487 | 2.06E-130 | 22 | *ENO2* | Retinoblastoma-like |
| NEUROD13 | 5.63E-144 | 0.7703 | 0.893 | 0.299 | 1.21E-139 | 22 | *NEUROD1* | Retinoblastoma-like |
| PSD3 | 2.93E-174 | 0.762708 | 0.82 | 0.243 | 6.29E-170 | 22 | *PSD3* | Retinoblastoma-like |
| CPLX3 | 0 | 0.752913 | 0.75 | 0.108 | 0 | 22 | *CPLX3* | Retinoblastoma-like |
| TPH11 | 4.62E-252 | 0.746582 | 0.596 | 0.083 | 9.92E-248 | 22 | *TPH1* | Retinoblastoma-like |
| ACYP1 | 1.00E-176 | 0.745136 | 0.84 | 0.259 | 2.15E-172 | 22 | *ACYP1* | Retinoblastoma-like |
| TMX41 | 1.44E-102 | 0.739946 | 0.882 | 0.414 | 3.08E-98 | 22 | *TMX4* | Retinoblastoma-like |
| FADS11 | 4.13E-143 | 0.734806 | 0.913 | 0.387 | 8.86E-139 | 22 | *FADS1* | Retinoblastoma-like |
| ARR31 | 2.81E-164 | 0.734132 | 0.61 | 0.127 | 6.02E-160 | 22 | *ARR3* | Retinoblastoma-like |
| TMEM106 | 4.13E-149 | 0.733854 | 0.89 | 0.328 | 8.86E-145 | 22 | *TMEM106* | Retinoblastoma-like |
| RBP41 | 0 | 0.724274 | 0.764 | 0.086 | 0 | 22 | *RBP4* | Retinoblastoma-like |
| CBX5 | 8.06E-113 | 0.714756 | 0.935 | 0.544 | 1.73E-108 | 22 | *CBX5* | Retinoblastoma-like |
| PPIA2 | 1.38E-130 | 0.713422 | 0.992 | 0.903 | 2.96E-126 | 22 | *PPIA* | Retinoblastoma-like |
| NEGR1 | 0 | 0.711754 | 0.697 | 0.065 | 0 | 22 | *NEGR1* | Retinoblastoma-like |
| HIPK2 | 4.81E-138 | 0.709492 | 0.831 | 0.303 | 1.03E-133 | 22 | *HIPK2* | Retinoblastoma-like |
| SMC3 | 4.27E-121 | 0.708187 | 0.972 | 0.588 | 9.17E-117 | 22 | *SMC3* | Retinoblastoma-like |
| RYBP | 3.84E-158 | 0.707789 | 0.823 | 0.263 | 8.23E-154 | 22 | *RYBP* | Retinoblastoma-like |
| CNTNAP21 | 4.05E-182 | 0.706052 | 0.792 | 0.206 | 8.68E-178 | 22 | *CNTNAP2* | Retinoblastoma-like |
| FAM111B4 | 1.98E-197 | 0.700343 | 0.792 | 0.18 | 4.24E-193 | 22 | *FAM111B* | Retinoblastoma-like |
| HMGA1 | 1.64E-118 | 0.694904 | 0.888 | 0.406 | 3.52E-114 | 22 | *HMGA1* | Retinoblastoma-like |
| TYMS4 | 2.52E-136 | 0.69197 | 0.924 | 0.336 | 5.41E-132 | 22 | *TYMS* | Retinoblastoma-like |
| MAP23 | 1.05E-106 | 0.686474 | 0.924 | 0.434 | 2.24E-102 | 22 | *MAP2* | Retinoblastoma-like |
| EPB411 | 1.43E-117 | 0.685017 | 0.921 | 0.416 | 3.07E-113 | 22 | *EPB41* | Retinoblastoma-like |
| RGS163 | 2.40E-55 | 0.682849 | 0.882 | 0.587 | 5.15E-51 | 22 | *RGS16* | Retinoblastoma-like |
| AIPL13 | 4.13E-140 | 0.678758 | 0.896 | 0.28 | 8.85E-136 | 22 | *AIPL1* | Retinoblastoma-like |
| CHODL2 | 2.99E-170 | 0.670204 | 0.607 | 0.119 | 6.41E-166 | 22 | *CHODL* | Retinoblastoma-like |
| DPYSL32 | 4.59E-153 | 0.667406 | 0.837 | 0.242 | 9.85E-149 | 22 | *DPYSL3* | Retinoblastoma-like |
| TMEM70 | 1.36E-112 | 0.664453 | 0.736 | 0.257 | 2.91E-108 | 22 | *TMEM70* | Retinoblastoma-like |
| SCG5 | 4.92E-187 | 0.663516 | 0.663 | 0.137 | 1.05E-182 | 22 | *SCG5* | Retinoblastoma-like |
| ENO3 | 0 | 0.661608 | 0.68 | 0.079 | 0 | 22 | *ENO3* | Retinoblastoma-like |
| OCIAD2 | 8.42E-103 | 0.658529 | 0.888 | 0.466 | 1.81E-98 | 22 | *OCIAD2* | Retinoblastoma-like |
| PCNA4 | 6.49E-135 | 0.657731 | 0.89 | 0.331 | 1.39E-130 | 22 | *PCNA* | Retinoblastoma-like |
| MAP1LC3A | 1.51E-126 | 0.657266 | 0.896 | 0.335 | 3.23E-122 | 22 | *MAP1LC3A* | Retinoblastoma-like |
| SLC20A1 | 2.34E-203 | 0.656712 | 0.778 | 0.18 | 5.01E-199 | 22 | *SLC20A1* | Retinoblastoma-like |
| GUK13 | 5.29E-96 | 0.654782 | 0.98 | 0.716 | 1.13E-91 | 22 | *GUK1* | Retinoblastoma-like |
| STMN12 | 1.71E-112 | 0.650097 | 0.994 | 0.953 | 3.68E-108 | 22 | *STMN1* | Retinoblastoma-like |
| PRCD1 | 1.70E-163 | 0.648232 | 0.711 | 0.178 | 3.65E-159 | 22 | *PRCD* | Retinoblastoma-like |
| SLFN13 | 0 | 0.648214 | 0.728 | 0.093 | 0 | 22 | *SLFN13* | Retinoblastoma-like |
| NRN11 | 8.89E-140 | 0.648022 | 0.677 | 0.174 | 1.91E-135 | 22 | *NRN1* | Retinoblastoma-like |
| OLFM11 | 6.10E-225 | 0.647482 | 0.725 | 0.137 | 1.31E-220 | 22 | *OLFM1* | Retinoblastoma-like |
| SLC1A21 | 5.76E-196 | 0.639564 | 0.795 | 0.184 | 1.24E-191 | 22 | *SLC1A2* | Retinoblastoma-like |
| HELLS4 | 3.88E-156 | 0.639271 | 0.868 | 0.263 | 8.33E-152 | 22 | *HELLS* | Retinoblastoma-like |
| HSPB111 | 1.85E-139 | 0.638826 | 0.837 | 0.29 | 3.97E-135 | 22 | *HSPB11* | Retinoblastoma-like |
| TCOF1 | 1.35E-165 | 0.636016 | 0.624 | 0.136 | 2.89E-161 | 22 | *TCOF1* | Retinoblastoma-like |
| PHOX2A | 0 | 0.6324 | 0.61 | 0.061 | 0 | 22 | *PHOX2A* | Retinoblastoma-like |
| PLEKHB11 | 1.39E-138 | 0.631117 | 0.767 | 0.228 | 2.97E-134 | 22 | *PLEKHB1* | Retinoblastoma-like |

| GNG8 | 0 | 0.628317 | 0.525 | 0.037 | 0 | 22 | *GNG8* | Retinoblastoma-like |
| --- | --- | --- | --- | --- | --- | --- | --- | --- |
| FAM213A | 2.16E-107 | 0.628185 | 0.921 | 0.483 | 4.63E-103 | 22 | *FAM213A* | Retinoblastoma-like |
| ACTN1 | 4.54E-194 | 0.625953 | 0.691 | 0.147 | 9.73E-190 | 22 | *ACTN1* | Retinoblastoma-like |
| ADCY1 | 6.61E-214 | 0.6249 | 0.753 | 0.154 | 1.42E-209 | 22 | *ADCY1* | Retinoblastoma-like |
| CADPS1 | 5.68E-146 | 0.623246 | 0.806 | 0.23 | 1.22E-141 | 22 | *CADPS* | Retinoblastoma-like |
| CPE3 | 1.27E-95 | 0.622683 | 0.969 | 0.556 | 2.72E-91 | 22 | *CPE* | Retinoblastoma-like |
| SYNE21 | 4.23E-82 | 0.622404 | 0.924 | 0.557 | 9.08E-78 | 22 | *SYNE2* | Retinoblastoma-like |
| MIR7-3HG | 1.32E-171 | 0.621495 | 0.711 | 0.164 | 2.83E-167 | 22 | *MIR7-3HG* | Retinoblastoma-like |
| LPGAT1 | 1.81E-135 | 0.618105 | 0.798 | 0.26 | 3.88E-131 | 22 | *LPGAT1* | Retinoblastoma-like |
| CCNE21 | 2.72E-292 | 0.617505 | 0.716 | 0.105 | 5.82E-288 | 22 | *CCNE2* | Retinoblastoma-like |
| GGCT | 7.87E-143 | 0.614764 | 0.806 | 0.265 | 1.69E-138 | 22 | *GGCT* | Retinoblastoma-like |
| GIP | 0 | 0.614413 | 0.427 | 0.023 | 0 | 22 | *GIP* | Retinoblastoma-like |
| SELENOW | 1.07E-88 | 0.613731 | 0.958 | 0.68 | 2.28E-84 | 22 | *SELENOW* | Retinoblastoma-like |
| CALCOCO2 | 3.29E-120 | 0.613575 | 0.834 | 0.315 | 7.05E-116 | 22 | *CALCOCO2* | Retinoblastoma-like |
| RBBP71 | 6.47E-104 | 0.609781 | 0.885 | 0.428 | 1.39E-99 | 22 | *RBBP7* | Retinoblastoma-like |
| COBLL11 | 1.37E-170 | 0.608075 | 0.798 | 0.204 | 2.94E-166 | 22 | *COBLL1* | Retinoblastoma-like |
| GNB5 | 2.54E-132 | 0.607633 | 0.837 | 0.298 | 5.44E-128 | 22 | *GNB5* | Retinoblastoma-like |
| SPARC | 8.57E-102 | 0.60702 | 0.77 | 0.303 | 1.84E-97 | 22 | *SPARC* | Retinoblastoma-like |
| PKIB1 | 2.84E-138 | 0.599856 | 0.73 | 0.202 | 6.08E-134 | 22 | *PKIB* | Retinoblastoma-like |
| MCM3 | 4.59E-164 | 0.599182 | 0.761 | 0.198 | 9.85E-160 | 22 | *MCM3* | Retinoblastoma-like |
| TECR | 7.46E-87 | 0.597767 | 0.879 | 0.502 | 1.60E-82 | 22 | *TECR* | Retinoblastoma-like |
| SUPT16H1 | 3.90E-94 | 0.597605 | 0.89 | 0.467 | 8.36E-90 | 22 | *SUPT16H* | Retinoblastoma-like |
| BTG31 | 7.24E-92 | 0.592722 | 0.902 | 0.457 | 1.55E-87 | 22 | *BTG3* | Retinoblastoma-like |
| WFIKKN2 | 0 | 0.592167 | 0.511 | 0.019 | 0 | 22 | *WFIKKN2* | Retinoblastoma-like |
| AHI1 | 2.04E-98 | 0.591751 | 0.86 | 0.405 | 4.37E-94 | 22 | *AHI1* | Retinoblastoma-like |
| MEIS22 | 3.69E-117 | 0.590831 | 0.916 | 0.376 | 7.92E-113 | 22 | *MEIS2* | Retinoblastoma-like |
| CLSPN1 | 8.91E-196 | 0.585188 | 0.739 | 0.156 | 1.91E-191 | 22 | *CLSPN* | Retinoblastoma-like |
| N4BP2 | 4.93E-107 | 0.579705 | 0.831 | 0.342 | 1.06E-102 | 22 | *N4BP2* | Retinoblastoma-like |
| ATP6V0B1 | 6.00E-92 | 0.578458 | 0.972 | 0.638 | 1.29E-87 | 22 | *ATP6V0B* | Retinoblastoma-like |
| FAM57B4 | 7.09E-118 | 0.577253 | 0.899 | 0.314 | 1.52E-113 | 22 | *FAM57B* | Retinoblastoma-like |
| KPNB11 | 4.26E-92 | 0.575987 | 0.913 | 0.506 | 9.13E-88 | 22 | *KPNB1* | Retinoblastoma-like |
| SLC38A12 | 3.92E-103 | 0.573956 | 0.893 | 0.366 | 8.41E-99 | 22 | *SLC38A1* | Retinoblastoma-like |
| ZNF83 | 2.47E-150 | 0.56897 | 0.733 | 0.203 | 5.30E-146 | 22 | *ZNF83* | Retinoblastoma-like |
| TUBA1B5 | 1.27E-80 | 0.565075 | 0.997 | 0.889 | 2.73E-76 | 22 | *TUBA1B* | Retinoblastoma-like |
| HRASLS2 | 2.33E-120 | 0.564115 | 0.694 | 0.202 | 5.00E-116 | 22 | *HRASLS* | Retinoblastoma-like |
| CARHSP11 | 4.49E-108 | 0.563113 | 0.817 | 0.322 | 9.62E-104 | 22 | *CARHSP1* | Retinoblastoma-like |
| PGAM1 | 4.69E-77 | 0.562784 | 0.93 | 0.584 | 1.01E-72 | 22 | *PGAM1* | Retinoblastoma-like |
| MFGE8 | 2.57E-125 | 0.562466 | 0.753 | 0.243 | 5.50E-121 | 22 | *MFGE8* | Retinoblastoma-like |
| PEX5L2 | 4.54E-176 | 0.559683 | 0.722 | 0.159 | 9.73E-172 | 22 | *PEX5L* | Retinoblastoma-like |
| PPP1R27 | 3.20E-162 | 0.559615 | 0.256 | 0.023 | 6.86E-158 | 22 | *PPP1R27* | Retinoblastoma-like |
| DHFR1 | 5.22E-160 | 0.558072 | 0.685 | 0.162 | 1.12E-155 | 22 | *DHFR* | Retinoblastoma-like |
| RRM12 | 1.88E-133 | 0.558044 | 0.809 | 0.255 | 4.04E-129 | 22 | *RRM1* | Retinoblastoma-like |
| PKP2 | 1.68E-261 | 0.553129 | 0.596 | 0.081 | 3.60E-257 | 22 | *PKP2* | Retinoblastoma-like |
| MLXIP2 | 8.71E-135 | 0.550085 | 0.73 | 0.201 | 1.87E-130 | 22 | *MLXIP* | Retinoblastoma-like |
| RTN3 | 8.56E-87 | 0.54874 | 0.963 | 0.637 | 1.83E-82 | 22 | *RTN3* | Retinoblastoma-like |
| SNX18 | 1.01E-186 | 0.548329 | 0.57 | 0.101 | 2.16E-182 | 22 | *SNX18* | Retinoblastoma-like |
| HNRNPAB1 | 1.63E-84 | 0.547822 | 0.924 | 0.541 | 3.49E-80 | 22 | *HNRNPAB* | Retinoblastoma-like |
| CASP8AP2 | 1.76E-113 | 0.547771 | 0.758 | 0.263 | 3.78E-109 | 22 | *CASP8AP2* | Retinoblastoma-like |
| MCM41 | 3.85E-145 | 0.547198 | 0.728 | 0.196 | 8.25E-141 | 22 | *MCM4* | Retinoblastoma-like |
| CRX3 | 2.52E-107 | 0.544532 | 0.823 | 0.278 | 5.41E-103 | 22 | *CRX* | Retinoblastoma-like |
| TTLL7 | 1.88E-132 | 0.543149 | 0.753 | 0.233 | 4.03E-128 | 22 | *TTLL7* | Retinoblastoma-like |
| RANBP13 | 2.84E-80 | 0.541573 | 0.944 | 0.608 | 6.08E-76 | 22 | *RANBP1* | Retinoblastoma-like |
| FEN11 | 1.86E-135 | 0.538334 | 0.775 | 0.234 | 3.98E-131 | 22 | *FEN1* | Retinoblastoma-like |
| NRXN11 | 5.95E-223 | 0.537863 | 0.607 | 0.095 | 1.28E-218 | 22 | *NRXN1* | Retinoblastoma-like |
| RBP7 | 3.09E-244 | 0.532604 | 0.553 | 0.074 | 6.62E-240 | 22 | *RBP7* | Retinoblastoma-like |
| PTPN132 | 4.36E-104 | 0.531849 | 0.711 | 0.234 | 9.35E-100 | 22 | *PTPN13* | Retinoblastoma-like |
| RAD23A | 3.41E-90 | 0.530559 | 0.857 | 0.433 | 7.31E-86 | 22 | *RAD23A* | Retinoblastoma-like |
| CAMK2B | 5.23E-199 | 0.530323 | 0.579 | 0.098 | 1.12E-194 | 22 | *CAMK2B* | Retinoblastoma-like |
| CHGB1 | 3.68E-172 | 0.527278 | 0.683 | 0.146 | 7.90E-168 | 22 | *CHGB* | Retinoblastoma-like |
| CRMP1 | 4.80E-141 | 0.527277 | 0.75 | 0.207 | 1.03E-136 | 22 | *CRMP1* | Retinoblastoma-like |
| WDR76 | 6.14E-188 | 0.527184 | 0.654 | 0.13 | 1.32E-183 | 22 | *WDR76* | Retinoblastoma-like |
| FAIM | 1.93E-138 | 0.525684 | 0.584 | 0.137 | 4.13E-134 | 22 | *FAIM* | Retinoblastoma-like |
| SLC25A4 | 5.61E-76 | 0.525164 | 0.801 | 0.405 | 1.20E-71 | 22 | *SLC25A4* | Retinoblastoma-like |

| RFC4 | 1.41E-138 | 0.524242 | 0.716 | 0.198 | 3.03E-134 | 22 | *RFC4* | Retinoblastoma-like |
| --- | --- | --- | --- | --- | --- | --- | --- | --- |
| DYNLT1 | 6.35E-74 | 0.524153 | 0.938 | 0.648 | 1.36E-69 | 22 | *DYNLT1* | Retinoblastoma-like |
| ANP32B | 3.39E-79 | 0.51803 | 0.868 | 0.457 | 7.26E-75 | 22 | *ANP32B* | Retinoblastoma-like |
| SLBP3 | 1.14E-115 | 0.517145 | 0.829 | 0.292 | 2.45E-111 | 22 | *SLBP* | Retinoblastoma-like |
| FAM107A1 | 3.42E-212 | 0.516223 | 0.654 | 0.113 | 7.32E-208 | 22 | *FAM107A* | Retinoblastoma-like |
| INSR | 9.54E-93 | 0.516126 | 0.68 | 0.243 | 2.05E-88 | 22 | *INSR* | Retinoblastoma-like |
| USP12 | 4.70E-81 | 0.515569 | 0.812 | 0.377 | 1.01E-76 | 22 | *USP1* | Retinoblastoma-like |
| FSTL52 | 1.45E-138 | 0.515311 | 0.756 | 0.2 | 3.11E-134 | 22 | *FSTL5* | Retinoblastoma-like |
| DTL | 1.34E-246 | 0.512502 | 0.598 | 0.085 | 2.87E-242 | 22 | *DTL* | Retinoblastoma-like |
| CELF41 | 1.73E-130 | 0.511195 | 0.795 | 0.236 | 3.71E-126 | 22 | *CELF4* | Retinoblastoma-like |
| ENPP2 | 2.64E-135 | 0.51097 | 0.48 | 0.092 | 5.65E-131 | 22 | *ENPP2* | Retinoblastoma-like |
| EIF1AX | 3.37E-73 | 0.510023 | 0.963 | 0.687 | 7.23E-69 | 22 | *EIF1AX* | Retinoblastoma-like |
| MTHFD2 | 2.20E-163 | 0.509379 | 0.652 | 0.144 | 4.72E-159 | 22 | *MTHFD2* | Retinoblastoma-like |
| DNMT1 | 5.08E-101 | 0.508764 | 0.787 | 0.303 | 1.09E-96 | 22 | *DNMT1* | Retinoblastoma-like |
| NFE2L3 | 0 | 0.507499 | 0.508 | 0.041 | 0 | 22 | *NFE2L3* | Retinoblastoma-like |
| CYP26B1 | 7.66E-106 | 0.506755 | 0.565 | 0.149 | 1.64E-101 | 22 | *CYP26B1* | Retinoblastoma-like |
| Sep-43 | 3.56E-89 | 0.503235 | 0.84 | 0.33 | 7.64E-85 | 22 | *Sep-04* | Retinoblastoma-like |
| SMC1A | 1.88E-85 | 0.503008 | 0.764 | 0.322 | 4.02E-81 | 22 | *SMC1A* | Retinoblastoma-like |
| IDH2 | 3.28E-119 | 0.502923 | 0.747 | 0.241 | 7.04E-115 | 22 | *IDH2* | Retinoblastoma-like |
| SCG2 | 0 | 0.501501 | 0.447 | 0.037 | 0 | 22 | *SCG2* | Retinoblastoma-like |
| HUNK | 5.86E-212 | 0.501385 | 0.635 | 0.11 | 1.26E-207 | 22 | *HUNK* | Retinoblastoma-like |
| RNF157 | 3.27E-152 | 0.500059 | 0.66 | 0.159 | 7.02E-148 | 22 | *RNF157* | Retinoblastoma-like |
| CYP26A14 | 3.24E-167 | 1.40311 | 0.983 | 0.552 | 6.95E-163 | 23 | *CYP26A1* | Muller Glia cells |
| HOXB52 | 0 | 1.254804 | 0.797 | 0.096 | 0 | 23 | *HOXB5* | Muller Glia cells |
| GPC33 | 5.67E-219 | 1.107632 | 0.873 | 0.237 | 1.22E-214 | 23 | *GPC3* | Muller Glia cells |
| HOXB61 | 0 | 1.045236 | 0.712 | 0.041 | 0 | 23 | *HOXB6* | Muller Glia cells |
| LAMP53 | 7.91E-121 | 0.901671 | 0.805 | 0.291 | 1.70E-116 | 23 | *LAMP5* | Muller Glia cells |
| HOXB2 | 7.42E-252 | 0.838937 | 0.743 | 0.136 | 1.59E-247 | 23 | *HOXB2* | Muller Glia cells |
| HOXB-AS3 | 0 | 0.825452 | 0.353 | 0.015 | 0 | 23 | *HOXB-AS3* | Muller Glia cells |
| HOXB81 | 0 | 0.811911 | 0.672 | 0.049 | 0 | 23 | *HOXB8* | Muller Glia cells |
| CCL2 | 2.43E-35 | 0.802844 | 0.215 | 0.059 | 5.22E-31 | 23 | *CCL2* | Muller Glia cells |
| DDR2 | 1.20E-208 | 0.761256 | 0.596 | 0.102 | 2.58E-204 | 23 | *DDR2* | Muller Glia cells |
| CYP26B11 | 7.63E-128 | 0.752285 | 0.59 | 0.148 | 1.64E-123 | 23 | *CYP26B1* | Muller Glia cells |
| HOXB4 | 0 | 0.736802 | 0.61 | 0.037 | 0 | 23 | *HOXB4* | Muller Glia cells |
| MEST | 1.40E-82 | 0.726411 | 0.87 | 0.461 | 2.99E-78 | 23 | *MEST* | Muller Glia cells |
| FOS3 | 2.49E-63 | 0.717699 | 0.946 | 0.66 | 5.34E-59 | 23 | *FOS* | Muller Glia cells |
| CRYM3 | 1.53E-100 | 0.71491 | 0.749 | 0.284 | 3.28E-96 | 23 | *CRYM* | Muller Glia cells |
| AGL3 | 1.39E-66 | 0.670696 | 0.788 | 0.404 | 2.98E-62 | 23 | *AGL* | Muller Glia cells |
| RBP11 | 2.12E-103 | 0.668073 | 1 | 0.961 | 4.55E-99 | 23 | *RBP1* | Muller Glia cells |
| ATP2B4 | 1.74E-103 | 0.66528 | 0.672 | 0.233 | 3.74E-99 | 23 | *ATP2B4* | Muller Glia cells |
| PDZRN3 | 1.61E-142 | 0.650345 | 0.647 | 0.167 | 3.45E-138 | 23 | *PDZRN3* | Muller Glia cells |
| SLC1A31 | 2.66E-122 | 0.624407 | 0.497 | 0.111 | 5.69E-118 | 23 | *SLC1A3* | Muller Glia cells |
| HES13 | 1.77E-60 | 0.604992 | 0.876 | 0.492 | 3.80E-56 | 23 | *HES1* | Muller Glia cells |
| CALM1 | 4.24E-50 | 0.583315 | 0.91 | 0.756 | 9.08E-46 | 23 | *CALM1* | Muller Glia cells |
| DHRS31 | 2.82E-82 | 0.583251 | 0.845 | 0.412 | 6.04E-78 | 23 | *DHRS3* | Muller Glia cells |
| HOXB3 | 1.68E-274 | 0.57856 | 0.542 | 0.064 | 3.59E-270 | 23 | *HOXB3* | Muller Glia cells |
| WWTR1 | 2.11E-115 | 0.569078 | 0.636 | 0.187 | 4.53E-111 | 23 | *WWTR1* | Muller Glia cells |
| LINC00461 | 2.23E-69 | 0.564145 | 0.726 | 0.323 | 4.78E-65 | 23 | *LINC00461* | Muller Glia cells |
| IER21 | 1.02E-47 | 0.540994 | 0.935 | 0.745 | 2.19E-43 | 23 | *IER2* | Muller Glia cells |
| EGR11 | 1.96E-50 | 0.520919 | 0.938 | 0.679 | 4.19E-46 | 23 | *EGR1* | Muller Glia cells |
| SPRY1 | 3.10E-153 | 0.509508 | 0.517 | 0.098 | 6.64E-149 | 23 | *SPRY1* | Muller Glia cells |
| AKAP94 | 2.42E-100 | 0.946517 | 0.99 | 0.622 | 5.20E-96 | 24 | *AKAP9* | Rod precursors |
| MPP42 | 9.98E-94 | 0.897045 | 0.59 | 0.181 | 2.14E-89 | 24 | *MPP4* | Rod precursors |
| ROM12 | 1.99E-47 | 0.818935 | 0.517 | 0.219 | 4.27E-43 | 24 | *ROM1* | Rod precursors |
| LINC00599 | 5.59E-89 | 0.795737 | 0.66 | 0.238 | 1.20E-84 | 24 | *LINC00599* | Rod precursors |
| MT-ND2 | 1.37E-67 | 0.77076 | 1 | 0.978 | 2.94E-63 | 24 | *MT-ND2* | Rod precursors |
| MAP24 | 6.91E-42 | 0.674239 | 0.692 | 0.438 | 1.48E-37 | 24 | *MAP2* | Rod precursors |
| RP12 | 2.58E-59 | 0.669566 | 0.479 | 0.165 | 5.54E-55 | 24 | *RP1* | Rod precursors |
| KCNQ1OT1 | 3.57E-37 | 0.653761 | 0.949 | 0.686 | 7.65E-33 | 24 | *KCNQ1OT1* | Rod precursors |
| MT-ATP6 | 1.11E-54 | 0.645712 | 1 | 0.993 | 2.39E-50 | 24 | *MT-ATP6* | Rod precursors |
| CPE4 | 8.21E-39 | 0.626573 | 0.79 | 0.559 | 1.76E-34 | 24 | *CPE* | Rod precursors |
| SCG36 | 7.71E-45 | 0.623771 | 0.733 | 0.409 | 1.65E-40 | 24 | *SCG3* | Rod precursors |
| MALAT1 | 2.19E-97 | 0.620296 | 1 | 1 | 4.69E-93 | 24 | *MALAT1* | Rod precursors |

| SYP4 | 9.33E-55 | 0.615104 | 0.638 | 0.288 | 2.00E-50 | 24 | *SYP* | Rod precursors |
| --- | --- | --- | --- | --- | --- | --- | --- | --- |
| IMPG23 | 1.35E-51 | 0.60417 | 0.517 | 0.209 | 2.89E-47 | 24 | *IMPG2* | Rod precursors |
| NTM | 2.72E-59 | 0.603195 | 0.517 | 0.197 | 5.84E-55 | 24 | *NTM* | Rod precursors |
| MT-ND1 | 1.03E-43 | 0.54898 | 1 | 0.982 | 2.21E-39 | 24 | *MT-ND1* | Rod precursors |
| XIST1 | 1.42E-17 | 0.540137 | 0.575 | 0.414 | 3.03E-13 | 24 | *XIST* | Rod precursors |
| UNC1193 | 8.26E-60 | 0.501952 | 0.889 | 0.497 | 1.77E-55 | 24 | *UNC119* | Rod precursors |
| PROM1 | 1.46E-37 | 0.501323 | 0.457 | 0.214 | 3.14E-33 | 24 | *PROM1* | Rod precursors |
| HIST1H4C2 | 3.09E-223 | 2.243048 | 0.98 | 0.365 | 6.63E-219 | 25 | *HIST1H4C* | Prolif. NRPCs (MKi67+) |
| TOP2A2 | 0 | 1.909978 | 1 | 0.185 | 0 | 25 | *TOP2A* | Prolif. NRPCs (MKi67+) |
| CENPF2 | 0 | 1.652511 | 1 | 0.189 | 0 | 25 | *CENPF* | Prolif. NRPCs (MKi67+) |
| HMGB23 | 1.32E-189 | 1.634525 | 1 | 0.519 | 2.83E-185 | 25 | *HMGB2* | Prolif. NRPCs (MKi67+) |
| NUSAP12 | 0 | 1.605206 | 1 | 0.193 | 0 | 25 | *NUSAP1* | Prolif. NRPCs (MKi67+) |
| HES64 | 4.88E-127 | 1.578596 | 0.987 | 0.593 | 1.05E-122 | 25 | *HES6* | Prolif. NRPCs (MKi67+) |
| HIST1H1D1 | 0 | 1.555434 | 0.872 | 0.148 | 0 | 25 | *HIST1H1D* | Prolif. NRPCs (MKi67+) |
| MKI672 | 0 | 1.541463 | 0.983 | 0.125 | 0 | 25 | *MKI67* | Prolif. NRPCs (MKi67+) |
| UBE2C2 | 0 | 1.410338 | 0.97 | 0.116 | 0 | 25 | *UBE2C* | Prolif. NRPCs (MKi67+) |
| GADD45A2 | 1.76E-146 | 1.373284 | 0.936 | 0.422 | 3.78E-142 | 25 | *GADD45A* | Prolif. NRPCs (MKi67+) |
| SMC42 | 3.56E-297 | 1.299647 | 0.983 | 0.21 | 7.64E-293 | 25 | *SMC4* | Prolif. NRPCs (MKi67+) |
| HMGN23 | 9.70E-166 | 1.281623 | 1 | 0.903 | 2.08E-161 | 25 | *HMGN2* | Prolif. NRPCs (MKi67+) |
| TUBA1B6 | 1.05E-158 | 1.279539 | 1 | 0.89 | 2.26E-154 | 25 | *TUBA1B* | Prolif. NRPCs (MKi67+) |
| UBE2S2 | 3.04E-173 | 1.24863 | 0.987 | 0.452 | 6.52E-169 | 25 | *UBE2S* | Prolif. NRPCs (MKi67+) |
| UBE2T3 | 6.10E-276 | 1.248435 | 0.987 | 0.237 | 1.31E-271 | 25 | *UBE2T* | Prolif. NRPCs (MKi67+) |
| CKS1B2 | 0 | 1.234589 | 0.946 | 0.181 | 0 | 25 | *CKS1B* | Prolif. NRPCs (MKi67+) |
| PTTG12 | 2.24E-260 | 1.182641 | 0.98 | 0.223 | 4.81E-256 | 25 | *PTTG1* | Prolif. NRPCs (MKi67+) |
| HIST1H1C1 | 2.28E-225 | 1.125772 | 0.848 | 0.191 | 4.90E-221 | 25 | *HIST1H1C* | Prolif. NRPCs (MKi67+) |
| CCDC343 | 1.80E-172 | 1.124628 | 0.953 | 0.356 | 3.85E-168 | 25 | *CCDC34* | Prolif. NRPCs (MKi67+) |
| H2AFZ3 | 3.01E-161 | 1.09256 | 1 | 0.924 | 6.46E-157 | 25 | *H2AFZ* | Prolif. NRPCs (MKi67+) |
| CDK12 | 0 | 1.063045 | 0.936 | 0.135 | 0 | 25 | *CDK1* | Prolif. NRPCs (MKi67+) |
| TPX22 | 0 | 1.047711 | 0.956 | 0.118 | 0 | 25 | *TPX2* | Prolif. NRPCs (MKi67+) |
| HMGB12 | 1.03E-152 | 1.007895 | 1 | 0.982 | 2.20E-148 | 25 | *HMGB1* | Prolif. NRPCs (MKi67+) |
| MAD2L12 | 5.14E-300 | 1.002998 | 0.929 | 0.17 | 1.10E-295 | 25 | *MAD2L1* | Prolif. NRPCs (MKi67+) |
| BIRC52 | 0 | 0.996159 | 0.929 | 0.092 | 0 | 25 | *BIRC5* | Prolif. NRPCs (MKi67+) |
| ASPM2 | 0 | 0.975119 | 0.869 | 0.089 | 0 | 25 | *ASPM* | Prolif. NRPCs (MKi67+) |
| CKS22 | 2.30E-173 | 0.951862 | 0.943 | 0.319 | 4.94E-169 | 25 | *CKS2* | Prolif. NRPCs (MKi67+) |
| TUBB4 | 7.17E-136 | 0.946731 | 1 | 0.911 | 1.54E-131 | 25 | *TUBB* | Prolif. NRPCs (MKi67+) |
| CENPU2 | 4.42E-269 | 0.941775 | 0.929 | 0.183 | 9.48E-265 | 25 | *CENPU* | Prolif. NRPCs (MKi67+) |
| PMAIP12 | 3.94E-159 | 0.926103 | 0.912 | 0.273 | 8.45E-155 | 25 | *PMAIP1* | Prolif. NRPCs (MKi67+) |
| TYMS5 | 1.53E-149 | 0.896525 | 0.96 | 0.337 | 3.29E-145 | 25 | *TYMS* | Prolif. NRPCs (MKi67+) |
| PBK2 | 0 | 0.895312 | 0.842 | 0.08 | 0 | 25 | *PBK* | Prolif. NRPCs (MKi67+) |
| TMPO3 | 3.05E-135 | 0.892383 | 0.946 | 0.437 | 6.53E-131 | 25 | *TMPO* | Prolif. NRPCs (MKi67+) |
| CDKN32 | 0 | 0.848438 | 0.805 | 0.111 | 0 | 25 | *CDKN3* | Prolif. NRPCs (MKi67+) |
| HIST1H1B1 | 0 | 0.847455 | 0.71 | 0.032 | 0 | 25 | *HIST1H1B* | Prolif. NRPCs (MKi67+) |
| PTMA1 | 8.69E-106 | 0.844758 | 1 | 1 | 1.86E-101 | 25 | *PTMA* | Prolif. NRPCs (MKi67+) |
| CALM21 | 1.85E-134 | 0.840873 | 1 | 0.965 | 3.96E-130 | 25 | *CALM2* | Prolif. NRPCs (MKi67+) |
| MYBL1 | 0 | 0.83657 | 0.785 | 0.092 | 0 | 25 | *MYBL1* | Prolif. NRPCs (MKi67+) |
| USP13 | 5.33E-138 | 0.833769 | 0.933 | 0.377 | 1.14E-133 | 25 | *USP1* | Prolif. NRPCs (MKi67+) |
| CKB4 | 6.22E-52 | 0.832328 | 0.997 | 0.952 | 1.33E-47 | 25 | *CKB* | Prolif. NRPCs (MKi67+) |
| GTSE12 | 0 | 0.8272 | 0.801 | 0.085 | 0 | 25 | *GTSE1* | Prolif. NRPCs (MKi67+) |
| TMSB15A3 | 5.32E-126 | 0.8112 | 0.96 | 0.422 | 1.14E-121 | 25 | *TMSB15A* | Prolif. NRPCs (MKi67+) |
| CCNB21 | 0 | 0.79771 | 0.778 | 0.085 | 0 | 25 | *CCNB2* | Prolif. NRPCs (MKi67+) |
| RAD51AP1 | 0 | 0.797276 | 0.805 | 0.11 | 0 | 25 | *RAD51AP1* | Prolif. NRPCs (MKi67+) |
| NEUROD14 | 1.17E-87 | 0.793402 | 0.788 | 0.302 | 2.50E-83 | 25 | *NEUROD1* | Prolif. NRPCs (MKi67+) |
| CKAP22 | 4.38E-188 | 0.788918 | 0.919 | 0.238 | 9.39E-184 | 25 | *CKAP2* | Prolif. NRPCs (MKi67+) |
| ATAD21 | 1.04E-209 | 0.78854 | 0.811 | 0.167 | 2.23E-205 | 25 | *ATAD2* | Prolif. NRPCs (MKi67+) |
| TUBB4B4 | 2.73E-94 | 0.785328 | 0.993 | 0.75 | 5.85E-90 | 25 | *TUBB4B* | Prolif. NRPCs (MKi67+) |
| H2AFX2 | 3.48E-191 | 0.782537 | 0.855 | 0.205 | 7.45E-187 | 25 | *H2AFX* | Prolif. NRPCs (MKi67+) |
| CALM32 | 1.73E-112 | 0.769863 | 0.956 | 0.493 | 3.71E-108 | 25 | *CALM3* | Prolif. NRPCs (MKi67+) |
| KIF20B2 | 3.00E-269 | 0.766043 | 0.771 | 0.115 | 6.43E-265 | 25 | *KIF20B* | Prolif. NRPCs (MKi67+) |
| KNL12 | 0 | 0.762752 | 0.744 | 0.066 | 0 | 25 | *KNL1* | Prolif. NRPCs (MKi67+) |
| PCLAF5 | 5.81E-177 | 0.761739 | 0.886 | 0.215 | 1.25E-172 | 25 | *PCLAF* | Prolif. NRPCs (MKi67+) |
| STMN13 | 2.64E-113 | 0.758888 | 1 | 0.953 | 5.65E-109 | 25 | *STMN1* | Prolif. NRPCs (MKi67+) |
| RAD211 | 5.16E-101 | 0.754938 | 0.956 | 0.526 | 1.11E-96 | 25 | *RAD21* | Prolif. NRPCs (MKi67+) |
| MIS18BP12 | 1.30E-228 | 0.736681 | 0.822 | 0.153 | 2.80E-224 | 25 | *MIS18BP1* | Prolif. NRPCs (MKi67+) |

| DEK3 | 8.31E-101 | 0.731348 | 0.997 | 0.777 | 1.78E-96 | 25 | *DEK* | Prolif. NRPCs (MKi67+) |
| --- | --- | --- | --- | --- | --- | --- | --- | --- |
| SPC251 | 0 | 0.728871 | 0.761 | 0.07 | 0 | 25 | *SPC25* | Prolif. NRPCs (MKi67+) |
| ANP32E2 | 9.49E-109 | 0.72633 | 0.943 | 0.464 | 2.04E-104 | 25 | *ANP32E* | Prolif. NRPCs (MKi67+) |
| AURKB2 | 0 | 0.724724 | 0.727 | 0.057 | 0 | 25 | *AURKB* | Prolif. NRPCs (MKi67+) |
| KIF112 | 0 | 0.717994 | 0.771 | 0.076 | 0 | 25 | *KIF11* | Prolif. NRPCs (MKi67+) |
| NUCKS12 | 5.11E-113 | 0.712712 | 1 | 0.906 | 1.10E-108 | 25 | *NUCKS1* | Prolif. NRPCs (MKi67+) |
| NUF22 | 0 | 0.707816 | 0.721 | 0.066 | 0 | 25 | *NUF2* | Prolif. NRPCs (MKi67+) |
| DLGAP52 | 0 | 0.706043 | 0.707 | 0.062 | 0 | 25 | *DLGAP5* | Prolif. NRPCs (MKi67+) |
| UBB2 | 1.90E-95 | 0.705277 | 0.99 | 0.926 | 4.08E-91 | 25 | *UBB* | Prolif. NRPCs (MKi67+) |
| H2AFV2 | 3.42E-101 | 0.702911 | 0.97 | 0.56 | 7.33E-97 | 25 | *H2AFV* | Prolif. NRPCs (MKi67+) |
| FBXO52 | 3.38E-196 | 0.701972 | 0.764 | 0.155 | 7.26E-192 | 25 | *FBXO5* | Prolif. NRPCs (MKi67+) |
| PPIA3 | 3.09E-105 | 0.701128 | 1 | 0.903 | 6.62E-101 | 25 | *PPIA* | Prolif. NRPCs (MKi67+) |
| SYNE22 | 4.83E-75 | 0.694643 | 0.923 | 0.558 | 1.04E-70 | 25 | *SYNE2* | Prolif. NRPCs (MKi67+) |
| SMC31 | 5.33E-82 | 0.6933 | 0.943 | 0.59 | 1.14E-77 | 25 | *SMC3* | Prolif. NRPCs (MKi67+) |
| HMGB31 | 1.46E-126 | 0.693251 | 0.835 | 0.282 | 3.14E-122 | 25 | *HMGB3* | Prolif. NRPCs (MKi67+) |
| ESCO21 | 0 | 0.686437 | 0.704 | 0.054 | 0 | 25 | *ESCO2* | Prolif. NRPCs (MKi67+) |
| DBI1 | 4.95E-92 | 0.677533 | 0.946 | 0.517 | 1.06E-87 | 25 | *DBI* | Prolif. NRPCs (MKi67+) |
| DTYMK2 | 4.44E-149 | 0.673616 | 0.886 | 0.269 | 9.53E-145 | 25 | *DTYMK* | Prolif. NRPCs (MKi67+) |
| CDKN2D1 | 1.53E-199 | 0.672124 | 0.724 | 0.134 | 3.28E-195 | 25 | *CDKN2D* | Prolif. NRPCs (MKi67+) |
| RRM13 | 4.26E-130 | 0.671919 | 0.825 | 0.257 | 9.14E-126 | 25 | *RRM1* | Prolif. NRPCs (MKi67+) |
| ORC61 | 5.16E-208 | 0.670779 | 0.751 | 0.14 | 1.11E-203 | 25 | *ORC6* | Prolif. NRPCs (MKi67+) |
| ZWINT1 | 1.54E-282 | 0.666348 | 0.768 | 0.109 | 3.30E-278 | 25 | *ZWINT* | Prolif. NRPCs (MKi67+) |
| HNRNPA0 | 1.84E-92 | 0.661816 | 0.973 | 0.651 | 3.94E-88 | 25 | *HNRNPA0* | Prolif. NRPCs (MKi67+) |
| NDC802 | 0 | 0.656672 | 0.71 | 0.066 | 0 | 25 | *NDC80* | Prolif. NRPCs (MKi67+) |
| HNRNPA2B | 3.05E-102 | 0.654455 | 1 | 0.946 | 6.55E-98 | 25 | *HNRNPA2B* | Prolif. NRPCs (MKi67+) |
| SMC22 | 1.83E-177 | 0.653898 | 0.815 | 0.187 | 3.93E-173 | 25 | *SMC2* | Prolif. NRPCs (MKi67+) |
| RTKN22 | 0 | 0.649308 | 0.721 | 0.072 | 0 | 25 | *RTKN2* | Prolif. NRPCs (MKi67+) |
| CKAP2L1 | 0 | 0.648552 | 0.707 | 0.053 | 0 | 25 | *CKAP2L* | Prolif. NRPCs (MKi67+) |
| KIFC12 | 0 | 0.646506 | 0.724 | 0.073 | 0 | 25 | *KIFC1* | Prolif. NRPCs (MKi67+) |
| KIF221 | 2.74E-133 | 0.641489 | 0.825 | 0.243 | 5.87E-129 | 25 | *KIF22* | Prolif. NRPCs (MKi67+) |
| LMNB12 | 1.61E-134 | 0.639799 | 0.778 | 0.215 | 3.45E-130 | 25 | *LMNB1* | Prolif. NRPCs (MKi67+) |
| ECT21 | 1.62E-182 | 0.636082 | 0.697 | 0.132 | 3.48E-178 | 25 | *ECT2* | Prolif. NRPCs (MKi67+) |
| CCNA22 | 0 | 0.635202 | 0.657 | 0.067 | 0 | 25 | *CCNA2* | Prolif. NRPCs (MKi67+) |
| BTG32 | 1.25E-80 | 0.631265 | 0.896 | 0.459 | 2.69E-76 | 25 | *BTG3* | Prolif. NRPCs (MKi67+) |
| SGO22 | 1.62E-300 | 0.627161 | 0.677 | 0.077 | 3.48E-296 | 25 | *SGO2* | Prolif. NRPCs (MKi67+) |
| CDCA32 | 0 | 0.616431 | 0.69 | 0.063 | 0 | 25 | *CDCA3* | Prolif. NRPCs (MKi67+) |
| C21orf582 | 1.53E-208 | 0.616069 | 0.721 | 0.126 | 3.28E-204 | 25 | *C21orf58* | Prolif. NRPCs (MKi67+) |
| SGO11 | 0 | 0.613072 | 0.66 | 0.062 | 0 | 25 | *SGO1* | Prolif. NRPCs (MKi67+) |
| MXD32 | 0 | 0.612022 | 0.704 | 0.065 | 0 | 25 | *MXD3* | Prolif. NRPCs (MKi67+) |
| ITGA61 | 1.46E-72 | 0.611991 | 0.552 | 0.178 | 3.12E-68 | 25 | *ITGA6* | Prolif. NRPCs (MKi67+) |
| CDCA51 | 0 | 0.610491 | 0.657 | 0.05 | 0 | 25 | *CDCA5* | Prolif. NRPCs (MKi67+) |
| RRM21 | 2.34E-276 | 0.610055 | 0.657 | 0.079 | 5.01E-272 | 25 | *RRM2* | Prolif. NRPCs (MKi67+) |
| INSM1 | 1.19E-137 | 0.606043 | 0.63 | 0.138 | 2.55E-133 | 25 | *INSM1* | Prolif. NRPCs (MKi67+) |
| ACYP11 | 3.97E-110 | 0.604018 | 0.791 | 0.261 | 8.52E-106 | 25 | *ACYP1* | Prolif. NRPCs (MKi67+) |
| NEK21 | 0 | 0.595585 | 0.599 | 0.057 | 0 | 25 | *NEK2* | Prolif. NRPCs (MKi67+) |
| GMNN2 | 3.85E-127 | 0.595373 | 0.818 | 0.241 | 8.26E-123 | 25 | *GMNN* | Prolif. NRPCs (MKi67+) |
| CENPE1 | 9.85E-252 | 0.595023 | 0.566 | 0.063 | 2.11E-247 | 25 | *CENPE* | Prolif. NRPCs (MKi67+) |
| NCAPG2 | 0 | 0.593146 | 0.643 | 0.067 | 0 | 25 | *NCAPG* | Prolif. NRPCs (MKi67+) |
| HNRNPAB2 | 6.93E-68 | 0.592964 | 0.906 | 0.542 | 1.49E-63 | 25 | *HNRNPAB* | Prolif. NRPCs (MKi67+) |
| NCAPD3 | 9.54E-263 | 0.592522 | 0.673 | 0.089 | 2.05E-258 | 25 | *NCAPD3* | Prolif. NRPCs (MKi67+) |
| PCM11 | 6.50E-75 | 0.587915 | 0.936 | 0.581 | 1.39E-70 | 25 | *PCM1* | Prolif. NRPCs (MKi67+) |
| SRSF3 | 1.22E-85 | 0.587532 | 0.993 | 0.849 | 2.61E-81 | 25 | *SRSF3* | Prolif. NRPCs (MKi67+) |
| KPNA21 | 2.02E-123 | 0.585803 | 0.734 | 0.198 | 4.34E-119 | 25 | *KPNA2* | Prolif. NRPCs (MKi67+) |
| ATAD5 | 2.30E-143 | 0.585296 | 0.697 | 0.161 | 4.93E-139 | 25 | *ATAD5* | Prolif. NRPCs (MKi67+) |
| PIMREG2 | 0 | 0.584502 | 0.633 | 0.059 | 0 | 25 | *PIMREG* | Prolif. NRPCs (MKi67+) |
| JPT13 | 2.20E-70 | 0.57808 | 0.899 | 0.502 | 4.71E-66 | 25 | *JPT1* | Prolif. NRPCs (MKi67+) |
| DYNLL11 | 2.39E-68 | 0.576299 | 0.99 | 0.869 | 5.13E-64 | 25 | *DYNLL1* | Prolif. NRPCs (MKi67+) |
| HIST1H1E | 6.02E-200 | 0.573192 | 0.579 | 0.085 | 1.29E-195 | 25 | *HIST1H1E* | Prolif. NRPCs (MKi67+) |
| BUB32 | 7.39E-101 | 0.571285 | 0.859 | 0.333 | 1.59E-96 | 25 | *BUB3* | Prolif. NRPCs (MKi67+) |
| CENPW1 | 6.82E-304 | 0.570587 | 0.63 | 0.067 | 1.46E-299 | 25 | *CENPW* | Prolif. NRPCs (MKi67+) |
| SRSF10 | 1.06E-76 | 0.569747 | 0.953 | 0.608 | 2.27E-72 | 25 | *SRSF10* | Prolif. NRPCs (MKi67+) |
| ASF1B2 | 1.08E-224 | 0.567413 | 0.68 | 0.101 | 2.31E-220 | 25 | *ASF1B* | Prolif. NRPCs (MKi67+) |
| HIST2H2AC | 0 | 0.566469 | 0.566 | 0.053 | 0 | 25 | *HIST2H2AC* | Prolif. NRPCs (MKi67+) |

| FAM111A1 | 4.11E-137 | 0.566164 | 0.64 | 0.144 | 8.81E-133 | 25 | *FAM111A* | Prolif. NRPCs (MKi67+) |
| --- | --- | --- | --- | --- | --- | --- | --- | --- |
| H2AFY2 | 7.17E-65 | 0.564538 | 0.946 | 0.625 | 1.54E-60 | 25 | *H2AFY* | Prolif. NRPCs (MKi67+) |
| H1FX2 | 1.36E-60 | 0.561597 | 0.966 | 0.723 | 2.91E-56 | 25 | *H1FX* | Prolif. NRPCs (MKi67+) |
| KIF151 | 0 | 0.560478 | 0.613 | 0.054 | 0 | 25 | *KIF15* | Prolif. NRPCs (MKi67+) |
| CRNDE1 | 7.01E-109 | 0.558225 | 0.781 | 0.247 | 1.50E-104 | 25 | *CRNDE* | Prolif. NRPCs (MKi67+) |
| H3F3A1 | 3.68E-110 | 0.553044 | 1 | 0.999 | 7.90E-106 | 25 | *H3F3A* | Prolif. NRPCs (MKi67+) |
| HJURP1 | 0 | 0.550233 | 0.609 | 0.049 | 0 | 25 | *HJURP* | Prolif. NRPCs (MKi67+) |
| CLSPN2 | 3.64E-131 | 0.548757 | 0.68 | 0.158 | 7.81E-127 | 25 | *CLSPN* | Prolif. NRPCs (MKi67+) |
| ARL6IP11 | 4.90E-50 | 0.54699 | 0.815 | 0.441 | 1.05E-45 | 25 | *ARL6IP1* | Prolif. NRPCs (MKi67+) |
| CDCA81 | 0 | 0.545592 | 0.63 | 0.054 | 0 | 25 | *CDCA8* | Prolif. NRPCs (MKi67+) |
| DHFR2 | 5.56E-110 | 0.545366 | 0.633 | 0.164 | 1.19E-105 | 25 | *DHFR* | Prolif. NRPCs (MKi67+) |
| NEAT11 | 5.39E-35 | 0.540373 | 0.98 | 0.92 | 1.16E-30 | 25 | *NEAT1* | Prolif. NRPCs (MKi67+) |
| KIF232 | 0 | 0.540268 | 0.62 | 0.06 | 0 | 25 | *KIF23* | Prolif. NRPCs (MKi67+) |
| CBX51 | 2.49E-69 | 0.539686 | 0.936 | 0.545 | 5.34E-65 | 25 | *CBX5* | Prolif. NRPCs (MKi67+) |
| SOX113 | 1.46E-53 | 0.538109 | 0.832 | 0.447 | 3.13E-49 | 25 | *SOX11* | Prolif. NRPCs (MKi67+) |
| ANP32B1 | 8.75E-67 | 0.536963 | 0.869 | 0.458 | 1.88E-62 | 25 | *ANP32B* | Prolif. NRPCs (MKi67+) |
| CENPN | 4.70E-270 | 0.536451 | 0.589 | 0.066 | 1.01E-265 | 25 | *CENPN* | Prolif. NRPCs (MKi67+) |
| IDH21 | 9.85E-98 | 0.535447 | 0.734 | 0.242 | 2.11E-93 | 25 | *IDH2* | Prolif. NRPCs (MKi67+) |
| CENPK1 | 1.45E-183 | 0.528651 | 0.717 | 0.134 | 3.11E-179 | 25 | *CENPK* | Prolif. NRPCs (MKi67+) |
| CENPH1 | 2.68E-117 | 0.528594 | 0.731 | 0.2 | 5.74E-113 | 25 | *CENPH* | Prolif. NRPCs (MKi67+) |
| RAN1 | 1.11E-70 | 0.527902 | 0.97 | 0.806 | 2.38E-66 | 25 | *RAN* | Prolif. NRPCs (MKi67+) |
| PRC11 | 7.91E-256 | 0.525061 | 0.603 | 0.071 | 1.70E-251 | 25 | *PRC1* | Prolif. NRPCs (MKi67+) |
| HMMR1 | 0 | 0.522475 | 0.566 | 0.049 | 0 | 25 | *HMMR* | Prolif. NRPCs (MKi67+) |
| GNAS | 9.74E-30 | 0.522385 | 0.926 | 0.775 | 2.09E-25 | 25 | *GNAS* | Prolif. NRPCs (MKi67+) |
| HNRNPA3 | 9.49E-64 | 0.521955 | 0.963 | 0.743 | 2.03E-59 | 25 | *HNRNPA3* | Prolif. NRPCs (MKi67+) |
| BRCA2 | 1.67E-245 | 0.521511 | 0.609 | 0.076 | 3.59E-241 | 25 | *BRCA2* | Prolif. NRPCs (MKi67+) |
| GADD45G3 | 1.09E-45 | 0.518201 | 0.724 | 0.354 | 2.33E-41 | 25 | *GADD45G* | Prolif. NRPCs (MKi67+) |
| NMU1 | 5.59E-189 | 0.51808 | 0.545 | 0.077 | 1.20E-184 | 25 | *NMU* | Prolif. NRPCs (MKi67+) |
| RBBP72 | 2.04E-68 | 0.516548 | 0.869 | 0.429 | 4.37E-64 | 25 | *RBBP7* | Prolif. NRPCs (MKi67+) |
| PFN21 | 4.67E-54 | 0.515912 | 0.785 | 0.42 | 1.00E-49 | 25 | *PFN2* | Prolif. NRPCs (MKi67+) |
| NUCB2 | 2.88E-60 | 0.515171 | 0.845 | 0.45 | 6.18E-56 | 25 | *NUCB2* | Prolif. NRPCs (MKi67+) |
| HP1BP31 | 1.23E-60 | 0.515164 | 0.902 | 0.523 | 2.65E-56 | 25 | *HP1BP3* | Prolif. NRPCs (MKi67+) |
| PCNA5 | 1.43E-70 | 0.515072 | 0.795 | 0.334 | 3.06E-66 | 25 | *PCNA* | Prolif. NRPCs (MKi67+) |
| TUBG1 | 1.19E-115 | 0.510898 | 0.684 | 0.182 | 2.54E-111 | 25 | *TUBG1* | Prolif. NRPCs (MKi67+) |
| NASP2 | 6.76E-60 | 0.510632 | 0.943 | 0.581 | 1.45E-55 | 25 | *NASP* | Prolif. NRPCs (MKi67+) |
| SMC1A1 | 5.56E-72 | 0.509956 | 0.761 | 0.323 | 1.19E-67 | 25 | *SMC1A* | Prolif. NRPCs (MKi67+) |
| RANBP14 | 4.44E-56 | 0.506051 | 0.933 | 0.609 | 9.52E-52 | 25 | *RANBP1* | Prolif. NRPCs (MKi67+) |
| PRR111 | 8.14E-296 | 0.504068 | 0.542 | 0.05 | 1.74E-291 | 25 | *PRR11* | Prolif. NRPCs (MKi67+) |
| SHD1 | 6.39E-115 | 0.501072 | 0.66 | 0.167 | 1.37E-110 | 25 | *SHD* | Prolif. NRPCs (MKi67+) |
| FOXM1 | 0 | 0.500727 | 0.606 | 0.043 | 0 | 25 | *FOXM1* | Prolif. NRPCs (MKi67+) |
| PTTG13 | 1.67E-228 | 1.577983 | 0.907 | 0.224 | 3.58E-224 | 26 | *PTTG1* | Proliferating cells (MKi67+) |
| HMGB24 | 3.79E-98 | 1.251007 | 0.918 | 0.52 | 8.12E-94 | 26 | *HMGB2* | Proliferating cells (MKi67+) |
| CENPF3 | 1.31E-266 | 1.235385 | 0.948 | 0.189 | 2.81E-262 | 26 | *CENPF* | Proliferating cells (MKi67+) |
| UBE2S3 | 1.56E-62 | 1.059461 | 0.766 | 0.454 | 3.34E-58 | 26 | *UBE2S* | Proliferating cells (MKi67+) |
| HSP90B11 | 4.48E-94 | 0.931566 | 0.935 | 0.69 | 9.60E-90 | 26 | *HSP90B1* | Proliferating cells (MKi67+) |
| BIRC53 | 1.84E-266 | 0.853496 | 0.715 | 0.095 | 3.95E-262 | 26 | *BIRC5* | Proliferating cells (MKi67+) |
| HMGN24 | 3.42E-95 | 0.822149 | 0.997 | 0.903 | 7.34E-91 | 26 | *HMGN2* | Proliferating cells (MKi67+) |
| MKI673 | 1.04E-211 | 0.800467 | 0.759 | 0.128 | 2.23E-207 | 26 | *MKI67* | Proliferating cells (MKi67+) |
| DYNLL12 | 1.43E-98 | 0.800122 | 0.99 | 0.87 | 3.08E-94 | 26 | *DYNLL1* | Proliferating cells (MKi67+) |
| CKB5 | 5.59E-81 | 0.759535 | 1 | 0.952 | 1.20E-76 | 26 | *CKB* | Proliferating cells (MKi67+) |
| LSM51 | 2.87E-85 | 0.750433 | 0.808 | 0.398 | 6.14E-81 | 26 | *LSM5* | Proliferating cells (MKi67+) |
| RAD212 | 2.73E-62 | 0.743617 | 0.849 | 0.528 | 5.85E-58 | 26 | *RAD21* | Proliferating cells (MKi67+) |
| CCNB22 | 3.51E-203 | 0.738013 | 0.601 | 0.087 | 7.52E-199 | 26 | *CCNB2* | Proliferating cells (MKi67+) |
| CCDC344 | 4.13E-77 | 0.736651 | 0.794 | 0.358 | 8.86E-73 | 26 | *CCDC34* | Proliferating cells (MKi67+) |
| CCNA11 | 0 | 0.718099 | 0.601 | 0.057 | 0 | 26 | *CCNA1* | Proliferating cells (MKi67+) |
| HMGB13 | 1.57E-106 | 0.711059 | 1 | 0.982 | 3.37E-102 | 26 | *HMGB1* | Proliferating cells (MKi67+) |
| NMU2 | 6.74E-253 | 0.701929 | 0.615 | 0.076 | 1.44E-248 | 26 | *NMU* | Proliferating cells (MKi67+) |
| H2AFZ4 | 1.04E-79 | 0.672253 | 0.99 | 0.924 | 2.23E-75 | 26 | *H2AFZ* | Proliferating cells (MKi67+) |
| TPX23 | 1.33E-134 | 0.665773 | 0.605 | 0.123 | 2.85E-130 | 26 | *TPX2* | Proliferating cells (MKi67+) |
| CALM33 | 4.11E-68 | 0.644878 | 0.849 | 0.494 | 8.81E-64 | 26 | *CALM3* | Proliferating cells (MKi67+) |
| HES65 | 1.31E-27 | 0.64314 | 0.852 | 0.594 | 2.80E-23 | 26 | *HES6* | Proliferating cells (MKi67+) |
| VIM6 | 1.13E-60 | 0.642087 | 0.993 | 0.855 | 2.43E-56 | 26 | *VIM* | Proliferating cells (MKi67+) |
| PTMS1 | 3.25E-69 | 0.641296 | 0.979 | 0.907 | 6.97E-65 | 26 | *PTMS* | Proliferating cells (MKi67+) |

| TOP2A3 | 1.27E-156 | 0.624458 | 0.811 | 0.188 | 2.73E-152 | 26 | *TOP2A* | Proliferating cells (MKi67+) |
| --- | --- | --- | --- | --- | --- | --- | --- | --- |
| HMGB32 | 8.21E-73 | 0.612586 | 0.704 | 0.283 | 1.76E-68 | 26 | *HMGB3* | Proliferating cells (MKi67+) |
| NUCKS13 | 3.03E-66 | 0.593112 | 0.986 | 0.907 | 6.50E-62 | 26 | *NUCKS1* | Proliferating cells (MKi67+) |
| CDKN33 | 1.16E-88 | 0.584155 | 0.488 | 0.115 | 2.48E-84 | 26 | *CDKN3* | Proliferating cells (MKi67+) |
| MARCKS2 | 7.35E-45 | 0.565646 | 0.966 | 0.898 | 1.58E-40 | 26 | *MARCKS* | Proliferating cells (MKi67+) |
| CCND11 | 4.89E-34 | 0.564976 | 0.883 | 0.647 | 1.05E-29 | 26 | *CCND1* | Proliferating cells (MKi67+) |
| ARL6IP12 | 7.09E-52 | 0.563417 | 0.78 | 0.442 | 1.52E-47 | 26 | *ARL6IP1* | Proliferating cells (MKi67+) |
| TAGLN22 | 6.09E-50 | 0.561662 | 0.821 | 0.508 | 1.31E-45 | 26 | *TAGLN2* | Proliferating cells (MKi67+) |
| HNRNPA2B | 6.83E-69 | 0.557644 | 0.993 | 0.946 | 1.47E-64 | 26 | *HNRNPA2B* | Proliferating cells (MKi67+) |
| CCNB11 | 3.28E-102 | 0.55475 | 0.44 | 0.085 | 7.04E-98 | 26 | *CCNB1* | Proliferating cells (MKi67+) |
| SMC43 | 2.53E-69 | 0.551276 | 0.625 | 0.214 | 5.42E-65 | 26 | *SMC4* | Proliferating cells (MKi67+) |
| CKS23 | 1.29E-52 | 0.548864 | 0.701 | 0.322 | 2.76E-48 | 26 | *CKS2* | Proliferating cells (MKi67+) |
| CYP26A15 | 1.10E-15 | 0.531844 | 0.742 | 0.556 | 2.37E-11 | 26 | *CYP26A1* | Proliferating cells (MKi67+) |
| TMSB15A4 | 1.27E-55 | 0.51897 | 0.814 | 0.424 | 2.72E-51 | 26 | *TMSB15A* | Proliferating cells (MKi67+) |
| ID31 | 1.02E-18 | 0.516732 | 0.533 | 0.322 | 2.20E-14 | 26 | *ID3* | Proliferating cells (MKi67+) |
| CDC201 | 2.34E-111 | 0.50798 | 0.368 | 0.056 | 5.02E-107 | 26 | *CDC20* | Proliferating cells (MKi67+) |
| ANP32E3 | 9.82E-39 | 0.501561 | 0.742 | 0.467 | 2.11E-34 | 26 | *ANP32E* | Proliferating cells (MKi67+) |
| FTL | 3.31E-158 | 1.710258 | 1 | 0.998 | 7.11E-154 | 27 | *FTL* | astrocytes |
| GDF15 | 0 | 1.687309 | 0.551 | 0.03 | 0 | 27 | *GDF15* | astrocytes |
| FTH11 | 1.16E-148 | 1.45023 | 1 | 0.999 | 2.49E-144 | 27 |  | astrocytes |
| ATF5 | 0 | 1.351523 | 0.763 | 0.105 | 0 | 27 | *ATF5* | astrocytes |
| SQSTM11 | 4.39E-106 | 1.191769 | 0.852 | 0.388 | 9.41E-102 | 27 | *SQSTM1* | astrocytes |
| MT2A | 2.15E-12 | 1.186586 | 0.265 | 0.132 | 4.60E-08 | 27 | *MT2A* | astrocytes |
| NUPR1 | 0 | 1.087041 | 0.548 | 0.041 | 0 | 27 | *NUPR1* | astrocytes |
| SDCBP | 2.76E-93 | 1.069394 | 0.795 | 0.365 | 5.91E-89 | 27 | *SDCBP* | astrocytes |
| CDKN1A | 3.48E-205 | 0.998273 | 0.76 | 0.149 | 7.47E-201 | 27 | *CDKN1A* | astrocytes |
| SLC3A2 | 2.37E-101 | 0.991295 | 0.841 | 0.376 | 5.09E-97 | 27 | *SLC3A2* | astrocytes |
| HSPA5 | 3.83E-60 | 0.975887 | 0.876 | 0.635 | 8.21E-56 | 27 | *HSPA5* | astrocytes |
| EIF1 | 1.29E-132 | 0.973177 | 1 | 0.993 | 2.76E-128 | 27 | *EIF1* | astrocytes |
| S100A61 | 2.63E-52 | 0.967987 | 0.841 | 0.521 | 5.63E-48 | 27 | *S100A6* | astrocytes |
| HERPUD1 | 7.23E-108 | 0.907743 | 0.823 | 0.323 | 1.55E-103 | 27 | *HERPUD1* | astrocytes |
| NDUFA4L2 | 3.59E-223 | 0.88872 | 0.477 | 0.051 | 7.71E-219 | 27 | *NDUFA4L2* | astrocytes |
| HSPB12 | 1.46E-85 | 0.828204 | 0.739 | 0.29 | 3.12E-81 | 27 | *HSPB1* | astrocytes |
| LINC02154 | 5.79E-283 | 0.814879 | 0.254 | 0.01 | 1.24E-278 | 27 | *LINC02154* | astrocytes |
| TRIB3 | 0 | 0.814313 | 0.597 | 0.054 | 0 | 27 | *TRIB3* | astrocytes |
| RPS27L | 1.05E-78 | 0.802908 | 0.887 | 0.576 | 2.25E-74 | 27 | *RPS27L* | astrocytes |
| TKT2 | 1.30E-60 | 0.766951 | 0.834 | 0.498 | 2.80E-56 | 27 | *TKT* | astrocytes |
| DDIT31 | 6.33E-72 | 0.763716 | 0.746 | 0.327 | 1.36E-67 | 27 | *DDIT3* | astrocytes |
| PHLDA3 | 7.52E-212 | 0.761684 | 0.731 | 0.128 | 1.61E-207 | 27 | *PHLDA3* | astrocytes |
| TPT11 | 6.39E-64 | 0.756264 | 1 | 1 | 1.37E-59 | 27 | *TPT1* | astrocytes |
| BNIP3 | 3.50E-57 | 0.753981 | 0.876 | 0.582 | 7.51E-53 | 27 | *BNIP3* | astrocytes |
| MT1X1 | 0.001534 | 0.752454 | 0.219 | 0.153 | 1 | 27 | *MT1X* | astrocytes |
| MAP1LC3B | 6.97E-62 | 0.746948 | 0.845 | 0.507 | 1.49E-57 | 27 | *MAP1LC3B* | astrocytes |
| ARF42 | 1.37E-64 | 0.741708 | 0.859 | 0.502 | 2.93E-60 | 27 | *ARF4* | astrocytes |
| HSPA9 | 1.13E-73 | 0.687262 | 0.813 | 0.415 | 2.42E-69 | 27 | *HSPA9* | astrocytes |
| TCEA1 | 2.13E-64 | 0.655782 | 0.876 | 0.569 | 4.58E-60 | 27 | *TCEA1* | astrocytes |
| UCHL11 | 1.36E-45 | 0.650159 | 0.816 | 0.536 | 2.92E-41 | 27 | *UCHL1* | astrocytes |
| LDHA | 1.26E-41 | 0.639006 | 0.922 | 0.804 | 2.70E-37 | 27 | *LDHA* | astrocytes |
| HSP90AB1 | 5.70E-77 | 0.624033 | 1 | 0.985 | 1.22E-72 | 27 | *HSP90AB1* | astrocytes |
| LGALS31 | 1.01E-84 | 0.623019 | 0.466 | 0.108 | 2.16E-80 | 27 | *LGALS3* | astrocytes |
| SLC2A11 | 5.46E-52 | 0.621238 | 0.753 | 0.405 | 1.17E-47 | 27 | *SLC2A1* | astrocytes |
| PURPL | 9.70E-277 | 0.612119 | 0.36 | 0.023 | 2.08E-272 | 27 | *PURPL* | astrocytes |
| ATF3 | 1.89E-109 | 0.609978 | 0.449 | 0.084 | 4.05E-105 | 27 | *ATF3* | astrocytes |
| PHPT1 | 2.37E-45 | 0.608822 | 0.873 | 0.628 | 5.07E-41 | 27 | *PHPT1* | astrocytes |
| KDELR2 | 1.02E-52 | 0.601118 | 0.746 | 0.413 | 2.18E-48 | 27 | *KDELR2* | astrocytes |
| S100A11 | 8.36E-138 | 0.585706 | 0.445 | 0.067 | 1.79E-133 | 27 | *S100A11* | astrocytes |
| SEC61G1 | 9.01E-44 | 0.582877 | 0.883 | 0.731 | 1.93E-39 | 27 | *SEC61G* | astrocytes |
| ANXA2 | 9.22E-71 | 0.580873 | 0.541 | 0.172 | 1.98E-66 | 27 | *ANXA2* | astrocytes |
| ANXA1 | 3.23E-146 | 0.578553 | 0.283 | 0.026 | 6.93E-142 | 27 | *ANXA1* | astrocytes |
| P4HA1 | 3.77E-58 | 0.57425 | 0.739 | 0.35 | 8.08E-54 | 27 | *P4HA1* | astrocytes |
| ZFAS1 | 6.77E-39 | 0.569962 | 0.958 | 0.896 | 1.45E-34 | 27 | *ZFAS1* | astrocytes |
| SNHG8 | 2.06E-53 | 0.552323 | 0.739 | 0.381 | 4.42E-49 | 27 | *SNHG8* | astrocytes |
| ANXA5 | 5.41E-67 | 0.552081 | 0.664 | 0.255 | 1.16E-62 | 27 | *ANXA5* | astrocytes |

| 2.21E-59 | 0.550865 | 0.763 | 0.38 | 4.73E-55 | 27 *SERP1* | astrocytes |
| --- | --- | --- | --- | --- | --- | --- |
| 3.15E-28 | 0.549527 | 0.18 | 0.044 | 6.75E-24 | 27 *CRYAB* | astrocytes |
| 9.85E-58 | 0.544657 | 0.473 | 0.148 | 2.11E-53 | 27 *TIMP3* | astrocytes |
| 4.79E-53 | 0.544591 | 0.618 | 0.26 | 1.03E-48 | 27 *LITAF* | astrocytes |
| 9.41E-38 | 0.543331 | 0.661 | 0.364 | 2.02E-33 | 27 *GPI* | astrocytes |
| 5.28E-41 | 0.543289 | 0.869 | 0.646 | 1.13E-36 | 27 *TRMT112* | astrocytes |
| 8.88E-58 | 0.542036 | 0.473 | 0.149 | 1.90E-53 | 27 *MFAP4* | astrocytes |
| 6.63E-54 | 0.54128 | 0.678 | 0.323 | 1.42E-49 | 27 *YBX3* | astrocytes |
| 2.47E-46 | 0.538257 | 0.731 | 0.391 | 5.30E-42 | 27 *FAM162A* | astrocytes |
| 1.00E-58 | 0.531026 | 0.703 | 0.324 | 2.15E-54 | 27 *SNHG7* | astrocytes |
| 2.50E-51 | 0.521053 | 0.862 | 0.564 | 5.35E-47 | 27 *ARF1* | astrocytes |
| 1.61E-58 | 0.519941 | 0.512 | 0.172 | 3.44E-54 | 27 *DDIT4* | astrocytes |
| 1.78E-46 | 0.51143 | 0.873 | 0.589 | 3.83E-42 | 27 *LAPTM4A* | astrocytes |
| 2.47E-32 | 0.504668 | 0.813 | 0.599 | 5.29E-28 | 27 *KRT10* | astrocytes |
| 88E-191 | 2.165331 | 0.996 | 0.4 | 8.33E-187 | 28 *PDE6H* | Cone precursors |
| 08E-207 | 1.781626 | 0.965 | 0.286 | 1.95E-202 | 28 *GUCA1A* | Cone precursors |
| 24E-182 | 1.575995 | 0.984 | 0.324 | 6.94E-178 | 28 *RRAD* | Cone precursors |
| 92E-215 | 1.487586 | 1 | 0.282 | 8.41E-211 | 28 *AIPL1* | Cone precursors |
| 16E-154 | 1.443357 | 1 | 0.623 | 4.64E-150 | 28 *AKAP9* | Cone precursors |
| 94E-148 | 1.44082 | 1 | 0.497 | 6.30E-144 | 28 *UNC119* | Cone precursors |
| 56E-305 | 1.433293 | 0.98 | 0.178 | 2.05E-300 | 28 *MPP4* | Cone precursors |
| 69E-247 | 1.404971 | 0.98 | 0.216 | 3.62E-243 | 28 *GNGT2* | Cone precursors |
| 71E-170 | 1.358997 | 1 | 0.435 | 1.23E-165 | 28 *MAP2* | Cone precursors |
| 19E-174 | 1.349531 | 1 | 0.358 | 8.98E-170 | 28 *GNB3* | Cone precursors |
| 49E-142 | 1.317975 | 0.961 | 0.349 | 7.47E-138 | 28 *RCVRN* | Cone precursors |
| 11E-143 | 1.296919 | 1 | 0.717 | 2.39E-139 | 28 *GUK1* | Cone precursors |
| 47E-238 | 1.264636 | 0.992 | 0.24 | 3.16E-234 | 28 *TULP1* | Cone precursors |
| 82E-101 | 1.255708 | 0.977 | 0.578 | 1.89E-96 | 28 *CRABP2* | Cone precursors |
| 97E-223 | 1.212349 | 0.773 | 0.127 | 4.21E-219 | 28 *ARR3* | Cone precursors |
| 30E-150 | 1.20993 | 0.973 | 0.419 | 1.35E-145 | 28 *PPP1CC* | Cone precursors |
| 09E-129 | 1.175729 | 1 | 0.457 | 2.34E-125 | 28 *PDC* | Cone precursors |
| 24E-210 | 1.131163 | 0.973 | 0.243 | 6.95E-206 | 28 *DPYSL3* | Cone precursors |
| 04E-304 | 1.111275 | 0.91 | 0.139 | 1.51E-299 | 28 *MYL4* | Cone precursors |
| 34E-239 | 1.092783 | 0.973 | 0.205 | 2.00E-234 | 28 *IMPG2* | Cone precursors |
| 0 | 1.083652 | 0.793 | 0.065 | 0 | 28 *GUCA1C* | Cone precursors |
| 14E-231 | 1.067253 | 0.941 | 0.2 | 4.60E-227 | 28 *FSTL5* | Cone precursors |
| 78E-203 | 1.063635 | 0.895 | 0.202 | 3.82E-199 | 28 *HRASLS* | Cone precursors |
| 98E-128 | 1.055163 | 0.988 | 0.468 | 1.50E-123 | 28 *NME1* | Cone precursors |
| 0 | 1.04767 | 0.906 | 0.125 | 0 | 28 *RS1* | Cone precursors |
| 98E-127 | 1.039983 | 0.992 | 0.407 | 1.50E-122 | 28 *SCG3* | Cone precursors |
| 56E-153 | 1.031192 | 0.977 | 0.331 | 7.63E-149 | 28 *Sep-04* | Cone precursors |
| 14E-131 | 1.026909 | 0.996 | 0.558 | 6.74E-127 | 28 *CPE* | Cone precursors |
| 90E-291 | 1.021828 | 0.902 | 0.141 | 1.48E-286 | 28 *KCNV2* | Cone precursors |
| 84E-134 | 1.007341 | 1 | 0.488 | 3.95E-130 | 28 *ENO2* | Cone precursors |
| 53E-202 | 0.994594 | 0.938 | 0.228 | 5.42E-198 | 28 *PLEKHB1* | Cone precursors |
| 14E-173 | 0.99341 | 0.984 | 0.279 | 2.44E-169 | 28 *CRX* | Cone precursors |
| 01E-274 | 0.981841 | 0.875 | 0.138 | 2.16E-270 | 28 *LMOD1* | Cone precursors |
| 40E-236 | 0.967069 | 0.887 | 0.161 | 3.00E-232 | 28 *CPLX4* | Cone precursors |
| 84E-114 | 0.945769 | 0.594 | 0.134 | 6.09E-110 | 28 *CLSTN2* | Cone precursors |
| 37E-231 | 0.944008 | 0.922 | 0.188 | 1.15E-226 | 28 *CC2D2A* | Cone precursors |
| 78E-143 | 0.941334 | 0.918 | 0.275 | 1.67E-138 | 28 *TUBA4A* | Cone precursors |
| 20E-149 | 0.937007 | 0.98 | 0.315 | 6.87E-145 | 28 *FAM57B* | Cone precursors |
| 34E-240 | 0.928207 | 0.891 | 0.159 | 5.02E-236 | 28 *PEX5L* | Cone precursors |
| 71E-187 | 0.910964 | 0.941 | 0.244 | 1.22E-182 | 28 *NEDD4L* | Cone precursors |
| 55E-115 | 0.908247 | 0.938 | 0.427 | 7.61E-111 | 28 *DHRS7* | Cone precursors |
| 66E-165 | 0.898989 | 0.887 | 0.223 | 3.55E-161 | 28 *ALDOC* | Cone precursors |
| 14E-170 | 0.889199 | 0.949 | 0.264 | 1.75E-165 | 28 *AGAP1* | Cone precursors |
| 86E-122 | 0.873765 | 0.961 | 0.452 | 3.98E-118 | 28 *KIF2A* | Cone precursors |
| 01E-134 | 0.857686 | 0.973 | 0.367 | 6.46E-130 | 28 *SLC38A1* | Cone precursors |
| 71E-143 | 0.853462 | 0.93 | 0.259 | 1.22E-138 | 28 *SLC38A5* | Cone precursors |
| 93E-142 | 0.843032 | 0.949 | 0.286 | 6.29E-138 | 28 *SYP* | Cone precursors |
| 1.09E-96 | 0.84147 | 1 | 0.863 | 2.34E-92 | 28 *MAP1B* | Cone precursors |
| 2.09E-79 | 0.839574 | 0.867 | 0.413 | 4.48E-75 | 28 *DST* | Cone precursors |

| SERP1 |
| --- |
| CRYAB |
| TIMP31 |
| LITAF |
| GPI |
| TRMT112 |
| MFAP41 |
| YBX3 |
| FAM162A |
| SNHG7 |
| ARF11 |
| DDIT4 |
| LAPTM4A |
| KRT10 |
| PDE6H3 3. |
| GUCA1A3 9. |
| RRAD4 3. |
| AIPL14 3. |
| AKAP95 2. |
| UNC1194 2. |
| MPP43 9. |
| GNGT23 1. |
| MAP25 5. |
| GNB34 4. |
| RCVRN3 3. |
| GUK14 1. |
| TULP13 1. |
| CRABP22 8. |
| ARR32 1. |
| PPP1CC1 6. |
| PDC5 1. |
| DPYSL33 3. |
| MYL41 7. |
| IMPG24 9. |
| GUCA1C |
| FSTL53 2. |
| HRASLS3 1. |
| NME11 6. |
| RS11 |
| SCG37 6. |
| Sep-44 3. |
| CPE5 3. |
| KCNV22 6. |
| ENO24 1. |
| PLEKHB12 2. |
| CRX4 1. |
| LMOD11 1. |
| CPLX42 1. |
| CLSTN22 2. |
| CC2D2A1 5. |
| TUBA4A3 7. |
| FAM57B5 3. |
| PEX5L3 2. |
| NEDD4L1 5. |
| DHRS71 3. |
| ALDOC3 1. |
| AGAP12 8. |
| KIF2A1 1. |
| SLC38A13 3. |
| SLC38A54 5. |
| SYP5 2. |
| MAP1B1 |
| DST1 |

| MLXIP3 | 4.90E-155 | 0.83606 | 0.82 | 0.202 | 1.05E-150 | 28 | *MLXIP* | Cone precursors |
| --- | --- | --- | --- | --- | --- | --- | --- | --- |
| PRDX13 | 4.10E-81 | 0.820616 | 0.988 | 0.815 | 8.78E-77 | 28 | *PRDX1* | Cone precursors |
| FAM107A2 | 1.79E-293 | 0.814078 | 0.836 | 0.113 | 3.83E-289 | 28 | *FAM107A* | Cone precursors |
| RIMS2 | 6.69E-123 | 0.808958 | 0.766 | 0.217 | 1.43E-118 | 28 | *RIMS2* | Cone precursors |
| ARL6IP51 | 2.69E-106 | 0.808555 | 0.949 | 0.495 | 5.76E-102 | 28 | *ARL6IP5* | Cone precursors |
| CNTNAP22 | 6.53E-163 | 0.807859 | 0.855 | 0.207 | 1.40E-158 | 28 | *CNTNAP2* | Cone precursors |
| MAP1LC3A | 3.99E-124 | 0.807559 | 0.941 | 0.337 | 8.54E-120 | 28 | *MAP1LC3A* | Cone precursors |
| PRCD2 | 7.10E-201 | 0.796764 | 0.875 | 0.178 | 1.52E-196 | 28 | *PRCD* | Cone precursors |
| PARP1 | 7.90E-97 | 0.791559 | 0.984 | 0.56 | 1.69E-92 | 28 | *PARP1* | Cone precursors |
| SLC17A72 | 9.90E-189 | 0.789179 | 0.844 | 0.176 | 2.12E-184 | 28 | *SLC17A7* | Cone precursors |
| SLC4A7 | 4.61E-107 | 0.785236 | 0.91 | 0.378 | 9.88E-103 | 28 | *SLC4A7* | Cone precursors |
| PROM11 | 6.03E-164 | 0.778437 | 0.852 | 0.21 | 1.29E-159 | 28 | *PROM1* | Cone precursors |
| TPD521 | 3.04E-115 | 0.751478 | 0.887 | 0.321 | 6.51E-111 | 28 | *TPD52* | Cone precursors |
| STX33 | 1.41E-157 | 0.750065 | 0.895 | 0.229 | 3.02E-153 | 28 | *STX3* | Cone precursors |
| COTL11 | 9.37E-131 | 0.74963 | 0.871 | 0.274 | 2.01E-126 | 28 | *COTL1* | Cone precursors |
| SYT12 | 1.80E-95 | 0.740561 | 0.996 | 0.635 | 3.86E-91 | 28 | *SYT1* | Cone precursors |
| LINC00599 | 9.66E-131 | 0.739929 | 0.875 | 0.237 | 2.07E-126 | 28 | *LINC00599* | Cone precursors |
| RP13 | 1.01E-149 | 0.736607 | 0.77 | 0.162 | 2.16E-145 | 28 | *RP1* | Cone precursors |
| PCBP45 | 1.51E-104 | 0.733628 | 0.984 | 0.391 | 3.23E-100 | 28 | *PCBP4* | Cone precursors |
| ARHGAP32 | 3.08E-159 | 0.718579 | 0.836 | 0.199 | 6.61E-155 | 28 | *ARHGAP32* | Cone precursors |
| SEZ6L22 | 7.89E-119 | 0.716805 | 0.902 | 0.295 | 1.69E-114 | 28 | *SEZ6L2* | Cone precursors |
| VAMP22 | 2.18E-94 | 0.712323 | 0.992 | 0.697 | 4.68E-90 | 28 | *VAMP2* | Cone precursors |
| GUCA1B | 0 | 0.702891 | 0.66 | 0.048 | 0 | 28 | *GUCA1B* | Cone precursors |
| NTM1 | 2.16E-172 | 0.695469 | 0.871 | 0.194 | 4.64E-168 | 28 | *NTM* | Cone precursors |
| KCNB11 | 2.29E-170 | 0.693956 | 0.797 | 0.163 | 4.91E-166 | 28 | *KCNB1* | Cone precursors |
| PTPN133 | 8.94E-119 | 0.692155 | 0.812 | 0.235 | 1.92E-114 | 28 | *PTPN13* | Cone precursors |
| UGCG1 | 2.30E-108 | 0.689967 | 0.828 | 0.271 | 4.93E-104 | 28 | *UGCG* | Cone precursors |
| CAMK1D | 2.33E-200 | 0.689725 | 0.789 | 0.143 | 4.99E-196 | 28 | *CAMK1D* | Cone precursors |
| MAP4 | 3.71E-100 | 0.669713 | 0.926 | 0.392 | 7.95E-96 | 28 | *MAP4* | Cone precursors |
| NRN12 | 5.61E-129 | 0.668349 | 0.746 | 0.175 | 1.20E-124 | 28 | *NRN1* | Cone precursors |
| RBP42 | 5.55E-235 | 0.658929 | 0.691 | 0.09 | 1.19E-230 | 28 | *RBP4* | Cone precursors |
| ANKRD33B | 6.83E-144 | 0.655124 | 0.824 | 0.196 | 1.47E-139 | 28 | *ANKRD33B* | Cone precursors |
| MEGF9 | 4.20E-134 | 0.653537 | 0.785 | 0.201 | 9.01E-130 | 28 | *MEGF9* | Cone precursors |
| LAPTM4B2 | 3.53E-84 | 0.652319 | 0.941 | 0.493 | 7.56E-80 | 28 | *LAPTM4B* | Cone precursors |
| PPP2R2B | 2.30E-251 | 0.649812 | 0.746 | 0.1 | 4.93E-247 | 28 | *PPP2R2B* | Cone precursors |
| ANK2 | 2.27E-114 | 0.647877 | 0.809 | 0.239 | 4.86E-110 | 28 | *ANK2* | Cone precursors |
| CALCOCO2 | 1.22E-102 | 0.645288 | 0.875 | 0.316 | 2.61E-98 | 28 | *CALCOCO2* | Cone precursors |
| VXN3 | 5.74E-83 | 0.644842 | 0.617 | 0.17 | 1.23E-78 | 28 | *VXN* | Cone precursors |
| PPP4R4 | 8.43E-232 | 0.641199 | 0.633 | 0.079 | 1.81E-227 | 28 | *PPP4R4* | Cone precursors |
| ABHD14A1 | 7.00E-115 | 0.640375 | 0.844 | 0.262 | 1.50E-110 | 28 | *ABHD14A* | Cone precursors |
| EPB412 | 5.25E-81 | 0.632092 | 0.926 | 0.418 | 1.13E-76 | 28 | *EPB41* | Cone precursors |
| KIF1B1 | 1.77E-85 | 0.631704 | 0.887 | 0.391 | 3.79E-81 | 28 | *KIF1B* | Cone precursors |
| TMX42 | 8.33E-70 | 0.630254 | 0.871 | 0.416 | 1.79E-65 | 28 | *TMX4* | Cone precursors |
| RD3 | 3.00E-229 | 0.628599 | 0.746 | 0.109 | 6.44E-225 | 28 | *RD3* | Cone precursors |
| AC110716. | 5.38E-274 | 0.626767 | 0.676 | 0.075 | 1.15E-269 | 28 | *AC110716.* | Cone precursors |
| CADM21 | 1.29E-105 | 0.626132 | 0.871 | 0.286 | 2.76E-101 | 28 | *CADM2* | Cone precursors |
| RAI141 | 8.66E-89 | 0.624776 | 0.824 | 0.307 | 1.86E-84 | 28 | *RAI14* | Cone precursors |
| IFI27L21 | 5.02E-72 | 0.619199 | 0.863 | 0.425 | 1.08E-67 | 28 | *IFI27L2* | Cone precursors |
| NLK1 | 3.03E-126 | 0.6176 | 0.82 | 0.215 | 6.49E-122 | 28 | *NLK* | Cone precursors |
| INA | 3.47E-152 | 0.615007 | 0.785 | 0.17 | 7.44E-148 | 28 | *INA* | Cone precursors |
| PKIB2 | 3.00E-116 | 0.614672 | 0.77 | 0.204 | 6.44E-112 | 28 | *PKIB* | Cone precursors |
| SPTAN1 | 9.78E-94 | 0.613048 | 0.859 | 0.319 | 2.10E-89 | 28 | *SPTAN1* | Cone precursors |
| SNAP25 | 1.50E-136 | 0.610088 | 0.805 | 0.198 | 3.22E-132 | 28 | *SNAP25* | Cone precursors |
| SLC6A6 | 1.59E-186 | 0.609023 | 0.758 | 0.135 | 3.40E-182 | 28 | *SLC6A6* | Cone precursors |
| OLFM12 | 1.40E-115 | 0.601315 | 0.629 | 0.141 | 3.01E-111 | 28 | *OLFM1* | Cone precursors |
| CADPS2 | 5.28E-104 | 0.601303 | 0.809 | 0.232 | 1.13E-99 | 28 | *CADPS* | Cone precursors |
| GOLGB1 | 1.43E-53 | 0.59977 | 0.887 | 0.498 | 3.06E-49 | 28 | *GOLGB1* | Cone precursors |
| MCF2L2 | 1.27E-234 | 0.593202 | 0.699 | 0.093 | 2.71E-230 | 28 | *MCF2L2* | Cone precursors |
| PKM3 | 1.66E-64 | 0.588907 | 0.988 | 0.828 | 3.56E-60 | 28 | *PKM* | Cone precursors |
| PTMS2 | 1.15E-54 | 0.588071 | 0.992 | 0.907 | 2.46E-50 | 28 | *PTMS* | Cone precursors |
| FAM19A4 | 2.80E-186 | 0.581807 | 0.695 | 0.112 | 5.99E-182 | 28 | *FAM19A4* | Cone precursors |
| COX171 | 3.45E-66 | 0.579746 | 0.871 | 0.424 | 7.41E-62 | 28 | *COX17* | Cone precursors |
| UBAP1L | 1.04E-197 | 0.578366 | 0.684 | 0.104 | 2.23E-193 | 28 | *UBAP1L* | Cone precursors |

| RTBDN | 9.47E-249 | 0.578049 | 0.633 | 0.071 | 2.03E-244 | 28 | *RTBDN* | Cone precursors |
| --- | --- | --- | --- | --- | --- | --- | --- | --- |
| SLC1A22 | 2.12E-124 | 0.575524 | 0.766 | 0.187 | 4.55E-120 | 28 | *SLC1A2* | Cone precursors |
| AANAT4 | 6.72E-98 | 0.575095 | 0.738 | 0.204 | 1.44E-93 | 28 | *AANAT* | Cone precursors |
| KIF21A | 1.21E-77 | 0.574783 | 0.75 | 0.276 | 2.60E-73 | 28 | *KIF21A* | Cone precursors |
| SLC2A111 | 1.19E-130 | 0.573903 | 0.68 | 0.148 | 2.56E-126 | 28 | *SLC2A11* | Cone precursors |
| CHCHD10 | 9.23E-98 | 0.573707 | 0.812 | 0.265 | 1.98E-93 | 28 | *CHCHD10* | Cone precursors |
| NEUROG11 | 1.62E-100 | 0.572998 | 0.668 | 0.165 | 3.47E-96 | 28 | *NEUROG1* | Cone precursors |
| SCAMP1 | 2.03E-86 | 0.572547 | 0.781 | 0.278 | 4.35E-82 | 28 | *SCAMP1* | Cone precursors |
| PLD4 | 9.12E-207 | 0.572274 | 0.543 | 0.063 | 1.96E-202 | 28 | *PLD4* | Cone precursors |
| MT-ND21 | 6.63E-65 | 0.570755 | 1 | 0.978 | 1.42E-60 | 28 | *MT-ND2* | Cone precursors |
| IGSF211 | 4.30E-165 | 0.567467 | 0.738 | 0.139 | 9.23E-161 | 28 | *IGSF21* | Cone precursors |
| CDHR1 | 8.01E-196 | 0.563404 | 0.684 | 0.104 | 1.72E-191 | 28 | *CDHR1* | Cone precursors |
| RGS9 | 8.94E-128 | 0.560206 | 0.723 | 0.169 | 1.92E-123 | 28 | *RGS9* | Cone precursors |
| AC023905. | 0 | 0.559172 | 0.547 | 0.036 | 0 | 28 | *AC023905.* | Cone precursors |
| CLCN4 | 8.37E-143 | 0.558711 | 0.66 | 0.129 | 1.80E-138 | 28 | *CLCN4* | Cone precursors |
| ZNF385A | 2.94E-93 | 0.558551 | 0.777 | 0.248 | 6.30E-89 | 28 | *ZNF385A* | Cone precursors |
| ATP5F1A | 4.46E-67 | 0.557775 | 0.957 | 0.616 | 9.56E-63 | 28 | *ATP5F1A* | Cone precursors |
| STXBP1 | 4.53E-76 | 0.557317 | 0.754 | 0.283 | 9.72E-72 | 28 | *STXBP1* | Cone precursors |
| CLCN31 | 4.62E-69 | 0.556242 | 0.895 | 0.444 | 9.90E-65 | 28 | *CLCN3* | Cone precursors |
| SOX71 | 4.58E-194 | 0.555819 | 0.621 | 0.086 | 9.82E-190 | 28 | *SOX7* | Cone precursors |
| FAM213A1 | 3.19E-61 | 0.554887 | 0.871 | 0.486 | 6.84E-57 | 28 | *FAM213A* | Cone precursors |
| VAX2 | 3.19E-147 | 0.553961 | 0.715 | 0.145 | 6.83E-143 | 28 | *VAX2* | Cone precursors |
| RYBP1 | 1.77E-70 | 0.553599 | 0.727 | 0.266 | 3.79E-66 | 28 | *RYBP* | Cone precursors |
| TTC31 | 1.55E-57 | 0.550385 | 0.988 | 0.853 | 3.32E-53 | 28 | *TTC3* | Cone precursors |
| PIK3R13 | 2.32E-43 | 0.549828 | 0.859 | 0.501 | 4.98E-39 | 28 | *PIK3R1* | Cone precursors |
| PDE4DIP | 6.97E-113 | 0.54876 | 0.699 | 0.172 | 1.49E-108 | 28 | *PDE4DIP* | Cone precursors |
| SLC24A2 | 0 | 0.548189 | 0.551 | 0.038 | 0 | 28 | *SLC24A2* | Cone precursors |
| GNAT2 | 2.79E-241 | 0.547907 | 0.609 | 0.069 | 5.98E-237 | 28 | *GNAT2* | Cone precursors |
| IMPDH1 | 3.49E-152 | 0.546115 | 0.688 | 0.13 | 7.48E-148 | 28 | *IMPDH1* | Cone precursors |
| RXRG2 | 1.67E-114 | 0.544752 | 0.793 | 0.207 | 3.58E-110 | 28 | *RXRG* | Cone precursors |
| ZNF3261 | 3.52E-68 | 0.542973 | 0.891 | 0.414 | 7.55E-64 | 28 | *ZNF326* | Cone precursors |
| DHRS11 | 5.43E-157 | 0.54033 | 0.648 | 0.116 | 1.16E-152 | 28 | *DHRS11* | Cone precursors |
| EYS | 2.33E-176 | 0.536249 | 0.648 | 0.102 | 4.99E-172 | 28 | *EYS* | Cone precursors |
| ATP1A3 | 4.62E-124 | 0.53553 | 0.719 | 0.166 | 9.90E-120 | 28 | *ATP1A3* | Cone precursors |
| PTP4A32 | 2.89E-62 | 0.53274 | 0.855 | 0.392 | 6.20E-58 | 28 | *PTP4A3* | Cone precursors |
| ARHGAP42 | 1.98E-123 | 0.532272 | 0.668 | 0.147 | 4.25E-119 | 28 | *ARHGAP42* | Cone precursors |
| KCNQ1OT1 | 3.02E-28 | 0.53226 | 0.891 | 0.688 | 6.49E-24 | 28 | *KCNQ1OT1* | Cone precursors |
| SLC12A2 | 2.48E-73 | 0.531832 | 0.715 | 0.252 | 5.32E-69 | 28 | *SLC12A2* | Cone precursors |
| KCNB2 | 7.66E-214 | 0.528985 | 0.633 | 0.082 | 1.64E-209 | 28 | *KCNB2* | Cone precursors |
| PPFIA2 | 2.33E-106 | 0.528054 | 0.703 | 0.18 | 4.99E-102 | 28 | *PPFIA2* | Cone precursors |
| COBLL12 | 1.10E-97 | 0.526825 | 0.742 | 0.207 | 2.36E-93 | 28 | *COBLL1* | Cone precursors |
| TLN2 | 4.76E-196 | 0.525358 | 0.672 | 0.1 | 1.02E-191 | 28 | *TLN2* | Cone precursors |
| GNB51 | 2.98E-65 | 0.52369 | 0.754 | 0.301 | 6.40E-61 | 28 | *GNB5* | Cone precursors |
| ACSL6 | 4.10E-158 | 0.523571 | 0.652 | 0.114 | 8.79E-154 | 28 | *ACSL6* | Cone precursors |
| CA2 | 9.40E-70 | 0.52305 | 0.57 | 0.167 | 2.02E-65 | 28 | *CA2* | Cone precursors |
| UQCC22 | 8.68E-55 | 0.52117 | 0.879 | 0.496 | 1.86E-50 | 28 | *UQCC2* | Cone precursors |
| AHI11 | 2.22E-54 | 0.516524 | 0.812 | 0.407 | 4.77E-50 | 28 | *AHI1* | Cone precursors |
| LBH | 2.66E-62 | 0.516472 | 0.703 | 0.279 | 5.69E-58 | 28 | *LBH* | Cone precursors |
| KCNH6 | 3.23E-148 | 0.515642 | 0.633 | 0.111 | 6.92E-144 | 28 | *KCNH6* | Cone precursors |
| YBX11 | 4.40E-74 | 0.512908 | 0.996 | 0.981 | 9.44E-70 | 28 | *YBX1* | Cone precursors |
| GJD2 | 1.27E-155 | 0.510637 | 0.629 | 0.107 | 2.73E-151 | 28 | *GJD2* | Cone precursors |
| INSR1 | 2.07E-74 | 0.507219 | 0.711 | 0.244 | 4.44E-70 | 28 | *INSR* | Cone precursors |
| EDIL3 | 1.14E-115 | 0.505055 | 0.621 | 0.134 | 2.45E-111 | 28 | *EDIL3* | Cone precursors |
| MIR7-3HG | 3.88E-80 | 0.501255 | 0.613 | 0.167 | 8.31E-76 | 28 | *MIR7-3HG* | Cone precursors |
| NSF | 2.81E-111 | 0.500474 | 0.656 | 0.156 | 6.02E-107 | 28 | *NSF* | Cone precursors |
| IGFBP53 | 3.31E-19 | 0.506976 | 0.74 | 0.478 | 7.09E-15 | 29 | *IGFBP5* | Astrocytes |
| STMN22 | 6.56E-225 | 2.278824 | 1 | 0.229 | 1.41E-220 | 30 | *STMN2* | Horizontal cells |
| ONECUT2 | 0 | 1.793976 | 0.851 | 0.055 | 0 | 30 | *ONECUT2* | Horizontal cells |
| SOX44 | 2.71E-114 | 1.624218 | 1 | 0.862 | 5.82E-110 | 30 | *SOX4* | Horizontal cells |
| ONECUT1 | 0 | 1.376344 | 0.761 | 0.03 | 0 | 30 | *ONECUT1* | Horizontal cells |
| CELF42 | 5.62E-177 | 1.250667 | 0.955 | 0.239 | 1.21E-172 | 30 | *CELF4* | Horizontal cells |
| PROX1 | 2.68E-149 | 1.243258 | 0.657 | 0.113 | 5.75E-145 | 30 | *PROX1* | Horizontal cells |
| DCX2 | 1.39E-201 | 1.230146 | 0.881 | 0.161 | 2.97E-197 | 30 | *DCX* | Horizontal cells |

| TUBB2B2 | 2.04E-110 | 1.198291 | 1 | 0.711 | 4.37E-106 | 30 | *TUBB2B* | Horizontal cells |
| --- | --- | --- | --- | --- | --- | --- | --- | --- |
| RTN11 | 0 | 1.191059 | 0.905 | 0.096 | 0 | 30 | *RTN1* | Horizontal cells |
| STMN42 | 1.53E-204 | 1.185257 | 0.896 | 0.162 | 3.28E-200 | 30 | *STMN4* | Horizontal cells |
| TUBB2A2 | 1.22E-149 | 1.133859 | 0.94 | 0.271 | 2.61E-145 | 30 | *TUBB2A* | Horizontal cells |
| TUBA1A3 | 1.51E-92 | 1.108656 | 1 | 0.939 | 3.24E-88 | 30 | *TUBA1A* | Horizontal cells |
| PAX61 | 2.83E-87 | 1.092204 | 0.925 | 0.5 | 6.07E-83 | 30 | *PAX6* | Horizontal cells |
| BASP13 | 2.58E-101 | 1.06438 | 1 | 0.671 | 5.54E-97 | 30 | *BASP1* | Horizontal cells |
| JPT14 | 6.40E-105 | 1.048923 | 0.98 | 0.503 | 1.37E-100 | 30 | *JPT1* | Horizontal cells |
| TMSB102 | 5.54E-94 | 1.035438 | 1 | 0.966 | 1.19E-89 | 30 | *TMSB10* | Horizontal cells |
| GAP431 | 4.80E-91 | 1.029454 | 0.577 | 0.125 | 1.03E-86 | 30 | *GAP43* | Horizontal cells |
| TNR | 0 | 1.020372 | 0.597 | 0.01 | 0 | 30 | *TNR* | Horizontal cells |
| CXXC5 | 3.07E-95 | 1.018639 | 0.955 | 0.497 | 6.57E-91 | 30 | *CXXC5* | Horizontal cells |
| NSG21 | 0 | 0.995973 | 0.811 | 0.068 | 0 | 30 | *NSG2* | Horizontal cells |
| NSG11 | 0 | 0.986287 | 0.776 | 0.048 | 0 | 30 | *NSG1* | Horizontal cells |
| CD242 | 5.20E-119 | 0.9448 | 0.96 | 0.322 | 1.12E-114 | 30 | *CD24* | Horizontal cells |
| KIF5C1 | 3.96E-101 | 0.892922 | 0.871 | 0.302 | 8.50E-97 | 30 | *KIF5C* | Horizontal cells |
| GRIA41 | 8.65E-156 | 0.892273 | 0.786 | 0.148 | 1.85E-151 | 30 | *GRIA4* | Horizontal cells |
| GRIA21 | 0 | 0.87292 | 0.721 | 0.053 | 0 | 30 | *GRIA2* | Horizontal cells |
| ZEB2 | 2.41E-55 | 0.865782 | 0.731 | 0.336 | 5.16E-51 | 30 | *ZEB2* | Horizontal cells |
| MLLT112 | 7.61E-84 | 0.862958 | 0.97 | 0.52 | 1.63E-79 | 30 | *MLLT11* | Horizontal cells |
| ELAVL31 | 6.52E-172 | 0.838206 | 0.791 | 0.138 | 1.40E-167 | 30 | *ELAVL3* | Horizontal cells |
| TLE4 | 1.40E-79 | 0.831745 | 0.637 | 0.177 | 3.00E-75 | 30 | *TLE4* | Horizontal cells |
| MAPT | 3.29E-167 | 0.821839 | 0.741 | 0.125 | 7.06E-163 | 30 | *MAPT* | Horizontal cells |
| INA1 | 2.02E-146 | 0.811439 | 0.821 | 0.172 | 4.32E-142 | 30 | *INA* | Horizontal cells |
| CRMP11 | 6.51E-119 | 0.809671 | 0.816 | 0.21 | 1.40E-114 | 30 | *CRMP1* | Horizontal cells |
| PCSK1N1 | 3.56E-88 | 0.808696 | 0.881 | 0.337 | 7.64E-84 | 30 | *PCSK1N* | Horizontal cells |
| RORB2 | 9.39E-57 | 0.808021 | 0.93 | 0.627 | 2.01E-52 | 30 | *RORB* | Horizontal cells |
| XIST2 | 5.61E-29 | 0.798229 | 0.687 | 0.414 | 1.20E-24 | 30 | *XIST* | Horizontal cells |
| ARL4C | 1.23E-60 | 0.795432 | 0.846 | 0.393 | 2.63E-56 | 30 | *ARL4C* | Horizontal cells |
| TAGLN3 | 1.12E-113 | 0.772435 | 0.816 | 0.214 | 2.40E-109 | 30 | *TAGLN3* | Horizontal cells |
| FNBP1L1 | 5.63E-76 | 0.769778 | 0.871 | 0.374 | 1.21E-71 | 30 | *FNBP1L* | Horizontal cells |
| NREP1 | 6.13E-67 | 0.759618 | 0.97 | 0.604 | 1.31E-62 | 30 | *NREP* | Horizontal cells |
| SNCA | 3.73E-86 | 0.750831 | 0.607 | 0.147 | 7.99E-82 | 30 | *SNCA* | Horizontal cells |
| SOSTDC1 | 4.66E-229 | 0.747728 | 0.438 | 0.03 | 9.99E-225 | 30 | *SOSTDC1* | Horizontal cells |
| PKIA | 1.26E-87 | 0.745711 | 0.831 | 0.292 | 2.70E-83 | 30 | *PKIA* | Horizontal cells |
| SYT4 | 2.94E-89 | 0.733123 | 0.567 | 0.12 | 6.30E-85 | 30 | *SYT4* | Horizontal cells |
| APBB2 | 1.01E-113 | 0.721286 | 0.622 | 0.122 | 2.16E-109 | 30 | *APBB2* | Horizontal cells |
| KIDINS220 | 6.40E-64 | 0.720391 | 0.821 | 0.363 | 1.37E-59 | 30 | *KIDINS220* | Horizontal cells |
| VGF | 0 | 0.690689 | 0.473 | 0.014 | 0 | 30 | *VGF* | Horizontal cells |
| C4orf48 | 1.70E-59 | 0.681742 | 0.891 | 0.491 | 3.63E-55 | 30 | *C4orf48* | Horizontal cells |
| CNTNAP23 | 1.14E-49 | 0.677645 | 0.607 | 0.211 | 2.45E-45 | 30 | *CNTNAP2* | Horizontal cells |
| ANK3 | 1.16E-65 | 0.671901 | 0.741 | 0.263 | 2.48E-61 | 30 | *ANK3* | Horizontal cells |
| SNCG | 0 | 0.668293 | 0.567 | 0.021 | 0 | 30 | *SNCG* | Horizontal cells |
| PCDH92 | 2.57E-40 | 0.658517 | 0.692 | 0.308 | 5.50E-36 | 30 | *PCDH9* | Horizontal cells |
| RBFOX2 | 3.14E-150 | 0.653814 | 0.746 | 0.136 | 6.73E-146 | 30 | *RBFOX2* | Horizontal cells |
| MYT1L | 0 | 0.65344 | 0.567 | 0.02 | 0 | 30 | *MYT1L* | Horizontal cells |
| ONECUT3 | 0 | 0.64813 | 0.502 | 0.003 | 0 | 30 | *ONECUT3* | Horizontal cells |
| RUNX1T11 | 8.89E-107 | 0.64082 | 0.587 | 0.11 | 1.91E-102 | 30 | *RUNX1T1* | Horizontal cells |
| GNG31 | 2.04E-172 | 0.63999 | 0.607 | 0.077 | 4.36E-168 | 30 | *GNG3* | Horizontal cells |
| CCNI | 2.27E-54 | 0.63992 | 0.99 | 0.928 | 4.86E-50 | 30 | *CCNI* | Horizontal cells |
| CEP170 | 1.12E-72 | 0.638811 | 0.831 | 0.318 | 2.40E-68 | 30 | *CEP170* | Horizontal cells |
| DPYSL21 | 2.04E-48 | 0.631941 | 0.811 | 0.422 | 4.37E-44 | 30 | *DPYSL2* | Horizontal cells |
| PPP1R1A | 4.41E-181 | 0.630576 | 0.627 | 0.079 | 9.45E-177 | 30 | *PPP1R1A* | Horizontal cells |
| TTC32 | 8.28E-56 | 0.630122 | 0.98 | 0.854 | 1.78E-51 | 30 | *TTC3* | Horizontal cells |
| MARCKS3 | 3.88E-50 | 0.610042 | 0.99 | 0.898 | 8.33E-46 | 30 | *MARCKS* | Horizontal cells |
| MAP1B2 | 6.86E-31 | 0.598895 | 0.975 | 0.863 | 1.47E-26 | 30 | *MAP1B* | Horizontal cells |
| TFAP2A1 | 1.17E-163 | 0.595927 | 0.393 | 0.033 | 2.52E-159 | 30 | *TFAP2A* | Horizontal cells |
| MARCKSL1 | 4.01E-58 | 0.594092 | 0.99 | 0.907 | 8.60E-54 | 30 | *MARCKSL1* | Horizontal cells |
| TERF2IP | 1.09E-48 | 0.586829 | 0.91 | 0.552 | 2.34E-44 | 30 | *TERF2IP* | Horizontal cells |
| DYNC1H1 | 6.24E-41 | 0.585596 | 0.786 | 0.45 | 1.34E-36 | 30 | *DYNC1H1* | Horizontal cells |
| TTC9B | 0 | 0.579089 | 0.552 | 0.024 | 0 | 30 | *TTC9B* | Horizontal cells |
| UCHL12 | 6.71E-25 | 0.578561 | 0.796 | 0.537 | 1.44E-20 | 30 | *UCHL1* | Horizontal cells |
| NNAT2 | 3.30E-45 | 0.570675 | 0.831 | 0.394 | 7.08E-41 | 30 | *NNAT* | Horizontal cells |

| 6.84E-40 | 0.56794 | 0.448 | 0.139 | 1.47E-35 | 30 *RND3* | Horizontal cells |
| --- | --- | --- | --- | --- | --- | --- |
| 1.20E-41 | 0.566701 | 0.876 | 0.542 | 2.57E-37 | 30 *DAAM1* | Horizontal cells |
| 79E-177 | 0.562807 | 0.552 | 0.062 | 2.10E-172 | 30 *HSPA12A* | Horizontal cells |
| 1.07E-79 | 0.561984 | 0.592 | 0.141 | 2.29E-75 | 30 *SCG5* | Horizontal cells |
| 1.20E-37 | 0.559403 | 0.856 | 0.526 | 2.58E-33 | 30 *YWHAB* | Horizontal cells |
| 9.90E-63 | 0.556865 | 0.756 | 0.277 | 2.12E-58 | 30 *APC* | Horizontal cells |
| 2.80E-26 | 0.555029 | 0.751 | 0.449 | 6.01E-22 | 30 *SOX11* | Horizontal cells |
| 6.18E-45 | 0.55279 | 0.164 | 0.021 | 1.33E-40 | 30 *PRPH* | Horizontal cells |
| 5.46E-66 | 0.548313 | 0.736 | 0.25 | 1.17E-61 | 30 *KIF3A* | Horizontal cells |
| 52E-113 | 0.54808 | 0.597 | 0.106 | 1.18E-108 | 30 *NAV1* | Horizontal cells |
| 0 | 0.545063 | 0.502 | 0.018 | 0 | 30 *SCRT2* | Horizontal cells |
| 5.05E-40 | 0.537765 | 0.896 | 0.572 | 1.08E-35 | 30 *BEX2* | Horizontal cells |
| 1.90E-37 | 0.536298 | 0.806 | 0.478 | 4.07E-33 | 30 *NDFIP1* | Horizontal cells |
| 3.65E-49 | 0.534213 | 0.751 | 0.347 | 7.82E-45 | 30 *YWHAG* | Horizontal cells |
| 2.28E-79 | 0.533182 | 0.582 | 0.138 | 4.88E-75 | 30 *JAKMIP2* | Horizontal cells |
| 4.49E-37 | 0.531028 | 0.582 | 0.237 | 9.63E-33 | 30 *ZFHX4* | Horizontal cells |
| 4.84E-89 | 0.52751 | 0.552 | 0.113 | 1.04E-84 | 30 *LY6H* | Horizontal cells |
| 05E-203 | 0.520794 | 0.458 | 0.037 | 4.40E-199 | 30 *CNTN4* | Horizontal cells |
| 2.35E-80 | 0.519113 | 0.562 | 0.128 | 5.04E-76 | 30 *ATCAY* | Horizontal cells |
| 4.48E-30 | 0.518171 | 0.801 | 0.466 | 9.61E-26 | 30 *TPM3* | Horizontal cells |
| 8.37E-91 | 0.518021 | 0.572 | 0.12 | 1.79E-86 | 30 *NDRG4* | Horizontal cells |
| 0 | 0.514702 | 0.433 | 0.007 | 0 | 30 *CPNE4* | Horizontal cells |
| 2.06E-52 | 0.513988 | 0.398 | 0.093 | 4.42E-48 | 30 *RELN* | Horizontal cells |
| 8.52E-64 | 0.512865 | 0.607 | 0.177 | 1.83E-59 | 30 *ZBTB38* | Horizontal cells |
| 2.06E-22 | 0.508103 | 0.751 | 0.505 | 4.42E-18 | 30 *SIX3* | Horizontal cells |
| 2.40E-30 | 0.50511 | 0.453 | 0.167 | 5.14E-26 | 30 *ID4* | Horizontal cells |
| 2.30E-77 | 0.504143 | 0.572 | 0.135 | 4.93E-73 | 30 *SBK1* | Horizontal cells |
| 4.15E-39 | 0.503605 | 0.562 | 0.216 | 8.90E-35 | 30 *VEGFA* | Horizontal cells |
| 1.69E-61 | 0.502203 | 0.632 | 0.196 | 3.61E-57 | 30 *CLASP2* | Horizontal cells |
| 2.79E-57 | 0.501832 | 0.602 | 0.185 | 5.99E-53 | 30 *AL391650.* | Horizontal cells |
| 2.60E-38 | 0.500209 | 0.935 | 0.724 | 5.58E-34 | 30 *YWHAQ* | Horizontal cells |
| 0 | 5.01417 | 0.979 | 0.076 | 0 | 31 *MGP* | Fibroblasts |
| 0 | 3.522981 | 0.995 | 0.029 | 0 | 31 *COL3A1* | Fibroblasts |
| 0 | 3.244074 | 1 | 0.04 | 0 | 31 *COL1A1* | Fibroblasts |
| 41E-169 | 2.837134 | 0.995 | 0.358 | 1.16E-164 | 31 *COL1A2* | Fibroblasts |
| 0 | 2.545783 | 0.995 | 0.044 | 0 | 31 *LGALS1* | Fibroblasts |
| 0 | 2.37823 | 0.948 | 0.009 | 0 | 31 *LUM* | Fibroblasts |
| 0 | 2.209478 | 0.964 | 0.005 | 0 | 31 *DCN* | Fibroblasts |
| 0 | 2.053552 | 0.995 | 0.014 | 0 | 31 *TGFBI* | Fibroblasts |
| 47E-303 | 1.855265 | 0.897 | 0.112 | 3.16E-299 | 31 *CTSC* | Fibroblasts |
| 0 | 1.732492 | 0.902 | 0.065 | 0 | 31 *IGFBP6* | Fibroblasts |
| 21E-138 | 1.721071 | 0.479 | 0.058 | 9.03E-134 | 31 *CCL2* | Fibroblasts |
| 0 | 1.715233 | 0.897 | 0.051 | 0 | 31 *PLAT* | Fibroblasts |
| 08E-125 | 1.672495 | 0.979 | 0.376 | 2.32E-121 | 31 *IFITM3* | Fibroblasts |
| 45E-158 | 1.659405 | 0.985 | 0.305 | 5.26E-154 | 31 *SPARC* | Fibroblasts |
| 0 | 1.569646 | 0.943 | 0.065 | 0 | 31 *S100A11* | Fibroblasts |
| 38E-272 | 1.548514 | 0.912 | 0.129 | 1.37E-267 | 31 *LY6E* | Fibroblasts |
| 1.16E-83 | 1.531993 | 0.866 | 0.374 | 2.48E-79 | 31 *TIMP1* | Fibroblasts |
| 36E-107 | 1.446943 | 1 | 0.616 | 5.07E-103 | 31 *B2M* | Fibroblasts |
| 0 | 1.43767 | 0.82 | 0.056 | 0 | 31 *LXN* | Fibroblasts |
| 0 | 1.434227 | 0.644 | 0.04 | 0 | 31 *FN1* | Fibroblasts |
| 05E-105 | 1.40945 | 0.381 | 0.047 | 8.67E-101 | 31 *TAGLN* | Fibroblasts |
| 43E-189 | 1.407125 | 0.613 | 0.072 | 3.07E-185 | 31 *RARRES1* | Fibroblasts |
| 92E-129 | 1.351765 | 0.691 | 0.143 | 4.11E-125 | 31 *IFI6* | Fibroblasts |
| 24E-274 | 1.35156 | 0.861 | 0.11 | 9.10E-270 | 31 *IGFBP4* | Fibroblasts |
| 0 | 1.349895 | 0.737 | 0.021 | 0 | 31 *ELN* | Fibroblasts |
| 89E-293 | 1.262678 | 0.84 | 0.093 | 4.05E-289 | 31 *BST2* | Fibroblasts |
| 1.55E-60 | 1.238158 | 0.876 | 0.453 | 3.32E-56 | 31 *PTN* | Fibroblasts |
| 01E-276 | 1.229693 | 0.876 | 0.11 | 2.17E-272 | 31 *TPM2* | Fibroblasts |
| 79E-117 | 1.221861 | 0.582 | 0.101 | 1.24E-112 | 31 *IER3* | Fibroblasts |
| 1.06E-85 | 1.207801 | 0.577 | 0.133 | 2.27E-81 | 31 *VCAN* | Fibroblasts |
| 94E-265 | 1.179096 | 0.897 | 0.125 | 8.46E-261 | 31 *PCOLCE* | Fibroblasts |
| 0 | 1.125617 | 0.784 | 0.006 | 0 | 31 *BGN* | Fibroblasts |

| RND31 |
| --- |
| DAAM1 |
| HSPA12A 9. |
| SCG51 |
| YWHAB |
| APC |
| SOX114 |
| PRPH |
| KIF3A |
| NAV1 5. |
| SCRT2 |
| BEX2 |
| NDFIP1 |
| YWHAG |
| JAKMIP2 |
| ZFHX41 |
| LY6H |
| CNTN4 2. |
| ATCAY |
| TPM3 |
| NDRG4 |
| CPNE4 |
| RELN |
| ZBTB38 |
| SIX3 |
| ID4 |
| SBK1 |
| VEGFA |
| CLASP2 |
| AL391650. |
| YWHAQ |
| MGP |
| COL3A1 |
| COL1A1 |
| COL1A21 5. |
| LGALS1 |
| LUM |
| DCN |
| TGFBI |
| CTSC 1. |
| IGFBP61 |
| CCL21 4. |
| PLAT |
| IFITM33 1. |
| SPARC1 2. |
| S100A111 |
| LY6E 6. |
| TIMP1 |
| B2M1 2. |
| LXN |
| FN1 |
| TAGLN 4. |
| RARRES1 1. |
| IFI6 1. |
| IGFBP4 4. |
| ELN |
| BST21 1. |
| PTN3 |
| TPM2 1. |
| IER3 5. |
| VCAN1 |
| PCOLCE 3. |
| BGN |

| SERPINH1 | 1.66E-269 | 1.124549 | 0.923 | 0.13 | 3.55E-265 | 31 | *SERPINH1* | Fibroblasts |
| --- | --- | --- | --- | --- | --- | --- | --- | --- |
| CEBPD | 3.51E-239 | 1.122298 | 0.68 | 0.072 | 7.54E-235 | 31 | *CEBPD* | Fibroblasts |
| GDF151 | 9.70E-188 | 1.114386 | 0.423 | 0.033 | 2.08E-183 | 31 | *GDF15* | Fibroblasts |
| MT2A1 | 5.02E-35 | 1.1023 | 0.428 | 0.131 | 1.08E-30 | 31 | *MT2A* | Fibroblasts |
| NUPR11 | 0 | 1.080498 | 0.794 | 0.041 | 0 | 31 | *NUPR1* | Fibroblasts |
| COL5A2 | 0 | 1.075944 | 0.706 | 0.053 | 0 | 31 | *COL5A2* | Fibroblasts |
| CRABP23 | 2.07E-74 | 1.03835 | 0.974 | 0.579 | 4.45E-70 | 31 | *CRABP2* | Fibroblasts |
| FBLN1 | 1.12E-94 | 1.017339 | 0.799 | 0.256 | 2.41E-90 | 31 | *FBLN1* | Fibroblasts |
| HLA-B | 2.64E-139 | 0.989153 | 0.866 | 0.214 | 5.66E-135 | 31 | *HLA-B* | Fibroblasts |
| ID32 | 6.08E-69 | 0.980587 | 0.83 | 0.321 | 1.30E-64 | 31 | *ID3* | Fibroblasts |
| IGFBP7 | 2.37E-152 | 0.959651 | 0.577 | 0.077 | 5.07E-148 | 31 | *IGFBP7* | Fibroblasts |
| THBS2 | 0 | 0.950962 | 0.773 | 0.005 | 0 | 31 | *THBS2* | Fibroblasts |
| ANXA21 | 1.17E-143 | 0.950869 | 0.809 | 0.172 | 2.50E-139 | 31 | *ANXA2* | Fibroblasts |
| RRBP1 | 4.55E-142 | 0.938634 | 0.825 | 0.189 | 9.75E-138 | 31 | *RRBP1* | Fibroblasts |
| TBX3 | 0 | 0.93541 | 0.727 | 0.055 | 0 | 31 | *TBX3* | Fibroblasts |
| ID11 | 8.63E-58 | 0.93454 | 0.639 | 0.21 | 1.85E-53 | 31 | *ID1* | Fibroblasts |
| CALD1 | 4.60E-84 | 0.931608 | 0.948 | 0.479 | 9.86E-80 | 31 | *CALD1* | Fibroblasts |
| CTGF | 2.21E-78 | 0.928793 | 0.247 | 0.026 | 4.74E-74 | 31 | *CTGF* | Fibroblasts |
| PRRX1 | 0 | 0.928606 | 0.686 | 0.002 | 0 | 31 | *PRRX1* | Fibroblasts |
| SELENOM1 | 3.22E-119 | 0.921335 | 0.881 | 0.261 | 6.91E-115 | 31 | *SELENOM* | Fibroblasts |
| AIF1 | 5.56E-218 | 0.915662 | 0.552 | 0.05 | 1.19E-213 | 31 | *AIF1* | Fibroblasts |
| POSTN | 0 | 0.910537 | 0.459 | 0.002 | 0 | 31 | *POSTN* | Fibroblasts |
| AKAP12 | 2.38E-24 | 0.900866 | 0.603 | 0.328 | 5.09E-20 | 31 | *AKAP12* | Fibroblasts |
| JUNB1 | 3.03E-49 | 0.897487 | 0.918 | 0.522 | 6.49E-45 | 31 | *JUNB* | Fibroblasts |
| ANXA51 | 9.53E-120 | 0.89591 | 0.866 | 0.255 | 2.04E-115 | 31 | *ANXA5* | Fibroblasts |
| FTL1 | 2.93E-80 | 0.894146 | 1 | 0.998 | 6.29E-76 | 31 | *FTL* | Fibroblasts |
| LAPTM4A1 | 5.68E-89 | 0.892764 | 0.979 | 0.589 | 1.22E-84 | 31 | *LAPTM4A* | Fibroblasts |
| TMSB4X | 3.66E-76 | 0.889464 | 1 | 0.97 | 7.85E-72 | 31 | *TMSB4X* | Fibroblasts |
| ACTA2 | 1.62E-31 | 0.889092 | 0.247 | 0.056 | 3.47E-27 | 31 | *ACTA2* | Fibroblasts |
| PPIB | 3.74E-83 | 0.879931 | 0.969 | 0.686 | 8.02E-79 | 31 | *PPIB* | Fibroblasts |
| MFAP42 | 1.03E-141 | 0.872491 | 0.768 | 0.148 | 2.21E-137 | 31 | *MFAP4* | Fibroblasts |
| COL6A2 | 1.26E-133 | 0.844376 | 0.784 | 0.174 | 2.71E-129 | 31 | *COL6A2* | Fibroblasts |
| KDELR21 | 7.18E-87 | 0.837748 | 0.938 | 0.413 | 1.54E-82 | 31 | *KDELR2* | Fibroblasts |
| TGM2 | 0 | 0.834837 | 0.603 | 0.014 | 0 | 31 | *TGM2* | Fibroblasts |
| ATF51 | 6.84E-108 | 0.8291 | 0.588 | 0.109 | 1.47E-103 | 31 | *ATF5* | Fibroblasts |
| MFAP21 | 1.21E-78 | 0.815881 | 0.851 | 0.334 | 2.59E-74 | 31 | *MFAP2* | Fibroblasts |
| MMP2 | 1.59E-241 | 0.786771 | 0.675 | 0.069 | 3.41E-237 | 31 | *MMP2* | Fibroblasts |
| COL6A3 | 0 | 0.782808 | 0.624 | 0.004 | 0 | 31 | *COL6A3* | Fibroblasts |
| SERPINE2 | 1.10E-89 | 0.776425 | 0.598 | 0.129 | 2.35E-85 | 31 | *SERPINE2* | Fibroblasts |
| HLA-C | 4.19E-93 | 0.770138 | 0.825 | 0.267 | 8.99E-89 | 31 | *HLA-C* | Fibroblasts |
| CLDN11 | 9.40E-100 | 0.767814 | 0.572 | 0.111 | 2.02E-95 | 31 | *CLDN11* | Fibroblasts |
| COL15A1 | 0 | 0.765986 | 0.459 | 0.001 | 0 | 31 | *COL15A1* | Fibroblasts |
| A2M | 2.39E-206 | 0.762828 | 0.309 | 0.016 | 5.11E-202 | 31 | *A2M* | Fibroblasts |
| IGFBP54 | 7.44E-16 | 0.760075 | 0.706 | 0.479 | 1.60E-11 | 31 | *IGFBP5* | Fibroblasts |
| CALU | 3.49E-72 | 0.758268 | 0.881 | 0.419 | 7.48E-68 | 31 | *CALU* | Fibroblasts |
| GPX8 | 9.61E-183 | 0.756803 | 0.706 | 0.1 | 2.06E-178 | 31 | *GPX8* | Fibroblasts |
| ADM | 1.17E-69 | 0.751103 | 0.495 | 0.113 | 2.50E-65 | 31 | *ADM* | Fibroblasts |
| S100A62 | 1.28E-50 | 0.737139 | 0.892 | 0.522 | 2.75E-46 | 31 | *S100A6* | Fibroblasts |
| ASPN | 0 | 0.734308 | 0.392 | 0.002 | 0 | 31 | *ASPN* | Fibroblasts |
| GSN1 | 1.76E-87 | 0.730086 | 0.758 | 0.213 | 3.77E-83 | 31 | *GSN* | Fibroblasts |
| HERPUD11 | 1.06E-55 | 0.724286 | 0.763 | 0.325 | 2.26E-51 | 31 | *HERPUD1* | Fibroblasts |
| PI15 | 0 | 0.720389 | 0.428 | 0.001 | 0 | 31 | *PI15* | Fibroblasts |
| TPT12 | 1.18E-80 | 0.717368 | 1 | 1 | 2.53E-76 | 31 | *TPT1* | Fibroblasts |
| DHRS32 | 3.71E-57 | 0.702332 | 0.856 | 0.415 | 7.95E-53 | 31 | *DHRS3* | Fibroblasts |
| SERF21 | 8.03E-67 | 0.701632 | 0.995 | 0.932 | 1.72E-62 | 31 | *SERF2* | Fibroblasts |
| EMILIN1 | 0 | 0.700706 | 0.624 | 0.01 | 0 | 31 | *EMILIN1* | Fibroblasts |
| EVA1B | 2.86E-123 | 0.693734 | 0.732 | 0.151 | 6.13E-119 | 31 | *EVA1B* | Fibroblasts |
| FCGRT | 0 | 0.693244 | 0.696 | 0.043 | 0 | 31 | *FCGRT* | Fibroblasts |
| NBL11 | 2.28E-129 | 0.691842 | 0.619 | 0.101 | 4.88E-125 | 31 | *NBL1* | Fibroblasts |
| EMP3 | 8.80E-60 | 0.691683 | 0.799 | 0.34 | 1.89E-55 | 31 | *EMP3* | Fibroblasts |
| MXRA8 | 1.87E-228 | 0.691378 | 0.691 | 0.076 | 4.01E-224 | 31 | *MXRA8* | Fibroblasts |
| NFIA | 6.82E-109 | 0.687558 | 0.68 | 0.146 | 1.46E-104 | 31 | *NFIA* | Fibroblasts |
| TPM1 | 5.46E-06 | 0.684234 | 0.515 | 0.396 | 0.117018 | 31 | *TPM1* | Fibroblasts |

| TIMP32 | 6.94E-110 | 0.683472 | 0.706 | 0.148 | 1.49E-105 | 31 *TIMP3* | Fibroblasts |
| --- | --- | --- | --- | --- | --- | --- | --- |
| HSP90B12 | 2.55E-40 | 0.677747 | 0.933 | 0.691 | 5.46E-36 | 31 *HSP90B1* | Fibroblasts |
| TPM4 | 9.12E-37 | 0.677247 | 0.763 | 0.43 | 1.95E-32 | 31 *TPM4* | Fibroblasts |
| INHBA | 0 | 0.672837 | 0.402 | 0.008 | 0 | 31 *INHBA* | Fibroblasts |
| CYTL1 | 0 | 0.670718 | 0.351 | 0.001 | 0 | 31 *CYTL1* | Fibroblasts |
| CYP26B12 | 1.64E-90 | 0.667525 | 0.66 | 0.151 | 3.51E-86 | 31 *CYP26B1* | Fibroblasts |
| LRP1 | 3.71E-128 | 0.665792 | 0.711 | 0.137 | 7.97E-124 | 31 *LRP1* | Fibroblasts |
| CXCL8 | 0 | 0.665399 | 0.165 | 0.001 | 0 | 31 *CXCL8* | Fibroblasts |
| OSTC | 2.23E-53 | 0.663357 | 0.856 | 0.466 | 4.77E-49 | 31 *OSTC* | Fibroblasts |
| LMNA | 4.05E-136 | 0.658976 | 0.716 | 0.132 | 8.68E-132 | 31 *LMNA* | Fibroblasts |
| MYL9 | 4.80E-164 | 0.654191 | 0.505 | 0.054 | 1.03E-159 | 31 *MYL9* | Fibroblasts |
| ITM2A | 1.59E-270 | 0.64963 | 0.577 | 0.044 | 3.40E-266 | 31 *ITM2A* | Fibroblasts |
| HLA-A | 1.16E-67 | 0.648617 | 0.856 | 0.367 | 2.49E-63 | 31 *HLA-A* | Fibroblasts |
| CTSB | 1.72E-74 | 0.643872 | 0.763 | 0.249 | 3.68E-70 | 31 *CTSB* | Fibroblasts |
| GJA11 | 3.58E-144 | 0.639763 | 0.552 | 0.071 | 7.68E-140 | 31 *GJA1* | Fibroblasts |
| SFRP12 | 4.67E-65 | 0.639154 | 0.649 | 0.191 | 1.00E-60 | 31 *SFRP1* | Fibroblasts |
| ITGB1 | 5.01E-51 | 0.636137 | 0.835 | 0.398 | 1.07E-46 | 31 *ITGB1* | Fibroblasts |
| C1R | 4.26E-219 | 0.634848 | 0.536 | 0.047 | 9.13E-215 | 31 *C1R* | Fibroblasts |
| NRP2 | 1.36E-300 | 0.627257 | 0.485 | 0.027 | 2.91E-296 | 31 *NRP2* | Fibroblasts |
| TINAGL1 | 0 | 0.619187 | 0.515 | 0.011 | 0 | 31 *TINAGL1* | Fibroblasts |
| NOV | 2.01E-218 | 0.618184 | 0.258 | 0.01 | 4.31E-214 | 31 *NOV* | Fibroblasts |
| WWTR11 | 4.08E-83 | 0.617389 | 0.701 | 0.189 | 8.76E-79 | 31 *WWTR1* | Fibroblasts |
| THBS1 | 0 | 0.61693 | 0.314 | 0.008 | 0 | 31 *THBS1* | Fibroblasts |
| P4HA11 | 8.61E-52 | 0.616516 | 0.794 | 0.351 | 1.85E-47 | 31 *P4HA1* | Fibroblasts |
| NCOA7 | 3.31E-84 | 0.616018 | 0.629 | 0.154 | 7.10E-80 | 31 *NCOA7* | Fibroblasts |
| RABAC11 | 9.37E-54 | 0.614719 | 0.912 | 0.501 | 2.01E-49 | 31 *RABAC1* | Fibroblasts |
| CTSK | 0 | 0.614535 | 0.567 | 0.014 | 0 | 31 *CTSK* | Fibroblasts |
| PPIC | 2.11E-213 | 0.61402 | 0.624 | 0.065 | 4.53E-209 | 31 *PPIC* | Fibroblasts |
| IRF1 | 5.39E-100 | 0.609221 | 0.521 | 0.089 | 1.16E-95 | 31 *IRF1* | Fibroblasts |
| NREP2 | 2.31E-27 | 0.604002 | 0.84 | 0.605 | 4.94E-23 | 31 *NREP* | Fibroblasts |
| SQSTM12 | 4.88E-53 | 0.603186 | 0.83 | 0.39 | 1.05E-48 | 31 *SQSTM1* | Fibroblasts |
| RPL10 | 3.52E-73 | 0.594962 | 1 | 1 | 7.55E-69 | 31 *RPL10* | Fibroblasts |
| FHL2 | 5.66E-191 | 0.59395 | 0.495 | 0.045 | 1.21E-186 | 31 *FHL2* | Fibroblasts |
| P4HA2 | 1.63E-92 | 0.590469 | 0.665 | 0.154 | 3.49E-88 | 31 *P4HA2* | Fibroblasts |
| FKBP10 | 3.12E-112 | 0.587469 | 0.716 | 0.153 | 6.69E-108 | 31 *FKBP10* | Fibroblasts |
| FTH12 | 1.25E-46 | 0.584887 | 1 | 0.999 | 2.67E-42 | 31 *FTH1* | Fibroblasts |
| KDELR3 | 1.07E-105 | 0.580671 | 0.572 | 0.105 | 2.30E-101 | 31 *KDELR3* | Fibroblasts |
| OST4 | 4.07E-47 | 0.580646 | 0.933 | 0.644 | 8.72E-43 | 31 *OST4* | Fibroblasts |
| TMSB103 | 3.47E-38 | 0.575485 | 0.99 | 0.966 | 7.44E-34 | 31 *TMSB10* | Fibroblasts |
| PDGFRA | 0 | 0.5748 | 0.557 | 0.031 | 0 | 31 *PDGFRA* | Fibroblasts |
| ALCAM | 3.50E-86 | 0.573991 | 0.598 | 0.134 | 7.50E-82 | 31 *ALCAM* | Fibroblasts |
| MRC2 | 4.36E-131 | 0.573034 | 0.562 | 0.083 | 9.34E-127 | 31 *MRC2* | Fibroblasts |
| NELL2 | 1.80E-58 | 0.572191 | 0.546 | 0.155 | 3.87E-54 | 31 *NELL2* | Fibroblasts |
| ZNF503 | 4.23E-146 | 0.565118 | 0.552 | 0.071 | 9.06E-142 | 31 *ZNF503* | Fibroblasts |
| XBP1 | 2.07E-68 | 0.564157 | 0.784 | 0.27 | 4.43E-64 | 31 *XBP1* | Fibroblasts |
| IFI44L | 0 | 0.56306 | 0.428 | 0.02 | 0 | 31 *IFI44L* | Fibroblasts |
| SERP11 | 4.29E-51 | 0.562761 | 0.814 | 0.381 | 9.19E-47 | 31 *SERP1* | Fibroblasts |
| GADD45B | 3.14E-37 | 0.560987 | 0.423 | 0.127 | 6.74E-33 | 31 *GADD45B* | Fibroblasts |
| LIMA1 | 6.17E-60 | 0.560276 | 0.613 | 0.181 | 1.32E-55 | 31 *LIMA1* | Fibroblasts |
| SMIM3 | 1.35E-199 | 0.559426 | 0.536 | 0.051 | 2.89E-195 | 31 *SMIM3* | Fibroblasts |
| CCDC80 | 2.63E-222 | 0.555553 | 0.474 | 0.035 | 5.64E-218 | 31 *CCDC80* | Fibroblasts |
| FAP | 2.06E-247 | 0.551037 | 0.479 | 0.032 | 4.42E-243 | 31 *FAP* | Fibroblasts |
| S1PR3 | 8.88E-130 | 0.550518 | 0.526 | 0.073 | 1.90E-125 | 31 *S1PR3* | Fibroblasts |
| COL9A31 | 7.62E-50 | 0.550085 | 0.598 | 0.197 | 1.63E-45 | 31 *COL9A3* | Fibroblasts |
| C12orf57 | 4.61E-43 | 0.547845 | 0.933 | 0.647 | 9.88E-39 | 31 *C12orf57* | Fibroblasts |
| PTGIS | 0 | 0.547654 | 0.521 | 0.01 | 0 | 31 *PTGIS* | Fibroblasts |
| FKBP14 | 1.08E-134 | 0.547305 | 0.644 | 0.106 | 2.31E-130 | 31 *FKBP14* | Fibroblasts |
| CD99 | 4.92E-64 | 0.545404 | 0.701 | 0.225 | 1.06E-59 | 31 *CD99* | Fibroblasts |
| IFI16 | 6.94E-162 | 0.541931 | 0.582 | 0.072 | 1.49E-157 | 31 *IFI16* | Fibroblasts |
| PIEZO2 | 9.74E-121 | 0.541806 | 0.541 | 0.082 | 2.09E-116 | 31 *PIEZO2* | Fibroblasts |
| COL5A1 | 3.86E-96 | 0.541529 | 0.577 | 0.112 | 8.27E-92 | 31 *COL5A1* | Fibroblasts |
| COL14A1 | 0 | 0.540808 | 0.433 | 0.002 | 0 | 31 *COL14A1* | Fibroblasts |
| ZFP36 | 7.06E-32 | 0.537358 | 0.598 | 0.257 | 1.51E-27 | 31 *ZFP36* | Fibroblasts |

| FBN11 | 8.28E-78 | 0.536753 | 0.608 | 0.144 | 1.78E-73 | 31 | *FBN1* | Fibroblasts |
| --- | --- | --- | --- | --- | --- | --- | --- | --- |
| CXCL12 | 2.90E-182 | 0.53437 | 0.464 | 0.041 | 6.21E-178 | 31 | *CXCL12* | Fibroblasts |
| TFPI2 | 1.89E-24 | 0.533682 | 0.227 | 0.057 | 4.05E-20 | 31 | *TFPI2* | Fibroblasts |
| SULT1E1 | 0 | 0.529975 | 0.371 | 0.001 | 0 | 31 | *SULT1E1* | Fibroblasts |
| MMP16 | 2.50E-106 | 0.529237 | 0.418 | 0.055 | 5.36E-102 | 31 | *MMP16* | Fibroblasts |
| FSTL1 | 1.14E-96 | 0.526176 | 0.639 | 0.136 | 2.45E-92 | 31 | *FSTL1* | Fibroblasts |
| PDGFRL | 0 | 0.522261 | 0.443 | 0.003 | 0 | 31 | *PDGFRL* | Fibroblasts |
| GJB6 | 0 | 0.52164 | 0.304 | 0 | 0 | 31 | *GJB6* | Fibroblasts |
| EEF1D | 4.58E-37 | 0.520604 | 0.928 | 0.655 | 9.81E-33 | 31 | *EEF1D* | Fibroblasts |
| FAM114A1 | 4.52E-112 | 0.520338 | 0.639 | 0.119 | 9.69E-108 | 31 | *FAM114A1* | Fibroblasts |
| COL16A1 | 0 | 0.517639 | 0.454 | 0.016 | 0 | 31 | *COL16A1* | Fibroblasts |
| RCN3 | 2.96E-294 | 0.515886 | 0.541 | 0.035 | 6.34E-290 | 31 | *RCN3* | Fibroblasts |
| PLAC9 | 0 | 0.51557 | 0.49 | 0.006 | 0 | 31 | *PLAC9* | Fibroblasts |
| CYR61 | 5.61E-12 | 0.514673 | 0.376 | 0.192 | 1.20E-07 | 31 | *CYR61* | Fibroblasts |
| MAFB | 4.02E-106 | 0.512649 | 0.366 | 0.043 | 8.62E-102 | 31 | *MAFB* | Fibroblasts |
| G0S2 | 7.08E-67 | 0.511436 | 0.309 | 0.046 | 1.52E-62 | 31 | *G0S2* | Fibroblasts |
| NPC21 | 3.10E-30 | 0.508279 | 0.835 | 0.485 | 6.64E-26 | 31 | *NPC2* | Fibroblasts |
| CD63 | 2.03E-44 | 0.505062 | 0.979 | 0.869 | 4.35E-40 | 31 | *CD63* | Fibroblasts |
| RPS121 | 1.42E-52 | 0.500097 | 1 | 0.999 | 3.04E-48 | 31 | *RPS12* | Fibroblasts |
| S100A112 | 1.22E-298 | 2.693669 | 0.85 | 0.067 | 2.62E-294 | 32 | *S100A11* | Ocular surface epithelium |
| KRT18 | 2.13E-204 | 2.49332 | 0.812 | 0.091 | 4.57E-200 | 32 | *KRT18* | Ocular surface epithelium |
| LCN2 | 0 | 2.158346 | 0.331 | 0.003 | 0 | 32 | *LCN2* | Ocular surface epithelium |
| IGFBP3 | 0 | 2.135737 | 0.594 | 0.012 | 0 | 32 | *IGFBP3* | Ocular surface epithelium |
| S100A63 | 1.69E-51 | 2.084932 | 0.887 | 0.522 | 3.63E-47 | 32 | *S100A6* | Ocular surface epithelium |
| KRT81 | 2.61E-171 | 2.055495 | 0.857 | 0.124 | 5.59E-167 | 32 | *KRT8* | Ocular surface epithelium |
| KRT19 | 0 | 2.047863 | 0.602 | 0.013 | 0 | 32 | *KRT19* | Ocular surface epithelium |
| B2M2 | 1.98E-65 | 1.997237 | 0.992 | 0.617 | 4.26E-61 | 32 | *B2M* | Ocular surface epithelium |
| RARRES11 | 1.08E-96 | 1.976578 | 0.526 | 0.074 | 2.31E-92 | 32 | *RARRES1* | Ocular surface epithelium |
| FN11 | 1.97E-54 | 1.90981 | 0.316 | 0.043 | 4.23E-50 | 32 | *FN1* | Ocular surface epithelium |
| SPP16 | 2.92E-06 | 1.855938 | 0.632 | 0.671 | 0.062714 | 32 | *SPP1* | Ocular surface epithelium |
| S100A9 | 0 | 1.828036 | 0.165 | 0.001 | 0 | 32 | *S100A9* | Ocular surface epithelium |
| SAT11 | 1.10E-55 | 1.819026 | 0.872 | 0.451 | 2.37E-51 | 32 | *SAT1* | Ocular surface epithelium |
| SST | 3.99E-128 | 1.794455 | 0.18 | 0.006 | 8.55E-124 | 32 | *SST* | Ocular surface epithelium |
| CD243 | 2.81E-55 | 1.728743 | 0.789 | 0.325 | 6.02E-51 | 32 | *CD24* | Ocular surface epithelium |
| IGFBP71 | 6.73E-239 | 1.715583 | 0.82 | 0.077 | 1.44E-234 | 32 | *IGFBP7* | Ocular surface epithelium |
| TFPI21 | 1.23E-239 | 1.694252 | 0.707 | 0.055 | 2.63E-235 | 32 | *TFPI2* | Ocular surface epithelium |
| S100A10 | 0 | 1.681953 | 0.82 | 0.014 | 0 | 32 | *S100A10* | Ocular surface epithelium |
| TPM11 | 2.19E-60 | 1.657825 | 0.872 | 0.394 | 4.70E-56 | 32 | *TPM1* | Ocular surface epithelium |
| ANXA22 | 2.42E-122 | 1.53987 | 0.85 | 0.173 | 5.18E-118 | 32 | *ANXA2* | Ocular surface epithelium |
| HLA-B1 | 1.56E-72 | 1.486612 | 0.759 | 0.216 | 3.35E-68 | 32 | *HLA-B* | Ocular surface epithelium |
| ELF3 | 1.03E-305 | 1.485689 | 0.684 | 0.04 | 2.21E-301 | 32 | *ELF3* | Ocular surface epithelium |
| ANXA12 | 0 | 1.481574 | 0.669 | 0.026 | 0 | 32 | *ANXA1* | Ocular surface epithelium |
| ID12 | 9.52E-73 | 1.411205 | 0.767 | 0.211 | 2.04E-68 | 32 | *ID1* | Ocular surface epithelium |
| TNFSF10 | 4.69E-286 | 1.403258 | 0.594 | 0.031 | 1.00E-281 | 32 | *TNFSF10* | Ocular surface epithelium |
| MMP7 | 0 | 1.354429 | 0.331 | 0.003 | 0 | 32 | *MMP7* | Ocular surface epithelium |
| AKAP121 | 5.76E-32 | 1.35224 | 0.692 | 0.328 | 1.24E-27 | 32 | *AKAP12* | Ocular surface epithelium |
| IER31 | 4.00E-127 | 1.350813 | 0.699 | 0.101 | 8.59E-123 | 32 | *IER3* | Ocular surface epithelium |
| GDF152 | 3.77E-172 | 1.342676 | 0.481 | 0.034 | 8.08E-168 | 32 | *GDF15* | Ocular surface epithelium |
| CLDN4 | 1.18E-210 | 1.335709 | 0.571 | 0.04 | 2.52E-206 | 32 | *CLDN4* | Ocular surface epithelium |
| KRT7 | 1.36E-190 | 1.311865 | 0.248 | 0.008 | 2.91E-186 | 32 | *KRT7* | Ocular surface epithelium |
| PERP | 7.32E-90 | 1.303491 | 0.774 | 0.18 | 1.57E-85 | 32 | *PERP* | Ocular surface epithelium |
| KRT17 | 2.72E-167 | 1.300601 | 0.248 | 0.009 | 5.83E-163 | 32 | *KRT17* | Ocular surface epithelium |
| CAST | 4.53E-59 | 1.294127 | 0.722 | 0.223 | 9.71E-55 | 32 | *CAST* | Ocular surface epithelium |
| HLA-C1 | 9.66E-76 | 1.279084 | 0.857 | 0.268 | 2.07E-71 | 32 | *HLA-C* | Ocular surface epithelium |
| GPRC5A | 3.51E-208 | 1.278206 | 0.444 | 0.024 | 7.52E-204 | 32 | *GPRC5A* | Ocular surface epithelium |
| LY6E1 | 8.04E-129 | 1.227918 | 0.789 | 0.132 | 1.72E-124 | 32 | *LY6E* | Ocular surface epithelium |
| CCL22 | 6.10E-50 | 1.224785 | 0.361 | 0.06 | 1.31E-45 | 32 | *CCL2* | Ocular surface epithelium |
| HLA-A1 | 2.62E-65 | 1.211173 | 0.865 | 0.368 | 5.61E-61 | 32 | *HLA-A* | Ocular surface epithelium |
| BST22 | 2.69E-77 | 1.175345 | 0.549 | 0.096 | 5.76E-73 | 32 | *BST2* | Ocular surface epithelium |
| TNFRSF12A | 5.39E-110 | 1.154864 | 0.774 | 0.144 | 1.16E-105 | 32 | *TNFRSF12A* | Ocular surface epithelium |
| PLAT1 | 2.83E-90 | 1.13003 | 0.451 | 0.056 | 6.08E-86 | 32 | *PLAT* | Ocular surface epithelium |
| PPY | 1.35E-10 | 1.099291 | 0.15 | 0.042 | 2.90E-06 | 32 | *PPY* | Ocular surface epithelium |
| IFI61 | 6.55E-33 | 1.078715 | 0.481 | 0.146 | 1.40E-28 | 32 | *IFI6* | Ocular surface epithelium |

| MYL12A | 6.21E-71 | 1.073113 | 0.932 | 0.425 | 1.33E-66 | 32 | *MYL12A* | Ocular surface epithelium |
| --- | --- | --- | --- | --- | --- | --- | --- | --- |
| WFDC21 | 6.30E-32 | 1.066522 | 0.639 | 0.261 | 1.35E-27 | 32 | *WFDC2* | Ocular surface epithelium |
| MYL12B | 1.44E-66 | 1.052252 | 0.962 | 0.572 | 3.09E-62 | 32 | *MYL12B* | Ocular surface epithelium |
| VAMP8 | 0 | 1.039256 | 0.481 | 0.013 | 0 | 32 | *VAMP8* | Ocular surface epithelium |
| HSPB13 | 6.03E-68 | 0.976959 | 0.895 | 0.292 | 1.29E-63 | 32 | *HSPB1* | Ocular surface epithelium |
| IFITM34 | 2.17E-38 | 0.974512 | 0.797 | 0.379 | 4.65E-34 | 32 | *IFITM3* | Ocular surface epithelium |
| SLPI | 0 | 0.969643 | 0.248 | 0.003 | 0 | 32 | *SLPI* | Ocular surface epithelium |
| CYR611 | 1.87E-36 | 0.967048 | 0.571 | 0.192 | 4.02E-32 | 32 | *CYR61* | Ocular surface epithelium |
| TMSB4X1 | 4.51E-32 | 0.965393 | 0.962 | 0.971 | 9.67E-28 | 32 | *TMSB4X* | Ocular surface epithelium |
| CYBA | 3.48E-90 | 0.961331 | 0.789 | 0.186 | 7.45E-86 | 32 | *CYBA* | Ocular surface epithelium |
| CLDN7 | 0 | 0.954836 | 0.414 | 0.004 | 0 | 32 | *CLDN7* | Ocular surface epithelium |
| LGALS32 | 4.97E-104 | 0.949781 | 0.684 | 0.109 | 1.07E-99 | 32 | *LGALS3* | Ocular surface epithelium |
| TPM41 | 2.00E-38 | 0.942646 | 0.82 | 0.431 | 4.29E-34 | 32 | *TPM4* | Ocular surface epithelium |
| IL32 | 0 | 0.919878 | 0.489 | 0.018 | 0 | 32 | *IL32* | Ocular surface epithelium |
| ATF31 | 1.37E-94 | 0.908705 | 0.571 | 0.085 | 2.94E-90 | 32 | *ATF3* | Ocular surface epithelium |
| ID33 | 1.10E-27 | 0.90253 | 0.699 | 0.323 | 2.35E-23 | 32 | *ID3* | Ocular surface epithelium |
| ANXA4 | 9.95E-245 | 0.892437 | 0.624 | 0.041 | 2.13E-240 | 32 | *ANXA4* | Ocular surface epithelium |
| RAB11FIP1 | 3.74E-52 | 0.890548 | 0.639 | 0.179 | 8.02E-48 | 32 | *RAB11FIP1* | Ocular surface epithelium |
| TMSB104 | 3.00E-32 | 0.876976 | 1 | 0.966 | 6.44E-28 | 32 | *TMSB10* | Ocular surface epithelium |
| CYP1B13 | 5.77E-22 | 0.84903 | 0.684 | 0.352 | 1.24E-17 | 32 | *CYP1B1* | Ocular surface epithelium |
| TGM21 | 0 | 0.835974 | 0.489 | 0.016 | 0 | 32 | *TGM2* | Ocular surface epithelium |
| MGST1 | 1.93E-58 | 0.82247 | 0.406 | 0.066 | 4.14E-54 | 32 | *MGST1* | Ocular surface epithelium |
| GNG111 | 1.95E-31 | 0.817804 | 0.459 | 0.137 | 4.19E-27 | 32 | *GNG11* | Ocular surface epithelium |
| PSCA | 1.22E-227 | 0.815003 | 0.135 | 0.002 | 2.62E-223 | 32 | *PSCA* | Ocular surface epithelium |
| KLF6 | 4.73E-58 | 0.812341 | 0.617 | 0.158 | 1.01E-53 | 32 | *KLF6* | Ocular surface epithelium |
| FHL21 | 1.65E-159 | 0.810897 | 0.534 | 0.046 | 3.55E-155 | 32 | *FHL2* | Ocular surface epithelium |
| PFN1 | 1.33E-45 | 0.801718 | 0.97 | 0.656 | 2.84E-41 | 32 | *PFN1* | Ocular surface epithelium |
| ITGB11 | 8.14E-40 | 0.796512 | 0.835 | 0.399 | 1.75E-35 | 32 | *ITGB1* | Ocular surface epithelium |
| ANXA11 | 1.45E-149 | 0.795873 | 0.722 | 0.089 | 3.11E-145 | 32 | *ANXA11* | Ocular surface epithelium |
| TPD52L11 | 1.17E-103 | 0.794927 | 0.617 | 0.089 | 2.52E-99 | 32 | *TPD52L1* | Ocular surface epithelium |
| PLK21 | 4.54E-70 | 0.79325 | 0.602 | 0.124 | 9.73E-66 | 32 | *PLK2* | Ocular surface epithelium |
| CTGF1 | 3.06E-122 | 0.791785 | 0.361 | 0.026 | 6.57E-118 | 32 | *CTGF* | Ocular surface epithelium |
| TINAGL11 | 0 | 0.780665 | 0.459 | 0.012 | 0 | 32 | *TINAGL1* | Ocular surface epithelium |
| APOE1 | 2.45E-33 | 0.777658 | 0.729 | 0.307 | 5.26E-29 | 32 | *APOE* | Ocular surface epithelium |
| MT2A2 | 2.89E-12 | 0.774422 | 0.323 | 0.132 | 6.19E-08 | 32 | *MT2A* | Ocular surface epithelium |
| NEAT12 | 2.59E-17 | 0.772842 | 0.94 | 0.92 | 5.55E-13 | 32 | *NEAT1* | Ocular surface epithelium |
| YBX31 | 8.90E-46 | 0.754157 | 0.812 | 0.324 | 1.91E-41 | 32 | *YBX3* | Ocular surface epithelium |
| ISG15 | 6.35E-38 | 0.753477 | 0.368 | 0.078 | 1.36E-33 | 32 | *ISG15* | Ocular surface epithelium |
| TXN | 1.21E-37 | 0.751686 | 0.94 | 0.583 | 2.59E-33 | 32 | *TXN* | Ocular surface epithelium |
| C9orf16 | 6.06E-38 | 0.749556 | 0.835 | 0.434 | 1.30E-33 | 32 | *C9orf16* | Ocular surface epithelium |
| TAGLN1 | 3.01E-44 | 0.745697 | 0.308 | 0.049 | 6.46E-40 | 32 | *TAGLN* | Ocular surface epithelium |
| ARPC2 | 5.84E-33 | 0.741744 | 0.865 | 0.527 | 1.25E-28 | 32 | *ARPC2* | Ocular surface epithelium |
| IGFBP62 | 7.27E-19 | 0.739039 | 0.263 | 0.071 | 1.56E-14 | 32 | *IGFBP6* | Ocular surface epithelium |
| SNCG1 | 2.16E-61 | 0.738426 | 0.248 | 0.024 | 4.64E-57 | 32 | *SNCG* | Ocular surface epithelium |
| PSME2 | 2.91E-48 | 0.737129 | 0.699 | 0.227 | 6.25E-44 | 32 | *PSME2* | Ocular surface epithelium |
| PSME1 | 9.69E-43 | 0.736102 | 0.835 | 0.387 | 2.08E-38 | 32 | *PSME1* | Ocular surface epithelium |
| PDLIM1 | 1.09E-82 | 0.732803 | 0.571 | 0.095 | 2.34E-78 | 32 | *PDLIM1* | Ocular surface epithelium |
| SLC40A1 | 9.15E-89 | 0.730819 | 0.391 | 0.042 | 1.96E-84 | 32 | *SLC40A1* | Ocular surface epithelium |
| GSN2 | 1.04E-47 | 0.726064 | 0.684 | 0.215 | 2.23E-43 | 32 | *GSN* | Ocular surface epithelium |
| RPS27L1 | 1.34E-32 | 0.720253 | 0.902 | 0.578 | 2.87E-28 | 32 | *RPS27L* | Ocular surface epithelium |
| PPDPF | 5.33E-30 | 0.720004 | 0.902 | 0.604 | 1.14E-25 | 32 | *PPDPF* | Ocular surface epithelium |
| CTSC1 | 6.27E-65 | 0.71253 | 0.571 | 0.116 | 1.34E-60 | 32 | *CTSC* | Ocular surface epithelium |
| CTSD | 1.89E-36 | 0.708841 | 0.617 | 0.216 | 4.05E-32 | 32 | *CTSD* | Ocular surface epithelium |
| RBPMS | 1.20E-180 | 0.698501 | 0.632 | 0.057 | 2.58E-176 | 32 | *RBPMS* | Ocular surface epithelium |
| NFKBIA | 2.97E-45 | 0.698047 | 0.654 | 0.205 | 6.37E-41 | 32 | *NFKBIA* | Ocular surface epithelium |
| ATP1B11 | 4.36E-29 | 0.697337 | 0.842 | 0.475 | 9.35E-25 | 32 | *ATP1B1* | Ocular surface epithelium |
| DSP | 3.53E-205 | 0.696007 | 0.496 | 0.03 | 7.57E-201 | 32 | *DSP* | Ocular surface epithelium |
| LMNA1 | 2.89E-90 | 0.694815 | 0.699 | 0.133 | 6.20E-86 | 32 | *LMNA* | Ocular surface epithelium |
| CP | 3.84E-13 | 0.693735 | 0.459 | 0.228 | 8.23E-09 | 32 | *CP* | Ocular surface epithelium |
| COL4A1 | 3.32E-65 | 0.692865 | 0.459 | 0.076 | 7.13E-61 | 32 | *COL4A1* | Ocular surface epithelium |
| CTSB1 | 4.02E-49 | 0.691332 | 0.737 | 0.25 | 8.62E-45 | 32 | *CTSB* | Ocular surface epithelium |
| CD151 | 4.76E-52 | 0.686335 | 0.812 | 0.287 | 1.02E-47 | 32 | *CD151* | Ocular surface epithelium |
| TKT3 | 2.12E-27 | 0.675247 | 0.857 | 0.5 | 4.54E-23 | 32 | *TKT* | Ocular surface epithelium |

| IRF11 | 7.75E-67 | 0.673752 | 0.511 | 0.09 | 1.66E-62 | 32 | *IRF1* | Ocular surface epithelium |
| --- | --- | --- | --- | --- | --- | --- | --- | --- |
| TM4SF1 | 0 | 0.664846 | 0.293 | 0.001 | 0 | 32 | *TM4SF1* | Ocular surface epithelium |
| FXYD3 | 7.84E-12 | 0.661975 | 0.361 | 0.177 | 1.68E-07 | 32 | *FXYD3* | Ocular surface epithelium |
| UCA1 | 0 | 0.654703 | 0.256 | 0 | 0 | 32 | *UCA1* | Ocular surface epithelium |
| PPIC1 | 1.27E-163 | 0.653323 | 0.654 | 0.066 | 2.72E-159 | 32 | *PPIC* | Ocular surface epithelium |
| CFI1 | 2.90E-64 | 0.652795 | 0.654 | 0.153 | 6.22E-60 | 32 | *CFI* | Ocular surface epithelium |
| S100A16 | 2.62E-74 | 0.652161 | 0.624 | 0.124 | 5.63E-70 | 32 | *S100A16* | Ocular surface epithelium |
| DUSP1 | 1.55E-28 | 0.647163 | 0.617 | 0.247 | 3.33E-24 | 32 | *DUSP1* | Ocular surface epithelium |
| SPINT2 | 8.26E-31 | 0.644611 | 0.782 | 0.422 | 1.77E-26 | 32 | *SPINT2* | Ocular surface epithelium |
| CDH6 | 7.86E-31 | 0.644586 | 0.391 | 0.103 | 1.68E-26 | 32 | *CDH6* | Ocular surface epithelium |
| DHRS33 | 4.63E-28 | 0.642908 | 0.767 | 0.417 | 9.92E-24 | 32 | *DHRS3* | Ocular surface epithelium |
| CSRP1 | 2.31E-113 | 0.636146 | 0.451 | 0.044 | 4.96E-109 | 32 | *CSRP1* | Ocular surface epithelium |
| PLAU | 2.31E-244 | 0.63499 | 0.301 | 0.009 | 4.95E-240 | 32 | *PLAU* | Ocular surface epithelium |
| TXNIP | 8.48E-15 | 0.63498 | 0.835 | 0.664 | 1.82E-10 | 32 | *TXNIP* | Ocular surface epithelium |
| LGALS3BP | 3.18E-44 | 0.630414 | 0.594 | 0.177 | 6.81E-40 | 32 | *LGALS3BP* | Ocular surface epithelium |
| ERP27 | 0 | 0.62881 | 0.293 | 0.002 | 0 | 32 | *ERP27* | Ocular surface epithelium |
| CEBPD1 | 3.00E-106 | 0.628389 | 0.571 | 0.074 | 6.44E-102 | 32 | *CEBPD* | Ocular surface epithelium |
| RARRES3 | 5.54E-73 | 0.62825 | 0.429 | 0.059 | 1.19E-68 | 32 | *RARRES3* | Ocular surface epithelium |
| UBE2H | 1.76E-32 | 0.628209 | 0.677 | 0.279 | 3.76E-28 | 32 | *UBE2H* | Ocular surface epithelium |
| STAT1 | 5.23E-53 | 0.626663 | 0.519 | 0.117 | 1.12E-48 | 32 | *STAT1* | Ocular surface epithelium |
| CGNL1 | 2.59E-38 | 0.626064 | 0.323 | 0.061 | 5.54E-34 | 32 | *CGNL1* | Ocular surface epithelium |
| SPATS2L | 1.45E-232 | 0.624497 | 0.489 | 0.026 | 3.11E-228 | 32 | *SPATS2L* | Ocular surface epithelium |
| ANXA52 | 2.66E-38 | 0.623609 | 0.692 | 0.258 | 5.69E-34 | 32 | *ANXA5* | Ocular surface epithelium |
| TRAM1 | 1.93E-18 | 0.62231 | 0.639 | 0.342 | 4.14E-14 | 32 | *TRAM1* | Ocular surface epithelium |
| CCDC198 | 0 | 0.615275 | 0.271 | 0.003 | 0 | 32 | *CCDC198* | Ocular surface epithelium |
| MAL2 | 2.06E-72 | 0.614794 | 0.376 | 0.047 | 4.41E-68 | 32 | *MAL2* | Ocular surface epithelium |
| ATP6V0E1 | 2.89E-36 | 0.612957 | 0.812 | 0.393 | 6.20E-32 | 32 | *ATP6V0E1* | Ocular surface epithelium |
| PSMB8 | 3.25E-128 | 0.612483 | 0.632 | 0.077 | 6.97E-124 | 32 | *PSMB8* | Ocular surface epithelium |
| HLA-E | 8.82E-95 | 0.611549 | 0.632 | 0.103 | 1.89E-90 | 32 | *HLA-E* | Ocular surface epithelium |
| CDKN2A | 1.03E-169 | 0.609083 | 0.338 | 0.017 | 2.21E-165 | 32 | *CDKN2A* | Ocular surface epithelium |
| RGS5 | 2.28E-31 | 0.608186 | 0.263 | 0.049 | 4.89E-27 | 32 | *RGS5* | Ocular surface epithelium |
| VMP1 | 5.86E-27 | 0.606818 | 0.617 | 0.264 | 1.26E-22 | 32 | *VMP1* | Ocular surface epithelium |
| CALD11 | 1.80E-19 | 0.604935 | 0.789 | 0.481 | 3.86E-15 | 32 | *CALD1* | Ocular surface epithelium |
| EPCAM | 1.01E-91 | 0.604102 | 0.436 | 0.051 | 2.17E-87 | 32 | *EPCAM* | Ocular surface epithelium |
| AHNAK | 1.82E-85 | 0.601615 | 0.564 | 0.087 | 3.89E-81 | 32 | *AHNAK* | Ocular surface epithelium |
| CYB5A | 8.84E-53 | 0.600669 | 0.594 | 0.147 | 1.90E-48 | 32 | *CYB5A* | Ocular surface epithelium |
| MIR4435-2 | 1.15E-268 | 0.597445 | 0.451 | 0.018 | 2.46E-264 | 32 | *MIR4435-2* | Ocular surface epithelium |
| EZR | 1.76E-30 | 0.596115 | 0.669 | 0.284 | 3.77E-26 | 32 | *EZR* | Ocular surface epithelium |
| NFKBIZ | 1.97E-107 | 0.593516 | 0.466 | 0.05 | 4.22E-103 | 32 | *NFKBIZ* | Ocular surface epithelium |
| ANXA3 | 0 | 0.592209 | 0.414 | 0.01 | 0 | 32 | *ANXA3* | Ocular surface epithelium |
| CDKN2B | 3.64E-246 | 0.591836 | 0.316 | 0.01 | 7.81E-242 | 32 | *CDKN2B* | Ocular surface epithelium |
| RAB25 | 0 | 0.589327 | 0.331 | 0.001 | 0 | 32 | *RAB25* | Ocular surface epithelium |
| CDKN1A1 | 7.80E-21 | 0.588849 | 0.421 | 0.154 | 1.67E-16 | 32 | *CDKN1A* | Ocular surface epithelium |
| RHOC | 2.84E-33 | 0.588515 | 0.752 | 0.332 | 6.10E-29 | 32 | *RHOC* | Ocular surface epithelium |
| HCFC1R1 | 1.49E-46 | 0.588498 | 0.662 | 0.201 | 3.20E-42 | 32 | *HCFC1R1* | Ocular surface epithelium |
| MYL91 | 2.93E-75 | 0.58376 | 0.421 | 0.055 | 6.27E-71 | 32 | *MYL9* | Ocular surface epithelium |
| ADGRF1 | 0 | 0.581 | 0.263 | 0.001 | 0 | 32 | *ADGRF1* | Ocular surface epithelium |
| GRN | 2.42E-46 | 0.579745 | 0.609 | 0.176 | 5.19E-42 | 32 | *GRN* | Ocular surface epithelium |
| FTH13 | 1.51E-28 | 0.579192 | 1 | 0.999 | 3.23E-24 | 32 | *FTH1* | Ocular surface epithelium |
| LIMA11 | 1.50E-30 | 0.575226 | 0.534 | 0.182 | 3.21E-26 | 32 | *LIMA1* | Ocular surface epithelium |
| H2AFJ | 1.01E-35 | 0.572962 | 0.714 | 0.282 | 2.17E-31 | 32 | *H2AFJ* | Ocular surface epithelium |
| CD9 | 5.86E-26 | 0.572442 | 0.624 | 0.256 | 1.26E-21 | 32 | *CD9* | Ocular surface epithelium |
| YWHAB1 | 9.53E-26 | 0.570167 | 0.842 | 0.527 | 2.04E-21 | 32 | *YWHAB* | Ocular surface epithelium |
| GLRX | 1.75E-39 | 0.565471 | 0.376 | 0.077 | 3.76E-35 | 32 | *GLRX* | Ocular surface epithelium |
| LITAF1 | 6.40E-32 | 0.560623 | 0.669 | 0.262 | 1.37E-27 | 32 | *LITAF* | Ocular surface epithelium |
| CARD16 | 0 | 0.560229 | 0.241 | 0 | 0 | 32 | *CARD16* | Ocular surface epithelium |
| PLSCR1 | 3.53E-99 | 0.559694 | 0.579 | 0.082 | 7.57E-95 | 32 | *PLSCR1* | Ocular surface epithelium |
| HOTAIRM1 | 6.24E-71 | 0.559018 | 0.383 | 0.049 | 1.34E-66 | 32 | *HOTAIRM1* | Ocular surface epithelium |
| C12orf75 | 1.14E-17 | 0.556164 | 0.346 | 0.122 | 2.43E-13 | 32 | *C12orf75* | Ocular surface epithelium |
| SPINT1 | 5.67E-265 | 0.55456 | 0.489 | 0.022 | 1.21E-260 | 32 | *SPINT1* | Ocular surface epithelium |
| ARPC3 | 6.97E-30 | 0.552051 | 0.925 | 0.645 | 1.49E-25 | 32 | *ARPC3* | Ocular surface epithelium |
| TUBA1C1 | 3.19E-51 | 0.551763 | 0.602 | 0.15 | 6.85E-47 | 32 | *TUBA1C* | Ocular surface epithelium |
| IFI44L1 | 3.17E-169 | 0.551535 | 0.383 | 0.021 | 6.79E-165 | 32 | *IFI44L* | Ocular surface epithelium |

| 1.54E-56 | 0.549848 | 0.662 | 0.171 | 3.31E-52 | 32 *RAB13* | Ocular surface epithelium |
| --- | --- | --- | --- | --- | --- | --- |
| 1.60E-31 | 0.549149 | 0.504 | 0.153 | 3.44E-27 | 32 *CYP26B1* | Ocular surface epithelium |
| 8.95E-58 | 0.548841 | 0.639 | 0.156 | 1.92E-53 | 32 *ABRACL* | Ocular surface epithelium |
| 3.25E-46 | 0.548735 | 0.391 | 0.074 | 6.96E-42 | 32 *IFI16* | Ocular surface epithelium |
| 2.87E-06 | 0.547497 | 0.887 | 0.802 | 0.06149 | 32 *HSBP1* | Ocular surface epithelium |
| 15E-271 | 0.546173 | 0.398 | 0.014 | 2.46E-267 | 32 *KLF5* | Ocular surface epithelium |
| 4.24E-33 | 0.546049 | 0.797 | 0.384 | 9.10E-29 | 32 *CLIC1* | Ocular surface epithelium |
| 31E-106 | 0.545317 | 0.541 | 0.067 | 1.35E-101 | 32 *B4GALT1* | Ocular surface epithelium |
| 1.16E-20 | 0.543457 | 0.744 | 0.442 | 2.49E-16 | 32 *CST3* | Ocular surface epithelium |
| 3.93E-33 | 0.543286 | 0.699 | 0.277 | 8.42E-29 | 32 *SPTBN1* | Ocular surface epithelium |
| 1.15E-28 | 0.543162 | 0.797 | 0.392 | 2.47E-24 | 32 *SQSTM1* | Ocular surface epithelium |
| 9.32E-29 | 0.542591 | 0.774 | 0.367 | 2.00E-24 | 32 *NFE2L2* | Ocular surface epithelium |
| 4.47E-30 | 0.538549 | 0.985 | 0.869 | 9.59E-26 | 32 *CD63* | Ocular surface epithelium |
| 3.39E-24 | 0.538153 | 0.564 | 0.22 | 7.27E-20 | 32 *CTSL* | Ocular surface epithelium |
| 2.10E-94 | 0.537036 | 0.489 | 0.061 | 4.51E-90 | 32 *LAMB1* | Ocular surface epithelium |
| 18E-149 | 0.534999 | 0.323 | 0.017 | 1.54E-144 | 32 *IFIT3* | Ocular surface epithelium |
| 5.59E-24 | 0.534363 | 0.278 | 0.066 | 1.20E-19 | 32 *SGK1* | Ocular surface epithelium |
| 0 | 0.534224 | 0.226 | 0 | 0 | 32 *GABRP* | Ocular surface epithelium |
| 4.37E-33 | 0.53322 | 0.504 | 0.152 | 9.38E-29 | 32 *SERPING1* | Ocular surface epithelium |
| 3.37E-11 | 0.529392 | 0.504 | 0.296 | 7.23E-07 | 32 *FAM3C* | Ocular surface epithelium |
| 14E-137 | 0.527036 | 0.338 | 0.02 | 2.45E-133 | 32 *PHLDA2* | Ocular surface epithelium |
| 1.31E-19 | 0.525914 | 0.218 | 0.05 | 2.80E-15 | 32 *ALDH1A3* | Ocular surface epithelium |
| 6.28E-26 | 0.522838 | 0.947 | 0.746 | 1.35E-21 | 32 *COX5B* | Ocular surface epithelium |
| 5.06E-71 | 0.521799 | 0.128 | 0.006 | 1.09E-66 | 32 *SERPINE1* | Ocular surface epithelium |
| 2.06E-19 | 0.521672 | 0.444 | 0.18 | 4.42E-15 | 32 *COL4A2* | Ocular surface epithelium |
| 1.05E-30 | 0.521147 | 0.977 | 0.932 | 2.24E-26 | 32 *SERF2* | Ocular surface epithelium |
| 1.10E-13 | 0.520369 | 0.759 | 0.521 | 2.36E-09 | 32 *DBI* | Ocular surface epithelium |
| 5.38E-25 | 0.519557 | 1 | 0.994 | 1.15E-20 | 32 *GAPDH* | Ocular surface epithelium |
| 2.76E-44 | 0.516938 | 1 | 1 | 5.91E-40 | 32 *TPT1* | Ocular surface epithelium |
| 9.78E-76 | 0.514911 | 0.556 | 0.094 | 2.10E-71 | 32 *MYH9* | Ocular surface epithelium |
| 1.65E-42 | 0.51221 | 0.496 | 0.124 | 3.53E-38 | 32 *DSG2* | Ocular surface epithelium |
| 87E-122 | 0.511637 | 0.346 | 0.024 | 6.16E-118 | 32 *DCDC2* | Ocular surface epithelium |
| 8.29E-23 | 0.510591 | 0.195 | 0.036 | 1.78E-18 | 32 *COL3A1* | Ocular surface epithelium |
| 1.71E-25 | 0.509643 | 0.662 | 0.309 | 3.66E-21 | 32 *ACTN4* | Ocular surface epithelium |
| 1.27E-39 | 0.508429 | 0.737 | 0.269 | 2.71E-35 | 32 *TSTD1* | Ocular surface epithelium |
| 0 | 0.504911 | 0.414 | 0.01 | 0 | 32 *SP100* | Ocular surface epithelium |
| 84E-136 | 0.5026 | 0.323 | 0.019 | 3.96E-132 | 32 *MX1* | Ocular surface epithelium |
| 0.000883 | 0.501129 | 0.586 | 0.529 | 1 | 32 *TSC22D1* | Ocular surface epithelium |
| 8.47E-45 | 0.501074 | 0.383 | 0.072 | 1.82E-40 | 32 *RAP2B* | Ocular surface epithelium |
| 0 | 0.500016 | 0.263 | 0.003 | 0 | 32 *MALL* | Ocular surface epithelium |
| 6.79E-47 | 3.168884 | 0.341 | 0.057 | 1.46E-42 | 33 *TTR* | RPE |
| 85E-238 | 2.858871 | 0.93 | 0.108 | 3.98E-234 | 33 *PMEL* | RPE |
| 13E-144 | 2.731653 | 0.891 | 0.173 | 4.56E-140 | 33 *DCT* | RPE |
| 59E-136 | 2.476856 | 0.953 | 0.218 | 5.56E-132 | 33 *PTGDS* | RPE |
| 15E-185 | 2.428451 | 0.953 | 0.148 | 2.48E-181 | 33 *TIMP3* | RPE |
| 35E-136 | 2.248502 | 0.93 | 0.21 | 5.04E-132 | 33 *SERPINF1* | RPE |
| 25E-119 | 2.161732 | 0.992 | 0.305 | 1.13E-114 | 33 *APOE* | RPE |
| 75E-281 | 2.084924 | 0.767 | 0.055 | 3.75E-277 | 33 *TFPI2* | RPE |
| 0 | 1.967407 | 0.868 | 0.019 | 0 | 33 *TRPM1* | RPE |
| 0 | 1.86866 | 0.798 | 0.042 | 0 | 33 *CRYAB* | RPE |
| 0 | 1.80919 | 0.884 | 0.008 | 0 | 33 *TYRP1* | RPE |
| 1.17E-91 | 1.788007 | 0.93 | 0.331 | 2.50E-87 | 33 *TMEM98* | RPE |
| 22E-179 | 1.78314 | 0.791 | 0.094 | 1.33E-174 | 33 *ENPP2* | RPE |
| 0 | 1.759351 | 0.876 | 0.022 | 0 | 33 *ELN* | RPE |
| 97E-103 | 1.721837 | 0.829 | 0.191 | 1.28E-98 | 33 *SFRP1* | RPE |
| 2.29E-82 | 1.660979 | 0.938 | 0.406 | 4.91E-78 | 33 *SLC2A1* | RPE |
| 59E-276 | 1.607669 | 0.891 | 0.077 | 7.71E-272 | 33 *IGFBP7* | RPE |
| 0 | 1.603917 | 0.783 | 0.028 | 0 | 33 *COL8A1* | RPE |
| 1.03E-56 | 1.566611 | 0.93 | 0.478 | 2.20E-52 | 33 *IGFBP5* | RPE |
| 07E-137 | 1.515867 | 0.504 | 0.045 | 4.45E-133 | 33 *CXCL14* | RPE |
| 4.47E-59 | 1.510217 | 0.899 | 0.441 | 9.59E-55 | 33 *CST3* | RPE |
| 08E-177 | 1.379141 | 0.814 | 0.099 | 1.30E-172 | 33 *HSD17B2* | RPE |
| 19E-247 | 1.322416 | 0.822 | 0.071 | 1.76E-242 | 33 *GJA1* | RPE |

| RAB13 |
| --- |
| CYP26B13 |
| ABRACL |
| IFI161 |
| HSBP1 |
| KLF5 1. |
| CLIC1 |
| B4GALT1 6. |
| CST3 |
| SPTBN1 |
| SQSTM13 |
| NFE2L2 |
| CD631 |
| CTSL |
| LAMB1 |
| IFIT3 7. |
| SGK1 |
| GABRP |
| SERPING1 |
| FAM3C |
| PHLDA2 1. |
| ALDH1A3 |
| COX5B |
| SERPINE1 |
| COL4A2 |
| SERF22 |
| DBI2 |
| GAPDH1 |
| TPT13 |
| MYH9 |
| DSG2 |
| DCDC2 2. |
| COL3A11 |
| ACTN4 |
| TSTD1 |
| SP100 |
| MX1 1. |
| TSC22D12 |
| RAP2B |
| MALL |
| TTR |
| PMEL 1. |
| DCT2 2. |
| PTGDS1 2. |
| TIMP33 1. |
| SERPINF1 2. |
| APOE2 5. |
| TFPI22 1. |
| TRPM1 |
| CRYAB1 |
| TYRP1 |
| TMEM98 |
| ENPP21 6. |
| ELN1 |
| SFRP13 5. |
| SLC2A12 |
| IGFBP72 3. |
| COL8A1 |
| IGFBP55 |
| CXCL141 2. |
| CST31 |
| HSD17B21 6. |
| GJA12 8. |

| SPARC2 | 8.08E-85 | 1.253902 | 0.907 | 0.307 | 1.73E-80 | 33 *SPARC* | RPE |
| --- | --- | --- | --- | --- | --- | --- | --- |
| GNG112 | 6.67E-135 | 1.24929 | 0.829 | 0.135 | 1.43E-130 | 33 *GNG11* | RPE |
| RLBP1 | 1.91E-163 | 1.226117 | 0.659 | 0.068 | 4.09E-159 | 33 *RLBP1* | RPE |
| DUSP4 | 5.88E-276 | 1.218477 | 0.698 | 0.044 | 1.26E-271 | 33 *DUSP4* | RPE |
| GNGT11 | 1.76E-124 | 1.19855 | 0.605 | 0.072 | 3.76E-120 | 33 *GNGT1* | RPE |
| SFRP5 | 0 | 1.176178 | 0.636 | 0.004 | 0 | 33 *SFRP5* | RPE |
| SDCBP1 | 1.06E-76 | 1.145376 | 0.938 | 0.367 | 2.28E-72 | 33 *SDCBP* | RPE |
| CLU3 | 5.23E-38 | 1.128736 | 0.992 | 0.753 | 1.12E-33 | 33 *CLU* | RPE |
| FRZB | 1.95E-126 | 1.098725 | 0.651 | 0.085 | 4.19E-122 | 33 *FRZB* | RPE |
| ATP6V1C2 | 0 | 1.090087 | 0.69 | 0.014 | 0 | 33 *ATP6V1C2* | RPE |
| GPNMB | 0 | 0.982608 | 0.713 | 0.021 | 0 | 33 *GPNMB* | RPE |
| BEST1 | 1.77E-265 | 0.979076 | 0.504 | 0.023 | 3.79E-261 | 33 *BEST1* | RPE |
| GSTP11 | 5.70E-52 | 0.975804 | 1 | 0.959 | 1.22E-47 | 33 *GSTP1* | RPE |
| TSPAN10 | 0 | 0.974253 | 0.775 | 0.032 | 0 | 33 *TSPAN10* | RPE |
| PLTP | 1.04E-108 | 0.968608 | 0.822 | 0.165 | 2.23E-104 | 33 *PLTP* | RPE |
| B2M3 | 2.19E-37 | 0.960073 | 0.953 | 0.618 | 4.70E-33 | 33 *B2M* | RPE |
| CD632 | 5.69E-57 | 0.950898 | 0.984 | 0.869 | 1.22E-52 | 33 *CD63* | RPE |
| CYP1B14 | 1.69E-29 | 0.931382 | 0.729 | 0.351 | 3.63E-25 | 33 *CYP1B1* | RPE |
| FAM84B | 5.52E-174 | 0.920851 | 0.705 | 0.073 | 1.18E-169 | 33 *FAM84B* | RPE |
| RGR | 7.56E-145 | 0.913996 | 0.45 | 0.034 | 1.62E-140 | 33 *RGR* | RPE |
| ASAH1 | 2.08E-57 | 0.897472 | 0.86 | 0.357 | 4.46E-53 | 33 *ASAH1* | RPE |
| NPC22 | 1.06E-52 | 0.895906 | 0.938 | 0.486 | 2.27E-48 | 33 *NPC2* | RPE |
| APP | 2.34E-51 | 0.880673 | 0.899 | 0.429 | 5.02E-47 | 33 *APP* | RPE |
| PCP4 | 7.81E-65 | 0.878826 | 0.543 | 0.1 | 1.67E-60 | 33 *PCP4* | RPE |
| GSN3 | 3.88E-37 | 0.878749 | 0.628 | 0.216 | 8.32E-33 | 33 *GSN* | RPE |
| TSC22D4 | 3.90E-57 | 0.86904 | 0.643 | 0.166 | 8.37E-53 | 33 *TSC22D4* | RPE |
| HLA-B2 | 5.73E-37 | 0.864118 | 0.62 | 0.217 | 1.23E-32 | 33 *HLA-B* | RPE |
| NCCRP1 | 0 | 0.856417 | 0.612 | 0.015 | 0 | 33 *NCCRP1* | RPE |
| CDO1 | 1.82E-96 | 0.846937 | 0.698 | 0.126 | 3.91E-92 | 33 *CDO1* | RPE |
| EFEMP1 | 2.60E-105 | 0.846724 | 0.643 | 0.095 | 5.59E-101 | 33 *EFEMP1* | RPE |
| CTNNAL1 | 1.54E-36 | 0.844787 | 0.822 | 0.406 | 3.30E-32 | 33 *CTNNAL1* | RPE |
| HLA-A2 | 1.62E-42 | 0.839715 | 0.814 | 0.368 | 3.47E-38 | 33 *HLA-A* | RPE |
| RNASE1 | 1.09E-255 | 0.838247 | 0.24 | 0.005 | 2.34E-251 | 33 *RNASE1* | RPE |
| SESN31 | 1.50E-38 | 0.838016 | 0.752 | 0.338 | 3.23E-34 | 33 *SESN3* | RPE |
| CPEB4 | 3.89E-40 | 0.817456 | 0.721 | 0.281 | 8.33E-36 | 33 *CPEB4* | RPE |
| TRPM31 | 8.18E-77 | 0.802234 | 0.636 | 0.123 | 1.75E-72 | 33 *TRPM3* | RPE |
| TMEM176 | 2.34E-49 | 0.800744 | 0.628 | 0.17 | 5.02E-45 | 33 *TMEM176* | RPE |
| COTL12 | 2.35E-53 | 0.787052 | 0.791 | 0.278 | 5.04E-49 | 33 *COTL1* | RPE |
| MYL92 | 2.66E-208 | 0.767466 | 0.674 | 0.054 | 5.70E-204 | 33 *MYL9* | RPE |
| COL9A21 | 1.25E-86 | 0.761545 | 0.752 | 0.152 | 2.69E-82 | 33 *COL9A2* | RPE |
| MITF | 3.40E-284 | 0.757697 | 0.698 | 0.043 | 7.30E-280 | 33 *MITF* | RPE |
| BMP7 | 8.90E-282 | 0.749733 | 0.628 | 0.034 | 1.91E-277 | 33 *BMP7* | RPE |
| TPM12 | 6.71E-26 | 0.731098 | 0.744 | 0.395 | 1.44E-21 | 33 *TPM1* | RPE |
| SARAF | 7.14E-24 | 0.728766 | 0.891 | 0.656 | 1.53E-19 | 33 *SARAF* | RPE |
| TMEM176 | 1.10E-53 | 0.725455 | 0.636 | 0.166 | 2.36E-49 | 33 *TMEM176* | RPE |
| WWTR12 | 1.98E-45 | 0.720095 | 0.643 | 0.191 | 4.24E-41 | 33 *WWTR1* | RPE |
| MT-CO1 | 1.71E-27 | 0.713366 | 0.992 | 0.996 | 3.66E-23 | 33 *MT-CO1* | RPE |
| ITM2C1 | 1.34E-31 | 0.708486 | 0.837 | 0.503 | 2.88E-27 | 33 *ITM2C* | RPE |
| SLC3A21 | 8.97E-39 | 0.707131 | 0.806 | 0.379 | 1.92E-34 | 33 *SLC3A2* | RPE |
| S100B | 1.20E-99 | 0.706873 | 0.349 | 0.029 | 2.58E-95 | 33 *S100B* | RPE |
| MT-CO2 | 2.05E-28 | 0.703716 | 1 | 0.995 | 4.39E-24 | 33 *MT-CO2* | RPE |
| TSHZ2 | 2.00E-53 | 0.702264 | 0.62 | 0.157 | 4.29E-49 | 33 *TSHZ2* | RPE |
| BST23 | 1.14E-95 | 0.700111 | 0.628 | 0.096 | 2.46E-91 | 33 *BST2* | RPE |
| ITGAV | 1.85E-58 | 0.698002 | 0.628 | 0.154 | 3.96E-54 | 33 *ITGAV* | RPE |
| SLC9A3R1 | 2.49E-151 | 0.694433 | 0.636 | 0.066 | 5.34E-147 | 33 *SLC9A3R1* | RPE |
| CTSD1 | 4.21E-59 | 0.694062 | 0.744 | 0.215 | 9.02E-55 | 33 *CTSD* | RPE |
| COLEC121 | 7.02E-77 | 0.686833 | 0.721 | 0.157 | 1.51E-72 | 33 *COLEC12* | RPE |
| ATP1B12 | 1.22E-35 | 0.679006 | 0.884 | 0.475 | 2.62E-31 | 33 *ATP1B1* | RPE |
| CTSB2 | 2.75E-54 | 0.677757 | 0.767 | 0.25 | 5.89E-50 | 33 *CTSB* | RPE |
| ITM2B1 | 5.77E-41 | 0.677587 | 1 | 0.896 | 1.24E-36 | 33 *ITM2B* | RPE |
| PLIN2 | 2.19E-30 | 0.672218 | 0.698 | 0.294 | 4.70E-26 | 33 *PLIN2* | RPE |
| CLIC6 | 2.39E-133 | 0.671907 | 0.465 | 0.039 | 5.12E-129 | 33 *CLIC6* | RPE |
| SLC16A3 | 1.61E-84 | 0.664111 | 0.643 | 0.117 | 3.46E-80 | 33 *SLC16A3* | RPE |

| OSBPL1A | 1.08E-50 | 0.649197 | 0.721 | 0.226 | 2.32E-46 | 33 | *OSBPL1A* | RPE |
| --- | --- | --- | --- | --- | --- | --- | --- | --- |
| MYRF | 0 | 0.643763 | 0.605 | 0.021 | 0 | 33 | *MYRF* | RPE |
| CRISPLD1 | 2.31E-50 | 0.64066 | 0.45 | 0.089 | 4.96E-46 | 33 | *CRISPLD1* | RPE |
| HSPB14 | 2.24E-43 | 0.639144 | 0.783 | 0.293 | 4.80E-39 | 33 | *HSPB1* | RPE |
| PSAP | 9.52E-35 | 0.636477 | 0.876 | 0.505 | 2.04E-30 | 33 | *PSAP* | RPE |
| COL8A2 | 0 | 0.630842 | 0.473 | 0.006 | 0 | 33 | *COL8A2* | RPE |
| TPRN | 4.91E-247 | 0.627793 | 0.574 | 0.033 | 1.05E-242 | 33 | *TPRN* | RPE |
| DNAJC3 | 2.76E-46 | 0.626819 | 0.636 | 0.19 | 5.91E-42 | 33 | *DNAJC3* | RPE |
| CTGF2 | 2.33E-161 | 0.624295 | 0.419 | 0.026 | 5.00E-157 | 33 | *CTGF* | RPE |
| TPD52L12 | 3.09E-84 | 0.617574 | 0.574 | 0.089 | 6.62E-80 | 33 | *TPD52L1* | RPE |
| CYP26A16 | 2.47E-11 | 0.611996 | 0.783 | 0.557 | 5.30E-07 | 33 | *CYP26A1* | RPE |
| SLC7A8 | 1.13E-256 | 0.608087 | 0.535 | 0.027 | 2.43E-252 | 33 | *SLC7A8* | RPE |
| MFAP3L | 2.75E-171 | 0.601127 | 0.558 | 0.044 | 5.90E-167 | 33 | *MFAP3L* | RPE |
| LAPTM4B3 | 4.53E-31 | 0.600954 | 0.899 | 0.495 | 9.72E-27 | 33 | *LAPTM4B* | RPE |
| SSNA1 | 7.71E-36 | 0.600291 | 0.713 | 0.304 | 1.65E-31 | 33 | *SSNA1* | RPE |
| MT-ND4 | 3.50E-25 | 0.596972 | 1 | 0.993 | 7.50E-21 | 33 | *MT-ND4* | RPE |
| STK24 | 3.41E-63 | 0.595784 | 0.55 | 0.109 | 7.30E-59 | 33 | *STK24* | RPE |
| REEP5 | 2.22E-28 | 0.591957 | 0.651 | 0.278 | 4.76E-24 | 33 | *REEP5* | RPE |
| HOMER3 | 9.28E-53 | 0.590763 | 0.55 | 0.125 | 1.99E-48 | 33 | *HOMER3* | RPE |
| CALD12 | 5.05E-25 | 0.590544 | 0.837 | 0.481 | 1.08E-20 | 33 | *CALD1* | RPE |
| GEM | 2.56E-132 | 0.587498 | 0.535 | 0.052 | 5.49E-128 | 33 | *GEM* | RPE |
| TMEM59 | 1.31E-25 | 0.583359 | 0.876 | 0.653 | 2.81E-21 | 33 | *TMEM59* | RPE |
| TYR | 0 | 0.580847 | 0.543 | 0.002 | 0 | 33 | *TYR* | RPE |
| GPX41 | 1.31E-26 | 0.57913 | 0.977 | 0.872 | 2.81E-22 | 33 | *GPX4* | RPE |
| BCAT1 | 1.84E-78 | 0.574738 | 0.457 | 0.062 | 3.95E-74 | 33 | *BCAT1* | RPE |
| PDPN | 2.84E-215 | 0.566323 | 0.527 | 0.031 | 6.09E-211 | 33 | *PDPN* | RPE |
| ZNF5031 | 1.19E-91 | 0.565657 | 0.535 | 0.072 | 2.56E-87 | 33 | *ZNF503* | RPE |
| PRSS33 | 1.30E-61 | 0.563438 | 0.403 | 0.06 | 2.78E-57 | 33 | *PRSS33* | RPE |
| C1R1 | 1.23E-124 | 0.563027 | 0.504 | 0.048 | 2.65E-120 | 33 | *C1R* | RPE |
| SGK11 | 3.05E-64 | 0.552732 | 0.434 | 0.065 | 6.53E-60 | 33 | *SGK1* | RPE |
| NEAT13 | 8.49E-09 | 0.548281 | 0.961 | 0.92 | 0.000182 | 33 | *NEAT1* | RPE |
| TKT4 | 6.87E-30 | 0.545915 | 0.884 | 0.5 | 1.47E-25 | 33 | *TKT* | RPE |
| TTLL4 | 4.14E-64 | 0.545284 | 0.519 | 0.094 | 8.88E-60 | 33 | *TTLL4* | RPE |
| CCL23 | 6.84E-34 | 0.5439 | 0.318 | 0.06 | 1.47E-29 | 33 | *CCL2* | RPE |
| CTSH | 9.28E-139 | 0.542028 | 0.558 | 0.054 | 1.99E-134 | 33 | *CTSH* | RPE |
| TRIOBP | 3.68E-38 | 0.541741 | 0.574 | 0.171 | 7.89E-34 | 33 | *TRIOBP* | RPE |
| VEGFA1 | 2.14E-31 | 0.541171 | 0.612 | 0.217 | 4.58E-27 | 33 | *VEGFA* | RPE |
| LTBP1 | 1.73E-99 | 0.53986 | 0.574 | 0.078 | 3.71E-95 | 33 | *LTBP1* | RPE |
| CSTB | 3.48E-25 | 0.535259 | 0.868 | 0.561 | 7.46E-21 | 33 | *CSTB* | RPE |
| APLP2 | 9.67E-23 | 0.53434 | 0.837 | 0.58 | 2.07E-18 | 33 | *APLP2* | RPE |
| ITGB8 | 0.000665 | 0.531705 | 0.481 | 0.369 | 1 | 33 | *ITGB8* | RPE |
| PRDX14 | 7.45E-18 | 0.531592 | 0.953 | 0.816 | 1.60E-13 | 33 | *PRDX1* | RPE |
| NREP3 | 1.14E-23 | 0.53145 | 0.899 | 0.606 | 2.45E-19 | 33 | *NREP* | RPE |
| BHLHE40 | 9.36E-73 | 0.528137 | 0.473 | 0.07 | 2.01E-68 | 33 | *BHLHE40* | RPE |
| ATP1A1 | 1.21E-28 | 0.52637 | 0.667 | 0.279 | 2.59E-24 | 33 | *ATP1A1* | RPE |
| DHRS34 | 8.73E-23 | 0.526286 | 0.775 | 0.417 | 1.87E-18 | 33 | *DHRS3* | RPE |
| MT-CYB | 4.19E-18 | 0.524593 | 1 | 0.992 | 8.98E-14 | 33 | *MT-CYB* | RPE |
| LAPTM4A2 | 7.42E-30 | 0.524105 | 0.938 | 0.59 | 1.59E-25 | 33 | *LAPTM4A* | RPE |
| CHCHD101 | 8.46E-31 | 0.52377 | 0.69 | 0.269 | 1.81E-26 | 33 | *CHCHD10* | RPE |
| ID34 | 1.98E-13 | 0.51925 | 0.597 | 0.323 | 4.24E-09 | 33 | *ID3* | RPE |
| HLA-C2 | 9.18E-28 | 0.518842 | 0.659 | 0.269 | 1.97E-23 | 33 | *HLA-C* | RPE |
| ALDH1A31 | 2.69E-61 | 0.518043 | 0.364 | 0.049 | 5.76E-57 | 33 | *ALDH1A3* | RPE |
| DSP1 | 1.71E-148 | 0.516698 | 0.434 | 0.03 | 3.67E-144 | 33 | *DSP* | RPE |
| LGALS3BP1 | 3.08E-45 | 0.514314 | 0.628 | 0.177 | 6.61E-41 | 33 | *LGALS3BP* | RPE |
| IGFBP63 | 5.24E-37 | 0.513141 | 0.357 | 0.07 | 1.12E-32 | 33 | *IGFBP6* | RPE |
| POLR2L | 7.30E-20 | 0.508838 | 0.806 | 0.558 | 1.56E-15 | 33 | *POLR2L* | RPE |
| IER32 | 3.35E-30 | 0.507745 | 0.403 | 0.103 | 7.19E-26 | 33 | *IER3* | RPE |
| SERF23 | 3.04E-26 | 0.507414 | 0.992 | 0.932 | 6.51E-22 | 33 | *SERF2* | RPE |
| CYP27A1 | 1.79E-49 | 0.507281 | 0.535 | 0.125 | 3.84E-45 | 33 | *CYP27A1* | RPE |
| UQCRB | 2.26E-25 | 0.506248 | 0.984 | 0.892 | 4.85E-21 | 33 | *UQCRB* | RPE |
| LAYN | 1.11E-70 | 0.505914 | 0.426 | 0.059 | 2.39E-66 | 33 | *LAYN* | RPE |
| BMP4 | 0 | 0.504136 | 0.504 | 0.01 | 0 | 33 | *BMP4* | RPE |
| TPM21 | 2.72E-71 | 0.500555 | 0.612 | 0.114 | 5.83E-67 | 33 | *TPM2* | RPE |

CYP1B15 DAPL12 SFRP25 PCLAF6 DUT4 TKT5 COL1A22 PSAT12 TYMS6 HSD17B22 ALDH1A12 CENPH2 PTN4 HELLS5 EFNA52 GCHFR1 PARD32 IGFBP64 CENPU3 TUBA1B7 MDK

| 9.30E-21 | 1.09347 | 0.875 | 0.352 | 1.99E-16 | 34 | *CYP1B1* | Late RPCs |
| --- | --- | --- | --- | --- | --- | --- | --- |
| 3.76E-21 | 0.993692 | 0.979 | 0.536 | 8.07E-17 | 34 | *DAPL1* | Late RPCs |
| 1.92E-14 | 0.958129 | 0.958 | 0.707 | 4.11E-10 | 34 | *SFRP2* | Late RPCs |
| 4.34E-36 | 0.951219 | 0.917 | 0.222 | 9.30E-32 | 34 | *PCLAF* | Late RPCs |
| 6.62E-25 | 0.886781 | 1 | 0.478 | 1.42E-20 | 34 | *DUT* | Late RPCs |
| 3.04E-18 | 0.818321 | 0.979 | 0.501 | 6.53E-14 | 34 | *TKT* | Late RPCs |
| 7.18E-27 | 0.808145 | 0.958 | 0.362 | 1.54E-22 | 34 | *COL1A2* | Late RPCs |
| 3.89E-33 | 0.787276 | 0.833 | 0.188 | 8.34E-29 | 34 | *PSAT1* | Late RPCs |
| 2.84E-24 | 0.776081 | 0.958 | 0.343 | 6.10E-20 | 34 | *TYMS* | Late RPCs |
| 2.87E-44 | 0.697337 | 0.708 | 0.101 | 6.16E-40 | 34 | *HSD17B2* | Late RPCs |
| 3.30E-25 | 0.696464 | 0.833 | 0.216 | 7.08E-21 | 34 | *ALDH1A1* | Late RPCs |
| 6.27E-32 | 0.695892 | 0.854 | 0.206 | 1.34E-27 | 34 | *CENPH* | Late RPCs |
| 3.97E-10 | 0.69574 | 0.812 | 0.455 | 8.52E-06 | 34 | *PTN* | Late RPCs |
| 5.71E-26 | 0.694874 | 0.917 | 0.271 | 1.22E-21 | 34 | *HELLS* | Late RPCs |
| 1.26E-35 | 0.694506 | 0.854 | 0.175 | 2.70E-31 | 34 | *EFNA5* | Late RPCs |
| 3.55E-21 | 0.673381 | 0.792 | 0.254 | 7.60E-17 | 34 | *GCHFR* | Late RPCs |
| 4.71E-23 | 0.66136 | 0.708 | 0.179 | 1.01E-18 | 34 | *PARD3* | Late RPCs |
| 2.37E-23 | 0.655381 | 0.438 | 0.071 | 5.09E-19 | 34 | *IGFBP6* | Late RPCs |
| 3.49E-31 | 0.651263 | 0.854 | 0.191 | 7.48E-27 | 34 | *CENPU* | Late RPCs |
| 1.01E-15 | 0.643859 | 1 | 0.891 | 2.16E-11 | 34 | *TUBA1B* | Late RPCs |
| 3.16E-11 | 0.628106 | 0.958 | 0.64 | 6.77E-07 | 34 | *MDK* | Late RPCs |
| 3.33E-21 | 0.623867 | 0.812 | 0.271 | 7.14E-17 | 34 | *GGH* | Late RPCs |
| 1.51E-31 | 0.614436 | 0.625 | 0.108 | 3.23E-27 | 34 | *LRRC17* | Late RPCs |
| 8.60E-16 | 0.607345 | 0.979 | 0.487 | 1.84E-11 | 34 | *NPC2* | Late RPCs |
| 5.77E-29 | 0.596441 | 0.833 | 0.199 | 1.24E-24 | 34 | *COL9A3* | Late RPCs |
| 1.17E-24 | 0.595016 | 0.75 | 0.196 | 2.50E-20 | 34 | *GINS2* | Late RPCs |
| 4.80E-19 | 0.589264 | 0.625 | 0.168 | 1.03E-14 | 34 | *DHFR* | Late RPCs |
| 2.29E-12 | 0.582143 | 0.854 | 0.38 | 4.90E-08 | 34 | *IFITM3* | Late RPCs |
| 3.86E-16 | 0.580987 | 0.854 | 0.338 | 8.28E-12 | 34 | *MFAP2* | Late RPCs |
| 2.63E-21 | 0.574218 | 0.625 | 0.154 | 5.63E-17 | 34 | *COL9A2* | Late RPCs |
| 1.74E-18 | 0.566112 | 0.812 | 0.293 | 3.74E-14 | 34 | *NME4* | Late RPCs |
| 1.53E-18 | 0.557208 | 0.562 | 0.14 | 3.28E-14 | 34 | *COL9A1* | Late RPCs |
| 2.06E-12 | 0.555295 | 0.938 | 0.584 | 4.41E-08 | 34 | *NASP* | Late RPCs |
| 1.56E-18 | 0.554987 | 1 | 0.997 | 3.35E-14 | 34 | *RPS6* | Late RPCs |
| 8.78E-12 | 0.547326 | 0.75 | 0.324 | 1.88E-07 | 34 | *ID3* | Late RPCs |
| 6.99E-33 | 0.546945 | 0.667 | 0.117 | 1.50E-28 | 34 | *EGFR* | Late RPCs |
| 4.09E-29 | 0.535041 | 0.646 | 0.123 | 8.78E-25 | 34 | *ZIC1* | Late RPCs |
| 1.01E-20 | 0.532504 | 0.562 | 0.125 | 2.18E-16 | 34 | *PLK2* | Late RPCs |
| 1.51E-21 | 0.531822 | 0.75 | 0.206 | 3.25E-17 | 34 | *MCM3* | Late RPCs |
| 2.79E-10 | 0.531033 | 1 | 0.846 | 5.99E-06 | 34 | *EEF1B2* | Late RPCs |
| 7.19E-10 | 0.529952 | 0.667 | 0.292 | 1.54E-05 | 34 | *RDH10* | Late RPCs |
| 2.06E-11 | 0.525965 | 1 | 0.996 | 4.41E-07 | 34 | *MT-CO1* | Late RPCs |
| 1.12E-30 | 0.524351 | 0.5 | 0.071 | 2.40E-26 | 34 | *ATP1A2* | Late RPCs |
| 1.19E-18 | 0.523174 | 0.646 | 0.174 | 2.55E-14 | 34 | *ATAD2* | Late RPCs |
| 1.48E-21 | 0.514312 | 0.604 | 0.14 | 3.17E-17 | 34 | *CENPK* | Late RPCs |
| 6.97E-34 | 0.513452 | 0.771 | 0.146 | 1.49E-29 | 34 | *ORC6* | Late RPCs |
| 8.48E-11 | 0.504371 | 0.896 | 0.528 | 1.82E-06 | 34 | *TSC22D1* | Late RPCs |
| 3.52E-21 | 0.502919 | 0.75 | 0.203 | 7.54E-17 | 34 | *MCM4* | Late RPCs |
| 3.13E-18 | 0.501589 | 1 | 1 | 6.71E-14 | 34 | *RPL41* | Late RPCs |

GGH LRRC172 NPC23 COL9A32 GINS21 DHFR3 IFITM35 MFAP22 COL9A22 NME4 COL9A11 NASP3 RPS61 ID35 EGFR ZIC11 PLK22 MCM31 EEF1B2 RDH101 MT-CO11 ATP1A21 ATAD22 CENPK2 ORC62 TSC22D13 MCM42 RPL411
