# Supplementary material for: pRB-Depleted Pluripotent Stem Cell Retinal Organoids Recapitulate Cell State Transitions of Retinoblastoma Development and Suggest an Important Role for pRB in Retinal Cell Differentiation
Source: Stem Cells Transl Med. 2022 Mar 23;11(4):415–33. doi: 10.1093/stcltm/szac008 (PMC9052432; doi:10.1093/stcltm/szac008)
Supplement: szac008_suppl_Supplementary_Table_S4 [file szac008_suppl_supplementary_table_s4.docx]

|  | **p_val** | **avg_logFC** | **pct.1** | **pct.2** | **p_val_adj** | **cluster** | ***gene*** | **predicted cell fate** |
| --- | --- | --- | --- | --- | --- | --- | --- | --- |
| TRH | 5.78E-251 | 1.993194 | 0.986 | 0.527 | 1.28E-246 | 0 | *TRH* | Proliferating Muller Glia cells |
| TF | 8.04E-97 | 1.831215 | 0.527 | 0.181 | 1.77E-92 | 0 | *TF* | Proliferating Muller Glia cells |
| SFRP2 | 1.32E-211 | 1.761513 | 0.986 | 0.5 | 2.92E-207 | 0 | *SFRP2* | Proliferating Muller Glia cells |
| LAMP5 | 5.60E-284 | 1.678963 | 0.914 | 0.261 | 1.24E-279 | 0 | *LAMP5* | Proliferating Muller Glia cells |
| CRYM | 4.07E-275 | 1.600962 | 0.874 | 0.243 | 8.98E-271 | 0 | *CRYM* | Proliferating Muller Glia cells |
| WIF1 | 5.65E-225 | 1.410774 | 0.691 | 0.147 | 1.25E-220 | 0 | *WIF1* | Proliferating Muller Glia cells |
| AGL | 1.65E-186 | 1.409752 | 0.835 | 0.329 | 3.64E-182 | 0 | *AGL* | Proliferating Muller Glia cells |
| PAX2 | 0 | 1.383656 | 0.747 | 0.102 | 0 | 0 | *PAX2* | Proliferating Muller Glia cells |
| CYP26A1 | 6.95E-135 | 1.354712 | 0.86 | 0.415 | 1.53E-130 | 0 | *CYP26A1* | Proliferating Muller Glia cells |
| NDP | 8.81E-172 | 1.296834 | 0.541 | 0.111 | 1.95E-167 | 0 | *NDP* | Proliferating Muller Glia cells |
| VIM | 1.20E-202 | 1.279108 | 0.998 | 0.781 | 2.66E-198 | 0 | *VIM* | Proliferating Muller Glia cells |
| PTN | 1.15E-153 | 1.239658 | 0.901 | 0.5 | 2.55E-149 | 0 | *PTN* | Proliferating Muller Glia cells |
| GPC3 | 2.25E-161 | 1.230521 | 0.784 | 0.299 | 4.97E-157 | 0 | *GPC3* | Proliferating Muller Glia cells |
| COL18A1 | 4.18E-176 | 1.227129 | 0.782 | 0.283 | 9.23E-172 | 0 | *COL18A1* | Proliferating Muller Glia cells |
| SPP1 | 2.61E-182 | 1.135117 | 0.969 | 0.444 | 5.77E-178 | 0 | *SPP1* | Proliferating Muller Glia cells |
| DKK3 | 6.06E-159 | 1.132776 | 0.899 | 0.444 | 1.34E-154 | 0 | *DKK3* | Proliferating Muller Glia cells |
| GPX3 | 3.17E-91 | 1.097362 | 0.418 | 0.115 | 7.00E-87 | 0 | *GPX3* | Proliferating Muller Glia cells |
| ANGPTL1 | 3.10E-224 | 1.080534 | 0.632 | 0.117 | 6.85E-220 | 0 | *ANGPTL1* | Proliferating Muller Glia cells |
| METRN | 2.80E-106 | 1.067214 | 0.741 | 0.374 | 6.18E-102 | 0 | *METRN* | Proliferating Muller Glia cells |
| SRPRB | 8.83E-87 | 1.06263 | 0.628 | 0.307 | 1.95E-82 | 0 | *SRPRB* | Proliferating Muller Glia cells |
| TTYH1 | 1.56E-170 | 1.031212 | 0.905 | 0.406 | 3.44E-166 | 0 | *TTYH1* | Proliferating Muller Glia cells |
| HES1 | 1.58E-137 | 0.985538 | 0.829 | 0.339 | 3.49E-133 | 0 | *HES1* | Proliferating Muller Glia cells |
| PLP1 | 4.11E-149 | 0.984384 | 0.543 | 0.121 | 9.08E-145 | 0 | *PLP1* | Proliferating Muller Glia cells |
| IFITM2 | 1.85E-93 | 0.9726 | 0.63 | 0.245 | 4.09E-89 | 0 | *IFITM2* | Proliferating Muller Glia cells |
| CRABP1 | 2.35E-133 | 0.943084 | 0.99 | 0.713 | 5.18E-129 | 0 | *CRABP1* | Proliferating Muller Glia cells |
| CLU | 2.80E-154 | 0.941925 | 0.981 | 0.639 | 6.19E-150 | 0 | *CLU* | Proliferating Muller Glia cells |
| GAD2 | 9.32E-117 | 0.925913 | 0.543 | 0.158 | 2.06E-112 | 0 | *GAD2* | Proliferating Muller Glia cells |
| SFRP1 | 1.95E-99 | 0.925155 | 0.498 | 0.153 | 4.31E-95 | 0 | *SFRP1* | Proliferating Muller Glia cells |
| LINC01833 | 4.86E-129 | 0.917069 | 0.763 | 0.337 | 1.07E-124 | 0 | *LINC01833* | Proliferating Muller Glia cells |
| PAX8 | 1.87E-205 | 0.907825 | 0.547 | 0.085 | 4.12E-201 | 0 | *PAX8* | Proliferating Muller Glia cells |
| GSN | 4.78E-115 | 0.880608 | 0.663 | 0.25 | 1.06E-110 | 0 | *GSN* | Proliferating Muller Glia cells |
| CDH6 | 6.80E-107 | 0.871739 | 0.496 | 0.139 | 1.50E-102 | 0 | *CDH6* | Proliferating Muller Glia cells |
| FABP7 | 1.80E-121 | 0.871351 | 0.928 | 0.579 | 3.97E-117 | 0 | *FABP7* | Proliferating Muller Glia cells |
| CYP1B1 | 2.92E-106 | 0.864684 | 0.759 | 0.357 | 6.44E-102 | 0 | *CYP1B1* | Proliferating Muller Glia cells |
| DIO3 | 7.51E-96 | 0.860225 | 0.685 | 0.269 | 1.66E-91 | 0 | *DIO3* | Proliferating Muller Glia cells |
| CD9 | 3.39E-119 | 0.842868 | 0.696 | 0.26 | 7.49E-115 | 0 | *CD9* | Proliferating Muller Glia cells |
| RTN4 | 1.13E-121 | 0.837624 | 0.99 | 0.906 | 2.49E-117 | 0 | *RTN4* | Proliferating Muller Glia cells |
| COL2A1 | 6.86E-105 | 0.825894 | 0.642 | 0.247 | 1.51E-100 | 0 | *COL2A1* | Proliferating Muller Glia cells |
| SOX2 | 1.73E-112 | 0.819828 | 0.815 | 0.366 | 3.81E-108 | 0 | *SOX2* | Proliferating Muller Glia cells |
| SLC1A3 | 1.85E-123 | 0.799535 | 0.465 | 0.107 | 4.08E-119 | 0 | *SLC1A3* | Proliferating Muller Glia cells |
| MOB3B | 6.77E-116 | 0.796203 | 0.615 | 0.205 | 1.49E-111 | 0 | *MOB3B* | Proliferating Muller Glia cells |
| THY1 | 1.18E-105 | 0.789539 | 0.669 | 0.259 | 2.60E-101 | 0 | *THY1* | Proliferating Muller Glia cells |
| ZFP36L1 | 6.70E-89 | 0.769512 | 0.811 | 0.404 | 1.48E-84 | 0 | *ZFP36L1* | Proliferating Muller Glia cells |
| AKAP12 | 4.16E-69 | 0.766676 | 0.685 | 0.378 | 9.19E-65 | 0 | *AKAP12* | Proliferating Muller Glia cells |
| ITGB8 | 3.02E-97 | 0.753633 | 0.681 | 0.293 | 6.66E-93 | 0 | *ITGB8* | Proliferating Muller Glia cells |
| FZD5 | 1.27E-107 | 0.734114 | 0.588 | 0.199 | 2.81E-103 | 0 | *FZD5* | Proliferating Muller Glia cells |
| GAS1 | 6.73E-60 | 0.733566 | 0.374 | 0.13 | 1.49E-55 | 0 | *GAS1* | Proliferating Muller Glia cells |
| SLC15A3 | 9.83E-169 | 0.728814 | 0.346 | 0.035 | 2.17E-164 | 0 | *SLC15A3* | Proliferating Muller Glia cells |
| OAF | 2.10E-76 | 0.720127 | 0.447 | 0.15 | 4.65E-72 | 0 | *OAF* | Proliferating Muller Glia cells |
| DNER | 4.74E-82 | 0.710669 | 0.584 | 0.236 | 1.05E-77 | 0 | *DNER* | Proliferating Muller Glia cells |
| LINC00461 | 2.49E-83 | 0.704789 | 0.617 | 0.254 | 5.51E-79 | 0 | *LINC00461* | Proliferating Muller Glia cells |
| LIMCH1 | 2.46E-82 | 0.702707 | 0.685 | 0.334 | 5.44E-78 | 0 | *LIMCH1* | Proliferating Muller Glia cells |
| MIR7-3HG | 9.14E-43 | -0.72236 | 0.035 | 0.329 | 2.02E-38 | 0 | *MIR7-3HG* | Proliferating Muller Glia cells |
| NEUROG1 | 3.77E-32 | -0.72455 | 0.014 | 0.241 | 8.32E-28 | 0 | *NEUROG1* | Proliferating Muller Glia cells |
| NEUROD4 | 2.23E-43 | -0.73111 | 0.019 | 0.31 | 4.93E-39 | 0 | *NEUROD4* | Proliferating Muller Glia cells |
| UBE2S | 3.93E-34 | -0.73468 | 0.461 | 0.687 | 8.68E-30 | 0 | *UBE2S* | Proliferating Muller Glia cells |
| AKAP9 | 3.47E-23 | -0.74513 | 0.537 | 0.67 | 7.65E-19 | 0 | *AKAP9* | Proliferating Muller Glia cells |
| ENO2 | 6.94E-41 | -0.75102 | 0.442 | 0.667 | 1.53E-36 | 0 | *ENO2* | Proliferating Muller Glia cells |
| IGSF21 | 7.40E-42 | -0.75967 | 0.029 | 0.316 | 1.63E-37 | 0 | *IGSF21* | Proliferating Muller Glia cells |

| STMN1 | 1.96E-67 | -0.7631 | 0.953 | 0.964 | 4.32E-63 | 0 | *STMN1* | Proliferating Muller Glia cells |
| --- | --- | --- | --- | --- | --- | --- | --- | --- |
| CHCHD2 | 9.05E-25 | -0.76796 | 0.249 | 0.456 | 2.00E-20 | 0 | *CHCHD2* | Proliferating Muller Glia cells |
| LINC00599 | 6.97E-43 | -0.76945 | 0.035 | 0.327 | 1.54E-38 | 0 | *LINC00599* | Proliferating Muller Glia cells |
| TUBB4B | 1.86E-41 | -0.77823 | 0.722 | 0.84 | 4.10E-37 | 0 | *TUBB4B* | Proliferating Muller Glia cells |
| DPYSL3 | 1.85E-45 | -0.78285 | 0.062 | 0.37 | 4.08E-41 | 0 | *DPYSL3* | Proliferating Muller Glia cells |
| VXN | 4.45E-32 | -0.79219 | 0.041 | 0.278 | 9.82E-28 | 0 | *VXN* | Proliferating Muller Glia cells |
| RXRG | 1.42E-40 | -0.79545 | 0.074 | 0.362 | 3.13E-36 | 0 | *RXRG* | Proliferating Muller Glia cells |
| MEIS2 | 5.37E-43 | -0.79903 | 0.235 | 0.532 | 1.19E-38 | 0 | *MEIS2* | Proliferating Muller Glia cells |
| TULP1 | 3.05E-42 | -0.82088 | 0.037 | 0.326 | 6.74E-38 | 0 | *TULP1* | Proliferating Muller Glia cells |
| UBE2C | 1.79E-14 | -0.82984 | 0.074 | 0.212 | 3.94E-10 | 0 | *UBE2C* | Proliferating Muller Glia cells |
| TPH1 | 1.49E-31 | -0.83718 | 0.019 | 0.246 | 3.29E-27 | 0 | *TPH1* | Proliferating Muller Glia cells |
| CADPS | 2.36E-51 | -0.8432 | 0.037 | 0.37 | 5.21E-47 | 0 | *CADPS* | Proliferating Muller Glia cells |
| AMER2 | 6.37E-34 | -0.85952 | 0.276 | 0.519 | 1.41E-29 | 0 | *AMER2* | Proliferating Muller Glia cells |
| MAP1B | 4.07E-83 | -0.86166 | 0.679 | 0.873 | 9.00E-79 | 0 | *MAP1B* | Proliferating Muller Glia cells |
| SYP | 2.09E-55 | -0.88689 | 0.066 | 0.419 | 4.61E-51 | 0 | *SYP* | Proliferating Muller Glia cells |
| CRX | 2.87E-43 | -0.88946 | 0.056 | 0.353 | 6.33E-39 | 0 | *CRX* | Proliferating Muller Glia cells |
| Sep-04 | 2.08E-46 | -0.91381 | 0.107 | 0.425 | 4.59E-42 | 0 | *Sep-04* | Proliferating Muller Glia cells |
| OTX2 | 1.06E-50 | -0.91414 | 0.054 | 0.387 | 2.33E-46 | 0 | *OTX2* | Proliferating Muller Glia cells |
| GADD45G | 1.23E-52 | -0.92138 | 0.158 | 0.5 | 2.71E-48 | 0 | *GADD45G* | Proliferating Muller Glia cells |
| SLC38A5 | 3.93E-38 | -0.9236 | 0.078 | 0.353 | 8.67E-34 | 0 | *SLC38A5* | Proliferating Muller Glia cells |
| BASP1 | 1.04E-53 | -0.92611 | 0.496 | 0.747 | 2.29E-49 | 0 | *BASP1* | Proliferating Muller Glia cells |
| ATP1A3 | 4.71E-55 | -0.92977 | 0.031 | 0.38 | 1.04E-50 | 0 | *ATP1A3* | Proliferating Muller Glia cells |
| HMGB2 | 2.37E-33 | -0.93103 | 0.578 | 0.747 | 5.23E-29 | 0 | *HMGB2* | Proliferating Muller Glia cells |
| HMGA1 | 1.17E-57 | -0.95455 | 0.377 | 0.685 | 2.59E-53 | 0 | *HMGA1* | Proliferating Muller Glia cells |
| UNC119 | 1.71E-28 | -0.95934 | 0.337 | 0.54 | 3.77E-24 | 0 | *UNC119* | Proliferating Muller Glia cells |
| SOX4 | 4.75E-56 | -0.96056 | 0.7 | 0.882 | 1.05E-51 | 0 | *SOX4* | Proliferating Muller Glia cells |
| CENPF | 3.26E-20 | -0.98081 | 0.13 | 0.319 | 7.20E-16 | 0 | *CENPF* | Proliferating Muller Glia cells |
| TOP2A | 1.30E-21 | -0.98622 | 0.103 | 0.296 | 2.88E-17 | 0 | *TOP2A* | Proliferating Muller Glia cells |
| RRAD | 8.39E-29 | -1.00245 | 0.107 | 0.341 | 1.85E-24 | 0 | *RRAD* | Proliferating Muller Glia cells |
| PCBP4 | 3.19E-48 | -1.00438 | 0.253 | 0.543 | 7.05E-44 | 0 | *PCBP4* | Proliferating Muller Glia cells |
| GADD45A | 4.24E-49 | -1.06398 | 0.228 | 0.559 | 9.37E-45 | 0 | *GADD45A* | Proliferating Muller Glia cells |
| DCT | 1.14E-41 | -1.11214 | 0.031 | 0.318 | 2.51E-37 | 0 | *DCT* | Proliferating Muller Glia cells |
| FAM57B | 1.63E-64 | -1.17316 | 0.115 | 0.497 | 3.59E-60 | 0 | *FAM57B* | Proliferating Muller Glia cells |
| GNB3 | 7.49E-47 | -1.19943 | 0.138 | 0.449 | 1.65E-42 | 0 | *GNB3* | Proliferating Muller Glia cells |
| AIPL1 | 3.50E-43 | -1.20179 | 0.064 | 0.359 | 7.72E-39 | 0 | *AIPL1* | Proliferating Muller Glia cells |
| PDE6H | 1.40E-27 | -1.27659 | 0.181 | 0.399 | 3.08E-23 | 0 | *PDE6H* | Proliferating Muller Glia cells |
| CPE | 9.46E-89 | -1.28337 | 0.307 | 0.702 | 2.09E-84 | 0 | *CPE* | Proliferating Muller Glia cells |
| NEUROD1 | 2.77E-75 | -1.33504 | 0.043 | 0.474 | 6.11E-71 | 0 | *NEUROD1* | Proliferating Muller Glia cells |
| STMN2 | 1.37E-53 | -1.35908 | 0.076 | 0.424 | 3.02E-49 | 0 | *STMN2* | Proliferating Muller Glia cells |
| SCG3 | 8.47E-65 | -1.36185 | 0.204 | 0.561 | 1.87E-60 | 0 | *SCG3* | Proliferating Muller Glia cells |
| HES6 | 8.42E-69 | -1.40804 | 0.364 | 0.692 | 1.86E-64 | 0 | *HES6* | Proliferating Muller Glia cells |
| NRL | 8.68E-38 | -1.52103 | 0.095 | 0.368 | 1.92E-33 | 0 | *NRL* | Proliferating Muller Glia cells |
| PDC | 3.63E-35 | -1.52985 | 0.202 | 0.447 | 8.01E-31 | 0 | *PDC* | Proliferating Muller Glia cells |
| HIST1H4C | 8.81E-35 | -1.6083 | 0.35 | 0.612 | 1.95E-30 | 0 | *HIST1H4C* | Proliferating Muller Glia cells |
| RCVRN | 3.26E-45 | -1.80197 | 0.103 | 0.406 | 7.20E-41 | 0 | *RCVRN* | Proliferating Muller Glia cells |
| SPP1.1 | 9.30E-169 | 1.144688 | 0.982 | 0.451 | 2.05E-164 | 1 | *SPP1* | Late RPCs |
| IFITM2.1 | 1.59E-112 | 1.132951 | 0.699 | 0.244 | 3.51E-108 | 1 | *IFITM2* | Late RPCs |
| LGALS3 | 2.36E-88 | 1.124525 | 0.532 | 0.168 | 5.20E-84 | 1 | *LGALS3* | Late RPCs |
| TTYH1.1 | 3.51E-136 | 1.037014 | 0.89 | 0.415 | 7.76E-132 | 1 | *TTYH1* | Late RPCs |
| DIO3.1 | 2.99E-112 | 1.021687 | 0.747 | 0.27 | 6.60E-108 | 1 | *DIO3* | Late RPCs |
| IFITM3 | 4.31E-111 | 0.992633 | 0.788 | 0.316 | 9.52E-107 | 1 | *IFITM3* | Late RPCs |
| BAALC | 1.29E-105 | 0.913438 | 0.737 | 0.302 | 2.86E-101 | 1 | *BAALC* | Late RPCs |
| ZFP36L1.1 | 1.10E-110 | 0.897935 | 0.886 | 0.404 | 2.44E-106 | 1 | *ZFP36L1* | Late RPCs |
| ZFP36L2 | 1.21E-91 | 0.874756 | 0.815 | 0.413 | 2.67E-87 | 1 | *ZFP36L2* | Late RPCs |
| QDPR | 3.50E-39 | 0.864578 | 0.582 | 0.367 | 7.73E-35 | 1 | *QDPR* | Late RPCs |
| GPM6B | 6.36E-92 | 0.862348 | 0.895 | 0.634 | 1.40E-87 | 1 | *GPM6B* | Late RPCs |
| MGARP | 1.48E-107 | 0.855733 | 0.685 | 0.248 | 3.26E-103 | 1 | *MGARP* | Late RPCs |
| DKK3.1 | 2.10E-87 | 0.810768 | 0.852 | 0.455 | 4.64E-83 | 1 | *DKK3* | Late RPCs |
| VIM.1 | 7.20E-110 | 0.79378 | 1 | 0.785 | 1.59E-105 | 1 | *VIM* | Late RPCs |
| CYP26A1.1 | 2.49E-71 | 0.791631 | 0.808 | 0.426 | 5.51E-67 | 1 | *CYP26A1* | Late RPCs |

| SOX2.1 | 7.16E-101 | 0.760478 | 0.849 | 0.37 | 1.58E-96 | 1 | *SOX2* | Late RPCs |
| --- | --- | --- | --- | --- | --- | --- | --- | --- |
| PLEKHA1 | 1.28E-74 | 0.755749 | 0.877 | 0.6 | 2.83E-70 | 1 | *PLEKHA1* | Late RPCs |
| PTH2 | 1.41E-80 | 0.751759 | 0.868 | 0.492 | 3.12E-76 | 1 | *PTH2* | Late RPCs |
| DAPL1 | 5.60E-73 | 0.751338 | 0.838 | 0.5 | 1.24E-68 | 1 | *DAPL1* | Late RPCs |
| PTPRZ1 | 4.90E-87 | 0.749839 | 0.694 | 0.281 | 1.08E-82 | 1 | *PTPRZ1* | Late RPCs |
| PRSS23 | 7.79E-78 | 0.74907 | 0.808 | 0.414 | 1.72E-73 | 1 | *PRSS23* | Late RPCs |
| RTN4.1 | 2.11E-115 | 0.747097 | 0.998 | 0.907 | 4.66E-111 | 1 | *RTN4* | Late RPCs |
| CLU.1 | 4.59E-106 | 0.72882 | 0.979 | 0.644 | 1.01E-101 | 1 | *CLU* | Late RPCs |
| MDK | 1.78E-82 | 0.722152 | 0.966 | 0.698 | 3.92E-78 | 1 | *MDK* | Late RPCs |
| PLP1.1 | 2.93E-80 | 0.719082 | 0.47 | 0.134 | 6.46E-76 | 1 | *PLP1* | Late RPCs |
| TF.1 | 1.90E-39 | 0.717466 | 0.441 | 0.194 | 4.20E-35 | 1 | *TF* | Late RPCs |
| DCBLD2 | 1.65E-65 | 0.716253 | 0.733 | 0.424 | 3.63E-61 | 1 | *DCBLD2* | Late RPCs |
| HMGB2.1 | 5.01E-09 | -0.706 | 0.717 | 0.732 | 0.000111 | 1 | *HMGB2* | Late RPCs |
| NEUROD4. | 1.25E-35 | -0.70714 | 0.023 | 0.306 | 2.77E-31 | 1 | *NEUROD4* | Late RPCs |
| MIR7-3HG. | 1.31E-34 | -0.70802 | 0.043 | 0.324 | 2.90E-30 | 1 | *MIR7-3HG* | Late RPCs |
| SOX4.1 | 1.37E-19 | -0.7174 | 0.813 | 0.869 | 3.03E-15 | 1 | *SOX4* | Late RPCs |
| MAP1LC3A | 9.62E-33 | -0.72228 | 0.235 | 0.502 | 2.12E-28 | 1 | *MAP1LC3A* | Late RPCs |
| OLFM1 | 8.95E-39 | -0.72394 | 0.05 | 0.359 | 1.98E-34 | 1 | *OLFM1* | Late RPCs |
| PTTG1 | 5.64E-15 | -0.72467 | 0.24 | 0.416 | 1.25E-10 | 1 | *PTTG1* | Late RPCs |
| TUBA4A | 2.67E-29 | -0.72912 | 0.144 | 0.406 | 5.90E-25 | 1 | *TUBA4A* | Late RPCs |
| VXN.1 | 1.95E-22 | -0.73401 | 0.064 | 0.272 | 4.31E-18 | 1 | *VXN* | Late RPCs |
| NUSAP1 | 1.29E-17 | -0.73571 | 0.162 | 0.35 | 2.84E-13 | 1 | *NUSAP1* | Late RPCs |
| COL1A1 | 0.001052 | -0.7394 | 0.132 | 0.085 | 1 | 1 | *COL1A1* | Late RPCs |
| FSTL5 | 1.07E-34 | -0.73942 | 0.032 | 0.308 | 2.36E-30 | 1 | *FSTL5* | Late RPCs |
| CRMP1 | 1.36E-42 | -0.7488 | 0.126 | 0.454 | 3.01E-38 | 1 | *CRMP1* | Late RPCs |
| IGSF21.1 | 2.03E-34 | -0.7502 | 0.034 | 0.311 | 4.48E-30 | 1 | *IGSF21* | Late RPCs |
| LINC00599 | 5.87E-35 | -0.75242 | 0.041 | 0.322 | 1.30E-30 | 1 | *LINC00599* | Late RPCs |
| MAP2 | 1.65E-29 | -0.76623 | 0.32 | 0.557 | 3.65E-25 | 1 | *MAP2* | Late RPCs |
| UBE2S.1 | 3.03E-27 | -0.76687 | 0.498 | 0.681 | 6.69E-23 | 1 | *UBE2S* | Late RPCs |
| TUBB4B.1 | 1.82E-35 | -0.77627 | 0.724 | 0.838 | 4.01E-31 | 1 | *TUBB4B* | Late RPCs |
| GADD45A. | 2.31E-18 | -0.77967 | 0.349 | 0.544 | 5.11E-14 | 1 | *GADD45A* | Late RPCs |
| SLC38A1 | 1.78E-43 | -0.78267 | 0.187 | 0.516 | 3.92E-39 | 1 | *SLC38A1* | Late RPCs |
| HMGA1.1 | 9.47E-29 | -0.78979 | 0.491 | 0.67 | 2.09E-24 | 1 | *HMGA1* | Late RPCs |
| SEZ6L2 | 1.37E-48 | -0.79183 | 0.089 | 0.447 | 3.03E-44 | 1 | *SEZ6L2* | Late RPCs |
| TULP1.1 | 1.14E-34 | -0.79784 | 0.041 | 0.322 | 2.51E-30 | 1 | *TULP1* | Late RPCs |
| MLLT11 | 1.92E-45 | -0.79802 | 0.459 | 0.71 | 4.24E-41 | 1 | *MLLT11* | Late RPCs |
| RXRG.1 | 2.42E-35 | -0.80077 | 0.071 | 0.358 | 5.35E-31 | 1 | *RXRG* | Late RPCs |
| DPYSL3.1 | 1.12E-40 | -0.80483 | 0.053 | 0.367 | 2.47E-36 | 1 | *DPYSL3* | Late RPCs |
| SEPT4.1 | 6.68E-28 | -0.81088 | 0.174 | 0.414 | 1.48E-23 | 1 | *Sep-04* | Late RPCs |
| BASP1.1 | 7.08E-34 | -0.81626 | 0.557 | 0.738 | 1.56E-29 | 1 | *BASP1* | Late RPCs |
| CHCHD2.1 | 3.62E-27 | -0.81883 | 0.196 | 0.457 | 7.98E-23 | 1 | *CHCHD2* | Late RPCs |
| HES6.1 | 1.70E-27 | -0.81969 | 0.454 | 0.679 | 3.75E-23 | 1 | *HES6* | Late RPCs |
| UBE2C.1 | 9.94E-13 | -0.82333 | 0.071 | 0.21 | 2.19E-08 | 1 | *UBE2C* | Late RPCs |
| MEIS2.1 | 1.61E-36 | -0.82541 | 0.242 | 0.526 | 3.55E-32 | 1 | *MEIS2* | Late RPCs |
| CD24 | 5.10E-30 | -0.82835 | 0.205 | 0.475 | 1.13E-25 | 1 | *CD24* | Late RPCs |
| TPH1.1 | 3.33E-25 | -0.83579 | 0.027 | 0.242 | 7.34E-21 | 1 | *TPH1* | Late RPCs |
| CADPS.1 | 1.54E-43 | -0.84072 | 0.037 | 0.365 | 3.39E-39 | 1 | *CADPS* | Late RPCs |
| AKAP9.1 | 1.94E-28 | -0.84195 | 0.479 | 0.673 | 4.28E-24 | 1 | *AKAP9* | Late RPCs |
| CRX.1 | 1.06E-35 | -0.89382 | 0.064 | 0.348 | 2.35E-31 | 1 | *CRX* | Late RPCs |
| ENO2.1 | 1.25E-47 | -0.89404 | 0.4 | 0.667 | 2.77E-43 | 1 | *ENO2* | Late RPCs |
| GADD45G. | 1.97E-47 | -0.89598 | 0.137 | 0.496 | 4.36E-43 | 1 | *GADD45G* | Late RPCs |
| ATP1A3.1 | 5.14E-46 | -0.92299 | 0.034 | 0.374 | 1.13E-41 | 1 | *ATP1A3* | Late RPCs |
| CENPF.1 | 3.48E-17 | -0.94444 | 0.13 | 0.316 | 7.69E-13 | 1 | *CENPF* | Late RPCs |
| OTX2.1 | 3.37E-47 | -0.94653 | 0.034 | 0.384 | 7.44E-43 | 1 | *OTX2* | Late RPCs |
| TOP2A.1 | 2.24E-16 | -0.95741 | 0.119 | 0.292 | 4.94E-12 | 1 | *TOP2A* | Late RPCs |
| SYP.1 | 1.02E-55 | -0.96085 | 0.03 | 0.417 | 2.26E-51 | 1 | *SYP* | Late RPCs |
| SLC38A5.1 | 7.61E-40 | -0.99873 | 0.043 | 0.352 | 1.68E-35 | 1 | *SLC38A5* | Late RPCs |
| UNC119.1 | 8.18E-29 | -1.00346 | 0.301 | 0.54 | 1.81E-24 | 1 | *UNC119* | Late RPCs |
| RRAD.1 | 3.31E-27 | -1.04831 | 0.096 | 0.338 | 7.31E-23 | 1 | *RRAD* | Late RPCs |
| PCBP4.1 | 1.76E-47 | -1.05332 | 0.215 | 0.542 | 3.89E-43 | 1 | *PCBP4* | Late RPCs |

| AMER2.1 | 3.64E-49 | -1.05599 | 0.176 | 0.524 | 8.05E-45 | 1 | *AMER2* | Late RPCs |
| --- | --- | --- | --- | --- | --- | --- | --- | --- |
| CPE.1 | 4.43E-56 | -1.10544 | 0.386 | 0.689 | 9.78E-52 | 1 | *CPE* | Late RPCs |
| DCT.1 | 1.88E-34 | -1.11918 | 0.037 | 0.313 | 4.15E-30 | 1 | *DCT* | Late RPCs |
| FAM57B.1 | 2.95E-55 | -1.18254 | 0.112 | 0.491 | 6.51E-51 | 1 | *FAM57B* | Late RPCs |
| PDE6H.1 | 1.27E-22 | -1.2551 | 0.187 | 0.395 | 2.81E-18 | 1 | *PDE6H* | Late RPCs |
| NEUROD1. | 1.14E-52 | -1.25665 | 0.1 | 0.462 | 2.52E-48 | 1 | *NEUROD1* | Late RPCs |
| GNB3.1 | 6.98E-42 | -1.25905 | 0.132 | 0.445 | 1.54E-37 | 1 | *GNB3* | Late RPCs |
| AIPL1.1 | 2.47E-40 | -1.26326 | 0.048 | 0.356 | 5.46E-36 | 1 | *AIPL1* | Late RPCs |
| STMN2.1 | 1.55E-41 | -1.31651 | 0.096 | 0.417 | 3.43E-37 | 1 | *STMN2* | Late RPCs |
| NRL.1 | 1.88E-25 | -1.4304 | 0.13 | 0.361 | 4.15E-21 | 1 | *NRL* | Late RPCs |
| HIST1H4C. | 5.01E-21 | -1.536 | 0.4 | 0.603 | 1.11E-16 | 1 | *HIST1H4C* | Late RPCs |
| SCG3.1 | 1.61E-72 | -1.58194 | 0.126 | 0.563 | 3.55E-68 | 1 | *SCG3* | Late RPCs |
| PDC.1 | 1.67E-31 | -1.60929 | 0.199 | 0.444 | 3.68E-27 | 1 | *PDC* | Late RPCs |
| RCVRN.1 | 2.08E-32 | -1.69367 | 0.132 | 0.399 | 4.60E-28 | 1 | *RCVRN* | Late RPCs |
| NRL.2 | 1.33E-214 | 2.359032 | 0.939 | 0.301 | 2.93E-210 | 2 | *NRL* | Rod precursors |
| ROM1 | 1.25E-248 | 1.965887 | 0.858 | 0.175 | 2.77E-244 | 2 | *ROM1* | Rod precursors |
| RCVRN.2 | 3.52E-162 | 1.960194 | 0.919 | 0.34 | 7.78E-158 | 2 | *RCVRN* | Rod precursors |
| PDC.2 | 2.10E-213 | 1.881425 | 0.991 | 0.385 | 4.64E-209 | 2 | *PDC* | Rod precursors |
| UNC119.2 | 2.60E-178 | 1.680785 | 0.98 | 0.489 | 5.75E-174 | 2 | *UNC119* | Rod precursors |
| NR2E3 | 4.54E-255 | 1.572237 | 0.733 | 0.105 | 1.00E-250 | 2 | *NR2E3* | Rod precursors |
| GNAT1 | 1.76E-261 | 1.503533 | 0.493 | 0.034 | 3.88E-257 | 2 | *GNAT1* | Rod precursors |
| AIPL1.2 | 5.52E-163 | 1.419499 | 0.922 | 0.29 | 1.22E-158 | 2 | *AIPL1* | Rod precursors |
| GNB3.2 | 1.64E-177 | 1.417505 | 0.977 | 0.382 | 3.62E-173 | 2 | *GNB3* | Rod precursors |
| PIK3R1 | 7.06E-116 | 1.298055 | 0.884 | 0.462 | 1.56E-111 | 2 | *PIK3R1* | Rod precursors |
| GNGT2 | 3.19E-177 | 1.235889 | 0.728 | 0.15 | 7.05E-173 | 2 | *GNGT2* | Rod precursors |
| CRX.2 | 3.18E-168 | 1.187564 | 0.913 | 0.285 | 7.02E-164 | 2 | *CRX* | Rod precursors |
| SLC38A5.2 | 3.30E-148 | 1.181678 | 0.878 | 0.289 | 7.29E-144 | 2 | *SLC38A5* | Rod precursors |
| SEPT4.2 | 1.06E-138 | 1.166083 | 0.896 | 0.36 | 2.34E-134 | 2 | *Sep-04* | Rod precursors |
| RRAD.2 | 2.77E-66 | 1.16527 | 0.672 | 0.294 | 6.12E-62 | 2 | *RRAD* | Rod precursors |
| NEUROG1. | 4.23E-105 | 1.10221 | 0.649 | 0.19 | 9.33E-101 | 2 | *NEUROG1* | Rod precursors |
| CABP5 | 1.53E-210 | 1.078945 | 0.388 | 0.025 | 3.39E-206 | 2 | *CABP5* | Rod precursors |
| RP1 | 1.26E-150 | 1.056222 | 0.562 | 0.1 | 2.77E-146 | 2 | *RP1* | Rod precursors |
| PCBP4.2 | 2.13E-121 | 1.049518 | 0.945 | 0.486 | 4.70E-117 | 2 | *PCBP4* | Rod precursors |
| NEUROD1. | 9.14E-113 | 1.046609 | 0.901 | 0.401 | 2.02E-108 | 2 | *NEUROD1* | Rod precursors |
| CPLX4 | 4.81E-167 | 1.042111 | 0.638 | 0.117 | 1.06E-162 | 2 | *CPLX4* | Rod precursors |
| FAM57B.2 | 4.14E-120 | 1.034239 | 0.939 | 0.428 | 9.14E-116 | 2 | *FAM57B* | Rod precursors |
| IMPG2 | 7.81E-151 | 1.020923 | 0.783 | 0.203 | 1.73E-146 | 2 | *IMPG2* | Rod precursors |
| CPE.2 | 1.13E-99 | 0.994832 | 0.954 | 0.645 | 2.48E-95 | 2 | *CPE* | Rod precursors |
| MAP2.1 | 3.82E-84 | 0.990863 | 0.896 | 0.514 | 8.43E-80 | 2 | *MAP2* | Rod precursors |
| LINC00599 | 1.73E-132 | 0.984127 | 0.823 | 0.263 | 3.82E-128 | 2 | *LINC00599* | Rod precursors |
| TUBB4B.2 | 5.77E-92 | 0.956883 | 0.98 | 0.818 | 1.27E-87 | 2 | *TUBB4B* | Rod precursors |
| PDE6H.2 | 5.98E-58 | 0.934877 | 0.742 | 0.354 | 1.32E-53 | 2 | *PDE6H* | Rod precursors |
| KCNV2 | 1.59E-113 | 0.932827 | 0.661 | 0.17 | 3.50E-109 | 2 | *KCNV2* | Rod precursors |
| SYP.2 | 6.07E-107 | 0.914437 | 0.858 | 0.354 | 1.34E-102 | 2 | *SYP* | Rod precursors |
| RBP3 | 3.00E-128 | 0.909354 | 0.675 | 0.166 | 6.62E-124 | 2 | *RBP3* | Rod precursors |
| SLC1A7 | 2.13E-135 | 0.90515 | 0.699 | 0.178 | 4.71E-131 | 2 | *SLC1A7* | Rod precursors |
| PTP4A3 | 8.04E-88 | 0.901076 | 0.841 | 0.427 | 1.78E-83 | 2 | *PTP4A3* | Rod precursors |
| PTPN13 | 6.81E-86 | 0.898476 | 0.684 | 0.259 | 1.50E-81 | 2 | *PTPN13* | Rod precursors |
| MYO9A | 3.26E-68 | 0.89568 | 0.641 | 0.272 | 7.20E-64 | 2 | *MYO9A* | Rod precursors |
| SCG3.2 | 5.32E-91 | 0.888327 | 0.936 | 0.499 | 1.17E-86 | 2 | *SCG3* | Rod precursors |
| MPP4 | 3.07E-111 | 0.874202 | 0.672 | 0.179 | 6.78E-107 | 2 | *MPP4* | Rod precursors |
| ATP1A3.2 | 3.47E-90 | 0.87355 | 0.783 | 0.317 | 7.66E-86 | 2 | *ATP1A3* | Rod precursors |
| FSTL5.1 | 2.62E-103 | 0.867327 | 0.757 | 0.253 | 5.78E-99 | 2 | *FSTL5* | Rod precursors |
| TULP1.2 | 1.02E-111 | 0.862098 | 0.806 | 0.264 | 2.26E-107 | 2 | *TULP1* | Rod precursors |
| EPS8 | 2.32E-74 | 0.850719 | 0.69 | 0.3 | 5.13E-70 | 2 | *EPS8* | Rod precursors |
| OTX2.2 | 3.93E-101 | 0.850319 | 0.838 | 0.322 | 8.67E-97 | 2 | *OTX2* | Rod precursors |
| ALDOC | 1.22E-78 | 0.841698 | 0.539 | 0.167 | 2.69E-74 | 2 | *ALDOC* | Rod precursors |
| TUBA4A.1 | 1.54E-78 | 0.840238 | 0.788 | 0.357 | 3.40E-74 | 2 | *TUBA4A* | Rod precursors |
| GUK1 | 7.86E-97 | 0.839733 | 0.977 | 0.768 | 1.74E-92 | 2 | *GUK1* | Rod precursors |
| AANAT | 9.93E-103 | 0.837081 | 0.678 | 0.205 | 2.19E-98 | 2 | *AANAT* | Rod precursors |

| AMER2.2 | 3.79E-65 | 0.824158 | 0.814 | 0.474 | 8.38E-61 | 2 | *AMER2* | Rod precursors |
| --- | --- | --- | --- | --- | --- | --- | --- | --- |
| PLEKHB1 | 2.00E-76 | 0.819102 | 0.736 | 0.309 | 4.41E-72 | 2 | *PLEKHB1* | Rod precursors |
| GUCA1A | 2.48E-22 | 0.802458 | 0.368 | 0.181 | 5.48E-18 | 2 | *GUCA1A* | Rod precursors |
| REEP6 | 6.51E-104 | 0.798568 | 0.559 | 0.143 | 1.44E-99 | 2 | *REEP6* | Rod precursors |
| PKM | 2.73E-82 | 0.794862 | 0.971 | 0.87 | 6.03E-78 | 2 | *PKM* | Rod precursors |
| STX3 | 1.65E-100 | 0.793192 | 0.745 | 0.27 | 3.65E-96 | 2 | *STX3* | Rod precursors |
| AKAP9.2 | 1.15E-70 | 0.784537 | 0.928 | 0.639 | 2.54E-66 | 2 | *AKAP9* | Rod precursors |
| RXRG.2 | 1.50E-74 | 0.782237 | 0.736 | 0.307 | 3.32E-70 | 2 | *RXRG* | Rod precursors |
| NEUROD4. | 3.99E-88 | 0.78139 | 0.713 | 0.253 | 8.82E-84 | 2 | *NEUROD4* | Rod precursors |
| PRDX1 | 4.36E-82 | 0.780502 | 0.968 | 0.813 | 9.63E-78 | 2 | *PRDX1* | Rod precursors |
| EPB41L2 | 3.52E-43 | 0.773914 | 0.591 | 0.298 | 7.76E-39 | 2 | *EPB41L2* | Rod precursors |
| MAP1LC3A | 1.09E-72 | 0.772327 | 0.838 | 0.456 | 2.40E-68 | 2 | *MAP1LC3A* | Rod precursors |
| AL451062. | 5.17E-85 | 0.771658 | 0.591 | 0.178 | 1.14E-80 | 2 | *AL451062.* | Rod precursors |
| ENO2.2 | 6.39E-70 | 0.767897 | 0.907 | 0.628 | 1.41E-65 | 2 | *ENO2* | Rod precursors |
| EYS | 5.48E-114 | 0.766917 | 0.565 | 0.13 | 1.21E-109 | 2 | *EYS* | Rod precursors |
| ATP1B2 | 4.18E-64 | 0.761539 | 0.817 | 0.479 | 9.23E-60 | 2 | *ATP1B2* | Rod precursors |
| SLC17A7 | 8.26E-107 | 0.757983 | 0.693 | 0.207 | 1.82E-102 | 2 | *SLC17A7* | Rod precursors |
| CADPS.2 | 3.24E-72 | 0.757943 | 0.736 | 0.311 | 7.15E-68 | 2 | *CADPS* | Rod precursors |
| CHRNA3 | 3.11E-62 | 0.755267 | 0.548 | 0.201 | 6.86E-58 | 2 | *CHRNA3* | Rod precursors |
| MAP1LC3B | 5.55E-62 | 0.752551 | 0.855 | 0.574 | 1.23E-57 | 2 | *MAP1LC3B* | Rod precursors |
| RAX2 | 9.50E-140 | 0.752263 | 0.406 | 0.051 | 2.10E-135 | 2 | *RAX2* | Rod precursors |
| COBLL1 | 6.46E-77 | 0.74487 | 0.646 | 0.236 | 1.43E-72 | 2 | *COBLL1* | Rod precursors |
| GPR160 | 4.88E-78 | 0.742566 | 0.328 | 0.061 | 1.08E-73 | 2 | *GPR160* | Rod precursors |
| PPFIA2 | 1.39E-90 | 0.74193 | 0.623 | 0.197 | 3.07E-86 | 2 | *PPFIA2* | Rod precursors |
| FAM19A4 | 2.60E-93 | 0.734196 | 0.548 | 0.143 | 5.74E-89 | 2 | *FAM19A4* | Rod precursors |
| PRMT1 | 7.69E-77 | 0.733296 | 0.922 | 0.711 | 1.70E-72 | 2 | *PRMT1* | Rod precursors |
| MAK | 2.89E-103 | 0.730538 | 0.559 | 0.136 | 6.37E-99 | 2 | *MAK* | Rod precursors |
| ZNF385A | 3.21E-66 | 0.719873 | 0.768 | 0.396 | 7.09E-62 | 2 | *ZNF385A* | Rod precursors |
| ABHD14A | 2.59E-67 | 0.719689 | 0.739 | 0.361 | 5.71E-63 | 2 | *ABHD14A* | Rod precursors |
| PRNP | 1.76E-68 | 0.717163 | 0.786 | 0.416 | 3.88E-64 | 2 | *PRNP* | Rod precursors |
| GUCY1A2 | 2.40E-55 | 0.716329 | 0.484 | 0.175 | 5.29E-51 | 2 | *GUCY1A2* | Rod precursors |
| SAG | 6.19E-50 | 0.70659 | 0.119 | 0.011 | 1.37E-45 | 2 | *SAG* | Rod precursors |
| ANKRD33B | 5.17E-83 | 0.704794 | 0.614 | 0.201 | 1.14E-78 | 2 | *ANKRD33B* | Rod precursors |
| SOX11 | 1.11E-15 | -0.70308 | 0.423 | 0.595 | 2.45E-11 | 2 | *SOX11* | Rod precursors |
| PTGDS | 2.01E-10 | -0.7031 | 0.067 | 0.201 | 4.43E-06 | 2 | *PTGDS* | Rod precursors |
| FABP5 | 4.99E-30 | -0.72631 | 0.316 | 0.588 | 1.10E-25 | 2 | *FABP5* | Rod precursors |
| COL18A1.1 | 1.59E-25 | -0.7276 | 0.081 | 0.348 | 3.51E-21 | 2 | *COL18A1* | Rod precursors |
| METRN.1 | 7.94E-19 | -0.74823 | 0.206 | 0.423 | 1.75E-14 | 2 | *METRN* | Rod precursors |
| PAX6 | 9.41E-29 | -0.75848 | 0.13 | 0.428 | 2.08E-24 | 2 | *PAX6* | Rod precursors |
| SAT1 | 3.60E-24 | -0.7603 | 0.38 | 0.595 | 7.96E-20 | 2 | *SAT1* | Rod precursors |
| AKAP12.1 | 2.43E-33 | -0.76155 | 0.099 | 0.428 | 5.37E-29 | 2 | *AKAP12* | Rod precursors |
| RGS16 | 3.11E-26 | -0.77411 | 0.354 | 0.618 | 6.88E-22 | 2 | *RGS16* | Rod precursors |
| NUSAP1.1 | 7.99E-17 | -0.79177 | 0.148 | 0.347 | 1.77E-12 | 2 | *NUSAP1* | Rod precursors |
| PTTG1.1 | 3.50E-13 | -0.79874 | 0.241 | 0.413 | 7.73E-09 | 2 | *PTTG1* | Rod precursors |
| CYP1B1.1 | 4.41E-21 | -0.81393 | 0.171 | 0.411 | 9.73E-17 | 2 | *CYP1B1* | Rod precursors |
| DHRS3 | 2.28E-34 | -0.82142 | 0.194 | 0.506 | 5.04E-30 | 2 | *DHRS3* | Rod precursors |
| PMAIP1 | 9.50E-24 | -0.82387 | 0.191 | 0.442 | 2.10E-19 | 2 | *PMAIP1* | Rod precursors |
| HIST1H4C. | 1.16E-10 | -0.82399 | 0.739 | 0.576 | 2.56E-06 | 2 | *HIST1H4C* | Rod precursors |
| PTH2.1 | 7.26E-30 | -0.83343 | 0.246 | 0.541 | 1.60E-25 | 2 | *PTH2* | Rod precursors |
| IFITM2.2 | 7.00E-21 | -0.84618 | 0.064 | 0.296 | 1.55E-16 | 2 | *IFITM2* | Rod precursors |
| TYMS | 6.13E-30 | -0.85179 | 0.417 | 0.638 | 1.35E-25 | 2 | *TYMS* | Rod precursors |
| DIO3.2 | 1.51E-23 | -0.86501 | 0.07 | 0.325 | 3.32E-19 | 2 | *DIO3* | Rod precursors |
| ID3 | 8.34E-19 | -0.86884 | 0.261 | 0.479 | 1.84E-14 | 2 | *ID3* | Rod precursors |
| TF.2 | 6.52E-06 | -0.8702 | 0.125 | 0.22 | 0.144054 | 2 | *TF* | Rod precursors |
| MEST | 7.50E-39 | -0.87813 | 0.2 | 0.537 | 1.66E-34 | 2 | *MEST* | Rod precursors |
| HMGB2.2 | 1.52E-17 | -0.88292 | 0.646 | 0.736 | 3.35E-13 | 2 | *HMGB2* | Rod precursors |
| UBE2C.2 | 9.30E-14 | -0.88975 | 0.046 | 0.209 | 2.05E-09 | 2 | *UBE2C* | Rod precursors |
| DAPL1.1 | 1.40E-24 | -0.94042 | 0.304 | 0.542 | 3.10E-20 | 2 | *DAPL1* | Rod precursors |
| GPC3.1 | 5.26E-25 | -0.94448 | 0.099 | 0.362 | 1.16E-20 | 2 | *GPC3* | Rod precursors |
| IFITM3.1 | 3.37E-26 | -0.94945 | 0.099 | 0.372 | 7.44E-22 | 2 | *IFITM3* | Rod precursors |

| PRSS23.1 | 4.25E-38 | -0.96276 | 0.113 | 0.469 | 9.38E-34 | 2 | *PRSS23* | Rod precursors |
| --- | --- | --- | --- | --- | --- | --- | --- | --- |
| HES1.1 | 1.45E-34 | -0.97803 | 0.078 | 0.407 | 3.20E-30 | 2 | *HES1* | Rod precursors |
| TOP2A.2 | 1.85E-16 | -1.00591 | 0.093 | 0.291 | 4.08E-12 | 2 | *TOP2A* | Rod precursors |
| CENPF.2 | 1.43E-12 | -1.01234 | 0.145 | 0.312 | 3.16E-08 | 2 | *CENPF* | Rod precursors |
| B2M | 9.16E-52 | -1.0145 | 0.493 | 0.774 | 2.02E-47 | 2 | *B2M* | Rod precursors |
| IGFBP5 | 2.10E-15 | -1.02295 | 0.252 | 0.445 | 4.63E-11 | 2 | *IGFBP5* | Rod precursors |
| SOX2.2 | 7.13E-43 | -1.02854 | 0.052 | 0.433 | 1.57E-38 | 2 | *SOX2* | Rod precursors |
| DKK3.2 | 1.76E-41 | -1.04879 | 0.139 | 0.511 | 3.88E-37 | 2 | *DKK3* | Rod precursors |
| IGFBP2 | 1.70E-11 | -1.08042 | 0.417 | 0.533 | 3.75E-07 | 2 | *IGFBP2* | Rod precursors |
| LAMP5.1 | 5.01E-22 | -1.09732 | 0.101 | 0.339 | 1.11E-17 | 2 | *LAMP5* | Rod precursors |
| ZFP36L1.2 | 1.79E-40 | -1.11089 | 0.104 | 0.466 | 3.95E-36 | 2 | *ZFP36L1* | Rod precursors |
| MDK.1 | 2.94E-56 | -1.11615 | 0.362 | 0.744 | 6.49E-52 | 2 | *MDK* | Rod precursors |
| PTN.1 | 1.31E-42 | -1.11877 | 0.186 | 0.562 | 2.89E-38 | 2 | *PTN* | Rod precursors |
| CCND1 | 3.83E-34 | -1.11907 | 0.145 | 0.473 | 8.46E-30 | 2 | *CCND1* | Rod precursors |
| EGR1 | 3.62E-50 | -1.13222 | 0.18 | 0.59 | 7.98E-46 | 2 | *EGR1* | Rod precursors |
| COL1A2 | 3.12E-32 | -1.17795 | 0.075 | 0.393 | 6.89E-28 | 2 | *COL1A2* | Rod precursors |
| NEAT1 | 5.08E-43 | -1.19741 | 0.568 | 0.788 | 1.12E-38 | 2 | *NEAT1* | Rod precursors |
| FOS | 2.91E-42 | -1.24717 | 0.232 | 0.58 | 6.43E-38 | 2 | *FOS* | Rod precursors |
| SPP1.2 | 1.78E-36 | -1.41159 | 0.188 | 0.515 | 3.92E-32 | 2 | *SPP1* | Rod precursors |
| TRH.1 | 1.16E-29 | -1.56452 | 0.345 | 0.586 | 2.56E-25 | 2 | *TRH* | Rod precursors |
| CLU.2 | 5.96E-69 | -1.5829 | 0.252 | 0.7 | 1.32E-64 | 2 | *CLU* | Rod precursors |
| CYP26A1.2 | 5.98E-27 | -1.72126 | 0.212 | 0.474 | 1.32E-22 | 2 | *CYP26A1* | Rod precursors |
| SFRP2.1 | 3.34E-40 | -1.75667 | 0.235 | 0.568 | 7.37E-36 | 2 | *SFRP2* | Rod precursors |
| CRABP1.1 | 2.65E-61 | -1.79859 | 0.464 | 0.759 | 5.86E-57 | 2 | *CRABP1* | Rod precursors |
| VIM.2 | 1.18E-56 | -1.83783 | 0.629 | 0.814 | 2.60E-52 | 2 | *VIM* | Rod precursors |
| APOC1 | 5.18E-96 | 1.103344 | 0.704 | 0.241 | 1.14E-91 | 3 | *APOC1* | Cone precursors |
| OLAH | 7.88E-142 | 1.047244 | 0.401 | 0.046 | 1.74E-137 | 3 | *OLAH* | Cone precursors |
| VXN.2 | 6.90E-110 | 0.968597 | 0.751 | 0.222 | 1.52E-105 | 3 | *VXN* | Cone precursors |
| RBP4 | 5.25E-170 | 0.961983 | 0.82 | 0.185 | 1.16E-165 | 3 | *RBP4* | Cone precursors |
| ARR3 | 4.85E-135 | 0.933559 | 0.593 | 0.114 | 1.07E-130 | 3 | *ARR3* | Cone precursors |
| SCG3.3 | 5.62E-100 | 0.926528 | 0.943 | 0.5 | 1.24E-95 | 3 | *SCG3* | Cone precursors |
| CPLX3 | 6.53E-126 | 0.925628 | 0.784 | 0.23 | 1.44E-121 | 3 | *CPLX3* | Cone precursors |
| PDE6H.3 | 1.82E-86 | 0.921399 | 0.841 | 0.348 | 4.01E-82 | 3 | *PDE6H* | Cone precursors |
| RS1 | 7.16E-140 | 0.908954 | 0.62 | 0.119 | 1.58E-135 | 3 | *RS1* | Cone precursors |
| PDC.3 | 5.29E-116 | 0.899469 | 0.964 | 0.388 | 1.17E-111 | 3 | *PDC* | Cone precursors |
| TUBA4A.2 | 7.51E-102 | 0.893908 | 0.862 | 0.353 | 1.66E-97 | 3 | *TUBA4A* | Cone precursors |
| NT5DC2 | 1.18E-69 | 0.89088 | 0.802 | 0.435 | 2.60E-65 | 3 | *NT5DC2* | Cone precursors |
| PHOX2A | 3.84E-160 | 0.880396 | 0.662 | 0.119 | 8.48E-156 | 3 | *PHOX2A* | Cone precursors |
| PRCD | 3.65E-129 | 0.867255 | 0.778 | 0.22 | 8.06E-125 | 3 | *PRCD* | Cone precursors |
| TMEM70 | 3.53E-54 | 0.847045 | 0.751 | 0.394 | 7.79E-50 | 3 | *TMEM70* | Cone precursors |
| PLEKHB1.1 | 2.36E-106 | 0.840063 | 0.838 | 0.303 | 5.20E-102 | 3 | *PLEKHB1* | Cone precursors |
| RTBDN | 3.64E-152 | 0.837304 | 0.662 | 0.128 | 8.03E-148 | 3 | *RTBDN* | Cone precursors |
| AKAP9.3 | 6.73E-69 | 0.83724 | 0.907 | 0.641 | 1.49E-64 | 3 | *AKAP9* | Cone precursors |
| C1QL1 | 7.22E-94 | 0.816614 | 0.674 | 0.216 | 1.59E-89 | 3 | *C1QL1* | Cone precursors |
| ENO2.3 | 2.95E-92 | 0.807852 | 0.955 | 0.625 | 6.52E-88 | 3 | *ENO2* | Cone precursors |
| GNG8 | 1.22E-145 | 0.802652 | 0.56 | 0.095 | 2.70E-141 | 3 | *GNG8* | Cone precursors |
| GNG4 | 1.67E-119 | 0.792857 | 0.811 | 0.268 | 3.68E-115 | 3 | *GNG4* | Cone precursors |
| CAMK2B | 9.52E-90 | 0.781884 | 0.68 | 0.229 | 2.10E-85 | 3 | *CAMK2B* | Cone precursors |
| MPP4.1 | 3.62E-115 | 0.781115 | 0.707 | 0.178 | 7.99E-111 | 3 | *MPP4* | Cone precursors |
| CRX.3 | 5.34E-99 | 0.766121 | 0.85 | 0.29 | 1.18E-94 | 3 | *CRX* | Cone precursors |
| HSPB11 | 3.56E-73 | 0.764014 | 0.862 | 0.51 | 7.87E-69 | 3 | *HSPB11* | Cone precursors |
| CPE.3 | 4.40E-76 | 0.753802 | 0.937 | 0.646 | 9.71E-72 | 3 | *CPE* | Cone precursors |
| MYLK | 9.28E-122 | 0.753018 | 0.65 | 0.158 | 2.05E-117 | 3 | *MYLK* | Cone precursors |
| CCNE2 | 3.33E-107 | 0.741163 | 0.749 | 0.234 | 7.36E-103 | 3 | *CCNE2* | Cone precursors |
| H2AFY | 3.15E-92 | 0.71835 | 0.955 | 0.801 | 6.95E-88 | 3 | *H2AFY* | Cone precursors |
| ATP1B1 | 3.00E-62 | 0.705178 | 0.907 | 0.692 | 6.62E-58 | 3 | *ATP1B1* | Cone precursors |
| SEPT4.3 | 7.49E-89 | 0.702979 | 0.901 | 0.361 | 1.65E-84 | 3 | *Sep-04* | Cone precursors |
| TMEM97 | 1.62E-73 | 0.700869 | 0.832 | 0.447 | 3.57E-69 | 3 | *TMEM97* | Cone precursors |
| RRAD.3 | 1.36E-42 | 0.700709 | 0.653 | 0.296 | 3.01E-38 | 3 | *RRAD* | Cone precursors |
| MIAT | 1.27E-12 | -0.70686 | 0.272 | 0.412 | 2.80E-08 | 3 | *MIAT* | Cone precursors |

| ZFP36L2.1 | 1.21E-15 | -0.7077 | 0.305 | 0.454 | 2.68E-11 | 3 | *ZFP36L2* | Cone precursors |
| --- | --- | --- | --- | --- | --- | --- | --- | --- |
| LGALS3.1 | 1.30E-10 | -0.71069 | 0.072 | 0.205 | 2.88E-06 | 3 | *LGALS3* | Cone precursors |
| GPC3.2 | 1.03E-06 | -0.71127 | 0.272 | 0.35 | 0.022659 | 3 | *GPC3* | Cone precursors |
| PTGDS.1 | 8.44E-11 | -0.71484 | 0.063 | 0.201 | 1.86E-06 | 3 | *PTGDS* | Cone precursors |
| NPC2 | 3.77E-26 | -0.72181 | 0.275 | 0.507 | 8.33E-22 | 3 | *NPC2* | Cone precursors |
| IER2 | 1.96E-24 | -0.72334 | 0.674 | 0.727 | 4.32E-20 | 3 | *IER2* | Cone precursors |
| TTYH1.2 | 1.18E-14 | -0.72566 | 0.323 | 0.462 | 2.61E-10 | 3 | *TTYH1* | Cone precursors |
| JUNB | 2.82E-15 | -0.72807 | 0.509 | 0.584 | 6.23E-11 | 3 | *JUNB* | Cone precursors |
| HLA-B | 8.09E-17 | -0.74226 | 0.081 | 0.279 | 1.79E-12 | 3 | *HLA-B* | Cone precursors |
| PRSS23.2 | 1.90E-10 | -0.74508 | 0.377 | 0.45 | 4.20E-06 | 3 | *PRSS23* | Cone precursors |
| PAX6.1 | 4.79E-25 | -0.74741 | 0.159 | 0.426 | 1.06E-20 | 3 | *PAX6* | Cone precursors |
| HLA-A | 7.40E-28 | -0.76166 | 0.189 | 0.467 | 1.63E-23 | 3 | *HLA-A* | Cone precursors |
| HES1.2 | 5.92E-17 | -0.79329 | 0.213 | 0.397 | 1.31E-12 | 3 | *HES1* | Cone precursors |
| MEST.1 | 2.12E-30 | -0.8192 | 0.281 | 0.531 | 4.68E-26 | 3 | *MEST* | Cone precursors |
| DAPL1.2 | 2.20E-14 | -0.8395 | 0.449 | 0.532 | 4.86E-10 | 3 | *DAPL1* | Cone precursors |
| DIO3.3 | 2.35E-23 | -0.91104 | 0.072 | 0.324 | 5.19E-19 | 3 | *DIO3* | Cone precursors |
| TF.3 | 2.41E-05 | -0.91338 | 0.138 | 0.219 | 0.531733 | 3 | *TF* | Cone precursors |
| FABP7.1 | 1.83E-08 | -0.94506 | 0.647 | 0.61 | 0.000404 | 3 | *FABP7* | Cone precursors |
| IFITM3.2 | 9.44E-29 | -0.94809 | 0.075 | 0.372 | 2.08E-24 | 3 | *IFITM3* | Cone precursors |
| IFITM2.3 | 7.07E-25 | -0.95648 | 0.039 | 0.297 | 1.56E-20 | 3 | *IFITM2* | Cone precursors |
| DKK3.3 | 1.08E-33 | -0.98472 | 0.198 | 0.506 | 2.39E-29 | 3 | *DKK3* | Cone precursors |
| SOX2.3 | 4.05E-37 | -0.99307 | 0.084 | 0.431 | 8.95E-33 | 3 | *SOX2* | Cone precursors |
| PTN.2 | 8.70E-24 | -1.02703 | 0.368 | 0.549 | 1.92E-19 | 3 | *PTN* | Cone precursors |
| MDK.2 | 5.61E-37 | -1.02996 | 0.602 | 0.727 | 1.24E-32 | 3 | *MDK* | Cone precursors |
| LAMP5.2 | 2.28E-20 | -1.06194 | 0.111 | 0.338 | 5.03E-16 | 3 | *LAMP5* | Cone precursors |
| IGFBP5.1 | 1.91E-22 | -1.10053 | 0.204 | 0.447 | 4.22E-18 | 3 | *IGFBP5* | Cone precursors |
| COL1A2.1 | 2.80E-29 | -1.11964 | 0.09 | 0.391 | 6.19E-25 | 3 | *COL1A2* | Cone precursors |
| ZFP36L1.3 | 5.35E-38 | -1.13593 | 0.126 | 0.464 | 1.18E-33 | 3 | *ZFP36L1* | Cone precursors |
| EGR1.1 | 2.75E-44 | -1.18536 | 0.266 | 0.583 | 6.06E-40 | 3 | *EGR1* | Cone precursors |
| CCND1.1 | 5.68E-39 | -1.21142 | 0.114 | 0.474 | 1.25E-34 | 3 | *CCND1* | Cone precursors |
| CLU.3 | 8.09E-34 | -1.22533 | 0.542 | 0.68 | 1.79E-29 | 3 | *CLU* | Cone precursors |
| TRH.2 | 2.92E-12 | -1.27455 | 0.503 | 0.575 | 6.44E-08 | 3 | *TRH* | Cone precursors |
| FOS.1 | 3.25E-44 | -1.46426 | 0.263 | 0.578 | 7.19E-40 | 3 | *FOS* | Cone precursors |
| SPP1.3 | 1.97E-37 | -1.51475 | 0.195 | 0.514 | 4.34E-33 | 3 | *SPP1* | Cone precursors |
| CYP26A1.3 | 2.03E-22 | -1.67781 | 0.26 | 0.47 | 4.48E-18 | 3 | *CYP26A1* | Cone precursors |
| CRABP1.2 | 1.70E-50 | -1.77392 | 0.596 | 0.749 | 3.75E-46 | 3 | *CRABP1* | Cone precursors |
| VIM.3 | 2.52E-65 | -1.91658 | 0.638 | 0.813 | 5.56E-61 | 3 | *VIM* | Cone precursors |
| SFRP2.2 | 1.23E-37 | -1.93874 | 0.263 | 0.565 | 2.72E-33 | 3 | *SFRP2* | Cone precursors |
| HIST1H4C. | 8.04E-139 | 2.593866 | 0.905 | 0.566 | 1.77E-134 | 4 | *HIST1H4C* | Mitotic cells |
| UBE2C.3 | 4.08E-251 | 1.934218 | 0.847 | 0.157 | 9.00E-247 | 4 | *UBE2C* | Mitotic cells |
| CENPF.3 | 3.49E-193 | 1.818037 | 0.89 | 0.264 | 7.72E-189 | 4 | *CENPF* | Mitotic cells |
| TFF1 | 2.66E-250 | 1.794115 | 0.521 | 0.038 | 5.88E-246 | 4 | *TFF1* | Mitotic cells |
| TOP2A.3 | 5.31E-174 | 1.717875 | 0.831 | 0.242 | 1.17E-169 | 4 | *TOP2A* | Mitotic cells |
| BIRC5 | 2.39E-206 | 1.432199 | 0.629 | 0.086 | 5.28E-202 | 4 | *BIRC5* | Mitotic cells |
| MKI67 | 3.32E-160 | 1.43149 | 0.702 | 0.159 | 7.32E-156 | 4 | *MKI67* | Mitotic cells |
| HMGB2.3 | 2.35E-145 | 1.416996 | 0.979 | 0.715 | 5.18E-141 | 4 | *HMGB2* | Mitotic cells |
| NUSAP1.2 | 8.50E-105 | 1.369142 | 0.748 | 0.308 | 1.88E-100 | 4 | *NUSAP1* | Mitotic cells |
| NEK2 | 4.18E-166 | 1.36481 | 0.528 | 0.074 | 9.23E-162 | 4 | *NEK2* | Mitotic cells |
| PTTG1.2 | 9.25E-100 | 1.35978 | 0.785 | 0.377 | 2.04E-95 | 4 | *PTTG1* | Mitotic cells |
| CKAP2 | 6.34E-83 | 1.312842 | 0.715 | 0.345 | 1.40E-78 | 4 | *CKAP2* | Mitotic cells |
| TUBB | 1.38E-121 | 1.292542 | 0.994 | 0.945 | 3.04E-117 | 4 | *TUBB* | Mitotic cells |
| SMC4 | 4.59E-90 | 1.286572 | 0.718 | 0.322 | 1.01E-85 | 4 | *SMC4* | Mitotic cells |
| CKS1B | 3.23E-80 | 1.248555 | 0.598 | 0.222 | 7.13E-76 | 4 | *CKS1B* | Mitotic cells |
| DCT.2 | 4.35E-57 | 1.232786 | 0.595 | 0.271 | 9.60E-53 | 4 | *DCT* | Mitotic cells |
| HLA-DQB1 | 4.37E-75 | 1.231808 | 0.344 | 0.069 | 9.65E-71 | 4 | *HLA-DQB1* | Mitotic cells |
| KIFC1 | 4.37E-131 | 1.222049 | 0.564 | 0.117 | 9.66E-127 | 4 | *KIFC1* | Mitotic cells |
| TPX2 | 2.93E-111 | 1.220182 | 0.613 | 0.168 | 6.47E-107 | 4 | *TPX2* | Mitotic cells |
| HLA-B.1 | 2.40E-06 | 1.187676 | 0.331 | 0.263 | 0.052901 | 4 | *HLA-B* | Mitotic cells |
| UBE2S.2 | 1.76E-78 | 1.184968 | 0.84 | 0.655 | 3.89E-74 | 4 | *UBE2S* | Mitotic cells |
| HSPA1A | 9.05E-15 | 1.170668 | 0.347 | 0.219 | 2.00E-10 | 4 | *HSPA1A* | Mitotic cells |

HMGA1.2 DEK HMGB1 KPNA2 HIST1H1D CCNB2 CDKN2D ANP32E CENPA GMNN ASPM CDC20 PBK

| 3.62E-47 | 1.139266 | 0.816 | 0.645 | 7.99E-43 | 4 | *HMGA1* | Mitotic cells |
| --- | --- | --- | --- | --- | --- | --- | --- |
| 1.30E-106 | 1.13432 | 0.966 | 0.857 | 2.87E-102 | 4 | *DEK* | Mitotic cells |
| 1.70E-138 | 1.120233 | 0.988 | 0.953 | 3.76E-134 | 4 | *HMGB1* | Mitotic cells |
| 5.17E-45 | 1.11357 | 0.635 | 0.388 | 1.14E-40 | 4 | *KPNA2* | Mitotic cells |
| 1.14E-36 | 1.110061 | 0.307 | 0.1 | 2.52E-32 | 4 | *HIST1H1D* | Mitotic cells |
| 3.88E-107 | 1.091568 | 0.5 | 0.104 | 8.56E-103 | 4 | *CCNB2* | Mitotic cells |
| 1.61E-46 | 1.076425 | 0.506 | 0.229 | 3.56E-42 | 4 | *CDKN2D* | Mitotic cells |
| 2.89E-67 | 1.058859 | 0.807 | 0.553 | 6.38E-63 | 4 | *ANP32E* | Mitotic cells |
| 8.07E-125 | 1.025786 | 0.396 | 0.051 | 1.78E-120 | 4 | *CENPA* | Mitotic cells |
| 6.31E-44 | 1.013661 | 0.669 | 0.443 | 1.39E-39 | 4 | *GMNN* | Mitotic cells |
| 5.45E-101 | 0.979155 | 0.46 | 0.089 | 1.20E-96 | 4 | *ASPM* | Mitotic cells |
| 2.84E-111 | 0.967698 | 0.387 | 0.055 | 6.27E-107 | 4 | *CDC20* | Mitotic cells |
| 7.77E-130 | 0.965498 | 0.469 | 0.072 | 1.72E-125 | 4 | *PBK* | Mitotic cells |
| 2.89E-14 | 0.960209 | 0.564 | 0.442 | 6.39E-10 | 4 | *HLA-A* | Mitotic cells |
| 5.06E-51 | 0.956333 | 0.423 | 0.145 | 1.12E-46 | 4 | *HIST1H1C* | Mitotic cells |
| 1.68E-53 | 0.939012 | 0.706 | 0.466 | 3.71E-49 | 4 | *UBE2T* | Mitotic cells |
| 1.00E-56 | 0.923413 | 0.951 | 0.826 | 2.21E-52 | 4 | *TUBB2B* | Mitotic cells |
| 2.54E-63 | 0.923223 | 0.426 | 0.122 | 5.61E-59 | 4 | *GTSE1* | Mitotic cells |
| 7.38E-43 | 0.919693 | 0.387 | 0.133 | 1.63E-38 | 4 | *CCNB1* | Mitotic cells |
| 1.58E-30 | 0.917546 | 0.702 | 0.581 | 3.50E-26 | 4 | *ARL6IP1* | Mitotic cells |
| 3.99E-127 | 0.905978 | 0.393 | 0.05 | 8.81E-123 | 4 | *KNL1* | Mitotic cells |
| 1.10E-115 | 0.901118 | 0.236 | 0.016 | 2.42E-111 | 4 | *RPS4Y1* | Mitotic cells |
| 1.39E-32 | 0.900649 | 0.567 | 0.361 | 3.06E-28 | 4 | *DDX39A* | Mitotic cells |
| 1.16E-44 | 0.893791 | 0.518 | 0.25 | 2.56E-40 | 4 | *MAD2L1* | Mitotic cells |
| 3.12E-56 | 0.891978 | 0.491 | 0.179 | 6.88E-52 | 4 | *RRM2* | Mitotic cells |
| 1.08E-33 | 0.878688 | 0.448 | 0.219 | 2.39E-29 | 4 | *CDK1* | Mitotic cells |
| 1.04E-94 | 0.873651 | 0.396 | 0.069 | 2.29E-90 | 4 | *NUF2* | Mitotic cells |
| 1.27E-76 | 0.872816 | 0.39 | 0.084 | 2.81E-72 | 4 | *SGO2* | Mitotic cells |
| 4.71E-28 | 0.866721 | 0.718 | 0.576 | 1.04E-23 | 4 | *SOX11* | Mitotic cells |
| 1.56E-41 | 0.861252 | 0.663 | 0.469 | 3.44E-37 | 4 | *CKS2* | Mitotic cells |
| 1.79E-77 | 0.859011 | 0.374 | 0.077 | 3.94E-73 | 4 | *NDC80* | Mitotic cells |
| 6.42E-37 | 0.858977 | 0.577 | 0.334 | 1.42E-32 | 4 | *SMC2* | Mitotic cells |
| 7.19E-92 | 0.85139 | 0.387 | 0.069 | 1.59E-87 | 4 | *CCNA2* | Mitotic cells |
| 7.48E-82 | 0.845167 | 0.38 | 0.076 | 1.65E-77 | 4 | *SGO1* | Mitotic cells |
| 2.42E-87 | 0.844879 | 0.383 | 0.071 | 5.35E-83 | 4 | *CKAP2L* | Mitotic cells |
| 2.12E-108 | 0.844776 | 0.224 | 0.015 | 4.68E-104 | 4 | *TPRX1* | Mitotic cells |
| 1.32E-100 | 0.835385 | 0.991 | 0.956 | 2.91E-96 | 4 | *TUBA1B* | Mitotic cells |
| 3.54E-94 | 0.834292 | 1 | 0.961 | 7.81E-90 | 4 | *STMN1* | Mitotic cells |
| 2.26E-107 | 0.832468 | 0.359 | 0.049 | 4.98E-103 | 4 | *AURKB* | Mitotic cells |
| 1.97E-96 | 0.832088 | 0.368 | 0.058 | 4.35E-92 | 4 | *PIMREG* | Mitotic cells |
| 9.45E-137 | 0.826834 | 0.304 | 0.024 | 2.09E-132 | 4 | *HIST1H1B* | Mitotic cells |
| 2.16E-57 | 0.824927 | 0.426 | 0.129 | 4.77E-53 | 4 | *KIF20B* | Mitotic cells |
| 3.94E-104 | 0.817428 | 0.374 | 0.054 | 8.69E-100 | 4 | *DLGAP5* | Mitotic cells |
| 3.07E-95 | 0.812359 | 0.979 | 0.934 | 6.77E-91 | 4 | *H2AFZ* | Mitotic cells |
| 4.68E-37 | 0.811317 | 0.42 | 0.178 | 1.03E-32 | 4 | *CDKN3* | Mitotic cells |
| 1.61E-27 | 0.804715 | 0.589 | 0.426 | 3.55E-23 | 4 | *MZT1* | Mitotic cells |
| 7.06E-19 | 0.796875 | 0.543 | 0.381 | 1.56E-14 | 4 | *STMN2* | Mitotic cells |
| 6.78E-11 | 0.789008 | 0.46 | 0.384 | 1.50E-06 | 4 | *TBCC* | Mitotic cells |
| 5.62E-31 | 0.788147 | 0.334 | 0.131 | 1.24E-26 | 4 | *PRC1* | Mitotic cells |
| 1.53E-91 | 0.77665 | 0.331 | 0.049 | 3.37E-87 | 4 | *CDCA8* | Mitotic cells |
| 7.65E-77 | 0.775242 | 0.347 | 0.065 | 1.69E-72 | 4 | *KIF23* | Mitotic cells |
| 9.29E-30 | 0.769805 | 0.776 | 0.7 | 2.05E-25 | 4 | *ILF2* | Mitotic cells |
| 8.01E-56 | 0.761619 | 0.874 | 0.848 | 1.77E-51 | 4 | *NUCKS1* | Mitotic cells |
| 9.95E-14 | 0.758085 | 0.267 | 0.144 | 2.20E-09 | 4 | *IGDCC3* | Mitotic cells |
| 1 3.79E-31 | 0.755451 | 0.451 | 0.227 | 8.38E-27 | 4 | *MIS18BP1* | Mitotic cells |
| 2.08E-29 | 0.750243 | 0.506 | 0.291 | 4.59E-25 | 4 | *CENPU* | Mitotic cells |
| 1.03E-19 | 0.740141 | 0.546 | 0.427 | 2.27E-15 | 4 | *HMGB3* | Mitotic cells |
| 4.31E-11 | 0.739272 | 0.555 | 0.514 | 9.52E-07 | 4 | *BRD2* | Mitotic cells |
| 1.48E-82 | 0.734733 | 0.298 | 0.045 | 3.27E-78 | 4 | *SKA3* | Mitotic cells |
| 1.48E-17 | 0.731984 | 0.442 | 0.3 | 3.26E-13 | 4 | *H2AFX* | Mitotic cells |

HLA-A.1 HIST1H1C UBE2T TUBB2B GTSE1 CCNB1 ARL6IP1 KNL1 RPS4Y1 DDX39A MAD2L1 RRM2 CDK1 NUF2 SGO2 SOX11.1 CKS2 NDC80 SMC2 CCNA2 SGO1 CKAP2L TPRX1 TUBA1B STMN1.1 AURKB PIMREG HIST1H1B KIF20B DLGAP5 H2AFZ CDKN3 MZT1 STMN2.2 TBCC PRC1 CDCA8 KIF23 ILF2 NUCKS1 IGDCC3 MIS18BP CENPU HMGB3 BRD2 SKA3 H2AFX

| RAD51AP1 | 8.76E-29 | 0.726908 | 0.387 | 0.179 | 1.93E-24 | 4 | *RAD51AP1* | Mitotic cells |
| --- | --- | --- | --- | --- | --- | --- | --- | --- |
| ODC1 | 4.90E-24 | 0.726315 | 0.696 | 0.611 | 1.08E-19 | 4 | *ODC1* | Mitotic cells |
| RBM8A | 3.80E-20 | 0.725973 | 0.678 | 0.614 | 8.38E-16 | 4 | *RBM8A* | Mitotic cells |
| TPH1.2 | 8.67E-17 | 0.72229 | 0.374 | 0.215 | 1.92E-12 | 4 | *TPH1* | Mitotic cells |
| PPP1CC | 4.07E-18 | 0.719534 | 0.635 | 0.549 | 8.99E-14 | 4 | *PPP1CC* | Mitotic cells |
| DHFR | 5.59E-25 | 0.718305 | 0.491 | 0.296 | 1.23E-20 | 4 | *DHFR* | Mitotic cells |
| CDCA3 | 7.60E-54 | 0.717759 | 0.319 | 0.076 | 1.68E-49 | 4 | *CDCA3* | Mitotic cells |
| SSTR2 | 4.38E-11 | 0.716628 | 0.325 | 0.212 | 9.68E-07 | 4 | *SSTR2* | Mitotic cells |
| FBL | 1.98E-10 | 0.715815 | 0.537 | 0.493 | 4.37E-06 | 4 | *FBL* | Mitotic cells |
| BUB3 | 1.96E-21 | 0.715139 | 0.61 | 0.505 | 4.33E-17 | 4 | *BUB3* | Mitotic cells |
| CENPW | 9.69E-42 | 0.715117 | 0.328 | 0.1 | 2.14E-37 | 4 | *CENPW* | Mitotic cells |
| GAP43 | 1.32E-11 | 0.711998 | 0.39 | 0.273 | 2.92E-07 | 4 | *GAP43* | Mitotic cells |
| NUDT3 | 3.19E-06 | 0.707992 | 0.39 | 0.346 | 0.070415 | 4 | *NUDT3* | Mitotic cells |
| CLSPN | 9.54E-25 | 0.700685 | 0.491 | 0.284 | 2.11E-20 | 4 | *CLSPN* | Mitotic cells |
| ROM1.1 | 4.35E-17 | -0.70625 | 0.031 | 0.231 | 9.60E-13 | 4 | *ROM1* | Mitotic cells |
| GPC3.3 | 2.20E-12 | -0.70781 | 0.172 | 0.357 | 4.87E-08 | 4 | *GPC3* | Mitotic cells |
| PTGDS.2 | 2.16E-13 | -0.71073 | 0.037 | 0.202 | 4.77E-09 | 4 | *PTGDS* | Mitotic cells |
| SPARC | 2.78E-31 | -0.7112 | 0.175 | 0.511 | 6.14E-27 | 4 | *SPARC* | Mitotic cells |
| DAPL1.3 | 4.18E-24 | -0.7177 | 0.239 | 0.546 | 9.23E-20 | 4 | *DAPL1* | Mitotic cells |
| CD81 | 6.23E-37 | -0.71813 | 0.16 | 0.547 | 1.38E-32 | 4 | *CD81* | Mitotic cells |
| CRABP1.3 | 2.27E-30 | -0.72983 | 0.396 | 0.762 | 5.00E-26 | 4 | *CRABP1* | Mitotic cells |
| TTYH1.3 | 3.45E-26 | -0.73088 | 0.166 | 0.472 | 7.62E-22 | 4 | *TTYH1* | Mitotic cells |
| METRN.2 | 4.19E-26 | -0.76996 | 0.129 | 0.427 | 9.24E-22 | 4 | *METRN* | Mitotic cells |
| APOE | 1.64E-23 | -0.77095 | 0.172 | 0.469 | 3.62E-19 | 4 | *APOE* | Mitotic cells |
| SYT1 | 2.47E-43 | -0.78457 | 0.245 | 0.659 | 5.46E-39 | 4 | *SYT1* | Mitotic cells |
| IFITM3.3 | 3.83E-20 | -0.80421 | 0.12 | 0.369 | 8.45E-16 | 4 | *IFITM3* | Mitotic cells |
| VIM.4 | 8.29E-29 | -0.82318 | 0.472 | 0.823 | 1.83E-24 | 4 | *VIM* | Mitotic cells |
| COL1A2.2 | 1.39E-14 | -0.85656 | 0.178 | 0.385 | 3.07E-10 | 4 | *COL1A2* | Mitotic cells |
| CRABP2 | 1.70E-42 | -0.90005 | 0.175 | 0.59 | 3.75E-38 | 4 | *CRABP2* | Mitotic cells |
| CYP26A1.4 | 1.54E-16 | -0.95277 | 0.236 | 0.471 | 3.40E-12 | 4 | *CYP26A1* | Mitotic cells |
| LAMP5.3 | 4.49E-17 | -0.95459 | 0.12 | 0.337 | 9.92E-13 | 4 | *LAMP5* | Mitotic cells |
| TF.4 | 2.16E-11 | -0.97335 | 0.071 | 0.223 | 4.77E-07 | 4 | *TF* | Mitotic cells |
| IGFBP5.2 | 2.05E-23 | -0.98568 | 0.156 | 0.45 | 4.53E-19 | 4 | *IGFBP5* | Mitotic cells |
| NRL.3 | 3.52E-08 | -0.98976 | 0.206 | 0.351 | 0.000777 | 4 | *NRL* | Mitotic cells |
| CLU.4 | 1.05E-36 | -1.0174 | 0.331 | 0.693 | 2.32E-32 | 4 | *CLU* | Mitotic cells |
| TRH.3 | 3.00E-21 | -1.02641 | 0.298 | 0.588 | 6.64E-17 | 4 | *TRH* | Mitotic cells |
| SPP1.4 | 1.64E-24 | -1.04107 | 0.23 | 0.511 | 3.63E-20 | 4 | *SPP1* | Mitotic cells |
| FABP7.2 | 3.21E-37 | -1.06614 | 0.248 | 0.636 | 7.08E-33 | 4 | *FABP7* | Mitotic cells |
| PDC.4 | 2.41E-23 | -1.34243 | 0.175 | 0.44 | 5.32E-19 | 4 | *PDC* | Mitotic cells |
| IGFBP2.1 | 2.21E-32 | -1.35699 | 0.202 | 0.546 | 4.87E-28 | 4 | *IGFBP2* | Mitotic cells |
| SFRP2.3 | 2.28E-23 | -1.39221 | 0.301 | 0.562 | 5.03E-19 | 4 | *SFRP2* | Mitotic cells |
| GADD45A. | 8.47E-119 | 1.446693 | 0.941 | 0.504 | 1.87E-114 | 5 | *GADD45A* | Proliferating NRPCs |
| PMAIP1.1 | 4.36E-132 | 1.429457 | 0.934 | 0.397 | 9.63E-128 | 5 | *PMAIP1* | Proliferating NRPCs |
| L1TD1 | 1.94E-131 | 1.170841 | 0.433 | 0.054 | 4.27E-127 | 5 | *L1TD1* | Proliferating NRPCs |
| PCLAF | 3.08E-123 | 1.061955 | 0.931 | 0.345 | 6.80E-119 | 5 | *PCLAF* | Proliferating NRPCs |
| SHD | 2.95E-122 | 1.057438 | 0.869 | 0.326 | 6.51E-118 | 5 | *SHD* | Proliferating NRPCs |
| ASF1B | 2.06E-127 | 0.984703 | 0.682 | 0.158 | 4.55E-123 | 5 | *ASF1B* | Proliferating NRPCs |
| CENPU.1 | 4.13E-114 | 0.932286 | 0.837 | 0.274 | 9.13E-110 | 5 | *CENPU* | Proliferating NRPCs |
| TYMS.1 | 5.43E-96 | 0.917811 | 0.976 | 0.604 | 1.20E-91 | 5 | *TYMS* | Proliferating NRPCs |
| CHGB | 3.58E-15 | 0.87196 | 0.453 | 0.269 | 7.90E-11 | 5 | *CHGB* | Proliferating NRPCs |
| SLBP | 1.71E-87 | 0.870557 | 0.9 | 0.508 | 3.78E-83 | 5 | *SLBP* | Proliferating NRPCs |
| FAM111B | 1.12E-91 | 0.857945 | 0.803 | 0.276 | 2.48E-87 | 5 | *FAM111B* | Proliferating NRPCs |
| NEAT1.1 | 1.77E-36 | 0.852695 | 0.907 | 0.767 | 3.90E-32 | 5 | *NEAT1* | Proliferating NRPCs |
| CXCR4 | 1.69E-81 | 0.84827 | 0.765 | 0.284 | 3.73E-77 | 5 | *CXCR4* | Proliferating NRPCs |
| PTMA | 1.62E-105 | 0.817531 | 1 | 0.989 | 3.57E-101 | 5 | *PTMA* | Proliferating NRPCs |
| HSPB11.1 | 3.16E-75 | 0.788035 | 0.886 | 0.512 | 6.99E-71 | 5 | *HSPB11* | Proliferating NRPCs |
| BTG3 | 1.93E-78 | 0.78639 | 0.938 | 0.651 | 4.26E-74 | 5 | *BTG3* | Proliferating NRPCs |
| WNT16 | 5.29E-135 | 0.780675 | 0.478 | 0.065 | 1.17E-130 | 5 | *WNT16* | Proliferating NRPCs |
| CCNE2.1 | 1.64E-90 | 0.776283 | 0.723 | 0.239 | 3.63E-86 | 5 | *CCNE2* | Proliferating NRPCs |
| CARHSP1 | 6.69E-78 | 0.767293 | 0.91 | 0.554 | 1.48E-73 | 5 | *CARHSP1* | Proliferating NRPCs |

| PCNA | 1.79E-71 | 0.760623 | 0.91 | 0.553 | 3.95E-67 | 5 | *PCNA* | Proliferating NRPCs |
| --- | --- | --- | --- | --- | --- | --- | --- | --- |
| SYNE2 | 3.55E-60 | 0.740544 | 0.92 | 0.651 | 7.83E-56 | 5 | *SYNE2* | Proliferating NRPCs |
| HMGN2 | 7.41E-72 | 0.737237 | 0.997 | 0.918 | 1.64E-67 | 5 | *HMGN2* | Proliferating NRPCs |
| USP1 | 1.10E-66 | 0.732337 | 0.889 | 0.511 | 2.43E-62 | 5 | *USP1* | Proliferating NRPCs |
| DLK1 | 3.70E-19 | 0.724115 | 0.343 | 0.152 | 8.16E-15 | 5 | *DLK1* | Proliferating NRPCs |
| FBLN1 | 8.26E-51 | 0.711851 | 0.792 | 0.428 | 1.82E-46 | 5 | *FBLN1* | Proliferating NRPCs |
| TMSB15A | 8.50E-63 | 0.711201 | 0.92 | 0.623 | 1.88E-58 | 5 | *TMSB15A* | Proliferating NRPCs |
| IFITM2.4 | 6.35E-09 | -0.70397 | 0.149 | 0.289 | 0.00014 | 5 | *IFITM2* | Proliferating NRPCs |
| PTGDS.3 | 4.97E-09 | -0.70623 | 0.066 | 0.199 | 0.00011 | 5 | *PTGDS* | Proliferating NRPCs |
| PRDX1.1 | 1.44E-33 | -0.70742 | 0.737 | 0.827 | 3.17E-29 | 5 | *PRDX1* | Proliferating NRPCs |
| SIX6 | 2.38E-26 | -0.70917 | 0.547 | 0.705 | 5.26E-22 | 5 | *SIX6* | Proliferating NRPCs |
| FAM57B.3 | 1.61E-06 | -0.71571 | 0.439 | 0.462 | 0.035622 | 5 | *FAM57B* | Proliferating NRPCs |
| TTYH1.4 | 8.07E-16 | -0.78167 | 0.28 | 0.464 | 1.78E-11 | 5 | *TTYH1* | Proliferating NRPCs |
| TULP1.3 | 2.37E-20 | -0.78725 | 0.066 | 0.312 | 5.22E-16 | 5 | *TULP1* | Proliferating NRPCs |
| STMN2.3 | 1.91E-06 | -0.85149 | 0.301 | 0.396 | 0.04218 | 5 | *STMN2* | Proliferating NRPCs |
| CRABP2.1 | 1.23E-22 | -0.86471 | 0.36 | 0.577 | 2.73E-18 | 5 | *CRABP2* | Proliferating NRPCs |
| IFITM3.4 | 1.03E-16 | -0.88114 | 0.149 | 0.366 | 2.27E-12 | 5 | *IFITM3* | Proliferating NRPCs |
| TF.5 | 0.001707 | -0.88299 | 0.152 | 0.218 | 1 | 5 | *TF* | Proliferating NRPCs |
| UNC119.3 | 7.48E-10 | -0.89825 | 0.443 | 0.525 | 1.65E-05 | 5 | *UNC119* | Proliferating NRPCs |
| LAMP5.4 | 5.27E-11 | -0.91127 | 0.17 | 0.332 | 1.16E-06 | 5 | *LAMP5* | Proliferating NRPCs |
| AKAP9.4 | 1.46E-23 | -0.9637 | 0.502 | 0.666 | 3.23E-19 | 5 | *AKAP9* | Proliferating NRPCs |
| DCT.3 | 1.10E-16 | -1.00237 | 0.083 | 0.302 | 2.44E-12 | 5 | *DCT* | Proliferating NRPCs |
| CYP26A1.5 | 0.001018 | -1.03305 | 0.439 | 0.458 | 1 | 5 | *CYP26A1* | Proliferating NRPCs |
| SPP1.5 | 1.21E-12 | -1.12758 | 0.381 | 0.5 | 2.67E-08 | 5 | *SPP1* | Proliferating NRPCs |
| PDE6H.4 | 5.21E-14 | -1.16778 | 0.208 | 0.388 | 1.15E-09 | 5 | *PDE6H* | Proliferating NRPCs |
| GNB3.3 | 1.63E-20 | -1.16848 | 0.204 | 0.432 | 3.61E-16 | 5 | *GNB3* | Proliferating NRPCs |
| NRL.4 | 4.15E-05 | -1.18588 | 0.273 | 0.346 | 0.915554 | 5 | *NRL* | Proliferating NRPCs |
| AIPL1.3 | 5.89E-21 | -1.19783 | 0.097 | 0.344 | 1.30E-16 | 5 | *AIPL1* | Proliferating NRPCs |
| HIST1H4C. | 0.00147 | -1.28638 | 0.585 | 0.587 | 1 | 5 | *HIST1H4C* | Proliferating NRPCs |
| PDC.5 | 4.15E-15 | -1.40324 | 0.256 | 0.433 | 9.17E-11 | 5 | *PDC* | Proliferating NRPCs |
| SFRP2.4 | 1.21E-06 | -1.47361 | 0.571 | 0.545 | 0.026783 | 5 | *SFRP2* | Proliferating NRPCs |
| RCVRN.3 | 3.98E-19 | -1.74327 | 0.166 | 0.389 | 8.79E-15 | 5 | *RCVRN* | Proliferating NRPCs |
| VSX1 | 1.18E-141 | 1.86523 | 0.592 | 0.105 | 2.61E-137 | 6 | *VSX1* | Bipolar cells |
| OTX2.3 | 8.77E-143 | 1.300276 | 0.923 | 0.324 | 1.94E-138 | 6 | *OTX2* | Bipolar cells |
| NEUROD4. | 2.86E-135 | 1.28792 | 0.831 | 0.252 | 6.31E-131 | 6 | *NEUROD4* | Bipolar cells |
| CRYBG3 | 2.26E-116 | 1.190656 | 0.81 | 0.267 | 4.98E-112 | 6 | *CRYBG3* | Bipolar cells |
| CHODL | 2.52E-41 | 1.07678 | 0.514 | 0.203 | 5.57E-37 | 6 | *CHODL* | Bipolar cells |
| AMER2.3 | 3.24E-83 | 1.067454 | 0.901 | 0.473 | 7.16E-79 | 6 | *AMER2* | Bipolar cells |
| NEUROG1. | 1.32E-112 | 1.046135 | 0.725 | 0.191 | 2.92E-108 | 6 | *NEUROG1* | Bipolar cells |
| TAGLN3 | 1.41E-66 | 1.019528 | 0.768 | 0.365 | 3.12E-62 | 6 | *TAGLN3* | Bipolar cells |
| FABP7.3 | 5.77E-35 | 1.015265 | 0.835 | 0.6 | 1.27E-30 | 6 | *FABP7* | Bipolar cells |
| C11orf96 | 4.55E-60 | 0.988227 | 0.479 | 0.141 | 1.01E-55 | 6 | *C11orf96* | Bipolar cells |
| GSG1 | 4.10E-81 | 0.9667 | 0.458 | 0.1 | 9.06E-77 | 6 | *GSG1* | Bipolar cells |
| PCBP4.3 | 1.57E-93 | 0.964349 | 0.951 | 0.492 | 3.48E-89 | 6 | *PCBP4* | Bipolar cells |
| BTG1 | 1.31E-67 | 0.935751 | 0.958 | 0.79 | 2.90E-63 | 6 | *BTG1* | Bipolar cells |
| CADM3 | 4.12E-91 | 0.910185 | 0.775 | 0.286 | 9.10E-87 | 6 | *CADM3* | Bipolar cells |
| NEUROD1. | 1.19E-74 | 0.89668 | 0.912 | 0.406 | 2.63E-70 | 6 | *NEUROD1* | Bipolar cells |
| SOX4.2 | 4.65E-83 | 0.894418 | 0.986 | 0.858 | 1.03E-78 | 6 | *SOX4* | Bipolar cells |
| LINC00599 | 4.33E-72 | 0.886063 | 0.725 | 0.275 | 9.57E-68 | 6 | *LINC00599* | Bipolar cells |
| RCOR2 | 8.66E-72 | 0.869796 | 0.655 | 0.238 | 1.91E-67 | 6 | *RCOR2* | Bipolar cells |
| INSM1 | 4.25E-68 | 0.821869 | 0.718 | 0.28 | 9.39E-64 | 6 | *INSM1* | Bipolar cells |
| LHX4 | 9.79E-158 | 0.81521 | 0.553 | 0.077 | 2.16E-153 | 6 | *LHX4* | Bipolar cells |
| CADPS.3 | 1.85E-67 | 0.812205 | 0.768 | 0.315 | 4.08E-63 | 6 | *CADPS* | Bipolar cells |
| RASD1 | 2.26E-56 | 0.810679 | 0.581 | 0.21 | 4.99E-52 | 6 | *RASD1* | Bipolar cells |
| SLC38A5.3 | 1.51E-44 | 0.79263 | 0.687 | 0.307 | 3.34E-40 | 6 | *SLC38A5* | Bipolar cells |
| TPH1.3 | 1.84E-42 | 0.761194 | 0.546 | 0.206 | 4.06E-38 | 6 | *TPH1* | Bipolar cells |
| ATOH7 | 3.71E-09 | 0.737735 | 0.158 | 0.069 | 8.18E-05 | 6 | *ATOH7* | Bipolar cells |
| DLL3 | 8.84E-64 | 0.735013 | 0.592 | 0.202 | 1.95E-59 | 6 | *DLL3* | Bipolar cells |
| BTBD8 | 2.27E-60 | 0.723913 | 0.634 | 0.244 | 5.01E-56 | 6 | *BTBD8* | Bipolar cells |
| STC1 | 8.87E-67 | 0.718637 | 0.556 | 0.177 | 1.96E-62 | 6 | *STC1* | Bipolar cells |

| SERPINF1 | 2.01E-68 | 0.71467 | 0.736 | 0.306 | 4.44E-64 | 6 | *SERPINF1* | Bipolar cells |
| --- | --- | --- | --- | --- | --- | --- | --- | --- |
| SYP.3 | 2.92E-69 | 0.712117 | 0.842 | 0.36 | 6.45E-65 | 6 | *SYP* | Bipolar cells |
| NLK | 1.40E-58 | 0.703801 | 0.648 | 0.259 | 3.09E-54 | 6 | *NLK* | Bipolar cells |
| CYP1B1.2 | 6.68E-12 | -0.70103 | 0.229 | 0.405 | 1.48E-07 | 6 | *CYP1B1* | Bipolar cells |
| ZFP36L2.2 | 1.08E-17 | -0.70319 | 0.222 | 0.458 | 2.39E-13 | 6 | *ZFP36L2* | Bipolar cells |
| IGFBP7 | 9.65E-11 | -0.70422 | 0.056 | 0.214 | 2.13E-06 | 6 | *IGFBP7* | Bipolar cells |
| PTGDS.4 | 4.22E-09 | -0.71129 | 0.063 | 0.199 | 9.32E-05 | 6 | *PTGDS* | Bipolar cells |
| PTN.3 | 2.25E-12 | -0.71989 | 0.377 | 0.547 | 4.98E-08 | 6 | *PTN* | Bipolar cells |
| NUSAP1.3 | 1.91E-11 | -0.72498 | 0.169 | 0.344 | 4.22E-07 | 6 | *NUSAP1* | Bipolar cells |
| TTYH1.5 | 3.95E-14 | -0.74247 | 0.278 | 0.463 | 8.73E-10 | 6 | *TTYH1* | Bipolar cells |
| PTTG1.3 | 1.33E-10 | -0.74667 | 0.243 | 0.411 | 2.94E-06 | 6 | *PTTG1* | Bipolar cells |
| CRYM.1 | 2.09E-16 | -0.7468 | 0.095 | 0.315 | 4.61E-12 | 6 | *CRYM* | Bipolar cells |
| DHRS3.1 | 6.01E-25 | -0.76869 | 0.211 | 0.501 | 1.33E-20 | 6 | *DHRS3* | Bipolar cells |
| UBE2C.4 | 2.95E-08 | -0.78069 | 0.074 | 0.205 | 0.000652 | 6 | *UBE2C* | Bipolar cells |
| APOE.1 | 5.72E-10 | -0.78551 | 0.31 | 0.459 | 1.26E-05 | 6 | *APOE* | Bipolar cells |
| SAT1.1 | 2.88E-22 | -0.79991 | 0.359 | 0.593 | 6.35E-18 | 6 | *SAT1* | Bipolar cells |
| GPC3.4 | 5.00E-14 | -0.82337 | 0.151 | 0.356 | 1.10E-09 | 6 | *GPC3* | Bipolar cells |
| EGR1.2 | 1.67E-20 | -0.83209 | 0.363 | 0.575 | 3.69E-16 | 6 | *EGR1* | Bipolar cells |
| PRSS23.3 | 1.27E-23 | -0.89694 | 0.18 | 0.461 | 2.81E-19 | 6 | *PRSS23* | Bipolar cells |
| DIO3.4 | 2.51E-21 | -0.92306 | 0.06 | 0.322 | 5.54E-17 | 6 | *DIO3* | Bipolar cells |
| IFITM2.5 | 6.20E-21 | -0.93249 | 0.039 | 0.295 | 1.37E-16 | 6 | *IFITM2* | Bipolar cells |
| CENPF.4 | 5.58E-09 | -0.93328 | 0.158 | 0.309 | 0.000123 | 6 | *CENPF* | Bipolar cells |
| TOP2A.4 | 9.08E-11 | -0.94893 | 0.12 | 0.287 | 2.01E-06 | 6 | *TOP2A* | Bipolar cells |
| TF.6 | 1.07E-06 | -0.95144 | 0.106 | 0.22 | 0.023562 | 6 | *TF* | Bipolar cells |
| DKK3.4 | 4.64E-26 | -0.95851 | 0.211 | 0.502 | 1.02E-21 | 6 | *DKK3* | Bipolar cells |
| TRH.4 | 1.21E-11 | -0.98614 | 0.433 | 0.578 | 2.67E-07 | 6 | *TRH* | Bipolar cells |
| FOS.2 | 1.84E-23 | -1.00328 | 0.317 | 0.572 | 4.07E-19 | 6 | *FOS* | Bipolar cells |
| HES1.3 | 1.40E-30 | -1.00867 | 0.063 | 0.404 | 3.10E-26 | 6 | *HES1* | Bipolar cells |
| SOX2.4 | 3.07E-31 | -1.01281 | 0.092 | 0.427 | 6.78E-27 | 6 | *SOX2* | Bipolar cells |
| IFITM3.5 | 5.30E-26 | -1.0203 | 0.067 | 0.37 | 1.17E-21 | 6 | *IFITM3* | Bipolar cells |
| LAMP5.5 | 2.22E-15 | -1.04366 | 0.127 | 0.334 | 4.89E-11 | 6 | *LAMP5* | Bipolar cells |
| CLU.5 | 5.92E-31 | -1.08308 | 0.43 | 0.685 | 1.31E-26 | 6 | *CLU* | Bipolar cells |
| ZFP36L1.4 | 7.35E-34 | -1.11342 | 0.102 | 0.462 | 1.62E-29 | 6 | *ZFP36L1* | Bipolar cells |
| CCND1.2 | 2.91E-27 | -1.11869 | 0.155 | 0.468 | 6.42E-23 | 6 | *CCND1* | Bipolar cells |
| COL1A2.3 | 2.70E-24 | -1.18021 | 0.102 | 0.387 | 5.97E-20 | 6 | *COL1A2* | Bipolar cells |
| CRABP1.4 | 2.25E-20 | -1.24039 | 0.701 | 0.742 | 4.97E-16 | 6 | *CRABP1* | Bipolar cells |
| SPP1.6 | 5.59E-32 | -1.55038 | 0.19 | 0.511 | 1.24E-27 | 6 | *SPP1* | Bipolar cells |
| VIM.5 | 1.62E-42 | -1.81788 | 0.662 | 0.81 | 3.59E-38 | 6 | *VIM* | Bipolar cells |
| CYP26A1.6 | 8.77E-29 | -1.84414 | 0.162 | 0.473 | 1.94E-24 | 6 | *CYP26A1* | Bipolar cells |
| SFRP2.5 | 3.82E-38 | -2.06526 | 0.208 | 0.565 | 8.42E-34 | 6 | *SFRP2* | Bipolar cells |
| PDE6H.5 | 8.08E-119 | 1.710256 | 0.898 | 0.352 | 1.78E-114 | 7 | *PDE6H* | Cone precursors |
| MPP4.2 | 9.99E-179 | 1.282737 | 0.875 | 0.178 | 2.21E-174 | 7 | *MPP4* | Cone precursors |
| RRAD.4 | 3.00E-93 | 1.260529 | 0.82 | 0.294 | 6.63E-89 | 7 | *RRAD* | Cone precursors |
| GNGT2.1 | 1.10E-174 | 1.241843 | 0.816 | 0.156 | 2.44E-170 | 7 | *GNGT2* | Cone precursors |
| RS1.1 | 4.22E-179 | 1.216601 | 0.746 | 0.121 | 9.32E-175 | 7 | *RS1* | Cone precursors |
| PLEKHB1.2 | 7.15E-125 | 1.190411 | 0.902 | 0.308 | 1.58E-120 | 7 | *PLEKHB1* | Cone precursors |
| AKAP9.5 | 1.02E-108 | 1.179117 | 0.996 | 0.64 | 2.25E-104 | 7 | *AKAP9* | Cone precursors |
| SCG3.4 | 6.53E-103 | 1.177779 | 0.98 | 0.505 | 1.44E-98 | 7 | *SCG3* | Cone precursors |
| AIPL1.4 | 3.57E-119 | 1.169242 | 0.918 | 0.301 | 7.89E-115 | 7 | *AIPL1* | Cone precursors |
| ARR3.1 | 4.79E-96 | 1.159226 | 0.57 | 0.122 | 1.06E-91 | 7 | *ARR3* | Cone precursors |
| CLSTN2 | 2.09E-93 | 1.151089 | 0.672 | 0.184 | 4.62E-89 | 7 | *CLSTN2* | Cone precursors |
| VXN.3 | 3.21E-85 | 1.120652 | 0.742 | 0.231 | 7.08E-81 | 7 | *VXN* | Cone precursors |
| GUK1.1 | 1.74E-102 | 1.099935 | 0.973 | 0.772 | 3.83E-98 | 7 | *GUK1* | Cone precursors |
| PHOX2A.1 | 8.84E-132 | 1.091633 | 0.66 | 0.127 | 1.95E-127 | 7 | *PHOX2A* | Cone precursors |
| RTBDN.1 | 6.67E-164 | 1.084343 | 0.746 | 0.132 | 1.47E-159 | 7 | *RTBDN* | Cone precursors |
| PDC.6 | 2.66E-101 | 1.065479 | 0.969 | 0.397 | 5.87E-97 | 7 | *PDC* | Cone precursors |
| UNC119.4 | 1.12E-97 | 1.054839 | 0.965 | 0.498 | 2.48E-93 | 7 | *UNC119* | Cone precursors |
| FSTL5.2 | 3.07E-129 | 1.054306 | 0.906 | 0.255 | 6.78E-125 | 7 | *FSTL5* | Cone precursors |
| NTM | 1.57E-124 | 1.04159 | 0.824 | 0.215 | 3.47E-120 | 7 | *NTM* | Cone precursors |
| NRN1 | 1.99E-87 | 1.034127 | 0.82 | 0.293 | 4.39E-83 | 7 | *NRN1* | Cone precursors |

| GNB3.4 | 2.62E-94 | 1.032846 | 0.945 | 0.393 | 5.79E-90 | 7 | *GNB3* | Cone precursors |
| --- | --- | --- | --- | --- | --- | --- | --- | --- |
| TULP1.4 | 3.22E-122 | 1.017926 | 0.891 | 0.269 | 7.12E-118 | 7 | *TULP1* | Cone precursors |
| FAM57B.4 | 8.68E-95 | 1.0117 | 0.945 | 0.436 | 1.92E-90 | 7 | *FAM57B* | Cone precursors |
| CRABP2.2 | 4.01E-55 | 1.00217 | 0.883 | 0.549 | 8.86E-51 | 7 | *CRABP2* | Cone precursors |
| SCG5 | 3.66E-79 | 0.991826 | 0.73 | 0.248 | 8.09E-75 | 7 | *SCG5* | Cone precursors |
| TUBA4A.3 | 4.58E-77 | 0.989588 | 0.84 | 0.362 | 1.01E-72 | 7 | *TUBA4A* | Cone precursors |
| FAM107A | 2.30E-189 | 0.987843 | 0.84 | 0.152 | 5.07E-185 | 7 | *FAM107A* | Cone precursors |
| DPYSL3.2 | 3.32E-105 | 0.98524 | 0.895 | 0.314 | 7.33E-101 | 7 | *DPYSL3* | Cone precursors |
| MIR7-3HG. | 3.26E-98 | 0.985238 | 0.836 | 0.274 | 7.20E-94 | 7 | *MIR7-3HG* | Cone precursors |
| CRX.4 | 2.25E-97 | 0.96377 | 0.875 | 0.297 | 4.97E-93 | 7 | *CRX* | Cone precursors |
| PRCD.1 | 2.34E-113 | 0.962536 | 0.797 | 0.227 | 5.18E-109 | 7 | *PRCD* | Cone precursors |
| CPE.4 | 4.28E-78 | 0.955123 | 0.977 | 0.649 | 9.45E-74 | 7 | *CPE* | Cone precursors |
| KCNV2.1 | 3.12E-127 | 0.939643 | 0.777 | 0.172 | 6.88E-123 | 7 | *KCNV2* | Cone precursors |
| MAP2.2 | 5.32E-73 | 0.93953 | 0.914 | 0.519 | 1.18E-68 | 7 | *MAP2* | Cone precursors |
| SEZ6L2.1 | 1.30E-89 | 0.935071 | 0.898 | 0.394 | 2.87E-85 | 7 | *SEZ6L2* | Cone precursors |
| SLC38A5.4 | 2.35E-93 | 0.918714 | 0.891 | 0.299 | 5.18E-89 | 7 | *SLC38A5* | Cone precursors |
| SEPT4.4 | 8.49E-92 | 0.902311 | 0.918 | 0.368 | 1.87E-87 | 7 | *Sep-04* | Cone precursors |
| GUCA1A.1 | 3.81E-92 | 0.900375 | 0.66 | 0.169 | 8.40E-88 | 7 | *GUCA1A* | Cone precursors |
| IMPG2.1 | 2.76E-114 | 0.873769 | 0.812 | 0.211 | 6.09E-110 | 7 | *IMPG2* | Cone precursors |
| SLC38A1.1 | 3.28E-80 | 0.872536 | 0.93 | 0.467 | 7.24E-76 | 7 | *SLC38A1* | Cone precursors |
| CNTNAP2 | 6.57E-100 | 0.869345 | 0.805 | 0.245 | 1.45E-95 | 7 | *CNTNAP2* | Cone precursors |
| NEDD4L | 2.76E-98 | 0.862051 | 0.812 | 0.274 | 6.09E-94 | 7 | *NEDD4L* | Cone precursors |
| SLC17A7.1 | 3.10E-121 | 0.861188 | 0.809 | 0.21 | 6.83E-117 | 7 | *SLC17A7* | Cone precursors |
| PEX5L | 5.51E-97 | 0.860017 | 0.758 | 0.218 | 1.22E-92 | 7 | *PEX5L* | Cone precursors |
| OLFM1.1 | 6.78E-83 | 0.851961 | 0.828 | 0.31 | 1.50E-78 | 7 | *OLFM1* | Cone precursors |
| IGSF21.2 | 1.43E-73 | 0.846883 | 0.77 | 0.265 | 3.15E-69 | 7 | *IGSF21* | Cone precursors |
| MYL4 | 2.74E-128 | 0.822781 | 0.598 | 0.101 | 6.05E-124 | 7 | *MYL4* | Cone precursors |
| MAP1LC3A | 3.37E-77 | 0.82198 | 0.926 | 0.458 | 7.43E-73 | 7 | *MAP1LC3A* | Cone precursors |
| ENO3 | 3.67E-104 | 0.815764 | 0.699 | 0.179 | 8.11E-100 | 7 | *ENO3* | Cone precursors |
| ENO2.4 | 9.81E-72 | 0.809088 | 0.949 | 0.63 | 2.17E-67 | 7 | *ENO2* | Cone precursors |
| TMEM176 | 2.01E-53 | 0.791214 | 0.648 | 0.258 | 4.44E-49 | 7 | *TMEM176* | Cone precursors |
| ELFN1 | 1.38E-90 | 0.781442 | 0.66 | 0.168 | 3.05E-86 | 7 | *ELFN1* | Cone precursors |
| LINC00599 | 1.12E-82 | 0.778432 | 0.832 | 0.272 | 2.48E-78 | 7 | *LINC00599* | Cone precursors |
| MLXIP | 1.60E-70 | 0.774682 | 0.727 | 0.256 | 3.53E-66 | 7 | *MLXIP* | Cone precursors |
| SYP.4 | 1.55E-79 | 0.768028 | 0.906 | 0.36 | 3.42E-75 | 7 | *SYP* | Cone precursors |
| THY1.1 | 2.88E-76 | 0.763558 | 0.781 | 0.274 | 6.35E-72 | 7 | *THY1* | Cone precursors |
| RBP4.1 | 3.98E-83 | 0.761244 | 0.719 | 0.2 | 8.79E-79 | 7 | *RBP4* | Cone precursors |
| ATP1A3.3 | 4.48E-63 | 0.75978 | 0.805 | 0.324 | 9.90E-59 | 7 | *ATP1A3* | Cone precursors |
| CYP26B1 | 5.04E-45 | 0.759172 | 0.559 | 0.202 | 1.11E-40 | 7 | *CYP26B1* | Cone precursors |
| CC2D2A | 6.21E-91 | 0.75255 | 0.766 | 0.241 | 1.37E-86 | 7 | *CC2D2A* | Cone precursors |
| DHRS7 | 5.67E-53 | 0.748508 | 0.859 | 0.54 | 1.25E-48 | 7 | *DHRS7* | Cone precursors |
| SOX7 | 3.14E-125 | 0.748303 | 0.672 | 0.128 | 6.94E-121 | 7 | *SOX7* | Cone precursors |
| RYBP | 1.82E-59 | 0.743298 | 0.773 | 0.334 | 4.02E-55 | 7 | *RYBP* | Cone precursors |
| THRB | 1.79E-88 | 0.743165 | 0.609 | 0.15 | 3.95E-84 | 7 | *THRB* | Cone precursors |
| AC110716. | 5.93E-164 | 0.73675 | 0.57 | 0.07 | 1.31E-159 | 7 | *AC110716.* | Cone precursors |
| TMEM38A | 7.05E-79 | 0.72994 | 0.648 | 0.19 | 1.56E-74 | 7 | *TMEM38A* | Cone precursors |
| LMOD1 | 2.10E-117 | 0.719927 | 0.68 | 0.139 | 4.63E-113 | 7 | *LMOD1* | Cone precursors |
| CALCOCO2 | 1.31E-59 | 0.711626 | 0.805 | 0.377 | 2.88E-55 | 7 | *CALCOCO2* | Cone precursors |
| ARL6IP5 | 2.73E-59 | 0.707886 | 0.898 | 0.529 | 6.02E-55 | 7 | *ARL6IP5* | Cone precursors |
| RXRG.3 | 7.83E-72 | 0.706902 | 0.836 | 0.31 | 1.73E-67 | 7 | *RXRG* | Cone precursors |
| AKAP12.2 | 2.77E-20 | -0.71319 | 0.148 | 0.42 | 6.11E-16 | 7 | *AKAP12* | Cone precursors |
| PTPRZ1.1 | 2.18E-20 | -0.71399 | 0.059 | 0.328 | 4.80E-16 | 7 | *PTPRZ1* | Cone precursors |
| RTN4.2 | 2.23E-26 | -0.72076 | 0.934 | 0.913 | 4.92E-22 | 7 | *RTN4* | Cone precursors |
| NKAIN4 | 1.99E-27 | -0.72287 | 0.035 | 0.371 | 4.40E-23 | 7 | *NKAIN4* | Cone precursors |
| CD9.1 | 7.68E-18 | -0.72515 | 0.066 | 0.313 | 1.69E-13 | 7 | *CD9* | Cone precursors |
| CRYM.2 | 6.14E-11 | -0.73364 | 0.145 | 0.311 | 1.36E-06 | 7 | *CRYM* | Cone precursors |
| FABP5.1 | 1.51E-19 | -0.73389 | 0.387 | 0.58 | 3.34E-15 | 7 | *FABP5* | Cone precursors |
| TTYH1.6 | 5.70E-13 | -0.73914 | 0.289 | 0.462 | 1.26E-08 | 7 | *TTYH1* | Cone precursors |
| NUSAP1.4 | 1.97E-08 | -0.74104 | 0.211 | 0.341 | 0.000435 | 7 | *NUSAP1* | Cone precursors |
| PLEKHA1.1 | 6.64E-12 | -0.74395 | 0.555 | 0.626 | 1.47E-07 | 7 | *PLEKHA1* | Cone precursors |

| 5.61E-20 | -0.75252 | 0.703 | 0.759 | 1.24E-15 | 7 *B2M* | Cone precursors |
| --- | --- | --- | --- | --- | --- | --- |
| 7.48E-12 | -0.75884 | 0.469 | 0.59 | 1.65E-07 | 7 *SOX11* | Cone precursors |
| 2.62E-20 | -0.75933 | 0.074 | 0.343 | 5.79E-16 | 7 *COL18A1* | Cone precursors |
| 2.99E-22 | -0.77499 | 0.613 | 0.729 | 6.61E-18 | 7 *IER2* | Cone precursors |
| 3.71E-12 | -0.78089 | 0.27 | 0.434 | 8.20E-08 | 7 *PMAIP1* | Cone precursors |
| 1.41E-18 | -0.78106 | 0.223 | 0.456 | 3.10E-14 | 7 *ZFP36L2* | Cone precursors |
| 2.72E-22 | -0.78998 | 0.281 | 0.527 | 6.02E-18 | 7 *MEST* | Cone precursors |
| 8.07E-07 | -0.79897 | 0.086 | 0.204 | 0.017829 | 7 *UBE2C* | Cone precursors |
| 1.02E-31 | -0.82728 | 0.379 | 0.638 | 2.25E-27 | 7 *CNN3* | Cone precursors |
| 1.68E-29 | -0.82978 | 0.711 | 0.803 | 3.70E-25 | 7 *BTG1* | Cone precursors |
| 9.45E-20 | -0.8607 | 0.133 | 0.399 | 2.09E-15 | 7 *HES1* | Cone precursors |
| 2.54E-25 | -0.8607 | 0.109 | 0.424 | 5.60E-21 | 7 *PAX6* | Cone precursors |
| 2.90E-12 | -0.87051 | 0.406 | 0.533 | 6.40E-08 | 7 *DAPL1* | Cone precursors |
| 1.52E-17 | -0.88194 | 0.078 | 0.32 | 3.35E-13 | 7 *DIO3* | Cone precursors |
| 4.64E-19 | -0.89411 | 0.152 | 0.407 | 1.02E-14 | 7 *CYP1B1* | Cone precursors |
| 3.65E-22 | -0.8957 | 0.375 | 0.59 | 8.07E-18 | 7 *JUNB* | Cone precursors |
| 1.16E-15 | -0.89932 | 0.07 | 0.292 | 2.57E-11 | 7 *IFITM2* | Cone precursors |
| 0.000292 | -0.91605 | 0.137 | 0.218 | 1 | 7 *TF* | Cone precursors |
| 8.87E-19 | -0.95657 | 0.105 | 0.358 | 1.96E-14 | 7 *GPC3* | Cone precursors |
| 1.77E-24 | -0.96177 | 0.164 | 0.46 | 3.91E-20 | 7 *PRSS23* | Cone precursors |
| 5.14E-25 | -0.9756 | 0.203 | 0.501 | 1.14E-20 | 7 *DKK3* | Cone precursors |
| 4.37E-18 | -0.98501 | 0.66 | 0.734 | 9.65E-14 | 7 *HMGB2* | Cone precursors |
| 4.59E-20 | -0.99123 | 0.102 | 0.367 | 1.01E-15 | 7 *IFITM3* | Cone precursors |
| 1.68E-30 | -1.026 | 0.066 | 0.426 | 3.70E-26 | 7 *SOX2* | Cone precursors |
| 5.24E-11 | -1.03285 | 0.133 | 0.31 | 1.16E-06 | 7 *CENPF* | Cone precursors |
| 7.50E-12 | -1.04383 | 0.105 | 0.287 | 1.66E-07 | 7 *TOP2A* | Cone precursors |
| 7.07E-13 | -1.05575 | 0.535 | 0.616 | 1.56E-08 | 7 *FABP7* | Cone precursors |
| 2.66E-10 | -1.07152 | 0.402 | 0.531 | 5.88E-06 | 7 *IGFBP2* | Cone precursors |
| 3.03E-16 | -1.08655 | 0.219 | 0.443 | 6.70E-12 | 7 *IGFBP5* | Cone precursors |
| 1.58E-16 | -1.09222 | 0.105 | 0.334 | 3.49E-12 | 7 *LAMP5* | Cone precursors |
| 7.36E-30 | -1.09724 | 0.109 | 0.459 | 1.62E-25 | 7 *ZFP36L1* | Cone precursors |
| 6.99E-30 | -1.1175 | 0.293 | 0.577 | 1.54E-25 | 7 *EGR1* | Cone precursors |
| 7.18E-29 | -1.18764 | 0.121 | 0.468 | 1.59E-24 | 7 *CCND1* | Cone precursors |
| 8.76E-34 | -1.23086 | 0.188 | 0.555 | 1.93E-29 | 7 *PTN* | Cone precursors |
| 1.09E-26 | -1.23192 | 0.059 | 0.388 | 2.41E-22 | 7 *COL1A2* | Cone precursors |
| 1.08E-46 | -1.26176 | 0.406 | 0.735 | 2.38E-42 | 7 *MDK* | Cone precursors |
| 9.09E-34 | -1.35466 | 0.449 | 0.682 | 2.01E-29 | 7 *CLU* | Cone precursors |
| 2.45E-12 | -1.3834 | 0.484 | 0.575 | 5.41E-08 | 7 *TRH* | Cone precursors |
| 1.71E-35 | -1.40541 | 0.234 | 0.574 | 3.77E-31 | 7 *FOS* | Cone precursors |
| 2.25E-24 | -1.47162 | 0.238 | 0.507 | 4.97E-20 | 7 *SPP1* | Cone precursors |
| 2.72E-18 | -1.54683 | 0.336 | 0.557 | 6.02E-14 | 7 *SFRP2* | Cone precursors |
| 8.87E-15 | -1.63195 | 0.289 | 0.465 | 1.96E-10 | 7 *CYP26A1* | Cone precursors |
| 6.41E-50 | -1.95125 | 0.477 | 0.753 | 1.42E-45 | 7 *CRABP1* | Cone precursors |
| 1.24E-49 | -2.00704 | 0.645 | 0.81 | 2.74E-45 | 7 *VIM* | Cone precursors |
| 1.98E-85 | 0.980418 | 0.896 | 0.353 | 4.37E-81 | 8 *PCLAF* | Late RPCs |
| 6.67E-70 | 0.910884 | 0.918 | 0.404 | 1.47E-65 | 8 *PMAIP1* | Late RPCs |
| 2.97E-67 | 0.840809 | 0.957 | 0.609 | 6.56E-63 | 8 *TYMS* | Late RPCs |
| 2.87E-77 | 0.839821 | 0.961 | 0.54 | 6.35E-73 | 8 *TMEM106* | Late RPCs |
| 1.14E-56 | 0.823329 | 0.948 | 0.608 | 2.53E-52 | 8 *PLEKHA1* | Late RPCs |
| 1.90E-69 | 0.812111 | 0.918 | 0.512 | 4.19E-65 | 8 *SLBP* | Late RPCs |
| 1.50E-78 | 0.798977 | 0.823 | 0.281 | 3.31E-74 | 8 *FAM111B* | Late RPCs |
| 5.13E-64 | 0.748099 | 0.952 | 0.555 | 1.13E-59 | 8 *PCNA* | Late RPCs |
| 6.08E-10 | -0.70158 | 0.056 | 0.226 | 1.34E-05 | 8 *ROM1* | Late RPCs |
| 5.37E-12 | -0.7073 | 0.355 | 0.51 | 1.19E-07 | 8 *MEIS2* | Late RPCs |
| 9.30E-15 | -0.70841 | 0.013 | 0.228 | 2.05E-10 | 8 *NEUROG1* | Late RPCs |
| 2.62E-16 | -0.71967 | 0.403 | 0.596 | 5.79E-12 | 8 *ARL6IP1* | Late RPCs |
| 2.98E-18 | -0.72523 | 0.035 | 0.297 | 6.57E-14 | 8 *FSTL5* | Late RPCs |
| 6.56E-12 | -0.72893 | 0.069 | 0.263 | 1.45E-07 | 8 *VXN* | Late RPCs |
| 2.64E-19 | -0.75451 | 0.026 | 0.3 | 5.82E-15 | 8 *IGSF21* | Late RPCs |
| 8.89E-16 | -0.75474 | 0.069 | 0.309 | 1.96E-11 | 8 *TULP1* | Late RPCs |

| B2M.1 |
| --- |
| SOX11.2 |
| COL18A1.2 |
| IER2.1 |
| PMAIP1.2 |
| ZFP36L2.3 |
| MEST.2 |
| UBE2C.5 |
| CNN3 |
| BTG1.1 |
| HES1.4 |
| PAX6.2 |
| DAPL1.4 |
| DIO3.5 |
| CYP1B1.3 |
| JUNB.1 |
| IFITM2.6 |
| TF.7 |
| GPC3.5 |
| PRSS23.4 |
| DKK3.5 |
| HMGB2.4 |
| IFITM3.6 |
| SOX2.5 |
| CENPF.5 |
| TOP2A.5 |
| FABP7.4 |
| IGFBP2.2 |
| IGFBP5.3 |
| LAMP5.6 |
| ZFP36L1.5 |
| EGR1.3 |
| CCND1.3 |
| PTN.4 |
| COL1A2.4 |
| MDK.3 |
| CLU.6 |
| TRH.5 |
| FOS.3 |
| SPP1.7 |
| SFRP2.6 |
| CYP26A1.7 |
| CRABP1.5 |
| VIM.6 |
| PCLAF.1 |
| PMAIP1.3 |
| TYMS.2 |
| TMEM106 |
| PLEKHA1.2 |
| SLBP.1 |
| FAM111B. |
| PCNA.1 |
| ROM1.2 |
| MEIS2.2 |
| NEUROG1. |
| ARL6IP1.1 |
| FSTL5.3 |
| VXN.4 |
| IGSF21.3 |
| TULP1.5 |

DPYSL3.3 LINC00599 CADPS.4 CHCHD2.2 TPH1.4 RXRG.4 SYP.5 SEPT4.5 CRX.5 ATP1A3.4 OTX2.4 AMER2.4 SLC38A5.5 AKAP9.6 RRAD.5 PCBP4.4 UNC119.5 DCT.4 CPE.5 NEUROD1. FAM57B.5 AIPL1.5 GNB3.5 SCG3.5 PDE6H.6 STMN2.4 HIST1H4C. NRL.5 PDC.7 RCVRN.4 HES6.2 GADD45A. ATOH7.1 MIAT.1 CRABP1.6 SOX11.3 CLDN5 DLK1.1 CDKN1C RBP1 SOX4.3 RXRG.5 TPH1.5 PLEKHB1.3 TULP1.6 AKAP9.7 CRABP2.3 APOE.2 UNC119.6 SLC38A5.6 CRX.6 DCT.5 SFRP2.7 AIPL1.6 HIST1H4C. GNB3.6 PDE6H.7 NRL.6 PDC.8 RCVRN.5

| 1.23E-18 | -0.75536 | 0.082 | 0.353 | 2.71E-14 | 8 | *DPYSL3* | Late RPCs |
| --- | --- | --- | --- | --- | --- | --- | --- |
| 1.14E-20 | -0.75878 | 0.022 | 0.311 | 2.51E-16 | 8 | *LINC00599* | Late RPCs |
| 2.18E-19 | -0.77277 | 0.069 | 0.35 | 4.82E-15 | 8 | *CADPS* | Late RPCs |
| 2.38E-15 | -0.78554 | 0.177 | 0.447 | 5.25E-11 | 8 | *CHCHD2* | Late RPCs |
| 1.34E-13 | -0.8052 | 0.026 | 0.233 | 2.97E-09 | 8 | *TPH1* | Late RPCs |
| 5.03E-20 | -0.8146 | 0.061 | 0.347 | 1.11E-15 | 8 | *RXRG* | Late RPCs |
| 9.66E-23 | -0.82543 | 0.082 | 0.399 | 2.13E-18 | 8 | *SYP* | Late RPCs |
| 4.93E-14 | -0.82616 | 0.195 | 0.403 | 1.09E-09 | 8 | *Sep-04* | Late RPCs |
| 3.37E-17 | -0.84839 | 0.082 | 0.336 | 7.44E-13 | 8 | *CRX* | Late RPCs |
| 2.49E-23 | -0.8949 | 0.043 | 0.36 | 5.49E-19 | 8 | *ATP1A3* | Late RPCs |
| 1.17E-21 | -0.90713 | 0.069 | 0.368 | 2.58E-17 | 8 | *OTX2* | Late RPCs |
| 2.97E-18 | -0.94096 | 0.268 | 0.506 | 6.55E-14 | 8 | *AMER2* | Late RPCs |
| 6.84E-19 | -0.94918 | 0.065 | 0.338 | 1.51E-14 | 8 | *SLC38A5* | Late RPCs |
| 1.61E-18 | -0.97487 | 0.519 | 0.663 | 3.55E-14 | 8 | *AKAP9* | Late RPCs |
| 1.36E-10 | -0.98466 | 0.147 | 0.326 | 3.00E-06 | 8 | *RRAD* | Late RPCs |
| 1.24E-21 | -1.01198 | 0.268 | 0.527 | 2.73E-17 | 8 | *PCBP4* | Late RPCs |
| 2.83E-16 | -1.05203 | 0.325 | 0.529 | 6.26E-12 | 8 | *UNC119* | Late RPCs |
| 2.86E-16 | -1.07124 | 0.056 | 0.301 | 6.32E-12 | 8 | *DCT* | Late RPCs |
| 4.65E-28 | -1.08823 | 0.433 | 0.675 | 1.03E-23 | 8 | *CPE* | Late RPCs |
| 3.06E-24 | -1.11352 | 0.126 | 0.447 | 6.75E-20 | 8 | *NEUROD1* | Late RPCs |
| 1.58E-25 | -1.15546 | 0.16 | 0.474 | 3.49E-21 | 8 | *FAM57B* | Late RPCs |
| 3.92E-19 | -1.19771 | 0.069 | 0.342 | 8.66E-15 | 8 | *AIPL1* | Late RPCs |
| 7.31E-22 | -1.3006 | 0.147 | 0.432 | 1.61E-17 | 8 | *GNB3* | Late RPCs |
| 1.02E-20 | -1.31779 | 0.338 | 0.536 | 2.26E-16 | 8 | *SCG3* | Late RPCs |
| 1.93E-14 | -1.35448 | 0.173 | 0.388 | 4.25E-10 | 8 | *PDE6H* | Late RPCs |
| 1.09E-23 | -1.38077 | 0.087 | 0.404 | 2.41E-19 | 8 | *STMN2* | Late RPCs |
| 2.80E-07 | -1.44378 | 0.489 | 0.591 | 0.006174 | 8 | *HIST1H4C* | Late RPCs |
| 5.09E-18 | -1.55746 | 0.091 | 0.353 | 1.12E-13 | 8 | *NRL* | Late RPCs |
| 1.18E-14 | -1.58327 | 0.234 | 0.432 | 2.61E-10 | 8 | *PDC* | Late RPCs |
| 4.51E-17 | -1.72371 | 0.139 | 0.388 | 9.96E-13 | 8 | *RCVRN* | Late RPCs |
| 8.29E-25 | 1.253665 | 0.876 | 0.651 | 1.83E-20 | 9 | *HES6* | NRPCs/T1 |
| 1.01E-48 | 1.249128 | 0.831 | 0.515 | 2.23E-44 | 9 | *GADD45A* | NRPCs/T1 |
| 1.21E-17 | 1.174489 | 0.213 | 0.068 | 2.68E-13 | 9 | *ATOH7* | NRPCs/T1 |
| 1.16E-60 | 1.047967 | 0.822 | 0.385 | 2.56E-56 | 9 | *MIAT* | NRPCs/T1 |
| 1.20E-34 | 0.923022 | 0.96 | 0.73 | 2.66E-30 | 9 | *CRABP1* | NRPCs/T1 |
| 4.62E-30 | 0.886174 | 0.773 | 0.576 | 1.02E-25 | 9 | *SOX11* | NRPCs/T1 |
| 9.08E-11 | 0.778322 | 0.298 | 0.156 | 2.01E-06 | 9 | *CLDN5* | NRPCs/T1 |
| 1.70E-08 | 0.768445 | 0.28 | 0.157 | 0.000376 | 9 | *DLK1* | NRPCs/T1 |
| 3.21E-06 | 0.757726 | 0.342 | 0.235 | 0.070985 | 9 | *CDKN1C* | NRPCs/T1 |
| 1.94E-43 | 0.704687 | 0.987 | 0.912 | 4.29E-39 | 9 | *RBP1* | NRPCs/T1 |
| 3.94E-24 | 0.703129 | 0.938 | 0.861 | 8.71E-20 | 9 | *SOX4* | NRPCs/T1 |
| 5.12E-14 | -0.70595 | 0.116 | 0.344 | 1.13E-09 | 9 | *RXRG* | NRPCs/T1 |
| 1.56E-09 | -0.7558 | 0.067 | 0.231 | 3.45E-05 | 9 | *TPH1* | NRPCs/T1 |
| 1.03E-19 | -0.77751 | 0.058 | 0.348 | 2.28E-15 | 9 | *PLEKHB1* | NRPCs/T1 |
| 3.99E-18 | -0.78502 | 0.04 | 0.31 | 8.82E-14 | 9 | *TULP1* | NRPCs/T1 |
| 4.41E-13 | -0.80463 | 0.529 | 0.663 | 9.74E-09 | 9 | *AKAP9* | NRPCs/T1 |
| 7.61E-18 | -0.8109 | 0.329 | 0.575 | 1.68E-13 | 9 | *CRABP2* | NRPCs/T1 |
| 5.01E-16 | -0.85927 | 0.2 | 0.462 | 1.11E-11 | 9 | *APOE* | NRPCs/T1 |
| 1.66E-12 | -0.88157 | 0.324 | 0.529 | 3.67E-08 | 9 | *UNC119* | NRPCs/T1 |
| 2.66E-16 | -0.8894 | 0.084 | 0.337 | 5.87E-12 | 9 | *SLC38A5* | NRPCs/T1 |
| 1.51E-19 | -0.89349 | 0.053 | 0.337 | 3.33E-15 | 9 | *CRX* | NRPCs/T1 |
| 7.59E-15 | -1.04068 | 0.067 | 0.3 | 1.68E-10 | 9 | *DCT* | NRPCs/T1 |
| 0.000249 | -1.13555 | 0.524 | 0.547 | 1 | 9 | *SFRP2* | NRPCs/T1 |
| 1.32E-19 | -1.19518 | 0.058 | 0.342 | 2.90E-15 | 9 | *AIPL1* | NRPCs/T1 |
| 0.000632 | -1.19833 | 0.502 | 0.591 | 1 | 9 | *HIST1H4C* | NRPCs/T1 |
| 5.92E-18 | -1.19865 | 0.178 | 0.43 | 1.31E-13 | 9 | *GNB3* | NRPCs/T1 |
| 1.31E-12 | -1.20242 | 0.178 | 0.387 | 2.88E-08 | 9 | *PDE6H* | NRPCs/T1 |
| 3.44E-08 | -1.3413 | 0.2 | 0.348 | 0.000759 | 9 | *NRL* | NRPCs/T1 |
| 4.80E-17 | -1.63349 | 0.196 | 0.434 | 1.06E-12 | 9 | *PDC* | NRPCs/T1 |
| 9.45E-17 | -1.72016 | 0.138 | 0.387 | 2.09E-12 | 9 | *RCVRN* | NRPCs/T1 |

| DAPL1.5 | 2.30E-103 | 1.462827 | 0.977 | 0.508 | 5.09E-99 | 10 | *DAPL1* | Late RPCs |
| --- | --- | --- | --- | --- | --- | --- | --- | --- |
| SFRP2.8 | 7.90E-92 | 1.264887 | 0.991 | 0.527 | 1.75E-87 | 10 | *SFRP2* | Late RPCs |
| CYP1B1.4 | 4.50E-53 | 1.15757 | 0.795 | 0.378 | 9.93E-49 | 10 | *CYP1B1* | Late RPCs |
| RDH10 | 4.17E-85 | 1.022639 | 0.755 | 0.232 | 9.21E-81 | 10 | *RDH10* | Late RPCs |
| IFITM3.7 | 2.42E-73 | 1.01859 | 0.868 | 0.332 | 5.34E-69 | 10 | *IFITM3* | Late RPCs |
| HSD17B2 | 8.08E-99 | 0.95404 | 0.445 | 0.062 | 1.78E-94 | 10 | *HSD17B2* | Late RPCs |
| LRRC17 | 1.17E-177 | 0.944114 | 0.614 | 0.066 | 2.57E-173 | 10 | *LRRC17* | Late RPCs |
| ALDH1A1 | 1.89E-61 | 0.88997 | 0.55 | 0.148 | 4.17E-57 | 10 | *ALDH1A1* | Late RPCs |
| CCND1.4 | 7.13E-52 | 0.856645 | 0.905 | 0.432 | 1.57E-47 | 10 | *CCND1* | Late RPCs |
| TSC22D1 | 1.33E-38 | 0.799573 | 0.914 | 0.628 | 2.93E-34 | 10 | *TSC22D1* | Late RPCs |
| ZFP36L1.6 | 1.30E-61 | 0.777932 | 0.941 | 0.422 | 2.88E-57 | 10 | *ZFP36L1* | Late RPCs |
| FOS.4 | 3.01E-39 | 0.777331 | 0.886 | 0.544 | 6.66E-35 | 10 | *FOS* | Late RPCs |
| PSAT1 | 2.82E-41 | 0.747712 | 0.641 | 0.277 | 6.23E-37 | 10 | *PSAT1* | Late RPCs |
| DKK3.6 | 7.27E-59 | 0.73019 | 0.945 | 0.468 | 1.61E-54 | 10 | *DKK3* | Late RPCs |
| MGARP.1 | 6.24E-59 | 0.727106 | 0.736 | 0.264 | 1.38E-54 | 10 | *MGARP* | Late RPCs |
| IGFBP5.4 | 6.29E-28 | 0.72145 | 0.755 | 0.419 | 1.39E-23 | 10 | *IGFBP5* | Late RPCs |
| PAX6.3 | 1.46E-61 | 0.720634 | 0.882 | 0.389 | 3.22E-57 | 10 | *PAX6* | Late RPCs |
| NUDT4 | 3.86E-53 | 0.71697 | 0.814 | 0.392 | 8.52E-49 | 10 | *NUDT4* | Late RPCs |
| DIO3.6 | 3.21E-44 | 0.703086 | 0.718 | 0.291 | 7.10E-40 | 10 | *DIO3* | Late RPCs |
| UBE2T.1 | 1.41E-13 | -0.70131 | 0.291 | 0.488 | 3.12E-09 | 10 | *UBE2T* | Late RPCs |
| STMN1.2 | 2.63E-25 | -0.70198 | 0.968 | 0.963 | 5.80E-21 | 10 | *STMN1* | Late RPCs |
| NEUROD4. | 4.03E-19 | -0.70916 | 0.014 | 0.294 | 8.89E-15 | 10 | *NEUROD4* | Late RPCs |
| TAGLN3.1 | 2.22E-18 | -0.71528 | 0.118 | 0.397 | 4.91E-14 | 10 | *TAGLN3* | Late RPCs |
| NEUROG1. | 1.56E-14 | -0.71587 | 0.009 | 0.228 | 3.44E-10 | 10 | *NEUROG1* | Late RPCs |
| AKAP9.8 | 8.93E-08 | -0.72315 | 0.632 | 0.658 | 0.001972 | 10 | *AKAP9* | Late RPCs |
| IGSF21.4 | 9.96E-16 | -0.72336 | 0.055 | 0.299 | 2.20E-11 | 10 | *IGSF21* | Late RPCs |
| FSTL5.4 | 3.04E-19 | -0.72913 | 0.014 | 0.297 | 6.72E-15 | 10 | *FSTL5* | Late RPCs |
| LINC00599 | 3.12E-18 | -0.74499 | 0.036 | 0.31 | 6.89E-14 | 10 | *LINC00599* | Late RPCs |
| SOX4.4 | 6.96E-11 | -0.74973 | 0.877 | 0.864 | 1.54E-06 | 10 | *SOX4* | Late RPCs |
| CADPS.5 | 1.34E-16 | -0.74985 | 0.091 | 0.349 | 2.95E-12 | 10 | *CADPS* | Late RPCs |
| CRMP1.1 | 4.87E-22 | -0.75881 | 0.127 | 0.44 | 1.08E-17 | 10 | *CRMP1* | Late RPCs |
| MAP1LC3A | 3.52E-17 | -0.7612 | 0.245 | 0.491 | 7.76E-13 | 10 | *MAP1LC3A* | Late RPCs |
| TULP1.7 | 4.75E-16 | -0.77104 | 0.059 | 0.309 | 1.05E-11 | 10 | *TULP1* | Late RPCs |
| VXN.5 | 4.82E-13 | -0.7793 | 0.055 | 0.264 | 1.06E-08 | 10 | *VXN* | Late RPCs |
| TUBB4B.3 | 8.66E-20 | -0.78585 | 0.786 | 0.831 | 1.91E-15 | 10 | *TUBB4B* | Late RPCs |
| HMGA1.3 | 1.14E-14 | -0.78798 | 0.518 | 0.661 | 2.51E-10 | 10 | *HMGA1* | Late RPCs |
| RXRG.6 | 7.57E-17 | -0.79334 | 0.086 | 0.345 | 1.67E-12 | 10 | *RXRG* | Late RPCs |
| HMGB2.5 | 3.53E-08 | -0.80619 | 0.727 | 0.731 | 0.00078 | 10 | *HMGB2* | Late RPCs |
| DPYSL3.4 | 8.98E-22 | -0.80686 | 0.041 | 0.354 | 1.98E-17 | 10 | *DPYSL3* | Late RPCs |
| TPH1.6 | 2.44E-13 | -0.8123 | 0.023 | 0.233 | 5.38E-09 | 10 | *TPH1* | Late RPCs |
| NUSAP1.5 | 2.51E-12 | -0.81539 | 0.132 | 0.343 | 5.54E-08 | 10 | *NUSAP1* | Late RPCs |
| UBE2S.3 | 5.75E-18 | -0.82097 | 0.518 | 0.672 | 1.27E-13 | 10 | *UBE2S* | Late RPCs |
| CHCHD2.3 | 4.69E-13 | -0.85011 | 0.232 | 0.445 | 1.04E-08 | 10 | *CHCHD2* | Late RPCs |
| GADD45G. | 1.34E-18 | -0.85385 | 0.205 | 0.478 | 2.96E-14 | 10 | *GADD45G* | Late RPCs |
| TUBA4A.4 | 6.20E-21 | -0.85823 | 0.091 | 0.397 | 1.37E-16 | 10 | *TUBA4A* | Late RPCs |
| SEPT4.6 | 4.32E-14 | -0.85842 | 0.191 | 0.403 | 9.55E-10 | 10 | *Sep-04* | Late RPCs |
| ATP1A3.5 | 6.19E-22 | -0.88674 | 0.045 | 0.359 | 1.37E-17 | 10 | *ATP1A3* | Late RPCs |
| OTX2.5 | 4.57E-20 | -0.88793 | 0.073 | 0.367 | 1.01E-15 | 10 | *OTX2* | Late RPCs |
| TOP2A.6 | 9.21E-06 | -0.89266 | 0.164 | 0.283 | 0.203304 | 10 | *TOP2A* | Late RPCs |
| TUBB.1 | 1.87E-32 | -0.893 | 0.955 | 0.948 | 4.13E-28 | 10 | *TUBB* | Late RPCs |
| UBE2C.6 | 2.51E-09 | -0.89973 | 0.045 | 0.205 | 5.54E-05 | 10 | *UBE2C* | Late RPCs |
| ENO2.5 | 9.11E-25 | -0.90413 | 0.414 | 0.655 | 2.01E-20 | 10 | *ENO2* | Late RPCs |
| SYP.6 | 7.64E-26 | -0.90482 | 0.045 | 0.4 | 1.69E-21 | 10 | *SYP* | Late RPCs |
| LAMP5.7 | 4.02E-06 | -0.90648 | 0.214 | 0.328 | 0.088741 | 10 | *LAMP5* | Late RPCs |
| CRX.7 | 7.18E-19 | -0.90853 | 0.059 | 0.336 | 1.59E-14 | 10 | *CRX* | Late RPCs |
| CENPF.6 | 8.25E-06 | -0.92649 | 0.186 | 0.306 | 0.182212 | 10 | *CENPF* | Late RPCs |
| UNC119.7 | 3.29E-11 | -0.96425 | 0.377 | 0.527 | 7.25E-07 | 10 | *UNC119* | Late RPCs |
| MAP2.3 | 4.62E-24 | -0.96616 | 0.25 | 0.55 | 1.02E-19 | 10 | *MAP2* | Late RPCs |
| SLC38A5.7 | 1.75E-20 | -0.99037 | 0.041 | 0.339 | 3.86E-16 | 10 | *SLC38A5* | Late RPCs |
| RRAD.6 | 9.69E-12 | -1.03907 | 0.127 | 0.327 | 2.14E-07 | 10 | *RRAD* | Late RPCs |

| DCT.6 | 4.56E-13 | -1.05054 | 0.086 | 0.299 | 1.01E-08 | 10 | *DCT* | Late RPCs |
| --- | --- | --- | --- | --- | --- | --- | --- | --- |
| AMER2.5 | 6.53E-26 | -1.09656 | 0.182 | 0.509 | 1.44E-21 | 10 | *AMER2* | Late RPCs |
| PCBP4.5 | 1.53E-25 | -1.10494 | 0.218 | 0.528 | 3.37E-21 | 10 | *PCBP4* | Late RPCs |
| HES6.3 | 2.53E-27 | -1.20449 | 0.405 | 0.671 | 5.59E-23 | 10 | *HES6* | Late RPCs |
| FAM57B.6 | 1.22E-27 | -1.20475 | 0.123 | 0.475 | 2.69E-23 | 10 | *FAM57B* | Late RPCs |
| AIPL1.7 | 1.00E-17 | -1.20852 | 0.077 | 0.341 | 2.21E-13 | 10 | *AIPL1* | Late RPCs |
| NEUROD1. | 8.34E-28 | -1.26895 | 0.086 | 0.447 | 1.84E-23 | 10 | *NEUROD1* | Late RPCs |
| PDE6H.8 | 3.73E-12 | -1.30312 | 0.191 | 0.386 | 8.25E-08 | 10 | *PDE6H* | Late RPCs |
| GNB3.7 | 9.23E-25 | -1.33833 | 0.1 | 0.433 | 2.04E-20 | 10 | *GNB3* | Late RPCs |
| STMN2.5 | 1.62E-22 | -1.34891 | 0.082 | 0.404 | 3.57E-18 | 10 | *STMN2* | Late RPCs |
| HIST1H4C. | 1.67E-08 | -1.49753 | 0.482 | 0.591 | 0.000369 | 10 | *HIST1H4C* | Late RPCs |
| SCG3.6 | 5.49E-33 | -1.50635 | 0.159 | 0.543 | 1.21E-28 | 10 | *SCG3* | Late RPCs |
| NRL.7 | 9.31E-15 | -1.52058 | 0.118 | 0.352 | 2.06E-10 | 10 | *NRL* | Late RPCs |
| PDC.9 | 2.99E-17 | -1.69695 | 0.195 | 0.434 | 6.61E-13 | 10 | *PDC* | Late RPCs |
| RCVRN.6 | 1.63E-18 | -1.7616 | 0.114 | 0.388 | 3.61E-14 | 10 | *RCVRN* | Late RPCs |
| STMN2.6 | 5.05E-131 | 1.939544 | 0.976 | 0.367 | 1.11E-126 | 11 | *STMN2* | Retinal ganglion cells |
| HOXB5 | 4.32E-68 | 1.885553 | 0.517 | 0.129 | 9.53E-64 | 11 | *HOXB5* | Retinal ganglion cells |
| SOX4.5 | 1.59E-94 | 1.604477 | 0.995 | 0.859 | 3.51E-90 | 11 | *SOX4* | Retinal ganglion cells |
| RTN1 | 6.40E-168 | 1.568255 | 0.848 | 0.168 | 1.41E-163 | 11 | *RTN1* | Retinal ganglion cells |
| HOXB8 | 5.51E-86 | 1.557068 | 0.455 | 0.078 | 1.22E-81 | 11 | *HOXB8* | Retinal ganglion cells |
| TUBB2A | 1.89E-103 | 1.469681 | 0.953 | 0.45 | 4.18E-99 | 11 | *TUBB2A* | Retinal ganglion cells |
| TUBA1A | 2.82E-102 | 1.41268 | 0.995 | 0.944 | 6.22E-98 | 11 | *TUBA1A* | Retinal ganglion cells |
| BASP1.2 | 9.97E-102 | 1.338169 | 0.986 | 0.712 | 2.20E-97 | 11 | *BASP1* | Retinal ganglion cells |
| HOXB6 | 5.26E-78 | 1.296512 | 0.431 | 0.075 | 1.16E-73 | 11 | *HOXB6* | Retinal ganglion cells |
| CD24.1 | 1.61E-105 | 1.250244 | 0.962 | 0.433 | 3.56E-101 | 11 | *CD24* | Retinal ganglion cells |
| TUBB2B.1 | 2.70E-95 | 1.241588 | 0.995 | 0.827 | 5.97E-91 | 11 | *TUBB2B* | Retinal ganglion cells |
| HOXB2 | 1.01E-55 | 1.220468 | 0.649 | 0.238 | 2.22E-51 | 11 | *HOXB2* | Retinal ganglion cells |
| SYT4 | 7.03E-103 | 1.217633 | 0.654 | 0.141 | 1.55E-98 | 11 | *SYT4* | Retinal ganglion cells |
| RND3 | 1.08E-97 | 1.190874 | 0.682 | 0.161 | 2.38E-93 | 11 | *RND3* | Retinal ganglion cells |
| DCX | 6.66E-151 | 1.181623 | 0.848 | 0.169 | 1.47E-146 | 11 | *DCX* | Retinal ganglion cells |
| NSG1 | 1.43E-279 | 1.180263 | 0.744 | 0.058 | 3.16E-275 | 11 | *NSG1* | Retinal ganglion cells |
| STMN4 | 8.79E-92 | 1.178343 | 0.815 | 0.259 | 1.94E-87 | 11 | *STMN4* | Retinal ganglion cells |
| PCSK1N | 8.31E-102 | 1.153804 | 0.957 | 0.435 | 1.83E-97 | 11 | *PCSK1N* | Retinal ganglion cells |
| NOVA1 | 8.49E-86 | 1.147629 | 0.796 | 0.264 | 1.87E-81 | 11 | *NOVA1* | Retinal ganglion cells |
| RBFOX2 | 4.54E-154 | 1.146533 | 0.825 | 0.174 | 1.00E-149 | 11 | *RBFOX2* | Retinal ganglion cells |
| PAX6.4 | 4.60E-20 | 1.119491 | 0.592 | 0.402 | 1.02E-15 | 11 | *PAX6* | Retinal ganglion cells |
| HOXB9 | 3.23E-43 | 1.113895 | 0.242 | 0.04 | 7.14E-39 | 11 | *HOXB9* | Retinal ganglion cells |
| MAB21L2 | 6.90E-118 | 1.112501 | 0.531 | 0.077 | 1.52E-113 | 11 | *MAB21L2* | Retinal ganglion cells |
| MAB21L1 | 7.54E-35 | 1.10877 | 0.863 | 0.63 | 1.67E-30 | 11 | *MAB21L1* | Retinal ganglion cells |
| SOX11.4 | 4.28E-55 | 1.065513 | 0.919 | 0.571 | 9.45E-51 | 11 | *SOX11* | Retinal ganglion cells |
| KIF5C | 1.76E-85 | 1.03632 | 0.882 | 0.379 | 3.89E-81 | 11 | *KIF5C* | Retinal ganglion cells |
| ELAVL3 | 9.24E-122 | 1.035875 | 0.877 | 0.234 | 2.04E-117 | 11 | *ELAVL3* | Retinal ganglion cells |
| GAP43.1 | 2.53E-68 | 1.020418 | 0.749 | 0.261 | 5.59E-64 | 11 | *GAP43* | Retinal ganglion cells |
| MLLT11.1 | 3.46E-88 | 1.015959 | 0.981 | 0.678 | 7.64E-84 | 11 | *MLLT11* | Retinal ganglion cells |
| CITED2 | 8.76E-71 | 1.005839 | 0.725 | 0.244 | 1.93E-66 | 11 | *CITED2* | Retinal ganglion cells |
| ELAVL4 | 3.38E-86 | 0.984783 | 0.507 | 0.095 | 7.46E-82 | 11 | *ELAVL4* | Retinal ganglion cells |
| JPT1 | 9.30E-72 | 0.982673 | 0.967 | 0.66 | 2.05E-67 | 11 | *JPT1* | Retinal ganglion cells |
| GNG3 | 4.80E-143 | 0.964417 | 0.744 | 0.136 | 1.06E-138 | 11 | *GNG3* | Retinal ganglion cells |
| CELF4 | 1.02E-73 | 0.961949 | 0.839 | 0.32 | 2.26E-69 | 11 | *CELF4* | Retinal ganglion cells |
| NREP | 3.54E-71 | 0.929607 | 0.986 | 0.686 | 7.82E-67 | 11 | *NREP* | Retinal ganglion cells |
| PTF1A | 3.81E-87 | 0.926069 | 0.185 | 0.009 | 8.41E-83 | 11 | *PTF1A* | Retinal ganglion cells |
| NRXN1 | 1.65E-77 | 0.916289 | 0.725 | 0.222 | 3.64E-73 | 11 | *NRXN1* | Retinal ganglion cells |
| MIR124-2H | 1.11E-72 | 0.904712 | 0.744 | 0.248 | 2.45E-68 | 11 | *MIR124-2H* | Retinal ganglion cells |
| HOXB3 | 2.29E-80 | 0.904104 | 0.564 | 0.127 | 5.06E-76 | 11 | *HOXB3* | Retinal ganglion cells |
| CRMP1.2 | 1.78E-80 | 0.896957 | 0.938 | 0.406 | 3.93E-76 | 11 | *CRMP1* | Retinal ganglion cells |
| SNCA | 1.87E-39 | 0.891359 | 0.531 | 0.198 | 4.13E-35 | 11 | *SNCA* | Retinal ganglion cells |
| NSG2 | 6.97E-147 | 0.882591 | 0.664 | 0.099 | 1.54E-142 | 11 | *NSG2* | Retinal ganglion cells |
| BEX2 | 4.28E-64 | 0.864779 | 0.953 | 0.664 | 9.46E-60 | 11 | *BEX2* | Retinal ganglion cells |
| GRIA4 | 2.26E-79 | 0.863394 | 0.555 | 0.121 | 5.00E-75 | 11 | *GRIA4* | Retinal ganglion cells |
| RUNX1T1 | 1.39E-66 | 0.849205 | 0.559 | 0.147 | 3.06E-62 | 11 | *RUNX1T1* | Retinal ganglion cells |

| TAGLN3.2 | 1.71E-67 | 0.849197 | 0.863 | 0.367 | 3.76E-63 | 11 | *TAGLN3* | Retinal ganglion cells |
| --- | --- | --- | --- | --- | --- | --- | --- | --- |
| AP1S2 | 3.06E-37 | 0.849136 | 0.891 | 0.627 | 6.77E-33 | 11 | *AP1S2* | Retinal ganglion cells |
| DNER.1 | 7.93E-61 | 0.846834 | 0.716 | 0.251 | 1.75E-56 | 11 | *DNER* | Retinal ganglion cells |
| PRPH | 1.94E-74 | 0.841074 | 0.27 | 0.028 | 4.28E-70 | 11 | *PRPH* | Retinal ganglion cells |
| CELF5 | 1.02E-89 | 0.828191 | 0.673 | 0.171 | 2.26E-85 | 11 | *CELF5* | Retinal ganglion cells |
| PLPPR3 | 5.91E-85 | 0.825388 | 0.825 | 0.278 | 1.30E-80 | 11 | *PLPPR3* | Retinal ganglion cells |
| UCHL1 | 1.77E-53 | 0.825365 | 0.943 | 0.639 | 3.92E-49 | 11 | *UCHL1* | Retinal ganglion cells |
| CRABP1.7 | 5.61E-23 | 0.821808 | 0.934 | 0.732 | 1.24E-18 | 11 | *CRABP1* | Retinal ganglion cells |
| GRIA2 | 2.98E-157 | 0.815429 | 0.559 | 0.061 | 6.57E-153 | 11 | *GRIA2* | Retinal ganglion cells |
| SIX3 | 1.42E-32 | 0.810691 | 0.825 | 0.569 | 3.14E-28 | 11 | *SIX3* | Retinal ganglion cells |
| RAB3A | 2.68E-72 | 0.803325 | 0.697 | 0.22 | 5.91E-68 | 11 | *RAB3A* | Retinal ganglion cells |
| TTC9B | 1.01E-258 | 0.795043 | 0.569 | 0.032 | 2.22E-254 | 11 | *TTC9B* | Retinal ganglion cells |
| MARCKS | 2.62E-66 | 0.794798 | 0.995 | 0.839 | 5.79E-62 | 11 | *MARCKS* | Retinal ganglion cells |
| HOXB4 | 1.71E-66 | 0.794052 | 0.417 | 0.08 | 3.77E-62 | 11 | *HOXB4* | Retinal ganglion cells |
| INA | 2.97E-85 | 0.794046 | 0.81 | 0.25 | 6.56E-81 | 11 | *INA* | Retinal ganglion cells |
| POU2F2 | 3.41E-48 | 0.788105 | 0.507 | 0.153 | 7.53E-44 | 11 | *POU2F2* | Retinal ganglion cells |
| MAPT | 7.90E-96 | 0.786163 | 0.611 | 0.125 | 1.74E-91 | 11 | *MAPT* | Retinal ganglion cells |
| FEZ1 | 6.11E-65 | 0.785723 | 0.758 | 0.288 | 1.35E-60 | 11 | *FEZ1* | Retinal ganglion cells |
| C4orf48 | 3.05E-55 | 0.780257 | 0.896 | 0.598 | 6.74E-51 | 11 | *C4orf48* | Retinal ganglion cells |
| ONECUT2 | 2.22E-37 | 0.778591 | 0.346 | 0.09 | 4.91E-33 | 11 | *ONECUT2* | Retinal ganglion cells |
| MARCKSL1 | 1.07E-69 | 0.778523 | 0.991 | 0.919 | 2.37E-65 | 11 | *MARCKSL1* | Retinal ganglion cells |
| CAMK2N1 | 3.38E-42 | 0.774937 | 0.787 | 0.436 | 7.47E-38 | 11 | *CAMK2N1* | Retinal ganglion cells |
| KIF3A | 9.87E-62 | 0.766582 | 0.758 | 0.318 | 2.18E-57 | 11 | *KIF3A* | Retinal ganglion cells |
| PKIA | 9.58E-58 | 0.759537 | 0.839 | 0.401 | 2.12E-53 | 11 | *PKIA* | Retinal ganglion cells |
| ZFHX3 | 2.32E-35 | 0.75078 | 0.531 | 0.214 | 5.12E-31 | 11 | *ZFHX3* | Retinal ganglion cells |
| TMSB10 | 4.88E-58 | 0.747906 | 0.995 | 0.969 | 1.08E-53 | 11 | *TMSB10* | Retinal ganglion cells |
| EEF1A2 | 1.78E-51 | 0.746867 | 0.754 | 0.299 | 3.93E-47 | 11 | *EEF1A2* | Retinal ganglion cells |
| VEGFA | 9.12E-36 | 0.746406 | 0.673 | 0.308 | 2.01E-31 | 11 | *VEGFA* | Retinal ganglion cells |
| TCEAL7 | 6.97E-43 | 0.738284 | 0.882 | 0.583 | 1.54E-38 | 11 | *TCEAL7* | Retinal ganglion cells |
| PRDM13 | 2.29E-120 | 0.738221 | 0.246 | 0.012 | 5.07E-116 | 11 | *PRDM13* | Retinal ganglion cells |
| MAFB | 1.65E-36 | 0.729529 | 0.336 | 0.086 | 3.64E-32 | 11 | *MAFB* | Retinal ganglion cells |
| SNCG | 9.99E-73 | 0.729232 | 0.275 | 0.03 | 2.21E-68 | 11 | *SNCG* | Retinal ganglion cells |
| FNBP1L | 1.17E-49 | 0.726262 | 0.82 | 0.413 | 2.58E-45 | 11 | *FNBP1L* | Retinal ganglion cells |
| HOXB7 | 1.95E-53 | 0.724571 | 0.332 | 0.06 | 4.32E-49 | 11 | *HOXB7* | Retinal ganglion cells |
| NNAT | 6.66E-36 | 0.723848 | 0.9 | 0.596 | 1.47E-31 | 11 | *NNAT* | Retinal ganglion cells |
| BEX4 | 5.33E-46 | 0.715652 | 0.924 | 0.666 | 1.18E-41 | 11 | *BEX4* | Retinal ganglion cells |
| PPP1R14B | 6.55E-50 | 0.708359 | 0.787 | 0.369 | 1.45E-45 | 11 | *PPP1R14B* | Retinal ganglion cells |
| CXXC5 | 3.55E-38 | 0.707446 | 0.891 | 0.66 | 7.84E-34 | 11 | *CXXC5* | Retinal ganglion cells |
| ANK3 | 7.40E-49 | 0.701732 | 0.692 | 0.272 | 1.63E-44 | 11 | *ANK3* | Retinal ganglion cells |
| JPH4 | 2.47E-160 | 0.700557 | 0.578 | 0.064 | 5.45E-156 | 11 | *JPH4* | Retinal ganglion cells |
| TAGLN2 | 1.04E-22 | -0.70265 | 0.265 | 0.59 | 2.30E-18 | 11 | *TAGLN2* | Retinal ganglion cells |
| MKI67.1 | 5.90E-10 | -0.70688 | 0.028 | 0.198 | 1.30E-05 | 11 | *MKI67* | Retinal ganglion cells |
| SSX2IP | 1.65E-25 | -0.71151 | 0.185 | 0.541 | 3.64E-21 | 11 | *SSX2IP* | Retinal ganglion cells |
| RPL23 | 9.04E-32 | -0.71568 | 0.919 | 0.906 | 2.00E-27 | 11 | *RPL23* | Retinal ganglion cells |
| TMEM123 | 6.06E-31 | -0.71704 | 0.09 | 0.507 | 1.34E-26 | 11 | *TMEM123* | Retinal ganglion cells |
| CD9.2 | 4.07E-15 | -0.72102 | 0.062 | 0.311 | 8.98E-11 | 11 | *CD9* | Retinal ganglion cells |
| VXN.6 | 1.97E-10 | -0.72234 | 0.076 | 0.262 | 4.35E-06 | 11 | *VXN* | Retinal ganglion cells |
| PLEKHB1.4 | 1.77E-16 | -0.72506 | 0.076 | 0.347 | 3.90E-12 | 11 | *PLEKHB1* | Retinal ganglion cells |
| ROM1.3 | 4.54E-10 | -0.72611 | 0.047 | 0.226 | 1.00E-05 | 11 | *ROM1* | Retinal ganglion cells |
| TPH1.7 | 2.91E-09 | -0.73191 | 0.062 | 0.231 | 6.44E-05 | 11 | *TPH1* | Retinal ganglion cells |
| NEUROD1. | 4.66E-08 | -0.73405 | 0.318 | 0.437 | 0.00103 | 11 | *NEUROD1* | Retinal ganglion cells |
| SEPT4.7 | 2.67E-08 | -0.7365 | 0.27 | 0.399 | 0.000589 | 11 | *Sep-04* | Retinal ganglion cells |
| PCNA.2 | 1.09E-17 | -0.74705 | 0.36 | 0.58 | 2.41E-13 | 11 | *PCNA* | Retinal ganglion cells |
| SLC16A1 | 5.36E-23 | -0.75088 | 0.19 | 0.521 | 1.18E-18 | 11 | *SLC16A1* | Retinal ganglion cells |
| CRABP2.4 | 3.99E-15 | -0.76602 | 0.341 | 0.574 | 8.82E-11 | 11 | *CRABP2* | Retinal ganglion cells |
| CRYM.3 | 2.46E-14 | -0.76922 | 0.071 | 0.313 | 5.44E-10 | 11 | *CRYM* | Retinal ganglion cells |
| TIMP1 | 1.09E-28 | -0.7721 | 0.118 | 0.507 | 2.41E-24 | 11 | *TIMP1* | Retinal ganglion cells |
| BTG3.1 | 2.88E-25 | -0.77359 | 0.403 | 0.677 | 6.36E-21 | 11 | *BTG3* | Retinal ganglion cells |
| TULP1.8 | 4.18E-16 | -0.78169 | 0.052 | 0.309 | 9.23E-12 | 11 | *TULP1* | Retinal ganglion cells |
| CYP1B1.5 | 5.36E-12 | -0.78241 | 0.19 | 0.404 | 1.18E-07 | 11 | *CYP1B1* | Retinal ganglion cells |

NEAT1.2 TTYH1.7 TUBB4B.4 NPC2.1 OTX2.6 RARRES2 DAPL1.6 PRSS23.5 RPL7 UNC119.8 GPC3.6 UBE2C.7 CRX.8 RPS6 PCLAF.2 PTTG1.4 SPARC.1 S100A6 EGR1.4 DIO3.7 ZFP36L2.4 HES1.5 NUSAP1.6 IFITM2.7 CKB SLC38A5.8 HMGN2.1 HIST1H4C. APOE.3 SOX2.6 GSTP1 IFITM3.8 MDK.4 CLU.7 RPL13A DCT.7 TF.8 CENPF.7 SPP1.8 RRAD.7 B2M.2 TRH.6 TOP2A.7 DEK.1 TYMS.3 RPS20 ZFP36L1.7 AIPL1.8 GNB3.8 GNG5 COL1A2.5 HES6.4 CCND1.5 HMGB2.6 PDE6H.9 NRL.8 PDC.10 RCVRN.7 VIM.7

| 5.96E-17 | -0.78313 | 0.668 | 0.778 | 1.32E-12 | 11 | *NEAT1* | Retinal ganglion cells |
| --- | --- | --- | --- | --- | --- | --- | --- |
| 2.40E-11 | -0.79478 | 0.284 | 0.461 | 5.31E-07 | 11 | *TTYH1* | Retinal ganglion cells |
| 1.76E-18 | -0.80121 | 0.801 | 0.83 | 3.90E-14 | 11 | *TUBB4B* | Retinal ganglion cells |
| 1.47E-25 | -0.80501 | 0.147 | 0.506 | 3.25E-21 | 11 | *NPC2* | Retinal ganglion cells |
| 2.78E-16 | -0.80562 | 0.1 | 0.366 | 6.15E-12 | 11 | *OTX2* | Retinal ganglion cells |
| 4.71E-25 | -0.8103 | 0.104 | 0.467 | 1.04E-20 | 11 | *RARRES2* | Retinal ganglion cells |
| 6.80E-14 | -0.81397 | 0.299 | 0.536 | 1.50E-09 | 11 | *DAPL1* | Retinal ganglion cells |
| 5.54E-14 | -0.82449 | 0.227 | 0.455 | 1.22E-09 | 11 | *PRSS23* | Retinal ganglion cells |
| 3.45E-53 | -0.83333 | 0.953 | 0.944 | 7.61E-49 | 11 | *RPL7* | Retinal ganglion cells |
| 5.13E-05 | -0.84089 | 0.507 | 0.521 | 1 | 11 | *UNC119* | Retinal ganglion cells |
| 8.12E-12 | -0.84507 | 0.137 | 0.354 | 1.79E-07 | 11 | *GPC3* | Retinal ganglion cells |
| 6.41E-07 | -0.85905 | 0.071 | 0.204 | 0.014154 | 11 | *UBE2C* | Retinal ganglion cells |
| 3.96E-16 | -0.86624 | 0.081 | 0.335 | 8.74E-12 | 11 | *CRX* | Retinal ganglion cells |
| 5.94E-73 | -0.88541 | 0.962 | 0.976 | 1.31E-68 | 11 | *RPS6* | Retinal ganglion cells |
| 2.45E-20 | -0.88554 | 0.081 | 0.388 | 5.40E-16 | 11 | *PCLAF* | Retinal ganglion cells |
| 7.64E-15 | -0.89435 | 0.152 | 0.412 | 1.69E-10 | 11 | *PTTG1* | Retinal ganglion cells |
| 3.16E-30 | -0.9032 | 0.104 | 0.507 | 6.98E-26 | 11 | *SPARC* | Retinal ganglion cells |
| 9.98E-16 | -0.90374 | 0.27 | 0.503 | 2.20E-11 | 11 | *S100A6* | Retinal ganglion cells |
| 5.91E-17 | -0.90838 | 0.351 | 0.573 | 1.30E-12 | 11 | *EGR1* | Retinal ganglion cells |
| 4.19E-15 | -0.91578 | 0.071 | 0.318 | 9.25E-11 | 11 | *DIO3* | Retinal ganglion cells |
| 2.52E-25 | -0.93638 | 0.1 | 0.459 | 5.56E-21 | 11 | *ZFP36L2* | Retinal ganglion cells |
| 2.58E-22 | -0.9404 | 0.062 | 0.399 | 5.71E-18 | 11 | *HES1* | Retinal ganglion cells |
| 1.93E-18 | -0.94394 | 0.062 | 0.346 | 4.25E-14 | 11 | *NUSAP1* | Retinal ganglion cells |
| 3.47E-17 | -0.94417 | 0.024 | 0.292 | 7.65E-13 | 11 | *IFITM2* | Retinal ganglion cells |
| 7.72E-43 | -0.9668 | 0.976 | 0.971 | 1.71E-38 | 11 | *CKB* | Retinal ganglion cells |
| 3.73E-17 | -0.96732 | 0.071 | 0.337 | 8.24E-13 | 11 | *SLC38A5* | Retinal ganglion cells |
| 9.19E-44 | -0.98019 | 0.9 | 0.923 | 2.03E-39 | 11 | *HMGN2* | Retinal ganglion cells |
| 0.007754 | -0.98977 | 0.706 | 0.582 | 1 | 11 | *HIST1H4C* | Retinal ganglion cells |
| 1.15E-16 | -0.99147 | 0.204 | 0.461 | 2.53E-12 | 11 | *APOE* | Retinal ganglion cells |
| 4.32E-24 | -0.99215 | 0.076 | 0.423 | 9.54E-20 | 11 | *SOX2* | Retinal ganglion cells |
| 3.06E-66 | -0.99373 | 0.763 | 0.922 | 6.76E-62 | 11 | *GSTP1* | Retinal ganglion cells |
| 1.59E-20 | -1.00257 | 0.052 | 0.366 | 3.51E-16 | 11 | *IFITM3* | Retinal ganglion cells |
| 5.64E-29 | -1.00983 | 0.479 | 0.729 | 1.24E-24 | 11 | *MDK* | Retinal ganglion cells |
| 7.13E-13 | -1.01142 | 0.616 | 0.674 | 1.57E-08 | 11 | *CLU* | Retinal ganglion cells |
| 1.85E-46 | -1.03031 | 0.9 | 0.921 | 4.09E-42 | 11 | *RPL13A* | Retinal ganglion cells |
| 2.89E-14 | -1.03041 | 0.062 | 0.3 | 6.38E-10 | 11 | *DCT* | Retinal ganglion cells |
| 3.63E-08 | -1.03107 | 0.066 | 0.22 | 0.000802 | 11 | *TF* | Retinal ganglion cells |
| 5.24E-09 | -1.03136 | 0.133 | 0.308 | 0.000116 | 11 | *CENPF* | Retinal ganglion cells |
| 2.63E-10 | -1.05121 | 0.36 | 0.499 | 5.81E-06 | 11 | *SPP1* | Retinal ganglion cells |
| 2.72E-13 | -1.05139 | 0.1 | 0.327 | 6.00E-09 | 11 | *RRAD* | Retinal ganglion cells |
| 2.99E-33 | -1.0559 | 0.502 | 0.767 | 6.61E-29 | 11 | *B2M* | Retinal ganglion cells |
| 2.68E-05 | -1.05712 | 0.512 | 0.573 | 0.590937 | 11 | *TRH* | Retinal ganglion cells |
| 1.66E-11 | -1.07352 | 0.085 | 0.286 | 3.67E-07 | 11 | *TOP2A* | Retinal ganglion cells |
| 1.03E-42 | -1.08689 | 0.701 | 0.87 | 2.28E-38 | 11 | *DEK* | Retinal ganglion cells |
| 8.92E-31 | -1.09558 | 0.303 | 0.637 | 1.97E-26 | 11 | *TYMS* | Retinal ganglion cells |
| 2.61E-46 | -1.10815 | 0.55 | 0.841 | 5.76E-42 | 11 | *RPS20* | Retinal ganglion cells |
| 2.43E-27 | -1.14352 | 0.081 | 0.458 | 5.36E-23 | 11 | *ZFP36L1* | Retinal ganglion cells |
| 7.11E-17 | -1.16183 | 0.076 | 0.341 | 1.57E-12 | 11 | *AIPL1* | Retinal ganglion cells |
| 1.58E-14 | -1.18467 | 0.218 | 0.428 | 3.48E-10 | 11 | *GNB3* | Retinal ganglion cells |
| 1.63E-62 | -1.18798 | 0.365 | 0.821 | 3.60E-58 | 11 | *GNG5* | Retinal ganglion cells |
| 1.49E-19 | -1.18969 | 0.085 | 0.384 | 3.28E-15 | 11 | *COL1A2* | Retinal ganglion cells |
| 2.72E-14 | -1.24618 | 0.621 | 0.662 | 6.01E-10 | 11 | *HES6* | Retinal ganglion cells |
| 1.29E-25 | -1.27283 | 0.114 | 0.465 | 2.84E-21 | 11 | *CCND1* | Retinal ganglion cells |
| 2.94E-32 | -1.27821 | 0.479 | 0.741 | 6.49E-28 | 11 | *HMGB2* | Retinal ganglion cells |
| 6.95E-17 | -1.3402 | 0.118 | 0.389 | 1.54E-12 | 11 | *PDE6H* | Retinal ganglion cells |
| 5.11E-08 | -1.39797 | 0.204 | 0.348 | 0.001128 | 11 | *NRL* | Retinal ganglion cells |
| 1.15E-13 | -1.56992 | 0.227 | 0.432 | 2.54E-09 | 11 | *PDC* | Retinal ganglion cells |
| 8.12E-12 | -1.68882 | 0.199 | 0.384 | 1.79E-07 | 11 | *RCVRN* | Retinal ganglion cells |
| 9.36E-33 | -1.78418 | 0.668 | 0.807 | 2.07E-28 | 11 | *VIM* | Retinal ganglion cells |
| 1.62E-21 | -1.89226 | 0.171 | 0.469 | 3.59E-17 | 11 | *CYP26A1* | Retinal ganglion cells |

CYP26A1.8

| SFRP2.9 | 1.20E-27 | -2.00761 | 0.213 | 0.56 | 2.64E-23 | 11 | *SFRP2* | Retinal ganglion cells |
| --- | --- | --- | --- | --- | --- | --- | --- | --- |
| CRYAB | 0.003568 | 1.744607 | 0.143 | 0.085 | 1 | 12 | *CRYAB* | Rod precursors |
| MALAT1 | 1.93E-07 | 0.719557 | 0.994 | 0.999 | 0.004255 | 12 | *MALAT1* | Rod precursors |
| FOS.5 | 1.89E-15 | -0.7369 | 0.246 | 0.569 | 4.17E-11 | 12 | *FOS* | Rod precursors |
| CYP26A1.9 | 0.000155 | -0.76298 | 0.32 | 0.462 | 1 | 12 | *CYP26A1* | Rod precursors |
| CENPF.8 | 3.87E-06 | -0.77868 | 0.149 | 0.306 | 0.085439 | 12 | *CENPF* | Rod precursors |
| TOP2A.8 | 1.81E-08 | -0.80892 | 0.086 | 0.284 | 0.000399 | 12 | *TOP2A* | Rod precursors |
| HIST1H4C. | 9.38E-10 | -1.34097 | 0.343 | 0.595 | 2.07E-05 | 12 | *HIST1H4C* | Rod precursors |
| PTGDS.5 | 2.52E-95 | 1.926853 | 0.754 | 0.174 | 5.57E-91 | 13 | *PTGDS* | RPE |
| PMEL | 6.47E-30 | 1.906959 | 0.485 | 0.171 | 1.43E-25 | 13 | *PMEL* | RPE |
| SERPINF1.1 | 1.16E-07 | 1.445684 | 0.485 | 0.324 | 0.002564 | 13 | *SERPINF1* | RPE |
| HSD17B2.1 | 2.33E-200 | 1.356964 | 0.689 | 0.058 | 5.14E-196 | 13 | *HSD17B2* | RPE |
| CLU.8 | 7.42E-55 | 1.343286 | 0.958 | 0.662 | 1.64E-50 | 13 | *CLU* | RPE |
| GJA1 | 6.94E-208 | 1.338433 | 0.617 | 0.045 | 1.53E-203 | 13 | *GJA1* | RPE |
| TIMP3 | 3.70E-57 | 1.24406 | 0.689 | 0.21 | 8.17E-53 | 13 | *TIMP3* | RPE |
| IGFBP7.1 | 1.03E-06 | 1.214255 | 0.341 | 0.201 | 0.022634 | 13 | *IGFBP7* | RPE |
| ARL4A | 8.63E-74 | 1.206274 | 0.784 | 0.261 | 1.91E-69 | 13 | *ARL4A* | RPE |
| AOC2 | 2.54E-221 | 1.178714 | 0.449 | 0.018 | 5.60E-217 | 13 | *AOC2* | RPE |
| ID3.1 | 1.96E-48 | 1.134194 | 0.868 | 0.452 | 4.33E-44 | 13 | *ID3* | RPE |
| EFNA5 | 1.13E-82 | 1.132491 | 0.701 | 0.174 | 2.50E-78 | 13 | *EFNA5* | RPE |
| GNG11 | 3.06E-56 | 1.12821 | 0.605 | 0.167 | 6.76E-52 | 13 | *GNG11* | RPE |
| TFPI2 | 3.44E-43 | 1.118455 | 0.275 | 0.044 | 7.60E-39 | 13 | *TFPI2* | RPE |
| TRPM3 | 1.93E-86 | 1.100221 | 0.611 | 0.119 | 4.27E-82 | 13 | *TRPM3* | RPE |
| FAM84A | 1.27E-121 | 1.091181 | 0.569 | 0.072 | 2.81E-117 | 13 | *FAM84A* | RPE |
| NPC2.2 | 2.09E-61 | 1.087026 | 0.922 | 0.479 | 4.62E-57 | 13 | *NPC2* | RPE |
| TSC22D1.1 | 9.49E-48 | 1.082014 | 0.892 | 0.632 | 2.10E-43 | 13 | *TSC22D1* | RPE |
| ITM2B | 1.63E-70 | 1.056947 | 0.982 | 0.853 | 3.60E-66 | 13 | *ITM2B* | RPE |
| ID1 | 2.17E-43 | 1.042594 | 0.701 | 0.27 | 4.79E-39 | 13 | *ID1* | RPE |
| APOE.4 | 1.55E-48 | 1.03063 | 0.868 | 0.438 | 3.43E-44 | 13 | *APOE* | RPE |
| SGK1 | 9.43E-116 | 1.01279 | 0.581 | 0.078 | 2.08E-111 | 13 | *SGK1* | RPE |
| SFRP1.1 | 7.23E-46 | 1.009589 | 0.587 | 0.173 | 1.60E-41 | 13 | *SFRP1* | RPE |
| PSAT1.1 | 6.26E-50 | 0.999157 | 0.713 | 0.279 | 1.38E-45 | 13 | *PSAT1* | RPE |
| RARRES3 | 5.16E-58 | 0.988574 | 0.407 | 0.071 | 1.14E-53 | 13 | *RARRES3* | RPE |
| COL9A2 | 3.17E-70 | 0.988168 | 0.671 | 0.183 | 7.01E-66 | 13 | *COL9A2* | RPE |
| IGFBP6 | 3.80E-31 | 0.976384 | 0.353 | 0.089 | 8.39E-27 | 13 | *IGFBP6* | RPE |
| ZIC1 | 4.06E-65 | 0.946036 | 0.707 | 0.212 | 8.97E-61 | 13 | *ZIC1* | RPE |
| CXCL14 | 6.61E-161 | 0.927854 | 0.407 | 0.022 | 1.46E-156 | 13 | *CXCL14* | RPE |
| IFITM3.9 | 1.63E-46 | 0.925954 | 0.844 | 0.338 | 3.59E-42 | 13 | *IFITM3* | RPE |
| TKT | 1.54E-49 | 0.919051 | 0.91 | 0.67 | 3.40E-45 | 13 | *TKT* | RPE |
| ALDH1A1.1 | 2.68E-82 | 0.909709 | 0.695 | 0.148 | 5.91E-78 | 13 | *ALDH1A1* | RPE |
| SLC2A1 | 1.45E-36 | 0.909299 | 0.814 | 0.449 | 3.21E-32 | 13 | *SLC2A1* | RPE |
| PARD3 | 8.73E-53 | 0.898771 | 0.629 | 0.196 | 1.93E-48 | 13 | *PARD3* | RPE |
| COLEC12 | 1.64E-83 | 0.896487 | 0.653 | 0.137 | 3.63E-79 | 13 | *COLEC12* | RPE |
| PCP4 | 8.39E-23 | 0.885255 | 0.311 | 0.091 | 1.85E-18 | 13 | *PCP4* | RPE |
| SESN3 | 1.98E-39 | 0.881346 | 0.76 | 0.356 | 4.36E-35 | 13 | *SESN3* | RPE |
| TYRP1 | 6.74E-151 | 0.881247 | 0.192 | 0.003 | 1.49E-146 | 13 | *TYRP1* | RPE |
| LRRC17.1 | 1.02E-104 | 0.859199 | 0.551 | 0.074 | 2.25E-100 | 13 | *LRRC17* | RPE |
| BST2 | 5.33E-79 | 0.856852 | 0.545 | 0.096 | 1.18E-74 | 13 | *BST2* | RPE |
| PCDH7 | 3.60E-67 | 0.840482 | 0.623 | 0.155 | 7.94E-63 | 13 | *PCDH7* | RPE |
| CTSD | 1.24E-08 | 0.833622 | 0.521 | 0.35 | 0.000273 | 13 | *CTSD* | RPE |
| SFRP2.10 | 4.55E-26 | 0.830612 | 0.814 | 0.538 | 1.00E-21 | 13 | *SFRP2* | RPE |
| MFAP2 | 2.59E-50 | 0.825953 | 0.731 | 0.264 | 5.73E-46 | 13 | *MFAP2* | RPE |
| GCHFR | 2.07E-49 | 0.815422 | 0.743 | 0.307 | 4.57E-45 | 13 | *GCHFR* | RPE |
| COL1A2.6 | 5.24E-59 | 0.790297 | 0.838 | 0.358 | 1.16E-54 | 13 | *COL1A2* | RPE |
| CYP1B1.6 | 5.29E-16 | 0.788137 | 0.641 | 0.388 | 1.17E-11 | 13 | *CYP1B1* | RPE |
| B2M.3 | 5.11E-22 | 0.787005 | 0.928 | 0.751 | 1.13E-17 | 13 | *B2M* | RPE |
| HSPB1 | 1.01E-40 | 0.784281 | 0.808 | 0.389 | 2.24E-36 | 13 | *HSPB1* | RPE |
| KRT18 | 1.77E-58 | 0.782838 | 0.539 | 0.122 | 3.92E-54 | 13 | *KRT18* | RPE |
| PKP4 | 8.67E-44 | 0.78059 | 0.623 | 0.224 | 1.91E-39 | 13 | *PKP4* | RPE |
| TMEM98 | 3.13E-10 | 0.773032 | 0.701 | 0.488 | 6.91E-06 | 13 | *TMEM98* | RPE |

| CPAMD8 | 9.40E-172 | 0.772591 | 0.461 | 0.028 | 2.08E-167 | 13 | *CPAMD8* | RPE |
| --- | --- | --- | --- | --- | --- | --- | --- | --- |
| CDON | 1.07E-48 | 0.766443 | 0.647 | 0.211 | 2.35E-44 | 13 | *CDON* | RPE |
| PAX6.5 | 1.00E-41 | 0.764591 | 0.808 | 0.397 | 2.22E-37 | 13 | *PAX6* | RPE |
| FTH1 | 4.53E-52 | 0.76242 | 1 | 0.985 | 9.99E-48 | 13 | *FTH1* | RPE |
| DAPL1.7 | 3.75E-24 | 0.755469 | 0.772 | 0.519 | 8.29E-20 | 13 | *DAPL1* | RPE |
| NR2F1 | 4.38E-35 | 0.74332 | 0.91 | 0.763 | 9.67E-31 | 13 | *NR2F1* | RPE |
| LMO4 | 7.11E-33 | 0.741924 | 0.707 | 0.327 | 1.57E-28 | 13 | *LMO4* | RPE |
| CALD1 | 1.22E-30 | 0.74092 | 0.814 | 0.5 | 2.69E-26 | 13 | *CALD1* | RPE |
| PCDH9 | 1.30E-42 | 0.737977 | 0.719 | 0.282 | 2.88E-38 | 13 | *PCDH9* | RPE |
| IGFBP5.5 | 2.48E-40 | 0.729737 | 0.856 | 0.419 | 5.47E-36 | 13 | *IGFBP5* | RPE |
| CYSTM1 | 7.64E-39 | 0.713101 | 0.76 | 0.359 | 1.69E-34 | 13 | *CYSTM1* | RPE |
| TPD52L1 | 1.20E-69 | 0.707723 | 0.557 | 0.109 | 2.65E-65 | 13 | *TPD52L1* | RPE |
| TTYH1.8 | 7.25E-09 | -0.70651 | 0.275 | 0.459 | 0.00016 | 13 | *TTYH1* | RPE |
| PCNA.3 | 3.05E-14 | -0.70689 | 0.347 | 0.579 | 6.73E-10 | 13 | *PCNA* | RPE |
| YWHAH | 2.04E-19 | -0.71174 | 0.347 | 0.619 | 4.51E-15 | 13 | *YWHAH* | RPE |
| PTH2.2 | 2.94E-09 | -0.7177 | 0.341 | 0.528 | 6.50E-05 | 13 | *PTH2* | RPE |
| NT5DC2.1 | 6.34E-14 | -0.72015 | 0.204 | 0.466 | 1.40E-09 | 13 | *NT5DC2* | RPE |
| ENO2.6 | 6.69E-12 | -0.7235 | 0.521 | 0.65 | 1.48E-07 | 13 | *ENO2* | RPE |
| CADPS.6 | 2.95E-12 | -0.73736 | 0.102 | 0.346 | 6.51E-08 | 13 | *CADPS* | RPE |
| LINC00599 | 1.20E-14 | -0.74981 | 0.03 | 0.307 | 2.65E-10 | 13 | *LINC00599* | RPE |
| RRAD.8 | 0.003958 | -0.75115 | 0.246 | 0.321 | 1 | 13 | *RRAD* | RPE |
| CRYM.4 | 1.70E-11 | -0.75363 | 0.072 | 0.311 | 3.75E-07 | 13 | *CRYM* | RPE |
| TAGLN3.3 | 2.16E-17 | -0.76087 | 0.072 | 0.396 | 4.76E-13 | 13 | *TAGLN3* | RPE |
| PCLAF.3 | 7.33E-14 | -0.76152 | 0.102 | 0.385 | 1.62E-09 | 13 | *PCLAF* | RPE |
| NCAM1 | 2.08E-23 | -0.76363 | 0.329 | 0.673 | 4.60E-19 | 13 | *NCAM1* | RPE |
| UNC119.9 | 0.000214 | -0.77035 | 0.479 | 0.522 | 1 | 13 | *UNC119* | RPE |
| MAP1B.1 | 6.57E-23 | -0.77066 | 0.737 | 0.858 | 1.45E-18 | 13 | *MAP1B* | RPE |
| VXN.7 | 3.06E-11 | -0.77172 | 0.036 | 0.262 | 6.75E-07 | 13 | *VXN* | RPE |
| RXRG.7 | 4.42E-12 | -0.77285 | 0.102 | 0.342 | 9.76E-08 | 13 | *RXRG* | RPE |
| IGSF21.5 | 4.84E-15 | -0.77481 | 0.018 | 0.297 | 1.07E-10 | 13 | *IGSF21* | RPE |
| DPYSL3.5 | 2.23E-15 | -0.77636 | 0.06 | 0.35 | 4.93E-11 | 13 | *DPYSL3* | RPE |
| HMGA1.4 | 2.44E-12 | -0.78362 | 0.527 | 0.66 | 5.38E-08 | 13 | *HMGA1* | RPE |
| MIAT.2 | 6.72E-11 | -0.78806 | 0.186 | 0.41 | 1.48E-06 | 13 | *MIAT* | RPE |
| UBE2C.8 | 1.45E-05 | -0.80764 | 0.072 | 0.203 | 0.320217 | 13 | *UBE2C* | RPE |
| CRMP1.3 | 2.83E-21 | -0.80954 | 0.066 | 0.439 | 6.26E-17 | 13 | *CRMP1* | RPE |
| TPH1.8 | 2.39E-10 | -0.81314 | 0.024 | 0.231 | 5.27E-06 | 13 | *TPH1* | RPE |
| ATP1A3.6 | 6.96E-16 | -0.83639 | 0.054 | 0.356 | 1.54E-11 | 13 | *ATP1A3* | RPE |
| UBE2T.2 | 1.82E-18 | -0.84181 | 0.162 | 0.49 | 4.03E-14 | 13 | *UBE2T* | RPE |
| UBE2S.4 | 2.89E-14 | -0.84428 | 0.491 | 0.672 | 6.38E-10 | 13 | *UBE2S* | RPE |
| TYMS.4 | 2.99E-13 | -0.85161 | 0.479 | 0.629 | 6.61E-09 | 13 | *TYMS* | RPE |
| NUSAP1.7 | 3.36E-12 | -0.85322 | 0.09 | 0.342 | 7.42E-08 | 13 | *NUSAP1* | RPE |
| PLEKHA1.3 | 3.21E-14 | -0.85499 | 0.395 | 0.63 | 7.08E-10 | 13 | *PLEKHA1* | RPE |
| SEPT4.8 | 6.06E-13 | -0.85535 | 0.15 | 0.402 | 1.34E-08 | 13 | *Sep-04* | RPE |
| BEX1 | 6.76E-27 | -0.86483 | 0.497 | 0.799 | 1.49E-22 | 13 | *BEX1* | RPE |
| SYP.7 | 2.30E-18 | -0.87652 | 0.066 | 0.396 | 5.08E-14 | 13 | *SYP* | RPE |
| GPC3.7 | 2.02E-13 | -0.8803 | 0.084 | 0.354 | 4.46E-09 | 13 | *GPC3* | RPE |
| SOX4.6 | 3.26E-13 | -0.88875 | 0.82 | 0.866 | 7.20E-09 | 13 | *SOX4* | RPE |
| H2AFY.1 | 7.00E-33 | -0.89177 | 0.569 | 0.818 | 1.55E-28 | 13 | *H2AFY* | RPE |
| GPM6B.1 | 4.22E-20 | -0.89357 | 0.377 | 0.664 | 9.32E-16 | 13 | *GPM6B* | RPE |
| MLLT11.2 | 1.54E-22 | -0.90075 | 0.455 | 0.697 | 3.41E-18 | 13 | *MLLT11* | RPE |
| HMGN2.2 | 5.59E-32 | -0.90265 | 0.844 | 0.925 | 1.23E-27 | 13 | *HMGN2* | RPE |
| GADD45G. | 3.82E-17 | -0.90924 | 0.168 | 0.477 | 8.44E-13 | 13 | *GADD45G* | RPE |
| GADD45A. | 4.69E-09 | -0.91591 | 0.377 | 0.533 | 0.000104 | 13 | *GADD45A* | RPE |
| GNB3.9 | 4.05E-08 | -0.9304 | 0.263 | 0.425 | 0.000894 | 13 | *GNB3* | RPE |
| TUBB.2 | 3.05E-28 | -0.94929 | 0.922 | 0.949 | 6.74E-24 | 13 | *TUBB* | RPE |
| RORB | 3.93E-26 | -0.95579 | 0.192 | 0.6 | 8.67E-22 | 13 | *RORB* | RPE |
| PCBP4.6 | 5.03E-19 | -0.95785 | 0.198 | 0.526 | 1.11E-14 | 13 | *PCBP4* | RPE |
| TF.9 | 0.000763 | -0.96045 | 0.12 | 0.217 | 1 | 13 | *TF* | RPE |
| SLC38A5.9 | 3.79E-14 | -0.9642 | 0.066 | 0.335 | 8.37E-10 | 13 | *SLC38A5* | RPE |
| TOP2A.9 | 2.24E-07 | -0.98955 | 0.114 | 0.283 | 0.004938 | 13 | *TOP2A* | RPE |

MAP2.4 TUBB4B.5 NNAT.1 DEK.2 AIPL1.9 AMER2.6 HMGB2.7 CENPF.9 LAMP5.8 TUBB2B.2 IGFBP2.3 SPP1.9 STMN1.3 RTN4.3 FAM57B.7 NEUROD1. RGS16.1 PDE6H.10 STMN2.7 NRL.9 FABP7.5 HIST1H4C. SCG3.7 TRH.7 PDC.11 RCVRN.8 HES6.5 CKB.1 TFF1.1 TPH1.9 DCT.8 HMGA1.5 RCVRN.9 TPRX1.1 MCM3 DEK.3 FBL.1 SSTR2.1 CHCHD2.4 TUBB.3 TULP1.9 PPP1CC.1 AIPL1.10 RPS4Y1.1 COL18A1.3 MEST.3 PAX6.6 GPM6B.2 CRYM.5 CYP1B1.7 NEAT1.3 HES1.6 GPC3.8 DIO3.8 DAPL1.8 IFITM2.8 IGFBP2.4 PRSS23.6 TTYH1.9 SOX2.7

| 9.54E-22 | -1.01318 | 0.198 | 0.549 | 2.11E-17 | 13 | *MAP2* | RPE |
| --- | --- | --- | --- | --- | --- | --- | --- |
| 2.14E-27 | -1.02549 | 0.653 | 0.834 | 4.73E-23 | 13 | *TUBB4B* | RPE |
| 9.75E-23 | -1.02683 | 0.293 | 0.618 | 2.15E-18 | 13 | *NNAT* | RPE |
| 2.26E-31 | -1.04357 | 0.701 | 0.869 | 4.98E-27 | 13 | *DEK* | RPE |
| 1.22E-11 | -1.056 | 0.102 | 0.338 | 2.68E-07 | 13 | *AIPL1* | RPE |
| 2.32E-21 | -1.05765 | 0.15 | 0.507 | 5.12E-17 | 13 | *AMER2* | RPE |
| 2.42E-17 | -1.07743 | 0.545 | 0.737 | 5.34E-13 | 13 | *HMGB2* | RPE |
| 2.16E-09 | -1.09588 | 0.102 | 0.308 | 4.78E-05 | 13 | *CENPF* | RPE |
| 1.05E-12 | -1.10628 | 0.078 | 0.331 | 2.31E-08 | 13 | *LAMP5* | RPE |
| 3.94E-30 | -1.11928 | 0.653 | 0.839 | 8.69E-26 | 13 | *TUBB2B* | RPE |
| 2.87E-11 | -1.13246 | 0.299 | 0.532 | 6.34E-07 | 13 | *IGFBP2* | RPE |
| 4.91E-09 | -1.15079 | 0.341 | 0.499 | 0.000108 | 13 | *SPP1* | RPE |
| 9.36E-49 | -1.15239 | 0.892 | 0.966 | 2.07E-44 | 13 | *STMN1* | RPE |
| 8.53E-44 | -1.1633 | 0.82 | 0.917 | 1.88E-39 | 13 | *RTN4* | RPE |
| 1.18E-20 | -1.16989 | 0.126 | 0.471 | 2.60E-16 | 13 | *FAM57B* | RPE |
| 9.16E-23 | -1.25939 | 0.06 | 0.445 | 2.02E-18 | 13 | *NEUROD1* | RPE |
| 1.38E-24 | -1.27338 | 0.287 | 0.612 | 3.04E-20 | 13 | *RGS16* | RPE |
| 1.09E-10 | -1.28445 | 0.168 | 0.385 | 2.40E-06 | 13 | *PDE6H* | RPE |
| 5.02E-17 | -1.3147 | 0.084 | 0.4 | 1.11E-12 | 13 | *STMN2* | RPE |
| 3.58E-12 | -1.37657 | 0.096 | 0.35 | 7.90E-08 | 13 | *NRL* | RPE |
| 5.85E-19 | -1.38105 | 0.359 | 0.621 | 1.29E-14 | 13 | *FABP7* | RPE |
| 3.40E-08 | -1.40227 | 0.461 | 0.591 | 0.000752 | 13 | *HIST1H4C* | RPE |
| 4.12E-27 | -1.51912 | 0.126 | 0.54 | 9.10E-23 | 13 | *SCG3* | RPE |
| 9.51E-18 | -1.58203 | 0.281 | 0.58 | 2.10E-13 | 13 | *TRH* | RPE |
| 1.50E-14 | -1.62208 | 0.168 | 0.432 | 3.31E-10 | 13 | *PDC* | RPE |
| 1.23E-16 | -1.64766 | 0.072 | 0.387 | 2.72E-12 | 13 | *RCVRN* | RPE |
| 6.97E-34 | -1.77491 | 0.263 | 0.673 | 1.54E-29 | 13 | *HES6* | RPE |
| 2.23E-84 | -2.06998 | 0.79 | 0.976 | 4.93E-80 | 13 | *CKB* | RPE |
| 1.09E-96 | 1.782926 | 0.464 | 0.055 | 2.41E-92 | 14 | *TFF1* | Cone precursors |
| 1.25E-32 | 1.075021 | 0.56 | 0.214 | 2.75E-28 | 14 | *TPH1* | Cone precursors |
| 1.67E-32 | 1.0717 | 0.651 | 0.279 | 3.69E-28 | 14 | *DCT* | Cone precursors |
| 1.16E-26 | 1.015909 | 0.807 | 0.651 | 2.56E-22 | 14 | *HMGA1* | Cone precursors |
| 1.02E-28 | 0.895145 | 0.723 | 0.366 | 2.26E-24 | 14 | *RCVRN* | Cone precursors |
| 4.23E-37 | 0.865706 | 0.187 | 0.023 | 9.35E-33 | 14 | *TPRX1* | Cone precursors |
| 4.60E-06 | 0.855432 | 0.524 | 0.469 | 0.101646 | 14 | *MCM3* | Cone precursors |
| 1.52E-15 | 0.843567 | 0.801 | 0.866 | 3.35E-11 | 14 | *DEK* | Cone precursors |
| 7.05E-05 | 0.822734 | 0.512 | 0.496 | 1 | 14 | *FBL* | Cone precursors |
| 6.53E-08 | 0.786674 | 0.355 | 0.215 | 0.001443 | 14 | *SSTR2* | Cone precursors |
| 5.26E-17 | 0.786349 | 0.663 | 0.429 | 1.16E-12 | 14 | *CHCHD2* | Cone precursors |
| 7.03E-21 | 0.761438 | 0.922 | 0.949 | 1.55E-16 | 14 | *TUBB* | Cone precursors |
| 1.83E-14 | 0.7498 | 0.518 | 0.292 | 4.04E-10 | 14 | *TULP1* | Cone precursors |
| 7.05E-08 | 0.744238 | 0.602 | 0.553 | 0.001557 | 14 | *PPP1CC* | Cone precursors |
| 8.96E-22 | 0.739288 | 0.639 | 0.321 | 1.98E-17 | 14 | *AIPL1* | Cone precursors |
| 9.48E-28 | 0.708142 | 0.169 | 0.025 | 2.09E-23 | 14 | *RPS4Y1* | Cone precursors |
| 1.50E-13 | -0.70988 | 0.066 | 0.339 | 3.32E-09 | 14 | *COL18A1* | Cone precursors |
| 1.01E-19 | -0.73468 | 0.145 | 0.527 | 2.24E-15 | 14 | *MEST* | Cone precursors |
| 4.69E-15 | -0.74467 | 0.127 | 0.418 | 1.04E-10 | 14 | *PAX6* | Cone precursors |
| 2.68E-21 | -0.74523 | 0.259 | 0.668 | 5.91E-17 | 14 | *GPM6B* | Cone precursors |
| 1.90E-11 | -0.74929 | 0.072 | 0.311 | 4.20E-07 | 14 | *CRYM* | Cone precursors |
| 6.96E-14 | -0.75124 | 0.108 | 0.404 | 1.54E-09 | 14 | *CYP1B1* | Cone precursors |
| 1.13E-18 | -0.75393 | 0.355 | 0.787 | 2.49E-14 | 14 | *NEAT1* | Cone precursors |
| 3.91E-15 | -0.80162 | 0.09 | 0.395 | 8.64E-11 | 14 | *HES1* | Cone precursors |
| 2.74E-11 | -0.82045 | 0.108 | 0.353 | 6.05E-07 | 14 | *GPC3* | Cone precursors |
| 1.80E-11 | -0.84714 | 0.078 | 0.316 | 3.96E-07 | 14 | *DIO3* | Cone precursors |
| 3.82E-16 | -0.84994 | 0.205 | 0.537 | 8.43E-12 | 14 | *DAPL1* | Cone precursors |
| 2.30E-10 | -0.8606 | 0.072 | 0.288 | 5.08E-06 | 14 | *IFITM2* | Cone precursors |
| 7.29E-10 | -0.90566 | 0.289 | 0.533 | 1.61E-05 | 14 | *IGFBP2* | Cone precursors |
| 7.02E-20 | -0.91973 | 0.09 | 0.457 | 1.55E-15 | 14 | *PRSS23* | Cone precursors |
| 4.24E-20 | -0.92269 | 0.096 | 0.465 | 9.36E-16 | 14 | *TTYH1* | Cone precursors |
| 3.36E-18 | -0.93495 | 0.084 | 0.419 | 7.43E-14 | 14 | *SOX2* | Cone precursors |

| IFITM3.10 | 2.50E-14 | -0.93737 | 0.078 | 0.363 | 5.51E-10 | 14 | *IFITM3* | Cone precursors |
| --- | --- | --- | --- | --- | --- | --- | --- | --- |
| ZFP36L1.8 | 3.23E-19 | -0.9854 | 0.096 | 0.454 | 7.13E-15 | 14 | *ZFP36L1* | Cone precursors |
| IGFBP5.6 | 6.11E-12 | -0.99048 | 0.169 | 0.441 | 1.35E-07 | 14 | *IGFBP5* | Cone precursors |
| TF.10 | 2.29E-06 | -0.99719 | 0.072 | 0.219 | 0.050649 | 14 | *TF* | Cone precursors |
| CCND1.6 | 5.65E-19 | -1.04252 | 0.102 | 0.463 | 1.25E-14 | 14 | *CCND1* | Cone precursors |
| COL1A2.7 | 4.82E-13 | -1.05639 | 0.114 | 0.381 | 1.07E-08 | 14 | *COL1A2* | Cone precursors |
| FABP7.6 | 3.94E-20 | -1.05979 | 0.235 | 0.625 | 8.71E-16 | 14 | *FABP7* | Cone precursors |
| LAMP5.9 | 5.31E-12 | -1.06679 | 0.084 | 0.331 | 1.17E-07 | 14 | *LAMP5* | Cone precursors |
| DKK3.7 | 4.79E-23 | -1.07413 | 0.102 | 0.499 | 1.06E-18 | 14 | *DKK3* | Cone precursors |
| PTN.5 | 1.40E-23 | -1.188 | 0.151 | 0.55 | 3.08E-19 | 14 | *PTN* | Cone precursors |
| TRH.8 | 1.91E-19 | -1.26368 | 0.199 | 0.582 | 4.22E-15 | 14 | *TRH* | Cone precursors |
| CYP26A1.1 | 2.79E-15 | -1.32595 | 0.151 | 0.467 | 6.16E-11 | 14 | *CYP26A1* | Cone precursors |
| SPP1.10 | 1.33E-22 | -1.34317 | 0.108 | 0.506 | 2.94E-18 | 14 | *SPP1* | Cone precursors |
| CLU.9 | 1.10E-34 | -1.47862 | 0.187 | 0.687 | 2.43E-30 | 14 | *CLU* | Cone precursors |
| SFRP2.11 | 1.10E-20 | -1.75491 | 0.193 | 0.558 | 2.42E-16 | 14 | *SFRP2* | Cone precursors |
| VIM.8 | 5.20E-38 | -1.82128 | 0.355 | 0.816 | 1.15E-33 | 14 | *VIM* | Cone precursors |
| CRABP1.8 | 1.62E-38 | -1.82461 | 0.253 | 0.755 | 3.59E-34 | 14 | *CRABP1* | Cone precursors |
| TOP2A.10 | 1.78E-143 | 2.318588 | 0.976 | 0.256 | 3.93E-139 | 15 | *TOP2A* | Mitotic cells |
| CENPF.10 | 8.33E-131 | 2.292555 | 0.97 | 0.28 | 1.84E-126 | 15 | *CENPF* | Mitotic cells |
| MKI67.2 | 2.75E-187 | 2.127581 | 0.963 | 0.167 | 6.08E-183 | 15 | *MKI67* | Mitotic cells |
| UBE2C.9 | 1.23E-154 | 2.051163 | 0.921 | 0.176 | 2.72E-150 | 15 | *UBE2C* | Mitotic cells |
| PTTG1.5 | 4.11E-115 | 2.034319 | 0.994 | 0.383 | 9.08E-111 | 15 | *PTTG1* | Mitotic cells |
| CCNB1.1 | 1.33E-184 | 2.033506 | 0.872 | 0.126 | 2.94E-180 | 15 | *CCNB1* | Mitotic cells |
| ASPM.1 | 2.36E-277 | 2.001263 | 0.927 | 0.086 | 5.22E-273 | 15 | *ASPM* | Mitotic cells |
| CCNB2.1 | 2.06E-226 | 1.887485 | 0.902 | 0.103 | 4.54E-222 | 15 | *CCNB2* | Mitotic cells |
| TPX2.1 | 1.62E-171 | 1.832642 | 0.939 | 0.172 | 3.58E-167 | 15 | *TPX2* | Mitotic cells |
| CKS2.1 | 1.54E-103 | 1.827379 | 0.988 | 0.465 | 3.39E-99 | 15 | *CKS2* | Mitotic cells |
| NUSAP1.8 | 8.83E-116 | 1.785457 | 0.97 | 0.315 | 1.95E-111 | 15 | *NUSAP1* | Mitotic cells |
| HMGB2.8 | 1.29E-90 | 1.723584 | 0.994 | 0.722 | 2.84E-86 | 15 | *HMGB2* | Mitotic cells |
| CDC20.1 | 0 | 1.716399 | 0.835 | 0.051 | 0 | 15 | *CDC20* | Mitotic cells |
| CDK1.1 | 3.85E-130 | 1.678256 | 0.89 | 0.212 | 8.50E-126 | 15 | *CDK1* | Mitotic cells |
| CDKN3.1 | 3.74E-169 | 1.649038 | 0.939 | 0.169 | 8.25E-165 | 15 | *CDKN3* | Mitotic cells |
| BIRC5.1 | 3.58E-264 | 1.600137 | 0.957 | 0.093 | 7.91E-260 | 15 | *BIRC5* | Mitotic cells |
| KPNA2.1 | 8.67E-82 | 1.5971 | 0.927 | 0.387 | 1.91E-77 | 15 | *KPNA2* | Mitotic cells |
| CKS1B.1 | 1.85E-147 | 1.559056 | 0.97 | 0.222 | 4.08E-143 | 15 | *CKS1B* | Mitotic cells |
| DLGAP5.1 | 0 | 1.557013 | 0.878 | 0.049 | 0 | 15 | *DLGAP5* | Mitotic cells |
| UBE2S.5 | 5.09E-72 | 1.463709 | 0.982 | 0.656 | 1.12E-67 | 15 | *UBE2S* | Mitotic cells |
| NUF2.1 | 2.77E-287 | 1.461595 | 0.86 | 0.065 | 6.11E-283 | 15 | *NUF2* | Mitotic cells |
| CENPE | 2.95E-260 | 1.454592 | 0.805 | 0.063 | 6.52E-256 | 15 | *CENPE* | Mitotic cells |
| PBK.1 | 1.76E-291 | 1.432351 | 0.902 | 0.071 | 3.89E-287 | 15 | *PBK* | Mitotic cells |
| SMC4.1 | 1.91E-99 | 1.42257 | 0.939 | 0.328 | 4.22E-95 | 15 | *SMC4* | Mitotic cells |
| NEK2.1 | 1.12E-204 | 1.397085 | 0.793 | 0.08 | 2.47E-200 | 15 | *NEK2* | Mitotic cells |
| MAD2L1.1 | 3.08E-129 | 1.392973 | 0.945 | 0.244 | 6.80E-125 | 15 | *MAD2L1* | Mitotic cells |
| TUBA1C | 2.62E-85 | 1.362249 | 0.817 | 0.255 | 5.79E-81 | 15 | *TUBA1C* | Mitotic cells |
| CCNA1 | 5.32E-223 | 1.35339 | 0.677 | 0.05 | 1.17E-218 | 15 | *CCNA1* | Mitotic cells |
| ARL6IP1.2 | 8.73E-65 | 1.350268 | 0.97 | 0.576 | 1.93E-60 | 15 | *ARL6IP1* | Mitotic cells |
| SGO2.1 | 1.87E-219 | 1.328185 | 0.811 | 0.08 | 4.12E-215 | 15 | *SGO2* | Mitotic cells |
| CCNA2.1 | 5.69E-271 | 1.320251 | 0.835 | 0.064 | 1.26E-266 | 15 | *CCNA2* | Mitotic cells |
| KIF20B.1 | 4.53E-159 | 1.311411 | 0.829 | 0.126 | 1.00E-154 | 15 | *KIF20B* | Mitotic cells |
| CENPA.1 | 5.84E-260 | 1.293725 | 0.75 | 0.051 | 1.29E-255 | 15 | *CENPA* | Mitotic cells |
| GTSE1.1 | 2.57E-163 | 1.28813 | 0.823 | 0.119 | 5.67E-159 | 15 | *GTSE1* | Mitotic cells |
| HMMR | 4.84E-250 | 1.258228 | 0.738 | 0.053 | 1.07E-245 | 15 | *HMMR* | Mitotic cells |
| CDCA3.1 | 1.37E-250 | 1.251878 | 0.817 | 0.068 | 3.03E-246 | 15 | *CDCA3* | Mitotic cells |
| CKAP2.1 | 1.01E-77 | 1.246747 | 0.89 | 0.351 | 2.22E-73 | 15 | *CKAP2* | Mitotic cells |
| KNSTRN | 4.16E-92 | 1.228486 | 0.677 | 0.144 | 9.18E-88 | 15 | *KNSTRN* | Mitotic cells |
| KNL1.1 | 1.02E-292 | 1.222044 | 0.787 | 0.048 | 2.25E-288 | 15 | *KNL1* | Mitotic cells |
| KIF23.1 | 2.52E-251 | 1.221444 | 0.78 | 0.06 | 5.57E-247 | 15 | *KIF23* | Mitotic cells |
| AURKA | 2.48E-208 | 1.219217 | 0.689 | 0.059 | 5.47E-204 | 15 | *AURKA* | Mitotic cells |
| PIMREG.1 | 0 | 1.217791 | 0.835 | 0.053 | 0 | 15 | *PIMREG* | Mitotic cells |
| HIST1H1D. | 9.40E-71 | 1.20902 | 0.524 | 0.099 | 2.08E-66 | 15 | *HIST1H1D* | Mitotic cells |

| MXD3 | 4.04E-223 | 1.190021 | 0.787 | 0.072 | 8.92E-219 | 15 | *MXD3* | Mitotic cells |
| --- | --- | --- | --- | --- | --- | --- | --- | --- |
| NDC80.1 | 3.28E-226 | 1.186243 | 0.799 | 0.073 | 7.24E-222 | 15 | *NDC80* | Mitotic cells |
| ECT2 | 2.36E-132 | 1.185964 | 0.86 | 0.168 | 5.21E-128 | 15 | *ECT2* | Mitotic cells |
| AURKB.1 | 0 | 1.172658 | 0.799 | 0.045 | 0 | 15 | *AURKB* | Mitotic cells |
| UBE2T.3 | 2.63E-71 | 1.171345 | 0.939 | 0.466 | 5.81E-67 | 15 | *UBE2T* | Mitotic cells |
| NMU | 1.02E-119 | 1.169137 | 0.787 | 0.151 | 2.24E-115 | 15 | *NMU* | Mitotic cells |
| PLK1 | 4.22E-264 | 1.168527 | 0.677 | 0.039 | 9.33E-260 | 15 | *PLK1* | Mitotic cells |
| TUBB4B.6 | 5.47E-55 | 1.153202 | 0.982 | 0.824 | 1.21E-50 | 15 | *TUBB4B* | Mitotic cells |
| MIS18BP1. | 9.68E-111 | 1.146101 | 0.884 | 0.22 | 2.14E-106 | 15 | *MIS18BP1* | Mitotic cells |
| CDCA8.1 | 3.93E-261 | 1.142418 | 0.72 | 0.045 | 8.68E-257 | 15 | *CDCA8* | Mitotic cells |
| H2AFX.1 | 4.78E-84 | 1.140807 | 0.86 | 0.291 | 1.06E-79 | 15 | *H2AFX* | Mitotic cells |
| RAD21 | 1.55E-65 | 1.086087 | 0.957 | 0.601 | 3.42E-61 | 15 | *RAD21* | Mitotic cells |
| HIST1H4C. | 8.50E-31 | 1.047091 | 0.854 | 0.578 | 1.88E-26 | 15 | *HIST1H4C* | Mitotic cells |
| BUB1 | 0 | 1.037188 | 0.744 | 0.032 | 0 | 15 | *BUB1* | Mitotic cells |
| KIF2C | 0 | 1.030616 | 0.732 | 0.035 | 0 | 15 | *KIF2C* | Mitotic cells |
| CKAP2L.1 | 3.49E-205 | 1.028249 | 0.75 | 0.07 | 7.71E-201 | 15 | *CKAP2L* | Mitotic cells |
| TROAP | 0 | 1.017288 | 0.738 | 0.038 | 0 | 15 | *TROAP* | Mitotic cells |
| KIF11 | 8.02E-185 | 1.012347 | 0.793 | 0.092 | 1.77E-180 | 15 | *KIF11* | Mitotic cells |
| SGO1.1 | 1.19E-182 | 1.005708 | 0.726 | 0.075 | 2.62E-178 | 15 | *SGO1* | Mitotic cells |
| HJURP | 3.85E-261 | 0.998506 | 0.707 | 0.044 | 8.50E-257 | 15 | *HJURP* | Mitotic cells |
| GAS2L3 | 2.37E-179 | 0.98363 | 0.701 | 0.07 | 5.24E-175 | 15 | *GAS2L3* | Mitotic cells |
| HMGB3.1 | 1.62E-65 | 0.955027 | 0.921 | 0.419 | 3.59E-61 | 15 | *HMGB3* | Mitotic cells |
| CCDC34 | 2.06E-67 | 0.943827 | 0.933 | 0.413 | 4.54E-63 | 15 | *CCDC34* | Mitotic cells |
| TACC3 | 1.72E-144 | 0.920753 | 0.744 | 0.104 | 3.79E-140 | 15 | *TACC3* | Mitotic cells |
| LSM5 | 6.91E-50 | 0.918054 | 0.915 | 0.504 | 1.53E-45 | 15 | *LSM5* | Mitotic cells |
| DEPDC1 | 1.97E-280 | 0.917755 | 0.634 | 0.03 | 4.36E-276 | 15 | *DEPDC1* | Mitotic cells |
| NUCKS1.1 | 6.36E-58 | 0.913356 | 0.988 | 0.845 | 1.40E-53 | 15 | *NUCKS1* | Mitotic cells |
| CKAP5 | 1.20E-70 | 0.909222 | 0.823 | 0.28 | 2.65E-66 | 15 | *CKAP5* | Mitotic cells |
| CALM3 | 9.81E-64 | 0.906354 | 0.988 | 0.647 | 2.17E-59 | 15 | *CALM3* | Mitotic cells |
| KIF20A | 0 | 0.904575 | 0.604 | 0.014 | 0 | 15 | *KIF20A* | Mitotic cells |
| SPC25 | 1.55E-127 | 0.902828 | 0.646 | 0.086 | 3.43E-123 | 15 | *SPC25* | Mitotic cells |
| NCAPG | 4.80E-146 | 0.900488 | 0.732 | 0.098 | 1.06E-141 | 15 | *NCAPG* | Mitotic cells |
| JPT1.1 | 8.61E-45 | 0.89895 | 0.957 | 0.663 | 1.90E-40 | 15 | *JPT1* | Mitotic cells |
| PRC1.1 | 5.00E-133 | 0.896716 | 0.774 | 0.124 | 1.10E-128 | 15 | *PRC1* | Mitotic cells |
| KIF14 | 0 | 0.896442 | 0.652 | 0.027 | 0 | 15 | *KIF14* | Mitotic cells |
| TTK | 4.72E-281 | 0.895882 | 0.677 | 0.036 | 1.04E-276 | 15 | *TTK* | Mitotic cells |
| MZT1.1 | 1.30E-48 | 0.888194 | 0.86 | 0.422 | 2.87E-44 | 15 | *MZT1* | Mitotic cells |
| DBF4 | 7.28E-68 | 0.887471 | 0.732 | 0.215 | 1.61E-63 | 15 | *DBF4* | Mitotic cells |
| KIF15 | 8.39E-251 | 0.884357 | 0.713 | 0.047 | 1.85E-246 | 15 | *KIF15* | Mitotic cells |
| TAGLN2.1 | 3.34E-38 | 0.881491 | 0.896 | 0.568 | 7.37E-34 | 15 | *TAGLN2* | Mitotic cells |
| RTKN2 | 1.93E-124 | 0.877076 | 0.652 | 0.09 | 4.27E-120 | 15 | *RTKN2* | Mitotic cells |
| TUBA1B.1 | 2.24E-60 | 0.871608 | 0.994 | 0.957 | 4.95E-56 | 15 | *TUBA1B* | Mitotic cells |
| CENPW.1 | 1.92E-140 | 0.870216 | 0.713 | 0.095 | 4.23E-136 | 15 | *CENPW* | Mitotic cells |
| CEP55 | 5.16E-225 | 0.869963 | 0.652 | 0.044 | 1.14E-220 | 15 | *CEP55* | Mitotic cells |
| H2AFZ.1 | 1.72E-68 | 0.860411 | 0.994 | 0.935 | 3.81E-64 | 15 | *H2AFZ* | Mitotic cells |
| KIF4A | 8.06E-157 | 0.85848 | 0.659 | 0.072 | 1.78E-152 | 15 | *KIF4A* | Mitotic cells |
| CDCA2 | 4.63E-227 | 0.854503 | 0.628 | 0.04 | 1.02E-222 | 15 | *CDCA2* | Mitotic cells |
| KIFC1.1 | 3.25E-122 | 0.852607 | 0.762 | 0.125 | 7.17E-118 | 15 | *KIFC1* | Mitotic cells |
| CEP70 | 7.31E-59 | 0.8491 | 0.732 | 0.243 | 1.61E-54 | 15 | *CEP70* | Mitotic cells |
| BUB1B | 6.55E-292 | 0.842807 | 0.677 | 0.033 | 1.45E-287 | 15 | *BUB1B* | Mitotic cells |
| CDKN2D.1 | 1.12E-55 | 0.828772 | 0.713 | 0.231 | 2.47E-51 | 15 | *CDKN2D* | Mitotic cells |
| HMGN2.3 | 1.86E-51 | 0.814896 | 1 | 0.92 | 4.10E-47 | 15 | *HMGN2* | Mitotic cells |
| PSRC1 | 1.49E-120 | 0.813768 | 0.695 | 0.108 | 3.29E-116 | 15 | *PSRC1* | Mitotic cells |
| LMNB1 | 5.05E-59 | 0.809563 | 0.854 | 0.328 | 1.12E-54 | 15 | *LMNB1* | Mitotic cells |
| NCAPD2 | 4.83E-131 | 0.807814 | 0.732 | 0.111 | 1.07E-126 | 15 | *NCAPD2* | Mitotic cells |
| HSP90B1 | 1.13E-38 | 0.807534 | 0.927 | 0.722 | 2.48E-34 | 15 | *HSP90B1* | Mitotic cells |
| DTYMK | 6.90E-52 | 0.801229 | 0.86 | 0.409 | 1.52E-47 | 15 | *DTYMK* | Mitotic cells |
| CDC25B | 5.25E-98 | 0.796479 | 0.61 | 0.097 | 1.16E-93 | 15 | *CDC25B* | Mitotic cells |
| AC084033. | 5.29E-47 | 0.789248 | 0.78 | 0.341 | 1.17E-42 | 15 | *AC084033.* | Mitotic cells |
| ASCL1 | 2.88E-45 | 0.789117 | 0.549 | 0.146 | 6.35E-41 | 15 | *ASCL1* | Mitotic cells |

| CALM2 | 6.16E-52 | 0.788467 | 0.994 | 0.944 | 1.36E-47 | 15 | *CALM2* | Mitotic cells |
| --- | --- | --- | --- | --- | --- | --- | --- | --- |
| FBXO5 | 1.16E-54 | 0.78805 | 0.732 | 0.25 | 2.55E-50 | 15 | *FBXO5* | Mitotic cells |
| RAD51AP1 | 1.18E-70 | 0.78414 | 0.701 | 0.176 | 2.60E-66 | 15 | *RAD51AP1* | Mitotic cells |
| TUBB6 | 4.15E-72 | 0.78397 | 0.713 | 0.182 | 9.16E-68 | 15 | *TUBB6* | Mitotic cells |
| BUB3.1 | 3.09E-49 | 0.78086 | 0.896 | 0.499 | 6.82E-45 | 15 | *BUB3* | Mitotic cells |
| HP1BP3 | 4.18E-40 | 0.768546 | 0.902 | 0.603 | 9.23E-36 | 15 | *HP1BP3* | Mitotic cells |
| HIST1H1C. | 4.39E-39 | 0.764765 | 0.512 | 0.15 | 9.69E-35 | 15 | *HIST1H1C* | Mitotic cells |
| HMGB1.1 | 6.83E-62 | 0.761469 | 1 | 0.954 | 1.51E-57 | 15 | *HMGB1* | Mitotic cells |
| PRR11 | 1.30E-136 | 0.75308 | 0.622 | 0.073 | 2.88E-132 | 15 | *PRR11* | Mitotic cells |
| KIF18A | 1.59E-229 | 0.742941 | 0.579 | 0.032 | 3.50E-225 | 15 | *KIF18A* | Mitotic cells |
| ARHGAP11 | 2.49E-185 | 0.741325 | 0.555 | 0.038 | 5.50E-181 | 15 | *ARHGAP11* | Mitotic cells |
| CENPN | 7.97E-167 | 0.726207 | 0.689 | 0.07 | 1.76E-162 | 15 | *CENPN* | Mitotic cells |
| OIP5 | 4.47E-249 | 0.724726 | 0.628 | 0.035 | 9.88E-245 | 15 | *OIP5* | Mitotic cells |
| TMPO | 2.89E-42 | 0.718297 | 0.927 | 0.569 | 6.39E-38 | 15 | *TMPO* | Mitotic cells |
| ANP32E.1 | 2.44E-42 | 0.715543 | 0.896 | 0.558 | 5.39E-38 | 15 | *ANP32E* | Mitotic cells |
| EMC9 | 1.02E-47 | 0.713671 | 0.768 | 0.301 | 2.25E-43 | 15 | *EMC9* | Mitotic cells |
| FAM83D | 3.34E-277 | 0.702537 | 0.543 | 0.02 | 7.37E-273 | 15 | *FAM83D* | Mitotic cells |
| ROM1.4 | 7.91E-07 | -0.70588 | 0.067 | 0.223 | 0.017473 | 15 | *ROM1* | Mitotic cells |
| LINC00599 | 1.72E-11 | -0.71184 | 0.073 | 0.306 | 3.80E-07 | 15 | *LINC00599* | Mitotic cells |
| AMER2.7 | 7.86E-06 | -0.71518 | 0.433 | 0.498 | 0.173461 | 15 | *AMER2* | Mitotic cells |
| TUBA4A.5 | 1.20E-10 | -0.72625 | 0.171 | 0.391 | 2.65E-06 | 15 | *TUBA4A* | Mitotic cells |
| MEIS2.3 | 1.24E-10 | -0.72869 | 0.317 | 0.509 | 2.74E-06 | 15 | *MEIS2* | Mitotic cells |
| ATP1A3.7 | 3.01E-09 | -0.72957 | 0.159 | 0.352 | 6.64E-05 | 15 | *ATP1A3* | Mitotic cells |
| SYP.8 | 2.11E-13 | -0.73308 | 0.134 | 0.394 | 4.66E-09 | 15 | *SYP* | Mitotic cells |
| HMGA1.6 | 7.74E-10 | -0.73803 | 0.579 | 0.658 | 1.71E-05 | 15 | *HMGA1* | Mitotic cells |
| PLEKHB1.5 | 3.43E-12 | -0.75154 | 0.098 | 0.343 | 7.57E-08 | 15 | *PLEKHB1* | Mitotic cells |
| TPH1.10 | 4.65E-07 | -0.7752 | 0.073 | 0.229 | 0.010272 | 15 | *TPH1* | Mitotic cells |
| CRX.9 | 9.27E-11 | -0.78488 | 0.11 | 0.332 | 2.05E-06 | 15 | *CRX* | Mitotic cells |
| TULP1.10 | 4.77E-12 | -0.79136 | 0.067 | 0.306 | 1.05E-07 | 15 | *TULP1* | Mitotic cells |
| MAP1LC3A | 5.32E-14 | -0.7966 | 0.244 | 0.488 | 1.18E-09 | 15 | *MAP1LC3A* | Mitotic cells |
| GUK1.2 | 4.20E-18 | -0.82941 | 0.713 | 0.784 | 9.28E-14 | 15 | *GUK1* | Mitotic cells |
| AKAP9.9 | 7.67E-12 | -0.83788 | 0.537 | 0.661 | 1.69E-07 | 15 | *AKAP9* | Mitotic cells |
| RRAD.9 | 1.25E-06 | -0.84068 | 0.165 | 0.323 | 0.02757 | 15 | *RRAD* | Mitotic cells |
| CPE.6 | 9.74E-14 | -0.89001 | 0.524 | 0.669 | 2.15E-09 | 15 | *CPE* | Mitotic cells |
| ENO2.7 | 7.47E-18 | -0.89497 | 0.439 | 0.652 | 1.65E-13 | 15 | *ENO2* | Mitotic cells |
| DCT.9 | 7.61E-10 | -0.94175 | 0.085 | 0.297 | 1.68E-05 | 15 | *DCT* | Mitotic cells |
| FAM57B.8 | 1.85E-14 | -0.97704 | 0.207 | 0.468 | 4.08E-10 | 15 | *FAM57B* | Mitotic cells |
| PCBP4.7 | 9.44E-17 | -1.01136 | 0.25 | 0.524 | 2.08E-12 | 15 | *PCBP4* | Mitotic cells |
| STMN2.8 | 1.43E-09 | -1.09441 | 0.195 | 0.397 | 3.16E-05 | 15 | *STMN2* | Mitotic cells |
| SCG3.8 | 1.53E-15 | -1.13418 | 0.274 | 0.535 | 3.37E-11 | 15 | *SCG3* | Mitotic cells |
| AIPL1.11 | 1.62E-13 | -1.16319 | 0.073 | 0.339 | 3.58E-09 | 15 | *AIPL1* | Mitotic cells |
| UNC119.10 | 5.93E-16 | -1.18016 | 0.28 | 0.528 | 1.31E-11 | 15 | *UNC119* | Mitotic cells |
| PDE6H.11 | 2.79E-08 | -1.24591 | 0.22 | 0.383 | 0.000617 | 15 | *PDE6H* | Mitotic cells |
| GNB3.10 | 2.57E-16 | -1.30269 | 0.14 | 0.428 | 5.68E-12 | 15 | *GNB3* | Mitotic cells |
| NRL.10 | 5.20E-07 | -1.39015 | 0.195 | 0.347 | 0.011481 | 15 | *NRL* | Mitotic cells |
| PDC.12 | 6.37E-10 | -1.49103 | 0.25 | 0.429 | 1.41E-05 | 15 | *PDC* | Mitotic cells |
| RCVRN.10 | 4.71E-12 | -1.69203 | 0.152 | 0.384 | 1.04E-07 | 15 | *RCVRN* | Mitotic cells |
| IGFBP7.2 | 8.44E-82 | 1.904049 | 0.738 | 0.19 | 1.86E-77 | 16 | *IGFBP7* | Muller Glia cells |
| PPY | 1.98E-101 | 1.872541 | 0.512 | 0.065 | 4.38E-97 | 16 | *PPY* | Muller Glia cells |
| LAMP5.10 | 8.43E-108 | 1.762178 | 0.956 | 0.304 | 1.86E-103 | 16 | *LAMP5* | Muller Glia cells |
| GPC3.9 | 1.37E-89 | 1.717815 | 0.912 | 0.328 | 3.03E-85 | 16 | *GPC3* | Muller Glia cells |
| MAL | 4.37E-235 | 1.608746 | 0.7 | 0.051 | 9.64E-231 | 16 | *MAL* | Muller Glia cells |
| CYP26A1.1 | 2.51E-54 | 1.564418 | 0.881 | 0.444 | 5.55E-50 | 16 | *CYP26A1* | Muller Glia cells |
| BCAM | 2.96E-70 | 1.382143 | 0.8 | 0.261 | 6.54E-66 | 16 | *BCAM* | Muller Glia cells |
| TNFSF10 | 1.70E-124 | 1.308349 | 0.506 | 0.051 | 3.76E-120 | 16 | *TNFSF10* | Muller Glia cells |
| GPX3.1 | 1.54E-82 | 1.235811 | 0.65 | 0.128 | 3.40E-78 | 16 | *GPX3* | Muller Glia cells |
| PAX8.1 | 9.56E-180 | 1.218432 | 0.85 | 0.107 | 2.11E-175 | 16 | *PAX8* | Muller Glia cells |
| CD24.2 | 7.31E-27 | 1.218185 | 0.762 | 0.444 | 1.61E-22 | 16 | *CD24* | Muller Glia cells |
| TPM1 | 2.02E-41 | 1.162506 | 0.919 | 0.494 | 4.47E-37 | 16 | *TPM1* | Muller Glia cells |
| FXYD2 | 1.17E-42 | 1.147759 | 0.212 | 0.026 | 2.57E-38 | 16 | *FXYD2* | Muller Glia cells |

| CCL2 | 3.29E-14 | 1.142993 | 0.238 | 0.074 | 7.27E-10 | 16 | *CCL2* | Muller Glia cells |
| --- | --- | --- | --- | --- | --- | --- | --- | --- |
| CDH6.1 | 9.91E-100 | 1.118799 | 0.762 | 0.155 | 2.19E-95 | 16 | *CDH6* | Muller Glia cells |
| S100A10 | 1.59E-158 | 1.091255 | 0.637 | 0.062 | 3.50E-154 | 16 | *S100A10* | Muller Glia cells |
| EVA1B | 1.70E-98 | 1.064472 | 0.819 | 0.184 | 3.76E-94 | 16 | *EVA1B* | Muller Glia cells |
| CFI | 6.38E-96 | 1.029521 | 0.756 | 0.161 | 1.41E-91 | 16 | *CFI* | Muller Glia cells |
| DHRS3.2 | 1.11E-51 | 1.013597 | 0.887 | 0.474 | 2.46E-47 | 16 | *DHRS3* | Muller Glia cells |
| ALDH1A1.2 | 1.48E-70 | 0.999385 | 0.669 | 0.149 | 3.27E-66 | 16 | *ALDH1A1* | Muller Glia cells |
| PTGDS.6 | 3.19E-24 | 0.996115 | 0.494 | 0.183 | 7.05E-20 | 16 | *PTGDS* | Muller Glia cells |
| CCDC198 | 0 | 0.993642 | 0.556 | 0.016 | 0 | 16 | *CCDC198* | Muller Glia cells |
| GNG11.1 | 1.13E-73 | 0.974256 | 0.7 | 0.165 | 2.50E-69 | 16 | *GNG11* | Muller Glia cells |
| COL18A1.4 | 1.62E-69 | 0.971976 | 0.881 | 0.314 | 3.58E-65 | 16 | *COL18A1* | Muller Glia cells |
| TPD52L1.1 | 2.50E-138 | 0.965617 | 0.75 | 0.104 | 5.51E-134 | 16 | *TPD52L1* | Muller Glia cells |
| CYP1B1.8 | 3.19E-53 | 0.961521 | 0.887 | 0.38 | 7.03E-49 | 16 | *CYP1B1* | Muller Glia cells |
| SAT1.2 | 3.18E-41 | 0.941252 | 0.919 | 0.571 | 7.02E-37 | 16 | *SAT1* | Muller Glia cells |
| FOS.6 | 1.93E-28 | 0.926687 | 0.856 | 0.549 | 4.25E-24 | 16 | *FOS* | Muller Glia cells |
| C9orf3 | 3.93E-84 | 0.908804 | 0.75 | 0.182 | 8.67E-80 | 16 | *C9orf3* | Muller Glia cells |
| CP | 2.58E-70 | 0.882524 | 0.725 | 0.178 | 5.70E-66 | 16 | *CP* | Muller Glia cells |
| SPP1.11 | 1.31E-13 | 0.858346 | 0.781 | 0.485 | 2.89E-09 | 16 | *SPP1* | Muller Glia cells |
| ANXA2 | 2.08E-47 | 0.855647 | 0.781 | 0.31 | 4.59E-43 | 16 | *ANXA2* | Muller Glia cells |
| PRDX6 | 1.60E-39 | 0.85483 | 0.963 | 0.705 | 3.53E-35 | 16 | *PRDX6* | Muller Glia cells |
| MYL12A | 1.25E-40 | 0.849218 | 0.887 | 0.505 | 2.75E-36 | 16 | *MYL12A* | Muller Glia cells |
| JUNB.2 | 1.01E-22 | 0.847297 | 0.838 | 0.572 | 2.24E-18 | 16 | *JUNB* | Muller Glia cells |
| LHX1 | 1.66E-95 | 0.846954 | 0.45 | 0.052 | 3.66E-91 | 16 | *LHX1* | Muller Glia cells |
| CHST2 | 6.44E-92 | 0.845704 | 0.525 | 0.076 | 1.42E-87 | 16 | *CHST2* | Muller Glia cells |
| MMP7 | 1.56E-76 | 0.845035 | 0.169 | 0.007 | 3.45E-72 | 16 | *MMP7* | Muller Glia cells |
| IFITM1 | 4.03E-64 | 0.837806 | 0.625 | 0.139 | 8.89E-60 | 16 | *IFITM1* | Muller Glia cells |
| NDP.1 | 6.20E-45 | 0.828882 | 0.537 | 0.14 | 1.37E-40 | 16 | *NDP* | Muller Glia cells |
| AKAP12.3 | 4.45E-33 | 0.818658 | 0.806 | 0.395 | 9.83E-29 | 16 | *AKAP12* | Muller Glia cells |
| TNFRSF12A | 1.09E-27 | 0.815257 | 0.444 | 0.141 | 2.41E-23 | 16 | *TNFRSF12A* | Muller Glia cells |
| PPP1R1A | 4.85E-73 | 0.799684 | 0.637 | 0.14 | 1.07E-68 | 16 | *PPP1R1A* | Muller Glia cells |
| S100A11 | 1.87E-71 | 0.7987 | 0.537 | 0.095 | 4.12E-67 | 16 | *S100A11* | Muller Glia cells |
| SST | 1.21E-40 | 0.785511 | 0.194 | 0.022 | 2.67E-36 | 16 | *SST* | Muller Glia cells |
| THY1.2 | 2.36E-37 | 0.763924 | 0.719 | 0.285 | 5.21E-33 | 16 | *THY1* | Muller Glia cells |
| JUN | 9.55E-27 | 0.762858 | 0.894 | 0.631 | 2.11E-22 | 16 | *JUN* | Muller Glia cells |
| HOXB2.1 | 9.66E-53 | 0.753116 | 0.756 | 0.239 | 2.13E-48 | 16 | *HOXB2* | Muller Glia cells |
| TSC22D1.2 | 1.24E-18 | 0.748064 | 0.894 | 0.632 | 2.73E-14 | 16 | *TSC22D1* | Muller Glia cells |
| CAV1 | 2.65E-65 | 0.745897 | 0.594 | 0.122 | 5.85E-61 | 16 | *CAV1* | Muller Glia cells |
| CYR61 | 1.99E-50 | 0.740927 | 0.581 | 0.146 | 4.38E-46 | 16 | *CYR61* | Muller Glia cells |
| AMIGO2 | 5.20E-129 | 0.732215 | 0.581 | 0.063 | 1.15E-124 | 16 | *AMIGO2* | Muller Glia cells |
| HES1.7 | 3.53E-32 | 0.727503 | 0.806 | 0.373 | 7.79E-28 | 16 | *HES1* | Muller Glia cells |
| RGS5 | 5.09E-78 | 0.727419 | 0.506 | 0.078 | 1.12E-73 | 16 | *RGS5* | Muller Glia cells |
| IMPA2 | 1.35E-39 | 0.726011 | 0.631 | 0.216 | 2.98E-35 | 16 | *IMPA2* | Muller Glia cells |
| IFI6 | 2.71E-44 | 0.725855 | 0.637 | 0.202 | 5.99E-40 | 16 | *IFI6* | Muller Glia cells |
| S100A13 | 2.34E-49 | 0.724921 | 0.944 | 0.485 | 5.17E-45 | 16 | *S100A13* | Muller Glia cells |
| SPRY1 | 7.46E-83 | 0.721281 | 0.7 | 0.139 | 1.65E-78 | 16 | *SPRY1* | Muller Glia cells |
| PDZK1 | 2.04E-76 | 0.705826 | 0.169 | 0.007 | 4.51E-72 | 16 | *PDZK1* | Muller Glia cells |
| PAX2.1 | 1.69E-83 | 0.705022 | 0.738 | 0.145 | 3.73E-79 | 16 | *PAX2* | Muller Glia cells |
| ID3.2 | 8.84E-17 | 0.701396 | 0.756 | 0.456 | 1.95E-12 | 16 | *ID3* | Muller Glia cells |
| CPLX3.1 | 8.31E-13 | -0.70088 | 0.019 | 0.271 | 1.83E-08 | 16 | *CPLX3* | Muller Glia cells |
| MLLT11.3 | 2.83E-12 | -0.70241 | 0.675 | 0.69 | 6.25E-08 | 16 | *MLLT11* | Muller Glia cells |
| MGP | 2.32E-06 | -0.70839 | 0.206 | 0.093 | 0.051245 | 16 | *MGP* | Muller Glia cells |
| OLFM1.2 | 1.15E-13 | -0.71489 | 0.069 | 0.342 | 2.54E-09 | 16 | *OLFM1* | Muller Glia cells |
| UBE2S.6 | 5.87E-10 | -0.71521 | 0.669 | 0.666 | 1.30E-05 | 16 | *UBE2S* | Muller Glia cells |
| TUBA1B.2 | 1.37E-23 | -0.72453 | 0.963 | 0.958 | 3.03E-19 | 16 | *TUBA1B* | Muller Glia cells |
| TMSB15A. | 1.04E-17 | -0.72729 | 0.369 | 0.648 | 2.30E-13 | 16 | *TMSB15A* | Muller Glia cells |
| DPYSL3.6 | 1.34E-11 | -0.73506 | 0.112 | 0.348 | 2.97E-07 | 16 | *DPYSL3* | Muller Glia cells |
| CENPV | 5.75E-20 | -0.74084 | 0.581 | 0.72 | 1.27E-15 | 16 | *CENPV* | Muller Glia cells |
| PCLAF.4 | 4.92E-11 | -0.74744 | 0.15 | 0.383 | 1.09E-06 | 16 | *PCLAF* | Muller Glia cells |
| RTN4.4 | 8.80E-22 | -0.75011 | 0.95 | 0.913 | 1.94E-17 | 16 | *RTN4* | Muller Glia cells |
| GPM6A | 3.93E-19 | -0.75144 | 0.569 | 0.706 | 8.69E-15 | 16 | *GPM6A* | Muller Glia cells |

TUBA1A.1 IGSF21.6 VXN.8 LINC00599 H2AFZ.2 CRMP1.4 UBE2C.10 NUSAP1.9 MAP2.5 TPH1.11 CADPS.7 UBE2T.4 TULP1.11 DEK.4 TUBB.4 EPB41 SYP.9 RXRG.8 TUBB2B.3 CRX.10 ATP1A3.8 GADD45G. PMAIP1.4 SLC38A5.1 OTX2.7 UNC119.11 TOP2A.11 TYMS.5 AMER2.8 ENO2.8 SEPT4.9 CENPF.11 STMN1.4 HMGA1.7 RRAD.10 RORB.1 MAP1B.2 PCBP4.8 CPE.7 DCT.10 AIPL1.12 BASP1.3 FAM57B.9 HMGB2.9 GNB3.11 STMN2.9 NEUROD1. CKB.2 PDE6H.12 NRL.11 SCG3.9 PDC.13 RCVRN.11 HES6.6 CCND1.7 PRSS23.7 PTPRZ1.2 PLEKHA1.4 SPP1.12 MGARP.2

| 4.28E-20 | -0.75233 | 0.95 | 0.946 | 9.44E-16 | 16 | *TUBA1A* | Muller Glia cells |
| --- | --- | --- | --- | --- | --- | --- | --- |
| 5.87E-12 | -0.75492 | 0.056 | 0.296 | 1.30E-07 | 16 | *IGSF21* | Muller Glia cells |
| 1.43E-09 | -0.75811 | 0.056 | 0.261 | 3.15E-05 | 16 | *VXN* | Muller Glia cells |
| 2.33E-13 | -0.75908 | 0.044 | 0.307 | 5.15E-09 | 16 | *LINC00599* | Muller Glia cells |
| 5.43E-32 | -0.76192 | 0.969 | 0.936 | 1.20E-27 | 16 | *H2AFZ* | Muller Glia cells |
| 1.48E-14 | -0.7629 | 0.175 | 0.435 | 3.26E-10 | 16 | *CRMP1* | Muller Glia cells |
| 7.73E-07 | -0.76769 | 0.05 | 0.203 | 0.017079 | 16 | *UBE2C* | Muller Glia cells |
| 1.39E-09 | -0.76895 | 0.131 | 0.341 | 3.07E-05 | 16 | *NUSAP1* | Muller Glia cells |
| 1.26E-07 | -0.76991 | 0.494 | 0.539 | 0.002788 | 16 | *MAP2* | Muller Glia cells |
| 8.49E-07 | -0.77038 | 0.075 | 0.229 | 0.018744 | 16 | *TPH1* | Muller Glia cells |
| 8.76E-15 | -0.79142 | 0.056 | 0.347 | 1.93E-10 | 16 | *CADPS* | Muller Glia cells |
| 4.13E-13 | -0.7945 | 0.269 | 0.486 | 9.12E-09 | 16 | *UBE2T* | Muller Glia cells |
| 9.71E-12 | -0.79681 | 0.069 | 0.306 | 2.14E-07 | 16 | *TULP1* | Muller Glia cells |
| 3.32E-20 | -0.82038 | 0.9 | 0.863 | 7.34E-16 | 16 | *DEK* | Muller Glia cells |
| 2.18E-20 | -0.82878 | 0.938 | 0.948 | 4.81E-16 | 16 | *TUBB* | Muller Glia cells |
| 4.23E-16 | -0.83083 | 0.262 | 0.525 | 9.34E-12 | 16 | *EPB41* | Muller Glia cells |
| 1.27E-15 | -0.85688 | 0.1 | 0.394 | 2.80E-11 | 16 | *SYP* | Muller Glia cells |
| 1.76E-15 | -0.8749 | 0.05 | 0.344 | 3.89E-11 | 16 | *RXRG* | Muller Glia cells |
| 3.14E-19 | -0.8803 | 0.725 | 0.836 | 6.93E-15 | 16 | *TUBB2B* | Muller Glia cells |
| 1.19E-12 | -0.88626 | 0.081 | 0.332 | 2.62E-08 | 16 | *CRX* | Muller Glia cells |
| 3.05E-16 | -0.90188 | 0.05 | 0.356 | 6.73E-12 | 16 | *ATP1A3* | Muller Glia cells |
| 1.23E-14 | -0.90806 | 0.219 | 0.475 | 2.72E-10 | 16 | *GADD45G* | Muller Glia cells |
| 3.57E-16 | -0.91374 | 0.125 | 0.435 | 7.89E-12 | 16 | *PMAIP1* | Muller Glia cells |
| 1.73E-11 | -0.92805 | 0.1 | 0.334 | 3.81E-07 | 16 | *SLC38A5* | Muller Glia cells |
| 8.99E-17 | -0.93334 | 0.05 | 0.365 | 1.98E-12 | 16 | *OTX2* | Muller Glia cells |
| 8.30E-06 | -0.93712 | 0.494 | 0.521 | 0.183306 | 16 | *UNC119* | Muller Glia cells |
| 6.24E-06 | -0.94631 | 0.138 | 0.282 | 0.137874 | 16 | *TOP2A* | Muller Glia cells |
| 5.15E-19 | -0.94643 | 0.375 | 0.632 | 1.14E-14 | 16 | *TYMS* | Muller Glia cells |
| 2.48E-11 | -0.95093 | 0.331 | 0.501 | 5.47E-07 | 16 | *AMER2* | Muller Glia cells |
| 1.64E-16 | -0.9658 | 0.556 | 0.648 | 3.63E-12 | 16 | *ENO2* | Muller Glia cells |
| 2.76E-14 | -0.98149 | 0.138 | 0.402 | 6.09E-10 | 16 | *Sep-04* | Muller Glia cells |
| 2.15E-06 | -0.98376 | 0.15 | 0.306 | 0.047582 | 16 | *CENPF* | Muller Glia cells |
| 7.35E-38 | -0.98897 | 0.925 | 0.965 | 1.62E-33 | 16 | *STMN1* | Muller Glia cells |
| 2.78E-19 | -1.02835 | 0.469 | 0.661 | 6.14E-15 | 16 | *HMGA1* | Muller Glia cells |
| 2.15E-08 | -1.0558 | 0.144 | 0.324 | 0.000474 | 16 | *RRAD* | Muller Glia cells |
| 3.17E-27 | -1.05868 | 0.188 | 0.599 | 6.99E-23 | 16 | *RORB* | Muller Glia cells |
| 9.45E-39 | -1.09473 | 0.688 | 0.86 | 2.09E-34 | 16 | *MAP1B* | Muller Glia cells |
| 1.08E-15 | -1.1102 | 0.312 | 0.522 | 2.39E-11 | 16 | *PCBP4* | Muller Glia cells |
| 1.71E-17 | -1.12592 | 0.575 | 0.667 | 3.78E-13 | 16 | *CPE* | Muller Glia cells |
| 4.43E-14 | -1.13376 | 0.025 | 0.299 | 9.78E-10 | 16 | *DCT* | Muller Glia cells |
| 3.25E-12 | -1.18528 | 0.094 | 0.338 | 7.18E-08 | 16 | *AIPL1* | Muller Glia cells |
| 8.74E-29 | -1.18922 | 0.4 | 0.733 | 1.93E-24 | 16 | *BASP1* | Muller Glia cells |
| 3.24E-19 | -1.23647 | 0.156 | 0.47 | 7.16E-15 | 16 | *FAM57B* | Muller Glia cells |
| 2.51E-21 | -1.23662 | 0.637 | 0.734 | 5.55E-17 | 16 | *HMGB2* | Muller Glia cells |
| 1.18E-12 | -1.24388 | 0.2 | 0.426 | 2.61E-08 | 16 | *GNB3* | Muller Glia cells |
| 2.79E-14 | -1.26069 | 0.119 | 0.399 | 6.17E-10 | 16 | *STMN2* | Muller Glia cells |
| 7.54E-20 | -1.30729 | 0.106 | 0.443 | 1.66E-15 | 16 | *NEUROD1* | Muller Glia cells |
| 5.16E-52 | -1.32527 | 0.938 | 0.972 | 1.14E-47 | 16 | *CKB* | Muller Glia cells |
| 3.90E-09 | -1.34729 | 0.206 | 0.384 | 8.61E-05 | 16 | *PDE6H* | Muller Glia cells |
| 4.73E-07 | -1.43901 | 0.2 | 0.346 | 0.01044 | 16 | *NRL* | Muller Glia cells |
| 1.07E-23 | -1.5619 | 0.188 | 0.538 | 2.37E-19 | 16 | *SCG3* | Muller Glia cells |
| 2.22E-13 | -1.65523 | 0.188 | 0.431 | 4.91E-09 | 16 | *PDC* | Muller Glia cells |
| 2.09E-12 | -1.8023 | 0.15 | 0.384 | 4.62E-08 | 16 | *RCVRN* | Muller Glia cells |
| 1.47E-30 | -1.82018 | 0.35 | 0.67 | 3.25E-26 | 16 | *HES6* | Muller Glia cells |
| 1.11E-37 | 1.125027 | 0.862 | 0.441 | 2.46E-33 | 17 | *CCND1* | Late RPCs |
| 1.36E-43 | 1.102333 | 0.877 | 0.435 | 3.00E-39 | 17 | *PRSS23* | Late RPCs |
| 7.67E-40 | 0.894999 | 0.754 | 0.303 | 1.69E-35 | 17 | *PTPRZ1* | Late RPCs |
| 5.34E-33 | 0.88878 | 0.891 | 0.616 | 1.18E-28 | 17 | *PLEKHA1* | Late RPCs |
| 1.72E-38 | 0.844631 | 0.957 | 0.482 | 3.79E-34 | 17 | *SPP1* | Late RPCs |
| 7.50E-38 | 0.820036 | 0.71 | 0.272 | 1.66E-33 | 17 | *MGARP* | Late RPCs |

| 4.46E-21 | 0.795895 | 0.71 | 0.395 | 9.84E-17 | 17 *MIAT* | Late RPCs |
| --- | --- | --- | --- | --- | --- | --- |
| 7.87E-19 | 0.754726 | 0.623 | 0.3 | 1.74E-14 | 17 *DIO3* | Late RPCs |
| 2.55E-27 | 0.726612 | 0.826 | 0.433 | 5.63E-23 | 17 *ZFP36L1* | Late RPCs |
| 6.88E-25 | 0.721591 | 0.884 | 0.555 | 1.52E-20 | 17 *EGR1* | Late RPCs |
| 1.60E-22 | 0.715375 | 1 | 0.97 | 3.53E-18 | 17 *CKB* | Late RPCs |
| 1.61E-06 | -0.70723 | 0.159 | 0.339 | 0.035491 | 17 *NUSAP1* | Late RPCs |
| 2.51E-11 | -0.70821 | 0.072 | 0.345 | 5.54E-07 | 17 *CADPS* | Late RPCs |
| 3.08E-11 | -0.70887 | 0.043 | 0.306 | 6.79E-07 | 17 *LINC00599* | Late RPCs |
| 7.32E-13 | -0.7094 | 0.471 | 0.712 | 1.62E-08 | 17 *ATP1B1* | Late RPCs |
| 3.61E-12 | -0.71316 | 0.203 | 0.488 | 7.98E-08 | 17 *MAP1LC3A* | Late RPCs |
| 2.36E-11 | -0.71924 | 0.696 | 0.832 | 5.20E-07 | 17 *TUBB4B* | Late RPCs |
| 4.83E-08 | -0.72275 | 0.246 | 0.459 | 0.001066 | 17 *CD24* | Late RPCs |
| 1.90E-08 | -0.7237 | 0.051 | 0.261 | 0.000419 | 17 *VXN* | Late RPCs |
| 4.60E-16 | -0.72601 | 0.072 | 0.427 | 1.02E-11 | 17 *SEZ6L2* | Late RPCs |
| .000577 | -0.73181 | 0.152 | 0.281 | 1 | 17 *TOP2A* | Late RPCs |
| 1.10E-14 | -0.75152 | 0.167 | 0.498 | 2.44E-10 | 17 *SLC38A1* | Late RPCs |
| 8.21E-08 | -0.75333 | 0.5 | 0.661 | 0.001812 | 17 *AKAP9* | Late RPCs |
| 2.05E-10 | -0.76373 | 0.478 | 0.669 | 4.52E-06 | 17 *CPE* | Late RPCs |
| 3.88E-11 | -0.77136 | 0.08 | 0.342 | 8.56E-07 | 17 *RXRG* | Late RPCs |
| 1.04E-11 | -0.77739 | 0.036 | 0.306 | 2.29E-07 | 17 *TULP1* | Late RPCs |
| 1.85E-13 | -0.77996 | 0.196 | 0.511 | 4.09E-09 | 17 *MEIS2* | Late RPCs |
| 2.21E-13 | -0.78005 | 0.051 | 0.349 | 4.87E-09 | 17 *DPYSL3* | Late RPCs |
| 6.68E-14 | -0.80323 | 0.08 | 0.394 | 1.48E-09 | 17 *SYP* | Late RPCs |
| 5.34E-12 | -0.81621 | 0.435 | 0.672 | 1.18E-07 | 17 *UBE2S* | Late RPCs |
| 1.47E-08 | -0.81933 | 0.029 | 0.229 | 0.000325 | 17 *TPH1* | Late RPCs |
| 8.59E-13 | -0.81936 | 0.065 | 0.363 | 1.90E-08 | 17 *OTX2* | Late RPCs |
| 1.83E-05 | -0.8278 | 0.138 | 0.306 | 0.404369 | 17 *CENPF* | Late RPCs |
| 1.26E-14 | -0.83238 | 0.217 | 0.546 | 2.79E-10 | 17 *MAP2* | Late RPCs |
| 1.06E-09 | -0.84375 | 0.094 | 0.333 | 2.34E-05 | 17 *SLC38A5* | Late RPCs |
| 1.00E-14 | -0.84846 | 0.065 | 0.393 | 2.21E-10 | 17 *TUBA4A* | Late RPCs |
| 1.29E-11 | -0.85486 | 0.065 | 0.332 | 2.85E-07 | 17 *CRX* | Late RPCs |
| 5.68E-11 | -0.85614 | 0.145 | 0.401 | 1.25E-06 | 17 *Sep-04* | Late RPCs |
| 3.18E-06 | -0.85772 | 0.043 | 0.203 | 0.070197 | 17 *UBE2C* | Late RPCs |
| 3.59E-12 | -0.85866 | 0.217 | 0.503 | 7.92E-08 | 17 *AMER2* | Late RPCs |
| 3.25E-16 | -0.92648 | 0.014 | 0.355 | 7.17E-12 | 17 *ATP1A3* | Late RPCs |
| 1.81E-08 | -0.94466 | 0.08 | 0.296 | 0.000399 | 17 *DCT* | Late RPCs |
| 2.75E-07 | -0.96611 | 0.13 | 0.323 | 0.006073 | 17 *RRAD* | Late RPCs |
| 6.79E-20 | -0.97706 | 0.319 | 0.654 | 1.50E-15 | 17 *ENO2* | Late RPCs |
| 4.72E-13 | -1.00252 | 0.232 | 0.528 | 1.04E-08 | 17 *UNC119* | Late RPCs |
| 2.22E-18 | -1.09512 | 0.159 | 0.525 | 4.90E-14 | 17 *PCBP4* | Late RPCs |
| 6.66E-15 | -1.10876 | 0.123 | 0.441 | 1.47E-10 | 17 *NEUROD1* | Late RPCs |
| 1.99E-12 | -1.14679 | 0.145 | 0.427 | 4.38E-08 | 17 *GNB3* | Late RPCs |
| 3.87E-18 | -1.14884 | 0.101 | 0.47 | 8.56E-14 | 17 *FAM57B* | Late RPCs |
| 1.93E-12 | -1.19594 | 0.058 | 0.338 | 4.27E-08 | 17 *AIPL1* | Late RPCs |
| 1.88E-08 | -1.21852 | 0.167 | 0.384 | 0.000416 | 17 *PDE6H* | Late RPCs |
| 1.21E-14 | -1.2902 | 0.072 | 0.399 | 2.67E-10 | 17 *STMN2* | Late RPCs |
| 6.34E-08 | -1.36276 | 0.239 | 0.429 | 0.001401 | 17 *PDC* | Late RPCs |
| 6.09E-07 | -1.39249 | 0.377 | 0.592 | 0.013444 | 17 *HIST1H4C* | Late RPCs |
| 1.00E-09 | -1.43352 | 0.109 | 0.348 | 2.22E-05 | 17 *NRL* | Late RPCs |
| 2.51E-22 | -1.4883 | 0.123 | 0.538 | 5.55E-18 | 17 *SCG3* | Late RPCs |
| 8.61E-13 | -1.71901 | 0.094 | 0.384 | 1.90E-08 | 17 *RCVRN* | Late RPCs |
| 6.93E-44 | 1.05477 | 0.891 | 0.366 | 1.53E-39 | 18 *PDE6H* | Cone precursors |
| 6.37E-53 | 0.955247 | 0.899 | 0.303 | 1.41E-48 | 18 *NRN1* | Cone precursors |
| 2.72E-40 | 0.922612 | 0.822 | 0.306 | 6.01E-36 | 18 *RRAD* | Cone precursors |
| 1.30E-42 | 0.88129 | 0.752 | 0.243 | 2.86E-38 | 18 *VXN* | Cone precursors |
| 2.42E-33 | 0.829668 | 0.961 | 0.65 | 5.35E-29 | 18 *AKAP9* | Cone precursors |
| 3.12E-37 | 0.825322 | 0.915 | 0.449 | 6.89E-33 | 18 *FAM57B* | Cone precursors |
| 3.48E-32 | 0.822642 | 0.86 | 0.421 | 7.68E-28 | 18 *YIF1A* | Cone precursors |
| 5.77E-60 | 0.802243 | 0.829 | 0.221 | 1.27E-55 | 18 *AANAT* | Cone precursors |
| 1.06E-38 | 0.798731 | 0.961 | 0.51 | 2.34E-34 | 18 *UNC119* | Cone precursors |

| MIAT.3 |
| --- |
| DIO3.9 |
| ZFP36L1.9 |
| EGR1.5 |
| CKB.3 |
| NUSAP1.10 |
| CADPS.8 |
| LINC00599 |
| ATP1B1.1 |
| MAP1LC3A |
| TUBB4B.7 |
| CD24.3 |
| VXN.9 |
| SEZ6L2.2 |
| TOP2A.12 0 |
| SLC38A1.2 |
| AKAP9.10 |
| CPE.8 |
| RXRG.9 |
| TULP1.12 |
| MEIS2.4 |
| DPYSL3.7 |
| SYP.10 |
| UBE2S.7 |
| TPH1.12 |
| OTX2.8 |
| CENPF.12 |
| MAP2.6 |
| SLC38A5.1 |
| TUBA4A.6 |
| CRX.11 |
| SEPT4.10 |
| UBE2C.11 |
| AMER2.9 |
| ATP1A3.9 |
| DCT.11 |
| RRAD.11 |
| ENO2.9 |
| UNC119.12 |
| PCBP4.9 |
| NEUROD1. |
| GNB3.12 |
| FAM57B.1 |
| AIPL1.13 |
| PDE6H.13 |
| STMN2.10 |
| PDC.14 |
| HIST1H4C. |
| NRL.12 |
| SCG3.10 |
| RCVRN.12 |
| PDE6H.14 |
| NRN1.1 |
| RRAD.12 |
| VXN.10 |
| AKAP9.11 |
| FAM57B.1 |
| YIF1A |
| AANAT.1 |
| UNC119.13 |

| RCVRN.13 | 2.72E-35 | 0.797253 | 0.86 | 0.365 | 6.00E-31 | 18 *RCVRN* | Cone precursors |
| --- | --- | --- | --- | --- | --- | --- | --- |
| SCG3.11 | 9.42E-36 | 0.79005 | 0.961 | 0.517 | 2.08E-31 | 18 *SCG3* | Cone precursors |
| ARF4 | 1.34E-21 | 0.777432 | 0.891 | 0.601 | 2.97E-17 | 18 *ARF4* | Cone precursors |
| PDC.15 | 1.80E-39 | 0.755886 | 0.961 | 0.411 | 3.98E-35 | 18 *PDC* | Cone precursors |
| AIPL1.14 | 1.67E-43 | 0.752645 | 0.907 | 0.316 | 3.69E-39 | 18 *AIPL1* | Cone precursors |
| TUBA4A.7 | 1.03E-38 | 0.720597 | 0.899 | 0.372 | 2.26E-34 | 18 *TUBA4A* | Cone precursors |
| MIR7-3HG. | 7.93E-42 | 0.711252 | 0.853 | 0.288 | 1.75E-37 | 18 *MIR7-3HG* | Cone precursors |
| KCNQ1OT1 | 6.30E-22 | 0.709262 | 0.938 | 0.578 | 1.39E-17 | 18 *KCNQ1OT1* | Cone precursors |
| SEPT4.11 | 2.66E-35 | 0.708969 | 0.899 | 0.382 | 5.88E-31 | 18 *Sep-04* | Cone precursors |
| TTYH1.10 | 9.34E-05 | -0.73423 | 0.395 | 0.455 | 1 | 18 *TTYH1* | Cone precursors |
| HES1.8 | 8.76E-08 | -0.76601 | 0.194 | 0.391 | 0.001933 | 18 *HES1* | Cone precursors |
| DAPL1.9 | 0.000469 | -0.78201 | 0.527 | 0.527 | 1 | 18 *DAPL1* | Cone precursors |
| EGR1.6 | 2.70E-06 | -0.78354 | 0.527 | 0.565 | 0.059553 | 18 *EGR1* | Cone precursors |
| GPC3.10 | 9.88E-05 | -0.79597 | 0.24 | 0.348 | 1 | 18 *GPC3* | Cone precursors |
| DIO3.10 | 3.75E-06 | -0.79709 | 0.147 | 0.312 | 0.082817 | 18 *DIO3* | Cone precursors |
| MDK.5 | 7.57E-10 | -0.82083 | 0.705 | 0.72 | 1.67E-05 | 18 *MDK* | Cone precursors |
| IFITM2.9 | 0.000165 | -0.8387 | 0.171 | 0.284 | 1 | 18 *IFITM2* | Cone precursors |
| PRSS23.8 | 1.31E-07 | -0.85196 | 0.295 | 0.45 | 0.002884 | 18 *PRSS23* | Cone precursors |
| CCND1.8 | 2.79E-06 | -0.85442 | 0.302 | 0.455 | 0.061665 | 18 *CCND1* | Cone precursors |
| DKK3.8 | 2.83E-07 | -0.85776 | 0.372 | 0.49 | 0.00625 | 18 *DKK3* | Cone precursors |
| SOX2.8 | 8.44E-10 | -0.85948 | 0.194 | 0.414 | 1.86E-05 | 18 *SOX2* | Cone precursors |
| ZFP36L1.10 | 3.58E-09 | -0.86104 | 0.233 | 0.448 | 7.91E-05 | 18 *ZFP36L1* | Cone precursors |
| B2M.4 | 8.97E-13 | -0.87419 | 0.752 | 0.756 | 1.98E-08 | 18 *B2M* | Cone precursors |
| IFITM3.11 | 1.63E-06 | -0.89449 | 0.202 | 0.358 | 0.03604 | 18 *IFITM3* | Cone precursors |
| LAMP5.11 | 0.002953 | -1.01101 | 0.271 | 0.325 | 1 | 18 *LAMP5* | Cone precursors |
| PTN.6 | 2.21E-08 | -1.01366 | 0.419 | 0.541 | 0.000488 | 18 *PTN* | Cone precursors |
| COL1A2.8 | 5.81E-07 | -1.02724 | 0.209 | 0.376 | 0.012832 | 18 *COL1A2* | Cone precursors |
| FOS.7 | 3.03E-08 | -1.1558 | 0.496 | 0.56 | 0.000668 | 18 *FOS* | Cone precursors |
| CLU.10 | 1.32E-11 | -1.21475 | 0.612 | 0.673 | 2.92E-07 | 18 *CLU* | Cone precursors |
| SPP1.13 | 6.26E-10 | -1.28819 | 0.302 | 0.499 | 1.38E-05 | 18 *SPP1* | Cone precursors |
| CRABP1.9 | 2.61E-14 | -1.5044 | 0.705 | 0.741 | 5.76E-10 | 18 *CRABP1* | Cone precursors |
| CYP26A1.1 | 0.000101 | -1.52245 | 0.395 | 0.459 | 1 | 18 *CYP26A1* | Cone precursors |
| VIM.9 | 1.90E-15 | -1.59242 | 0.829 | 0.801 | 4.20E-11 | 18 *VIM* | Cone precursors |
| SFRP2.12 | 3.82E-09 | -1.61983 | 0.395 | 0.55 | 8.44E-05 | 18 *SFRP2* | Cone precursors |
| NRL.13 | 5.62E-51 | 1.465165 | 0.893 | 0.329 | 1.24E-46 | 19 *NRL* | Rod precursors |
| PDC.16 | 4.43E-57 | 1.291903 | 0.984 | 0.411 | 9.79E-53 | 19 *PDC* | Rod precursors |
| TPH1.13 | 7.17E-34 | 1.087098 | 0.656 | 0.214 | 1.58E-29 | 19 *TPH1* | Rod precursors |
| SCG3.12 | 8.51E-45 | 1.045429 | 0.975 | 0.517 | 1.88E-40 | 19 *SCG3* | Rod precursors |
| NEUROG1. | 9.09E-48 | 0.997546 | 0.738 | 0.207 | 2.01E-43 | 19 *NEUROG1* | Rod precursors |
| GNB3.13 | 4.14E-39 | 0.956776 | 0.902 | 0.408 | 9.14E-35 | 19 *GNB3* | Rod precursors |
| RRAD.13 | 4.45E-24 | 0.95643 | 0.721 | 0.309 | 9.83E-20 | 19 *RRAD* | Rod precursors |
| CHODL.1 | 7.31E-26 | 0.942615 | 0.574 | 0.211 | 1.61E-21 | 19 *CHODL* | Rod precursors |
| FAM57B.1 | 2.56E-40 | 0.93181 | 0.934 | 0.449 | 5.66E-36 | 19 *FAM57B* | Rod precursors |
| RBP4.2 | 3.33E-38 | 0.92329 | 0.672 | 0.214 | 7.34E-34 | 19 *RBP4* | Rod precursors |
| TUBA4A.8 | 8.25E-31 | 0.894426 | 0.795 | 0.375 | 1.82E-26 | 19 *TUBA4A* | Rod precursors |
| SEPT4.12 | 8.61E-41 | 0.876317 | 0.885 | 0.383 | 1.90E-36 | 19 *Sep-04* | Rod precursors |
| UNC119.14 | 3.96E-35 | 0.857021 | 0.926 | 0.511 | 8.75E-31 | 19 *UNC119* | Rod precursors |
| NEUROD1. | 5.89E-32 | 0.856686 | 0.869 | 0.423 | 1.30E-27 | 19 *NEUROD1* | Rod precursors |
| CADPS.9 | 2.67E-30 | 0.829925 | 0.762 | 0.329 | 5.89E-26 | 19 *CADPS* | Rod precursors |
| ROM1.5 | 1.79E-31 | 0.817912 | 0.631 | 0.209 | 3.96E-27 | 19 *ROM1* | Rod precursors |
| SLC38A5.1 | 1.79E-32 | 0.816943 | 0.795 | 0.316 | 3.95E-28 | 19 *SLC38A5* | Rod precursors |
| RXRG.10 | 2.66E-34 | 0.810455 | 0.803 | 0.324 | 5.87E-30 | 19 *RXRG* | Rod precursors |
| IGSF21.7 | 2.24E-37 | 0.806038 | 0.77 | 0.278 | 4.95E-33 | 19 *IGSF21* | Rod precursors |
| RCVRN.14 | 1.20E-22 | 0.797849 | 0.779 | 0.368 | 2.66E-18 | 19 *RCVRN* | Rod precursors |
| MIR7-3HG. | 3.66E-25 | 0.754004 | 0.689 | 0.292 | 8.09E-21 | 19 *MIR7-3HG* | Rod precursors |
| PTP4A3.1 | 2.00E-23 | 0.745044 | 0.795 | 0.446 | 4.42E-19 | 19 *PTP4A3* | Rod precursors |
| MAP1LC3A | 2.36E-23 | 0.742372 | 0.844 | 0.472 | 5.21E-19 | 19 *MAP1LC3A* | Rod precursors |
| CRX.12 | 8.67E-34 | 0.74075 | 0.82 | 0.313 | 1.91E-29 | 19 *CRX* | Rod precursors |
| CPLX3.2 | 1.90E-31 | 0.73075 | 0.697 | 0.254 | 4.20E-27 | 19 *CPLX3* | Rod precursors |
| AMER2.10 | 2.81E-26 | 0.713473 | 0.852 | 0.487 | 6.20E-22 | 19 *AMER2* | Rod precursors |

| HPCA | 8.27E-32 | 0.707724 | 0.705 | 0.275 | 1.83E-27 | 19 | *HPCA* | Rod precursors |
| --- | --- | --- | --- | --- | --- | --- | --- | --- |
| NANOS1 | 7.89E-31 | 0.704095 | 0.672 | 0.243 | 1.74E-26 | 19 | *NANOS1* | Rod precursors |
| MDK.6 | 9.19E-10 | -0.72095 | 0.541 | 0.723 | 2.03E-05 | 19 | *MDK* | Rod precursors |
| NKAIN4.1 | 1.81E-13 | -0.72105 | 0.041 | 0.363 | 3.99E-09 | 19 | *NKAIN4* | Rod precursors |
| AGL.1 | 8.81E-07 | -0.72939 | 0.189 | 0.381 | 0.019452 | 19 | *AGL* | Rod precursors |
| NEAT1.4 | 2.20E-07 | -0.73677 | 0.68 | 0.776 | 0.004868 | 19 | *NEAT1* | Rod precursors |
| SOX11.5 | 9.88E-07 | -0.73871 | 0.443 | 0.588 | 0.021815 | 19 | *SOX11* | Rod precursors |
| SAT1.3 | 4.89E-09 | -0.74562 | 0.385 | 0.585 | 0.000108 | 19 | *SAT1* | Rod precursors |
| PAX6.7 | 1.54E-10 | -0.7586 | 0.139 | 0.415 | 3.40E-06 | 19 | *PAX6* | Rod precursors |
| MIAT.4 | 9.28E-07 | -0.76432 | 0.238 | 0.407 | 0.0205 | 19 | *MIAT* | Rod precursors |
| DAPL1.10 | 2.91E-09 | -0.77586 | 0.303 | 0.532 | 6.44E-05 | 19 | *DAPL1* | Rod precursors |
| ID3.3 | 2.54E-06 | -0.78005 | 0.287 | 0.469 | 0.056102 | 19 | *ID3* | Rod precursors |
| MEST.4 | 1.10E-12 | -0.80856 | 0.23 | 0.522 | 2.44E-08 | 19 | *MEST* | Rod precursors |
| UBE2C.12 | 0.003638 | -0.81681 | 0.107 | 0.201 | 1 | 19 | *UBE2C* | Rod precursors |
| HES1.9 | 2.13E-09 | -0.8258 | 0.139 | 0.392 | 4.69E-05 | 19 | *HES1* | Rod precursors |
| B2M.5 | 1.29E-12 | -0.8446 | 0.631 | 0.759 | 2.84E-08 | 19 | *B2M* | Rod precursors |
| GPC3.11 | 1.12E-06 | -0.85803 | 0.164 | 0.35 | 0.024778 | 19 | *GPC3* | Rod precursors |
| IFITM2.10 | 6.05E-09 | -0.88181 | 0.049 | 0.287 | 0.000134 | 19 | *IFITM2* | Rod precursors |
| EGR1.7 | 5.91E-12 | -0.89725 | 0.295 | 0.57 | 1.30E-07 | 19 | *EGR1* | Rod precursors |
| TOP2A.13 | 0.001931 | -0.91899 | 0.18 | 0.28 | 1 | 19 | *TOP2A* | Rod precursors |
| CENPF.13 | 0.000276 | -0.93053 | 0.172 | 0.304 | 1 | 19 | *CENPF* | Rod precursors |
| DIO3.11 | 1.09E-10 | -0.93222 | 0.041 | 0.315 | 2.42E-06 | 19 | *DIO3* | Rod precursors |
| IFITM3.12 | 9.11E-11 | -0.96072 | 0.082 | 0.36 | 2.01E-06 | 19 | *IFITM3* | Rod precursors |
| PRSS23.9 | 2.71E-12 | -0.96292 | 0.164 | 0.452 | 5.98E-08 | 19 | *PRSS23* | Rod precursors |
| TF.11 | 0.000164 | -0.9701 | 0.082 | 0.217 | 1 | 19 | *TF* | Rod precursors |
| IGFBP2.5 | 0.006336 | -0.97152 | 0.492 | 0.526 | 1 | 19 | *IGFBP2* | Rod precursors |
| PTN.7 | 8.52E-11 | -0.98226 | 0.279 | 0.544 | 1.88E-06 | 19 | *PTN* | Rod precursors |
| SOX2.9 | 1.49E-14 | -1.02045 | 0.082 | 0.417 | 3.30E-10 | 19 | *SOX2* | Rod precursors |
| LAMP5.12 | 1.90E-08 | -1.04187 | 0.098 | 0.329 | 0.000421 | 19 | *LAMP5* | Rod precursors |
| CCND1.9 | 2.12E-12 | -1.05394 | 0.148 | 0.459 | 4.68E-08 | 19 | *CCND1* | Rod precursors |
| ZFP36L1.11 | 2.45E-14 | -1.10269 | 0.123 | 0.45 | 5.41E-10 | 19 | *ZFP36L1* | Rod precursors |
| DKK3.9 | 1.87E-18 | -1.1311 | 0.09 | 0.496 | 4.13E-14 | 19 | *DKK3* | Rod precursors |
| COL1A2.9 | 7.29E-12 | -1.20797 | 0.09 | 0.379 | 1.61E-07 | 19 | *COL1A2* | Rod precursors |
| SPP1.14 | 5.56E-13 | -1.2278 | 0.18 | 0.501 | 1.23E-08 | 19 | *SPP1* | Rod precursors |
| TRH.9 | 8.24E-09 | -1.32262 | 0.369 | 0.575 | 0.000182 | 19 | *TRH* | Rod precursors |
| FOS.8 | 3.42E-16 | -1.33727 | 0.238 | 0.566 | 7.55E-12 | 19 | *FOS* | Rod precursors |
| CLU.11 | 2.09E-18 | -1.39313 | 0.385 | 0.678 | 4.61E-14 | 19 | *CLU* | Rod precursors |
| CYP26A1.1 | 9.00E-09 | -1.45651 | 0.238 | 0.462 | 0.000199 | 19 | *CYP26A1* | Rod precursors |
| VIM.10 | 2.14E-20 | -1.82307 | 0.672 | 0.805 | 4.73E-16 | 19 | *VIM* | Rod precursors |
| SFRP2.13 | 3.21E-12 | -1.82332 | 0.303 | 0.552 | 7.09E-08 | 19 | *SFRP2* | Rod precursors |
| CRABP1.10 | 4.19E-22 | -1.86811 | 0.5 | 0.745 | 9.25E-18 | 19 | *CRABP1* | Rod precursors |
| TFF1.2 | 4.78E-160 | 2.352843 | 0.667 | 0.054 | 1.06E-155 | 20 | *TFF1* | Cone precursors |
| HIST1H4C. | 1.88E-47 | 2.03237 | 0.9 | 0.58 | 4.14E-43 | 20 | *HIST1H4C* | Cone precursors |
| DCT.12 | 1.95E-46 | 1.464262 | 0.775 | 0.28 | 4.31E-42 | 20 | *DCT* | Cone precursors |
| RPS4Y1.2 | 2.13E-105 | 1.325788 | 0.358 | 0.022 | 4.70E-101 | 20 | *RPS4Y1* | Cone precursors |
| HMGA1.8 | 7.54E-37 | 1.249973 | 0.892 | 0.65 | 1.66E-32 | 20 | *HMGA1* | Cone precursors |
| SSTR2.2 | 1.73E-32 | 1.247534 | 0.592 | 0.21 | 3.82E-28 | 20 | *SSTR2* | Cone precursors |
| HSPA1A.1 | 2.11E-07 | 1.246293 | 0.367 | 0.223 | 0.004663 | 20 | *HSPA1A* | Cone precursors |
| TUBB.5 | 1.10E-44 | 1.207721 | 0.992 | 0.947 | 2.43E-40 | 20 | *TUBB* | Cone precursors |
| HLA-B.2 | 2.23E-05 | 1.117798 | 0.375 | 0.265 | 0.491394 | 20 | *HLA-B* | Cone precursors |
| DEK.5 | 9.98E-41 | 1.108075 | 0.983 | 0.861 | 2.20E-36 | 20 | *DEK* | Cone precursors |
| RCVRN.15 | 5.95E-39 | 1.104256 | 0.85 | 0.366 | 1.31E-34 | 20 | *RCVRN* | Cone precursors |
| TPH1.14 | 1.58E-24 | 1.042345 | 0.558 | 0.217 | 3.50E-20 | 20 | *TPH1* | Cone precursors |
| TPRX1.2 | 2.65E-66 | 0.994075 | 0.283 | 0.022 | 5.86E-62 | 20 | *TPRX1* | Cone precursors |
| GMNN.1 | 7.62E-12 | 0.975445 | 0.617 | 0.453 | 1.68E-07 | 20 | *GMNN* | Cone precursors |
| FBL.2 | 7.86E-11 | 0.972283 | 0.608 | 0.494 | 1.73E-06 | 20 | *FBL* | Cone precursors |
| IGDCC3.1 | 3.78E-13 | 0.959949 | 0.35 | 0.147 | 8.34E-09 | 20 | *IGDCC3* | Cone precursors |
| RRM2.1 | 3.71E-12 | 0.952663 | 0.4 | 0.193 | 8.18E-08 | 20 | *RRM2* | Cone precursors |
| CHCHD2.5 | 4.81E-23 | 0.924062 | 0.75 | 0.429 | 1.06E-18 | 20 | *CHCHD2* | Cone precursors |
| GAP43.2 | 9.81E-17 | 0.881303 | 0.542 | 0.274 | 2.17E-12 | 20 | *GAP43* | Cone precursors |

| SOX11.6 | 5.52E-18 | 0.879757 | 0.783 | 0.58 | 1.22E-13 | 20 *SOX11* | Cone precursors |
| --- | --- | --- | --- | --- | --- | --- | --- |
| HLA-DQB1. | 6.68E-17 | 0.875043 | 0.283 | 0.081 | 1.47E-12 | 20 *HLA-DQB1* | Cone precursors |
| STMN1.5 | 2.76E-33 | 0.869489 | 0.992 | 0.963 | 6.09E-29 | 20 *STMN1* | Cone precursors |
| AIPL1.15 | 5.24E-27 | 0.84399 | 0.742 | 0.321 | 1.16E-22 | 20 *AIPL1* | Cone precursors |
| MCM3.1 | 6.69E-08 | 0.843274 | 0.575 | 0.468 | 0.001478 | 20 *MCM3* | Cone precursors |
| SLC25A6 | 2.14E-13 | 0.832065 | 0.725 | 0.63 | 4.73E-09 | 20 *SLC25A6* | Cone precursors |
| TUBB2B.4 | 2.30E-26 | 0.830381 | 0.95 | 0.83 | 5.07E-22 | 20 *TUBB2B* | Cone precursors |
| KIFC1.2 | 5.27E-16 | 0.829938 | 0.375 | 0.139 | 1.16E-11 | 20 *KIFC1* | Cone precursors |
| HMGB1.2 | 6.78E-35 | 0.824557 | 0.983 | 0.954 | 1.50E-30 | 20 *HMGB1* | Cone precursors |
| STMN2.11 | 1.11E-24 | 0.807495 | 0.75 | 0.382 | 2.46E-20 | 20 *STMN2* | Cone precursors |
| NUDT3.1 | 1.41E-07 | 0.79166 | 0.475 | 0.346 | 0.003111 | 20 *NUDT3* | Cone precursors |
| CUTA | 1.48E-15 | 0.785284 | 0.817 | 0.776 | 3.26E-11 | 20 *CUTA* | Cone precursors |
| PIM1 | 2.63E-06 | 0.769271 | 0.308 | 0.18 | 0.05807 | 20 *PIM1* | Cone precursors |
| BRD2.1 | 1.84E-08 | 0.747348 | 0.592 | 0.515 | 0.000406 | 20 *BRD2* | Cone precursors |
| TULP1.13 | 5.85E-14 | 0.743961 | 0.55 | 0.293 | 1.29E-09 | 20 *TULP1* | Cone precursors |
| PPP1CC.2 | 1.55E-08 | 0.743053 | 0.617 | 0.553 | 0.000342 | 20 *PPP1CC* | Cone precursors |
| ATAT1 | 0.000863 | 0.741829 | 0.3 | 0.22 | 1 | 20 *ATAT1* | Cone precursors |
| RXRG.11 | 4.18E-14 | 0.741103 | 0.583 | 0.329 | 9.23E-10 | 20 *RXRG* | Cone precursors |
| YWHAQ | 6.76E-18 | 0.734521 | 0.883 | 0.833 | 1.49E-13 | 20 *YWHAQ* | Cone precursors |
| GNG4.1 | 9.20E-07 | 0.712671 | 0.433 | 0.298 | 0.020312 | 20 *GNG4* | Cone precursors |
| HMGB2.10 | 7.01E-26 | 0.711435 | 0.908 | 0.727 | 1.55E-21 | 20 *HMGB2* | Cone precursors |
| OLFM1.3 | 7.10E-08 | 0.701174 | 0.492 | 0.331 | 0.001568 | 20 *OLFM1* | Cone precursors |
| GLRX5 | 2.21E-06 | 0.700174 | 0.575 | 0.537 | 0.04881 | 20 *GLRX5* | Cone precursors |
| SPINT2 | 1.25E-17 | -0.70224 | 0.067 | 0.478 | 2.76E-13 | 20 *SPINT2* | Cone precursors |
| SAT1.4 | 6.13E-14 | -0.70666 | 0.192 | 0.59 | 1.35E-09 | 20 *SAT1* | Cone precursors |
| PDC.17 | 0.000254 | -0.7138 | 0.258 | 0.428 | 1 | 20 *PDC* | Cone precursors |
| BTG1.2 | 1.30E-14 | -0.72136 | 0.408 | 0.808 | 2.86E-10 | 20 *BTG1* | Cone precursors |
| CD81.1 | 1.92E-14 | -0.72716 | 0.15 | 0.532 | 4.23E-10 | 20 *CD81* | Cone precursors |
| ID3.4 | 8.36E-10 | -0.73604 | 0.167 | 0.471 | 1.85E-05 | 20 *ID3* | Cone precursors |
| MT-ND5 | 4.38E-24 | -0.77349 | 0.525 | 0.925 | 9.67E-20 | 20 *MT-ND5* | Cone precursors |
| COL18A1.5 | 1.63E-12 | -0.78457 | 0.025 | 0.338 | 3.61E-08 | 20 *COL18A1* | Cone precursors |
| CRYM.6 | 3.24E-10 | -0.7953 | 0.042 | 0.309 | 7.15E-06 | 20 *CRYM* | Cone precursors |
| FTL | 3.18E-25 | -0.82287 | 0.817 | 0.971 | 7.01E-21 | 20 *FTL* | Cone precursors |
| PLEKHA1.5 | 4.37E-15 | -0.82403 | 0.225 | 0.632 | 9.66E-11 | 20 *PLEKHA1* | Cone precursors |
| DHRS3.3 | 1.35E-15 | -0.83834 | 0.117 | 0.494 | 2.99E-11 | 20 *DHRS3* | Cone precursors |
| MT-CO1 | 1.05E-29 | -0.83866 | 0.975 | 0.994 | 2.31E-25 | 20 *MT-CO1* | Cone precursors |
| RBP1.1 | 1.00E-24 | -0.86786 | 0.567 | 0.923 | 2.22E-20 | 20 *RBP1* | Cone precursors |
| GPC3.12 | 1.40E-09 | -0.87205 | 0.083 | 0.352 | 3.08E-05 | 20 *GPC3* | Cone precursors |
| MEST.5 | 1.32E-18 | -0.88229 | 0.083 | 0.525 | 2.90E-14 | 20 *MEST* | Cone precursors |
| SOX2.10 | 3.73E-15 | -0.88592 | 0.042 | 0.417 | 8.24E-11 | 20 *SOX2* | Cone precursors |
| NEAT1.5 | 6.74E-19 | -0.88958 | 0.3 | 0.785 | 1.49E-14 | 20 *NEAT1* | Cone precursors |
| APOE.5 | 4.00E-12 | -0.89156 | 0.125 | 0.458 | 8.84E-08 | 20 *APOE* | Cone precursors |
| PRSS23.10 | 4.34E-13 | -0.89593 | 0.117 | 0.453 | 9.58E-09 | 20 *PRSS23* | Cone precursors |
| GPM6B.3 | 1.32E-19 | -0.9106 | 0.208 | 0.666 | 2.91E-15 | 20 *GPM6B* | Cone precursors |
| IFITM2.11 | 1.59E-10 | -0.91399 | 0.017 | 0.287 | 3.50E-06 | 20 *IFITM2* | Cone precursors |
| HES1.10 | 4.23E-14 | -0.91552 | 0.042 | 0.394 | 9.34E-10 | 20 *HES1* | Cone precursors |
| METRN.3 | 3.92E-15 | -0.93884 | 0.05 | 0.417 | 8.65E-11 | 20 *METRN* | Cone precursors |
| PTH2.3 | 6.36E-17 | -0.9569 | 0.117 | 0.532 | 1.40E-12 | 20 *PTH2* | Cone precursors |
| PTN.8 | 1.22E-13 | -0.96426 | 0.183 | 0.546 | 2.69E-09 | 20 *PTN* | Cone precursors |
| IGFBP5.7 | 3.90E-10 | -0.97042 | 0.133 | 0.439 | 8.61E-06 | 20 *IGFBP5* | Cone precursors |
| TF.12 | 3.96E-07 | -0.97411 | 0.025 | 0.219 | 0.00875 | 20 *TF* | Cone precursors |
| CRABP2.5 | 3.87E-18 | -0.97882 | 0.142 | 0.575 | 8.55E-14 | 20 *CRABP2* | Cone precursors |
| LAMP5.13 | 3.56E-09 | -1.00213 | 0.075 | 0.329 | 7.86E-05 | 20 *LAMP5* | Cone precursors |
| DIO3.12 | 1.28E-12 | -1.018 | 0.008 | 0.315 | 2.82E-08 | 20 *DIO3* | Cone precursors |
| IFITM3.13 | 3.03E-13 | -1.03426 | 0.033 | 0.361 | 6.70E-09 | 20 *IFITM3* | Cone precursors |
| TTYH1.11 | 1.16E-17 | -1.03619 | 0.058 | 0.463 | 2.56E-13 | 20 *TTYH1* | Cone precursors |
| DKK3.10 | 7.94E-19 | -1.09988 | 0.075 | 0.496 | 1.75E-14 | 20 *DKK3* | Cone precursors |
| DAPL1.11 | 1.89E-19 | -1.11404 | 0.1 | 0.537 | 4.18E-15 | 20 *DAPL1* | Cone precursors |
| ZFP36L1.12 | 1.37E-16 | -1.12847 | 0.067 | 0.451 | 3.02E-12 | 20 *ZFP36L1* | Cone precursors |
| CCND1.10 | 6.65E-17 | -1.16188 | 0.058 | 0.461 | 1.47E-12 | 20 *CCND1* | Cone precursors |

| COL1A2.10 | 1.56E-14 | -1.268 | 0.033 | 0.38 | 3.44E-10 | 20 *COL1A2* | Cone precursors |
| --- | --- | --- | --- | --- | --- | --- | --- |
| IGFBP2.6 | 1.28E-11 | -1.31912 | 0.217 | 0.532 | 2.82E-07 | 20 *IGFBP2* | Cone precursors |
| CLU.12 | 6.94E-24 | -1.38459 | 0.192 | 0.682 | 1.53E-19 | 20 *CLU* | Cone precursors |
| SPP1.15 | 6.61E-18 | -1.48904 | 0.092 | 0.503 | 1.46E-13 | 20 *SPP1* | Cone precursors |
| FABP7.7 | 2.29E-22 | -1.50096 | 0.15 | 0.623 | 5.06E-18 | 20 *FABP7* | Cone precursors |
| TRH.10 | 1.39E-17 | -1.51848 | 0.158 | 0.58 | 3.08E-13 | 20 *TRH* | Cone precursors |
| SFRP2.14 | 7.25E-16 | -1.70778 | 0.175 | 0.555 | 1.60E-11 | 20 *SFRP2* | Cone precursors |
| CYP26A1.1 | 1.08E-15 | -1.83276 | 0.092 | 0.465 | 2.38E-11 | 20 *CYP26A1* | Cone precursors |
| CRABP1.11 | 4.62E-34 | -2.00358 | 0.167 | 0.753 | 1.02E-29 | 20 *CRABP1* | Cone precursors |
| VIM.11 | 8.79E-35 | -2.09427 | 0.267 | 0.814 | 1.94E-30 | 20 *VIM* | Cone precursors |
| RTN1.1 | 2.22E-27 | 1.883027 | 0.595 | 0.188 | 4.90E-23 | 21 *RTN1* | Retinal ganglion cells |
| STMN2.12 | 6.92E-20 | 1.429118 | 0.722 | 0.386 | 1.53E-15 | 21 *STMN2* | Retinal ganglion cells |
| HOXB8.1 | 1.94E-16 | 1.249237 | 0.342 | 0.089 | 4.29E-12 | 21 *HOXB8* | Retinal ganglion cells |
| SYT4.1 | 0.002466 | 1.213828 | 0.253 | 0.16 | 1 | 21 *SYT4* | Retinal ganglion cells |
| PCSK1N.1 | 1.88E-08 | 1.196826 | 0.582 | 0.454 | 0.000414 | 21 *PCSK1N* | Retinal ganglion cells |
| STMN4.1 | 3.73E-12 | 1.187662 | 0.532 | 0.277 | 8.24E-08 | 21 *STMN4* | Retinal ganglion cells |
| TUBB2A.1 | 7.91E-13 | 1.179209 | 0.671 | 0.467 | 1.75E-08 | 21 *TUBB2A* | Retinal ganglion cells |
| NSG1.1 | 2.70E-22 | 1.139956 | 0.367 | 0.081 | 5.96E-18 | 21 *NSG1* | Retinal ganglion cells |
| APP | 0.000902 | 1.063483 | 0.506 | 0.507 | 1 | 21 *APP* | Retinal ganglion cells |
| NRXN1.1 | 9.08E-05 | 1.051938 | 0.367 | 0.24 | 1 | 21 *NRXN1* | Retinal ganglion cells |
| PLPPR3.1 | 1.15E-07 | 1.051925 | 0.468 | 0.296 | 0.00253 | 21 *PLPPR3* | Retinal ganglion cells |
| NSG2.1 | 7.30E-10 | 1.020732 | 0.316 | 0.118 | 1.61E-05 | 21 *NSG2* | Retinal ganglion cells |
| TMEM59L | 6.36E-07 | 1.016112 | 0.367 | 0.193 | 0.014044 | 21 *TMEM59L* | Retinal ganglion cells |
| TUBA1A.2 | 1.08E-14 | 1.013571 | 0.899 | 0.947 | 2.39E-10 | 21 *TUBA1A* | Retinal ganglion cells |
| HOXB5.1 | 4.34E-09 | 1.010692 | 0.342 | 0.141 | 9.59E-05 | 21 *HOXB5* | Retinal ganglion cells |
| PRPH.1 | 6.42E-10 | 0.98968 | 0.165 | 0.036 | 1.42E-05 | 21 *PRPH* | Retinal ganglion cells |
| SARAF | 7.95E-05 | 0.981729 | 0.646 | 0.744 | 1 | 21 *SARAF* | Retinal ganglion cells |
| SCG2 | 0.0065 | 0.962249 | 0.177 | 0.097 | 1 | 21 *SCG2* | Retinal ganglion cells |
| BASP1.4 | 3.87E-13 | 0.951792 | 0.823 | 0.722 | 8.55E-09 | 21 *BASP1* | Retinal ganglion cells |
| HOXB9.1 | 2.11E-11 | 0.927963 | 0.203 | 0.046 | 4.65E-07 | 21 *HOXB9* | Retinal ganglion cells |
| GPM6A.1 | 1.00E-05 | 0.894659 | 0.684 | 0.703 | 0.221426 | 21 *GPM6A* | Retinal ganglion cells |
| SYT1.1 | 0.00507 | 0.866162 | 0.582 | 0.635 | 1 | 21 *SYT1* | Retinal ganglion cells |
| SOX4.7 | 1.10E-09 | 0.841635 | 0.835 | 0.865 | 2.42E-05 | 21 *SOX4* | Retinal ganglion cells |
| EEF1A2.1 | 1.60E-05 | 0.829245 | 0.456 | 0.314 | 0.352577 | 21 *EEF1A2* | Retinal ganglion cells |
| CD24.4 | 9.61E-09 | 0.827672 | 0.633 | 0.451 | 0.000212 | 21 *CD24* | Retinal ganglion cells |
| FAM171A2 | 9.34E-05 | 0.825515 | 0.354 | 0.234 | 1 | 21 *FAM171A2* | Retinal ganglion cells |
| ANK3.1 | 2.61E-05 | 0.824227 | 0.418 | 0.286 | 0.576894 | 21 *ANK3* | Retinal ganglion cells |
| MAPT.1 | 2.51E-07 | 0.820509 | 0.316 | 0.142 | 0.005543 | 21 *MAPT* | Retinal ganglion cells |
| SERPINI1 | 0.00453 | 0.784947 | 0.114 | 0.048 | 1 | 21 *SERPINI1* | Retinal ganglion cells |
| PCMT1 | 0.007628 | 0.784568 | 0.506 | 0.527 | 1 | 21 *PCMT1* | Retinal ganglion cells |
| CRMP1.5 | 6.01E-08 | 0.775935 | 0.582 | 0.425 | 0.001328 | 21 *CRMP1* | Retinal ganglion cells |
| PODXL2 | 0.000776 | 0.769454 | 0.418 | 0.332 | 1 | 21 *PODXL2* | Retinal ganglion cells |
| CAMK2N1. | 0.004398 | 0.767716 | 0.456 | 0.45 | 1 | 21 *CAMK2N1* | Retinal ganglion cells |
| OLFM1.4 | 0.000717 | 0.765882 | 0.443 | 0.333 | 1 | 21 *OLFM1* | Retinal ganglion cells |
| DCX.1 | 4.15E-06 | 0.765766 | 0.354 | 0.193 | 0.091588 | 21 *DCX* | Retinal ganglion cells |
| ELAVL4.1 | 1.53E-09 | 0.755167 | 0.304 | 0.109 | 3.37E-05 | 21 *ELAVL4* | Retinal ganglion cells |
| TM2D3 | 0.000866 | 0.751032 | 0.443 | 0.369 | 1 | 21 *TM2D3* | Retinal ganglion cells |
| NDFIP1 | 0.008694 | 0.746155 | 0.544 | 0.62 | 1 | 21 *NDFIP1* | Retinal ganglion cells |
| GRIA2.1 | 1.14E-06 | 0.744818 | 0.215 | 0.079 | 0.025237 | 21 *GRIA2* | Retinal ganglion cells |
| PPP1R14B. | 0.001294 | 0.74338 | 0.456 | 0.384 | 1 | 21 *PPP1R14B* | Retinal ganglion cells |
| Mar-06 | 0.000114 | 0.741181 | 0.557 | 0.514 | 1 | 21 *Mar-06* | Retinal ganglion cells |
| C4orf48.1 | 0.005405 | 0.740883 | 0.557 | 0.611 | 1 | 21 *C4orf48* | Retinal ganglion cells |
| GAP43.3 | 0.001672 | 0.734644 | 0.38 | 0.278 | 1 | 21 *GAP43* | Retinal ganglion cells |
| CHL1 | 0.001676 | 0.73381 | 0.253 | 0.151 | 1 | 21 *CHL1* | Retinal ganglion cells |
| L1CAM | 0.003309 | 0.732938 | 0.241 | 0.151 | 1 | 21 *L1CAM* | Retinal ganglion cells |
| CD81.2 | 1.06E-05 | 0.727613 | 0.62 | 0.522 | 0.234932 | 21 *CD81* | Retinal ganglion cells |
| TUBB2B.5 | 1.70E-09 | 0.726272 | 0.81 | 0.833 | 3.74E-05 | 21 *TUBB2B* | Retinal ganglion cells |
| HOXB6.1 | 2.53E-06 | 0.718408 | 0.228 | 0.087 | 0.055813 | 21 *HOXB6* | Retinal ganglion cells |
| ELAVL3.1 | 0.003397 | 0.71591 | 0.342 | 0.257 | 1 | 21 *ELAVL3* | Retinal ganglion cells |
| CELF5.1 | 2.79E-05 | 0.712134 | 0.329 | 0.189 | 0.615409 | 21 *CELF5* | Retinal ganglion cells |

| TSHZ2 | 0.001998 | 0.707176 | 0.291 | 0.198 | 1 | 21 | *TSHZ2* | Retinal ganglion cells |
| --- | --- | --- | --- | --- | --- | --- | --- | --- |
| VGF | 4.97E-10 | 0.700785 | 0.152 | 0.031 | 1.10E-05 | 21 | *VGF* | Retinal ganglion cells |
| RPL27A | 2.86E-15 | -0.70298 | 0.532 | 0.919 | 6.31E-11 | 21 | *RPL27A* | Retinal ganglion cells |
| TMPO.1 | 6.91E-12 | -0.70359 | 0.139 | 0.586 | 1.53E-07 | 21 | *TMPO* | Retinal ganglion cells |
| TOP2A.14 | 0.003145 | -0.70427 | 0.127 | 0.28 | 1 | 21 | *TOP2A* | Retinal ganglion cells |
| IFITM3.14 | 1.17E-06 | -0.70572 | 0.076 | 0.358 | 0.025813 | 21 | *IFITM3* | Retinal ganglion cells |
| GPC3.13 | 1.55E-06 | -0.70645 | 0.076 | 0.35 | 0.034288 | 21 | *GPC3* | Retinal ganglion cells |
| RPS6.1 | 9.97E-19 | -0.71958 | 0.734 | 0.979 | 2.20E-14 | 21 | *RPS6* | Retinal ganglion cells |
| HMGB2.11 | 1.82E-06 | -0.72019 | 0.405 | 0.736 | 0.0401 | 21 | *HMGB2* | Retinal ganglion cells |
| DAPL1.12 | 8.41E-08 | -0.72174 | 0.19 | 0.532 | 0.001857 | 21 | *DAPL1* | Retinal ganglion cells |
| GNG5.1 | 5.18E-14 | -0.72216 | 0.304 | 0.811 | 1.14E-09 | 21 | *GNG5* | Retinal ganglion cells |
| CD9.3 | 1.43E-07 | -0.73049 | 0.025 | 0.306 | 0.00316 | 21 | *CD9* | Retinal ganglion cells |
| MDK.7 | 1.88E-10 | -0.73323 | 0.316 | 0.725 | 4.16E-06 | 21 | *MDK* | Retinal ganglion cells |
| UNC119.15 | 2.30E-06 | -0.734 | 0.228 | 0.525 | 0.050791 | 21 | *UNC119* | Retinal ganglion cells |
| DIO3.13 | 1.73E-06 | -0.74363 | 0.051 | 0.312 | 0.038239 | 21 | *DIO3* | Retinal ganglion cells |
| HMGN2.4 | 1.70E-11 | -0.75151 | 0.608 | 0.927 | 3.74E-07 | 21 | *HMGN2* | Retinal ganglion cells |
| SOX2.11 | 2.56E-08 | -0.7589 | 0.089 | 0.414 | 0.000566 | 21 | *SOX2* | Retinal ganglion cells |
| EGR1.8 | 2.37E-10 | -0.77608 | 0.152 | 0.57 | 5.23E-06 | 21 | *EGR1* | Retinal ganglion cells |
| HLA-A.2 | 8.10E-10 | -0.7778 | 0.089 | 0.455 | 1.79E-05 | 21 | *HLA-A* | Retinal ganglion cells |
| CYP1B1.9 | 6.19E-09 | -0.83223 | 0.063 | 0.4 | 0.000137 | 21 | *CYP1B1* | Retinal ganglion cells |
| AIPL1.16 | 1.06E-05 | -0.84867 | 0.101 | 0.334 | 0.233521 | 21 | *AIPL1* | Retinal ganglion cells |
| TRH.11 | 8.81E-05 | -0.86932 | 0.316 | 0.574 | 1 | 21 | *TRH* | Retinal ganglion cells |
| RPS20.1 | 1.52E-16 | -0.86953 | 0.316 | 0.837 | 3.36E-12 | 21 | *RPS20* | Retinal ganglion cells |
| GSTP1.1 | 4.00E-20 | -0.87816 | 0.481 | 0.922 | 8.83E-16 | 21 | *GSTP1* | Retinal ganglion cells |
| GNB3.14 | 1.63E-06 | -0.88062 | 0.152 | 0.424 | 0.036085 | 21 | *GNB3* | Retinal ganglion cells |
| CENPF.14 | 9.55E-06 | -0.88194 | 0.063 | 0.305 | 0.210938 | 21 | *CENPF* | Retinal ganglion cells |
| FOS.9 | 8.77E-09 | -0.88507 | 0.19 | 0.564 | 0.000194 | 21 | *FOS* | Retinal ganglion cells |
| RPL13A.1 | 7.92E-16 | -0.8857 | 0.532 | 0.926 | 1.75E-11 | 21 | *RPL13A* | Retinal ganglion cells |
| CLU.13 | 1.29E-08 | -0.88803 | 0.329 | 0.676 | 0.000284 | 21 | *CLU* | Retinal ganglion cells |
| ZFP36L2.5 | 5.61E-12 | -0.90256 | 0.038 | 0.451 | 1.24E-07 | 21 | *ZFP36L2* | Retinal ganglion cells |
| PDE6H.15 | 3.28E-05 | -0.95731 | 0.152 | 0.382 | 0.723437 | 21 | *PDE6H* | Retinal ganglion cells |
| NRL.14 | 0.004414 | -1.01212 | 0.19 | 0.344 | 1 | 21 | *NRL* | Retinal ganglion cells |
| DCT.13 | 7.14E-07 | -1.04174 | 0.038 | 0.294 | 0.015755 | 21 | *DCT* | Retinal ganglion cells |
| CYP26A1.1 | 5.98E-05 | -1.04308 | 0.228 | 0.46 | 1 | 21 | *CYP26A1* | Retinal ganglion cells |
| VIM.12 | 3.88E-07 | -1.06608 | 0.595 | 0.805 | 0.008572 | 21 | *VIM* | Retinal ganglion cells |
| DEK.6 | 4.53E-19 | -1.07354 | 0.316 | 0.872 | 1.00E-14 | 21 | *DEK* | Retinal ganglion cells |
| HIST1H4C. | 9.17E-06 | -1.22026 | 0.278 | 0.591 | 0.202421 | 21 | *HIST1H4C* | Retinal ganglion cells |
| SFRP2.15 | 4.19E-07 | -1.27238 | 0.253 | 0.551 | 0.009244 | 21 | *SFRP2* | Retinal ganglion cells |
| SPARCL1 | 1.27E-189 | 3.117267 | 0.848 | 0.056 | 2.81E-185 | 22 | *SPARCL1* | Astrocytes |
| CRYAB.1 | 1.94E-89 | 2.425908 | 0.684 | 0.078 | 4.28E-85 | 22 | *CRYAB* | Astrocytes |
| NMB | 2.39E-103 | 2.357402 | 0.671 | 0.063 | 5.28E-99 | 22 | *NMB* | Astrocytes |
| C1QL1.1 | 3.02E-49 | 2.158661 | 0.823 | 0.235 | 6.66E-45 | 22 | *C1QL1* | Astrocytes |
| SERPINI1.1 | 8.69E-160 | 2.089395 | 0.684 | 0.04 | 1.92E-155 | 22 | *SERPINI1* | Astrocytes |
| HOTAIRM1 | 4.56E-109 | 2.06537 | 0.772 | 0.085 | 1.01E-104 | 22 | *HOTAIRM1* | Astrocytes |
| POSTN | 0 | 2.048835 | 0.646 | 0.011 | 0 | 22 | *POSTN* | Astrocytes |
| AGT | 1.05E-114 | 1.953622 | 0.709 | 0.064 | 2.33E-110 | 22 | *AGT* | Astrocytes |
| MGST1 | 9.50E-84 | 1.684542 | 0.81 | 0.129 | 2.10E-79 | 22 | *MGST1* | Astrocytes |
| IGFBP2.7 | 3.72E-29 | 1.676632 | 0.899 | 0.52 | 8.21E-25 | 22 | *IGFBP2* | Astrocytes |
| PDLIM3 | 3.06E-49 | 1.613388 | 0.797 | 0.227 | 6.76E-45 | 22 | *PDLIM3* | Astrocytes |
| PTN.9 | 1.11E-22 | 1.575281 | 0.861 | 0.533 | 2.45E-18 | 22 | *PTN* | Astrocytes |
| ID3.5 | 2.13E-21 | 1.575188 | 0.797 | 0.46 | 4.71E-17 | 22 | *ID3* | Astrocytes |
| METRN.4 | 7.93E-34 | 1.564292 | 0.861 | 0.402 | 1.75E-29 | 22 | *METRN* | Astrocytes |
| CST3 | 2.71E-16 | 1.552731 | 0.886 | 0.579 | 5.99E-12 | 22 | *CST3* | Astrocytes |
| S100A10.1 | 3.49E-87 | 1.548256 | 0.658 | 0.07 | 7.70E-83 | 22 | *S100A10* | Astrocytes |
| GFAP | 1.61E-238 | 1.527864 | 0.456 | 0.008 | 3.55E-234 | 22 | *GFAP* | Astrocytes |
| C1orf61 | 2.64E-87 | 1.522733 | 0.494 | 0.039 | 5.84E-83 | 22 | *C1orf61* | Astrocytes |
| CEBPD | 1.31E-69 | 1.485163 | 0.797 | 0.143 | 2.88E-65 | 22 | *CEBPD* | Astrocytes |
| GOLIM4 | 1.95E-34 | 1.478079 | 0.848 | 0.383 | 4.31E-30 | 22 | *GOLIM4* | Astrocytes |
| HOPX | 2.49E-41 | 1.452059 | 0.557 | 0.103 | 5.51E-37 | 22 | *HOPX* | Astrocytes |
| SAT1.5 | 2.08E-31 | 1.446587 | 0.924 | 0.576 | 4.58E-27 | 22 | *SAT1* | Astrocytes |

| ID4 | 1.82E-43 | 1.432709 | 0.772 | 0.217 | 4.01E-39 | 22 | *ID4* | Astrocytes |
| --- | --- | --- | --- | --- | --- | --- | --- | --- |
| IGFBP7.3 | 8.60E-22 | 1.406269 | 0.62 | 0.2 | 1.90E-17 | 22 | *IGFBP7* | Astrocytes |
| PTGDS.7 | 1.02E-57 | 1.380371 | 0.848 | 0.182 | 2.24E-53 | 22 | *PTGDS* | Astrocytes |
| HOXB9.2 | 1.46E-218 | 1.37584 | 0.797 | 0.037 | 3.22E-214 | 22 | *HOXB9* | Astrocytes |
| SKAP2 | 5.79E-127 | 1.339989 | 0.696 | 0.055 | 1.28E-122 | 22 | *SKAP2* | Astrocytes |
| ITM2A | 4.74E-69 | 1.329902 | 0.582 | 0.069 | 1.05E-64 | 22 | *ITM2A* | Astrocytes |
| IGFBP5.8 | 2.32E-22 | 1.324518 | 0.861 | 0.426 | 5.12E-18 | 22 | *IGFBP5* | Astrocytes |
| TMOD1 | 1.31E-135 | 1.318686 | 0.582 | 0.034 | 2.89E-131 | 22 | *TMOD1* | Astrocytes |
| HOXB7.1 | 3.46E-131 | 1.300425 | 0.747 | 0.061 | 7.63E-127 | 22 | *HOXB7* | Astrocytes |
| SFRP4 | 9.92E-230 | 1.285046 | 0.608 | 0.018 | 2.19E-225 | 22 | *SFRP4* | Astrocytes |
| NFIA | 4.92E-73 | 1.280088 | 0.797 | 0.137 | 1.09E-68 | 22 | *NFIA* | Astrocytes |
| VIM.13 | 6.16E-28 | 1.265483 | 0.987 | 0.799 | 1.36E-23 | 22 | *VIM* | Astrocytes |
| APOE.6 | 1.99E-18 | 1.265215 | 0.823 | 0.445 | 4.40E-14 | 22 | *APOE* | Astrocytes |
| CD81.3 | 3.26E-31 | 1.25649 | 0.873 | 0.519 | 7.20E-27 | 22 | *CD81* | Astrocytes |
| CLU.14 | 7.43E-24 | 1.251401 | 0.975 | 0.667 | 1.64E-19 | 22 | *CLU* | Astrocytes |
| HOXB8.2 | 4.00E-105 | 1.250944 | 0.785 | 0.083 | 8.83E-101 | 22 | *HOXB8* | Astrocytes |
| S100B | 1.84E-174 | 1.243493 | 0.696 | 0.037 | 4.05E-170 | 22 | *S100B* | Astrocytes |
| GPC3.14 | 3.14E-28 | 1.240955 | 0.848 | 0.338 | 6.93E-24 | 22 | *GPC3* | Astrocytes |
| RGMA | 2.45E-189 | 1.223152 | 0.658 | 0.029 | 5.41E-185 | 22 | *RGMA* | Astrocytes |
| ANGPTL4 | 3.48E-118 | 1.216555 | 0.671 | 0.052 | 7.68E-114 | 22 | *ANGPTL4* | Astrocytes |
| LAPTM4B | 1.15E-18 | 1.176303 | 0.848 | 0.629 | 2.54E-14 | 22 | *LAPTM4B* | Astrocytes |
| CD9.4 | 3.92E-36 | 1.176209 | 0.848 | 0.294 | 8.66E-32 | 22 | *CD9* | Astrocytes |
| DHRS3.4 | 9.86E-30 | 1.148928 | 0.886 | 0.48 | 2.18E-25 | 22 | *DHRS3* | Astrocytes |
| FAM181B | 0 | 1.139756 | 0.646 | 0.011 | 0 | 22 | *FAM181B* | Astrocytes |
| CD99 | 5.29E-31 | 1.122614 | 0.861 | 0.388 | 1.17E-26 | 22 | *CD99* | Astrocytes |
| CFI.1 | 2.78E-48 | 1.105131 | 0.759 | 0.17 | 6.14E-44 | 22 | *CFI* | Astrocytes |
| BAALC.1 | 1.67E-27 | 1.09842 | 0.823 | 0.33 | 3.68E-23 | 22 | *BAALC* | Astrocytes |
| GPM6B.4 | 1.65E-23 | 1.056587 | 0.911 | 0.652 | 3.64E-19 | 22 | *GPM6B* | Astrocytes |
| SERPINE2 | 1.12E-25 | 1.045699 | 0.709 | 0.238 | 2.47E-21 | 22 | *SERPINE2* | Astrocytes |
| PRCP | 5.00E-42 | 1.043114 | 0.785 | 0.217 | 1.10E-37 | 22 | *PRCP* | Astrocytes |
| PSAP | 8.56E-27 | 1.031236 | 0.924 | 0.574 | 1.89E-22 | 22 | *PSAP* | Astrocytes |
| F3 | 1.50E-64 | 1.023191 | 0.544 | 0.063 | 3.31E-60 | 22 | *F3* | Astrocytes |
| A2M | 1.64E-75 | 1.021499 | 0.468 | 0.039 | 3.62E-71 | 22 | *A2M* | Astrocytes |
| CDO1 | 2.37E-35 | 0.986369 | 0.722 | 0.206 | 5.23E-31 | 22 | *CDO1* | Astrocytes |
| SCRG1 | 1.52E-45 | 0.98499 | 0.468 | 0.066 | 3.37E-41 | 22 | *SCRG1* | Astrocytes |
| SOX2.12 | 2.42E-22 | 0.967865 | 0.848 | 0.403 | 5.34E-18 | 22 | *SOX2* | Astrocytes |
| CHL1.1 | 2.67E-51 | 0.946496 | 0.734 | 0.143 | 5.89E-47 | 22 | *CHL1* | Astrocytes |
| HILPDA | 3.56E-17 | 0.937499 | 0.658 | 0.292 | 7.86E-13 | 22 | *HILPDA* | Astrocytes |
| BRINP1 | 7.98E-90 | 0.936639 | 0.671 | 0.069 | 1.76E-85 | 22 | *BRINP1* | Astrocytes |
| CHPF | 1.47E-28 | 0.932805 | 0.759 | 0.259 | 3.25E-24 | 22 | *CHPF* | Astrocytes |
| IGFBP4 | 3.09E-35 | 0.909083 | 0.709 | 0.195 | 6.82E-31 | 22 | *IGFBP4* | Astrocytes |
| LMO4.1 | 2.91E-16 | 0.908027 | 0.722 | 0.333 | 6.43E-12 | 22 | *LMO4* | Astrocytes |
| PPP1R17 | 1.39E-152 | 0.899311 | 0.418 | 0.013 | 3.07E-148 | 22 | *PPP1R17* | Astrocytes |
| LINC02381 | 5.03E-63 | 0.884603 | 0.722 | 0.112 | 1.11E-58 | 22 | *LINC02381* | Astrocytes |
| MIR99AHG | 5.95E-64 | 0.863365 | 0.658 | 0.095 | 1.31E-59 | 22 | *MIR99AHG* | Astrocytes |
| G0S2 | 1.09E-25 | 0.861205 | 0.456 | 0.101 | 2.42E-21 | 22 | *G0S2* | Astrocytes |
| HOXB6.2 | 1.63E-96 | 0.860597 | 0.759 | 0.079 | 3.59E-92 | 22 | *HOXB6* | Astrocytes |
| ELN | 6.26E-72 | 0.853401 | 0.544 | 0.055 | 1.38E-67 | 22 | *ELN* | Astrocytes |
| CA14 | 3.22E-33 | 0.853126 | 0.608 | 0.138 | 7.11E-29 | 22 | *CA14* | Astrocytes |
| PLTP | 8.92E-27 | 0.852481 | 0.797 | 0.322 | 1.97E-22 | 22 | *PLTP* | Astrocytes |
| CNN3.1 | 9.40E-19 | 0.843168 | 0.886 | 0.622 | 2.07E-14 | 22 | *CNN3* | Astrocytes |
| NDUFA4L2 | 7.76E-10 | 0.842248 | 0.241 | 0.07 | 1.71E-05 | 22 | *NDUFA4L2* | Astrocytes |
| VEGFA.1 | 1.22E-13 | 0.840531 | 0.671 | 0.317 | 2.69E-09 | 22 | *VEGFA* | Astrocytes |
| ITM2B.1 | 1.97E-25 | 0.83632 | 0.949 | 0.855 | 4.35E-21 | 22 | *ITM2B* | Astrocytes |
| SLC1A3.1 | 1.29E-26 | 0.829627 | 0.557 | 0.135 | 2.86E-22 | 22 | *SLC1A3* | Astrocytes |
| PON2 | 3.55E-33 | 0.828266 | 0.772 | 0.233 | 7.83E-29 | 22 | *PON2* | Astrocytes |
| VCAN | 1.26E-21 | 0.825581 | 0.544 | 0.151 | 2.78E-17 | 22 | *VCAN* | Astrocytes |
| ANXA2.1 | 3.52E-20 | 0.816948 | 0.734 | 0.318 | 7.77E-16 | 22 | *ANXA2* | Astrocytes |
| HOXB4.1 | 3.52E-75 | 0.808389 | 0.684 | 0.084 | 7.78E-71 | 22 | *HOXB4* | Astrocytes |
| NOVA1.1 | 5.00E-27 | 0.806999 | 0.772 | 0.278 | 1.10E-22 | 22 | *NOVA1* | Astrocytes |

| HOXB-AS3 | 3.63E-132 | 0.806841 | 0.544 | 0.028 | 8.01E-128 | 22 | *HOXB-AS3* | Astrocytes |
| --- | --- | --- | --- | --- | --- | --- | --- | --- |
| SDC3 | 6.59E-40 | 0.80677 | 0.595 | 0.114 | 1.46E-35 | 22 | *SDC3* | Astrocytes |
| BCAN | 9.74E-123 | 0.799061 | 0.316 | 0.009 | 2.15E-118 | 22 | *BCAN* | Astrocytes |
| DCLK1 | 6.54E-25 | 0.793565 | 0.759 | 0.301 | 1.44E-20 | 22 | *DCLK1* | Astrocytes |
| ID2 | 2.67E-25 | 0.79033 | 0.709 | 0.227 | 5.90E-21 | 22 | *ID2* | Astrocytes |
| TKT.1 | 4.80E-19 | 0.786098 | 0.924 | 0.674 | 1.06E-14 | 22 | *TKT* | Astrocytes |
| JUNB.3 | 1.09E-14 | 0.783621 | 0.848 | 0.576 | 2.40E-10 | 22 | *JUNB* | Astrocytes |
| TMEM47 | 1.65E-38 | 0.774002 | 0.759 | 0.197 | 3.65E-34 | 22 | *TMEM47* | Astrocytes |
| LINC00461 | 1.11E-23 | 0.773207 | 0.772 | 0.281 | 2.44E-19 | 22 | *LINC00461* | Astrocytes |
| TSC22D4 | 6.19E-41 | 0.767412 | 0.785 | 0.196 | 1.37E-36 | 22 | *TSC22D4* | Astrocytes |
| TUBB2A.2 | 8.03E-11 | 0.767203 | 0.734 | 0.466 | 1.77E-06 | 22 | *TUBB2A* | Astrocytes |
| HOXA3 | 4.14E-208 | 0.765314 | 0.57 | 0.018 | 9.14E-204 | 22 | *HOXA3* | Astrocytes |
| FRZB | 2.96E-09 | 0.764557 | 0.304 | 0.104 | 6.54E-05 | 22 | *FRZB* | Astrocytes |
| LY6H | 2.70E-22 | 0.763975 | 0.557 | 0.164 | 5.96E-18 | 22 | *LY6H* | Astrocytes |
| ATP6AP2 | 3.98E-19 | 0.761262 | 0.899 | 0.612 | 8.80E-15 | 22 | *ATP6AP2* | Astrocytes |
| SELENOM | 1.24E-21 | 0.760806 | 0.747 | 0.326 | 2.73E-17 | 22 | *SELENOM* | Astrocytes |
| HOXA5 | 1.16E-193 | 0.758641 | 0.468 | 0.012 | 2.56E-189 | 22 | *HOXA5* | Astrocytes |
| FOS.10 | 1.96E-14 | 0.757816 | 0.835 | 0.554 | 4.33E-10 | 22 | *FOS* | Astrocytes |
| GAS6 | 6.06E-30 | 0.755496 | 0.696 | 0.206 | 1.34E-25 | 22 | *GAS6* | Astrocytes |
| ATP1A2 | 4.40E-95 | 0.754822 | 0.633 | 0.055 | 9.72E-91 | 22 | *ATP1A2* | Astrocytes |
| FXYD6 | 2.64E-15 | 0.752433 | 0.886 | 0.529 | 5.83E-11 | 22 | *FXYD6* | Astrocytes |
| HOXB3.1 | 3.38E-54 | 0.745561 | 0.747 | 0.135 | 7.46E-50 | 22 | *HOXB3* | Astrocytes |
| NLRP1 | 7.24E-11 | 0.745021 | 0.797 | 0.546 | 1.60E-06 | 22 | *NLRP1* | Astrocytes |
| PLA2G16 | 3.61E-18 | 0.74496 | 0.772 | 0.362 | 7.98E-14 | 22 | *PLA2G16* | Astrocytes |
| EDNRB | 2.56E-33 | 0.737794 | 0.468 | 0.083 | 5.66E-29 | 22 | *EDNRB* | Astrocytes |
| CYP26B1.1 | 1.21E-23 | 0.737127 | 0.684 | 0.212 | 2.67E-19 | 22 | *CYP26B1* | Astrocytes |
| CYP26A1.1 | 1.28E-06 | 0.736473 | 0.709 | 0.453 | 0.028189 | 22 | *CYP26A1* | Astrocytes |
| ZFP36L2.6 | 1.91E-15 | 0.726585 | 0.81 | 0.44 | 4.21E-11 | 22 | *ZFP36L2* | Astrocytes |
| SMIM3 | 3.43E-49 | 0.726396 | 0.557 | 0.083 | 7.58E-45 | 22 | *SMIM3* | Astrocytes |
| LGALS3.2 | 7.39E-28 | 0.725598 | 0.684 | 0.19 | 1.63E-23 | 22 | *LGALS3* | Astrocytes |
| SEMA3C | 1.43E-76 | 0.723012 | 0.481 | 0.04 | 3.16E-72 | 22 | *SEMA3C* | Astrocytes |
| GATM | 2.97E-29 | 0.721074 | 0.519 | 0.116 | 6.56E-25 | 22 | *GATM* | Astrocytes |
| RPS27L | 1.53E-13 | 0.720071 | 0.911 | 0.711 | 3.37E-09 | 22 | *RPS27L* | Astrocytes |
| HOXB2.2 | 9.06E-30 | 0.719223 | 0.785 | 0.246 | 2.00E-25 | 22 | *HOXB2* | Astrocytes |
| TIMP1.1 | 2.58E-18 | 0.716332 | 0.835 | 0.487 | 5.70E-14 | 22 | *TIMP1* | Astrocytes |
| WLS | 6.34E-109 | 0.707929 | 0.481 | 0.027 | 1.40E-104 | 22 | *WLS* | Astrocytes |
| ZIC1.1 | 2.46E-19 | 0.703795 | 0.62 | 0.221 | 5.43E-15 | 22 | *ZIC1* | Astrocytes |
| SRI | 1.27E-15 | 0.703093 | 0.823 | 0.569 | 2.81E-11 | 22 | *SRI* | Astrocytes |
| NTRK2 | 2.94E-25 | 0.700087 | 0.468 | 0.106 | 6.49E-21 | 22 | *NTRK2* | Astrocytes |
| CNTLN | 6.70E-09 | -0.70313 | 0.291 | 0.548 | 0.000148 | 22 | *CNTLN* | Astrocytes |
| TYMS.6 | 7.38E-05 | -0.70389 | 0.582 | 0.625 | 1 | 22 | *TYMS* | Astrocytes |
| NUSAP1.11 | 2.09E-05 | -0.70687 | 0.114 | 0.338 | 0.462072 | 22 | *NUSAP1* | Astrocytes |
| CHODL.2 | 2.02E-05 | -0.72387 | 0.025 | 0.222 | 0.447064 | 22 | *CHODL* | Astrocytes |
| RXRG.12 | 0.000451 | -0.72403 | 0.203 | 0.337 | 1 | 22 | *RXRG* | Astrocytes |
| RPL13A.2 | 2.52E-11 | -0.72508 | 0.924 | 0.92 | 5.57E-07 | 22 | *RPL13A* | Astrocytes |
| TPH1.15 | 0.001513 | -0.72729 | 0.089 | 0.226 | 1 | 22 | *TPH1* | Astrocytes |
| LINC00599 | 1.31E-06 | -0.7349 | 0.063 | 0.302 | 0.028843 | 22 | *LINC00599* | Astrocytes |
| AKAP9.12 | 0.000799 | -0.74147 | 0.709 | 0.656 | 1 | 22 | *AKAP9* | Astrocytes |
| H2AFZ.3 | 1.51E-15 | -0.75815 | 0.848 | 0.938 | 3.33E-11 | 22 | *H2AFZ* | Astrocytes |
| EPB41.1 | 1.69E-10 | -0.7612 | 0.152 | 0.523 | 3.73E-06 | 22 | *EPB41* | Astrocytes |
| UBE2S.8 | 2.84E-05 | -0.76396 | 0.633 | 0.666 | 0.626669 | 22 | *UBE2S* | Astrocytes |
| CKB.4 | 2.49E-12 | -0.76493 | 0.949 | 0.971 | 5.49E-08 | 22 | *CKB* | Astrocytes |
| DIO3.14 | 0.000503 | -0.76753 | 0.152 | 0.311 | 1 | 22 | *DIO3* | Astrocytes |
| RGS16.2 | 3.57E-07 | -0.77168 | 0.38 | 0.605 | 0.007888 | 22 | *RGS16* | Astrocytes |
| UBE2C.13 | 0.000874 | -0.77838 | 0.051 | 0.201 | 1 | 22 | *UBE2C* | Astrocytes |
| PCLAF.5 | 1.10E-06 | -0.77869 | 0.127 | 0.38 | 0.02429 | 22 | *PCLAF* | Astrocytes |
| TULP1.14 | 6.91E-07 | -0.78496 | 0.051 | 0.303 | 0.015267 | 22 | *TULP1* | Astrocytes |
| BASP1.5 | 7.76E-07 | -0.79035 | 0.671 | 0.724 | 0.017145 | 22 | *BASP1* | Astrocytes |
| VXN.11 | 4.58E-06 | -0.79725 | 0.038 | 0.258 | 0.101116 | 22 | *VXN* | Astrocytes |
| SOX11.7 | 0.000389 | -0.79828 | 0.557 | 0.585 | 1 | 22 | *SOX11* | Astrocytes |

| SYP.11 | 3.78E-06 | -0.81821 | 0.19 | 0.389 | 0.083377 | 22 | *SYP* | Astrocytes |
| --- | --- | --- | --- | --- | --- | --- | --- | --- |
| CYP1B1.10 | 3.61E-06 | -0.82486 | 0.165 | 0.399 | 0.079806 | 22 | *CYP1B1* | Astrocytes |
| ATP1A3.10 | 1.98E-06 | -0.84583 | 0.127 | 0.35 | 0.043816 | 22 | *ATP1A3* | Astrocytes |
| GADD45G. | 2.98E-08 | -0.85509 | 0.19 | 0.471 | 0.000658 | 22 | *GADD45G* | Astrocytes |
| OTX2.9 | 1.22E-06 | -0.87094 | 0.127 | 0.359 | 0.027028 | 22 | *OTX2* | Astrocytes |
| GADD45A. | 0.000166 | -0.88316 | 0.43 | 0.529 | 1 | 22 | *GADD45A* | Astrocytes |
| HMGA1.9 | 7.11E-08 | -0.89132 | 0.57 | 0.657 | 0.00157 | 22 | *HMGA1* | Astrocytes |
| RRAD.14 | 0.00584 | -0.89499 | 0.215 | 0.32 | 1 | 22 | *RRAD* | Astrocytes |
| DAPL1.13 | 8.26E-06 | -0.89576 | 0.38 | 0.529 | 0.182332 | 22 | *DAPL1* | Astrocytes |
| TOP2A.15 | 0.000462 | -0.90259 | 0.114 | 0.28 | 1 | 22 | *TOP2A* | Astrocytes |
| PTMA.1 | 9.08E-30 | -0.91164 | 0.949 | 0.99 | 2.01E-25 | 22 | *PTMA* | Astrocytes |
| CRX.13 | 2.97E-08 | -0.92479 | 0.038 | 0.329 | 0.000656 | 22 | *CRX* | Astrocytes |
| CCND1.11 | 0.000279 | -0.93087 | 0.329 | 0.453 | 1 | 22 | *CCND1* | Astrocytes |
| SLC38A5.1 | 2.53E-06 | -0.93497 | 0.101 | 0.33 | 0.05596 | 22 | *SLC38A5* | Astrocytes |
| TMSB15A. | 1.79E-12 | -0.93723 | 0.367 | 0.643 | 3.94E-08 | 22 | *TMSB15A* | Astrocytes |
| HMGN2.5 | 2.20E-17 | -0.94522 | 0.899 | 0.923 | 4.87E-13 | 22 | *HMGN2* | Astrocytes |
| PMAIP1.5 | 8.47E-11 | -0.97169 | 0.051 | 0.431 | 1.87E-06 | 22 | *PMAIP1* | Astrocytes |
| DEK.7 | 2.75E-15 | -0.98036 | 0.835 | 0.864 | 6.06E-11 | 22 | *DEK* | Astrocytes |
| SEPT4.13 | 1.04E-07 | -0.98693 | 0.139 | 0.398 | 0.002303 | 22 | *Sep-04* | Astrocytes |
| UNC119.16 | 0.000917 | -0.99385 | 0.506 | 0.521 | 1 | 22 | *UNC119* | Astrocytes |
| PCBP4.10 | 3.34E-08 | -0.99498 | 0.278 | 0.519 | 0.000739 | 22 | *PCBP4* | Astrocytes |
| CENPF.15 | 4.40E-05 | -1.0114 | 0.101 | 0.304 | 0.970599 | 22 | *CENPF* | Astrocytes |
| HMGB2.12 | 3.54E-09 | -1.02101 | 0.62 | 0.732 | 7.82E-05 | 22 | *HMGB2* | Astrocytes |
| DCT.14 | 1.86E-05 | -1.02289 | 0.089 | 0.294 | 0.409761 | 22 | *DCT* | Astrocytes |
| MAB21L1. | 5.80E-16 | -1.03171 | 0.177 | 0.646 | 1.28E-11 | 22 | *MAB21L1* | Astrocytes |
| AMER2.11 | 4.31E-08 | -1.03222 | 0.266 | 0.499 | 0.000953 | 22 | *AMER2* | Astrocytes |
| NNAT.2 | 3.94E-12 | -1.03238 | 0.316 | 0.613 | 8.70E-08 | 22 | *NNAT* | Astrocytes |
| NEUROD1. | 1.42E-07 | -1.03596 | 0.177 | 0.437 | 0.003131 | 22 | *NEUROD1* | Astrocytes |
| PTH2.4 | 4.07E-12 | -1.04283 | 0.127 | 0.528 | 8.98E-08 | 22 | *PTH2* | Astrocytes |
| AIPL1.17 | 3.60E-06 | -1.06715 | 0.101 | 0.334 | 0.079454 | 22 | *AIPL1* | Astrocytes |
| TUBB.6 | 1.83E-18 | -1.08808 | 0.899 | 0.949 | 4.03E-14 | 22 | *TUBB* | Astrocytes |
| SIX6.1 | 1.34E-19 | -1.09673 | 0.165 | 0.704 | 2.96E-15 | 22 | *SIX6* | Astrocytes |
| SOX4.8 | 2.27E-12 | -1.09752 | 0.835 | 0.865 | 5.02E-08 | 22 | *SOX4* | Astrocytes |
| SFRP2.16 | 2.58E-07 | -1.11856 | 0.304 | 0.55 | 0.0057 | 22 | *SFRP2* | Astrocytes |
| FAM57B.1 | 8.85E-08 | -1.12735 | 0.241 | 0.464 | 0.001954 | 22 | *FAM57B* | Astrocytes |
| RORB.2 | 2.13E-17 | -1.17264 | 0.089 | 0.594 | 4.71E-13 | 22 | *RORB* | Astrocytes |
| GNB3.15 | 3.65E-06 | -1.18785 | 0.228 | 0.422 | 0.080621 | 22 | *GNB3* | Astrocytes |
| STMN1.6 | 5.26E-27 | -1.22772 | 0.937 | 0.964 | 1.16E-22 | 22 | *STMN1* | Astrocytes |
| SCG3.13 | 1.65E-10 | -1.3545 | 0.228 | 0.532 | 3.64E-06 | 22 | *SCG3* | Astrocytes |
| STMN2.13 | 6.25E-09 | -1.38245 | 0.089 | 0.395 | 0.000138 | 22 | *STMN2* | Astrocytes |
| NRL.15 | 7.14E-05 | -1.40505 | 0.165 | 0.345 | 1 | 22 | *NRL* | Astrocytes |
| PDE6H.16 | 2.62E-07 | -1.44144 | 0.127 | 0.382 | 0.005788 | 22 | *PDE6H* | Astrocytes |
| CRABP1.12 | 5.95E-12 | -1.4427 | 0.519 | 0.743 | 1.31E-07 | 22 | *CRABP1* | Astrocytes |
| HIST1H4C. | 0.009619 | -1.45438 | 0.62 | 0.586 | 1 | 22 | *HIST1H4C* | Astrocytes |
| TRH.12 | 9.34E-12 | -1.47142 | 0.177 | 0.576 | 2.06E-07 | 22 | *TRH* | Astrocytes |
| HES6.7 | 4.65E-11 | -1.49409 | 0.456 | 0.664 | 1.03E-06 | 22 | *HES6* | Astrocytes |
| PDC.18 | 1.20E-06 | -1.52043 | 0.203 | 0.427 | 0.026516 | 22 | *PDC* | Astrocytes |
| RCVRN.16 | 0.000328 | -1.55746 | 0.241 | 0.379 | 1 | 22 | *RCVRN* | Astrocytes |
| KRT19 | 1.06E-55 | 2.183969 | 0.329 | 0.026 | 2.35E-51 | 23 | *KRT19* | Ocular surface epithelium |
| S100A11.1 | 4.45E-38 | 1.915317 | 0.532 | 0.102 | 9.83E-34 | 23 | *S100A11* | Ocular surface epithelium |
| GDF15 | 4.05E-65 | 1.909621 | 0.456 | 0.045 | 8.94E-61 | 23 | *GDF15* | Ocular surface epithelium |
| ANXA1 | 1.06E-105 | 1.780437 | 0.57 | 0.042 | 2.34E-101 | 23 | *ANXA1* | Ocular surface epithelium |
| KRT18.1 | 3.68E-17 | 1.756614 | 0.43 | 0.13 | 8.13E-13 | 23 | *KRT18* | Ocular surface epithelium |
| SLPI | 7.69E-55 | 1.685572 | 0.139 | 0.004 | 1.70E-50 | 23 | *SLPI* | Ocular surface epithelium |
| KRT17 | 8.54E-15 | 1.649738 | 0.101 | 0.01 | 1.89E-10 | 23 | *KRT17* | Ocular surface epithelium |
| S100A10.2 | 1.59E-36 | 1.629452 | 0.443 | 0.073 | 3.52E-32 | 23 | *S100A10* | Ocular surface epithelium |
| S100A6.1 | 3.25E-16 | 1.615536 | 0.797 | 0.49 | 7.17E-12 | 23 | *S100A6* | Ocular surface epithelium |
| FTL.1 | 2.06E-22 | 1.594703 | 0.987 | 0.967 | 4.55E-18 | 23 | *FTL* | Ocular surface epithelium |
| ANXA2.2 | 2.17E-25 | 1.453172 | 0.759 | 0.318 | 4.78E-21 | 23 | *ANXA2* | Ocular surface epithelium |
| KRT13 | 3.20E-95 | 1.437759 | 0.101 | 0 | 7.06E-91 | 23 | *KRT13* | Ocular surface epithelium |

| 3.99E-23 | 1.437343 | 0.532 | 0.158 | 8.81E-19 | 23 *KRT8* | Ocular surface epithelium |
| --- | --- | --- | --- | --- | --- | --- |
| 3.17E-20 | 1.331289 | 0.785 | 0.481 | 7.01E-16 | 23 *SQSTM1* | Ocular surface epithelium |
| 0.005906 | 1.311433 | 0.329 | 0.223 | 1 | 23 *MT2A* | Ocular surface epithelium |
| 1.73E-15 | 1.289033 | 0.506 | 0.204 | 3.82E-11 | 23 *ATF5* | Ocular surface epithelium |
| 8.01E-87 | 1.283973 | 0.127 | 0.001 | 1.77E-82 | 23 *TACSTD2* | Ocular surface epithelium |
| 2.90E-08 | 1.22489 | 0.241 | 0.08 | 0.000641 | 23 *RARRES1* | Ocular surface epithelium |
| 7.47E-31 | 1.214845 | 0.671 | 0.19 | 1.65E-26 | 23 *LGALS3* | Ocular surface epithelium |
| 0.00011 | 1.209869 | 0.19 | 0.077 | 1 | 23 *CCL2* | Ocular surface epithelium |
| 4.80E-26 | 1.189134 | 0.975 | 0.986 | 1.06E-21 | 23 *FTH1* | Ocular surface epithelium |
| 1.87E-40 | 1.16452 | 0.582 | 0.118 | 4.13E-36 | 23 *ATF3* | Ocular surface epithelium |
| 2.55E-06 | 1.162838 | 0.367 | 0.189 | 0.056278 | 23 *PTGDS* | Ocular surface epithelium |
| 6.67E-40 | 1.124607 | 0.367 | 0.046 | 1.47E-35 | 23 *NUPR1* | Ocular surface epithelium |
| 8.95E-13 | 1.111046 | 0.823 | 0.577 | 1.98E-08 | 23 *SAT1* | Ocular surface epithelium |
| 9.73E-14 | 1.084968 | 0.899 | 0.754 | 2.15E-09 | 23 *B2M* | Ocular surface epithelium |
| 4.96E-05 | 1.069066 | 0.722 | 0.727 | 1 | 23 *HSPA5* | Ocular surface epithelium |
| 2.08E-07 | 1.064459 | 0.139 | 0.033 | 0.004584 | 23 *MT1E* | Ocular surface epithelium |
| 1.24E-13 | 1.049483 | 0.266 | 0.064 | 2.74E-09 | 23 *CLDN4* | Ocular surface epithelium |
| 1.51E-10 | 1.008359 | 0.671 | 0.476 | 3.34E-06 | 23 *SDCBP* | Ocular surface epithelium |
| 5.23E-10 | 0.970092 | 0.532 | 0.263 | 1.16E-05 | 23 *HLA-B* | Ocular surface epithelium |
| 1.07E-09 | 0.964104 | 0.228 | 0.064 | 2.37E-05 | 23 *ELF3* | Ocular surface epithelium |
| 2.02E-12 | 0.963759 | 0.671 | 0.398 | 4.47E-08 | 23 *HSPB1* | Ocular surface epithelium |
| 3.10E-13 | 0.959692 | 0.456 | 0.174 | 6.85E-09 | 23 *IER3* | Ocular surface epithelium |
| 8.40E-05 | 0.939435 | 0.215 | 0.096 | 1 | 23 *IGFBP6* | Ocular surface epithelium |
| 3.31E-12 | 0.921592 | 0.759 | 0.541 | 7.31E-08 | 23 *SLC3A2* | Ocular surface epithelium |
| 2.94E-08 | 0.921097 | 0.253 | 0.084 | 0.000648 | 23 *CRYAB* | Ocular surface epithelium |
| 1.27E-20 | 0.865242 | 0.582 | 0.205 | 2.80E-16 | 23 *PHLDA3* | Ocular surface epithelium |
| 1.53E-19 | 0.859547 | 0.987 | 0.961 | 3.37E-15 | 23 *EIF1* | Ocular surface epithelium |
| 8.76E-14 | 0.85247 | 0.278 | 0.07 | 1.93E-09 | 23 *NDUFA4L2* | Ocular surface epithelium |
| 2.54E-17 | 0.839217 | 0.418 | 0.119 | 5.61E-13 | 23 *TPD52L1* | Ocular surface epithelium |
| 2.00E-10 | 0.828013 | 0.203 | 0.048 | 4.43E-06 | 23 *TFPI2* | Ocular surface epithelium |
| 5.09E-13 | 0.825429 | 0.57 | 0.266 | 1.12E-08 | 23 *CDKN1A* | Ocular surface epithelium |
| 6.74E-10 | 0.819733 | 0.759 | 0.606 | 1.49E-05 | 23 *ARF4* | Ocular surface epithelium |
| 1.23E-17 | 0.815894 | 0.342 | 0.083 | 2.71E-13 | 23 *TRIB3* | Ocular surface epithelium |
| 1.94E-32 | 0.804858 | 0.127 | 0.006 | 4.29E-28 | 23 *TM4SF1* | Ocular surface epithelium |
| 1.02E-06 | 0.799532 | 0.544 | 0.397 | 0.022489 | 23 *DDIT3* | Ocular surface epithelium |
| 5.99E-06 | 0.794283 | 0.696 | 0.591 | 0.132299 | 23 *MAP1LC3B* | Ocular surface epithelium |
| 4.41E-11 | 0.782056 | 0.671 | 0.438 | 9.73E-07 | 23 *ANXA5* | Ocular surface epithelium |
| 2.34E-14 | 0.781793 | 0.57 | 0.261 | 5.16E-10 | 23 *PPP1R15A* | Ocular surface epithelium |
| 5.73E-11 | 0.779154 | 0.532 | 0.248 | 1.27E-06 | 23 *PERP* | Ocular surface epithelium |
| 5.05E-11 | 0.775265 | 0.544 | 0.275 | 1.12E-06 | 23 *CAST* | Ocular surface epithelium |
| 0.007511 | 0.753563 | 0.342 | 0.256 | 1 | 23 *LY6E* | Ocular surface epithelium |
| 8.51E-11 | 0.748335 | 0.835 | 0.675 | 1.88E-06 | 23 *TKT* | Ocular surface epithelium |
| 9.12E-07 | 0.747817 | 0.658 | 0.514 | 0.020147 | 23 *MYL12A* | Ocular surface epithelium |
| 0.000833 | 0.742589 | 0.215 | 0.11 | 1 | 23 *S100A4* | Ocular surface epithelium |
| 1.32E-15 | 0.737975 | 0.57 | 0.245 | 2.91E-11 | 23 *LMNA* | Ocular surface epithelium |
| 8.61E-08 | 0.736308 | 0.608 | 0.421 | 0.001901 | 23 *HERPUD1* | Ocular surface epithelium |
| 1.74E-86 | 0.728592 | 0.101 | 0.001 | 3.83E-82 | 23 *AGR2* | Ocular surface epithelium |
| 0.000558 | 0.72514 | 0.456 | 0.299 | 1 | 23 *CD9* | Ocular surface epithelium |
| 1.61E-11 | 0.719455 | 0.38 | 0.135 | 3.55E-07 | 23 *MGST1* | Ocular surface epithelium |
| 7.62E-08 | 0.71707 | 0.734 | 0.636 | 0.001682 | 23 *KRT10* | Ocular surface epithelium |
| 6.05E-06 | 0.714456 | 0.544 | 0.352 | 0.133532 | 23 *CTSD* | Ocular surface epithelium |
| 4.70E-13 | 0.705875 | 0.203 | 0.041 | 1.04E-08 | 23 *GPRC5A* | Ocular surface epithelium |
| 1.31E-07 | -0.70571 | 0 | 0.268 | 0.002884 | 23 *CPLX3* | Ocular surface epithelium |
| 2.14E-06 | -0.71475 | 0.101 | 0.35 | 0.047329 | 23 *SMC4* | Ocular surface epithelium |
| 8.56E-06 | -0.71911 | 0.013 | 0.222 | 0.189081 | 23 *CHODL* | Ocular surface epithelium |
| 5.48E-16 | -0.72728 | 0.835 | 0.997 | 1.21E-11 | 23 *MT-CO3* | Ocular surface epithelium |
| 4.18E-06 | -0.73204 | 0.43 | 0.625 | 0.092201 | 23 *PLEKHA1* | Ocular surface epithelium |
| 2.22E-05 | -0.73241 | 0.291 | 0.49 | 0.49117 | 23 *DKK3* | Ocular surface epithelium |
| 1.51E-05 | -0.73618 | 0 | 0.195 | 0.333845 | 23 *MKI67* | Ocular surface epithelium |
| 5.76E-07 | -0.74108 | 0.051 | 0.303 | 0.012709 | 23 *LINC00599* | Ocular surface epithelium |

| KRT8 |
| --- |
| SQSTM1 |
| MT2A |
| ATF5 |
| TACSTD2 |
| RARRES1 |
| LGALS3.3 |
| CCL2.1 |
| FTH1.1 |
| ATF3 |
| PTGDS.8 |
| NUPR1 |
| SAT1.6 |
| B2M.6 |
| HSPA5 |
| MT1E |
| CLDN4 |
| SDCBP |
| HLA-B.3 |
| ELF3 |
| HSPB1.1 |
| IER3 |
| IGFBP6.1 |
| SLC3A2 |
| CRYAB.2 |
| PHLDA3 |
| EIF1 |
| NDUFA4L2 |
| TPD52L1.2 |
| TFPI2.1 |
| CDKN1A |
| ARF4.1 |
| TRIB3 |
| TM4SF1 |
| DDIT3 |
| MAP1LC3B |
| ANXA5 |
| PPP1R15A |
| PERP |
| CAST |
| LY6E |
| TKT.2 |
| MYL12A.1 |
| S100A4 |
| LMNA |
| HERPUD1 |
| AGR2 |
| CD9.5 |
| MGST1.1 |
| KRT10 |
| CTSD.1 |
| GPRC5A |
| CPLX3.3 |
| SMC4.2 |
| CHODL.3 |
| MT-CO3 |
| PLEKHA1.6 |
| DKK3.11 |
| MKI67.3 |
| LINC00599 |

| CPE.9 | 4.70E-07 | -0.74685 | 0.443 | 0.668 | 0.010368 | 23 | *CPE* | Ocular surface epithelium |
| --- | --- | --- | --- | --- | --- | --- | --- | --- |
| EPB41.2 | 2.47E-08 | -0.75272 | 0.241 | 0.521 | 0.000546 | 23 | *EPB41* | Ocular surface epithelium |
| TYMS.7 | 2.67E-07 | -0.7534 | 0.418 | 0.627 | 0.005897 | 23 | *TYMS* | Ocular surface epithelium |
| UBE2C.14 | 0.000984 | -0.75502 | 0.051 | 0.201 | 1 | 23 | *UBE2C* | Ocular surface epithelium |
| HMGN2.6 | 4.72E-13 | -0.76488 | 0.785 | 0.924 | 1.04E-08 | 23 | *HMGN2* | Ocular surface epithelium |
| CADM1 | 5.21E-12 | -0.77137 | 0.203 | 0.623 | 1.15E-07 | 23 | *CADM1* | Ocular surface epithelium |
| RXRG.13 | 4.21E-07 | -0.77874 | 0.076 | 0.339 | 0.009288 | 23 | *RXRG* | Ocular surface epithelium |
| NT5DC2.2 | 1.08E-08 | -0.78441 | 0.165 | 0.462 | 0.000239 | 23 | *NT5DC2* | Ocular surface epithelium |
| TAGLN3.4 | 4.19E-10 | -0.78773 | 0.038 | 0.391 | 9.25E-06 | 23 | *TAGLN3* | Ocular surface epithelium |
| UBE2T.5 | 1.75E-09 | -0.80145 | 0.165 | 0.485 | 3.86E-05 | 23 | *UBE2T* | Ocular surface epithelium |
| CHD7 | 6.98E-13 | -0.80484 | 0.215 | 0.6 | 1.54E-08 | 23 | *CHD7* | Ocular surface epithelium |
| TPH1.16 | 1.57E-05 | -0.81298 | 0.025 | 0.227 | 0.346203 | 23 | *TPH1* | Ocular surface epithelium |
| UNC119.17 | 7.61E-05 | -0.81319 | 0.354 | 0.523 | 1 | 23 | *UNC119* | Ocular surface epithelium |
| PSIP1 | 6.07E-16 | -0.81465 | 0.633 | 0.874 | 1.34E-11 | 23 | *PSIP1* | Ocular surface epithelium |
| GPM6A.2 | 7.89E-14 | -0.81831 | 0.291 | 0.709 | 1.74E-09 | 23 | *GPM6A* | Ocular surface epithelium |
| CADPS.10 | 1.11E-08 | -0.82245 | 0.038 | 0.343 | 0.000245 | 23 | *CADPS* | Ocular surface epithelium |
| SEPT4.14 | 2.76E-06 | -0.82305 | 0.165 | 0.398 | 0.060909 | 23 | *Sep-04* | Ocular surface epithelium |
| PCLAF.6 | 6.97E-08 | -0.824 | 0.089 | 0.38 | 0.001538 | 23 | *PCLAF* | Ocular surface epithelium |
| HMGB2.13 | 2.74E-07 | -0.8246 | 0.544 | 0.734 | 0.006049 | 23 | *HMGB2* | Ocular surface epithelium |
| TOP2A.16 | 0.000167 | -0.83827 | 0.089 | 0.281 | 1 | 23 | *TOP2A* | Ocular surface epithelium |
| MAP2.7 | 2.14E-09 | -0.84576 | 0.215 | 0.543 | 4.71E-05 | 23 | *MAP2* | Ocular surface epithelium |
| TMSB15A. | 5.67E-14 | -0.85187 | 0.241 | 0.645 | 1.25E-09 | 23 | *TMSB15A* | Ocular surface epithelium |
| PCBP4.11 | 1.07E-07 | -0.85357 | 0.241 | 0.52 | 0.002356 | 23 | *PCBP4* | Ocular surface epithelium |
| TUBB.7 | 1.72E-14 | -0.8622 | 0.886 | 0.949 | 3.79E-10 | 23 | *TUBB* | Ocular surface epithelium |
| RORB.3 | 1.28E-10 | -0.86601 | 0.253 | 0.592 | 2.83E-06 | 23 | *RORB* | Ocular surface epithelium |
| FAM57B.1 | 5.58E-08 | -0.87416 | 0.177 | 0.465 | 0.001233 | 23 | *FAM57B* | Ocular surface epithelium |
| SOX4.9 | 7.26E-08 | -0.87875 | 0.696 | 0.867 | 0.001602 | 23 | *SOX4* | Ocular surface epithelium |
| CRX.14 | 4.54E-08 | -0.87878 | 0.038 | 0.329 | 0.001003 | 23 | *CRX* | Ocular surface epithelium |
| DIO3.15 | 1.45E-06 | -0.88215 | 0.063 | 0.312 | 0.032028 | 23 | *DIO3* | Ocular surface epithelium |
| SLC38A5.1 | 2.11E-06 | -0.88274 | 0.089 | 0.33 | 0.04654 | 23 | *SLC38A5* | Ocular surface epithelium |
| TUBB2B.6 | 5.25E-12 | -0.88925 | 0.544 | 0.837 | 1.16E-07 | 23 | *TUBB2B* | Ocular surface epithelium |
| SYP.12 | 2.69E-10 | -0.8969 | 0.038 | 0.391 | 5.94E-06 | 23 | *SYP* | Ocular surface epithelium |
| TUBB4B.8 | 9.66E-12 | -0.90493 | 0.62 | 0.832 | 2.13E-07 | 23 | *TUBB4B* | Ocular surface epithelium |
| ATP1A3.11 | 1.84E-09 | -0.9126 | 0.025 | 0.351 | 4.07E-05 | 23 | *ATP1A3* | Ocular surface epithelium |
| OTX2.10 | 7.25E-09 | -0.92229 | 0.051 | 0.36 | 0.00016 | 23 | *OTX2* | Ocular surface epithelium |
| NCAM1.1 | 2.11E-16 | -0.9278 | 0.215 | 0.669 | 4.66E-12 | 23 | *NCAM1* | Ocular surface epithelium |
| NNAT.3 | 2.16E-14 | -0.92975 | 0.165 | 0.615 | 4.76E-10 | 23 | *NNAT* | Ocular surface epithelium |
| DCT.15 | 7.02E-06 | -0.95519 | 0.063 | 0.294 | 0.15502 | 23 | *DCT* | Ocular surface epithelium |
| DEK.8 | 4.19E-16 | -1.00315 | 0.595 | 0.868 | 9.25E-12 | 23 | *DEK* | Ocular surface epithelium |
| GNB3.16 | 1.73E-06 | -1.01269 | 0.177 | 0.423 | 0.038137 | 23 | *GNB3* | Ocular surface epithelium |
| CENPF.16 | 0.000107 | -1.01829 | 0.114 | 0.304 | 1 | 23 | *CENPF* | Ocular surface epithelium |
| STMN1.7 | 4.32E-23 | -1.06995 | 0.835 | 0.965 | 9.55E-19 | 23 | *STMN1* | Ocular surface epithelium |
| CRABP1.13 | 1.22E-09 | -1.07016 | 0.519 | 0.743 | 2.68E-05 | 23 | *CRABP1* | Ocular surface epithelium |
| AMER2.12 | 4.63E-12 | -1.07025 | 0.101 | 0.501 | 1.02E-07 | 23 | *AMER2* | Ocular surface epithelium |
| AIPL1.18 | 2.98E-06 | -1.09604 | 0.101 | 0.334 | 0.065738 | 23 | *AIPL1* | Ocular surface epithelium |
| SFRP2.17 | 0.004754 | -1.09923 | 0.494 | 0.547 | 1 | 23 | *SFRP2* | Ocular surface epithelium |
| BASP1.6 | 1.02E-14 | -1.14546 | 0.367 | 0.728 | 2.25E-10 | 23 | *BASP1* | Ocular surface epithelium |
| STMN2.14 | 4.87E-07 | -1.16378 | 0.127 | 0.394 | 0.010756 | 23 | *STMN2* | Ocular surface epithelium |
| PDE6H.17 | 1.50E-05 | -1.2027 | 0.165 | 0.382 | 0.331535 | 23 | *PDE6H* | Ocular surface epithelium |
| NEUROD1. | 1.58E-11 | -1.21483 | 0.051 | 0.438 | 3.48E-07 | 23 | *NEUROD1* | Ocular surface epithelium |
| NRL.16 | 2.28E-05 | -1.2511 | 0.127 | 0.345 | 0.502715 | 23 | *NRL* | Ocular surface epithelium |
| TRH.13 | 1.65E-08 | -1.25874 | 0.291 | 0.575 | 0.000365 | 23 | *TRH* | Ocular surface epithelium |
| RCVRN.17 | 1.17E-05 | -1.50218 | 0.165 | 0.38 | 0.258233 | 23 | *RCVRN* | Ocular surface epithelium |
| SCG3.14 | 3.24E-14 | -1.54025 | 0.101 | 0.534 | 7.15E-10 | 23 | *SCG3* | Ocular surface epithelium |
| CKB.5 | 6.41E-32 | -1.57877 | 0.81 | 0.973 | 1.42E-27 | 23 | *CKB* | Ocular surface epithelium |
| HES6.8 | 3.90E-15 | -1.64159 | 0.278 | 0.666 | 8.61E-11 | 23 | *HES6* | Ocular surface epithelium |
| PDC.19 | 4.52E-09 | -1.68744 | 0.114 | 0.428 | 9.99E-05 | 23 | *PDC* | Ocular surface epithelium |
| MGP.1 | 1.10E-125 | 4.757926 | 0.84 | 0.086 | 2.43E-121 | 24 | *MGP* | Fibroblasts |
| COL1A1.1 | 3.22E-140 | 4.415942 | 0.853 | 0.078 | 7.11E-136 | 24 | *COL1A1* | Fibroblasts |
| COL3A1 | 0 | 3.557368 | 0.907 | 0.032 | 0 | 24 | *COL3A1* | Fibroblasts |

| COL1A2.11 | 3.75E-53 | 3.403513 | 0.947 | 0.364 | 8.27E-49 | 24 | *COL1A2* | Fibroblasts |
| --- | --- | --- | --- | --- | --- | --- | --- | --- |
| LUM | 2.05E-275 | 3.109435 | 0.533 | 0.009 | 4.52E-271 | 24 | *LUM* | Fibroblasts |
| LGALS1 | 6.69E-195 | 2.994363 | 0.947 | 0.067 | 1.48E-190 | 24 | *LGALS1* | Fibroblasts |
| DCN | 0 | 2.511575 | 0.68 | 0.006 | 0 | 24 | *DCN* | Fibroblasts |
| FN1 | 1.41E-94 | 2.361386 | 0.68 | 0.072 | 3.12E-90 | 24 | *FN1* | Fibroblasts |
| C7 | 3.75E-21 | 2.272337 | 0.227 | 0.032 | 8.28E-17 | 24 | *C7* | Fibroblasts |
| S100A11.2 | 2.36E-97 | 2.088928 | 0.8 | 0.098 | 5.22E-93 | 24 | *S100A11* | Fibroblasts |
| SPARC.2 | 1.63E-34 | 2.000676 | 0.92 | 0.485 | 3.61E-30 | 24 | *SPARC* | Fibroblasts |
| COL5A2 | 4.71E-93 | 1.951057 | 0.627 | 0.059 | 1.04E-88 | 24 | *COL5A2* | Fibroblasts |
| TAGLN | 2.24E-48 | 1.879591 | 0.48 | 0.065 | 4.94E-44 | 24 | *TAGLN* | Fibroblasts |
| PLAT | 1.18E-26 | 1.821418 | 0.493 | 0.127 | 2.60E-22 | 24 | *PLAT* | Fibroblasts |
| CXCL14.1 | 3.13E-08 | 1.801997 | 0.147 | 0.033 | 0.000692 | 24 | *CXCL14* | Fibroblasts |
| TGFBI | 4.40E-198 | 1.791171 | 0.547 | 0.017 | 9.71E-194 | 24 | *TGFBI* | Fibroblasts |
| MEG3 | 7.29E-105 | 1.67948 | 0.36 | 0.015 | 1.61E-100 | 24 | *MEG3* | Fibroblasts |
| S100A10.3 | 7.32E-50 | 1.646481 | 0.52 | 0.073 | 1.62E-45 | 24 | *S100A10* | Fibroblasts |
| ANXA2.3 | 5.54E-36 | 1.639872 | 0.827 | 0.317 | 1.22E-31 | 24 | *ANXA2* | Fibroblasts |
| BGN | 6.33E-245 | 1.625905 | 0.533 | 0.011 | 1.40E-240 | 24 | *BGN* | Fibroblasts |
| COL6A2 | 6.38E-27 | 1.61106 | 0.76 | 0.35 | 1.41E-22 | 24 | *COL6A2* | Fibroblasts |
| LXN | 3.80E-33 | 1.600752 | 0.453 | 0.084 | 8.39E-29 | 24 | *LXN* | Fibroblasts |
| IGFBP5.9 | 5.40E-14 | 1.525584 | 0.707 | 0.428 | 1.19E-09 | 24 | *IGFBP5* | Fibroblasts |
| GSN.1 | 8.27E-13 | 1.521836 | 0.573 | 0.285 | 1.83E-08 | 24 | *GSN* | Fibroblasts |
| IGFBP4.1 | 1.51E-21 | 1.503562 | 0.56 | 0.197 | 3.34E-17 | 24 | *IGFBP4* | Fibroblasts |
| TPM2 | 2.34E-24 | 1.48852 | 0.573 | 0.188 | 5.17E-20 | 24 | *TPM2* | Fibroblasts |
| TIMP1.2 | 1.19E-11 | 1.461186 | 0.653 | 0.49 | 2.63E-07 | 24 | *TIMP1* | Fibroblasts |
| PCOLCE | 8.46E-23 | 1.452343 | 0.507 | 0.148 | 1.87E-18 | 24 | *PCOLCE* | Fibroblasts |
| POSTN.1 | 2.42E-92 | 1.446384 | 0.347 | 0.015 | 5.34E-88 | 24 | *POSTN* | Fibroblasts |
| ITM2A.1 | 1.71E-52 | 1.432657 | 0.52 | 0.07 | 3.78E-48 | 24 | *ITM2A* | Fibroblasts |
| APOE.7 | 0.004926 | 1.426302 | 0.493 | 0.45 | 1 | 24 | *APOE* | Fibroblasts |
| COL5A1 | 6.11E-15 | 1.403338 | 0.467 | 0.164 | 1.35E-10 | 24 | *COL5A1* | Fibroblasts |
| SERPINH1 | 1.11E-39 | 1.359119 | 0.653 | 0.159 | 2.46E-35 | 24 | *SERPINH1* | Fibroblasts |
| MFAP4 | 1.11E-26 | 1.348162 | 0.6 | 0.18 | 2.45E-22 | 24 | *MFAP4* | Fibroblasts |
| A2M.1 | 3.09E-74 | 1.338983 | 0.467 | 0.039 | 6.83E-70 | 24 | *A2M* | Fibroblasts |
| ZFP36 | 1.09E-12 | 1.318035 | 0.507 | 0.214 | 2.41E-08 | 24 | *ZFP36* | Fibroblasts |
| CTGF | 1.61E-20 | 1.304141 | 0.2 | 0.025 | 3.56E-16 | 24 | *CTGF* | Fibroblasts |
| RGS5.1 | 1.01E-12 | 1.301071 | 0.307 | 0.087 | 2.23E-08 | 24 | *RGS5* | Fibroblasts |
| CEBPD.1 | 5.17E-16 | 1.298676 | 0.453 | 0.149 | 1.14E-11 | 24 | *CEBPD* | Fibroblasts |
| VCAN.1 | 1.50E-24 | 1.29582 | 0.533 | 0.151 | 3.31E-20 | 24 | *VCAN* | Fibroblasts |
| ACTA2 | 3.11E-10 | 1.293648 | 0.307 | 0.098 | 6.87E-06 | 24 | *ACTA2* | Fibroblasts |
| IGFBP7.4 | 6.26E-18 | 1.290669 | 0.547 | 0.201 | 1.38E-13 | 24 | *IGFBP7* | Fibroblasts |
| PLAC9 | 4.21E-125 | 1.285886 | 0.32 | 0.009 | 9.30E-121 | 24 | *PLAC9* | Fibroblasts |
| IGFBP6.2 | 4.89E-37 | 1.273259 | 0.507 | 0.092 | 1.08E-32 | 24 | *IGFBP6* | Fibroblasts |
| OGN | 8.43E-169 | 1.244155 | 0.253 | 0.003 | 1.86E-164 | 24 | *OGN* | Fibroblasts |
| SERPINE2.1 | 6.81E-05 | 1.240987 | 0.373 | 0.243 | 1 | 24 | *SERPINE2* | Fibroblasts |
| SFRP4.1 | 2.40E-07 | 1.239817 | 0.12 | 0.025 | 0.005307 | 24 | *SFRP4* | Fibroblasts |
| GDF15.1 | 3.24E-11 | 1.233881 | 0.213 | 0.048 | 7.16E-07 | 24 | *GDF15* | Fibroblasts |
| KERA | 3.91E-120 | 1.219016 | 0.133 | 0.001 | 8.63E-116 | 24 | *KERA* | Fibroblasts |
| COL14A1 | 7.40E-245 | 1.217775 | 0.387 | 0.005 | 1.63E-240 | 24 | *COL14A1* | Fibroblasts |
| FOS.11 | 9.60E-17 | 1.184076 | 0.827 | 0.554 | 2.12E-12 | 24 | *FOS* | Fibroblasts |
| FCGRT | 5.32E-66 | 1.172892 | 0.56 | 0.066 | 1.18E-61 | 24 | *FCGRT* | Fibroblasts |
| COL6A1 | 5.37E-13 | 1.172492 | 0.6 | 0.298 | 1.19E-08 | 24 | *COL6A1* | Fibroblasts |
| FOSB | 8.03E-25 | 1.161309 | 0.507 | 0.123 | 1.77E-20 | 24 | *FOSB* | Fibroblasts |
| IFITM3.15 | 4.08E-20 | 1.131507 | 0.747 | 0.349 | 9.00E-16 | 24 | *IFITM3* | Fibroblasts |
| LMNA.1 | 1.05E-25 | 1.130816 | 0.667 | 0.244 | 2.31E-21 | 24 | *LMNA* | Fibroblasts |
| EMILIN1 | 2.02E-95 | 1.128298 | 0.413 | 0.023 | 4.47E-91 | 24 | *EMILIN1* | Fibroblasts |
| COL12A1 | 2.66E-237 | 1.124901 | 0.373 | 0.004 | 5.88E-233 | 24 | *COL12A1* | Fibroblasts |
| XIST | 1.16E-119 | 1.124232 | 0.267 | 0.006 | 2.56E-115 | 24 | *XIST* | Fibroblasts |
| RARRES1.1 | 1.21E-19 | 1.11031 | 0.36 | 0.079 | 2.66E-15 | 24 | *RARRES1* | Fibroblasts |
| ID3.6 | 3.46E-14 | 1.102941 | 0.76 | 0.46 | 7.64E-10 | 24 | *ID3* | Fibroblasts |
| JUNB.4 | 3.97E-07 | 1.093438 | 0.667 | 0.579 | 0.008769 | 24 | *JUNB* | Fibroblasts |
| MYL9 | 2.57E-26 | 1.092616 | 0.36 | 0.06 | 5.68E-22 | 24 | *MYL9* | Fibroblasts |

CD99.1 IER3.1 NUPR1.1 COL16A1

| 2.94E-21 | 1.087604 | 0.76 | 0.39 | 6.48E-17 | 24 | *CD99* | Fibroblasts |
| --- | --- | --- | --- | --- | --- | --- | --- |
| 2.09E-13 | 1.079752 | 0.453 | 0.175 | 4.60E-09 | 24 | *IER3* | Fibroblasts |
| 2.55E-60 | 1.073487 | 0.453 | 0.045 | 5.63E-56 | 24 | *NUPR1* | Fibroblasts |
| 2.70E-44 | 1.059872 | 0.4 | 0.048 | 5.97E-40 | 24 | *COL16A1* | Fibroblasts |
| 1.16E-11 | 1.041848 | 0.267 | 0.074 | 2.56E-07 | 24 | *C2orf40* | Fibroblasts |
| 2.87E-08 | 1.038976 | 0.387 | 0.169 | 0.000633 | 24 | *GADD45B* | Fibroblasts |
| 8.11E-175 | 1.030437 | 0.333 | 0.006 | 1.79E-170 | 24 | *COL6A3* | Fibroblasts |
| 8.56E-14 | 1.029114 | 0.173 | 0.028 | 1.89E-09 | 24 | *GAL* | Fibroblasts |
| 1.04E-10 | 1.024122 | 0.427 | 0.181 | 2.30E-06 | 24 | *CTSC* | Fibroblasts |
| 2.04E-06 | 1.020276 | 0.52 | 0.324 | 0.045073 | 24 | *DUSP1* | Fibroblasts |
| 1.69E-30 | 0.990204 | 0.333 | 0.046 | 3.74E-26 | 24 | *ANXA1* | Fibroblasts |
| 1.79E-14 | 0.985058 | 0.693 | 0.398 | 3.96E-10 | 24 | *HSPB1* | Fibroblasts |
| 1.67E-11 | 0.975062 | 0.8 | 0.756 | 3.68E-07 | 24 | *B2M* | Fibroblasts |
| 6.36E-18 | 0.970017 | 0.6 | 0.253 | 1.40E-13 | 24 | *LY6E* | Fibroblasts |
| 0.00624 | 0.959232 | 0.333 | 0.223 | 1 | 24 | *MT2A* | Fibroblasts |
| 5.52E-34 | 0.958416 | 0.387 | 0.057 | 1.22E-29 | 24 | *ELN* | Fibroblasts |
| 6.86E-05 | 0.941331 | 0.507 | 0.406 | 1 | 24 | *AKAP12* | Fibroblasts |
| 1.80E-16 | 0.941201 | 0.987 | 0.799 | 3.98E-12 | 24 | *VIM* | Fibroblasts |
| 7.33E-12 | 0.941068 | 0.64 | 0.438 | 1.62E-07 | 24 | *ANXA5* | Fibroblasts |
| 2.79E-12 | 0.921282 | 0.347 | 0.11 | 6.15E-08 | 24 | *CTHRC1* | Fibroblasts |
| 2.83E-06 | 0.919784 | 0.72 | 0.638 | 0.062443 | 24 | *JUN* | Fibroblasts |
| 8.38E-139 | 0.915771 | 0.387 | 0.012 | 1.85E-134 | 24 | *THBS2* | Fibroblasts |
| 9.95E-30 | 0.908282 | 0.453 | 0.086 | 2.20E-25 | 24 | *MMP2* | Fibroblasts |
| 2.58E-15 | 0.907673 | 0.56 | 0.235 | 5.69E-11 | 24 | *FKBP10* | Fibroblasts |
| 9.87E-19 | 0.904389 | 0.827 | 0.685 | 2.18E-14 | 24 | *LAPTM4A* | Fibroblasts |
| 3.87E-92 | 0.90287 | 0.4 | 0.021 | 8.54E-88 | 24 | *LAMA4* | Fibroblasts |
| 1.10E-59 | 0.895105 | 0.347 | 0.026 | 2.43E-55 | 24 | *CAVIN1* | Fibroblasts |
| 7.06E-42 | 0.8948 | 0.293 | 0.027 | 1.56E-37 | 24 | *GLT8D2* | Fibroblasts |
| 4.03E-14 | 0.882115 | 0.387 | 0.122 | 8.89E-10 | 24 | *MRC2* | Fibroblasts |
| 2.39E-50 | 0.878549 | 0.28 | 0.02 | 5.29E-46 | 24 | *OLFML3* | Fibroblasts |
| 1.61E-22 | 0.853854 | 0.44 | 0.105 | 3.56E-18 | 24 | *BST2* | Fibroblasts |
| 3.97E-09 | 0.851147 | 0.533 | 0.279 | 8.76E-05 | 24 | *ID1* | Fibroblasts |
| 1.55E-09 | 0.842732 | 0.347 | 0.126 | 3.43E-05 | 24 | *PMP22* | Fibroblasts |
| 1.32E-10 | 0.841002 | 0.547 | 0.275 | 2.92E-06 | 24 | *MFAP2* | Fibroblasts |
| 2.64E-23 | 0.839178 | 0.133 | 0.01 | 5.84E-19 | 24 | *EGFL6* | Fibroblasts |
| 7.48E-43 | 0.835073 | 0.36 | 0.039 | 1.65E-38 | 24 | *PDGFRA* | Fibroblasts |
| 3.51E-11 | 0.832708 | 0.467 | 0.177 | 7.75E-07 | 24 | *GNG11* | Fibroblasts |
| 8.39E-08 | 0.83173 | 0.64 | 0.508 | 0.001853 | 24 | *CALD1* | Fibroblasts |
| 1.69E-05 | 0.825422 | 0.413 | 0.224 | 0.372091 | 24 | *HSPA1A* | Fibroblasts |
| 1.41E-19 | 0.823132 | 0.48 | 0.142 | 3.11E-15 | 24 | *NFIA* | Fibroblasts |
| 3.23E-70 | 0.815815 | 0.133 | 0.002 | 7.14E-66 | 24 | *SUCNR1* | Fibroblasts |
| 2.17E-19 | 0.814448 | 0.48 | 0.147 | 4.79E-15 | 24 | *PLS3* | Fibroblasts |
| 3.37E-11 | 0.809924 | 0.467 | 0.199 | 7.45E-07 | 24 | *EVA1B* | Fibroblasts |
| 1.11E-12 | 0.809338 | 0.227 | 0.048 | 2.44E-08 | 24 | *TFPI2* | Fibroblasts |
| 1.69E-10 | 0.809137 | 0.493 | 0.221 | 3.73E-06 | 24 | *TIMP3* | Fibroblasts |
| 1.78E-21 | 0.794621 | 0.36 | 0.074 | 3.94E-17 | 24 | *NR4A1* | Fibroblasts |
| 3.40E-21 | 0.784909 | 1 | 0.99 | 7.51E-17 | 24 | *RPL10* | Fibroblasts |
| 6.40E-12 | 0.784518 | 0.813 | 0.73 | 1.41E-07 | 24 | *EEF1D* | Fibroblasts |
| 1.18E-56 | 0.773624 | 0.293 | 0.019 | 2.60E-52 | 24 | *FOXC1* | Fibroblasts |
| 0.000178 | 0.765752 | 0.627 | 0.524 | 1 | 24 | *IGFBP2* | Fibroblasts |
| 1.63E-14 | 0.765347 | 0.147 | 0.02 | 3.61E-10 | 24 | *TGM2* | Fibroblasts |
| 3.07E-12 | 0.764703 | 0.173 | 0.031 | 6.78E-08 | 24 | *RGCC* | Fibroblasts |
| 1.16E-45 | 0.763203 | 0.373 | 0.04 | 2.56E-41 | 24 | *TWIST1* | Fibroblasts |
| 2.98E-138 | 0.758 | 0.187 | 0.002 | 6.57E-134 | 24 | *PITX2* | Fibroblasts |
| 1.08E-07 | 0.754973 | 0.667 | 0.514 | 0.00239 | 24 | *MYL12A* | Fibroblasts |
| 1.06E-71 | 0.751066 | 0.147 | 0.003 | 2.34E-67 | 24 | *CD34* | Fibroblasts |
| 2.63E-24 | 0.742453 | 0.36 | 0.067 | 5.80E-20 | 24 | *C1R* | Fibroblasts |
| 0.003617 | 0.734087 | 0.547 | 0.447 | 1 | 24 | *FBLN1* | Fibroblasts |
| 7.81E-27 | 0.733149 | 0.28 | 0.037 | 1.72E-22 | 24 | *ARHGDIB* | Fibroblasts |
| 2.56E-14 | 0.732698 | 0.88 | 0.86 | 5.65E-10 | 24 | *CD63* | Fibroblasts |

C2orf40 GADD45B COL6A3 GAL

CTSC DUSP1 ANXA1.1 HSPB1.2 B2M.7 LY6E.1 MT2A.1 ELN.1 AKAP12.4 VIM.14 ANXA5.1 CTHRC1 JUN.1 THBS2 MMP2 FKBP10 LAPTM4A LAMA4 CAVIN1 GLT8D2 MRC2 OLFML3 BST2.1 ID1.1 PMP22 MFAP2.1 EGFL6 PDGFRA GNG11.2 CALD1.1 HSPA1A.2 NFIA.1 SUCNR1 PLS3 EVA1B.1 TFPI2.2 TIMP3.1 NR4A1 RPL10 EEF1D FOXC1 IGFBP2.8 TGM2 RGCC TWIST1 PITX2 MYL12A.2 CD34

C1R FBLN1.1 ARHGDIB CD63

| SEMA3C.1 | 4.05E-11 | 0.727658 | 0.2 | 0.044 | 8.95E-07 | 24 | *SEMA3C* | Fibroblasts |
| --- | --- | --- | --- | --- | --- | --- | --- | --- |
| RRBP1 | 5.39E-11 | 0.725494 | 0.467 | 0.207 | 1.19E-06 | 24 | *RRBP1* | Fibroblasts |
| SERPING1 | 2.64E-08 | 0.715053 | 0.387 | 0.166 | 0.000583 | 24 | *SERPING1* | Fibroblasts |
| SPATS2L | 1.23E-43 | 0.713409 | 0.453 | 0.061 | 2.71E-39 | 24 | *SPATS2L* | Fibroblasts |
| COL4A1 | 3.14E-10 | 0.711801 | 0.32 | 0.104 | 6.94E-06 | 24 | *COL4A1* | Fibroblasts |
| PRRX1 | 5.64E-181 | 0.709796 | 0.387 | 0.008 | 1.25E-176 | 24 | *PRRX1* | Fibroblasts |
| CAVIN3 | 1.82E-30 | 0.708193 | 0.227 | 0.022 | 4.01E-26 | 24 | *CAVIN3* | Fibroblasts |
| PDGFRL | 1.27E-74 | 0.708166 | 0.213 | 0.007 | 2.79E-70 | 24 | *PDGFRL* | Fibroblasts |
| EDN3 | 1.13E-71 | 0.708091 | 0.173 | 0.004 | 2.49E-67 | 24 | *EDN3* | Fibroblasts |
| KLF2 | 1.22E-26 | 0.702507 | 0.147 | 0.011 | 2.70E-22 | 24 | *KLF2* | Fibroblasts |
| CTSK | 3.10E-50 | 0.70006 | 0.253 | 0.016 | 6.85E-46 | 24 | *CTSK* | Fibroblasts |
| TTYH1.12 | 1.29E-07 | -0.70119 | 0.147 | 0.458 | 0.002837 | 24 | *TTYH1* | Fibroblasts |
| MT-ND6 | 2.30E-11 | -0.70375 | 0.32 | 0.654 | 5.08E-07 | 24 | *MT-ND6* | Fibroblasts |
| ROM1.6 | 0.001684 | -0.7083 | 0.08 | 0.221 | 1 | 24 | *ROM1* | Fibroblasts |
| GADD45G. | 4.98E-08 | -0.70992 | 0.16 | 0.471 | 0.001101 | 24 | *GADD45G* | Fibroblasts |
| DAAM1 | 1.14E-11 | -0.7115 | 0.24 | 0.637 | 2.52E-07 | 24 | *DAAM1* | Fibroblasts |
| SOX4.10 | 2.84E-07 | -0.71395 | 0.6 | 0.868 | 0.006273 | 24 | *SOX4* | Fibroblasts |
| PTPRZ1.3 | 2.18E-07 | -0.71574 | 0.04 | 0.319 | 0.00482 | 24 | *PTPRZ1* | Fibroblasts |
| NEUROD4. | 7.69E-08 | -0.71615 | 0 | 0.287 | 0.001699 | 24 | *NEUROD4* | Fibroblasts |
| OTX2.11 | 1.40E-06 | -0.71996 | 0.093 | 0.359 | 0.030937 | 24 | *OTX2* | Fibroblasts |
| NEAT1.6 | 5.90E-05 | -0.72003 | 0.573 | 0.777 | 1 | 24 | *NEAT1* | Fibroblasts |
| SLC38A1.3 | 3.51E-08 | -0.72626 | 0.187 | 0.494 | 0.000776 | 24 | *SLC38A1* | Fibroblasts |
| TMEM97.1 | 1.89E-10 | -0.72858 | 0.107 | 0.476 | 4.18E-06 | 24 | *TMEM97* | Fibroblasts |
| OLFM1.5 | 1.21E-07 | -0.73538 | 0.053 | 0.338 | 0.002666 | 24 | *OLFM1* | Fibroblasts |
| HMGN2.7 | 7.43E-12 | -0.73626 | 0.747 | 0.925 | 1.64E-07 | 24 | *HMGN2* | Fibroblasts |
| CRMP1.6 | 2.86E-08 | -0.73845 | 0.133 | 0.431 | 0.000632 | 24 | *CRMP1* | Fibroblasts |
| SOX2.13 | 2.55E-06 | -0.75379 | 0.173 | 0.412 | 0.056225 | 24 | *SOX2* | Fibroblasts |
| RXRG.14 | 9.06E-08 | -0.75557 | 0.04 | 0.339 | 0.002001 | 24 | *RXRG* | Fibroblasts |
| WFDC2 | 6.43E-10 | -0.75703 | 0.053 | 0.415 | 1.42E-05 | 24 | *WFDC2* | Fibroblasts |
| ATP1B1.2 | 6.62E-10 | -0.76204 | 0.36 | 0.71 | 1.46E-05 | 24 | *ATP1B1* | Fibroblasts |
| SSX2IP.1 | 1.35E-11 | -0.76594 | 0.147 | 0.533 | 2.99E-07 | 24 | *SSX2IP* | Fibroblasts |
| RTN3 | 8.74E-15 | -0.76652 | 0.427 | 0.768 | 1.93E-10 | 24 | *RTN3* | Fibroblasts |
| LINC00599 | 7.01E-08 | -0.76872 | 0.013 | 0.303 | 0.001548 | 24 | *LINC00599* | Fibroblasts |
| TPD52 | 2.06E-10 | -0.77084 | 0.067 | 0.429 | 4.54E-06 | 24 | *TPD52* | Fibroblasts |
| TUBA4A.9 | 1.35E-06 | -0.77613 | 0.133 | 0.388 | 0.029825 | 24 | *TUBA4A* | Fibroblasts |
| CLU.15 | 5.95E-08 | -0.77691 | 0.36 | 0.676 | 0.001314 | 24 | *CLU* | Fibroblasts |
| CENPV.1 | 1.11E-13 | -0.78683 | 0.347 | 0.721 | 2.45E-09 | 24 | *CENPV* | Fibroblasts |
| ARL4C | 3.45E-10 | -0.79169 | 0.187 | 0.534 | 7.62E-06 | 24 | *ARL4C* | Fibroblasts |
| CRYM.7 | 3.82E-06 | -0.79219 | 0.067 | 0.307 | 0.084385 | 24 | *CRYM* | Fibroblasts |
| CADPS.11 | 1.75E-08 | -0.79237 | 0.027 | 0.343 | 0.000385 | 24 | *CADPS* | Fibroblasts |
| FZD3 | 9.38E-13 | -0.79448 | 0.093 | 0.523 | 2.07E-08 | 24 | *FZD3* | Fibroblasts |
| BAZ2B | 6.53E-13 | -0.80189 | 0.2 | 0.599 | 1.44E-08 | 24 | *BAZ2B* | Fibroblasts |
| BASP1.7 | 2.72E-10 | -0.80853 | 0.387 | 0.728 | 6.02E-06 | 24 | *BASP1* | Fibroblasts |
| PSIP1.1 | 2.62E-17 | -0.81135 | 0.533 | 0.876 | 5.79E-13 | 24 | *PSIP1* | Fibroblasts |
| H2AFY.2 | 3.98E-14 | -0.82248 | 0.493 | 0.815 | 8.79E-10 | 24 | *H2AFY* | Fibroblasts |
| NASP | 4.55E-15 | -0.82325 | 0.44 | 0.796 | 1.00E-10 | 24 | *NASP* | Fibroblasts |
| PCBP4.12 | 2.83E-08 | -0.82341 | 0.2 | 0.52 | 0.000624 | 24 | *PCBP4* | Fibroblasts |
| EPB41.3 | 4.43E-10 | -0.83507 | 0.173 | 0.522 | 9.78E-06 | 24 | *EPB41* | Fibroblasts |
| NNAT.4 | 3.03E-11 | -0.84144 | 0.187 | 0.614 | 6.70E-07 | 24 | *NNAT* | Fibroblasts |
| LAMP5.14 | 0.000359 | -0.84667 | 0.16 | 0.326 | 1 | 24 | *LAMP5* | Fibroblasts |
| SMC3 | 2.30E-14 | -0.84913 | 0.4 | 0.765 | 5.09E-10 | 24 | *SMC3* | Fibroblasts |
| GADD45A. | 1.81E-06 | -0.85644 | 0.267 | 0.531 | 0.039911 | 24 | *GADD45A* | Fibroblasts |
| STMN2.15 | 1.60E-05 | -0.85972 | 0.147 | 0.394 | 0.354338 | 24 | *STMN2* | Fibroblasts |
| AKAP9.13 | 1.59E-08 | -0.86032 | 0.387 | 0.661 | 0.000351 | 24 | *AKAP9* | Fibroblasts |
| PMAIP1.6 | 1.64E-07 | -0.86271 | 0.147 | 0.43 | 0.003618 | 24 | *PMAIP1* | Fibroblasts |
| AGL.2 | 1.44E-07 | -0.86342 | 0.093 | 0.381 | 0.003179 | 24 | *AGL* | Fibroblasts |
| MLLT11.4 | 6.20E-12 | -0.86623 | 0.347 | 0.694 | 1.37E-07 | 24 | *MLLT11* | Fibroblasts |
| SEPT4.15 | 5.97E-09 | -0.86634 | 0.053 | 0.399 | 0.000132 | 24 | *Sep-04* | Fibroblasts |
| RAX | 4.61E-13 | -0.87489 | 0.04 | 0.483 | 1.02E-08 | 24 | *RAX* | Fibroblasts |
| MIAT.5 | 9.03E-09 | -0.87492 | 0.08 | 0.408 | 0.000199 | 24 | *MIAT* | Fibroblasts |

CHD7.1 MAP1B.3 SLC38A5.1 SYP.13 CRX.15 TMSB15A. RRAD.15 TXNIP HMGA1.10 MAP2.8 DEK.9 BEX1.1 PHYHIPL SOX11.8 DCT.16 RGS16.3 SIX3.1 RTN4.5 DAPL1.14 DIO3.16 PLEKHA1.7 GPM6A.3 FAM57B.1 CD24.5 ENO2.10 GNB3.17 SYT1.2 UNC119.18 AMER2.13 RORB.4 PDE6H.18 SIX6.2 PTH2.5 MAB21L1. AIPL1.19 TUBB2B.7 RCVRN.18 NEUROD1. NRL.17 SPP1.16 SCG3.15 PDC.20 HES6.9 FABP7.8 SFRP2.18 CKB.6 CRABP1.14 TRH.14 CHGB.1 CHODL.4 SLC38A5.1 CSRP2 MAP2K6 HMGN2.8 KIF22 TYMS.8 PCNA.4 PMAIP1.7 FABP7.9 PCLAF.7

| 2.90E-16 | -0.88207 | 0.08 | 0.602 | 6.40E-12 | 24 | *CHD7* | Fibroblasts |
| --- | --- | --- | --- | --- | --- | --- | --- |
| 4.34E-14 | -0.8832 | 0.587 | 0.858 | 9.58E-10 | 24 | *MAP1B* | Fibroblasts |
| 6.56E-07 | -0.90252 | 0.067 | 0.33 | 0.014488 | 24 | *SLC38A5* | Fibroblasts |
| 1.75E-09 | -0.90467 | 0.053 | 0.39 | 3.87E-05 | 24 | *SYP* | Fibroblasts |
| 8.27E-08 | -0.91184 | 0.04 | 0.329 | 0.001825 | 24 | *CRX* | Fibroblasts |
| 2.06E-14 | -0.91533 | 0.173 | 0.646 | 4.54E-10 | 24 | *TMSB15A* | Fibroblasts |
| 8.04E-05 | -0.93574 | 0.12 | 0.321 | 1 | 24 | *RRAD* | Fibroblasts |
| 5.21E-16 | -0.93637 | 0.227 | 0.736 | 1.15E-11 | 24 | *TXNIP* | Fibroblasts |
| 1.16E-13 | -0.94178 | 0.227 | 0.662 | 2.56E-09 | 24 | *HMGA1* | Fibroblasts |
| 1.83E-11 | -0.94359 | 0.147 | 0.543 | 4.05E-07 | 24 | *MAP2* | Fibroblasts |
| 2.16E-15 | -0.95847 | 0.573 | 0.868 | 4.76E-11 | 24 | *DEK* | Fibroblasts |
| 1.77E-18 | -0.95908 | 0.28 | 0.796 | 3.90E-14 | 24 | *BEX1* | Fibroblasts |
| 8.46E-17 | -0.9628 | 0.04 | 0.565 | 1.87E-12 | 24 | *PHYHIPL* | Fibroblasts |
| 5.24E-11 | -0.96569 | 0.2 | 0.59 | 1.16E-06 | 24 | *SOX11* | Fibroblasts |
| 8.00E-07 | -0.97994 | 0.027 | 0.294 | 0.01766 | 24 | *DCT* | Fibroblasts |
| 3.75E-11 | -0.98517 | 0.227 | 0.607 | 8.28E-07 | 24 | *RGS16* | Fibroblasts |
| 5.13E-15 | -0.99094 | 0.093 | 0.586 | 1.13E-10 | 24 | *SIX3* | Fibroblasts |
| 6.68E-16 | -0.99145 | 0.68 | 0.918 | 1.48E-11 | 24 | *RTN4* | Fibroblasts |
| 1.28E-09 | -1.00091 | 0.187 | 0.532 | 2.82E-05 | 24 | *DAPL1* | Fibroblasts |
| 3.45E-08 | -1.00604 | 0.013 | 0.313 | 0.000762 | 24 | *DIO3* | Fibroblasts |
| 2.51E-11 | -1.04339 | 0.293 | 0.627 | 5.54E-07 | 24 | *PLEKHA1* | Fibroblasts |
| 5.20E-22 | -1.08745 | 0.107 | 0.711 | 1.15E-17 | 24 | *GPM6A* | Fibroblasts |
| 3.13E-11 | -1.09321 | 0.067 | 0.466 | 6.92E-07 | 24 | *FAM57B* | Fibroblasts |
| 5.22E-12 | -1.09376 | 0.04 | 0.459 | 1.15E-07 | 24 | *CD24* | Fibroblasts |
| 2.21E-15 | -1.10127 | 0.213 | 0.652 | 4.87E-11 | 24 | *ENO2* | Fibroblasts |
| 3.40E-07 | -1.1267 | 0.16 | 0.423 | 0.007509 | 24 | *GNB3* | Fibroblasts |
| 1.22E-16 | -1.13332 | 0.16 | 0.641 | 2.69E-12 | 24 | *SYT1* | Fibroblasts |
| 2.81E-08 | -1.14967 | 0.253 | 0.524 | 0.000621 | 24 | *UNC119* | Fibroblasts |
| 3.60E-14 | -1.16468 | 0.027 | 0.502 | 7.94E-10 | 24 | *AMER2* | Fibroblasts |
| 3.36E-18 | -1.19994 | 0.04 | 0.595 | 7.41E-14 | 24 | *RORB* | Fibroblasts |
| 5.24E-07 | -1.21105 | 0.107 | 0.382 | 0.01156 | 24 | *PDE6H* | Fibroblasts |
| 5.56E-22 | -1.2134 | 0.093 | 0.705 | 1.23E-17 | 24 | *SIX6* | Fibroblasts |
| 3.27E-14 | -1.22924 | 0.067 | 0.529 | 7.21E-10 | 24 | *PTH2* | Fibroblasts |
| 2.97E-19 | -1.23545 | 0.08 | 0.647 | 6.55E-15 | 24 | *MAB21L1* | Fibroblasts |
| 4.92E-08 | -1.25702 | 0.04 | 0.335 | 0.001086 | 24 | *AIPL1* | Fibroblasts |
| 1.69E-19 | -1.29136 | 0.387 | 0.839 | 3.74E-15 | 24 | *TUBB2B* | Fibroblasts |
| 7.47E-06 | -1.31468 | 0.147 | 0.38 | 0.165048 | 24 | *RCVRN* | Fibroblasts |
| 1.90E-11 | -1.3357 | 0.053 | 0.438 | 4.19E-07 | 24 | *NEUROD1* | Fibroblasts |
| 4.17E-07 | -1.41145 | 0.067 | 0.346 | 0.0092 | 24 | *NRL* | Fibroblasts |
| 3.29E-11 | -1.5265 | 0.12 | 0.499 | 7.26E-07 | 24 | *SPP1* | Fibroblasts |
| 6.85E-15 | -1.56361 | 0.053 | 0.534 | 1.51E-10 | 24 | *SCG3* | Fibroblasts |
| 1.16E-07 | -1.58267 | 0.16 | 0.428 | 0.002569 | 24 | *PDC* | Fibroblasts |
| 6.23E-16 | -1.60025 | 0.187 | 0.667 | 1.38E-11 | 24 | *HES6* | Fibroblasts |
| 6.28E-17 | -1.62223 | 0.107 | 0.62 | 1.39E-12 | 24 | *FABP7* | Fibroblasts |
| 3.73E-09 | -1.6693 | 0.227 | 0.551 | 8.23E-05 | 24 | *SFRP2* | Fibroblasts |
| 5.85E-35 | -1.82709 | 0.56 | 0.977 | 1.29E-30 | 24 | *CKB* | Fibroblasts |
| 1.21E-18 | -1.8658 | 0.267 | 0.746 | 2.67E-14 | 24 | *CRABP1* | Fibroblasts |
| 1.60E-14 | -1.86587 | 0.107 | 0.577 | 3.54E-10 | 24 | *TRH* | Fibroblasts |
| 3.70E-39 | 1.569942 | 0.865 | 0.27 | 8.17E-35 | 25 | *CHGB* | NRPC/T3 |
| 1.78E-27 | 1.13461 | 0.689 | 0.213 | 3.92E-23 | 25 | *CHODL* | NRPC/T3 |
| 7.22E-30 | 0.956859 | 0.905 | 0.319 | 1.59E-25 | 25 | *SLC38A5* | NRPC/T3 |
| 9.24E-21 | 0.839633 | 0.973 | 0.645 | 2.04E-16 | 25 | *CSRP2* | NRPC/T3 |
| 6.17E-16 | 0.834581 | 0.568 | 0.226 | 1.36E-11 | 25 | *MAP2K6* | NRPC/T3 |
| 1.11E-25 | 0.820971 | 1 | 0.921 | 2.44E-21 | 25 | *HMGN2* | NRPC/T3 |
| 1.62E-22 | 0.807704 | 0.811 | 0.34 | 3.58E-18 | 25 | *KIF22* | NRPC/T3 |
| 6.06E-21 | 0.781868 | 0.946 | 0.62 | 1.34E-16 | 25 | *TYMS* | NRPC/T3 |
| 4.99E-23 | 0.774472 | 0.946 | 0.566 | 1.10E-18 | 25 | *PCNA* | NRPC/T3 |
| 3.62E-24 | 0.7713 | 0.946 | 0.419 | 8.00E-20 | 25 | *PMAIP1* | NRPC/T3 |
| 7.62E-13 | 0.765499 | 0.905 | 0.609 | 1.68E-08 | 25 | *FABP7* | NRPC/T3 |
| 7.15E-23 | 0.758141 | 0.892 | 0.369 | 1.58E-18 | 25 | *PCLAF* | NRPC/T3 |

| HOPX.1 | 2.23E-07 | 0.741927 | 0.284 | 0.107 | 0.004922 | 25 *HOPX* | NRPC/T3 |
| --- | --- | --- | --- | --- | --- | --- | --- |
| FAM111B. | 1.31E-22 | 0.736154 | 0.797 | 0.298 | 2.90E-18 | 25 *FAM111B* | NRPC/T3 |
| PTGDS.9 | 0.003919 | -0.7014 | 0.068 | 0.194 | 1 | 25 *PTGDS* | NRPC/T3 |
| TTYH1.13 | 0.000263 | -0.70414 | 0.284 | 0.456 | 1 | 25 *TTYH1* | NRPC/T3 |
| DKK3.12 | 0.000103 | -0.71641 | 0.297 | 0.49 | 1 | 25 *DKK3* | NRPC/T3 |
| APOE.8 | 0.001761 | -0.79739 | 0.338 | 0.452 | 1 | 25 *APOE* | NRPC/T3 |
| DIO3.17 | 3.41E-06 | -0.90792 | 0.068 | 0.312 | 0.075303 | 25 *DIO3* | NRPC/T3 |
| HES1.11 | 1.50E-08 | -0.93525 | 0.068 | 0.39 | 0.000331 | 25 *HES1* | NRPC/T3 |
| IFITM3.16 | 1.02E-05 | -0.93992 | 0.135 | 0.357 | 0.225733 | 25 *IFITM3* | NRPC/T3 |
| IFITM2.12 | 9.28E-07 | -0.94181 | 0.027 | 0.285 | 0.02049 | 25 *IFITM2* | NRPC/T3 |
| CCND1.12 | 1.47E-05 | -0.9867 | 0.243 | 0.455 | 0.323908 | 25 *CCND1* | NRPC/T3 |
| SOX2.14 | 1.26E-09 | -1.01059 | 0.068 | 0.414 | 2.79E-05 | 25 *SOX2* | NRPC/T3 |
| ZFP36L1.13 | 2.56E-08 | -1.04366 | 0.149 | 0.447 | 0.000565 | 25 *ZFP36L1* | NRPC/T3 |
| LAMP5.15 | 9.23E-05 | -1.04418 | 0.135 | 0.326 | 1 | 25 *LAMP5* | NRPC/T3 |
| FOS.12 | 7.13E-08 | -1.12808 | 0.324 | 0.561 | 0.001574 | 25 *FOS* | NRPC/T3 |
| COL1A2.12 | 6.67E-08 | -1.20036 | 0.081 | 0.376 | 0.001473 | 25 *COL1A2* | NRPC/T3 |
| SPP1.17 | 3.57E-06 | -1.36252 | 0.311 | 0.496 | 0.078724 | 25 *SPP1* | NRPC/T3 |
| VIM.15 | 5.08E-06 | -1.37374 | 0.838 | 0.802 | 0.112079 | 25 *VIM* | NRPC/T3 |
| CYP26A1.1 | 5.46E-06 | -1.68503 | 0.243 | 0.46 | 0.120464 | 25 *CYP26A1* | NRPC/T3 |
| SFRP2.19 | 1.28E-09 | -1.91837 | 0.23 | 0.551 | 2.84E-05 | 25 *SFRP2* | NRPC/T3 |
