# Supplementary material for: pRB-Depleted Pluripotent Stem Cell Retinal Organoids Recapitulate Cell State Transitions of Retinoblastoma Development and Suggest an Important Role for pRB in Retinal Cell Differentiation
Source: Stem Cells Transl Med. 2022 Mar 23;11(4):415–33. doi: 10.1093/stcltm/szac008 (PMC9052432; doi:10.1093/stcltm/szac008)
Supplement: szac008_suppl_Supplementary_Table_S5 [file szac008_suppl_supplementary_table_s5.docx]

| **Antibody** | **Species** | **Company** | **Cat. No** | **Concentration** |
| --- | --- | --- | --- | --- |
| Ap2α | Mouse | Santa Cruz Biotechology | SC-12726 | 1 in 200 |
| ARR3 | Rabbit | Novus Biologicals | NBP2-41249 | 1 in 100 |
| CASP3 | Rabbit | Cell Signaling | 9661S | 1 in 400 |
| CRX | Mouse | Abnova | H00001406-M02 | 1 in 200 |
| Ki67 | Mouse | BD Pharmingen | 550609 | 1 in 50 |
|  | Rabbit | Abcam | AB15580 | 1 in 200 |
| NRL (F2) | Mouse | Santa Cruz Biotechology | SC-374277 | 1 in 100 |
| OPN1SW | Rabbit | Millipore | AB5407 | 1 in 200 |
| OPN 1LW/MW | Rabbit | Millipore | AB5405 | 1 in 200 |
| PKC-α | Rabbit | Sigma | SAB4502354 | 1 in 50 |
| RB1 | Mouse | BD Pharmingen | 554136 | 1 in 400 |
| PROX1 | Mouse | Sigma | MAB5654 | 1 in 200 |
|  | Rabbit | Millipore | AB5475 | 1 in 1500 |
| Recoverin | Rabbit | Millipore | AB5585 | 1 in 1000 |
| RHO (Clone ID4) | Mouse | Millipore | MAB5356 | 1 in 200 |
| RHO | Rabbit | Abcam | AB59260 | 1 in 100 |
| RXRγ | Rabbit | Santa Cruz Biotechology | SC-555 | 1 in 200 |
| SNCG | Mouse | Abnova | H00006623-M01A | 1 in 500 |
|  | Rabbit | Abcam | AB55424 | 1 in 200 |
| Vimentin | Rabbit | Abcam | AB92547 | 1 in 400 |
| VSX2 | Rabbit | Sigma Atlas | HPA003436 | 1 in 50 |

**Table S5. List of primary antibodies used for immunofluorescence analysis.**
