# Supplementary material for: pRB-Depleted Pluripotent Stem Cell Retinal Organoids Recapitulate Cell State Transitions of Retinoblastoma Development and Suggest an Important Role for pRB in Retinal Cell Differentiation
Source: Stem Cells Transl Med. 2022 Mar 23;11(4):415–33. doi: 10.1093/stcltm/szac008 (PMC9052432; doi:10.1093/stcltm/szac008)
Supplement: szac008_suppl_Supplementary_Legends [file szac008_suppl_supplementary_legends.docx]

**Figure legends**

**Figure 1. Characterisation of H9 RB1-null derived retinal organoids. A.** Representative light microscopy images of retinal organoids differentiated from H9 control and RB1-null at day 35, 90 and 150 (scale bar = 100 µm). **B**. Representative western-blot analysis of pRB and related proteins steady-state levels in control (*RB1^+/+^*) and H9 RB1-null (*RB1^-/-^*) hESC organoids at day 35, 90 and 150 of differentiation; GAPDH was used as loading control. **C.** Representative immunofluorescence images of anti-pRB staining at day 35, 90 and 150 in control and RB1-null hESC organoids alongside quantification of pRB positive cells in H9 control. Insets show higher magnification images. Data are presented as mean ± SEM (n = 10 sections from each biological replicate). Scale bars; 50 µm.

**Figure 2.** **Immunohistochemical analysis of RB1-null hESC retinal organoids at day 35 and 90 of differentiation**. **A.** Bar charts showing the retinal cell-types and Ki67^+^ co-expressing fractions (left) and representative immunofluorescence analysis for CRX, SNCG and Ki67, counterstained with Hoechst (right) at day 35. **B.** Bar charts showing the percentage of Caspase-3^+^ cells (CASP3), retinal cell-types and Ki67^+^ co-expressing fractions (left) and representative immunofluorescence analysis for RXRγ, SNCG, PROX1, AP2α and Ki67, counterstained with Hoechst (right) at day 90. Data are presented as mean ± SEM (n = 10 sections from each biological replicate). White arrowheads point at co-localisation of Ki67 with the specified retinal marker. Scale bars; 50 µm.

**Figure 3. Tumorigenic characteristics of RB1-null hESC organoids. A.** Characterisation of RB1-null derived retinal organoids at day 150 of differentiation. Bar charts showing the percentage of Caspase-3^+^ cells (CASP3), the retinal cell-types and Ki67^+^ co-expressing fractions (left) and representative immunofluorescence analysis for RXRγ, SNCG, AP2α, RHO and Ki67, counterstained with Hoechst (right) at day 150 of differentiation. Data are presented as mean ± SEM (n = 10 sections from each biological replicate). White arrowheads point at co-localisation of Ki67 with the specified retinal marker. Scale bar; 50 µm. **B**. Soft agar colony formation assay showing ability of pRB-depleted retinal cells originating from day 90 RB1-null hESC retinal organoids to grow in suspension (bright field images, scale bar = 100 μm). **C**. Transmission electron microscopy of photoreceptor inner segments (IS), visible mitochondria (white arrowheads) and outer limiting-like membrane (black arrows) (scale bar; 1μm). **D.** Representative TEM example of mitotic cell in RB1-null organoid at day 200 of differentiation (left panel) followed by rosette-like structure (marked with dashed ellipse) shown in TEM images (middle panel) and H&E-stained sections in the right-hand side panel, followed by VSX2 and Ki67 immunofluorescence analysis; rosette-like structure marked with dashed ellipse (scale bars; 5 μm, 2 μm, 50 µm, 5 µm respectively).

**Figure 4. Single cell RNA-Seq reveals the presence of retinoma and Rb cells in H9 RB1-null organoids. A. and B.** UMAPs showing the cell clusters in control and RB1-null hESC organoids (A) and cell cycle distribution (B). **C**. UMAP of integrated control and H9 RB1-null hESC organoids with cell cluster annotations. **D**. Expression of key retinal cell type markers genes used to facilitate cluster definition in C overlaid onto the UMAPs. **E**. Percentages of cells in each cell cluster of control and RB1-null hESC organoids. **F**. Monocle-2 pseudotime showing progression from UPRCs to retinoma and Rb cell clusters. Cluster 11 was exempted from analysis because of low read number. RPCs: retinal progenitor cells, MGs: Müller glia cells, NRPCs: neurogenic RPCs, RGCs: retinal ganglion cells, UPRCs: cones with high expression of genes involved in unfolded protein response, RGCP: RGC precursor, T1, T2, T3: transient neurogenic progenitor populations.

**Figure 5. Characterisation of *RB1* patient-iPSC-derived organoids.**  **A.** Sequencing confirms the correction of c.2082delC mutation in the CRISPR-Cas9 corrected heterozygous *RB1* patient iPSC line and the introduction of the mutation in the second allele (homozygous mutant). Highlighted in yellow, the letter N, indicates heterozygosity of the original iPSC line caused by the single C deletion. C is marked by an arrow in wild-type sequence. **B.** Western-blot analysis of pRB steady-state level in iPSC lines (isogenic control, heterozygous and homozygous mutant for c.2082delC), GAPDH was used as loading control. **C.** Pluripotency test. The pluripotency plot window provides a visual representation of the tested samples in the analysis. The pluripotency and novelty x/y scatter plot combine the pluripotency score on the y-axis with the novelty score on the x-axis. The red and blue background hint to the empirical distribution of the pluripotent (red) and non-pluripotent (blue) samples in the reference data set. Samples were analysed using an algorithm that integrates gene expression data to authenticate pluripotency status. Samples were screened against samples in the stem cell database and given a pluripotency score (PluriCor) and novelty score (NovelCor), which are shown in the table. Pass shows a clear pluripotency signature. Fail means the samples are not pluripotent. A non-iPSC sample was used in this experiment to serve as a negative control for non-pluripotency. **D.** The whole genome view displays all somatic and sex chromosomes in one frame with high level copy number. The smooth signal plot (right y-axis) is the smoothing of the log2 ratios which depict the signal intensities of probes on the microarray. A value of 2 represents a normal copy number state (CN = 2), value of 3 represents chromosomal gain (CN = 3), value of 1 represents a chromosomal loss (CN = 1). The pink, green and yellow colours indicate the raw signal for each individual chromosome probe, while the blue signal represents the normalized probe signal which is used to identify copy number and aberrations (if any).

**Figure 6**. ***RB1* patient-specific** **derived retinal organoids**. **A.** Representative light microscopy images of retinal organoids differentiated from iPSC isogenic control, heterozygous and homozygous mutant for c.2082delC at day 35, 90 and 150, scale bar = 100 μm. **B.** Western-blot analysis of pRB and related proteins steady-state levels in control (*RB1^+/+^*), heterozygous (*RB1^+/-^*) and homozygous (*RB1^-/-^*) organoids at day 35, 90 and 150 of differentiation, GAPDH was used as loading control. Below, example immunostaining for pRB, counterstained with Hoechst at day 35 of differentiation, scale bar; 50 µm. **C.** Representative images of immunostaining for RXRγ and Ki67, counterstained with Hoechst at day 90 of differentiation alongside graphs depicting the percentage of RXRγ expressing cells and Ki67^+^ co-expression fractions. Data are presented as mean ± SEM (n = 9 sections from each biological replicate), scale bar; 50 µm.

**Figure 7**. **Single cell RNA-Seq of *RB1* patient-specific iPSC-derived retinal organoids.** UMAPs showing the cell clusters in control (*RB1^+/+^*), heterozygous (*RB1^+/-^*) and homozygous (*RB1^-/-^*) organoids (**A**) and cell cycle distribution (**B**). **C**. UMAP of integrated control (*RB1^+/+^*), heterozygous (*RB1^+/-^*) and homozygous (*RB1^-/-^*) organoids with cell cluster annotations. Cluster 17 was exempted from analysis because of low read number. **D**. Percentages of cells in each cell cluster of control *RB1^+/+^*, *RB1^+/-^* and *RB1^-/-^* organoids. **E**. UMAPS with overlays of representative marker gene expression.

**Figure 8.** **Assessment of clinically used chemotherapeutic agents for Rb treatment in control and RB1-null hESC organoids. A.** Bar graphs showing the percentage of proliferating cone precursors (RXRγ^+^Ki67^+^) in immunostained sections of treated organoids agent (Melphalan; 8, 16, 32 µM, Topotecan; 5, 10, 15, 150 µM, TW-37; 0.1, 0.5, 1, 10 µM alongside vehicle only sample; 0.1% DMSO). **B.** Apoptotic response (cleaved-caspase-3; CASP3) after application of chemotherapeutic agents. Data presented as mean ± SEM (n = 5 sections from each biological replicate).**C-D.** Representative immunostaining of CASP3 counterstained with Hoechst for control (C) and RB1-null (D) hESC organoids; vehicle only, Melphalan 32 µM, Topotecan 15 µM, and TW-37 10 µM. Scale bars; 50 µm.

**Supplementary information**

**Figure S1**. **Immunofluorescence analyses of control hESC- derived organoids for pRB expression at day 35 and 90 of differentiation**. **A**. Co-staining of pRB with CRX, VSX2, SNCG or Ki67 in day 35 retinal organoids. Scale bars; 50 µm. **B**. Co-staining of pRB with RCVRN, ARR3, VSX2, AP2α, PKCα, PROX1, Vimentin, RXRƴ, CASP3, Ki67, counterstained with Hoechst. White arrowheads point at co-localisation of pRB with specified marker. Scale bars; 50 µm.

**Figure S2**. **Immunohistochemical analyses of control hESC-derived organoids for pRB expression at day 150 of differentiation**. Co-staining of pRB with RCVRN, ARR3, RXRγ, VSX2, PKCα, AP2α, PROX1, OPN1LW/MW, RHO, Vimentin, CASP3, Ki67, counterstained with Hoechst in day 150 H9 control retinal organoids, scale bars; 50μm. White arrowheads point at co-localisation of pRB with specified marker. Scale bars; 50 µm.

**Figure S3. Representative immunofluorescent staining of VSX2 and Ki67, counterstained with Hoechst for control and RB1-null hESC organoids at day 35 of differentiation** (**A**) and ARR3, VSX2, CASP3, and Ki67 at day 90 (**B**). White arrowheads point at co-localisation of Ki67 with specified marker. Insets show higher magnification images. Scale bars; 50 µm. **C.** Staining TUNEL-positive cells in H9 control and RB1-null organoids (day 90 of differentiation) sections alongside enumeration of positive cells. Scale bars; 50 µm. Data presented as mean ± SEM, n=5.

**Figure S4**. **Representative immunofluorescent staining of neural retina cell type markers** VSX2, ARR3, OPN1SW, OPN1LW/MW, PROX1, PKCα and apoptotic (CASP3) with proliferation (Ki67) marker, counterstained with Hoechst for control and RB1-null hESC organoids at day 150. White arrowheads point at co-localisation of Ki67 with specified marker. Insets show higher magnification images. Scale bars; 50 µm.

**Figure S5**. **Representative immunofluorescent staining of (A) SNCG and Ki67 and (B) RXRγ or SNCG with Ki67** counterstained with Hoechst for control and RB1-null hESC organoids at day 35 and 90 respectively. White arrowheads point at co-localisation of Ki67 with specified marker. Insets show higher magnification images. Scale bars; 50 µm.

**Figure S6**. **Representative immunofluorescent staining of RXRγ or SNCG with Ki67** counterstained with Hoechst for control and RB1-null hESC organoids at day 150 (**A**). White arrowheads point at co-localisation of Ki67 with specified marker. Insets show higher magnification images. Scale bars; 50 µm. **B**. Average number of mitochondria in photoreceptor inner segment (on the left), number of cristae (on the right) in RB1-null organoids versus control. Data are presented as mean ± SEM (n = 3 – 6 images).

**Figure S7.** **Expression of key Rb and retinoma marker genes used to facilitate cluster definition in Figure 4C overlaid onto the UMAPs. A**. Rb markers; *CDC25A*, *CDC25C*, *CHEK1*, *DEK*, *E2F3*, *KIF14*, *MYCN*, *PRC1*, *SKP2*, *SYK*, *TOP2A*, *UBE2C*. **B**. Retinoma markers; *CDCA7*, *HELLS*, *PCNA*, *MKi67*.

**Figure S8.** **pRB inactivation in hESC-retinal organoids leads to dysregulation of retinal neurogenesis. A**. Expression of *RXRγ* (cone precursor marker) and *GAP43* (RGC marker) or *ONECUT2* (horizontal cells) in panel **C**, overlaid onto the UMAPs of control and RB1-null hESC organoids. **B**. Co-staining of RXRγ with SNCG (RGC marker) or PROX1 (horizontal cell marker) in panel **D**, counterstained with Hoechst for control and RB1-null hESC organoids at day 90. White arrowheads point at co-localisation of specified markers. Insets show higher magnification images. Scale bars; 50 µm.

**Figure S9.** RNA expression of *RXRγ*, *GAP43, MKi67* (**A**) and *RXRγ*, *ONECUT1, MKi67* (**B**) and their co-expression in Rb tumours. Raw expression values were normalised, log transformed and summarised. The size of the dots indicates the proportion of cells, while the colour indicates the mean expression.

**Figure S10.** **Representative immunofluorescent staining of neural retina cell type markers AP2α, SNCG or PROX1 with apoptotic marker CASP3 in H9 RB1-null and control organoids at day 90 of differentiation.** PROX1, CASP3 immunostainings were performed on adjacent sections. White arrowheads point at co-localisation of CASP3 with specified marker. Insets show higher magnification images. Scale bars; 50 µm.

**Figure S11**. **Patient-derived iPSC lines with heterozygous c.2082delC in *RB1*. A**. iPSC reprogramming from peripheral blood cells of *RB1* patient using Sendai Virus method. Scale bars; 100 µm. **B.** Example of DNA sequencing of iPSC clones generated from *RB1* patients’ blood sample**.** WT3 is a control iPSC line with wild-type *RB1*, characters highlighted in yellow indicate heterozygosity in alleles caused by the DNA frameshift, marked by an arrow is wild-type nucleotide. **C.** Western-blot analysis of pRB steady-state level in patient-specific iPSC lines, GAPDH was used as loading control.

**Figure S12.** **CRISPR/Cas9 edition of c.2082delC *RB1* mutation in heterozygous *RB1* iPSC line to obtain *RB1* gene-corrected isogenic control and homozygous mutant**. Sequencing chromatogram depicts highlighted in yellow, the letter M, indicating heterozygosity in alleles caused by the silent mutation (in red) introduced in the PAM sequence (in blue) of ssODN, W and R represent heterozygous changes in *RB1* gene around the cut site. Rectangle marks the mutation site.

**Figure S13.** **Immunofluorescence characterisation of patient iPSC (c.2082delC) derived retinal organoids at day 35 of differentiation**. **A.** Representative images of immunostaining for VSX2, CRX, SNCG, pRB and Ki67, counterstained with Hoechst. **B.** Bar graphs showing percentage of retinal cell types, pRB^+^ and Ki67^+^ co-expressing fractions. Data are presented as mean ± SEM (n = 9 sections from each biological replicate). White arrowheads point at co-localisation of Ki67 with the specified retinal marker; scale bars; 50 µm.

**Figure S14.** **Immunofluorescence characterisation of patient iPSC (c.2082delC) derived retinal organoids at day 90 of differentiation**. Representative immunostaining of retinal cell types; PROX1, AP2α, VSX2, SNCG, NRL, ARR3, VIMENTIN, and apoptotic (CASP3) cells as well as pRB expressing cells in combination with proliferation marker (Ki67^+^) counterstained with Hoechst. Scale bars; 50 µm. **B.** Bar graphs showing percentage of Caspase-3 ^+^ cells (CASP3), retinal cell types, pRB and Ki67^+^ co-expressing fractions. Data are presented as mean ± SEM (n = 9 sections from each biological replicate). White arrowheads point at co-localisation of Ki67 with the specified retinal marker; scale bars: 50 µm. Insets show higher magnification images. **C**. Graph depicting the percentage of TUNEL-positive cells in patient iPSC derived organoids. Data presented as mean ± SEM, n=5.

**Figure S15.** **Immunofluorescence characterisation of patient iPSC (c.2082delC) derived retinal organoids at day 150 of differentiation and cellular transformation *in vitro*. A.** Representative immunostaining of PROX1, AP2α, VSX2, SNCG, RXRƴ, ARR3, CASP3 and pRB expressing cells in combination with proliferation marker (Ki67^+^), counterstained with Hoechst. **B.** Bar graphs showing the percentage of Caspase-3^+^ (CASP3), retinal cell types, pRB and Ki67^+^ co-expressing fractions. Data are presented as mean ± SEM (n = 9 sections from each biological replicate). White arrowheads point at co-localisation of Ki67 with the specified retinal marker, pRB or Caspase-3; scale bars: 50µm. Insets show higher magnification images. **C.** Soft agar colony formation assay showing the ability of *RB1^-/-^* cells originating from day 90 retinal organoids to grow in suspension (bright field images, scale bar; 100 μm) **D.** Transmission electron microscopy of photoreceptor inner segments (IS), visible mitochondria (white arrowheads) and outer limiting-like membrane (black arrows) (scale bar; *RB1^+/+^*; 1 μm, *RB1^+/-^;* 2 μm, *RB1^-/-^*; 2 μm). **E**. Average number of mitochondria in photoreceptor inner segment (on the left), number of cristae (on the right) in iPSC patient-derived organoids. Data are presented as mean ± SEM (n = 6). **F.** Representative image of mitotic cell revealed by TEM in *RB1^-/-^* organoid at day 200 of differentiation followed by rosette-like structure (marked with dashed ellipse) shown in H&E-stained sections (scale bar; 2 μm, 50 µm), below VSX2 and Ki67 immunofluorescence analysis; rosette-like structure marked with dashed ellipse (scale bar; 5µm).

**Figure S16. Expression of key Rb marker genes (*CCNE2*, *DEK*, *MCM3*, *NUSAP1*, *SYK*) used to facilitate cluster definition in Figure 7C overlaid onto the UMAPs.**

**Figure S17**. **Representative immunofluorescent staining of neural retina cell type markers SNCG or PROX1 with apoptotic marker CASP3 in patient iPSC-derived organoids at day 90 of differentiation.** PROX1, CASP3 immunostainings were performed on adjacent sections. White arrowheads point at co-localisation of CASP3 with specified marker. Insets show higher magnification images. Scale bars; 50 µm.

**Figure S18**. **pRB inactivation leads to enrichment of heterogeneous subpopulations of cells in iPSC patient-derived retinal organoids. A**. Expression of *RXRγ* (cone precursor marker) and *ELAVL3* (RGC marker) overlaid onto the UMAPs of *RB1^+/+^*, *RB1^+/-^*, *RB1^-/-^* retinal organoids. **B**. Co-staining of RXRγ (cone precursor marker) with SNCG (RGC marker) or **C**. PROX1 (horizontal cell marker) counterstained with Hoechst for *RB1^+/+^*, *RB1^+/-^*, *RB1^-/-^*. White arrowheads point at co-localisation of specified markers. Insets show higher magnification images. Scale bars; 50 µm.

**Figure S19**. **Comparison of RB1-null hESC to homozygous patient specific *RB1^-/-^* retinal organoids**. UMAP showing the cell clusters in the integrated data set (**A**) and percentages of cells in each cell cluster (**B**).

**Figure S20.** **Assessment of clinically used chemotherapeutic agents for Rb treatment in iPSC patient-derived *RB1^-/-^* and isogenic control *RB1^+/+^* organoids.** **A.** Bar graphs showing the percentage of proliferating cone precursors (RXRγ^+^Ki67^+^) in immunostained sections of treated organoids (Melphalan; 8, 16, 32 µM, Topotecan; 5, 10, 15, 150 µM, TW-37; 0.1, 0.5, 1,10 µM alongside vehicle only sample; 0.1% DMSO). **B.** Apoptotic response (cleaved-caspase-3; CASP3) after application of chemotherapeutic agents. Data presented as mean ± SEM (n = 5 sections from each biological replicate). **C-D.** Representative immunostaining of CASP3 counterstained with Hoechst for *RB1^+/+^* (C) and *RB1^-/-^* (D) organoids; vehicle only, Melphalan 32 µM, Topotecan 15 µM, and TW-37 10 µM. Scale bars; 50 µm.

**Figure S21**. Representative images from secondary only IF control experiments. Scale bars; 50 µm.

**Table S1. A full list of highly and differentially expressed genes between the 22 clusters identified in the H9 hESC-derived retinal organoids.**

**Table S2. Off-target sequences for c.2082delC in *RB1*.** Listed are primers used to screen off-target sites for gRNAs to obtain isogenic WT and homozygous mutant from heterozygous iPSC line. R in sequencing result no VII, was confirmed heterozygosity originating from the patient.

**Table S3**. **A full list of highly and differentially expressed genes between the 35 clusters identified in the iPSC-derived retinal organoids.**

**Table S4. A full list of highly and differentially expressed genes between the 25 clusters found in the integrated dataset comprised of H9 RB1- null and patient homozygous organoids.**

**Table S5. List of primary antibodies used for immunofluorescence analysis.**

**Table S6. List of secondary antibodies used for immunofluorescence analysis**.
